# A cheap metal catalyzed ring expansion/cross-coupling cascade: a new route to functionalized medium-sized and macrolactones†

**DOI:** 10.1039/d2sc06157k

**Published:** 2023-04-17

**Authors:** Shuai Liu, Pengchen Ma, Lu Zhang, Shenyu Shen, Hong-Jie Miao, Le Liu, K. N. Houk, Xin-Hua Duan, Li-Na Guo

**Affiliations:** a Department of Chemistry, School of Chemistry, Xi'an Key Laboratory of Sustainable Energy Material Chemistry and Engineering Research Center of Energy Storage Materials and Devices, Ministry of Education, Xi'an Jiaotong University Xi'an 710049 China duanxh@xjtu.edu.cn guoln81@xjtu.edu.cn; b Department of Chemistry and Biochemistry, University of California Los Angeles California 90095-1569 USA houk@chem.ucla.edu

## Abstract

An efficient alkoxyl radical-triggered ring expansion/cross-coupling cascade was developed under cheap metal catalysis. Through the metal-catalyzed radical relay strategy, a wide range of medium-sized lactones (9–11 membered) and macrolactones (12, 13, 15, 18, and 19-membered) were constructed in moderate to good yields, along with diverse functional groups including CN, N_3_, SCN, and X groups installed concurrently. Density functional theory (DFT) calculations revealed that reductive elimination of the cycloalkyl-Cu(iii) species is a more favorable reaction pathway for the cross-coupling step. Based on the results of experiments and DFT, a Cu(i)/Cu(ii)/Cu(iii) catalytic cycle is proposed for this tandem reaction.

## Abstract 2

A Cu or Fe-catalyzed radical ring expansion/coupling cascade was developed. It provides a new access to medium-sized and macrolactones with CN, N_3_, SCN, and X groups. DFT calculations suggest a Cu(i)/Cu(ii)/Cu(iii) catalytic cycle for the cyanation.

## Graphical abstract

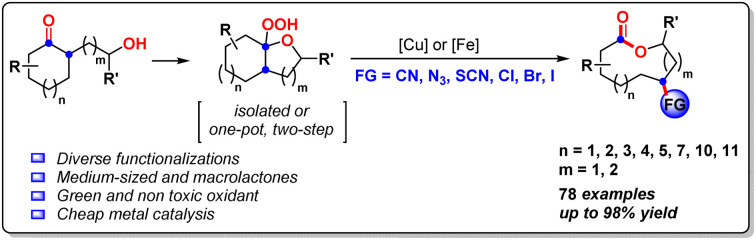

## 1 Introduction

**Fig. 1:**
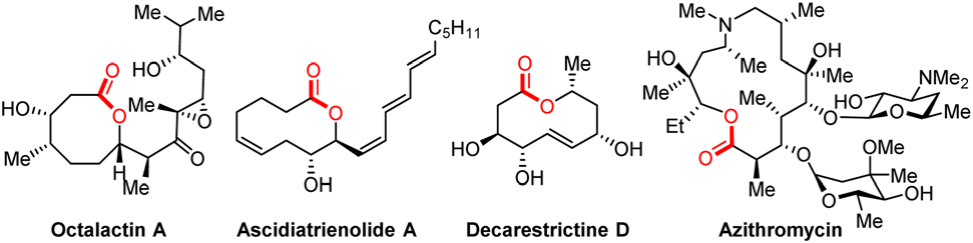
Examples of drugs and natural products containing a macrolactone framework.

**Scheme 1:**
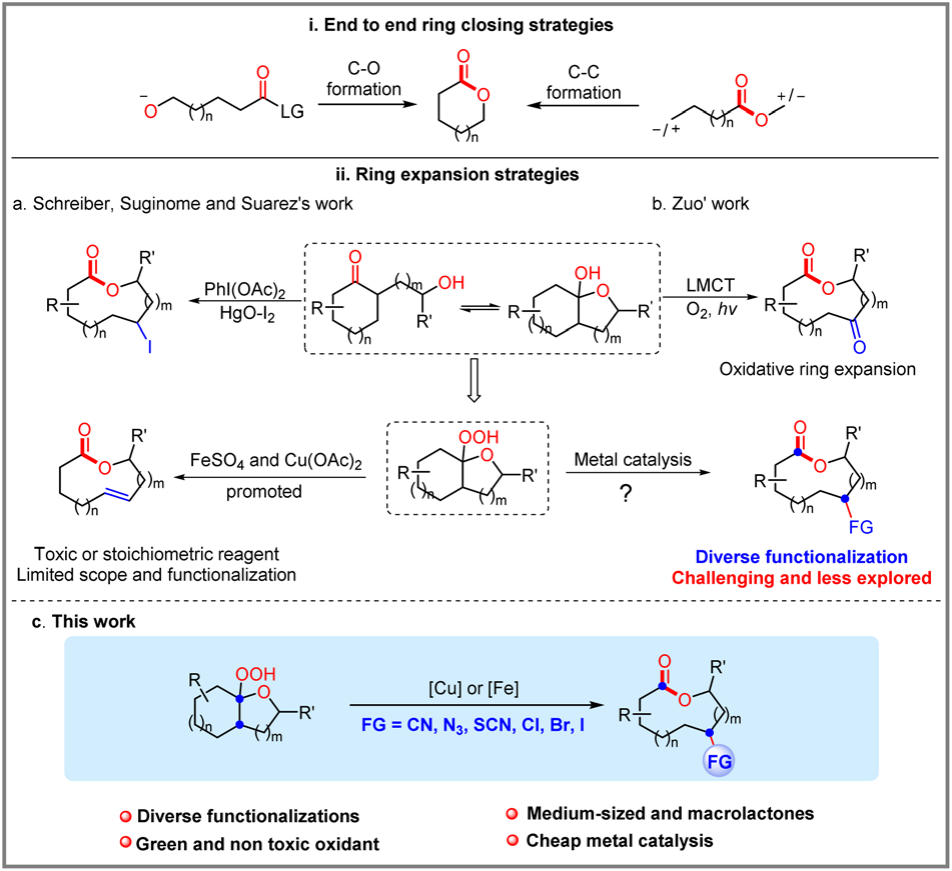
Diverse strategies for macrolactones.

Medium-sized lactones and macrolactones are structural cores of many bioactive pharmaceuticals, agrochemicals, and natural products (Fig. 1).^1^ As a consequence, their synthesis has attracted sustained interest from chemists, and diverse strategies have been developed over the past few decades.^2^ In this field, one of the most classical strategies is based on the end-to-end ring closing reactions, including transition metal-catalysed couplings, NHC-catalysed cyclizations, electrophilic halogenations, *etc.* wherein the C–O bond formation or C–C bond formation have been designed as the final step to realize cyclization (Scheme 1(i)).^2*f*,3^ Although significant progress has been made by means of transition metal catalysis, it is still challenging to achieve macrolactonization due to the obstacles of entropy and the competing intermolecular coupling side reactions.^4^ In contrast to the cyclization strategy, the ring expansion strategy represents another promising and high-efficiency alternative to avoid the abovementioned problems. Over the past few years, both ionic-type and the radical triggered ring expansion reactions have received much attention from the chemical community.^5^ Among them, alkoxyl radical-triggered fragmentation of fused bicyclic systems has emerged as an efficient strategy for the construction of medium-sized lactones and macrolactones (Scheme 1(ii)). In the 1980s, Schreiber, Suginome and E. Suárez *et al.* respectively disclosed several impressive examples for oxidative fragmentation of hemiketals, which offered the olefinic- and iodo-substituted medium-sized lactones (Scheme 1(iia)).^6^ Later on, Posner and Maio *et al.* successfully applied this strategy to the synthesis of natural (−)-phoracantholide-J and (+)-cis-lauthisan.^7^ However, these methods still suffer from requirement of toxic reagents (HgO) or stoichiometric amounts of organic oxidants (PhI(OAc)_2_). In addition, only few examples were investigated. In recent years, a rapid development of alkoxyl radical triggered C–C bond cleavage has occurred, but focusing on the ring-opening reactions.^8^ For the ring expansion aspect, the group of Zuo disclosed an elegant photocatalytic aerobic oxidative ring expansion of α-hydroxyalkylketones to the keto-macrolactones through the LMCT process (Scheme 1(iib)),^9^ whereas it is limited to introducing a carbonyl group into the macrolactone framework. Despite this impressive success in the radical ring expansion strategy, diverse functionalization based on the nascent carbon radical of ring expansion is still challenging and less explored. Therefore, exploration of new catalytic systems and chemical transformations is still highly desirable and rewarding to carry forward the ring expansion strategy for the diverse functionalized macrolactone synthesis.

In recent years, radical mediated C–C bond cleavage has emerged as a powerful platform for the C–C and C–X bond formation, taking advantage of the broad radical precursors, diverse mild catalytic system, as well as excellent selectivity and functional group tolerance.^10^ In these reactions, the generated carbon-centered radical intermediates could undergo diverse chemical transformations, including radical–radical coupling, radical addition and so on. As we know, the transition metal catalyzed radical relay strategy offered an attractive avenue for radical transformations.^11^ Recently, the groups of Liu, Stahl and others disclosed some elegant examples of C(sp^3^)–H functionalization reactions through the copper catalyzed radical relay strategy.^12^ Inspired by these studies and our previous studies,^13^ we intend to challenge the diverse synthesis of functionalized macrolactones through a ring expansion/cross-coupling cascade based on the transition metal catalyzed radical relay strategy (Scheme 1(iic)). Therein, the reactive transition metal species (with different valence states) not only serve as a SET reagent but also capture the nascent carbon-centered radical to form new chemical bonds.^11^

Herein, we disclose a range of efficient copper or iron-catalyzed ring expansion/cross-coupling cascade of hemiketal hydroperoxides with different nucleophiles (TMSCN, TMSN_3_, NH_4_SCN, and HX), which affords a variety of functionalized medium-sized lactones and macrolactones in good to excellent yields under mild conditions.

## 2 Results and discussion

Initially, the hemiketal hydroperoxide 1a and TMSCN were elected as model substrates to find the optimal conditions under copper catalysis (Table 1). Luckily, the ring expansion/cyanation of 1a proceeded efficiently by using CuI (5 mol%) as the catalyst and 1,10-phen (5 mol%) as the ligand in THF at ambient temperature, affording the desired cyano-substituted medium-sized lactone 2a in 88% yield.^14^ Solvent screening revealed that MeOH was the optimal solvent, yielding 2a in 98% yield (entries 1–4). Other copper catalysts such as CuOTf and Cu(OAc)_2_ were less effective than CuI, while an iron catalyst such as Fe(OTf)_2_ was totally ineffective (entries 5–7). The screening of ligands indicated that 1,10-phen gave better yield than 2,2′-bpy (entry 3 *vs.* entry 8). The control experiments revealed that the ligand is beneficial to obtain a higher yield and copper catalyst was essential for the success of this transformation (entries 9 and 10). Surprisingly, it was found that the reaction could be completed in 10 min, giving a 95% isolated yield of 2a (entry 11).

**Table 1:** Optimization of the ring expansion/cyanation of 1a with TMSCN^a^

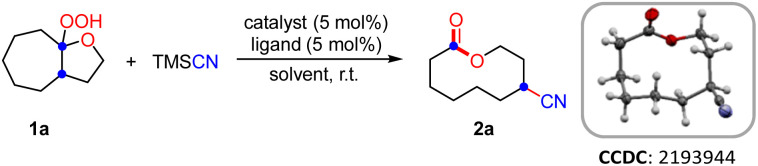				
Entry	Catalyst (mol%)	Solvent	Ligand	Yield (%)
1	CuI	THF	1,10-Phen	88
2	CuI	DMF	1,10-Phen	78
3	CuI	MeOH	1,10-Phen	98(95)^b^
4	CuI	Toluene	1,10-Phen	14
5	CuOTf	MeOH	1,10-Phen	40
6	Cu(OAc)_2_	MeOH	1,10-Phen	12
7	Fe(OTf)_2_	MeOH	1,10-Phen	N.R.
8	CuI	MeOH	2,2′-bpy	70
9	CuI	MeOH	—	40
10	—	MeOH	1,10-Phen	N.R.
11	CuI	MeOH	1,10-Phen	95^b^^,^^c^

a Reaction conditions: 1a (0.2 mmol, 1.0 equiv.), TMSCN (0.3 mmol, 1.5 equiv.), catalyst (0.01 mmol, 5 mol%), ligand (0.01 mmol, 5 mol%), solvent (2.0 mL), at 25 °C for 12 h under N_2_. Yields were determined by GC-FID analysis using dodecane as the internal standard.

b Isolated yields.

c Reaction for 10 min.

**Scheme 2:**
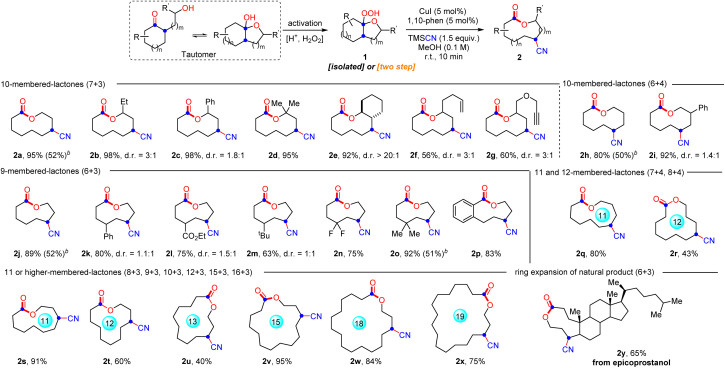
Scope of the ring expansion/cyanation.^*a*^ Reaction conditions A: 1 (0.2 mmol, 1.0 equiv.), TMSCN (0.3 mmol, 1.5 equiv.), CuI (0.01 mmol, 5 mol%) and 1,10-phen (0.01 mmol, 5 mol%) in MeOH (2.0 mL) at 25 °C for 10 min under N_2_. Isolated yields.^*b*^ Total yield through a two-step telescoped procedure is given in parentheses.

With the optimal conditions in hand, the generality and limitations of hemiketal peroxides 1 for this ring expansion/cyanation reaction were evaluated (Scheme 2). Substrates derived from a variety of strained and unstrained cycloalkanones with oxiranes were all amenable to afford the cyano-substituted medium-sized lactones (9 to 11-membered) and macrolactones (12 to 19-membered) in moderate to good yields (2a–2i, 2j–2p, 2s, 2r and 2t–2x). The ring strain did not show obvious impact on the reaction efficiency (2a, 2h and 2s–2x). Synthetically useful functional groups which existed in the carbocycle framework such as alkenyl (2f), alkynyl (2g), ester (2l), and halogen (2n) groups survived well in this transformation. Substrates synthesized from 2-substituted oxiranes also reacted smoothly to produce the desired lactones 2b–2g in good yields. In addition, substrates derived from six, seven and eight-membered cyclic ketones with oxetanes furnished the four-atom ring enlarged lactones 2h, 2i, 2q and 2r in moderate to good yields. Remarkably, natural epicoprostanol could also be modified to afford the ring expansion/cyanation product 2y in good yield, highlighting the potential applications of our protocol in the late-stage functionalization of complex molecules. Finally, to simplify the synthetic procedure, a telescoped protocol was tested. Satisfactorily, the two-step reactions of α-hydroxyalkylketones also proceeded smoothly to deliver the target lactones in moderate yields (2a, 2h, 2j and 2o, for details, see the ESI†). Finally, it is worth mentioning that changing the 1,10-phen to other ligands, such as d*t*bpy and Biox ligands failed to improve the d.r. value of 2b, but resulted in a decreased yield of 2b. In addition, with the reaction of 1p as an example, the use of chiral ligands instead of 1,10-phen did not improve the reaction enantioselectivity (for details, see the ESI,† S-10).

**Scheme 3:**
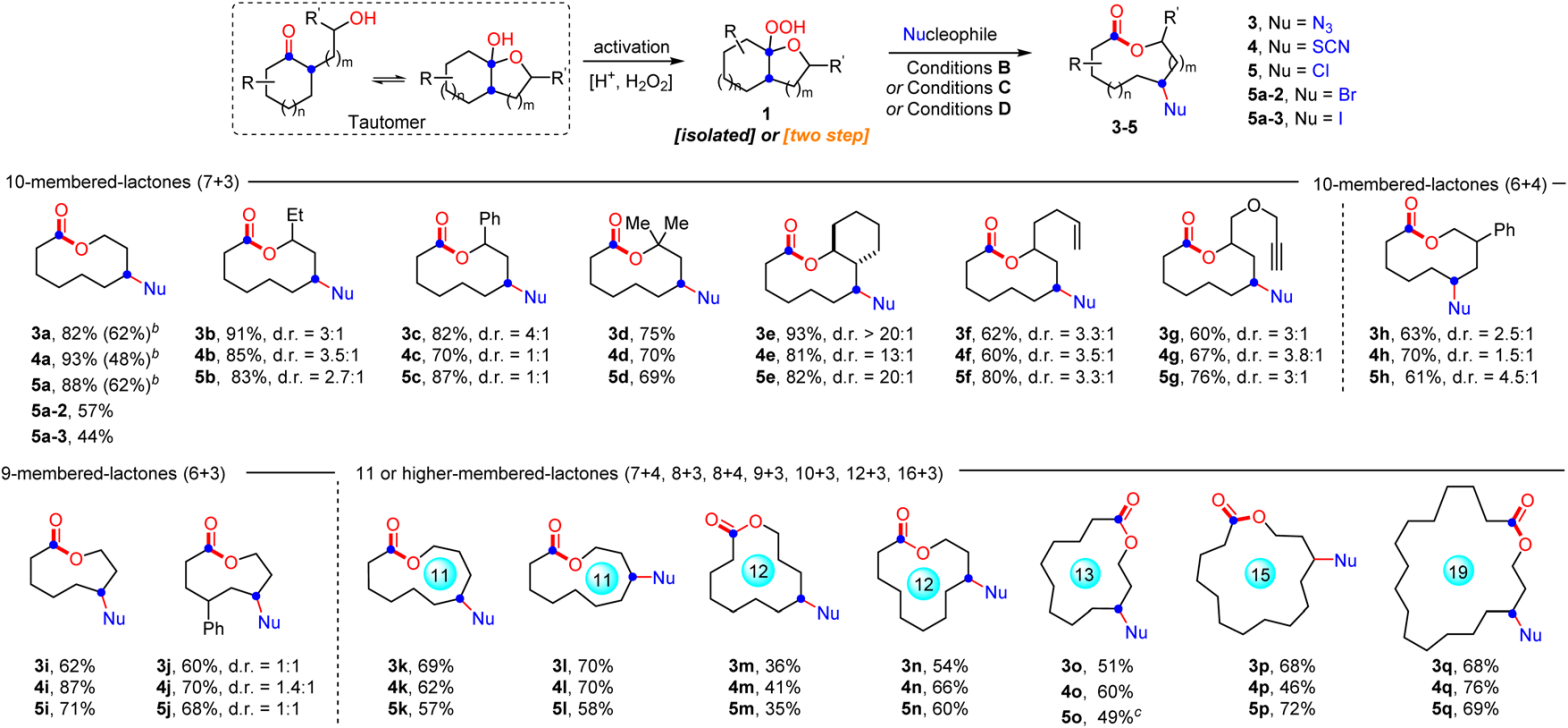
Scope of other ring expansion/functionalizations.^*a*^ Reaction conditions B for 3 : 1 (0.2 mmol, 1.0 equiv.), TMSN_3_ (0.3 mmol, 1.5 equiv.) and Fe(OTf)_2_ (0.01 mmol, 5 mol%) in EtOAc (2.0 mL) at 25 °C for 10 min under N_2_. Isolated yields. Reaction conditions C for 4 : 1 (0.2 mmol, 1.0 equiv.), NH_4_SCN (0.3 mmol, 1.5 equiv.) and CuCl (0.01 mmol, 5 mol%) in MeCN (2.0 mL) at 25 °C for 1 h under N_2_. Isolated yields. Reaction conditions D for 5 : 1 (0.2 mmol, 1.0 equiv.), HCl (36%, aq.) or HBr (40%, aq.) or HI (55–58%, aq.) (0.4 mmol, 2.0 equiv.), and CuCl (0.01 mmol, 5 mol%), in NMP (2.0 mL) at 25 °C for 10 min under N_2_. Isolated yields.^*b*^ Total yield through a two-step telescoped procedure is given in parentheses.^*c*^ 2.0 equiv. MgCl_2_ as the chlorine source.

**Scheme 4:**
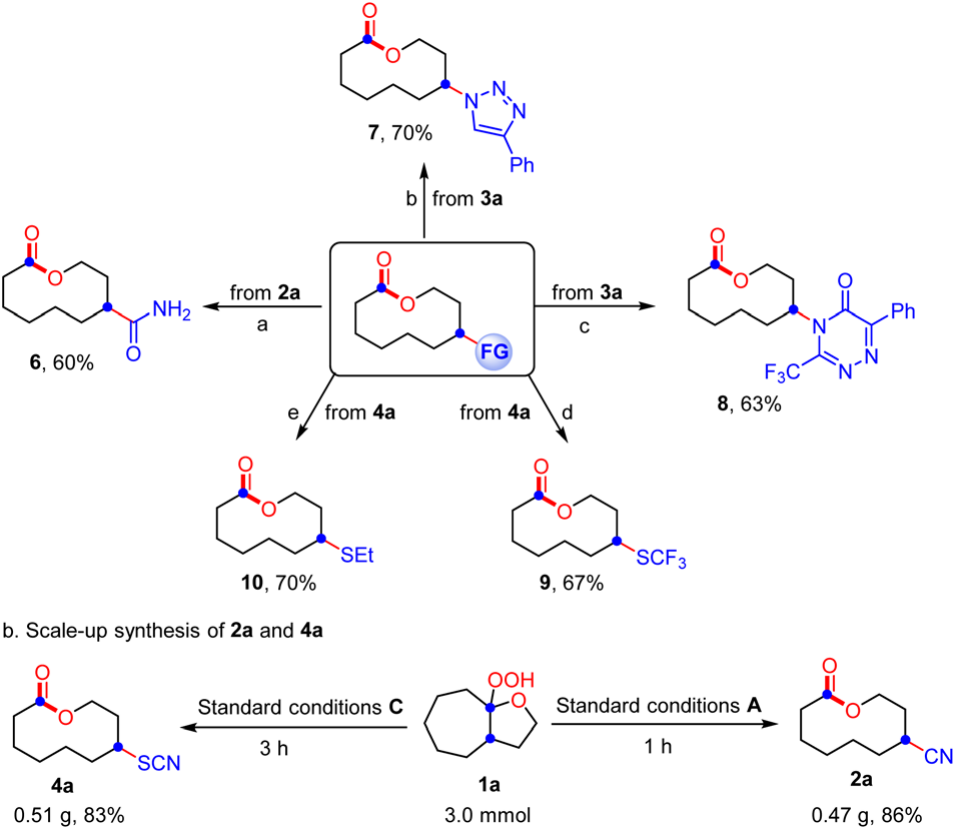
Diverse derivatizations of lactone products and scale-up synthesis.^*a*^ Reaction conditions: 2a (0.2 mmol, 1.0 equiv.), Cu(OAc)_2_ (0.004 mmol, 2 mol%), NEt_2_OH (0.6 mmol, 2.0 equiv.), H_2_O (0.1 M), 25 °C, 3 h under N_2_.^*b*^3a (0.2 mmol, 1.0 equiv.), phenylacetylene (0.4 mmol, 2.0 equiv.), CuSO_4_ (0.04 mmol, 20 mol%), sodium ascorbate (0.08 mmol, 40 mol%), *t*-BuOH/H_2_O (1 : 1, 0.1 M), 25 °C, 24 h under N_2_.^*c*^3a (0.2 mmol, 1.0 equiv.), phenylacetylene (0.4 mmol, 2.0 equiv.), CuI (0.01 mmol, 5 mol%), Et_3_N (0.4 mmol, 2.0 equiv.), (CF_3_CO)_2_O (0.3 mmol, 1.5 equiv.), THF (0.1 M), 25 °C, 24 h under N_2_.^*d*^4a (0.2 mmol, 1.0 equiv.), TMSCF_3_ (0.4 mmol, 2.0 equiv.), Cs_2_CO_3_ (0.4 mmol, 2.0 equiv.), MeCN (0.1 M), 25 °C, 16 h.^*e*^4a (0.2 mmol, 1.0 equiv.), EtMgBr (0.6 mmol, 3.0 equiv.), THF (0.1 M), −40 °C, 4 h under N_2_.

Subsequently, the ring expansion/cross-coupling cascade with other useful nucleophiles was also examined. Satisfactorily, TMSN_3_, NH_4_SCN and HX all were efficient nucleophiles, affording the corresponding N_3_-substituted, SCN-substituted and halogenated medium-sized lactones and macrolactones in moderate to good yields under slightly modified reaction conditions (Scheme 3). For TMSN_3_, the iron catalyst showed much better catalytic efficiency than the copper catalyst, while the copper catalyst is more efficient for the nucleophiles NH_4_SCN and HX. Notably, the ligand was unnecessary for these transformations. Undoubtedly, the success of incorporating these functional groups provides opportunities for further functionalization of the lactones. As expected, it was found that the functional groups installed into the lactone products can be easily converted to diverse functional groups in good yields, which demonstrated the great significance of this ring expansion/cross-coupling cascade (Scheme 4(a)). For example, the CN-containing lactone 2a could be hydrolyzed selectively to give the amide 6 in 60% yield. The N_3_-containing lactone 3a underwent the copper(i)-catalyzed [3 + 2] cycloaddition reaction with phenylacetylene smoothly to afford 1,2,3-triazole 7 in 70% yield. In the presence of trifluoroacetic anhydride, a copper(i)-catalyzed interrupted click reaction also worked to deliver the 3-trifluoromethyl-substituted 1,2,4-triazinone 8 in 63% yield. The SCN-containing lactone 4a could also be converted into the CF_3_S- or EtS-substituted lactones 9 and 10 in good yields. In addition, the scale-up synthesis of 2a and 4a was also conducted, respectively. When the reactions were carried out on a 3.0 mmol scale, both gave good yields (Scheme 4(b)). However, other nucleophiles such as alcohol, phenol and thioalcohol failed to provide the desired products (not shown).

**Scheme 5:**
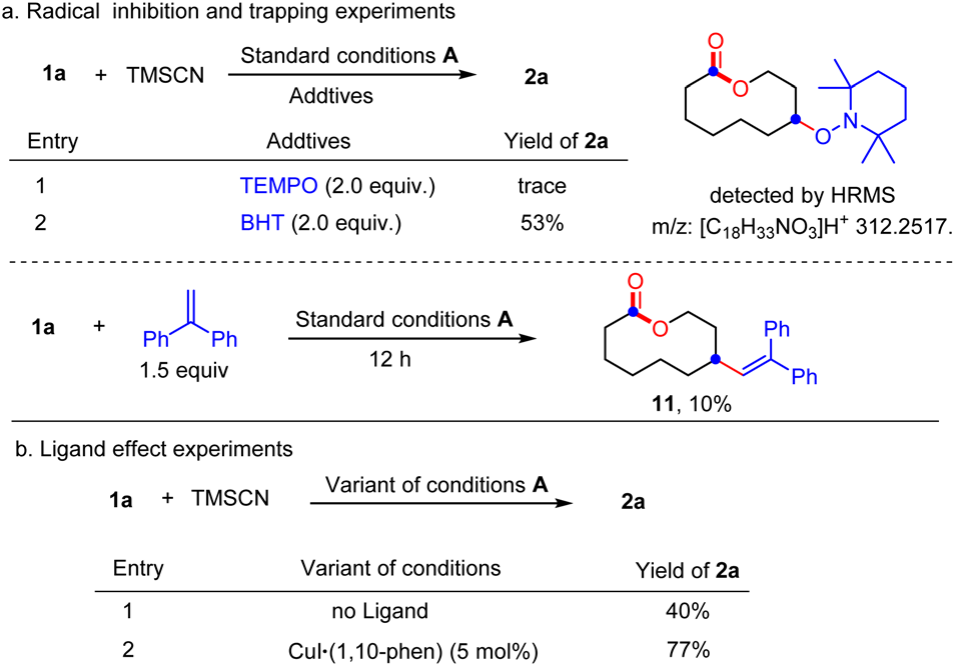
Mechanism studies.

**Fig. 2:**
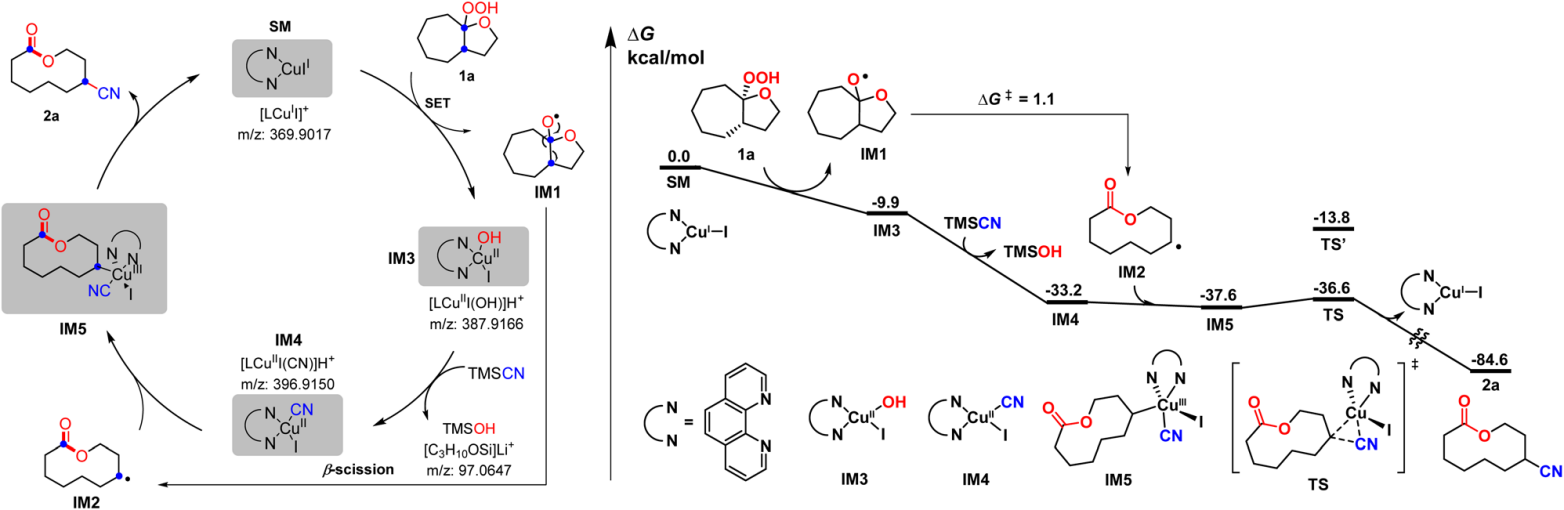
Proposed mechanism and computed energy profile for the Cu-catalyzed ring expansion/cyanation reaction of SM to 2a. Energies are in kcal mol^−1^.

**Fig. 3:**
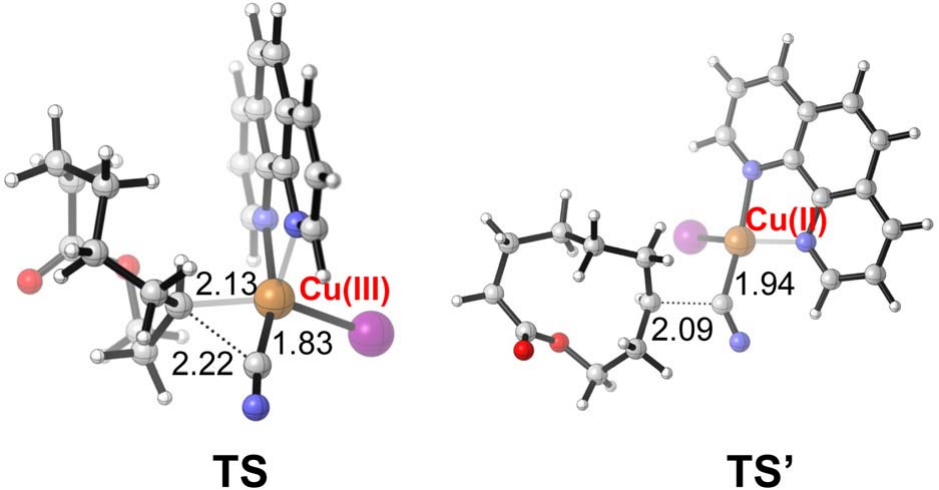
The transition state structures for the formation of a cyano-lactone product involving the Cu(iii) complex (left) or Cu(ii) complex (right). Bond distance is labeled in Å.

To shed light on the reaction mechanism, the radical inhibiting and trapping experiments were first performed (Scheme 5(a)). The addition of 2.0 equiv. of TEMPO to the reaction of 1a and TMSCN almost completely inhibited the formation of 2a, along with a TEMPO-adduct detected by HMRS. The yield of 2a was also decreased to 53% when radical inhibitor BHT (2.0 equiv.) was added. Moreover, replacing TMSCN with 1,2-diphenylethylene as the radial acceptor could afford the alkenylated product 11 in 10% yield under standard conditions. All these results suggest a radical pathway for this ring expansion/cross-coupling cascade. Then, the effect of the ligand was investigated. Without a ligand, just 40% yield of 2a was isolated. When a CuI/1,10-phen complex was used as the catalyst, the product 2a was obtained in 77% yield. These results indicated that the ligand plays an important role in the copper-catalyzed process (Scheme 5(b)). Luckily, some important metallic copper species including cationic [LCu^II^]^+^, [LCu^III^(OH)]H^+^ and [LCu^III^(CN)]H^+^ could be successfully detected by HRMS (Fig. 2, for details, see the ESI†). Density functional theory (DFT) calculations were performed to learn more about the reaction mechanism, and the energy profile for the reaction is shown in Fig. 2. Initially, a single electron transfer (SET) reaction occurs between Cu(i) species SM and hemiketal hydroperoxide 1a, producing the alkoxyl radical IM1 and Cu(ii) hydroxide iodide species IM3 (−9.9 kcal mol^−1^). IM1 undergoes β-scission selectively to afford the carbon-centered radical IM2 with a 1.1 kcal mol^−1^ energy barrier. Radical IM2 then is trapped by copper complex IM4 (−33.2 kcal mol^−1^), which was formed by a kinetically favorable transmetalation between IM3 and TMSCN, giving d^8^ metal centered Cu(iii) cyanide species IM5 (−37.6 kcal mol^−1^). Reductive elimination of IM5 has an energy barrier of only 1.0 kcal mol^−1^. A triplet state for IM5 is −33.8 kcal mol^−1^, and triplet state for TS is −15.8 kcal mol^−1^, indicating that the triplet state surface of IM5 and TS is over the singlet state surface, as shown in Fig. S1† (for details, see the ESI†). This is in accordance with the Cu(i)/Cu(ii)/Cu(iii) mechanism proposed in the previous copper-catalyzed radical reaction study.^12*c*,15^ The C–C bond length of the transition state in the reductive elimination step is 2.22 Å, as shown in Fig. 3 (left), affording the final cyano-lactone product 2a (−84.6 kcal mol^−1^), and regenerating SM to finish the catalytic cycle. Another possible reaction pathway involved Cu(i)/Cu(ii). Cu(ii) complex IM4 accepting one electron from isocyanide, regenerating Cu(i) *via* an outer-sphere pathway,^16^ in which the IM2 radical couples directly with the terminal carbon atom of IM4, with a 2.09 Å length of the C–C bond TS′ as shown in Fig. 3 (right). The transition state energy of TS′ is 22.8 kcal mol^−1^ higher than that of the Cu(iii) complex reductive elimination transition state TS.

## 3 Conclusions

We have developed a highly efficient alkoxyl radical initiated ring expansion/cross-coupling cascade involving copper or iron catalysis. A wide range of medium-sized and macrolactones were constructed in moderate to good yields, accompanied by the introduction of diverse functional groups including CN, N_3_, SCN, and X groups. In view of the mild conditions, wide substrate scope, good functional group tolerance and easy derivatizations of the products, this protocol should be of value to organic chemists. Furthermore, based on the results of experiments and DFT calculations, a Cu(i)/Cu(ii)/Cu(iii) catalytic cycle is proposed for this tandem reaction, wherein the reductive elimination of the cycloalkyl-Cu^III^ species is a more favorable reaction pathway for the cross-coupling step. This work would further expand the application potential of radical C–C bond cleavage in the functionalized medium-sized and macrocyclic compound synthesis.

## Data availability

All experimental and characterization data including NMR spectra and DFT calculations are available in the ESI.† Crystallographic data for compound 2a have been deposited in the Cambridge Crystallographic Data Centre under accession number CCDC 2193944.

## Author contributions

S. Liu performed all the experiments and prepared the manuscript and ESI.† L. Zhang and H.-J. Miao performed the preparation of raw materials. P. Ma, S. Shen and K. N. Houk conducted the DFT calculations. L. Liu, X.-H. Duan and L.-N. Guo directed this project and revised the manuscript. All authors have given approval to the final version of the manuscript.

## Conflicts of interest

There are no conflicts to declare.

## Supplementary Material

SC-014-D2SC06157K-s001
