# Supplementary material for: A cheap metal catalyzed ring expansion/cross-coupling cascade: a new route to functionalized medium-sized and macrolactones
Source: Chem Sci. 2023 Apr 17;14(19):5220–5. doi: 10.1039/d2sc06157k (PMC10189895; doi:10.1039/d2sc06157k)
Supplement: SC-014-D2SC06157K-s001 [file SC-014-D2SC06157K-s001.pdf]

Electronic Supplementary Material (ESI) for Chemical Science.
This journal is © The Royal Society of Chemistry 2023

Supporting Information

Cheap metal catalyzed ring expansion/cross-coupling cascade: A new route to functionalized medium-sized and macrolactones

Shuai Liu,‡[a] Pengchen Ma,‡[ab] Lu Zhang,[a] Shenyu Shen,[a] Hong-Jie Miao,[a] Le Liu,[a] K. N. Houk,*[b] Xin-Hua Duan,*[a] and Li-Na Guo*[a]

[a] Department of Chemistry, School of Chemistry, Xi'an Key Laboratory of Sustainable Energy Material Chemistry and Engineering Research Center of Energy Storage Materials and Devices, Ministry of Education, Xi'an Jiaotong University, Xi'an 710049, China.

[b] Department of Chemistry and Biochemistry, University of California, Los Angeles, California 90095-1569, U.S.A.

E-mail: houk@chem.ucla.edu

E-mail: guoln81@xjtu.edu.cn; duanxh@xjtu.edu.cn

‡ These authors contributed equally to this work.

Table of Contents

1. General Information S3

2. Starting Materials S4

2.1 General Procedure for the Synthesis of Hemiketal Hydroperoxides S4

2.2 Synthesis of the Hemiketal Hydroperoxide **1i** S5

2.3 Notes for Starting Materials S5

3. Optimization of Reaction Conditions S8

3.1 General Procedure for Ring Expansion/Functionalization of Hydroperoxide **1a** S8

3.2 Optimization of Reaction **1a** with TMSCN S9

3.3 Optimization of Reaction **1a** with $TMSN_3$ S11

3.4 Optimization of Reaction **1a** with NH_4SCN S13

3.5 Optimization of Reaction **1a** with HCl (aq) S15

4. Representative Procedure for Schemes **2** and **3** S17

4.1 Representative Procedure for the Reaction of **1** with TMSCN S17

4.2 Representative Procedure for the Reaction of **1** with $TMSN_3$ S17

4.3 Representative Procedure for the Reaction of **1** with NH_4SCN S17

4.4 Representative Procedure for the Reaction of **1** with HX (aq) S17

4.5 Representative Telescoped Procedure for Ring Expansion Coupling/Functionalization of α-Hydroxyalkylketones with Nucleophiles S18

5. Procedures for Diverse Derivatizations of **2a**, **3a** and **4a** S19

6. Scale-up Synthesis S21

7. Investigation of the Reaction Mechanism S21

7.1 Radical Trapping Experiments S21

7.2 Radical Inhibiting Experiment S22

7.3 Ligand Effect Experiments S23

8. Mass Spectrometry Experiments S24

9. Computational Details S28

10. Characterization of Hemiketal Hydroperoxides **1** S38

11. Characterization of Functionalized Lactones **2**-**5** S46

12. Characterization of Products **6-11** S75

13. References S78

14. ^{1}H NMR and ^{13}C NMR Spectra of Hemiketal Hydroperoxides **1** S79

15. ^{1}H NMR and ^{13}C NMR Spectra of Products **2-5** S104

16. ^{1}H NMR and ^{13}C NMR Spectra of Products **6-11** S182

1. General Information

Unless otherwise noted, all reactions were carried out under nitrogen atmosphere, reagents and solvents were obtained from commercial suppliers and were used without further purification. The concentration of HX (aq) are HCl (36%), HBr (40%) and HI (55%-58%). Analytical TLC: aluminum backed plates pre-coated (0.25 mm) with Merck Silica Gel 60F-254. Column chromatography purifications were carried out using 200-300 mesh silica gel. Melting points were measured using open glass capillaries in a SGW® X-4A apparatus. ^{1}H and ^{13}C NMR spectra were recorded on a JNM-ECZ400S/L1 400 MHz spectrometer at ambient temperature. Coupling constants are reported in Hz with multiplicities denoted as s (singlet), d (doublet), t (triplet), q (quartet), m (multiplet) and br (broad). Infrared spectra were recorded on a Bruker V 70 and only major peaks were reported in cm^{-1}. HRMS were obtained on a WATERS I-Class VION IMS Q-Tof with an ESI source. Compounds were visualized by exposure to UV-light or by dipping the plates in $KMnO_4$ stain followed by heating.

2. Starting Materials

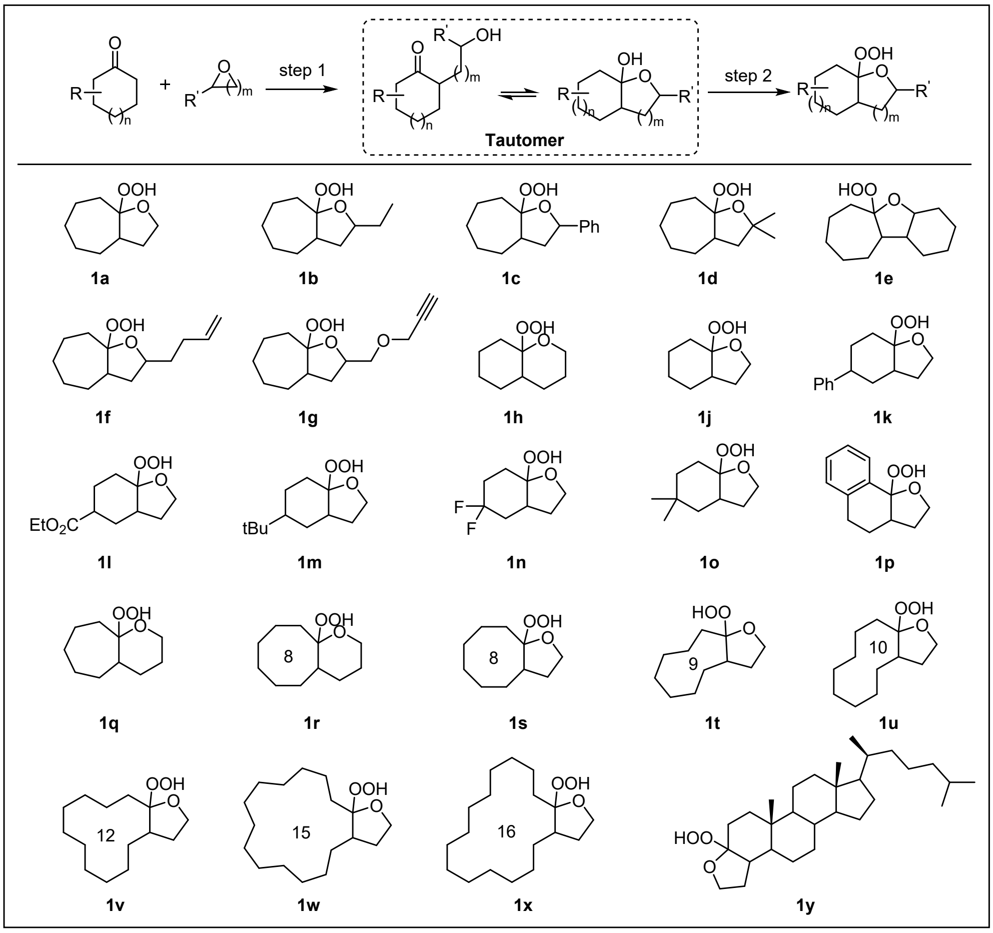

Followed General Procedure for the Synthesis of Hemiketal Hydroperoxides.

2.1 General Procedure for the Synthesis of Hemiketal Hydroperoxides[1,3]

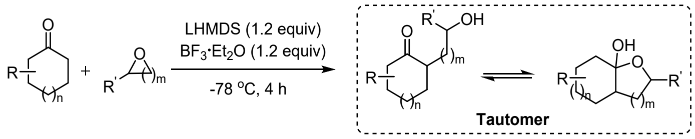

To a stirred solution of LHMDS (1.0 M in THF, 1.2 equiv.) in THF at -78 °C was added cyclic ketone (10 mmol, 1.0 equiv.) over 5 min. After 1 h, epoxide (3.0 M in THF, 2.0 equiv.) was added to the reaction solution. After 1 h, the $BF_3 \cdot Et_2O$ (1.2 equiv.) was added very slowly. The reaction mixture was stirred for 2 h at -78 °C and the reaction was quenched by the addition of saturated aqueous NH_4Cl solution at -78 °C. Layers were separated and the aqueous layer was extracted with EtOAc. The combined organic layer were washed with brine, dried over Na_2SO_4 and concentrated in vacuo. The residue was purified by column chromatography on silica gel (petroleum ether/ethyl acetate = 4:1) to obtain the tautomer of α-hydroxyalkylketones and lactols.

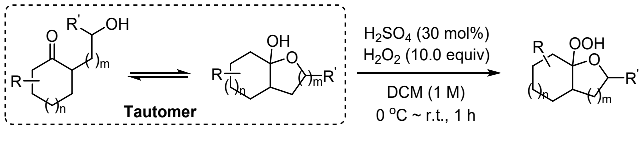

Based on the amount of the tautomer of α-hydroxyalkylketones and lactols: To a reaction flask was added a solution of H_2O_2 (30% wt in H_2O, 10.0 equiv.), and conc. H_2SO_4 (30 mol%). Then a solution of the tautomer of α-hydroxyalkylketones and lactols in DCM (1.0 M) at 0 °C was added. The reaction mixture was stirred for 1 h at room temperature. The aqueous layer was extracted with DCM, the combined organic layer was washed with brine, dried over Na_2SO_4 and concentrated in vacuo. The residue was purified by column chromatography on silica gel (petroleum ether/ethyl acetate = 4:1~10:1) to obtain the hemiketal hydroperoxides.

2.2 Synthesis of the Hemiketal Hydroperoxide 1i[2,3]

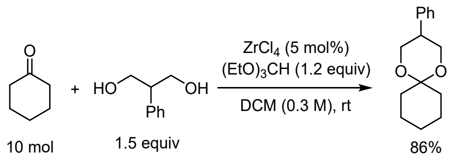

To a solution of cyclic ketones (10 mmol) and 2-phenyl-1,3-propanediol (1.5 equiv.), was added $(EtO)_3CH$ (1.0 M) in dry DCM (0.3 M) and $ZrCl_4$ (5 mol%). Then, the resulting solution was stirred at room temperature. After completion of the reaction, it was quenched with a cold aqueous solution of NaOH (10%) and the organic layer was separated and the aqueous layer was extracted with DCM. The combined organic layer was washed with brine, dried over anhydrous Na_2SO_4 and concentrated in vacuo. The residue was purified by column chromatography on silica gel (petroleum ether/ethyl acetate = 10:1) to obtain the 3-phenyl-1,5-dioxaspiro[5.5]undecane (2.0 g, 86%).

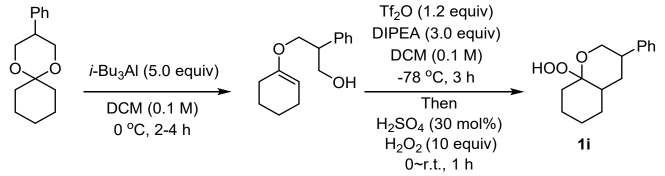

To a cold (0 °C) solution of 3-Phenyl-1,5-dioxaspiro[5.5]undecane (8.6 mmol) in DCM (0.1 M) was added *i*-Bu_3Al (5.0 equiv., 1.0 M in dry toluene). The reaction mixture was stirred at 0 °C for 2-4 h, then, poured into cold 1 N NaOH (aq). The aqueous layer was extracted with DCM, the combined organic layer was washed with brine, dried over Na_2SO_4 and concentrated in vacuo. The residue was purified by column chromatography on silica gel (petroleum ether/ethyl acetate = 10:1~4:1) to obtain the hydroxy vinyl ether (1.5 g, 76%)

The hydroxy vinyl ether (6.5 mmol) was diluted in DCM (0.1 M) and *N*,*N*-diisopropylethylamine (1.0 M). After cooling to -78 °C, trifluoromethanesulfonic anhydride (1.2 equiv.) was slowly added dropwise to this solution. The reaction mixture was stirred at the same temperature for 3 h. After the reaction completed, the reaction mixture was diluted with DCM and brine, the combined organic layer was washed with brine, dried over Na_2SO_4 and concentrated in vacuo. Then the residue was diluted with DCM in 0 °C, and was added a solution of H_2O_2 (30% wt in H_2O, 10.0 equiv.), and conc. H_2SO_4 (30 mol%). The reaction mixture was stirred vigorously for 1 h at room temperature. The aqueous layer was extracted with DCM, the combined organic layer was washed with brine, dried over Na_2SO_4 and concentrated in vacuo. The residue was purified by column chromatography on silica gel (petroleum ether/ethyl acetate = 5:1) to obtain the hemiketal hydroperoxides **1i** (0.5 g, 31%); (20%, three steps)

2.3 Notes for Starting Materials

1. These α-hydroxyalkyl ketones readily tautomerize to the lactols in solution, and the ratio of the two tautomers could be affected easily under acid or basic conditions, concentration, solvent, temperature, and others. This property was also described in these articles: *Tetrahedron*, 1969, **25**, 3157; *Tetrahedron*, 1987, **43**, 3371; *Angew. Chem., Int. Ed.*, 2021, **60**, 5370.

For an example NMR data of automer of α-hydroxyalkylketone and lactol. The ^{1}H NMR spectra showed that the ratio were 2.1:1.9, by comparing the signal areas of protons connected hydroxyl group and protons ether of lactol. Both the ketone carbonyl carbon (216.0, 215.8 ppm) and the tetrasubstituted carbon of lactol (111.99, 111.95 ppm) could be observed in the ^{13}C NMR spectrum.

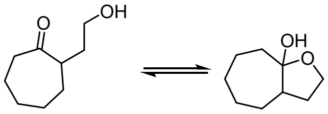

^{1}H NMR (400 MHz, $CDCl_3$) δ 3.79 – 3.51 (m, 2H), 3.43 – 3.15 (m, 1.85H), 2.70 – 2.54 (m, 1H), 2.47 – 2.35 (m, 2H), 2.13 – 1.83 (m, 4H), 1.82 – 1.65 (m, 6H), 1.60 – 1.46 (m, 4H), 1.45 – 1.11 (m, 10H). ^{13}C NMR (100 MHz, $CDCl_3$) δ 216.0, 215.8, 112.0, 112.0, 66.1, 57.6, 57.2, 51.0, 48.8, 48.7, 42.9, 34.4, 32.7, 32.5, 32.4, 32.0, 32.0, 31.5, 31.1, 29.4, 29.2, 28.9, 28.7, 24.3, 24.2, 22.8.

^{1}H NMR (400 MHz, $CDCl_3$) and ^{13}C NMR (100 MHz, $CDCl_3$) spectra of tautomer of α-hydroxyalkylketones and lactols.

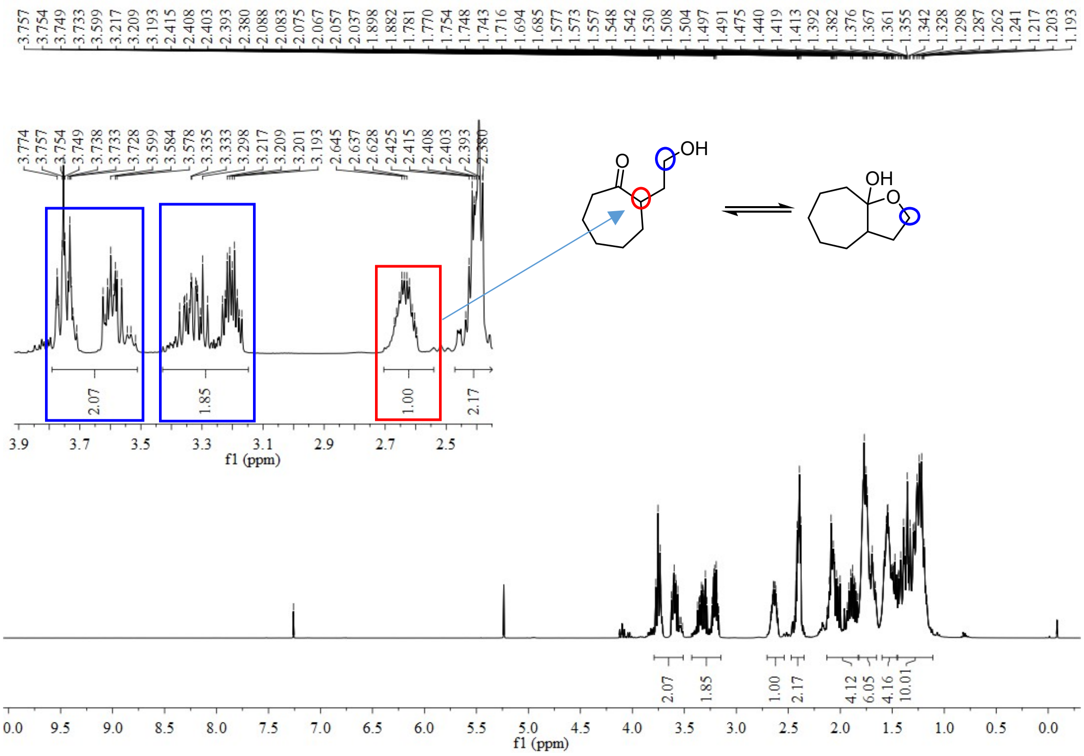

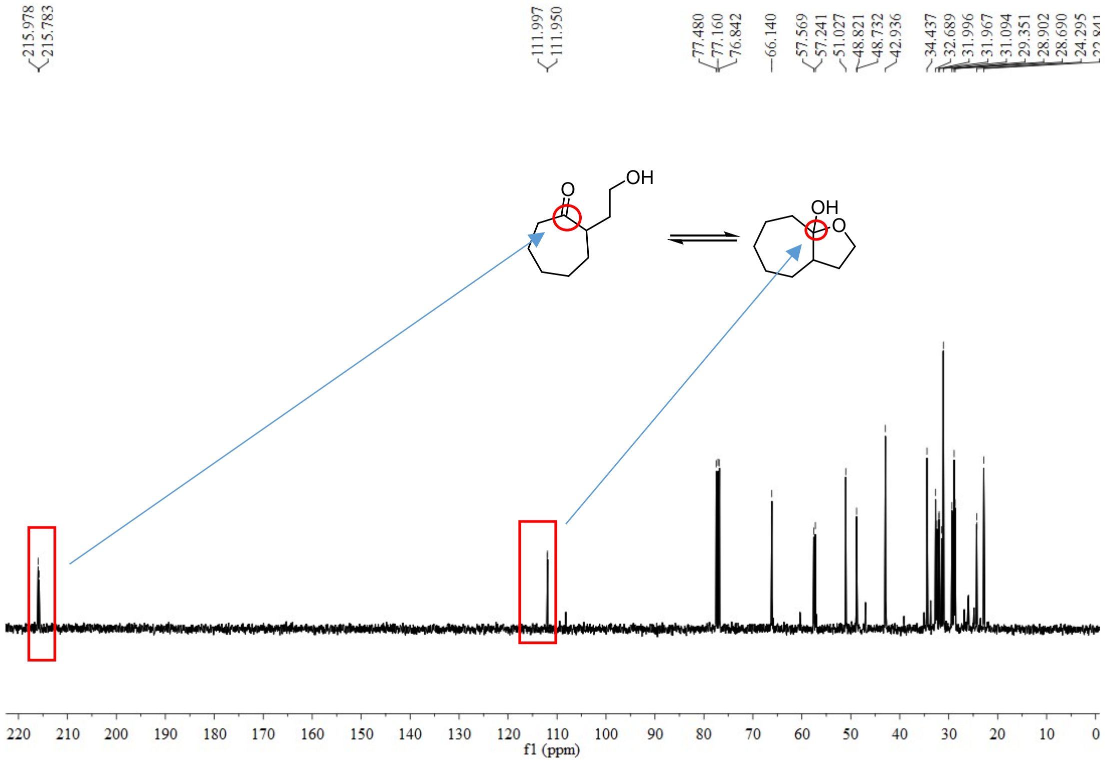

Consider the hemiketal peroxides **1** derived from the mixture of *α*-hydroxyalkyl ketones and the lactols are pure and stable. Thus, the hemiketal peroxides **1** characterization and ^{1}H NMR, ^{13}C NMR spectral data and IR, HRMS data for these compounds were given.

2. All hemiketal hydroperoxides were heated and concentrated by vacuo at below 30 °C and were stored under -20 °C. We have never experienced a safety problem with these materials.

3. Hydroperoxides are bicyclic fused compounds with an inseparable mixture of *cis-trans* isomers, we have tried our utmost to get cleaner ^{1}H NMR and ^{13}C NMR spectra of hydroperoxides.

3. Optimization of Reaction Conditions

3.1 General Procedure for Ring Expansion/Functionalization of Hydroperoxide 1a

A 10 mL oven-dried Schlenk-tube equipped with a magnetic stirrer was added catalyst and ligand. Then, the tube was evacuated and backfilled with nitrogen for three times. Subsequently, a solution of hemiketal hydroperoxide **1a** (0.2 mmol, 1.0 equiv.) and nucleophiles (0.3 mmol, 1.5 equiv.) in solvent (2.0 mL) was added by syringe under nitrogen atmosphere. The tube was then sealed and the mixture was stirred at 25 ^{o}C for specified time. After the reaction completed, the reaction mixture was concentrated in vacuo and then purified by column chromatography on silica gel to give the target products.

3.2 Optimization of Reaction 1a with TMSCN[a]

Screening of Solvents

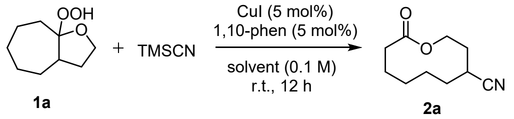

Entry	Solvent	Yield (%)
1	NMP	78
2	DMF	78
3	MeCN	87
**4**	**MeOH**	**98(95)**[b]
5	THF	88
6	DCE	85
7	toluene	14
8	1,4-dioxane	25
9	MTBE	trace

[a]Reaction conditions: **1a** (0.2 mmol, 1.0 equiv.), TMSCN (0.3 mmol, 1.5 equiv.), CuI (0.01 mmol, 5 mol%), 1,10-phen (0.01 mmol, 5 mol%), and solvent (2.0 mL) at 25 °C for 12 h under N_2. Yields were determined by GC-FID analysis of the crude reaction mixture with dodecane as the internal standard. [b]Isolated yield was given in parenthesis.

Screening of Catalysts

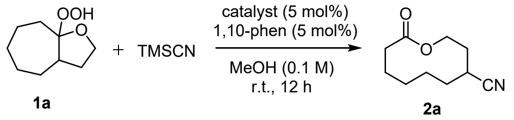

[a]Reaction conditions: **1a** (0.2 mmol, 1.0 equiv.), TMSCN (0.3 mmol, 1.5 equiv.), catalyst (0.01 mmol, 5 mol%),

Entry	Catalyst (5 mol%)	Yield (%)
**1**	**CuI**	**98**
2	CuOTf	40
3	$Cu(OAc)_2$	12
4	$Fe(OTf)_2$	N.R.

1,10- phen (0.01 mmol, 5 mol%), and MeOH (2.0 mL) at 25 °C for 12 h under N_2. Yields were determined by GC-FID analysis of the crude reaction mixture with dodecane as the internal standard.

Screening of Ligands

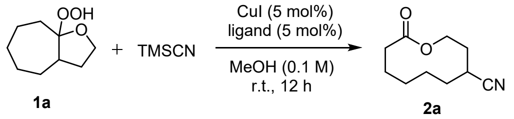

[a]Reaction conditions: **1a** (0.2 mmol, 1.0 equiv.), TMSCN (0.3 mmol, 1.5 equiv.), CuI (0.01 mmol, 5 mol%), ligand

Entry	Ligand (5 mol%)	Yield (%)
**1**	**1,10-phen**	**98**
2	2,2'-bpy	70
3	-	40

(0.01 mmol, 5 mol%), and MeOH (2.0 mL) at 25 °C for 12 h under N_2. Yields were determined by GC-FID analysis of the crude reaction mixture with dodecane as the internal standard.

Screening of Time

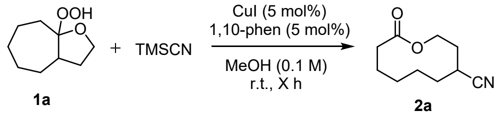

Entry	Time (X h)	Yield (%)
1	12	98
2	6	95
3	1	96
4	0.5	97
5	15 min	97(94)[b]
**6**	**10 min**	**98(95)[b]**
7	5 min	92(88)[b]

[a]Reaction conditions: **1a** (0.2 mmol, 1.0 equiv.), TMSCN (0.3 mmol, 1.5 equiv.), CuI (0.01 mmol, 5 mol%), 1,10-phen (0.01 mmol, 5 mol%), and MeOH (2.0 mL) at 25 °C for X h under N_2. Yields were determined by GC-FID analysis of the crude reaction mixture with dodecane as the internal standard. [b]Isolated yields were given in parenthesis.

Screening of Ligands

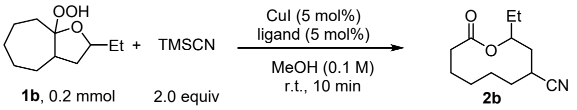

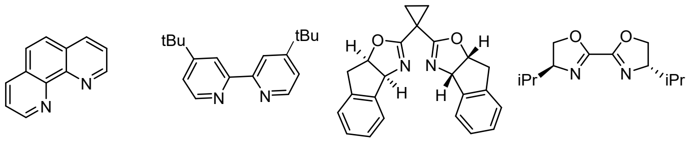

98%, d.r. = 3:1 | 70%, d.r. = 3:1 | 65%, d.r. = 3:1 | 61%, d.r. = 3:1

[a]Reaction conditions: **1b** (0.2 mmol, 1.0 equiv.), TMSCN (0.3 mmol, 1.5 equiv.), CuI (0.01 mmol, 5 mol%), ligand (0.01 mmol, 5 mol%), and MeOH (2.0 mL) at 25 °C for 10 min under N_2. Isolated yields

Screening of Chiral Ligands

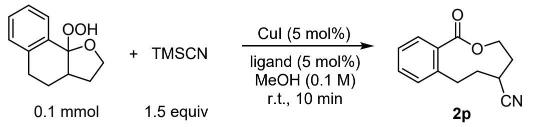

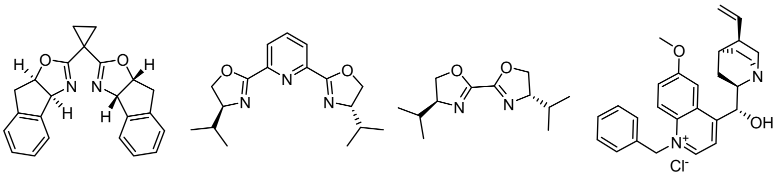

37%, (0% ee) | 29%, (0% ee) | 39%, (0% ee) | 36%, (0% ee)

[a]Reaction conditions: **1p** (0.1 mmol, 1.0 equiv.), TMSCN (0.15 mmol, 1.5 equiv.), CuI (0.005 mmol, 5 mol%), ligand (0.005 mmol, 5 mol%), and MeOH (1.0 mL) at 25 °C for 10 min under N_2. Isolated yields. The ee values were determined by HPLC analysis.

3.3 Optimization of Reaction 1a with $TMSN_3$[a]

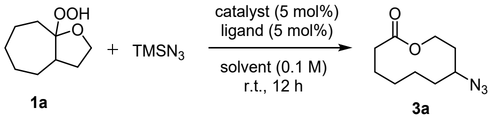

Entry	Catalyst (5 mol%)	Solvent	Ligand	Yield (%)
1	CuI	MeOH	1,10-phen	10
2	CuI	NMP	1,10-phen	18
3	CuI	DMF	1,10-phen	14
4	CuI	THF	1,10-phen	27
**5**	**$Fe(OTf)_2$**	**MeOH**	**1,10-phen**	**74**
6	$Fe(OTf)_2$	MeOH	-	70

[a]Reaction conditions: **1a** (0.2 mmol, 1.0 equiv.), $TMSN_3$ (0.3 mmol, 1.5 equiv.), catalyst (0.01 mmol, 5 mol%), ligand (0.01 mmol, 5 mol%), and solvent (2.0 mL) at 25 °C for 12 h under N_2. Isolated yields.

Screening of Solvents

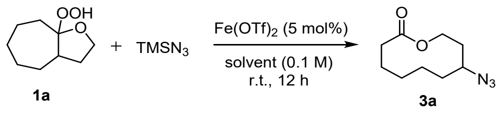

Entry	Solvent	Yield (%)
1	DMF	14
2	MeCN	70
3	DCE	12
4	THF	67
5	toluene	trace
6	acetone	trace
7	MTBE	73
8	MeOH	70
9	*i*-PrOH	75
10	DME	80(70)[b]
**11**	**EtOAc**	**89(84)[b]**

[a]Reaction conditions: **1a** (0.2 mmol, 1.0 equiv.), $TMSN_3$ (0.3 mmol, 1.5 equiv.), $Fe(OTf)_2$ (0.01 mmol, 5 mol%), and solvent (2.0 mL) at 25 °C for 12 h under N_2. Yields were determined by GC-FID analysis of the crude reaction mixture with dodecane as the internal standard. [b]Isolated yields were given in parenthesis.

Screening of Catalysts

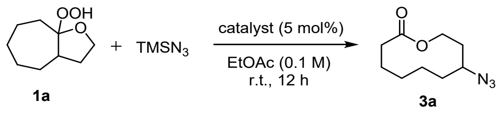

Entry	Catalyst (5 mol%)	Yield (%)
**1**	**$Fe(OTf)_2$**	**89**
2	$Fe(OTf)_3$	73
3	$Fe(OTs)_3$	50
4	$FeBr_2$	49
5	$Fe(OAc)_2$	41
6	$FeCl_2$	42
7	CuI	trace
8	CuOTf	trace

[a]Reaction conditions: **1a** (0.2 mmol, 1.0 equiv.), $TMSN_3$ (0.3 mmol, 1.5 equiv.), catalyst (0.01 mmol, 5 mol%), and EtOAc (2.0 mL) at 25 °C for 12 h under N_2. Yields were determined by GC-FID analysis of the crude reaction mixture with dodecane as the internal standard.

Screening of Time

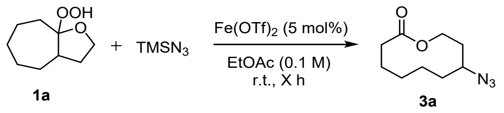

Entry	Time (X h)	Yield (%)
1	1	88
**6**	**10 min**	**87(82)**[b]
7	5 min	82(78)[b]

[a]Reaction conditions: **1a** (0.2 mmol, 1.0 equiv.), $TMSN_3$ (0.3 mmol, 1.5 equiv.), $Fe(OTf)_2$ (0.01 mmol, 5 mol%), and EtOAc (2.0 mL) at 25 °C for X h under N_2. Yields were determined by GC-FID analysis of the crude reaction mixture with dodecane as the internal standard. [b]Isolated yields were given in parenthesis.

3.4 Optimization of Reaction 1a with NH_4SCN^a

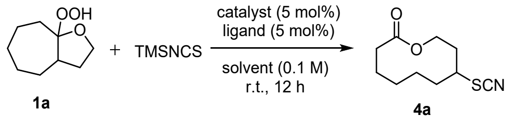

Entry	Catalyst (5 mol%)	Solvent	Ligand	Yield (%)
1	CuI	MeOH	1,10-phen	trace
2	CuI	NMP	1,10-phen	trace

[a]Reaction conditions: **1a** (0.2 mmol, 1.0 equiv.), TMSNCS (0.3 mmol, 1.5 equiv.), CuI (0.01 mmol, 5 mol%), 1,10-phen (0.01 mmol, 5 mol%), and solvent (2.0 mL) at 25 °C for 12 h under N_2. Isolated yields.

Screening of "SCN" Source

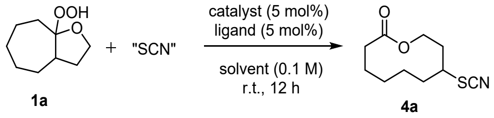

Entry	Catalyst	"SCN" Source	Solvent	Ligand	Yield (%)
1	CuI	TMSNCS	NMP	1,10-phen	trace
2	CuI	NH_4SCN	NMP	1.10-phen	67
3	CuI	NH_4SCN	NMP	-	60

[a]Reaction conditions: **1a** (0.2 mmol, 1.0 equiv.), thiocyanation source (0.3 mmol, 1.5 equiv.), CuI (0.01 mmol, 5 mol%), ligand (0.01 mmol, 5 mol%), and NMP (2.0 mL) at 25 °C for 12 h under N_2. Isolated yields.

Screening of Solvents

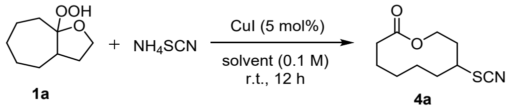

Entry	Solvent	Yield (%)
1	DMF	66
2	NMP	60
3	DMA	39
4	DMSO	36
5	DCE	52
6	THF	trace
7	MeOH	trace
**8**	**MeCN**	**82**

[a]Reaction conditions: **1a** (0.2 mmol, 1.0 equiv.), NH_4SCN (0.3 mmol, 1.5 equiv.), CuI (0.01 mmol, 5 mol%), and solvent (2.0 mL) at 25 °C for 12 h under N_2. Yields were determined by GC-FID analysis of the crude reaction mixture with dodecane as the internal standard.

Screening of Catalysts

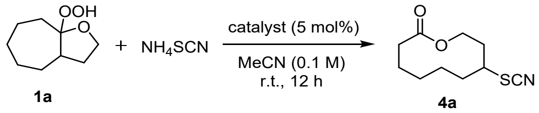

Entry	Catalyst (5 mol%)	Yield (%)
1	CuI	82
2	CuOTf	82
3	$Cu(OAc)_2$	92
4	$Cu(CH_3CN)_4PF_6$	96
**5**	**CuCl**	**99(94)**[b]
6	CuSCN	87

[a]Reaction conditions: **1a** (0.2 mmol, 1.0 equiv.), NH_4SCN (0.3 mmol, 1.5 equiv.), catalyst (0.01 mmol, 5 mol%), and MeCN (2.0 mL) at 25 °C for 12 h under N_2. Yields were determined by GC-FID analysis of the crude reaction mixture with dodecane as the internal standard. [b]Isolated yield was given in parenthesis.

Screening of Time

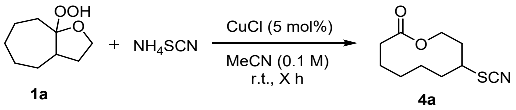

Entry	Time (X h)	Yield (%)
1	3	97
**2**	**1**	**98(93)** [b]
3	10 min	82(73)[b]

[a]Reaction conditions: **1a** (0.2 mmol, 1.0 equiv.), NH_4SCN (0.3 mmol, 1.5 equiv.), CuCl (0.01 mmol, 5 mol%), and MeCN (2.0 mL) at 25 °C for X h under N_2. Yields were determined by GC-FID analysis of the crude reaction mixture with dodecane as the internal standard. [b]Isolated yields were given in parenthesis.

3.5 Optimization of Reaction 1a with HCl (aq)[a]

Screening of Solvents

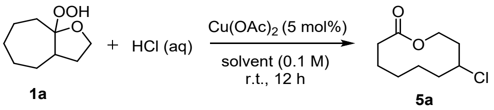

Entry	Solvent	Yield (%)
1	DMF	68
2	NMP	76
3	MeCN	46
4	THF	9
5	toluene	trace

[a]Reaction conditions: **1a** (0.2 mmol, 1.0 equiv.), HCl (aq) (0.4 mmol, 2.0 equiv.), $Cu(OAc)_2$ (0.01 mmol, 5 mol%), and solvent (2.0 mL) at 25 °C for 12 h under N_2. Yields were determined by GC-FID analysis of the crude reaction mixture with dodecane as the internal standard.

Screening of Catalysts

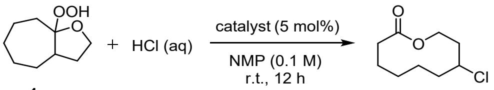

Entry	Catalyst (5 mol%)	Yield (%)
1	CuI	90
2	CuOTf	86
3	$Cu(OAc)_2$	76
4	$Cu(CH_3CN)_4PF_6$	90
**5**	**CuCl**	**92(88)**[b]
6	CuSCN	88
7	CuTC	81
8	$CuCl_2$	87

[a]Reaction conditions: **1a** (0.2 mmol, 1.0 equiv.), HCl (aq) (0.4 mmol, 2.0 equiv.), catalyst (0.01 mmol, 5 mol%), and NMP (2.0 mL) at 25 °C for 12 h under N_2. Yields were determined by GC-FID analysis of the crude reaction mixture with dodecane as the internal standard. [b]Isolated yield was given in parenthesis.

Screening of Time

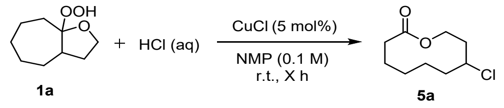

Entry	Time (X h)	Yield (%)
1	1	90(87)[b]
**2**	**10 min**	**92(88)[b]**
3	5 min	88(83)[b]

[a]Reaction conditions: **1a** (0.2 mmol, 1.0 equiv.), HCl (aq) (0.4 mmol, 2.0 equiv.), CuCl (0.01 mmol, 5 mol%), and NMP (2.0 mL) at 25 ^{o}C for X h under N_2. Yields were determined by GC-FID analysis of the crude reaction mixture with dodecane as the internal standard. [b]Isolated yields were given in parenthesis.

4. Representative Procedure for Schemes 2 and 3

4.1 Representative Procedure for the Reaction of 1 with TMSCN

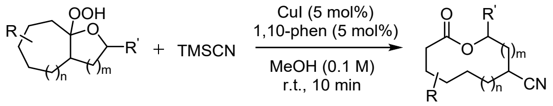

1 2

To a 10 mL oven-dried Schlenk-tube equipped with a magnetic stirrer was added CuI (0.01 mmol, 5 mol%), 1,10-phen (0.01 mmol, 5 mol%). Then, the tube was evacuated and backfilled with nitrogen for three times. Subsequently, a solution of hemiketal hydroperoxides **1** (0.2 mmol, 1.0 equiv.), and TMSCN (0.3 mmol, 1.5 equiv.) in MeOH (2.0 mL) was added by syringe under nitrogen atmosphere. The tube was then sealed and mixture was stirred at 25 ^{o}C for 10 min. After the reaction completed, the reaction mixture was concentrated and then purified by column chromatography on silica gel (petroleum ether/ethyl acetate = 5:1) to give the target products **2**.

4.2 Representative Procedure for the Reaction of 1 with $TMSN_3$

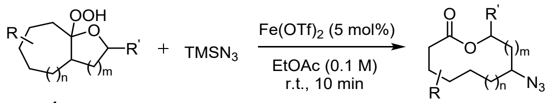

1 3

To a 10 mL oven-dried Schlenk-tube equipped with a magnetic stirrer was added $Fe(OTf)_2$ (0.01 mmol, 5 mol%). Then, the tube was evacuated and backfilled with nitrogen for three times. Subsequently, a solution of hemiketal hydroperoxides **1** (0.2 mmol, 1.0 equiv.), and $TMSN_3$ (0.3 mmol, 1.5 equiv.) in EtOAc (2.0 mL) was added by syringe under nitrogen atmosphere. The tube was then sealed and the mixture was stirred at 25 ^{o}C for 10 min. After the reaction completed, the reaction mixture was concentrated and then purified by column chromatography on silica gel (petroleum ether/ethyl acetate = 40:1) to give the target products **3**.

4.3 Representative Procedure for the Reaction of 1 with NH_4SCN

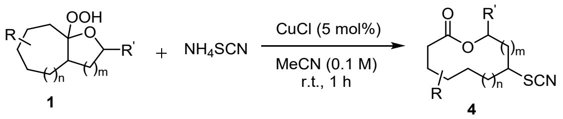

1 4

To a 10 mL oven-dried Schlenk-tube equipped with a magnetic stirrer was added NH_4SCN (0.3 mmol, 1.5 equiv.), CuCl (0.01 mmol, 5 mol%). Then, the tube was evacuated and backfilled with nitrogen for three times. Subsequently, a solution of hemiketal hydroperoxides **1** (0.2 mmol, 1.0 equiv.) in MeCN (2.0 mL) was added by syringe under nitrogen atmosphere. The tube was then sealed and the mixture was stirred at 25 ^{o}C for 1 h. After the reaction completed, the reaction mixture was concentrated and then purified by column chromatography on silica gel (petroleum ether/ethyl acetate = 5:1) to give the target products **4**.

4.4 Representative Procedure for the Reaction of 1 with HX (aq)

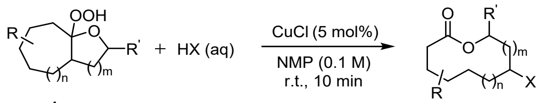

1

5, X = Cl, Br, I

To a 10 mL oven-dried Schlenk-tube equipped with a magnetic stirrer was added CuCl (0.01 mmol, 5 mol%). Then, the tube was evacuated and backfilled with nitrogen for three times. Subsequently, a solution of hemiketal hydroperoxides **1** (0.2 mmol, 1.0 equiv.), and HCl (aq) or HBr (aq) or HI (aq) (0.4 mmol, 2.0 equiv.) in NMP (2.0 mL) was added by syringe under nitrogen atmosphere. The tube was then sealed and the mixture was stirred at 25 °C for 10 min. After the reaction completed, the reaction mixture was diluted with EtOAc (5.0 mL) and H_2O (5.0 mL). The organic layer was separated and the water layer was extracted with EtOAc (3 × 5 mL). The combined organic layer was washed with saturated brine, dried over Na_2SO_4 and concentrated in vacuo. The residue was purified by column chromatography on silica gel (petroleum ether/ethyl acetate = 40:1) to give the target products **5**.

4.5 Representative Telescoped Procedure for Ring Expansion/Functionalization of α-Hydroxyalkylketones with Nucleophiles

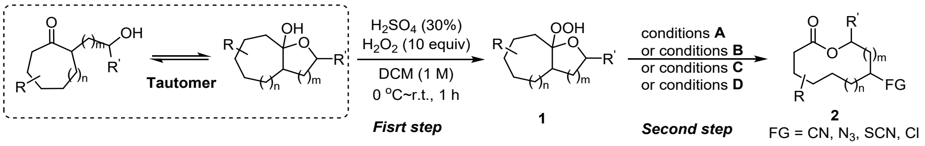

First step:

To a 10 mL reaction tube equipped with a magnetic stirrer was added a solution of H_2O_2 (30% wt in H_2O, 10.0 equiv.), and conc. H_2SO_4 (30 mol%). Then a solution of the tautomer of α-hydroxyalkylketones and lactols (0.2 mmol, 1.0 equiv.) in DCM (1.0 M) at 0 °C was added. Then, the reaction mixture was stirred for 1 h from 0 °C to 25 °C. After the reaction completed, it was diluted with DCM (5.0 mL) and H_2O (5.0 mL). The organic layer was separated and the water layer was extracted with EtOAc (3 × 5 mL). The combined organic layer was washed with saturated brine, dried over Na_2SO_4 and concentrated in vacuo. The crude products of **1** were used in the next step without further purification

Second step:

Followed the above-mentioned representative procedure for ring-expansion/cyanation or azidation or thiocyanation or halogenation of **1**. The total yields based on the mixture of α-hydroxyalkyl ketones and lactols were given in Scheme 2 and 3.

5. Procedures for Diverse Derivatizations of 2a, 3a and 4a[4-7]

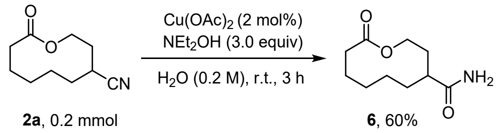

To a 10 mL reaction tube equipped with a magnetic stirrer was added **2a** (0.2 mmol, 1.0 equiv.), $Cu(OAc)_2$ (0.04 momol, 2 mol%), NEt_2OH (0.6 mmol, 3.0 equiv.) in H_2O (1 mL). The reaction mixture was stirred at 25°C for 3 h. After the reaction completed, the reaction mixture was diluted with EtOAc (5.0 mL) and H_2O (5.0 mL). The organic layer was separated and the water layer was extracted with EtOAc (3 × 5 mL). The combined organic layer was washed with saturated brine, dried over Na_2SO_4 and concentrated in vacuo. The residue was purified by column chromatography on silica gel (ethyl acetate) to give the target product **6** in 60% yield.

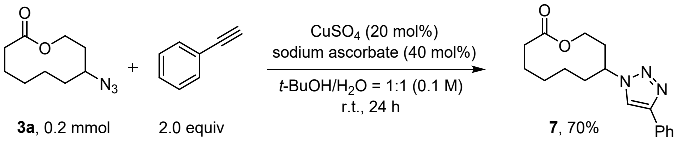

To a 10 mL oven-dried Schlenk-tube equipped with a magnetic stirrer was added **3a** (0.2 mmol, 1.0 equiv.), $CuSO_4$ (0.04 mmol, 20 mol%) and sodium ascorbate (0.08 mmol, 40 mol%). Then, the tube was evacuated and backfilled with nitrogen for three times. Subsequently, a solution of phenylacetylene (0.4 mmol, 2.0 equiv.) in *t*-BuOH/H_2O (1:1, 2.0 mL) was added by syringe under nitrogen atmosphere. The tube was then sealed and the mixture was stirred at 25 °C for 24 h. After the reaction completed, the reaction mixture was diluted with EtOAc (5.0 mL) and H_2O (5.0 mL). The organic layer was separated and the water layer was extracted with EtOAc (3 × 5 mL). The combined organic layer was washed with saturated brine, dried over Na_2SO_4 and concentrated in vacuo. The residue was purified by column chromatography on silica gel (petroleum ether/ethyl acetate = 2:1) to give the target product **7** in 70% yield.

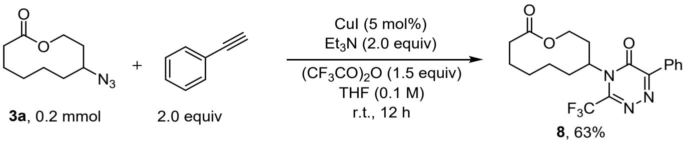

To a 10 mL oven-dried Schlenk-tube equipped with a magnetic stirrer was added **3a** (0.2 mmol, 1.0 equiv.), CuI (0.01 mmol, 5 mol%). Then, the tube was evacuated and backfilled with nitrogen for three times. Subsequently, a solution of phenylacetylene (0.4 mmol, 2.0 equiv.), Et_3N (0.4 mmol, 2.0 equiv.) and $(CF_3CO)_2O$ (0.3 mmol, 1.5 equiv.) in dry THF (2.0 mL) was added by syringe under nitrogen atmosphere. The tube was then sealed and the mixture was stirred at 25 °C for 12 h. After the reaction completed, the reaction mixture was concentrated in vacuo and was further purified by column chromatography on silica gel (petroleum ether/ethyl acetate = 10:1) to give the target product **8** in 63% yield.

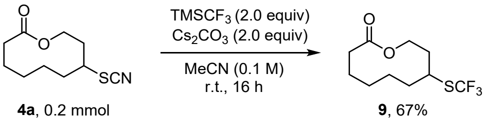

To a 10 mL oven-dried Schlenk-tube equipped with a magnetic stirrer was added **4a** (0.2 mmol, 1.0 equiv.), Cs_2CO_3 (0.4 mmol, 2.0 equiv.). Then, the tube was evacuated and backfilled with nitrogen for

three times. Subsequently, MeCN (2.0 mL) was added by syringe under nitrogen atmosphere. Then, $TMSCF_3$ (0.4 mmol, 2.0 equiv.) was added slowly by microsyringe at 0 °C. The tube was then sealed and the mixture was stirred at 25 °C for 16 h. After the reaction completed, the reaction mixture was concentrated in vacuo and was further purified by column chromatography on silica gel (petroleum ether/ethyl acetate = 40:1) to give the target product **9** in 67% yield.

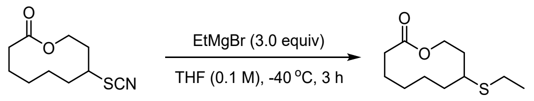

4a, 0.2 mmol   **10**, 70%

To a 10 mL oven-dried Schlenk-tube equipped with a magnetic stirrer was added **4a** (0.2 mmol, 1.0 equiv.), and backfilled with nitrogen for three times. Subsequently, dry THF (2.0 mL) was added by syringe under nitrogen atmosphere. Then, EtMgBr (1M THF) (0.6 mmol, 3.0 equiv.) was added slowly at -40 °C. The tube was then sealed and the mixture was stirred at -40 °C for 3 h. After the reaction completed, the reaction mixture was concentrated in vacuo, and then purified by column chromatography on silica gel (petroleum ether/ethyl acetate = 50:1) to give the target product **10** in 70% yield.

6. Scale-up Synthesis

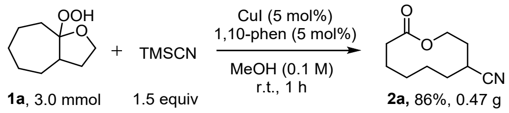

To a 100 mL oven-dried Schlenk-tube equipped with a magnetic stirrer was added CuI (0.15 mmol, 5 mol%), 1,10-phen (0.15 mmol, 5 mol%). Then, the tube was evacuated and backfilled with nitrogen for three times. Subsequently, a solution of hemiketal hydroperoxide **1a** (3 mmol, 1.0 equiv.), and TMSCN (4.5 mmol, 1.5 equiv.) in MeOH (30 mL) was added by syringe under nitrogen atmosphere. The tube was then sealed and the mixture was stirred at 25 ^{o}C for 1 h. After the reaction completed, the reaction mixture was concentrated in vacuo, and then purified by column chromatography on silica gel (petroleum ether/ethyl acetate = 5:1) to give the target products **2a** (470 mg, 86%).

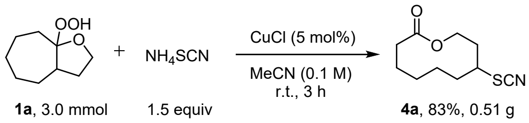

To a 100 mL oven-dried Schlenk-tube equipped with a magnetic stirrer was added CuCl (0.15 mmol, 5 mol%), NH_4SCN (4.5 mmol, 1.5 equiv.). Then, the tube was evacuated and backfilled with nitrogen for three times. Subsequently, a solution of hemiketal hydroperoxide **1a** (3 mmol, 1.0 equiv.) in MeCN (30 mL) was added by syringe under nitrogen atmosphere. The tube was then sealed and the mixture was stirred at 25 ^{o}C for 3 h. After the reaction completed, the reaction mixture was concentrated in vacuo, and then purified by column chromatography on silica gel (petroleum ether/ethyl acetate = 5:1) to give the target products **4a** (513 mg, 83%).

7. Investigation of the Reaction Mechanism

7.1 Radical Trapping Experiments

(1) Using TEMPO as the radical trapping reagent

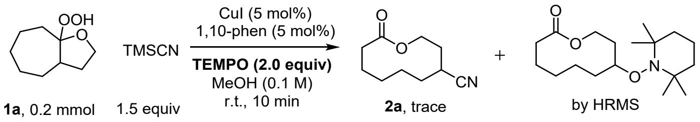

To a 10 mL oven-dried Schlenk-tube equipped with a magnetic stirrer was added CuI (0.01 mmol, 5 mol%), 1,10-phen (0.01 mmol, 5 mol%), and TEMPO (0.4 mmol, 2.0 equiv.). Then, the tube was evacuated and backfilled with nitrogen for three times. Subsequently, a solution of hemiketal hydroperoxide **1a** (0.2 mmol, 1.0 equiv.), and TMSCN (0.3 mmol, 1.5 equiv.) in MeOH (2.0 mL) was added by syringe under nitrogen atmosphere. The tube was then sealed and the mixture was stirred at 25 ^{o}C for 10 min. After the reaction completed, it was found that the reaction was totally inhibited and the TEMPO-adduct could be detected by LC-MS (HRMS (ESI) calcd for $C_{18}H_{34}NO_3$ $[M+H]^+$ 312.2533, found 312.2517. These results indicate that a radical intermediate might be involved in this transformation.

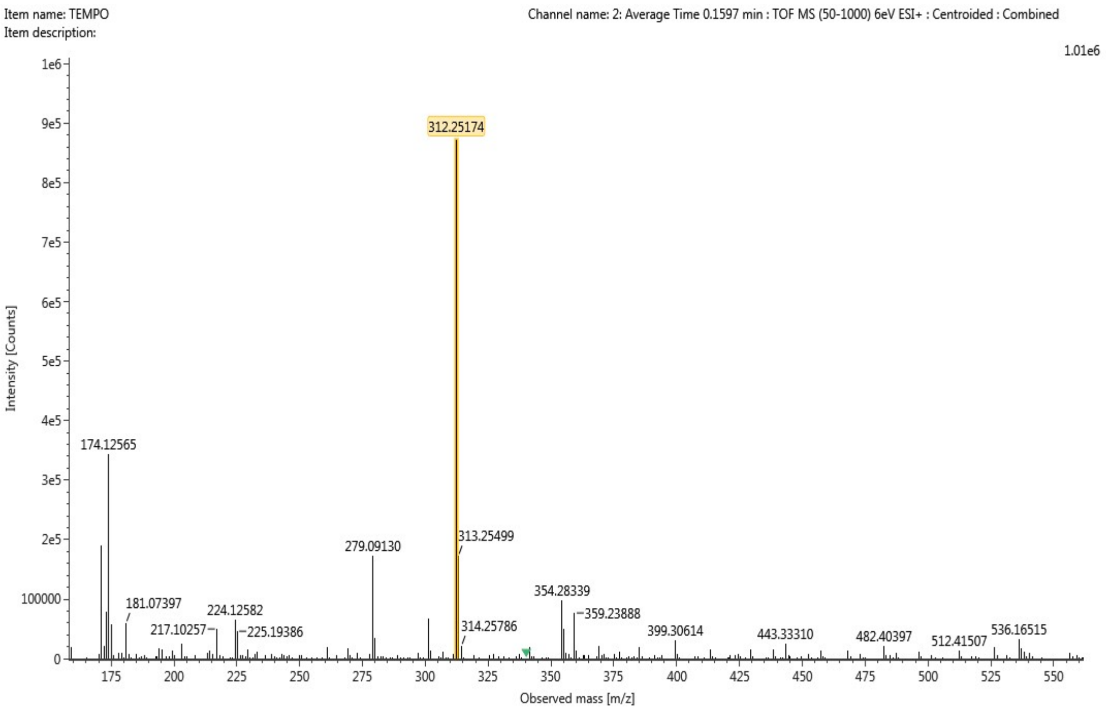

(2) Using 1,1-diphenylethylene as the radical trapping reagent

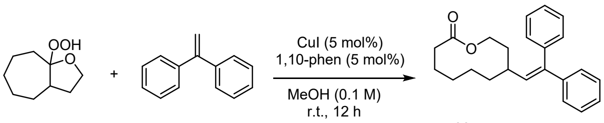

1a, 0.2 mmol 1.5 equiv **11**, 10%

To a 10 mL oven-dried Schlenk-tube equipped with a magnetic stirrer was added CuI (0.01 mmol, 5 mol%), 1,10-phen (0.01 mmol, 5 mol%), and 1,1-diphenylethylene (1.5 equiv.). Then, the tube was evacuated and backfilled with nitrogen for three times. Subsequently, a solution of hemiketal hydroperoxide **1a** (0.2 mmol, 1.0 equiv.) in MeOH (2.0 mL) was added by syringe under nitrogen atmosphere. The tube was then sealed and the mixture was stirred at 25 °C for 12 h. After the reaction completed, the reaction mixture was concentrated in vacuo, and then purified by column chromatography on silica gel (petroleum ether/ethyl acetate = 20:1) to give the alkenylated product **11** in 10% yield. This result proved that the reaction probably proceeded *via* a radical pathway. Notably, when the reaction was conducted under the $Fe(OTf)_2$ catalysis in EtOAc, a 52% yield of **11** could be obtained.

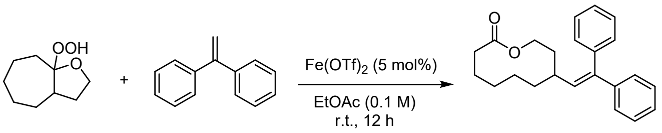

1a, 0.2 mmol 1.5 equiv **11**, 52%

7.2 Radical Inhibiting Experiment

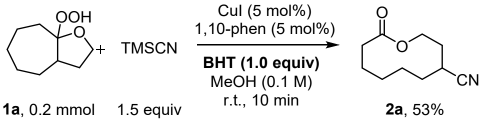

1a, 0.2 mmol 1.5 equiv **2a**, 53%

To a 10 mL oven-dried Schlenk-tube equipped with a magnetic stirrer was added CuI (0.01 mmol, 5 mol%), 1,10-phen (0.01 mmol, 5 mol%), and BHT (0.2 mmol, 1.0 equiv.). Then, the tube was evacuated and backfilled with nitrogen for three times. Subsequently, a solution of hemiketal hydroperoxide **1a** (0.2 mmol, 1.0 equiv.), and TMSCN (0.3 mmol, 1.5 equiv.) in MeOH (2.0 mL) was added by syringe under

nitrogen atmosphere. The tube was then sealed and the mixture was stirred at 25 °C for 10 min. After the reaction completed, the reaction mixture was concentrated in vacuo, and then purified by column chromatography on silica gel. In this case, the yield of **2a** was reduced to 53%. This result indicates that the reaction might proceed via a radical pathway.

7.3 Ligand Effect Experiments

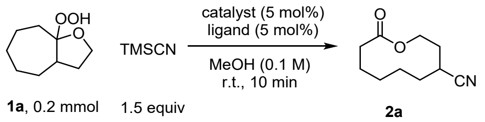

[a]Reaction conditions: **1a** (0.2 mmol, 1.0 equiv.), TMSCN (0.3 mmol, 1.5 equiv.), catalyst (0.01 mmol, 5 mol%),

Entry	Catalyst + Ligand (5 mol%)	Yield (%)
1	CuI+1,10-phen	95
2	CuI+2,2'-bpy	70
3	CuI	40
4	CuI·(1,10-phen) complex	77

ligand (0.01 mmol, 5 mol%), and MeOH (2.0 mL) at 25 °C for 10 min under N_2. Isolated yields.

8. Mass Spectrometry Experiments

The experiments were performed with an I-Class VION IMS Q-Tof instrument with an electrospray ion source, it was purchased from the Waters company. The instrument equipped a quadrupole mass filter after ion source for the selection of target ions. In the collision induced dissociation (CID) process, the mass-selected ions enter an argon filled linear ion trap (argon pressure: 8×10^{-3} mbar) after accelerated by electric field. After collision, the ions enter a reflection time-of-flight (TOF) region for determination. For a given ion of interest, it's type is defined via mass-to-charge ratio and collision induced dissociation. Collision induced dissociation was also employed to analyze structural fragments.

Proceduer 1. CuI (0.01 mmol) and 1,10-phen (0.015 mmol) were dissolved in MeOH (1.0 mL), and the mixture was stirred at room temperature for 30 minutes. After 30 minutes, 10.0 μL reaction mixture was picked up with pipette and dissolved into 1.0 mL MeCN. Next, this dilution was transferred into an injection syringe, and injected into the high-resolution electrospray mass spectrometry (I-Class VION IMS Q-Tof) by injection pump. Finally, the MS data were collected and analyzed.

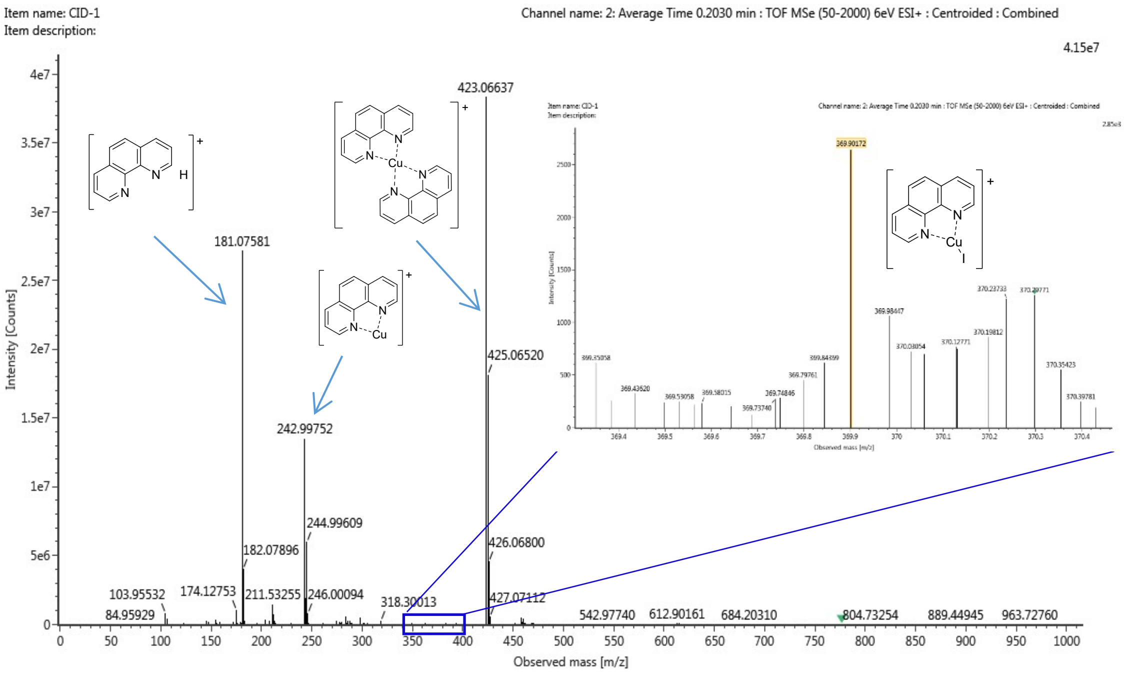

The mass spectra of **procedure 1**

The mass spectra of procedure 1. We successfully detected moderate intensity of the intermediate **[Cu(1,10-phen)I]**$^+$ HRMS (ESI) calcd for $C_{12}H_8CuIN_2$ $[M]^+$ 369.9028, found 369.9017.

Procedure 2: CuI (0.01 mmol) and 1,10-phen (0.015 mmol) were dissolved in MeOH (1.0 mL), and the mixture was stirred at room temperature for 30 minutes. Then hemiketal hydroperoxide **1a** (0.1 mmol) was added to the mixture. After 30 minutes, 10.0 μL reaction mixture was picked up with pipette and dissolved into 1.0 mL MeCN. Next, this dilution was transferred into an injection syringe, and injected into the high-resolution electrospray mass spectrometry (I-Class VION IMS Q-Tof) by injection pump. Finally, the MS data were collected and analyzed.

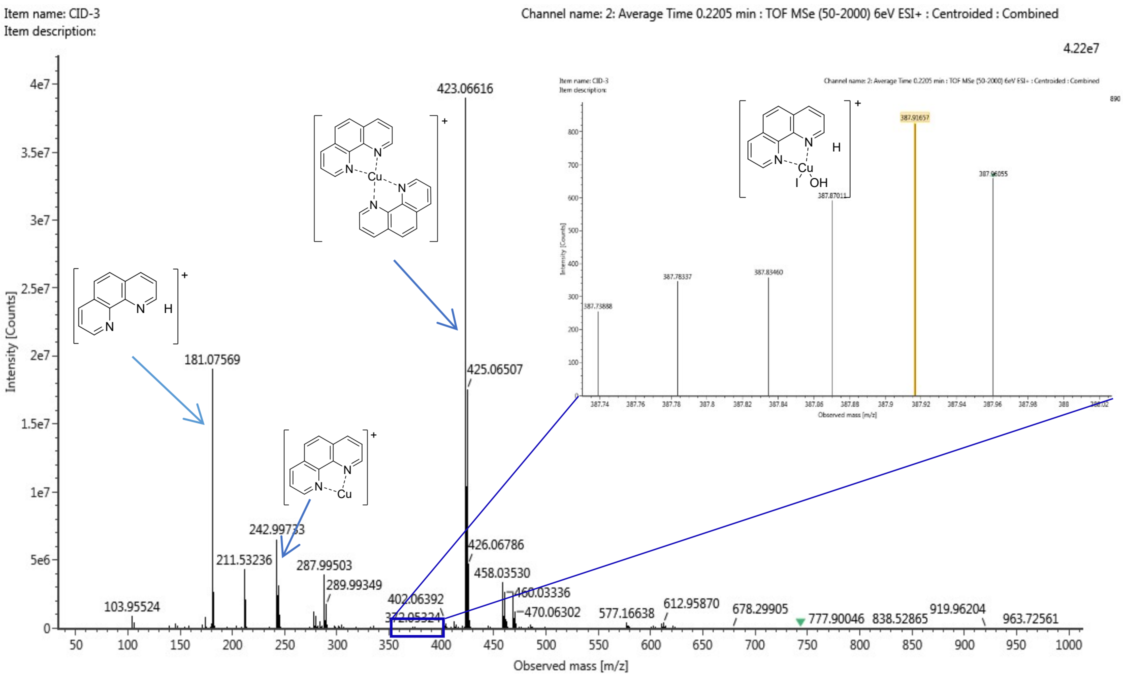

The mass spectra of **procedure 2**

The mass spectra of procedure 2. We successfully detected moderate intensity of the intermediate **[Cu(1,10-phen)(OH)I]H**$^+$ HRMS (ESI) calcd for $C_{12}H_{10}CuIN_2O$ $[M+H]^+$ 387.9128, found 387.9165.

Procedure 3: CuI (0.01 mmol) and 1,10-phen (0.015 mmol) were dissolved in MeOH (1.0 mL), and the mixture was stirred at room temperature for 30 minutes. Then hemiketal hydroperoxide **1a** (0.1 mmol) was added to the mixture, and the reaction mixture was stirred at room temperature for 30 minutes. Finally, TMSCN (0.1 mmol) was added to the mixture. After 30 minutes, 10.0 μL reaction mixture was picked up with pipette and dissolved into 1.0 mL MeCN. Next, this dilution was transferred into an injection syringe, and injected into the high-resolution electrospray mass spectrometry (I-Class VION IMS Q-Tof) by injection pump. At last, the MS data were collected and analyzed.

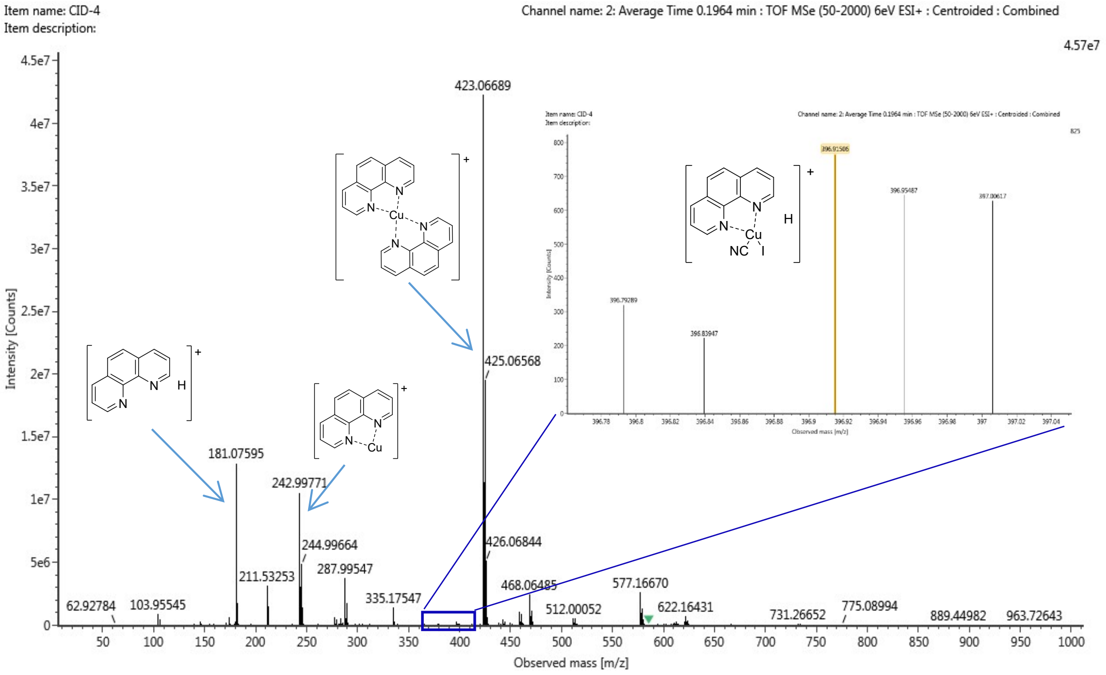

The mass spectra of **procedure 3**

The mass spectra of procedure 3. We successfully detected moderate intensity of the intermediate **[Cu(1,10-phen)(CN)I]H**$^+$ HRMS (ESI) calcd for $C_{13}H_9CuIN_3$ $[M+H]^+$ 396.9132, found 396.9150.

Related CID data:

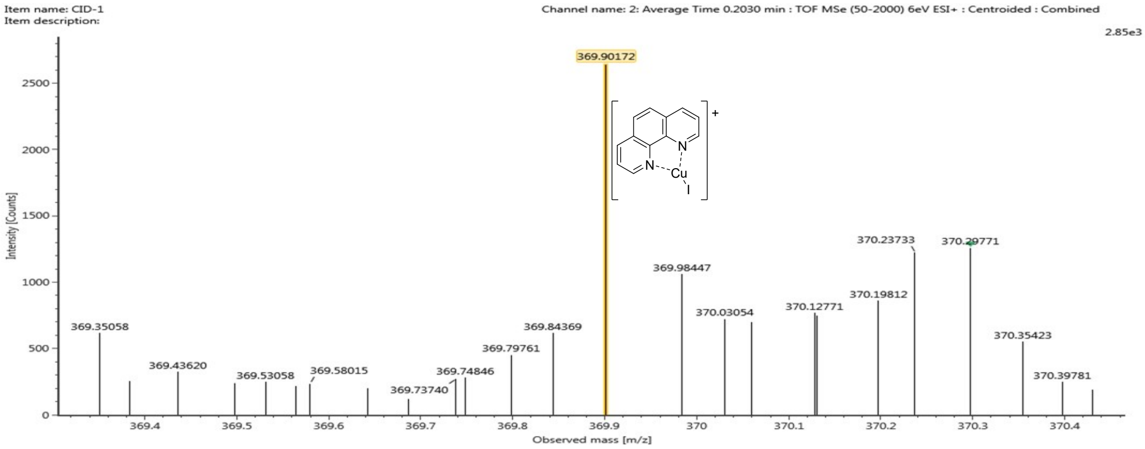

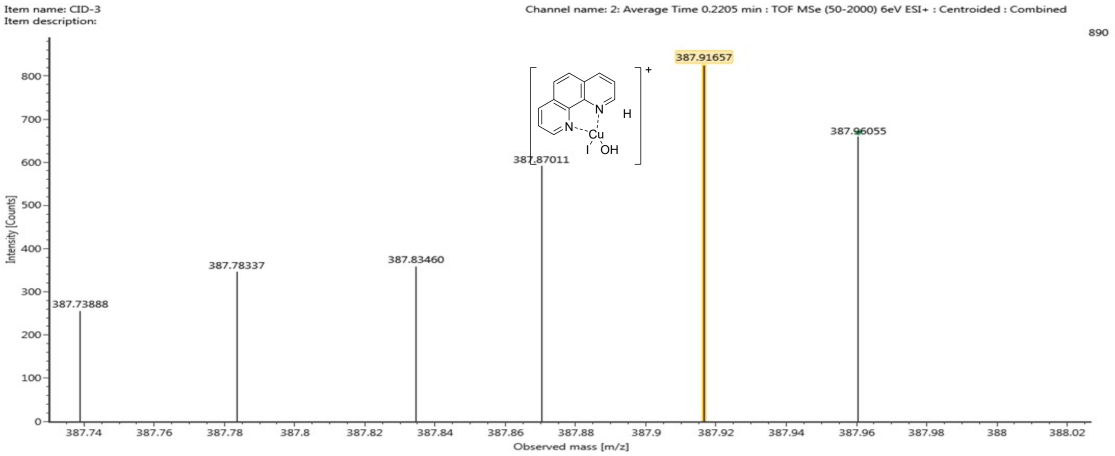

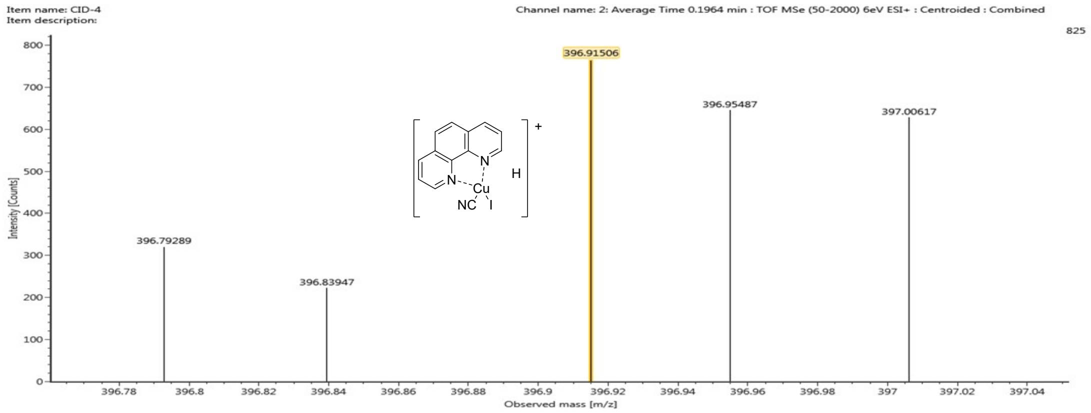
Item name: CID-4
Item description:
Channel name: 2: Average Time 0.1964 min : TOF MSe (50-2000) 6eV ESI+ : Centroided : Combined
825
396.91506
396.95487
397.00617
396.79289
396.83947
Intensity [Counts]
Observed mass [m/z]
N
Cu
NC
I
H
+

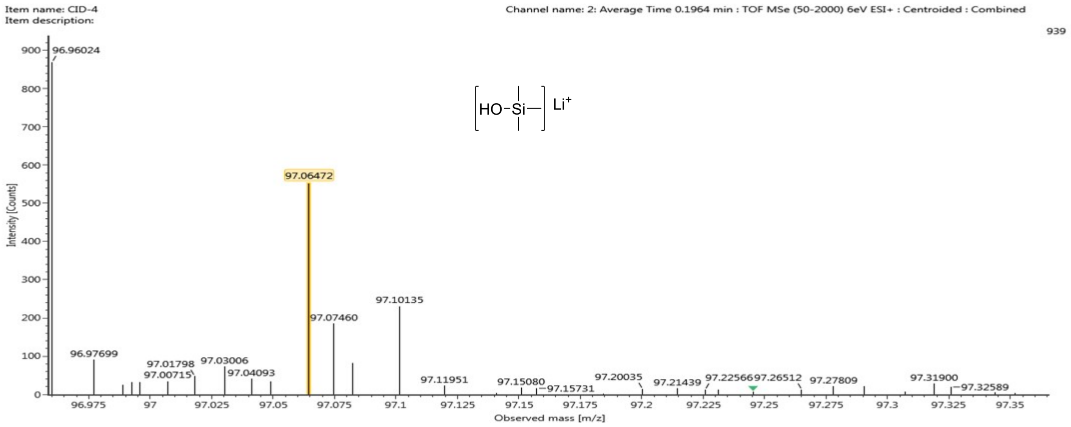
Item name: CID-4
Item description:
Channel name: 2: Average Time 0.1964 min : TOF MSe (50-2000) 6eV ESI+ : Centroided : Combined
939
96.96024
97.06472
97.10135
97.07460
96.97699
97.01798
97.00715
97.03006
97.04093
97.11951
97.15080
97.15731
97.20035
97.21439
97.22566
97.26512
97.27809
97.31900
97.32589
Intensity [Counts]
Observed mass [m/z]
HO
Si
Li+

9. Computational Details

All calculations were carried out with the Gaussian 16 Rev. A.03.[8] Molecular geometry optimizations and frequency calculations were performed by the B3LYP[9] functional with D3(BJ) dispersion correction[10], which have been proven to be particularly effective in calculating transition metal complexes. Frequency calculations were conducted to determine the stationary points (no imaginary frequencies) and transition state structures (only one imaginary frequency). A mixed basis set of SDD[11] was used for Cu and I, the 6-31G(d)[12] was used for all other atoms. The single point energies were calculated at the B3LYP-D3(BJ)/def2-TZVP[13] level with SMD[14] solvation model (methanol as solvent). All the transition states were confirmed by using the IRC (intrinsic reaction coordinate)[15] calculation. Molecular structure visualizations were generated by using CYL view 1.0[16].

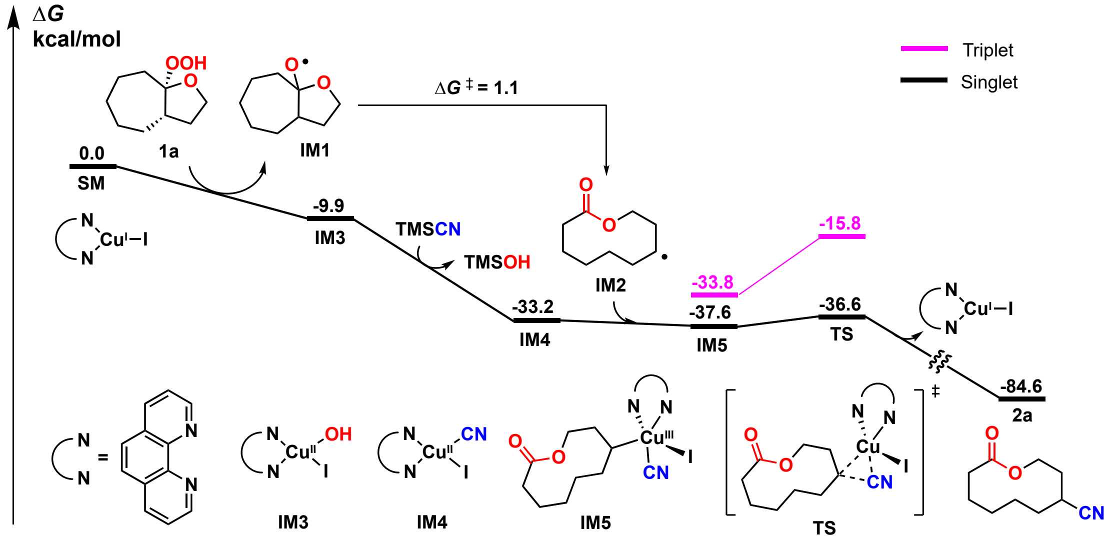

Figure S1: Computed Energy Profile for the Cu-Catalyzed Ring Expansion/Cyanation Reaction in Singlet and Triplet State. Energies are in kcal/mol.

The Cartesian Coordinates of Calculated Structures.

SM

C	-1.54475400	6.49144200	0.32439700
C	-2.12634900	7.77202900	0.40994200
C	-3.49004100	7.87869400	0.60257300
C	-4.27265700	6.70714900	0.70982500
C	-3.59440400	5.46843500	0.61081800
C	-4.33767300	4.23526000	0.71254900
C	-4.31293700	1.92174400	0.70324700
C	-5.70489500	1.86531700	0.89946600
C	-6.41820900	3.04346700	1.00310500
C	-5.73877700	4.27806900	0.91039500
C	-6.39584400	5.55084000	1.00642200
C	-5.69357700	6.71528000	0.91035000
H	-6.20101900	7.67288800	0.98446000
H	-7.47128300	5.56609200	1.15821300
H	-7.49396200	3.03391500	1.15485600
H	-6.19675300	0.90102800	0.96640600
H	-3.71950200	1.01719600	0.61733600
H	-3.96966900	8.85112000	0.67286000
H	-1.50003300	8.65349700	0.32398500
H	-0.47672100	6.36144600	0.17333500
Cu	-1.63050200	3.30706700	0.32758500
I	0.63649800	2.39776600	0.00451300
N	-3.64683400	3.06920400	0.61215500
N	-2.25722000	5.37781700	0.42187000

1a

C	-0.45115700	1.81782800	1.45542500
C	-1.47420600	0.71422400	1.78277900
C	-2.14917300	0.08889200	0.54620300
C	-1.36427800	-1.03086500	-0.17327100
C	0.15954000	-0.92479400	-0.09100100
C	0.75588100	0.46807600	-0.39763100
C	0.87897600	1.36794600	0.83308600
C	0.94437500	-1.86308100	-1.01740000
C	2.30804600	-1.17561700	-1.06068100
H	-0.21226500	2.36452900	2.37664400
H	-0.93031300	2.54008700	0.78370900
H	-1.01313100	-0.06260800	2.40987800
H	-2.25620900	1.17079100	2.40235500
H	-3.12513700	-0.31668100	0.83963200
H	-2.35274800	0.89465000	-0.16685300
H	-1.64174900	-2.00398000	0.25273800

H	-1.66310600	-1.05379300	-1.22617200
H	0.46564600	-1.15393200	0.93833900
H	1.47171000	0.80732800	1.56594400
H	1.47002000	2.24487700	0.54855900
H	1.00544700	-2.88751900	-0.63695600
H	0.48588000	-1.88343800	-2.00960500
H	2.86706500	-1.34684800	-1.98590600
H	2.93851600	-1.48588700	-0.21524900
H	0.87859700	0.66271900	-2.87599600
O	2.04516800	0.23362600	-0.96034400
O	0.01040100	1.25452100	-1.32360300
O	-0.05179700	0.55894800	-2.59462500

2a

C	4.67450900	0.87270900	-1.45414600
C	3.45879700	0.11343600	-2.02105700
H	3.39525900	0.27230400	-3.10398200
H	2.54349200	0.54648000	-1.60020900
C	3.52663000	-1.40257300	-1.74344900
H	4.06306200	-1.58573200	-0.80755000
H	4.14394700	-1.87100700	-2.51997600
C	2.17350900	-2.12459500	-1.70571300
C	1.30136000	-1.74417500	-0.47982200
C	1.97466800	-2.08493500	0.88565300
H	1.39335600	-2.84042900	1.42364700
H	2.96316300	-2.51618700	0.71128000
C	2.11830100	-0.86379200	1.78943900
H	1.14279700	-0.48917400	2.10817900
O	2.70758800	0.24629100	1.07665200
C	4.05793400	0.24331600	0.93329700
O	4.78596000	-0.57750400	1.44855900
C	4.49448500	1.35602000	0.00629300
H	3.74552300	2.15274000	0.03555600
H	5.44368300	1.75068600	0.38013200
H	2.72796900	-1.09659900	2.66527700
H	1.12518500	-0.66310400	-0.51550200
C	-0.01359900	-2.38527400	-0.58733500
N	-1.04836000	-2.90543200	-0.67116600
H	2.33666500	-3.20882600	-1.68685000
H	1.60679600	-1.90856700	-2.61945900
H	5.56540900	0.23562400	-1.51352500
H	4.88417800	1.76078000	-2.06064800

IM1

C	-0.48075400	1.84916900	1.43538100
C	-1.50541000	0.75249500	1.77482500
C	-2.14531300	0.08678700	0.54250600
C	-1.34709400	-1.06341000	-0.10423800
C	0.17515300	-0.94027500	-0.06829200
C	0.79914000	0.48231800	-0.38521700
C	0.86536800	1.38024700	0.88376100
C	0.92989700	-1.81226000	-1.07596200
C	2.32441900	-1.18012300	-1.09379100
H	-0.27156800	2.43138000	2.34262400
H	-0.93940200	2.54326100	0.72099900
H	-1.05189000	-0.00330200	2.43282900
H	-2.30390700	1.22032000	2.36386100
H	-3.13280800	-0.30484200	0.81554700
H	-2.31689100	0.86363200	-0.20980200
H	-1.59717900	-2.00805300	0.39728600
H	-1.67526900	-1.17374400	-1.14448100
H	0.53804300	-1.17033000	0.94106400
H	1.41693900	0.80007600	1.63226300
H	1.48809200	2.24326700	0.62611500
H	0.94832200	-2.86820200	-0.78750100
H	0.45138400	-1.73397900	-2.05852800
H	2.81279800	-1.25498200	-2.07146100
H	2.98264800	-1.64546800	-0.34628600
O	2.15081500	0.20056700	-0.76114900
O	0.08462000	1.06991400	-1.34985200

IM2

C	-0.31628500	1.79888300	1.46131400
C	-1.39975700	0.71687400	1.60311800
C	-1.87979900	0.06784100	0.28170000
C	-1.49787700	-1.43145500	0.13523500
C	-0.04229500	-1.74449900	0.25342900
C	0.87292800	1.38849500	-0.74489700
C	0.98069600	1.33276500	0.77536900
C	0.89089600	-1.89138400	-0.90315000
C	1.97595200	-0.80594000	-1.02150500
H	-0.06392100	2.16671400	2.46315100
H	-0.70616100	2.65183100	0.89645300
H	-1.03341600	-0.06431700	2.28321800
H	-2.25404000	1.17536500	2.11549600
H	-2.97291100	0.13058200	0.21500700
H	-1.50049900	0.62695100	-0.57854200
H	-2.04773400	-1.98823400	0.90852200

H	-1.87330100	-1.78438200	-0.83499100
H	0.38401100	-1.84928700	1.24966700
H	1.27475900	0.35171600	1.14896900
H	1.79224000	2.02817700	1.02945900
H	1.42857100	-2.85229900	-0.83350600
H	0.33608100	-1.90648000	-1.84909900
H	2.71088600	-1.09983500	-1.77603700
H	2.50671700	-0.66208400	-0.07515500
O	1.44137100	0.42719800	-1.51669700
O	0.33740900	2.31613700	-1.30798300

IM3

C	-1.54661200	6.31846300	0.25104800
C	-2.06299800	7.62098300	0.39124200
C	-3.41021100	7.79712300	0.63708600
C	-4.24879200	6.66530900	0.74396200
C	-3.63949600	5.39940300	0.58865000
C	-4.44114700	4.21215700	0.68705200
C	-4.47451400	1.89781100	0.60724900
C	-5.86252100	1.88441900	0.85332000
C	-6.53691800	3.08048200	1.01746800
C	-5.82871500	4.30136500	0.93689300
C	-6.41930900	5.60045200	1.09002900
C	-5.66122700	6.73153900	0.99735100
H	-6.11776700	7.71002500	1.11475700
H	-7.48624500	5.66735000	1.28202800
H	-7.60659600	3.09383200	1.20800100
H	-6.38534200	0.93596600	0.91104800
H	-3.87253500	1.00451300	0.46716300
H	-3.83285200	8.79180100	0.74914300
H	-1.39175100	8.46819800	0.30354600
H	-0.49465300	6.13309100	0.05731900
Cu	-1.73567200	3.20196300	0.16884100
I	0.82402800	3.63062200	-0.26710200
O	-1.69572600	1.36900200	0.09202800
H	-0.78201600	1.09321300	-0.08000800
N	-3.80056000	3.03578600	0.52968500

IM4

C	-1.39775400	1.42710800	-0.15932400
N	-1.49474000	0.28498300	-0.38497600
C	-1.51768600	6.34955500	0.23408900
C	-2.06672100	7.64326600	0.30706600
C	-3.41902800	7.79497200	0.53962700

C	-4.23078700	6.64894300	0.69240800
C	-3.59306800	5.39086300	0.59443000
C	-4.36907500	4.18951700	0.72630700
C	-4.37693700	1.87297500	0.70682400
C	-5.76640400	1.84766400	0.93994400
C	-6.45639700	3.03776500	1.06519800
C	-5.76166800	4.26406000	0.95516900
C	-6.38016300	5.55475500	1.05802200
C	-5.64549600	6.69724000	0.93193400
H	-6.12247400	7.66985500	1.00834300
H	-7.45012700	5.60458500	1.23707200
H	-7.52813500	3.04404500	1.24323500
H	-6.27501900	0.89287700	1.01443400
H	-3.78886500	0.96846000	0.58086200
H	-3.86712900	8.78244600	0.60614700
H	-1.41624800	8.50201000	0.18404700
H	-0.45645200	6.18359800	0.07991100
Cu	-1.61516600	3.26886500	0.30256700
N	-3.70795100	3.01494500	0.61194800
N	-2.25883600	5.25547900	0.37329500
I	0.90189300	3.75218800	0.84179100

IM5

C	0.64000100	1.16612400	-1.52672300
C	0.59398900	-0.17952300	-2.27938500
H	0.57824000	0.02060400	-3.35715200
H	-0.36603000	-0.66290400	-2.06367900
C	1.76994300	-1.14193500	-1.96840800
H	2.28910800	-0.81643800	-1.06236400
H	2.51320100	-1.07031200	-2.77163700
C	1.36707800	-2.62984400	-1.81978600
C	0.53071400	-2.81216000	-0.56694400
C	1.30846300	-2.77908000	0.74496000
H	1.50528400	-3.78450000	1.12885800
H	2.29329400	-2.34120900	0.54215800
C	0.62778100	-1.94304500	1.83768200
H	-0.17213000	-2.48862400	2.33584600
O	-0.04168600	-0.79408500	1.26132900
C	0.75661700	0.21127100	0.82456100
O	1.92875600	0.31103500	1.11706800
C	0.01680300	1.14426400	-0.11141600
H	-1.03678400	0.85313700	-0.16457900
H	0.07298800	2.15309900	0.31362800
H	1.35460200	-1.60532100	2.57929600

H	-0.26647800	-2.07592400	-0.55588800
C	0.61298500	-5.32536400	-0.94592600
N	1.58076300	-5.95260100	-1.12448400
C	-2.50757400	-3.26527400	1.62569100
C	-3.33712900	-2.36818700	2.31687600
C	-3.90507100	-1.31304200	1.62909300
C	-3.67987900	-1.18421300	0.24260700
C	-2.84678300	-2.14819500	-0.37553900
C	-2.64575600	-2.11034900	-1.80377400
C	-1.72667500	-3.10094200	-3.66956900
C	-2.28015900	-2.12010900	-4.51663100
C	-3.03662700	-1.10593700	-3.96204300
C	-3.25144100	-1.08054000	-2.56641400
C	-4.04497600	-0.08640600	-1.90272100
C	-4.25603300	-0.14060300	-0.55696800
H	-4.87531900	0.60350700	-0.06476200
H	-4.49124100	0.70395000	-2.49938000
H	-3.47674500	-0.33269500	-4.58557000
H	-2.10707100	-2.17241400	-5.58610300
H	-1.12984700	-3.91710400	-4.06732700
H	-4.53618200	-0.59053300	2.13861900
H	-3.51045000	-2.50986200	3.37772400
H	-2.04536500	-4.11037200	2.12478400
H	2.25785200	-3.26157000	-1.77527500
H	0.80643400	-2.93771300	-2.70688800
H	1.67603200	1.51586500	-1.44923500
H	0.09315300	1.92350900	-2.10087300
Cu	-0.88882700	-4.30334900	-0.64536500
N	-2.24651600	-3.13722600	0.32950500
N	-1.89652600	-3.08795100	-2.35705400
I	-2.58453800	-6.31025800	-0.67183000

TS

C	0.06730800	1.26577000	-0.53124700
C	0.08693700	0.14845900	-1.59602000
H	0.12603100	0.59423300	-2.57467500
H	0.75339100	-0.53086300	-1.40388300
C	1.42035700	-0.64081100	-1.68938300
H	2.03379600	-0.43753400	-0.80665100
H	1.99997800	-0.26498300	-2.54096800
C	1.25911800	-2.17129000	-1.85785600
C	0.66699800	-2.75338100	-0.59072700
C	1.59735100	-2.87627300	0.61002200
H	2.03400000	-3.87419800	0.69295200

H	2.43332700	-2.18358400	0.46053300
C	0.90225000	-2.51819900	1.92609900
H	0.22589000	-3.31063000	2.25250900
O	0.03602700	-1.36804100	1.74528800
C	0.63886700	-0.18206500	1.48287300
O	1.82043400	0.02322300	1.65807300
C	0.33599300	0.81649600	0.89287200
H	1.34170500	0.38575400	0.88608300
H	0.34521700	1.69586100	1.54730800
H	1.62942400	-2.30885800	2.71347900
H	0.20519200	-2.16700000	-0.31934100
C	0.71424400	-4.86850300	-1.25809500
N	1.60984500	-5.40621700	-1.78305700
C	-2.67579800	-2.73141400	1.29586400
C	-3.60530900	-1.76011800	1.70485100
C	-4.22489200	-0.97939800	0.74906600
C	-3.93619000	-1.18955800	-0.61722500
C	-2.99414400	-2.19820400	-0.93613200
C	-2.69490800	-2.48034700	-2.31960600
C	-1.52999000	-3.77082300	-3.83747900
C	-2.09913000	-3.07880700	-4.92554200
C	-2.99264200	-2.05511500	-4.67511100
C	-3.32250900	-1.72563900	-3.34135600
C	-4.24804400	-0.68990800	-2.98281300
C	-4.54573900	-0.43729000	-1.67675800
H	-5.25694100	0.34019900	-1.41331500
H	-4.71740000	-0.11562100	-3.77640000
H	-3.44943500	-1.50163100	-5.49094800
H	-1.83104100	-3.35550400	-5.93949400
H	-0.81655400	-4.57754500	-3.98421600
H	-4.94065200	-0.21280400	1.03265800
H	-3.81499700	-1.63340100	2.76098500
H	-2.17660900	-3.37281100	2.01283600
H	2.22319100	-2.63780000	-2.07781400
H	0.60357900	-2.37315700	-2.70988700
H	1.04983500	1.74933700	-0.48212600
H	-0.64959100	2.03920000	-0.83110900
Cu	-0.88629200	-4.21399800	-0.64195500
N	-2.36458600	-2.92768900	0.02097800
N	-1.82185400	-3.47646500	-2.58013800
I	-2.08928100	-6.35267600	0.22007600

TMSCN

Si	-0.99502900	0.56596300	-0.05984900

C	-0.37154700	1.44820500	1.46788000
N	0.01607700	1.99684800	2.41786100
C	-0.32933600	1.50763900	-1.54351000
H	-0.66264000	1.03652700	-2.47591100
H	-0.68073500	2.54490400	-1.54558500
H	0.76573300	1.52217400	-1.54585700
C	-2.87225800	0.60838600	0.01371600
H	-3.30033700	0.10359800	-0.86055300
H	-3.24593900	0.10362900	0.91092400
H	-3.24592600	1.63777400	0.02511900
C	-0.32911700	-1.18965700	0.01395900
H	-0.68086700	-1.71029600	0.91095700
H	-0.66190500	-1.76145600	-0.86051100
H	0.76595100	-1.19878500	0.02589600

TMSI

Si	-0.99919900	0.56017700	-0.06995300
C	-0.33497300	1.49972400	-1.55516000
H	-0.67323200	1.02244500	-2.48415700
H	-0.68733400	2.53614500	-1.55616800
H	0.75963500	1.51217600	-1.55692300
C	-2.87618700	0.60109500	0.00115100
H	-3.29704000	0.09392700	-0.87686700
H	-3.24814500	0.09559600	0.89805800
H	-3.24826400	1.63054700	0.01145900
C	-0.33462900	-1.19565300	0.00138400
H	-0.68840000	-1.71533500	0.89754300
H	-0.67114600	-1.76114000	-0.87738000
H	0.75996000	-1.20301900	0.01324200
I	-0.16046700	1.74716800	1.98534600

TMSOH

Si	-0.98112200	0.60353400	-0.01548400
C	-0.35791800	1.50289300	-1.55097800
H	-0.71595500	1.00952700	-2.46322900
H	-0.70308900	2.54360300	-1.58733300
H	0.73748700	1.51292900	-1.58455400
C	-2.86656200	0.61582400	-0.01467100
H	-3.25953900	0.11059100	-0.90580200
H	-3.26373400	0.09760300	0.86558500
H	-3.27183300	1.63528000	-0.01364000
C	-0.33132500	-1.15461100	0.03061900
H	-0.68163300	-1.67887500	0.92660700
H	-0.66572900	-1.72370600	-0.84496100

H	0.76414600	-1.16779100	0.04223600
O	-0.38044600	1.32209100	1.38033500
H	-0.62584500	2.24755600	1.51435000

10. Characterization of Hemiketal Hydroperoxides 1

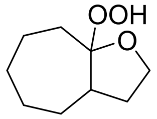

8a-Hydroperoxyoctahydro-2*H*-cyclohepta[b]furan (1a) White solid (1.0 g, 58%, two steps). Melting point (°C): 46-47. R_f = 0.3 (petroleum ether/ethyl acetate = 5:1). ^{1}H NMR (400 MHz, $CDCl_3$) δ 8.59 (br, 1H), 3.95 – 3.90 (m, 1H), 3.84 – 3.77 (m, 1H), 2.41 – 2.36 (m, 1H), 2.15 – 1.99 (m, 2H), 1.75 – 1.71 (m, 3H), 1.63 – 1.47 (m, 4H), 1.42 – 1.33 (m, 1H), 1.29 – 1.24 (m, 2H). ^{13}C NMR (100 MHz, $CDCl_3$) δ 117.9, 66.9, 47.5, 34.4, 32.3, 31.7, 31.2, 28.3, 23.2. IR (neat): υ_{max} (cm^{-1}) 2938, 1734, 1267, 755. HRMS (ESI) calcd for $C_9H_{16}O_3Li$ [M+Li]$^+$ 179.1254, found 179.1262.

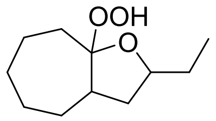

2-Ethyl-8a-hydroperoxyoctahydro-2*H*-cyclohepta[b]furan (1b) Colorless oil (1.3 g, 65%, two steps). R_f = 0.3 (petroleum ether/ethyl acetate = 7:1). ^{1}H NMR (400 MHz, $CDCl_3$) δ 8.00 (br, 0.6H), 4.15 – 3.91 (m, 1H), 2.48 – 2.33 (m, 1H), 2.20 – 2.15 (m, 1H), 2.10 – 1.99 (m, 1H), 1.78 – 1.61 (m, 6H), 1.53 – 1.46 (m, 2H), 1.39 – 1.24 (m, 3H), 1.19 – 1.14 (m, 1H), 0.94 – 0.90 (m, 3H). ^{13}C NMR (100 MHz, $CDCl_3$) major isomer δ 117.6, 79.4, 47.8, 39.7, 32.5, 31.9, 31.3, 28.2, 27.5, 23.3, 10.1; minor isomer δ 118.5, 82.1, 47.5, 39.1, 32.7, 32.1, 31.1, 29.8, 29.3, 23.3, 10.6. IR (neat): υ_{max} (cm^{-1}) 2895, 1268, 755. HRMS (ESI) calcd for $C_{11}H_{20}O_3K$ [M+K]$^+$ 239.1044, found 239.1046.

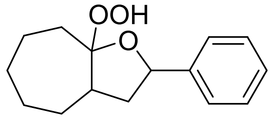

8a-Hydroperoxy-2-phenyloctahydro-2*H*-cyclohepta[b]furan (1c) Colorless oil (0.7 g, 28%, two steps). R_f = 0.3 (petroleum ether/ethyl acetate = 5:1). ^{1}H NMR (400 MHz, $CDCl_3$) δ 8.62 (br, 0.7H), 7.31 – 7.20 (m, 5H), 4.18 – 4.10 (m, 1H), 3.98 – 3.93 (m, 1H), 2.98 – 2.91 (m, 1H), 2.48 – 2.42 (dd, *J* = 14.0, 7.6 Hz, 1H), 2.13 – 2.07 (m, 1H), 1.75 – 1.56 (m, 6H), 1.44 – 1.19 (m, 3H). ^{13}C NMR (100 MHz, $CDCl_3$) δ 139.0, 128.8, 128.1, 127.2, 118.1, 73.0, 56.2, 32.0, 31.4, 30.6, 27.9, 23.3. IR (neat): υ_{max} (cm^{-1}) 2967, 1756, 1421, 1267, 755, 689. HRMS (ESI) calcd for $C_{15}H_{24}NO_3$ [M+NH_4]$^+$ 266.1751, found 266.1735.

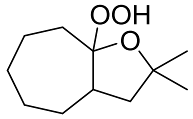

8a-Hydroperoxy-2,2-dimethyloctahydro-2*H*-cyclohepta[b]furan (1d) Colorless oil (0.4 g, 20%, two steps). R_f = 0.3 (petroleum ether/ethyl acetate = 8:1). ^{1}H NMR (400 MHz, $CDCl_3$) δ 7.71 (br, 1H), 2.37 – 2.32 (m, 1H), 2.29 – 2.21 (m, 1H), 2.10 (dd, *J* = 12.4, 8.8 Hz, 1H), 1.80 – 1.71 (m, 3H), 1.67 – 1.42 (m, 6H), 1.36 (s, 3H), 1.29 (s, 3H), 1.27 – 1.26 (m, 1H). ^{13}C NMR (100 MHz, $CDCl_3$) δ 118.4, 82.3, 48.1, 46.7, 32.8, 32.7, 31.3, 29.8, 28.6, 28.3, 23.3. IR (neat): υ_{max} (cm^{-1}) 2894, 1730, 1266, 753. HRMS (ESI) calcd for $C_{11}H_{24}NO_3$ $[M+NH_4]^+$ 218.1751, found 218.1762.

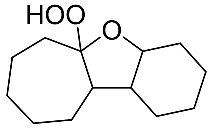

5a-Hydroperoxydodecahydro-1*H*-cyclohepta[b]benzofuran (1e) White solid (1.03 g, 46%, two steps). Melting point (°C): 92-93. R_f = 0.3 (petroleum ether/ethyl acetate = 10:1). ^{1}H NMR (400 MHz, $CDCl_3$) δ 8.63 (s, 0.2H), 8.47 (s, 0.7H), 3.49 – 3.40 (m, 1H), 2.55 – 2.29 (m, 1H), 2.14 – 1.95 (m, 2H), 1.89 – 1.57 (m, 10H), 1.41 – 1.31 (m, 2H), 1.29 – 1.08 (m, 5H). ^{13}C NMR (100 MHz, $CDCl_3$) major isomer δ 118.3, 82.1, 50.2, 48.2, 33.7, 32.3, 31.2, 30.6, 25.8, 25.7, 24.9, 24.2, 23.5. minor isomer δ 117.5, 81.0, 52.9, 52.5, 35.1, 31.6, 30.7, 30.0, 27.9, 27.2, 26.0, 25.1, 24.3. IR (neat): υ_{max} (cm^{-1}) 2978, 2298, 1268, 755. HRMS (ESI) calcd for $C_{13}H_{26}NO_3$ $[M+NH_4]^+$ 244.1907, found 244.1921.

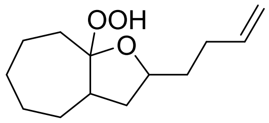

2-(But-3-en-1-yl)-8a-hydroperoxyoctahydro-2H-cyclohepta[b]furan (1f) Colorless oil (0.8 g, 35%, two steps). R_f = 0.3 (petroleum ether/ethyl acetate = 10:1). ^{1}H NMR (400 MHz, $CDCl_3$) δ 7.91 (s, 0.14H), 7.75 (s, 0.85H), 5.89 – 5.78 (m, 1H), 5.06 – 4.94 (m, 2H), 4.24 – 4.17 (m, 0.88H), 4.04 – 3.97 (m, 0.13H), 2.46 – 2.33 (m, 1H), 2.22 – 1.98 (m, 4H), 1.85 – 1.67 (m, 4H), 1.65 – 1.51 (m, 5H), 1.47 – 1.24 (m, 3H). ^{13}C NMR (100 MHz, $CDCl_3$) δ 138.3, 118.6, 115.0, 80.1, 47.5, 39.5, 36.1, 32.6, 32.2, 31.2, 30.7, 29.2, 23.2. IR (neat): υ_{max} (cm^{-1}) 2922, 2098, 1722, 1640, 1448, 1006, 910, 639. HRMS (ESI) calcd for $C_{13}H_{22}O_3K$ $[M+K]^+$ 265.1200, found 265.1191.

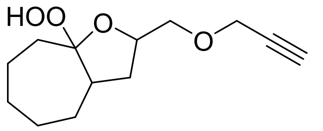

8a-Hydroperoxy-2-((prop-2-yn-1-yloxy)methyl)octahydro-2H-cyclohepta[b]furan (1g) Colorless oil (0.7 g, 29%, two steps). R_f = 0.3 (petroleum ether/ethyl acetate = 10:1). ^{1}H NMR (400 MHz, $CDCl_3$)

δ 8.59 (s, 1H), 4.46 – 4.40 (m, 1H), 4.28 – 4.18 (m, 2H), 3.68 – 3.59 (m, 2H), 2.67 (t, *J* = 2.4 Hz, 1H), 2.44 (t, *J* = 6.4 Hz, 1H), 2.27 – 2.15 (m, 2H), 1.84 – 1.74 (m, 2H), 1.68 – 1.52 (m, 5H), 1.46 – 1.26 (m, 3H). ^{13}C NMR (100 MHz, $CDCl_3$) δ 118.1, 79.6, 76.67, 74.9, 71.8, 58.7, 47.3, 36.5, 32.5, 31.8, 31.2, 28.2, 23.2. IR (neat): υ_{max} (cm^{-1}) 2937, 2176, 1722, 1407, 1260, 669. HRMS (ESI) calcd for $C_{13}H_{24}NO_4$ $[M+NH_4]^+$ 258.1700, found 258.1714.

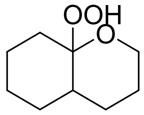

8a-Hydroperoxyoctahydro-2*H*-chromene (1h) Colorless oil (0.73 g, 42%, two steps). R_f = 0.3 (petroleum ether/ethyl acetate = 6:1). ^{1}H NMR (400 MHz, $CDCl_3$) δ 7.46 (br, 0.6H), 3.94 – 3.87 (m, 1H), 3.74 – 3.70 (m, 1H), 2.27 – 2.20 (m, 1H), 1.76 – 1.68 (m, 2H), 1.65 – 1.56 (m, 4H), 1.53 – 1.47 (m, 1H), 1.39 – 1.33 (m, 3H), 1.27 – 1.19 (m, 2H). ^{13}C NMR (100 MHz, $CDCl_3$) major isomer δ 103.9, 61.8, 43.7, 32.3, 29.5, 26.3, 25.9, 24.9, 22.8; minor isomer δ 104.3, 62.2, 35.6, 33.3, 28.3, 25.1, 24.2, 22.9, 20.3. IR (neat): υ_{max} (cm^{-1}) 2928, 1268, 1078, 755. HRMS (ESI) calcd for $C_9H_{16}O_3Li$ $[M+Li]^+$ 179.1254, found 179.1244.

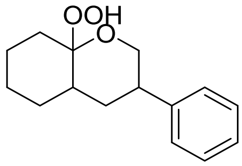

8a-Hydroperoxy-3-phenyloctahydro-2*H*-chromene (1i) Colorless oil (0.5 g, 20%, two steps) R_f = 0.3 (petroleum ether/ethyl acetate = 8:1). ^{1}H NMR (400 MHz, $CDCl_3$) δ 7.34 (br, 0.6H), 7.34 – 7.30 (m, 2H), 7.25 – 7.22 (m, 3H), 3.93 – 3.77 (m, 2H), 2.20 – 2.98 (m, 1H), 2.38 – 2.35 (m, 1H), 1.90 – 1.77 (m, 3H), 1.71 – 1.60 (m, 3H), 1.55 – 1.46 (m, 2H), 1.37 – 1.26 (m, 2H). ^{13}C NMR (100 MHz, $CDCl_3$) δ 141.7, 128.7, 127.5, 126.9, 103.3, 66.8, 43.6, 43.4, 32.1, 31.7, 29.4, 25.9, 22.9. IR (neat): υ_{max} (cm^{-1}) 2893, 1271, 755, 701. HRMS (ESI) calcd for $C_{15}H_{21}O_3$ $[M+H]^+$ 249.1485, found 249.1499.

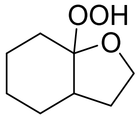

7a-Hydroperoxyoctahydrobenzofuran (1j) Colorless oil (0.79 g, 50%, two steps). R_f = 0.3 (petroleum ether/ethyl acetate = 5:1). ^{1}H NMR (400 MHz, $CDCl_3$) δ 8.15 (br, 0.5H), 4.08 – 3.99 (m, 2H), 2.36 – 2.19 (m, 2H), 2.03 – 1.97 (m, 1H), 1.73 – 1.50 (m, 5H), 1.32 – 1.03 (m, 3H). ^{13}C NMR (100 MHz, $CDCl_3$)

δ 113.1, 66.9, 40.2, 30.7, 29.69, 29.65, 24.0, 22.4. IR (neat): υ_{max} (cm^{-1}) 2987, 2360, 1268, 755. HRMS (ESI) calcd for $C_8H_{18}NO_3$ $[M+NH_4]^+$ 176.1281, found 176.1269.

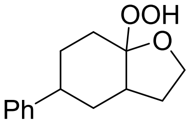

7a-Hydroperoxy-5-phenyloctahydrobenzofuran (1k) White solid (0.93 g, 40%, two steps). Melting point (°C): 79-80. R_f = 0.3 (petroleum ether/ethyl acetate = 3:1). ^1H NMR (400 MHz, $CDCl_3$) δ 8.38 (br, 0.5H), 7.33 – 7.28 (m, 2H), 7.23 – 7.20 (m, 3H), 4.20 – 4.10 (m, 2H), 2.63 – 2.57 (m, 2H), 2.37 – 2.35 (m, 1H), 2.28 – 2.22 (m, 1H), 1.94 – 1.86 (m, 3H), 1.68 – 1.57 (m, 2H), 1.39 – 1.29 (m, 1H). ^{13}C NMR (100 MHz, $CDCl_3$) δ 146.1, 128.6, 126.8, 126.4, 112.6, 67.1, 42.3, 40.9, 38.0, 30.7, 30.2, 29.9. IR (neat): υ_{max} (cm^{-1}) 2893, 2378, 1267, 755. HRMS (ESI) calcd for $C_{14}H_{19}O_3$ $[M+H]^+$ 235.1329, found 235.1334.

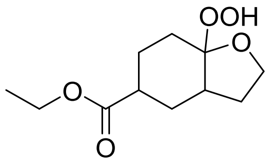

Ethyl 7a-hydroperoxyoctahydrobenzofuran-5-carboxylate (1l) Colorless oil (0.85 g, 37%, two steps). R_f = 0.3 (petroleum ether/ethyl acetate = 2:1). ^1H NMR (400 MHz, $CDCl_3$) δ 8.37 (br, 0.4H), 4.15 – 3.99 (m, 4H), 2.61 – 2.45 (m, 1H), 2.34 – 2.04 (m, 3H), 2.02 – 1.79 (m, 2H), 1.76 – 1.35 (m, 3H), 1.32 – 1.19 (m, 4H). ^{13}C NMR (100 MHz, $CDCl_3$) δ 175.3, 112.2, 66.9, 60.6, 41.4, 39.9, 31.9, 30.6, 29.0, 25.4, 14.3. IR (neat): υ_{max} (cm^{-1}) 2923, 1725, 1267, 755. HRMS (ESI) calcd for $C_{11}H_{18}O_5Na$ $[M+Na]^+$ 253.1046, found 253.1028.

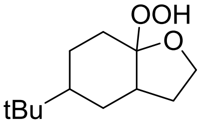

5-(*tert*-butyl)-7a-Hydroperoxyoctahydrobenzofuran (1m) White solid (1.1 g, 51%, two steps). Melting point (°C): 50-51. R_f = 0.3 (petroleum ether/ethyl acetate = 5:1). ^1H NMR (400 MHz, $CDCl_3$) δ 8.00 (br, 0.3H), 4.09 – 3.99 (m, 2H), 2.43 – 2.40 (m, 1H), 2.32 – 2.23 (m, 1H), 2.04 – 1.98 (m, 1H), 1.74 – 1.63 (m, 3H), 1.55 – 1.50 (m, 1H), 1.08 – 0.99 (m, 2H), 0.83 – 0.81 (m, 10H). ^{13}C NMR (100 MHz, $CDCl_3$) δ 113.1, 67.0, 46.0, 41.2, 32.3, 31.3, 31.1, 30.1, 27.6, 23.5. IR (neat): υ_{max} (cm^{-1}) 2921, 1268, 755. HRMS (ESI) calcd for $C_{12}H_{22}O_3Na$ $[M+Na]^+$ 237.1461, found 237.1461.

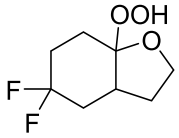

5,5-Difluoro-7a-hydroperoxyoctahydrobenzofuran (1n) White solid (1.1 g, 57%, two steps). Melting point (°C): 64-65. R_f = 0.3 (petroleum ether/ethyl acetate = 5:1). ^{1}H NMR (400 MHz, $CDCl_3$) δ 8.44 (br, 1H), 4.14 – 4.06 (m, 2H), 2.46 – 2.27 (m, 3H), 2.20 – 1.98 (m, 3H), 1.95 – 1.78 (m, 1H), 1.67 – 1.50 (m, 2H). ^{13}C NMR (100 MHz, $CDCl_3$) δ 122.8 (t, *J* = 239 Hz), 111.5, 67.1, 38.3 (d, *J* = 9 Hz), 35.9 (t, *J* = 24 Hz), 30.1, 30.0 (t, *J* = 25 Hz), 26.1 (d, *J* = 8 Hz). IR (neat): υ_{max} (cm^{-1}) 2918, 1369, 1267, 755. HRMS (ESI) calcd for $C_8H_{12}F_2O_3Na$ $[M+Na]^+$ 217.0647, found 217.0645.

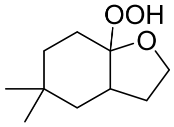

7a-Hydroperoxy-5,5-dimethyloctahydrobenzofuran (1o) Colorless oil (0.56 g, 30%, two steps). R_f = 0.3 (petroleum ether/ethyl acetate = 5:1). ^{1}H NMR (400 MHz, $CDCl_3$) δ 8.73 – 7.39 (br, 0.4H), 4.08 – 3.98 (m, 2H), 2.33 – 2.23 (m, 2H), 2.19 – 2.12 (m, 1H), 1.90 – 1.82 (m, 1H), 1.51 – 1.46 (m, 1H), 1.40 – 1.23 (m, 3H), 0.99 (d, *J* = 12.8 Hz, 1H), 0.91 (s, 3H), 0.90 (s, 3H). ^{13}C NMR (100 MHz, $CDCl_3$) δ 113.0, 66.7, 42.7, 37.6, 35.1, 31.9, 30.6, 30.1, 26.3, 23.3. IR (neat): υ_{max} (cm^{-1}) 2923, 1268, 755. HRMS (ESI) calcd for $C_{10}H_{18}O_3Na$ $[M+Na]^+$ 209.1148, found 209.1163.

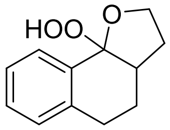

9b-Hydroperoxy-2,3,3a,4,5,9b-hexahydronaphtho[1,2-b] furan (1p) White solid (0.37 g, 18%, two steps). Melting point (°C): 70-71. R_f = 0.3 (petroleum ether/ethyl acetate = 5:1).^{1}H NMR (400 MHz, $CDCl_3$) δ 8.03 (d, *J* = 8.0 Hz, 1H), 7.47 (td, *J* = 7.6, 1.2 Hz, 1H), 7.31 (t, *J* = 7.6 Hz, 1H), 7.24 (d, *J* = 7.6 Hz, 1H), 3.87 – 3.84 (m, 2H), 3.10 – 2.96 (m, 2H), 2.73 – 2.66 (m, 1H), 2.40 (br, 1H), 2.27 – 2.15 (m, 2H), 2.02 – 1.92 (m, 1H), 1.79 – 1.72 (m, 1H). ^{13}C NMR (100 MHz, $CDCl_3$) δ144.2, 133.5, 132.3, 128.7, 127.6, 126.7, 100.0, 61.3, 46.0, 33.3, 29.7, 28.9. IR (neat): υ_{max} (cm^{-1}) 2923, 1268, 755. HRMS (ESI) calcd for $C_{12}H_{14}O_3Na$ $[M+Na]^+$ 229.0835, found 229.0829.

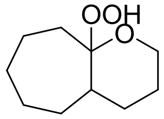

9a-Hydroperoxydecahydrocyclohepta[b]pyran (1q) White solid (0.56 g, 30%, two steps). Melting point (°C): 43-44. R_f = 0.3 (petroleum ether/ethyl acetate = 7:1). ^{1}H NMR (400 MHz, $CDCl_3$) δ 7.45 (br, 0.6H), 3.79 – 3.69 (m, 2H), 2.47 – 2.42 (m, 0.5H), 2.26 – 2.20 (m, 0.5H), 1.88 – 1.57 (m, 8H), 1.50 – 1.27 (m, 6H). ^{13}C NMR (100 MHz, $CDCl_3$) δ 108.5, 106.5, 69.8, 64.8, 61.8, 61.4, 45.0, 38.7, 36.5, 34.0, 31.4, 31.0, 30.8, 29.8, 27.72, 27.65, 26.8, 26.6, 26.4, 25.9, 21.5, 21.1, 20.7. IR (neat): υ_{max} (cm^{-1}) 2897, 2278, 1268, 755. HRMS (ESI) calcd for $C_{10}H_{18}O_3Na$ $[M+Na]^+$ 209.1148, found 209.1161.

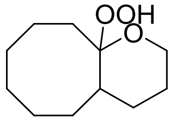

10a-Hydroperoxydecahydro-2H-cycloocta[b]pyran (1r) Colorless oil (0.5 g, 25%, two steps). R_f = 0.3 (petroleum ether/ethyl acetate = 15:1). ^{1}H NMR (400 MHz, $CDCl_3$) δ 7.70 (s, 0.25H), 7.69 (s, 0.7H), 3.84 – 3.68 (m, 2H), 2.05 – 1.88 (m, 3H), 1.82 – 1.38 (m, 13H), 1.27 – 1.08 (m, 1H). ^{13}C NMR (100 MHz, $CDCl_3$) major isomer δ 106.8, 61.4, 38.7, 34.2, 30.8, 29.3, 28.1, 26.6, 26.3, 24.7, 21.4; minor isomer δ 107.7, 61.9, 35.4, 31.5, 29.8, 29.2, 27.6, 26.3, 25.7, 21.5, 20.10. IR (neat): υ_{max} (cm^{-1}) 2931, 2332, 1722, 1421, 1223, 710. HRMS (ESI) calcd for $C_{11}H_{20}O_3Na$ $[M+Na]^+$ 223.1304, found 223.1305.

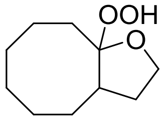

9a-Hydroperoxydecahydrocycloocta[b]furan (1s) Colorless oil (1.1 g, 59%, two steps). R_f = 0.3 (petroleum ether/ethyl acetate = 6:1). ^{1}H NMR (400 MHz, $CDCl_3$) δ 8.66 (br, 0.7H), 3.85 – 3.72 (m, 2H), 2.23 – 2.18 (m, 2H), 1.96 – 1.86 (m, 1H), 1.72 – 1.60 (m, 5H), 1.54 – 1.39 (m, 6H), 1.34 – 1.30 (m, 1H). ^{13}C NMR (100 MHz, $CDCl_3$) major isomer δ 116.8, 65.2, 45.0, 36.3, 31.6, 30.6, 28.4, 25.3 (overlapped), 24.3. minor isomer δ 113.2, 65.8, 45.3, 36.25, 35.3, 33.7, 29.7, 28.1, 25.5, 22.7. IR (neat): υ_{max} (cm^{-1}) 2987, 2321, 1268, 755. HRMS (ESI) calcd for $C_{10}H_{18}O_3Na$ $[M+Na]^+$ 209.1148, found 209.1152.

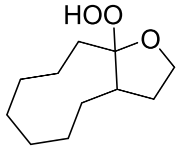

10a-Hydroperoxydecahydro-2H-cyclonona[b]furan (1t) Colorless oil (0.8 g, 40%, two steps). R_f = 0.3 (petroleum ether/ethyl acetate = 15:1). ^{1}H NMR (400 MHz, $CDCl_3$) δ 8.13 (d, J = 11.2 Hz, 0.14H), 8.07 (d, J = 7.2 Hz, 0.86H), 3.99 – 3.75 (m, 2H), 2.48 – 2.26 (m, 2H), 2.15 – 2.02 (m, 1H), 1.91 – 1.68 (m, 5H), 1.61 – 1.44 (m, 9H). ^{13}C NMR (100 MHz, $CDCl_3$) minor isomer δ 117.7, 65.6, 45.0, 35.8, 30.7,

28.1, 27.7, 25.7, 25.4, 25.1, 21.8 (overlapped); major isomer δ 114.0, 66.1, 45.4, 34.7, 33.2, 29.4, 29.2, 29.1, 26.1, 25.9, 21.8 (overlapped). IR (neat): $υ_{max}$ (cm^{-1}) 2922, 2101, 1721, 1422, 1166, 821. HRMS (ESI) calcd for $C_{11}H_{20}O_3$ $[M]^+$ 200.1407, found 200.1399.

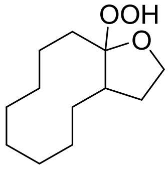

11a-Hydroperoxydodecahydrocyclodeca[b]furan (1u) Colorless oil (0.7 g, 20%, two steps). R_f = 0.3 (petroleum ether/ethyl acetate = 10:1). ^1H NMR (400 MHz, DMSO-d_6) δ 10.80 (s, 0.3H), 10.75 (s, 0.7H), 3.91 – 3.85 (m, 0.7H), 3.78 – 3.65 (m, 1.3H), 2.40 – 2.32 (m, 0.6H), 2.16 – 1.95 (m, 1.5H), 1.89 – 1.70 (m, 3H), 1.65 – 1.24 (m, 14H). ^{13}C NMR (100 MHz, DMSO-d_6) major isomer δ113.2, 65.8, 41.4, 33.12, 30.7, 27.7, 26.3, 24.3, 23.9, 23.6, 23.3, 19.5; minor isomer δ 116.0, 64.9, 42.5, 33.06, 29.2, 28.5, 27.0, 25.8, 25.7, 23.7, 23.5, 21.7. IR (neat): $υ_{max}$ (cm^{-1}) 2922, 2101, 1721, 1422, 1166, 821. HRMS (ESI) calcd for $C_{12}H_{22}O_3Li$ $[M+Li]^+$ 221.1723, found 221.1726.

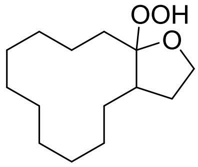

13a-Hydroperoxytetradecahydrocyclododeca[b]furan (1v) White solid (1.25 g, 52%, two steps). Melting point (°C): 92-93. R_f = 0.3 (petroleum ether/ethyl acetate = 8:1). ^1H NMR (400 MHz, $CDCl_3$) δ 8.47 (br, 0.7H), 4.11 (td, J = 8.8, 2.0 Hz, 1H), 3.89 – 3.83 (m, 1H), 2.33 – 2.21 (m, 1H), 2.10 – 2.00 (m, 2H), 1.87 – 1.69 (m, 2H), 1.65 – 1.59 (m, 1H), 1.49 – 1.27 (m, 17H). ^{13}C NMR (100 MHz, $CDCl_3$) δ 115.0, 68.3, 40.0, 30.6, 27.5, 26.2, 26.0, 25.0, 24.2, 22.3, 22.2, 22.0, 21.7, 19.5. IR (neat): $υ_{max}$ (cm^{-1}) 2987, 1268, 755. HRMS (ESI) calcd for $C_{14}H_{26}O_3Na$ $[M+Na]^+$ 265.1774, found 265.1788.

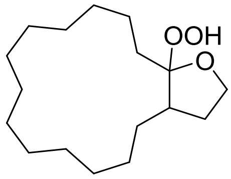

16a-Hydroperoxyhexadecahydro-2*H*-cyclopentadeca[b]furan (1w) Colorless oil (0.28 g, 10%, two steps). R_f = 0.3 (petroleum ether/ethyl acetate = 8:1). ^1H NMR (400 MHz, $CDCl_3$) δ 8.67 (br, 0.6H), 4.10 – 3.78 (m, 2H), 2.16 – 1.99 (m, 2H), 1.90 – 1.69 (m, 2H), 1.45 – 1.27 (m, 25).^{13}C NMR (100 MHz,

$CDCl_3$) δ 117.1, 114.2, 67.6, 66.7, 44.6, 42.7, 33.6, 31.8, 30.7, 29.6, 27.8, 27.7, 27.3, 26.79, 26.75, 26.71, 26.67, 26.6, 26.5, 26.4, 26.33, 26.30, 26.23, 26.15, 26.0, 25.3, 25.1, 22.6, 22.4. IR (neat): υ_{max} (cm^{-1}) 2921, 1752, 1267, 755. HRMS (ESI) calcd for $C_{17}H_{33}O_3$ $[M+H]^+$ 285.2424, found 285.2415.

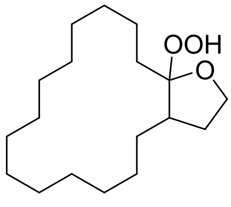

17a-Hydroperoxyoctadecahydrocyclohexadeca[b]furan (1x) Colorless oil (1.2 g, 40%, two steps). R_f = 0.3 (petroleum ether/ethyl acetate = 10:1). ^1H NMR (400 MHz, $CDCl_3$) δ 8.20 (br, 0.3H), 4.11 – 3.82 (m, 2H), 2.21 – 1.97 (m, 2H), 1.90 – 1.75 (m, 2H), 1.68 – 1.61 (m, 1H), 1.43 – 1.21 (m, 26H). ^{13}C NMR (100 MHz, $CDCl_3$) 117.1, 114.3, 67.7, 66.8, 44.2, 42.9, 32.7, 31.2, 30.3, 29.7, 28.5, 27.8, 27.7, 27.5, 27.4, 27.3, 27.2, 27.0, 26.9, 26.8, 26.6, 26.54, 26.47, 26.4, 26.3, 26.11, 26.06, 25.8, 23.3, 22.7. IR (neat): υ_{max} (cm^{-1}) 2924, 1250, 1268, 755. HRMS (ESI) calcd for $C_{18}H_{35}O_3$ $[M+H]^+$ 299.2581, found 299.2573.

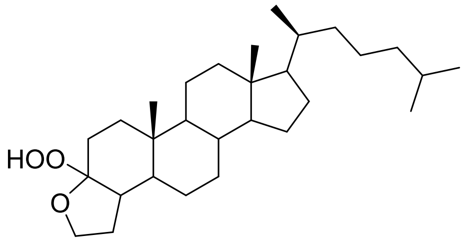

(5aR,7aR)-3a-Hydroperoxy-5a,7a-dimethyl-8-((S)-6-methylheptan-2-yl)octadecahydro-2*H*-cyclopenta[7,8]phenanthro[2,1-b]furan (1y) White solid (2.0 g, 45%, two steps). Melting point (°C): 150-151. R_f = 0.3 (petroleum ether/ethyl acetate = 10:1). ^1H NMR (400 MHz, $CDCl_3$) δ 7.82 (br, 0.7H), 4.17 – 3.94 (m, 2H), 2.35 – 2.26 (m, 1H), 2.18 – 2.04 (m, 2H), 2.01 – 1.94 (m, 1H), 1.85 – 1.72 (m, 2H), 1.67 – 1.60 (m, 2H), 1.57 – 1.44 (m, 4H), 1.35 – 1.19 (m, 9H), 1.16 – 1.05 (m, 6H), 1.03 – 0.94 (m, 3H), 0.90 – 0.85 (m, 11H), 0.80 (s, 3H), 0.64 (s, 3H).^{13}C NMR (100 MHz, $CDCl_3$) δ 113.2, 66.8, 56.5, 56.3, 53.5, 42.6, 42.3, 42.0, 40.1, 39.6, 37.4, 36.2, 36.0, 35.9, 35.4, 33.4, 31.9, 30.8, 28.3, 28.1, 24.3, 23.9, 22.9, 22.7, 21.3, 18.8, 12.1, 11.3. IR (neat): υ_{max} (cm^{-1}) 2926, 2298, 1268, 755. HRMS (ESI) calcd for $C_{29}H_{51}O_3$ $[M+H]^+$ 447.3833, found 447.3813.

11. Characterization of Functionalized Lactones 2-5

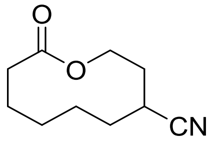

10-Oxooxecane-4-carbonitrile (2a) Colorless oil (34.4 mg, 95%). R_f = 0.3 (petroleum ether/ethyl acetate = 5:1). ^{1}H NMR (400 MHz, $CDCl_3$) δ 4.42 – 4.34 (m, 2H), 2.85 – 2,79 (m, 1H), 2.45 – 2,27 (m, 2H), 2.23 – 2.15 (m, 1H), 2.03 – 1.81 (m, 4H), 1.79 – 1.71 (m, 1H), 1.58 – 1.48 (m, 2H), 1.37 – 1.28 (m, 2H). ^{13}C NMR (100 MHz, $CDCl_3$) δ 173.4, 122.1, 62.4, 34.6, 28.4, 27.2, 26.9, 26.4, 20.7, 20.3. IR (neat): υ_{max} (cm^{-1}) 2932, 2398, 1725,1267, 755. HRMS (ESI) calcd for $C_{10}H_{16}NO_2$ $[M+H]^+$ 182.1175, found 182.1157.

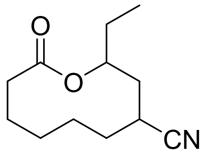

2-Ethyl-10-oxooxecane-4-carbonitrile (2b) Colorless oil (41.0 mg, 98%). R_f = 0.3 (petroleum ether/ethyl acetate = 10:1). d.r. = 3:1, ^{1}H NMR (400 MHz, $CDCl_3$) δ 4.88 – 4.82 (m, 1H), 3.02 – 2.96 (m, 0.22H), 2.57 – 2.44 (m, 1.85H), 2.36 – 2.15 (m, 2H), 2.00 – 1.89 (m, 3H), 1.87 – 1.34 (m, 7H), 1.25 – 1.15 (m, 1H), 0.95 – 0.91 (m, 3H). ^{13}C NMR (100 MHz, $CDCl_3$, two diastereoisomers) major isomer δ 173.8, 123.1, 75.6, 34.79, 32.9, 28.4, 27.24, 27.15, 26.1, 23.0, 20.5, 9.9; minor isomer δ 173.4, 122.1, 74.1, 34.75, 29.8, 26.5, 26.4, 25.23, 25.19, 20.8, 20.3, 10.1. IR (neat): υ_{max} (cm^{-1}) 2927, 2093, 1727, 1265, 755. HRMS (ESI) calcd for $C_{12}H_{23}N_2O_2$ $[M+NH_4]^+$ 227.1754, found 227.1761.

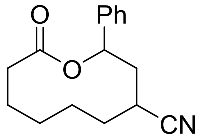

10-Oxo-2-phenyloxecane-4-carbonitrile (2c) Colorless oil (50.6 mg, 98%). R_f = 0.3 (petroleum ether/ethyl acetate = 8:1). d.r. = 1.8:1 ^{1}H NMR (400 MHz, $CDCl_3$) δ 7.40 – 7.23 (m, 5H), 4.58 (dd, *J* = 11.8, 3.4 Hz, 0.51H), 4.14 – 4.12 (m, 0.24H), 4.07 (t, *J* = 11.6 Hz, 0.61H), 3.96 – 3.91 (m, 0.29H), 3.57 (td, *J* = 11.6, 3.2 Hz, 0.53H), 3.14 (td, *J* = 11.6, 3.6 Hz, 0.5H), 3.00 – 2.93 (m, 0.27H), 2.58 (dd, *J* = 4.4, 2.4 Hz, 0.23H), 2.54 (dd, *J* = 4.0, 2.4 Hz, 0.33H), 2.49 – 2.43 (m, 0.29H), 2.37 – 2.28 (m, 1.19H), 2.13 – 1.98 (m, 1H), 1.89 – 1.46 (m, 5.6H), 1.46 – 1.14 (m, 2.68H), 0.94 – 0.86 (m, 0.29H). ^{13}C NMR (100 MHz, $CDCl_3$, two diastereoisomers) major isomer δ 173.2 (overlapped), 137.6, 129.2, 127.9, 127.2,

121.0, 67.1, 56.3, 42.5, 36.6, 34.5, 26.8, 26.5, 20.4. minor isomer δ 173.2 (overlapped), 139.1, 128.7, 128.3, 128.1, 118.0, 72.9, 53.9, 31.9, 31.3, 30.6, 28.0, 23.2, 20.5. IR (neat): υ_{max} (cm^{-1}) 2928, 2201, 1728, 1248, 754, 699. HRMS (ESI) calcd for $C_{16}H_{20}NO_2$ $[M+H]^+$ 258.1489, found 258.1472.

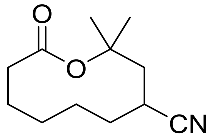

2,2-Dimethyl-10-oxooxecane-4-carbonitrile (2d) Colorless oil (39.8 mg, 95%). R_f = 0.3 (petroleum ether/ethyl acetate = 10:1). ^{1}H NMR (400 MHz, $CDCl_3$) δ 2.97 – 2.91 (m, 1H), 2.69 (dd, *J* = 15.2, 10.0 Hz, 1H), 2.36 (ddd, *J* = 15.2, 8.0, 2.8 Hz, 1H), 2.17 (ddd, *J* = 15.2, 10.8, 2.8 Hz, 1H), 2.00 – 1.84 (m, 3H), 1.78 – 1.71 (m, 1H), 1.70 – 1.56 (m, 3H), 1.65 (s, 3H), 1.54 – 1.44 (m, 1H), 1.41 – 1.30 (m, 1H), 1.38 (s, 3H). ^{13}C NMR (100 MHz, $CDCl_3$) δ 174.0, 122.4, 81.6, 36.3, 34.1, 28.4, 26.8, 26.6, 26.3, 25.6, 21.1, 19.9. IR (neat): υ_{max} (cm^{-1}) 2931, 2203, 1727, 1267, 755. HRMS (ESI) calcd for $C_{12}H_{20}NO_2$ $[M+H]^+$ 210.1488, found 210.1470.

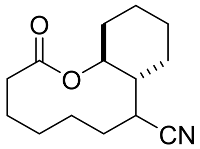

2-Oxododecahydro-2*H*-benzo[b]oxecine-8-carbonitrile (2e) Colorless oil (43.2 mg, 92%). R_f = 0.3 (petroleum ether/ethyl acetate = 10:1). d.r. > 20:1, ^{1}H NMR (400 MHz, $CDCl_3$) δ 4.49 (td, *J* = 10.3, 4.8 Hz, 1H), 2.49 (ddd, *J* = 15.7, 5.9, 3.3 Hz, 1H), 2.33 – 2.18 (m, 3H), 2.10 – 1.96 (m, 2H), 1.93 – 1.79 (m, 3H), 1.78 – 1.70 (m, 3H), 1.66 – 1.53 (m, 2H), 1.38 – 1.27 (m, 3H), 1.26 – 1.17 (m, 2H), 1.15 – 1.03 (m, 1H). ^{13}C NMR (100 MHz, $CDCl_3$) δ 173.6, 122.3, 77.7, 44.5, 36.3, 35.1, 32.6, 31.6, 29.8, 26.0, 25.3, 25.1, 24.4, 20.3. IR (neat): υ_{max} (cm^{-1}) 2931, 2210, 1724, 1249, 755. HRMS (ESI) calcd for $C_{14}H_{22}NO_2$ $[M+H]^+$ 236.1645, found 236.1628.

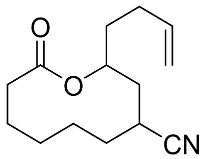

2-(But-3-en-1-yl)-10-oxooxecane-4-carbonitrile (2f) Colorless oil (26.1 mg, 56%). R_f= 0.3 (petroleum ether/ethyl acetate = 15:1). d.r. = 3:1, ^{1}H NMR (400 MHz, $CDCl_3$) δ 5.83 – 5.73 (m, 1H), 5.06 – 4.93 (m, 3H), 2.59 – 2.46 (m, 2H), 2.26 – 2.19 (m, 2H), 2.13 – 2.08 (m, 2H), 2.0 – 1.87 (m, 4H), 1.80 – 1.71 (m, 2H), 1.68 – 1.56 (m, 3H), 1.45 – 1.36 (m, 1H), 1.26 – 1.15 (m, 1H). ^{13}C NMR (100 MHz, $CDCl_3$,

single isomer) δ 173.8, 137.2, 123.0, 115.6, 73.8, 34.9, 33.3, 33.0, 29.7, 28.4, 27.1, 26.2, 23.0, 20.6. IR (neat): υ_{max} (cm^{-1}) 3076, 2237, 1726, 1640, 1455, 1251, 842. HRMS (ESI) calcd for $C_{14}H_{22}NO_2$ $[M+H]^+$ 236.1645, found 236.1651.

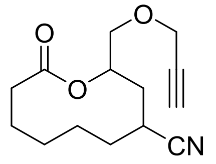

10-Oxo-2-((prop-2-yn-1-yloxy)methyl)oxecane-4-carbonitrile (2g) Colorless oil (30.0 mg, 60%). R_f= 0.3 (petroleum ether/ethyl acetate = 12:1). d.r. = 3:1, ^1H NMR (400 MHz, $CDCl_3$) δ 5.15 – 5.10 (m, 0.24H), 5.05 – 5.00 (m, 0.75H), 4.19 (t, J = 2.4 Hz, 2H), 3.74 – 3.65 (m, 2H), 3.14 – 3.08 (m, 0.23H), 2.56 – 2.45 (m, 2.84H), 2.33 – 2.20 (m, 2H), 2.15 – 1.90 (m, 3H), 1.82 – 1.55 (m, 4H), 1.51 – 1.36 (m, 1H), 1.25 – 1.11 (m, 1H). ^{13}C NMR (100 MHz, $CDCl_3$, two diastereoisomers) major isomer δ 173.8, 122.7, 79.1, 75.2, 72.6, 70.3, 58.6, 34.8 (overlapped), 31.6, 28.6, 27.6, 26.2, 23.3, 20.6; minor isomer δ 173.3, 121.9, 79.0, 75.4, 70.9, 69.8, 58.7, 34.8 (overlapped), 29.4, 26.7, 26.6, 26.1, 21.0, 20.9. IR (neat): υ_{max} (cm^{-1}) 3272, 2958, 2239, 1723, 1455, 1092. HRMS (ESI) calcd for $C_{14}H_{20}NO_3$ $[M+H]^+$ 250.1437, found 250.1433.

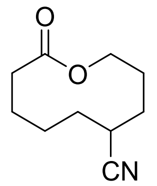

10-Oxooxecane-5-carbonitrile (2h) Colorless oil (29.0 mg, 80%). R_f = 0.3 (petroleum ether/ethyl acetate = 5:1). ^1H NMR (400 MHz, $CDCl_3$) δ 4.75 – 4.70 (m, 1H), 3.89 – 3.83 (m, 1H), 3.11 – 3.04 (m, 1H), 2.59 – 2.53 (m, 1H), 2.23 – 2.16 (m, 1H), 2.13 – 1.89 (m, 3H), 1.83 – 1.75 (m, 2H), 1.69 – 1.56 (m, 4H), 1.54 – 1.46 (m, 1H). ^{13}C NMR (100 MHz, $CDCl_3$) δ 173.0, 123.4, 65.3, 34.5, 29.7, 26.7, 26.5, 25.5, 23.1, 20.2. IR (neat): υ_{max} (cm^{-1}) 2893, 2298, 1726, 1267, 755. HRMS (ESI) calcd for $C_{10}H_{15}NO_2Na$ $[M+Na]^+$ 204.0995, found 204.1011.

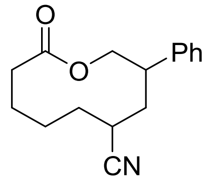

10-Oxo-3-phenyloxecane-5-carbonitrile (2i) Colorless oil (47.2 mg, 92%). R_f = 0.3 (petroleum ether/ethyl acetate = 5:1). d.r. = 1.4:1,^1H NMR (400 MHz, $CDCl_3$) δ 7.35 – 7.31 (m, 2H), 7.29 – 7.22

(m, 2H), 7.20 – (m, 1H), 4.77 – 4.73 (m, 0.2H), 4.52 – 4.39 (m, 0.7H), 4.19 – 4.06 (m, 0.7H), 3.93 (t, *J* = 11.2Hz, 0.2H), 3.81 – 3.73 (m, 0.1H), 3.46 – 3.37 (m, 0.45H), 3.24 – 3.16 (m, 0.64H), 3.01 – 2.96 (m, 0.64H), 2.79 (t, *J* = 13.2Hz, 0.1H), 2.65 – 2.22 (m, 3H), 2.08 – 1.51 (m, 7H). ^{13}C NMR (100 MHz, $CDCl_3$, two diastereoisomers) major isomer δ 173.3, 140.6, 129.1, 127.6, 127.4, 123.3, 68.99, 44.7, 37.6, 34.7, 31.3, 27.6, 24.4, 20.4; minor isomer δ 172.8, 139.6, 129.0, 127.6, 127.5, 123.1, 68.96, 43.7, 38.3, 36.7, 34.4, 26.2, 25.8, 20.1. IR (neat): υ_{max} (cm^{-1}) 2923, 2237, 1734, 1289, 755. HRMS (ESI) calcd for $C_{16}H_{20}NO_2$ $[M+H]^+$ 258.1489, found 258.1470.

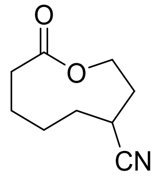

9-Oxooxonane-4-carbonitrile (2j) Colorless oil (29.7 mg, 89%). R_f = 0.3 (petroleum ether/ethyl acetate = 7:1). ^{1}H NMR (400 MHz, $CDCl_3$) δ 4.45 – 4.39 (m, 1H), 4.32 – 4.27 (m, 1H), 2.79 – 2.73(m, 1H), 2.38 – 2.26 (m, 2H), 2.20 – 2.05 (m, 2H), 1.89 – 1.78 (m, 2H), 1.77 – 1.62 (m, 4H). ^{13}C NMR (100 MHz, $CDCl_3$) δ 174.3, 122.3, 61.6, 34.2, 31.3, 30.5, 26.7, 24.4, 22.1. IR (neat): υ_{max} (cm^{-1}) 2931, 2250, 1726, 1261, 755. HRMS (ESI) calcd for $C_9H_{17}N_2O_2$ $[M+NH_4]^+$ 185.1285, found 185.1272.

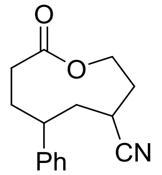

9-Oxo-6-phenyloxonane-4-carbonitrile (2k) Colorless oil (38.8 mg, 80%) R_f = 0.3 (petroleum ether/ethyl acetate = 7:1). d.r. = 1.1:1, ^{1}H NMR (400 MHz, $CDCl_3$) δ 7.35 – 7.30 (m, 2H), 7.26 – 7.20 (m, 2H), 7.16 – 7.13 (m, 1H), 4.87 – 4.81 (m, 0.5H), 4.65 – 4.59 (m, 0.43H), 4.30 – 4.24 (m, 0.44H), 4.20 – 4.15 (m, 0.51H), 3.12 – 3.06 (m, 0.43H), 2.93 – 2.86 (m, 1H), 2.78 – 2.71 (m, 0.52H), 2.50 – 2.40 (m, 2.4H), 2.22 – 1.91 (m, 5.4H). ^{13}C NMR (100 MHz, $CDCl_3$, both diastereoisomers) 174.2, 173.8, 147.1, 146.1, 129.0, 128.9, 127.2, 127.04, 127.69, 126.8, 122.4, 121.5, 61.6, 61.4, 40.8, 40.7, 40.1, 38.5, 34.4, 32.9, 32.0, 31.3, 29.6, 26.1, 25.4. IR (neat): υ_{max} (cm^{-1}) 2936, 2231, 1732, 1453, 1249, 755, 701. HRMS (ESI) calcd for $C_{15}H_{18}NO_2$ $[M+H]^+$ 244.1332, found 244.1322.

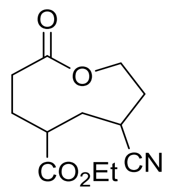

Ethyl 7-cyano-2-oxooxonane-5-carboxylate (2l) Colorless oil (35.9 mg, 75%). R_f = 0.3 (petroleum ether/ethyl acetate = 5:1). d.r. = 1.5:1 ^{1}H NMR (400 MHz, $CDCl_3$) δ 4.77 – 3.96 (m, 4H), 2.95 – 2.79 (m, 1H), 2.62 – 2.04 (m, 6H), 1.93– 1.48 (m, 3H), 1.30 – 1.25 (m, 3H). ^{13}C NMR (100 MHz, $CDCl_3$, both diastereoisomers) δ 175.3, 174.7, 174.3, 173.5, 173.1, 122.0, 121.2, 111.1, 111.0, 66.7, 61.53, 61.49, 61.4, 61.3, 60.5, 41.7, 41.4, 40.4, 40.24, 40.19, 40.1, 33.8, 33.6, 33.3, 32.6, 32.3, 32.0, 31.0, 30.6, 29.8, 29.7, 29.6, 27.9, 27.3, 27.0, 25.53, 25.49, 25.3, 14.3, 14.2. IR (neat): υ_{max} (cm^{-1}) 2935, 2309, 1730, 1453, 1267, 755. HRMS (ESI) calcd for $C_{12}H_{18}NO_4$ $[M+H]^+$ 240.1230, found 240.1219.

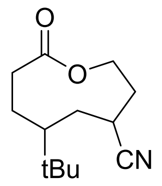

6-(*tert*-butyl)-9-Oxooxonane-4-carbonitrile (2m) Colorless oil (28.1 mg, 63%). R_f = 0.3 (petroleum ether/ethyl acetate = 10:1). d.r. = 1:1, ^{1}H NMR (400 MHz, $CDCl_3$) δ 4.67 – 4.61 (m, 0.5H), 4.53 – 4.57 (m, 0.5H), 4.29 – 4.23 (m, 0.5H), 4.16 – 4.10 (m, 0.5H), 2.85 – 2.79 (m, 0.5H), 2.59 – 2.53 (m, 0.5H), 2.50 – 2.43 (m, 1H), 2.29 – 1.83 (m, 5H), 1.50 – 1.25 (m, 3H), 0.91 (s, 4.5H), 0.89 (s, 4.5H). ^{13}C NMR (100 MHz, $CDCl_3$, both diastereoisomers) δ 175.0, 173.5, 122.9, 122.8, 61.9, 61.7, 47.8, 43.6, 37.1, 36.4, 35.9, 34.6, 34.5, 31.9, 31.0, 30.3, 28.8, 27.7, 27.3, 27.1, 24.9. IR (neat): υ_{max} (cm^{-1}) 2954, 2260, 1738, 1460, 1267, 755. HRMS (ESI) calcd for $C_{13}H_{21}NO_2Li$ $[M+Li]^+$ 230.1727, found 230.1736.

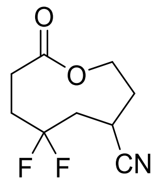

6,6-Difluoro-9-oxooxonane-4-carbonitrile (2n) Colorless oil (30.3 mg, 75%). R_f = 0.3 (petroleum ether/ethyl acetate = 5:1). ^{1}H NMR (400 MHz, $CDCl_3$) δ 4.72 – 4.66 (m, 1H), 4.20 (dt, *J* = 11.2, 8.0 Hz, 1H), 2.83 – 2.77 (m, 1H), 2.65 – 2.42 (m, 4H), 2.38 – 2.29 (m, 1H), 2.25 – 2.19 (m, 2H), 2.09 (ddt, *J* = 34.0, 16.0, 3.6 Hz, 1H). ^{13}C NMR (100 MHz, $CDCl_3$) δ 172.0, 123.9 (t, *J* = 241 Hz), 121.4, 60.1, 39.6 (dd, *J* = 27.0, 4.0 Hz), 32.42 (t, *J* = 28.0 Hz), 31.1, 30.0 (d, *J* = 40.0 Hz), 20.9 (d, *J* = 40.0 Hz). IR (neat):

υ_{max} (cm^{-1}) 2931, 2367, 1765, 1267, 755. HRMS (ESI) calcd for $C_9H_{12}F_2NO_2$ $[M+H]^+$ 204.0831, found 204.0823.

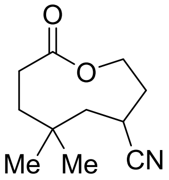

6,6-Dimethyl-9-oxooxonane-4-carbonitrile (2o) Colorless oil (36.0 mg, 92%). R_f = 0.3 (petroleum ether/ethyl acetate = 5:1). ^1H NMR (400 MHz, $CDCl_3$) δ 4.75 – 4.69 (m, 1H), 4.07 – 4.00 (m, 1H), 2.65 – 2.60 (m, 1H), 2.44 (ddd, *J* = 15.2, 5.2, 3.6 Hz, 1H), 2.34 – 2.30 (m, 1H), 2.18 – 2.10 (m, 2H), 2.09 – 1.99 (m, 1H), 1.71 (ddd, *J* = 16.8, 7.2, 0.8 Hz, 1H), 1.53 (d, *J* = 16.0 Hz, 1H), 1.41 – 11.35 (m, 1H), 1.04 (s, 3H), 0.93 (s, 3H). ^{13}C NMR (100 MHz, $CDCl_3$) δ 175.0, 124.0, 59.4, 43.4, 35.2, 34.8, 31.4, 31.1, 28.8, 28.3, 21.0. IR (neat): υ_{max} (cm^{-1}) 2897, 2321, 1732, 1267, 754. HRMS (ESI) calcd for $C_{11}H_{21}N_2O_2$ $[M+NH_4]^+$ 213.1598, found 213.1593.

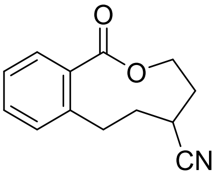

1-Oxo-1,3,4,5,6,7-Hexahydrobenzo[c]oxonine-5-carbonitrile (2p) Colorless oil (35.6 mg, 83%). R_f = 0.3 (petroleum ether/ethyl acetate = 5:1). ^1H NMR (400 MHz, $CDCl_3$) δ 7.80 (dd, *J* = 7.6, 1.6 Hz, 1H), 7.41 (td, *J* = 7.6, 1.6 Hz, 1H), 7.32 (td, *J* = 7.6, 1.2 Hz, 1H), 7.22 (dd, *J* = 7.6, 0.8 Hz, 1H), 4.79 – 4.74 (m, 1H), 4.56 – 4.50 (m, 1H), 3.40 (dt, *J* = 14.8, 5.6 Hz, 1H), 3.04 – 2.91 (m, 2H), 2.28 – 2.20 (m, 3H), 1.79 – 1.75 (m, 1H). ^{13}C NMR (100 MHz, $CDCl_3$) δ 169.2, 143.7, 132.3, 131.1, 130.8, 130.5, 127.1, 121.1, 63.3, 33.3, 32.4, 30.7, 29.0. IR (neat): υ_{max} (cm^{-1}) 2789, 2319, 1729, 1265, 749. HRMS (ESI) calcd for $C_{13}H_{13}NO_2Li$ $[M+Li]^+$ 222.1101, found 222.1087.

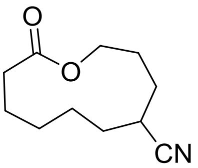

11-Oxooxacycloundecane-5-carbonitrile (2q) Colorless oil (31.2 mg, 80%). R_f = 0.3 (petroleum ether/ethyl acetate = 5:1). ^1H NMR (400 MHz, $CDCl_3$) δ 4.24 – 4.15 (m, 2H), 2.68 – 2.60 (m, 1H), 2.45 – 2.39 (m, 1H), 2.32 – 2.25 (m, 1H), 1.99– 1.84 (m, 4H), 1.81 – 1.53 (m, 6H), 1.45 – 1.27 (m, 2H). ^{13}C

NMR (100 MHz, $CDCl_3$) δ 173.7, 122.8, 64.0, 35.1, 28.3, 27.4, 26.1, 25.8, 24.0, 22.9, 21.0. IR (neat): υ_{max} (cm^{-1}) 2920, 2250, 1768, 1267, 755. HRMS (ESI) calcd for $C_{11}H_{17}NO_2K$ $[M+K]^+$ 234.0891, found 234.0906.

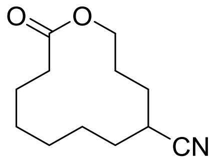

12-Oxooxacyclododecane-5-carbonitrile (2r) Colorless oil (18 mg, 43%). R_f = 0.3 (petroleum ether/ethyl acetate = 10:1). ^1H NMR (400 MHz, $CDCl_3$) δ 4.55 – 4.50 (m, 1H), 3.98 – 3.92 (m, 1H), 2.92 – 2.85 (m, 1H), 2.45 – 2.33 (m, 2H), 1.99 – 1.90 (m, 1H), 1.88 – 1.57 (m, 8H), 1.54 – 1.47 (m, 1H), 1.42 – 1.32 (m, 3H), 1.30 – 1.24 (m, 1H). ^{13}C NMR (100 MHz, $CDCl_3$) δ 173.4, 122.9, 64.2, 33.7, 28.6, 27.2, 26.6, 24.7, 24.2, 23.5, 23.3, 22.6. IR (neat): υ_{max} (cm^{-1}) 2936, 2237, 1730, 1456, 1239, 784. HRMS (ESI) calcd for $C_{12}H_{19}NO_2Na$ $[M+Na]^+$ 232.1308, found 232.1305.

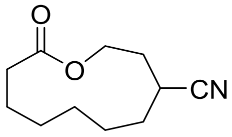

11-Oxooxacycloundecane-4-carbonitrile (2s) Colorless oil (35.5 mg, 91%). R_f = 0.3 (petroleum ether/ethyl acetate = 5:1). ^1H NMR (400 MHz, $CDCl_3$) δ 4.31 – 4.17 (m, 2H), 2.80 – 2.74 (m, 1H), 2.42 – 2.26 (m, 2H), 2.23 – 2.15 (m, 1H), 2.13 – 2.04 (m, 1H), 1.79 – 1.57 (m, 4H), 1.52 (m, 2H), 1.46 – 1.32 (m, 4H). ^{13}C NMR (100 MHz, $CDCl_3$) δ 173.5, 122.6, 61.7, 35.1, 28.5, 27.9, 27.5, 24.94, 24.85, 21.5, 20.6. IR (neat): υ_{max} (cm^{-1}) 2942, 2245, 1768, 1267, 755. HRMS (ESI) calcd for $C_{11}H_{21}N_2O_2$ $[M+NH_4]^+$ 213.1598, found 213.1590.

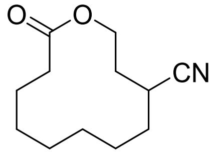

12-Oxooxacyclododecane-4-carbonitrile (2t) Colorless oil (25.0 mg, 60%). R_f = 0.3 (petroleum ether/ethyl acetate = 10:1). ^1H NMR (400 MHz, $CDCl_3$) δ 4.60 – 4.54 (m, 1H), 4.10 – 4.04 (m, 1H), 2.88 – 2.81 (m, 1H), 2.50 – 2.44 (m, 1H), 2.34 – 2.27 (m, 1H), 2.07 – 1.93 (m, 2H), 1.85 – 1.71 (m, 3H), 1.66 – 1.60 (m, 1H), 1.57 – 1.41 (m, 2H), 1.41 – 1.28 (m, 6H). ^{13}C NMR (100 MHz, $CDCl_3$) δ 173.4, 122.5, 61.2, 34.1, 29.4, 28.4, 26.6, 24.7, 24.0, 23.7, 23.4, 21.8. IR (neat): υ_{max} (cm^{-1}) 2935, 2239, 1731, 1455, 1234, 750. HRMS (ESI) calcd for HRMS (ESI) calcd for $C_{12}H_{19}NO_2Na$ $[M+Na]^+$ 232.1308, found

232.1305.

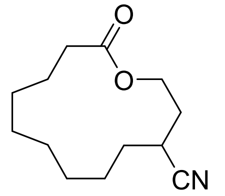

13-oxooxacyclotridecane-4-carbonitrile (2u) Colorless oil (18 mg, 40%). R_f = 0.3 (petroleum ether/ethyl acetate = 10:1). ^{1}H NMR (400 MHz, $CDCl_3$). δ 4.33 – 4.20 (m, 2H), 2.74 – 2.67 (m, 1H), 2.46 – 2.40 (m, 1H), 2.35 – 2.28 (m, 1H), 2.12 – 1.91 (m, 2H), 1.76 – 1.64 (m, 4H), 1.60 – 1.50 (m, 1H), 1.48 – 1.29 (m, 9H). ^{13}C NMR (100 MHz, $CDCl_3$) δ 173.7, 122.4, 61.7, 34.5, 31.1, 30.2, 26.8, 26.7, 26.4, 25.2, 24.9, 24.4, 23.7. IR (neat): υ_{max} (cm^{-1}) 2921, 2224, 1733, 1466, 1221, 765. HRMS (ESI) calcd for $C_{13}H_{21}NO_2Na$ [M+Na]$^+$ 246.1464, found 246.1460.

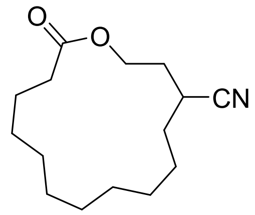

15-Oxooxacyclopentadecane-4-carbonitrile (2v) Colorless oil (47.7 mg, 95%). R_f = 0.3 (petroleum ether/ethyl acetate = 10:1). ^{1}H NMR (400 MHz, $CDCl_3$). δ 4.33 – 4.28 (m, 1H), 4.18 – 4.12 (m, 1H), 2.73 (q, J = 7.2 Hz, 1H), 2.41 – 2.29 (m, 2H), 2.08 – 1.99 (m, 1H), 1.96 – 1.88 (m, 1H), 1.69 – 1.60 (m, 5H), 1.598– 1.51 (m, 1H), 1.41 – 1.22 (m, 12H). ^{13}C NMR (100 MHz, $CDCl_3$) δ 173.9, 121.9, 61.1, 33.8, 31.7, 29.4, 27.9, 26.9, 26.60, 26.57, 26.5, 25.5, 25.1, 24.8, 24.7. IR (neat): υ_{max} (cm^{-1}) 2857, 2230, 1733, 1165, 755. HRMS (ESI) calcd for $C_{15}H_{25}NO_2Na$ [M+Na]$^+$ 274.1777, found 274.1756.

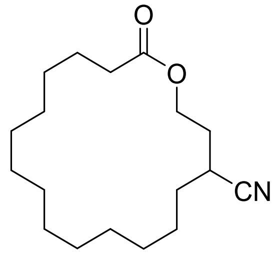

18-Oxooxacyclooctadecane-4-carbonitrile (2w) Colorless oil (49.1 mg, 84%). R_f = 0.3 (petroleum ether/ethyl acetate = 20:1). ^{1}H NMR (400 MHz, $CDCl_3$). δ 4.37 – 4.32 (m, 1H), 4.18 – 4.12 (m, 1H), 2.78 – 2.70 (m, 1H), 2.32 (t, J = 7.2 Hz, 2H), 1.95 – 1.90 (m, 2H), 1.72 – 1.47 (m, 6H), 1.38 – 1.24 (m, 18H). ^{13}C NMR (100 MHz, $CDCl_3$) δ 173.4, 121.6, 61.0, 34.6, 31.5, 31.2, 28.3, 28.0, 27.7, 27.2, 27.0,

26.92, 26.91, 26.7, 26.6, 26.1, 25.8, 24.8. IR (neat): υ_{max} (cm^{-1}) 2925, 2260, 1734, 1453, 1265, 755. HRMS (ESI) calcd for $C_{18}H_{32}NO_2$ $[M+H]^+$ 294.2428, found 294.2411.

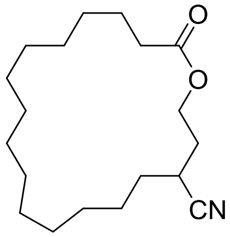

19-Oxooxacyclononadecane-4-carbonitrile (2x) Colorless oil (46.0 mg, 75%). R_f = 0.3 (petroleum ether/ethyl acetate = 20:1). ^1H NMR (400 MHz, $CDCl_3$). 4.35 – 4.30 (m, 1H), 4.20 – 4.14 (m, 1H), 2.73 – 2.66 (m, 1H), 2.33 (t, J = 7.2 Hz, 2H), 1.97 – 1.92 (m, 2H), 1.70 – 1.48 (m, 7H), 1.35 – 1.27 (m, 19H). ^{13}C NMR (100 MHz, $CDCl_3$) δ 173.6, 121.7, 61.2, 34.5, 31.6, 28.5, 28.2, 28.0, 27.7, 27.6, 27.5, 27.34, 27.29, 26.9, 26.5, 24.8. IR (neat): υ_{max} (cm^{-1}) 2924, 2210, 1734, 1451, 1266, 1159, 755. HRMS (ESI) calcd for $C_{19}H_{34}NO_2$ $[M+H]^+$ 308.2584, found 308.2581.

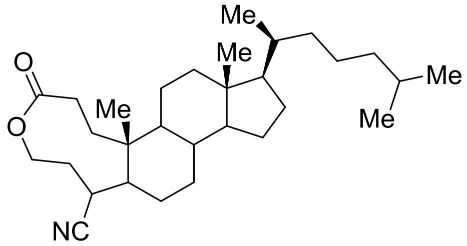

(7aR,9aR,10R)-7a,9a-Dimethyl-10-((S)-6-methylheptan-2-yl)-5-oxooctadecahydro-1*H* cyclopenta [5,6] naphtho[1,2-e]oxonine-1-carbonitrile (2y) Colorless oil (59.1 mg, 65%). R_f = 0.3 (petroleum ether/ethyl acetate = 10:1). ^1H NMR (400 MHz, $CDCl_3$). δ 4.85 – 4.79 (m, 1H), 3.96 – 3.89 (m, 1H), 2.84 – 2.79 (m, 1H), 2.31 – 1.97 (m, 8H), 1.86 – 1.76 (m, 2H), 1.71 – 1.65 (m, 1H), 1.57 – 1.39 (m, 4H), 1.36 – 1.28 (m, 4H), 1.25 – 1.23 (m, 2H), 1.14 – 0.93 (m, 10H), 0.88 (d, J = 6.8 Hz, 3H), 0.85 (dd, J = 4.4, 2.0 Hz, 6H), 0.81 (s, 3H), 0.65 (s, 3H). ^{13}C NMR (100 MHz, $CDCl_3$) δ 174.7, 123.2, 59.1, 56.7, 56.0, 47.9, 42.5, 41.3, 40.8, 39.9, 39.60, 39.55, 39.2, 36.2, 35.9, 35.5, 31.8, 31.1, 29.5, 28.4, 28.1, 24.2, 23.8, 22.9, 22.7, 21.8, 19.2, 18.7, 15.6, 12.3. IR (neat): υ_{max} (cm^{-1}) 2936, 2198, 1737, 1459, 1268, 754. HRMS (ESI) calcd for $C_{30}H_{49}NO_2Na$ $[M+Na]^+$ 478.3656, found 478.3659.

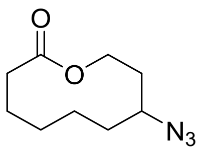

8-Azidooxecan-2-one (3a) Colorless oil (32.3 mg, 82%). R_f = 0.3 (petroleum ether/ethyl acetate = 30:1). ^{1}H NMR (400 MHz, $CDCl_3$). δ 4.36 – 4.28 (m, 2H), 3.74 – 3.69 (m, 1H), 2.44 – 2.37 (ddd, *J* = 16.4, 8.0, 2.8 Hz, 1H), 2.29 (ddd, *J* = 16.4, 10.0, 2.8 Hz, 1H), 2.13 – 2.05 (m, 1H), 1.94 – 1.70 (m, 5H), 1.59 – 1.45 (m, 2H), 1.40 – 1.31 (m, 1H), 1.24 – 1.13 (m, 1H). ^{13}C NMR (100 MHz, $CDCl_3$) δ 173.5, 61.2, 60.1, 34.7, 29.0, 28.4, 27.2, 20.4, 20.0. IR (neat): υ_{max} (cm^{-1}) 2930, 2092, 1726, 1454, 1262, 1060, 755. HRMS (ESI) calcd for $C_9H_{15}N_3O_2Li$ $[M+Li]^+$ 204.1319, found 204.1335.

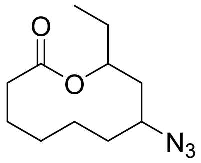

8-Azido-10-ethyloxecan-2-one (3b) Colorless oil (41.0 mg, 91%). R_f = 0.3 (petroleum ether/ethyl acetate = 30:1). d.r. = 3:1 ^{1}H NMR (400 MHz, $CDCl_3$). δ 4.87 –4.79 (m, 1H), 3.88 – 3.82 (m, 0.2H), 3.46 – 3.41 (m, 0.8H), 2.51 – 2.43 (m, 1H), 2.24 – 2.12 (m, 1H), 2.06 – 1.93 (m, 2H), 1.91 – 1.79 (m, 2H), 1.77 – 1.62 (m, 3H), 1.61 – 1.52 (m, 3H), 1.46 – 1.37 (m, 1H), 1.17 – 1.07 (m, 1H), 0.95 – 0.89 (m, 3H). ^{13}C NMR (100 MHz, $CDCl_3$, two diastereoisomers) major isomer δ 173.1, 73.7, 59.4, 34.6, 33.9, 29.3, 26.7, 25.6, 21.1, 19.6, 8.9; minor isomer δ 172.7, 72.6, 56.7, 33.8, 30.5, 27.4, 25.9, 24.6, 19.9, 17.9, 9.3. IR (neat): υ_{max} (cm^{-1}) 2934, 2093, 1723, 1456, 1249, 737. HRMS (ESI) calcd for $C_{11}H_{20}N_3O_2$ $[M+H]^+$ 226.1550, found 226.1568.

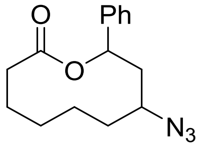

8-Azido-10-phenyloxecan-2-one (3c) Colorless oil (44.9 mg, 82%). R_f = 0.3 (petroleum ether/ethyl acetate = 30:1). d.r. = 4:1 ^{1}H NMR (400 MHz, $CDCl_3$). δ 7.39 – 7.34 (m, 2H), 7.33 – 7.27 (m, 2H), 7.26 – 7.21 (m, 1H), 4.68 (dd, *J* = 8.4, 2.8 Hz, 0.2H), 4.52 (dd, *J* = 8.4, 3.6 Hz, 0.8H), 4.67 (d, *J* = 11.2 Hz, 0.2H), 4.00 (d, *J* = 12 Hz, 0.6H), 3,98 – 3.93 (m, 1H), 3.75 – 3.70 (m, 0.2H), 3.56 (dt, *J* = 11.2, 2.8 Hz, 0.2H), 3.40 (td, *J* = 11.6, 3.6 Hz, 0.8H), 2.61 – 2.52 (m, 1H), 2.32 – 2.02 (m, 3H), 1.95 – 1.75 (m, 1H), 1.73 – 1.53 (m, 4H), 1.40 – 1.24 (m, 1H). ^{13}C NMR (100 MHz, $CDCl_3$, two diastereoisomers) major isomer δ 173.3, 138.5, 129.0, 128.1, 127.8, 65.9, 65.8, 44.7, 34.5, 28.7, 27.2, 20.4, 19.1; minor isomer δ

173.5, 138.9, 128.6, 128.4, 127.4, 63.3, 63.0, 42.1, 34.6, 29.3, 27.3, 20.7, 20.2. IR (neat): υ_{max} (cm^{-1}) 2893, 2098, 1728, 1262, 733. HRMS (ESI) calcd for $C_{15}H_{19}N_3O_2Li$ $[M+Li]^+$ 280.1631, found 280.1625.

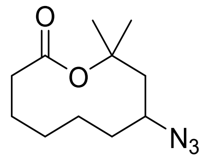

8-Azido-10,10-dimethyloxecan-2-one (3d) Colorless oil (33.8 mg, 75%). R_f = 0.3 (petroleum ether/ethyl acetate = 30:1). ^{1}H NMR (400 MHz, $CDCl_3$) δ 3.86 –3.80 (m, 1H), 2.55 (dd, *J* = 15.2, 10.0 Hz, 1H), 2.37 (ddd, *J* = 15.2, 7.2, 2.8 Hz, 1H), 2.17 – 2.10 (m, 1H), 1.96 – 1.84 (m, 2H), 1.78 – 1.71 (m, 2H), 1.67 – 1.58 (m, 5H), 1.53 – 1.41 (m, 2H), 1.37 (s, 3H), 1.32 – 1.28 (m, 1H). ^{13}C NMR (100 MHz, $CDCl_3$) δ 174.1, 81.3, 58.9, 36.4, 36.2, 28.8, 27.6, 27.2, 27.1, 21.1, 18.7. IR (neat): υ_{max} (cm^{-1}) 2897, 2099, 1725, 1262, 733. HRMS (ESI) calcd for $C_{11}H_{20}N_3O_2$ $[M+H]^+$ 226.1550, found 226.1561.

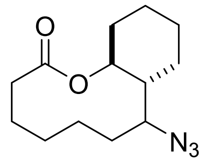

8-Azidododecahydro-2*H*-benzo[b]oxecin-2-one (3e) Colorless oil (46.8 mg, 93%). R_f= 0.3 (petroleum ether/ethyl acetate = 30:1). d.r. > 20:1,^{1}H NMR (400 MHz, $CDCl_3$) δ 4.51 (td, *J* = 10.4, 4.4 Hz, 1H), 2.96 – 2.92 (m, 1H), 2.48 (ddd, *J* = 15.6, 6.4, 3.2 Hz, 1H), 2.27 – (m, 2H), 2.09 – 1.92 (m, 3H), 1.78 – 1.67 (m, 4H), 1.66 – 1.58 (m, 3H), 1.46 – 1.38 (m, 1H), 1.35 – 1.27 (m, 2H), 1.23 – 1.113 (m, 2H), 1.02 –0.92 (m, 1H). ^{13}C NMR (100 MHz, $CDCl_3$) δ 174.0, 75.9, 67.4, 46.7, 35.1, 32.7, 31.3, 30.3, 26.7, 25.1, 24.6, 23.7, 20.6. IR (neat): υ_{max} (cm^{-1}) 2934, 2095, 1723, 1258, 734. HRMS (ESI) calcd for $C_{13}H_{21}N_3O_2K$ $[M+K]^+$ 290.1265, found 290.1254.

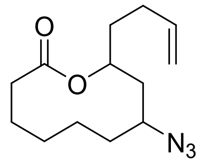

8-Azido-10-(but-3-en-1-yl)oxecan-2-one (3f) Colorless oil (31.0 mg, 62%). R_f = 0.3 (petroleum ether/ethyl acetate = 30:1). d.r. = 3.3:1, ^{1}H NMR (400 MHz, $CDCl_3$) δ 5.84 – 5.74 (m, 1H), 5.05 – 4.90 (m, 3H), 3.89 – 3.84 (m, 0.22H), 3.50 – 3.45 (m, 0.75H), 2.51 – 2.43 (m, 1H), 2.25 – 1.96 (m, 5H), 1.91 – 1.78 (m, 3H), 1.74 – 1.54 (m, 5H), 1.46 – 1.40 (m, 1H), 1.17 – 1.10 (m, 1H). ^{13}C NMR (100 MHz, $CDCl_3$, two diastereoisomers) major isomer δ 174.1, 137.6, 115.3, 73.1, 60.4, 35.6, 35.0, 33.8, 30.3, 29.9, 26.7, 22.1, 20.8; minor isomer δ 173.7, 137.3, 115.6, 71.8, 57.9, 34.9, 31.9, 31.7, 30.1, 28.6, 27.0, 21.0,

19.0. IR (neat): υ_{max} (cm^{-1}) 3074, 2095, 1727, 1642, 1455, 1249, 913. HRMS (ESI) calcd for $C_{13}H_{22}N_3O_2$ $[M+H]^+$ 252.1706, found 252.1693.

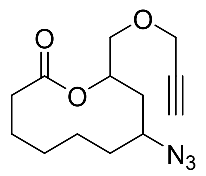

8-Azido-10-((prop-2-yn-1-yloxy)methyl)oxecan-2-one (3g) Colorless oil (31.6 mg, 60%). R_f = 0.3 (petroleum ether/ethyl acetate = 30:1). d.r. = 3:1, ^{1}H NMR (400 MHz, $CDCl_3$) δ 5.13 – 5.08 (m, 0.24H), 5.05 – 5.00 (m, 0.76H), 4.19 (t, *J* = 2.0 Hz, 2H), 3.94 – 3.88 (m, 0.22H), 3.77 – 3.65 (m, 2H), 3.50 – 3.44 (m, 0.76H), 2.56 – 2.48 (m, 1H), 2.47 – 2.44 (m, 1H), 2.27 – 2.09 (m, 2H), 2.07 – 1.83 (m, 3H), 1.66 – 1.53 (m, 4H), 1.50 – 1.41 (m, 1H), 1.15 – 1.03 (m, 1H). ^{13}C NMR (100 MHz, $CDCl_3$, two diastereoisomers) major isomer δ 174.0, 79.3, 75.1, 72.0, 70.9, 60.3, 58.6, 34.9, 33.2, 30.3, 26.8, 22.1, 20.6; minor isomer δ 173.7, 79.2, 75.2, 70.4, 70.2, 58.6, 58.2, 34.8, 30.7, 28.7, 27.0, 21.2, 19.5. IR (neat): υ_{max} (cm^{-1}) 3289, 2937, 2093, 1724, 1453, 1240, 1093, 637. HRMS (ESI) calcd for $C_{13}H_{19}N_3O_3Na$ $[M+Na]^+$ 288.1318, found 288.1317.

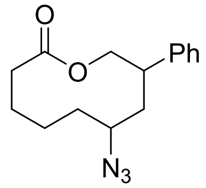

7-Azido-9-phenyloxecan-2-one (3h) Colorless oil (34.3 mg, 63%). R_f = 0.3 (petroleum ether/ethyl acetate = 20:1). d.r. = 2.5:1. ^{1}H NMR (400 MHz, $CDCl_3$) δ 7.36 – 7.31 (m, 2H), 7.29 – 7.26 (m, 1H), 7.24 – 7.20 (m, 2H), 4.74 (ddd, *J* = 11.2, 3.6, 1.6 Hz, 0.3H), 4.48 (t, *J* = 11.6 Hz, 0.8H), 4.07 (dd, *J* = 11.2, 4.4 Hz, 1H), 3.92 (t, *J* = 11.6 Hz, 0.3H), 3.78 – 3.73 (m, 0.74H), 3.36 (tt, *J* = 12, 4.0 Hz, 0.3H), 3.28 – 3.20 (m, 0.75H), 2.64 – 2.56 (m, 1H), 2.34 – 2.24 (m, 1.4H), 2.17 – 2.04 (m, 0.4H), 2.03 – 2.0 (m, 1H), 1.97 – 1.65 (m, 4H), 1.59 – 1.42 (m, 2H). ^{13}C NMR (100 MHz, $CDCl_3$, two diastereoisomers) major isomer δ173.5, 141.3, 128.92, 127.6 (overlapped), 127.4, 68.93, 60.5, 44.2, 40.3, 34.8, 33.0, 23.9, 20.9; minor isomer δ 173.1, 140.3, 128.86, 127.6 (overlapped), 127.3, 68.87, 58.0, 39.3, 39.1, 34.5, 28.0, 25.2, 20.7. IR (neat): υ_{max} (cm^{-1}) 2944, 2090, 1729, 1243, 807, 697. HRMS (ESI) calcd for $C_{15}H_{19}N_3O_2Li$ $[M+Li]^+$ 280.1632, found 280.1618.

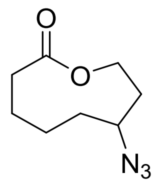

7-Azidooxonan-2-one (3i) Colorless oil (22.6 mg, 62%). R_f = 0.3 (petroleum ether/ethyl acetate = 30:1). ^{1}H NMR (400 MHz, $CDCl_3$). δ 4.37 – 4.28 (m, 2H), 3.64 – 3.59 (m, 1H), 2.35 – 2.25 (m, 2H), 2.07 – 1.93 (m, 2H), 1.80 – 1.59 (m, 6H). ^{13}C NMR (100 MHz, $CDCl_3$) δ 174.7, 61.5, 59.4, 34.2, 32.8, 32.4, 24.7, 20.8. IR (neat): υ_{max} (cm^{-1}) 2943, 2091, 1728, 1254, 754. HRMS (ESI) calcd for $C_8H_{13}N_3O_2Na$ $[M+Na]^+$ 206.0900, found 206.0909.

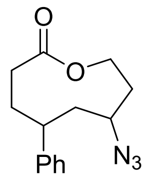

7-Azido-5-phenyloxonan-2-one (3j) Colorless oil (31.0 mg, 60%). R_f = 0.3 (petroleum ether/ethyl acetate = 30:1). d.r. = 1:1, ^{1}H NMR (400 MHz, $CDCl_3$). δ 7.34 – 7.30 (m, 2H), 7.25 – 7.20 (m, 2H), 7.19 – 7.16 (m, 1H), 4.87 – 4.81 (m, 0.5H), 4.63 (td, J = 12.0, 2.4 Hz, 0.5H), 4.18 (dt, J = 11.6, 3.6 Hz, 0.6H), 4.06 – 4.01 (m, 0.5H), 3.78 – 3.71 (m, 0.5H), 3.67 – 3.61 (m, 0.5H), 3.08 – 3.02 (m, 0.5H), 2.95 – 2.90 (m, 0.5H), 2.50 – 2.35 (m, 2.5H), 2.24 – 1.89 (m, 5H), 1.84 – 1.78 (m, 0.5H). ^{13}C NMR (100 MHz, $CDCl_3$, both diastereoisomers) δ 174.6, 174.1, 148.2, 147.2, 128.2, 128.8, 127.2, 127.1, 126.7, 126.4, 62.0, 61.0, 58.7, 57.8, 41.3, 40.7, 40.3, 39.1, 34.4, 33.4, 33.0, 32.8, 32.1. IR (neat): υ_{max} (cm^{-1}) 2936, 2078, 1768, 1265, 760, 701. HRMS (ESI) calcd for $C_{14}H_{21}N_4O_2$ $[M+NH_4]^+$ 277.1659, found 277.1656.

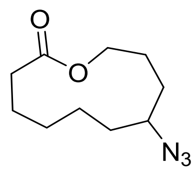

8-Azidooxacycloundecan-2-one (3k) Colorless oil (29.1 mg, 69%). R_f = 0.3 (petroleum ether/ethyl acetate = 40:1). ^{1}H NMR (400 MHz, $CDCl_3$) δ 4.25 – 4.14 (m, 2H), 3.52 – 3.46 (m, 1H), 2.46 – 2.40 (m, 1H), 2.30 – 2.25 (m, 1H), 1.94 – 1.61 (m, 8H), 1.49 – 1.32 (m, 4H). ^{13}C NMR (100 MHz, $CDCl_3$) δ 173.9, 64.4, 60.1, 35.2, 29.4, 27.6, 26.5, 23.1, 22.1, 21.0. IR (neat): υ_{max} (cm^{-1}) 2894, 2489, 2289, 1754, 1267, 755. HRMS (ESI) calcd for $C_{10}H_{17}N_3O_2Na$ $[M+Na]^+$ 234.1213, found 234.1196.

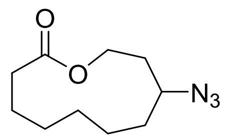

9-Azidooxacycloundecan-2-one (3l) Colorless oil (29.5 mg, 70%). R_f = 0.3 (petroleum ether/ethyl acetate = 30:1). ^1H NMR (400 MHz, $CDCl_3$) δ 4.26 – 4.15 (m, 2H), 3.69 – 3.63 (m, 1H), 2.39 – 2.27 (m, 2H), 2.09 – 2.02 (m, 1H), 1.99 – 1.91 (m, 1H), 1.75 – 1.65 (m, 1H), 1.64 – 1.56 (m, 3H), 1.51 – 1.44 (m, 3H), 1.41 – 1.33 (m, 3H). ^{13}C NMR (100 MHz, $CDCl_3$) δ 173.5, 61.0, 60.0, 35.4, 30.4, 29.5, 25.7, 24.6, 21.3, 19.7. IR (neat): υ_{max} (cm^{-1}) 2898, 2154, 1733, 1267, 755. HRMS (ESI) calcd for $C_{10}H_{21}N_4O_2$ $[M+NH_4]^+$ 229.1659, found 229.1654.

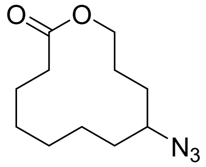

9-Azidooxacyclododecan-2-one (3m) Colorless oil (16.2 mg, 36%). R_f = 0.3 (petroleum ether/ethyl acetate = 40:1). ^1H NMR (400 MHz, $CDCl_3$) δ 4.53 – 4.89 (m, 1H), 3.98 – 392 (m, 1H), 3.67 – 3.61 (m, 1H), 2.45 – 2.32 (m, 2H), 1.85 – 1.52 (m, 8H), 1.48 – 1.31 (m, 6H). ^{13}C NMR (100 MHz, $CDCl_3$) δ 173.7, 64.3, 59.3, 34.0, 30.3, 28.5, 24.6, 24.5, 24.0, 23.5, 21.9. IR (neat): υ_{max} (cm^{-1}) 2906, 2162, 1743, 1254, 763. HRMS (ESI) calcd for $C_{11}H_{19}N_3O_2Na$ $[M+Na]^+$ 248.1369, found 248.1362.

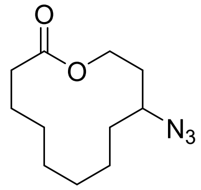

10-Azidooxacyclododecan-2-one (3n) Colorless oil (24.4 mg, 54%). R_f = 0.3 (petroleum ether/ethyl acetate = 40:1). ^1H NMR (400 MHz, $CDCl_3$) δ 4.54 – 4.90 (m, 1H), 4.08 – 4.03 (m, 1H), 3.67 – 3.61 (m, 1H), 2.48 – 2.42 (m, 1H), 2.34 – 2.27 (m, 1H), 1.94 – 1.86 (m, 1H), 1.82 – 172 (m, 3H), 1.67 – 1.59 (m, 2H), 1.50 – 1.28 (m, 8H). ^{13}C NMR (100 MHz, $CDCl_3$) δ 173.6, 60.9, 58.9, 34.1, 31.5, 30.5, 24.6, 24.3, 24.2, 23.5, 21.2. IR (neat): υ_{max} (cm^{-1}) 2936, 2094, 1455, 1232, 1143, 825. HRMS (ESI) calcd $C_{11}H_{19}N_3O_2Na$ $[M+Na]^+$ 248.1369, found 248.1369.

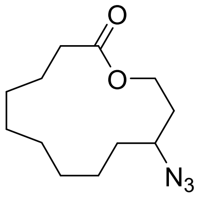

11-azidooxacyclotridecan-2-one (3o) Colorless oil (24.5 mg, 51%). R_f = 0.3 (petroleum ether/ethyl acetate = 50:1). ^{1}H NMR (400 MHz, $CDCl_3$) δ 4.26 – 4.24 (m, 2H), 3.50 – 3.44 (m, 1H), 2.45 – 2.27 (m, 2H), 1.95 – 1.87 (m, 1H), 1.82 – 1.75 (m, 1H), 1.69 – 1.60 (m, 4H), 1.57 – 1.48 (m, 1H), 1.44 – 1.30 (m, 9H). ^{13}C NMR (100 MHz, $CDCl_3$) δ 174.0, 61.5, 58.7, 34.5, 33.1, 32.3, 26.7, 26.2, 25.5, 25.4, 24.5, 22.6. IR (neat): υ_{max} (cm^{-1}) 2912, 2160, 1753, 12561, 755. HRMS (ESI) calcd $C_{12}H_{22}N_3O_2$ $[M+H]^+$ 240.1706, foun 240.1699.

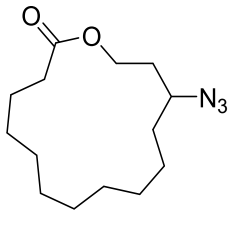

13-Azidooxacyclopentadecan-2-one (3p) Colorless oil (36.1 mg, 68%). R_f = 0.3 (petroleum ether/ethyl acetate = 30:1). ^{1}H NMR (400 MHz, $CDCl_3$) δ 4.27 (ddd, J = 11.6, 6.0, 3.6 Hz, 1H), 4.17 (ddd, J = 16.0, 8.0, 3.6 Hz, 1H), 3.50 – 3.43 (m, 1H), 2.38 – 2.34 (m, 2H), 1.91 – 1.78 (m, 2H), 1.71 – 1.62 (m, 3H), 1.61 – 1.52 (m, 3H), 1.48 – 1.41 (m, 2H), 1.38 – 1.27 (m, 10H). ^{13}C NMR (100 MHz, $CDCl_3$) δ 174.1, 61.0, 58.3, 34.1, 33.8, 32.0, 27.9, 26.7, 26.62, 26.60, 26.1, 25.6, 24.9, 23.9. IR (neat): υ_{max} (cm^{-1}) 2929, 2092, 1730, 1244, 756. HRMS (ESI) calcd for $C_{14}H_{25}N_3O_2Na$ $[M+Na]^+$ 290.1839, found 290.1850.

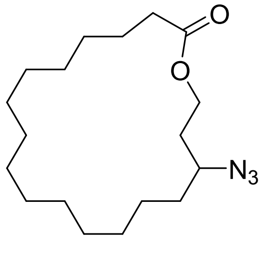

17-Azidooxacyclononadecan-2-one (3q) Colorless oil (44 mg, 68%). R_f = 0.3 (petroleum ether/ethyl acetate = 100:1). ^{1}H NMR (400 MHz, $CDCl_3$) δ 4.28 – 4.14 (m, 2H), 3.47 – 3.40 (m, 1H), 2.32 (t, J = 7.2 Hz, 2H), 1.91 – 1.83 (m, 1H), 1.77 – 1.69 (m, 1H), 1.65 – 1.51 (m, 4H), 1.46 – 1.41 (m, 2H), 1.35 – 1.26 (m, 20H). ^{13}C NMR (100 MHz, $CDCl_3$) δ 173.8, 60.9, 59.4, 34.5, 34.0, 33.5, 28.49, 28.46, 28.1, 27.9, 27.7, 27.61, 27.57 (overlapped), 27.4, 27.0, 25.4, 24.9. IR (neat): υ_{max} (cm^{-1}) 2924, 2095, 1734,

1450, 1241, 796. HRMS (ESI) calcd for $C_{18}H_{33}N_3O_2Li$ $[M+Li]^+$ 330.2727, found 330.2732.

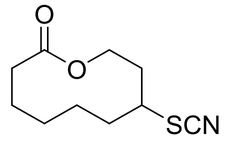

8-Thiocyanatooxecan-2-one (4a) Colorless oil (39.5 mg, 93%). R_f = 0.3 (petroleum ether/ethyl acetate = 5:1). ^{1}H NMR (400 MHz, $CDCl_3$) δ 4.45 (ddd, *J* = 12.0, 7.6, 2.4 Hz, 1H), 4.34 (ddd, *J* = 12.0, 7.6, 2.4 Hz, 1H), 3.71 – 3.65 (m, 1H), 2.44 – 2.28 (m, 3H), 2.17 – 2.08 (m, 1H), 2.04 – 1.90 (m, 2H), 1.89 – 1.73 (m, 2H), 1.61 – 1.46 (m, 2H), 1.42 – 1.30 (m, 2H). ^{13}C NMR (100 MHz, $CDCl_3$) δ 173.3, 111.9, 61.6, 48.4, 34.6, 30.2, 29.4, 26.9, 20.8, 20.4. IR (neat): υ_{max} (cm^{-1}) 2929, 2092, 1726, 1454, 1264, 755. HRMS (ESI) calcd for $C_{10}H_{16}NO_2S$ [M+H]$^+$ 214.0896, found 214.0883.

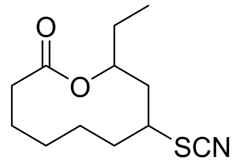

10-Ethyl-8-thiocyanatooxecan-2-one (4b) Colorless oil (41.0 mg, 85%). R_f= 0.3 (petroleum ether/ethyl acetate = 5:1). d.r. = 3.5:1.^{1}H NMR (400 MHz, $CDCl_3$) δ 4.91 – 4.85 (m, 1H), 3.88 – 3.83 (m, 0.2H), 3.24 (ddd, *J* = 13.6, 6.4, 3.2 Hz, 0.8H), 2.48 (ddd, *J* = 15.2, 6.0, 3.2 Hz, 1H), 2.42 – 2.32 (m, 0.2H), 2.28 – 2.17 (m, 2H), 2.14 – 1.99 (m, 1.8H), 1.97 – 1.87 (m, 1H), 1.78 – 1.54 (m, 6H), 1.41 – 1.33 (m, 0.8H), 1.27 – 1.20 (m, 0.8H), 1.15 – 1.06 (m, 0.2H), 0.97 – 0.89 (m, 3H). ^{13}C NMR (100 MHz, $CDCl_3$, two diastereoisomers) major isomer δ 174.0, 112.0, 75.3, 47.0, 39.3, 34.9, 32.3, 28.2, 25.9, 23.6, 20.7, 9.6; minor isomer δ 173.5, 119.2, 74.1, 46.2, 36.7, 34.7, 29.3, 26.6, 25.2, 20.9, 19.3, 10.3. IR (neat): υ_{max} (cm^{-1}) 2938, 2109, 1726, 1456, 1265, 755. HRMS (ESI) calcd for $C_{12}H_{19}NO_2SNa$ [M+Na]$^+$ 264.1029, found 264.1013.

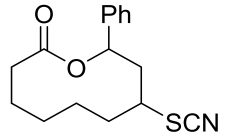

10-Phenyl-8-thiocyanatooxecan-2-one (4c) Colorless oil (40.6 mg, 70%). R_f = 0.3 (petroleum ether/ethyl acetate = 20:1). d.r. = 1:1 ^{1}H NMR (400 MHz, $CDCl_3$) δ 7.39 – 7.29 (m, 3H), 7.25 – 7.23 (m, 2H), 4.54 (dd, *J* = 12.0, 3.6 Hz, 1H), 4.13 (dt, *J* = 11.2, 3.6 Hz, 1H), 3.95 (t, *J* = 12.0 Hz, 1H), 3.56 (td, *J* = 11.6, 3.6 Hz, 1H), 2.56 (ddd, *J* = 16.8, 6.4, 2.0 Hz, 1H), 2.32 – 2.22 (m, 2H), 2.10 – 2.00 (m, 1H), 1.96 – 1.87 (m, 1H), 1.75 – 1.62 (m, 3H), 1.60 – 1.52 (m, 1H), 1.41 – 1.31 (m, 1H). ^{13}C NMR (100 MHz, $CDCl_3$, single isomer) δ 173.3, 137.8, 129.2, 128.2, 128.0, 65.5, 62.6, 45.9, 34.5, 30.3, 26.9, 20.5, 19.2.

IR (neat): υ_{max} (cm^{-1}) 2943, 2082, 1729, 1453, 1265, 755, 701. HRMS (ESI) calcd for $C_{16}H_{20}NO_2S$ $[M+H]^+$ 290.1209, found 290.1193.

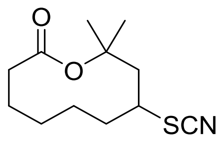

10,10-Dimethyl-8-thiocyanatooxecan-2-one (4d) Colorless oil (33.7 mg, 70%). R_f = 0.3 (petroleum ether/ethyl acetate = 10:1). ^1H NMR (400 MHz, $CDCl_3$) δ 3.90 – 3.84 (m, 1H), 2.84 (dd, *J* = 15.2, 11.6 Hz, 1H), 2.44 (ddd, *J* = 14.8, 6.8, 2.8 Hz, 1H), 2.25 – 2.18 (m, 1H), 2.14 – 2.04 (m, 1H), 2.01 – 1.90 (m, 1H), 1.86 – 1.76 (m, 2H), 1.70 – 1.44 (m, 4H), 1.66 (s, 3H), 1.37 (s, 3H), 1.31 – 1.21 (m, 1H). ^{13}C NMR (100 MHz, $CDCl_3$) δ 174.0, 112.2, 81.7, 47.5, 37.5, 36.3, 28.8, 28.2, 26.8, 26.5, 21.2, 18.8. IR (neat): υ_{max} (cm^{-1}) 2924, 2109, 1733, 1452, 1267, 755. HRMS (ESI) calcd for $C_{12}H_{23}N_2O_2S$ $[M+NH_4]^+$ 259.1474, found 259.1473.

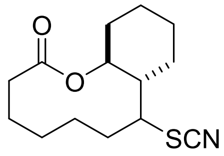

8-Thiocyanatododecahydro-2*H*-benzo[b]oxecin-2-one (4e) Colorless oil (43.0 mg, 81%). R_f = 0.3 (petroleum ether/ethyl acetate = 10:1). d.r. = 13:1. ^1H NMR (400 MHz, $CDCl_3$) δ 4.51 – 4.45 (m, 1H), 3.35 – 3.30 (m, 1H), 2.50 – 2.44 (m, 1H), 2.25 – 2.20 (m, 2H), 2.11 – 2.04 (m, 2H), 1.94 – 1.84 (m, 2H), 1.77 – 1.72 (m, 3H), 1.66 – 1.58 (m, 3H), 1.34 – 1.20 (m, 5H), 1.09 – 1.02 (m, 1H). ^{13}C NMR (100 MHz, $CDCl_3$, single isomer) δ 173.8, 129.9, 75.5, 63.0, 47.1, 35.1, 33.5, 32.5, 30.6, 26.3, 24.9, 24.4, 23.3, 20.8. IR (neat): υ_{max} (cm^{-1}) 2935, 2095, 1727, 1266, 755. HRMS (ESI) calcd for $C_{14}H_{21}NO_2SNa$ $[M+Na]^+$ 290.1185, found 290.1178.

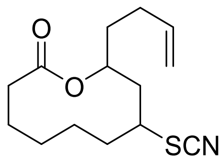

10-(But-3-en-1-yl)-8-thiocyanatooxecan-2-one (4f) Colorless oil (32.0 mg, 60%). R_f = 0.3 (petroleum ether/ethyl acetate = 15:1). d.r. = 3.5:1. ^1H NMR (400 MHz, $CDCl_3$) δ 5.83 – 5.73 (m, 1H), 5.07 – 4.95 (m, 3H), 3.90 – 3.85 (m, 0.22H), 3.29 – 3.23 (m, 0.77H), 2.53 – 2.37 (m, 1H), 2.30 – 2.00 (m, 6H), 1.96 – 1.57 (m, 8H), 1.43 – 1.29 (m, 1H). ^{13}C NMR (100 MHz, $CDCl_3$, two diastereoisomers) major isomer δ 174.0, 137.3, 115.5, 112.0, 73.8, 47.0, 39.6, 35.0, 34.4, 32.3, 29.5, 26.0, 23.6, 20.8; minor isomer δ

173.4, 136.9, 115.9, 119.9, 72.2, 46.3, 34.8, 32.6, 31.2, 29.9, 29.4, 26.6, 21.0, 19.3. IR (neat): υ_{max} (cm^{-1}) 2930, 2151, 1726, 1641, 1452, 1246, 916. HRMS (ESI) calcd for $C_{14}H_{21}NO_2SNa$ $[M+Na]^+$ 290.1185, found 290.1181.

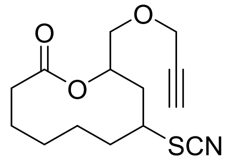

10-((Prop-2-yn-1-yloxy)methyl)-8-thiocyanatooxecan-2-one (4g) Colorless oil (37.4 mg, 67%). R_f = 0.3 (petroleum ether/ethyl acetate = 15:1). d.r. = 3.8:1. ^1H NMR (400 MHz, $CDCl_3$) δ 5.17 – 5.12 (m, 0.21H), 5.05 – 5.00 (m, 0.79H), 4.22 – 4.13 (m, 2H), 3.97 – 3.91 (m, 0.2H), 3.75 – 3.61 (m, 2H), 3.29 – 3.23 (m, 0.78H), 2.56 – 2.47 (m, 1H), 2.46 (t, J = 2.4 Hz, 1H), 2.40 – 2.20 (m, 3H), 2.15 – 1.91 (m, 2H), 1.97 – 1.54 (m, 4H), 1.52 – 1.35 (m, 1H), 1.22 – 1.09 (m, 1H). ^{13}C NMR (100 MHz, $CDCl_3$, two diastereoisomers) major isomer δ 173.9, 111.8, 79.1, 75.3, 72.5 (overlapped), 70.7, 58.6, 47.0, 36.9, 34.9, 32.3, 26.2, 23.8, 20.6; minor isomer δ 173.2, 111.9, 79.0, 75.4, 72.5 (overlapped), 69.7, 58.7, 46.6, 34.8, 31.4, 29.7, 26.6, 21.1, 19.8. IR (neat): υ_{max} (cm^{-1}) 2925, 2123, 1726, 1458, 1237, 1089, 710. HRMS (ESI) calcd for $C_{14}H_{19}NO_3SNa$ $[M+Na]^+$ 304.0977, found 304.0975.

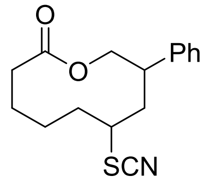

9-Phenyl-7-thiocyanatooxecan-2-one (4h) Colorless oil (40.6 mg, 70%). R_f = 0.3 (petroleum ether/ethyl acetate = 20:1). d.r. = 1.5:1. ^1H NMR (400 MHz, $CDCl_3$) δ 7.36 – 7.32 (m, 2H), 7.30 – 7.27 (m, 1H), 7.25 – 7.20 (m, 2H), 4.79 (ddd, J = 11.2, 3.6, 1.6 Hz, 0.4H), 4.58 (t, J = 11.6 Hz, 0.6H), 4.15 – 4.05 (m, 0.7H), 3.95 (t, J = 11.6 Hz, 0.3H), 3.93 – 3.86 (m, 0.4H), 3.64 – 3.58 (m, 0.6H), 3.49 – 3.41 (m, 0.4H), 3.30 (ddd, J = 16.4, 8.4, 4.4 Hz, 0.6H), 2.69 – 2.57 (m, 1H), 2.51 (ddd, J = 14.0, 12.4, 4.4 Hz, 0.4H), 2.37 – 2.28 (m, 1H), 2.26 – 2.17 (m, 1.6H), 2.04 –1.85 (m, 3H), 1.82 – 1.76 (m, 1H), 1.65 – 1.58 (m, 1H), 1.56 – 1.45 (m, 1H). ^{13}C NMR (100 MHz, $CDCl_3$, two diastereoisomers) major isomer δ 173.1, 140.5, 129.03, 127.6 (overlapped), 127.4 (overlapped), 112.0, 68.8, 48.0, 45.0, 42.7, 40.0, 34.8, 24.3, 20.5; minor isomer δ 172.7, 139.4, 128.96, 127.6 (overlapped), 127.4 (overlapped), 111.4, 68.9, 46.0, 42.5, 34.7, 34.4, 28.4, 25.1, 20.2. IR (neat): υ_{max} (cm^{-1}) 2853, 2367, 1764, 1478, 1267, 755. HRMS (ESI) calcd for $C_{16}H_{20}NO_2S$ $[M+H]^+$ 290.1209, found 290.1189.

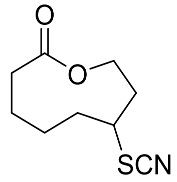

7-Thiocyanatooxonan-2-one (4i) Colorless oil (34.8 mg, 87%). R_f = 0.3 (petroleum ether/ethyl acetate = 8:1). ^{1}H NMR (400 MHz, $CDCl_3$) δ 4.44 – 4.33 (m, 2H), 3.55 – 3.49 (m, 1H), 2.40 – 2.16 (m, 4H), 1.90 – 1.76 (m, 3H), 1.75 – 1.66 (m, 3H). ^{13}C NMR (100 MHz, $CDCl_3$) δ 174.5, 111.8, 61.6, 46.2, 34.3, 34.1, 33.7, 24.3, 22.0. IR (neat): υ_{max} (cm^{-1}) 2949, 2208, 1733, 1457, 1266, 755. HRMS (ESI) calcd for $C_9H_{13}NO_2SNa$ $[M+Na]^+$ 222.0559, found 222.0543.

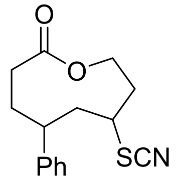

5-Phenyl-7-thiocyanatooxonan-2-one (4j) Colorless oil (38.5 mg, 70%). R_f = 0.3 (petroleum ether/ethyl acetate = 5:1). d.r. = 1.4:1. ^{1}H NMR (400 MHz, $CDCl_3$) δ 7.35 – 7.30 (m, 2H), 7.25 – 7.20 (m, 2H), 7.16 – 7.14 (m, 1H), 4.95 – 4.89 (m, 0.4H), 4.63 (td, *J* = 11.6, 2.8 Hz, 0.6H), 4.30 (ddd, *J* = 11.6, 4.0, 2.8 Hz, 0.6H), 4.13 (ddd, *J* = 12.0, 4.8, 3.6 Hz, 0.4H), 3.63 – 3.55 (m, 1H), 3.05 – 2.99 (m, 1H), 2.71 – 2.63 (m, 0.4H), 2.51 – 2.39 (m, 2.6H), 2.24 – 1.89 (m, 5H). ^{13}C NMR (100 MHz, $CDCl_3$, two diastereoisomers) major isomer δ 174.5, 146.9, 129.0, 127.3 (overlapped), 127.0, 111.7 (overlapped), 62.3, 44.9, 42.8, 40.3, 35.0, 34.5, 33.0; minor isomer δ 174.0, 146.1, 129.1, 127.3 (overlapped), 126.9, 111.7 (overlapped), 60.5, 45.5, 41.6, 41.1, 33.1, 33.0 (overlapped), 32.4. IR (neat): υ_{max} (cm^{-1}) 2947, 2150, 1734, 1453, 1267, 755, 702. HRMS (ESI) calcd for $C_{15}H_{17}NO_2SNa$ $[M+Na]^+$ 298.0872, found 298.0856.

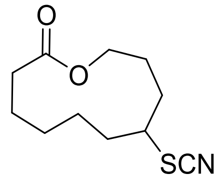

8-Thiocyanatooxacycloundecan-2-one (4k) Colorless oil (28.2 mg, 62%). R_f = 0.3 (petroleum ether/ethyl acetate = 10:1). ^{1}H NMR (400 MHz, $CDCl_3$) δ 4.27 – 4.15 (m, 2H), 3.40 – 3.36 (m, 1H), 2.47 – 2.41 (m, 1H), 2.31 – 2.24 (m, 1H), 2.12 – 1.94 (m, 4H), 1.86 – 1.55 (m, 6H), 1.44 – 1.35 (m, 1H), 1.29 – 1.21 (m, 1H). ^{13}C NMR (100 MHz, $CDCl_3$) δ 173.6, 111.8, 64.2, 48.1, 35.1, 32.2, 28.6, 25.7, 24.3, 23.2, 21.3. IR (neat): υ_{max} (cm^{-1}) 2867, 2358, 1729, 1438, 1267, 755. HRMS (ESI) calcd for

$C_{11}H_{17}NO_2SLi$ $[M+Li]^+$ 234.1135, found 234.1126.

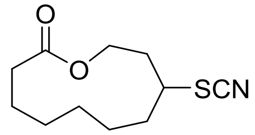

9-Thiocyanatooxacycloundecan-2-one (4l) Colorless oil (31.8 mg, 70%). R_f = 0.3 (petroleum ether/ethyl acetate = 10:1). ^{1}H NMR (400 MHz, $CDCl_3$) δ 4.30 – 4.23 (m, 2H), 3.57 – 3.51 (m, 1H), 2.43 – 2.18 (m, 4H), 1.79 – 1.71 (m, 3H), 1.66 – 1.59 (m, 1H), 1.56 – 1.50 (m, 2H), 1.48 – 1.44 (m, 1H), 1.41 – 1.36 (m, 3H). ^{13}C NMR (100 MHz, $CDCl_3$) δ 173.6, 111.9, 61.4, 47.2, 34.9, 32.1, 31.1, 24.92, 24.89, 21.5, 21.1. IR (neat): υ_{max} (cm^{-1}) 2896, 2208, 1735, 1267, 755. HRMS (ESI) calcd for $C_{11}H_{21}N_2O_2S$ $[M+NH_4]^+$ 245.1318, found 245.1319.

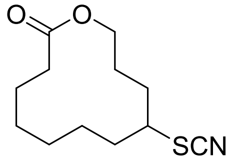

9-Thiocyanatooxacyclododecan-2-one (4m) Colorless oil (20.0 mg, 41%). R_f = 0.3 (petroleum ether/ethyl acetate = 20:1). ^{1}H NMR (400 MHz, $CDCl_3$) δ 4.63 – 4.58 (m, 1H), 3.95 – 3.89 (m, 1H), 3.54 – 3.48 (m, 1H), 2.46 – 2.32 (m, 2H), 2.05 (q, J = 6.4 Hz, 2H), 1.97 – 1.76 (m, 4H), 1.72 – 1.59 (m, 3H), 1.54 – 1.44 (m, 1H), 1.43 – 1.29 (m, 4H). ^{13}C NMR (100 MHz, $CDCl_3$) δ 173.5, 111.7, 64.4, 47.3, 33.6, 33.3, 29.7, 25.2, 24.4, 23.6, 23.2, 22.8. IR (neat): υ_{max} (cm^{-1}) 2911, 2213, 1745, 1278, 765. HRMS (ESI) calcd for $C_{12}H_{19}NO_2SNa$ $[M+Na]^+$ 264.1028, found 264.1026.

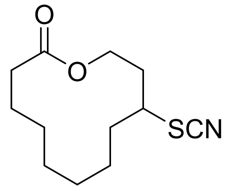

10-Thiocyanatooxacyclododecan-2-one (4n) Colorless oil (31.7 mg, 66%). R_f = 0.3 (petroleum ether/ethyl acetate = 15:1). ^{1}H NMR (400 MHz, $CDCl_3$) 4.65 – 4.60 (m, 1H), 4.06 – 4.00 (m, 1H), 3.46 – 3.40 (m, 1H), 2.50 – 2.44 (m, 1H), 2.32 – 2.45 (m, 1H), 2.22 – 2.14 (m, 1H), 2.09 – 1.92 (m, 2H), 1.83 – 1.71 (m, 2H), 1.65 – 1.51 (m, 2H), 1.48 – 1.29 (m, 7H). ^{13}C NMR (100 MHz, $CDCl_3$) δ 173.4, 111.4, 60.9, 45.9, 34.4, 33.2, 32.5, 25.0, 24.4, 23.7, 23.2, 22.3. IR (neat): υ_{max} (cm^{-1}) 2932, 2150, 1729, 1452, 1231, 1038, 799. HRMS (ESI) calcd for $C_{12}H_{19}NO_2SNa$ $[M+Na]^+$ 264.1028, found 264.1025.

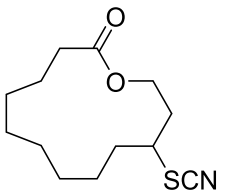

11-Thiocyanatooxacyclotridecan-2-one (4o) Colorless oil (30.7 mg, 60%). R_f = 0.3 (petroleum ether/ethyl acetate = 20:1). ^{1}H NMR (400 MHz, $CDCl_3$) δ 4.41 – 4.34 (m, 1H), 4.23 – 4.22 (m, 1H), 3.16 – 3.09 (m, 1H), 2.47 – 2.40 (m, 1H), 2.32 – 2.25 (m, 1H), 2.21 – 2.03 (m, 2H), 1.95 – 1.76 (m, 2H), 1.68 – 1.54 (m, 3H), 1.42 – 1.29 (m, 9H). ^{13}C NMR (100 MHz, $CDCl_3$) δ 173.8, 111.1, 61.2, 45.9, 34.9, 34.5, 33.8, 26.6, 26.2, 25.1, 24.8, 24.4, 23.5. IR (neat): υ_{max} (cm^{-1}). 2907, 2213, 1745, 1287, 762. HRMS (ESI) calcd for $C_{13}H_{21}NO_2SNa$ $[M+Na]^+$ 278.1185, found 278.1182.

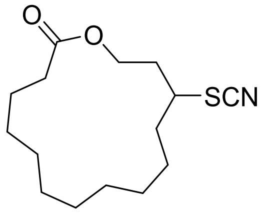

13-Thiocyanatooxacyclopentadecan-2-one (4p) Colorless oil (26.0 mg, 46%). R_f = 0.3 (petroleum ether/ethyl acetate = 20:1). ^{1}H NMR (400 MHz, $CDCl_3$) δ 4.30 – 4.27 (m, 2H), 3.18 – 3.11 (m, 1H), 2.42 – 2.29 (m, 2H), 2.16 – 2.12 (m, 2H), 1.94 – 1.82 (m, 1H), 1.79 – 1.71 (m, 1H), 1.70 – 1.61 (m, 3H), 1.58 – 1.48 (m, 1H), 1.36 – 1.25 (m, 12H). ^{13}C NMR (100 MHz, $CDCl_3$) δ 173.9, 110.7, 61.1, 46.3, 35.5, 33.8, 32.4, 27.8, 26.7, 26.6, 26.4, 25.6, 25.3, 24.7, 24.6. IR (neat): υ_{max} (cm^{-1}) 2928, 2130, 1732, 1452, 1267, 755. HRMS (ESI) calcd for $C_{15}H_{25}NO_2SNa$ $[M+Na]^+$ 306.1498, found 306.1479.

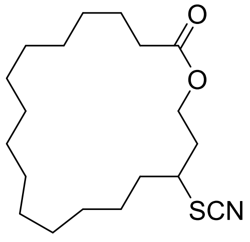

17-Thiocyanatooxacyclononadecan-2-one (4q) Colorless oil (51.6 mg, 76%). R_f = 0.3 (petroleum ether/ethyl acetate = 20:1). ^{1}H NMR (400 MHz, $CDCl_3$) δ 4.33 – 4.22 (m, 2H), 3.17 – 3.10 (m, 1H), 2.32 (t, J = 7.2 Hz, 2H), 2.19 – 2.00 (m, 2H), 1.83 – 1.78 (m, 2H), 1.64 – 1.60 (m, 2H), 1.53 – 1.46 (m, 2H), 1.35 – 1.27 (m, 20H). ^{13}C NMR (100 MHz, $CDCl_3$) δ 173.7, 110.8, 60.9, 47.4, 34.9, 34.8, 34.4, 28.4, 28.3, 28.0, 27.9, 27.7, 27.43, 27.37 (overlapped), 27.3, 26.9, 26.3, 24.8. IR (neat): υ_{max} (cm^{-1}) 2924, 2201, 1733, 1450, 1267, 755. HRMS (ESI) calcd for $C_{19}H_{34}NO_2S$ $[M+H]^+$ 340.2305, found 340.2302.

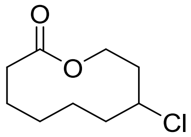

8-Chlorooxecan-2-one (5a) Colorless oil (33.6 mg, 88%). R_f = 0.3 (petroleum ether/ethyl acetate = 40:1). ^{1}H NMR (400 MHz, $CDCl_3$) δ 4.48 – 4.42 (m, 1H), 4.37 – 4.35 (m, 1H), 4.27 – 4.21 (m, 1H), 2.47 – 2.37 (m, 2H), 2.29 (ddd, *J* = 16.4, 10.0, 2.4 Hz, 1H), 2.13 – 1.91 (m, 4H), 1.78 – 1.69 (m, 1H), 1.55 – 1.50 (m, 2H), 1.42 – 1.33 (m, 1H), 1.29 – 1.21 (m, 1H). ^{13}C NMR (100 MHz, $CDCl_3$) δ 173.4, 61.1, 60.1, 34.6, 33.9, 32.1, 27.1, 20.6, 20.4. IR (neat): υ_{max} (cm^{-1}) 2931, 1725, 1454, 1266, 755. HRMS (ESI) calcd for $C_9H_{15}ClO_2K$ $[M+K]^+$ 229.0392, found 229.0388.

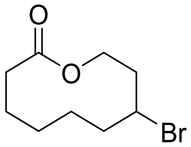

8-Bromooxecan-2-one (5a-2) Colorless oil (26.6 mg, 57%). R_f = 0.3 (petroleum ether/ethyl acetate = 40:1). ^{1}H NMR (400 MHz, $CDCl_3$) δ 4.47 – 4.34 (m, 3H), 2.48 – 2.39 (m, 2H), 2.33 – 2.26 (m, 1H), 2.21 – 2.07 (m, 3H), 1.95 – 1.87 (m, 1H), 1.75 – 1.73 (m, 1H), 1.56 – 1.48 (m, 2H), 1.42 – 1.31 (m, 1H), 1.27 – 1.24 (m, 1H). ^{13}C NMR (100 MHz, $CDCl_3$) δ 173.5, 62.3, 53.2, 34.8, 34.7, 32.7, 27.1, 21.4, 20.5. IR (neat): υ_{max} (cm^{-1}) 2923, 1726, 1455, 1267, 755. HRMS (ESI) calcd for $C_9H_{15}BrO_2K$ $[M+K]^+$ 272.9887, found 272.9891.

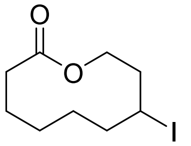

8-Iodooxecan-2-one (5a-3) Colorless oil (25.0 mg, 44%). R_f = 0.3 (petroleum ether/ethyl acetate = 40:1). ^{1}H NMR (400 MHz, $CDCl_3$) δ 4.54 – 4.48 (m, 1H), 4.39 – 4.34 (m, 2H), 2.44 – 2.25 (m, 5H), 2.19 – 2.11 (m, 1H), 1.94 – 1.85 (m, 1H), 1.78 – 1.73 (m, 1H), 1.59 – 1.44 (m, 2H), 1.39 – 1.24 (m, 2H). ^{13}C NMR (100 MHz, $CDCl_3$) δ 173.5, 64.2, 36.8, 34.7, 34.5, 32.4, 27.0, 22.7, 20.6. IR (neat): υ_{max} (cm^{-1}) 2930, 1727, 1453, 1267, 755. HRMS (ESI) calcd for $C_9H_{16}IO_2$ $[M+H]^+$ 283.0189, found 283.0177.

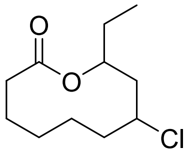

8-Chloro-10-ethyloxecan-2-one (5b) Colorless oil (36.3 mg, 83%). R_f = 0.3 (petroleum ether/ethyl

acetate = 40:1). d.r. = 2.7:1. ^{1}H NMR (400 MHz, $CDCl_3$) δ 4.87 – 4.76 (m, 1H), 4.44 – 4.38 (m, 0.3H), 4.04 – 3.98 (m, 0.7H), 2.51 – 2.40 (m, 1.4H), 2.28 – 2.20 (m, 2H), 2.18 – 2.12 (m, 0.7H), 2.09 – 1.91 (m, 2.3H), 1.83 – 1.56 (m, 6H), 1.45 – 1.38 (m, 0.8H), 1.29 – 1.21 (m, 1H), 0.96 – 0.89 (m, 3H). ^{13}C NMR (100 MHz, $CDCl_3$, two diastereoisomers) major isomer δ 174.6, 75.2, 59.6, 40.6, 35.0, 34.9, 28.1, 26.5, 22.0, 21.4, 9.9; minor isomer δ 173.8, 74.2, 58.6, 36.0, 34.9 (overlapped), 32.5, 26.9, 25.4, 21.1, 19.2, 10.4. IR (neat): υ_{max} (cm^{-1}) 2933, 1725, 1455, 1263, 755. HRMS (ESI) calcd for $C_{11}H_{19}ClO_2$ $[M]^+$ 218.1068, found 218.1060.

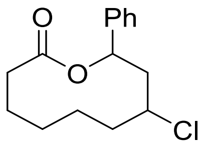

8-Chloro-10-phenyloxecan-2-one (5c) Colorless oil (46.4 mg, 87%). R_f = 0.3 (petroleum ether/ethyl acetate = 40:1). d.r. = 1:1.^{1}H NMR (400 MHz, $CDCl_3$) δ 7.38 – 7.30 (m, 2H), 7.29 – 7.25 (m, 2H), 7.20 – 7.18 (m, 1H), 4.78 (d, *J* = 10.0 Hz, 0.5H), 4.66 (t, *J* = 11.2 Hz, 0.5H), 4.59 (dd, *J* = 11.6, 3.2 Hz, 0.5H),4.53 (dt, *J* = 11.2, 3.2 Hz, 0.5H), 4.21 (ddd, *J* = 12.0, 4.8, 2.4 Hz, 0.5H), 3.90 (t, *J* = 12.0 Hz, 0.5H), 3.78 (dt, *J* = 11.2, 2.4 Hz, 0.5H), 3.60 (td, *J* = 11.6, 3.6 Hz, 0.5H), 2.63 – 2.56 (m, 1H), 2.52 – 2.38 (m, 1H), 2.28 (ddd, *J* = 16.4, 12.4, 2.0 Hz, 1H), 2.15 – 1.93 (m, 2H), 1.85 –1.54 (m, 4H), 1.38 – 1.28 (m, 1H). ^{13}C NMR (100 MHz, $CDCl_3$, both diastereoisomers) δ 173.5, 173.2, 139.1, 139.0, 128.8, 128.4, 128.3, 127.9, 127.6, 127.3, 66.7, 66.4, 65.7, 63.1, 47.5, 34.7, 34.6, 34.5, 32.4, 27.3, 27.0, 21.8, 20.7, 20.2, 19.1. IR (neat): υ_{max} (cm^{-1}) 2946, 2156, 1729, 1453, 1263, 755, 695. HRMS (ESI) calcd for $C_{15}H_{20}ClO_2$ $[M+H]^+$ 267.1146, found 267.1135.

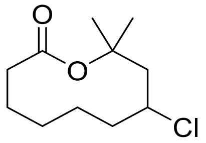

8-Chloro-10,10-dimethyloxecan-2-one (5d) Colorless oil (30.0 mg, 69%). R_f = 0.3 (petroleum ether/ethyl acetate = 50:1). ^{1}H NMR (400 MHz, $CDCl_3$) δ 4.45 – 4.39 (m, 1H), 2.94 (dd, *J* = 15.2, 11.2 Hz, 1H), 2.46 – 2.40 (m, 1H), 2.16 – 2.04 (m, 2H), 1.99 – 1.77 (m, 3H), 1.67 – 1.56 (m, 3H), 1.63 (s, 3H), 1.51 – 1.41 (m, 1H), 1.32 (s, 3H), 1.30 – 1.24 (m, 1H). ^{13}C NMR (100 MHz, $CDCl_3$) δ 173.8, 62.7, 42.7, 34.9, 30.4, 29.0, 27.3, 25.0, 21.4, 20.6, 14.9. IR (neat): υ_{max} (cm^{-1}) 2925, 1725, 1457, 1266, 755. HRMS (ESI) calcd for $C_{11}H_{23}ClNO_2$ $[M+NH_4]^+$ 236.1411, found 236.1415.

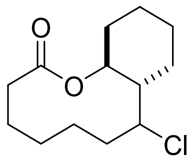

8-Chlorododecahydro-2*H*-benzo[b]oxecin-2-one (5e) Colorless oil (39.8 mg, 82%). R_f = 0.3 (petroleum ether/ethyl acetate = 40:1). d.r. = 20:1, ^{1}H NMR (400 MHz, $CDCl_3$) δ 4.55 – 4.49 (m, 1H), 3.71 – 3.66 (m, 1H), 2.49 – 2.40 (m, 2H), 2.30 – 2.23 (m, 1H), 2.12 – 2.04 (m, 2H), 1.94 – 1.90 (m, 2H), 1.80 – 1.73 (m, 3H), 1.67 – 1.60 (m, 3H), 1.42 – 1.28 (m, 5H), 1.01 – 0.91 (m, 1H). ^{13}C NMR (100 MHz, $CDCl_3$, single isomer) δ 173.4, 75.1, 66.9, 47.3, 34.6, 34.2, 31.9, 30.7, 25.7, 24.3, 23.8, 21.8, 20.8. IR (neat): υ_{max} (cm^{-1}) 2929, 1725, 1453, 1265, 755. HRMS (ESI) calcd for $C_{13}H_{25}ClNO_2$ $[M+NH_4]^+$ 262.1568, found 262.1577.

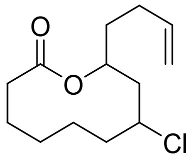

10-(but-3-en-1-yl)-8-chlorooxecan-2-one (5f) Colorless oil (38.8 mg, 80%). R_f = 0.3 (petroleum ether/ethyl acetate = 50:1). d.r. = 3.3:1, ^{1}H NMR (400 MHz, $CDCl_3$) δ 5.84 – 5.73 (m, 1H), 5.04 – 4.86 (m, 3H), 4.44 – 4.38 (m, 0.22H), 4.05 – 3.99 (m, 0.73H), 2.50 – 2.40 (m, 1H), 2.30 – 1.91 (m, 7H), 1.88 – 1.69 (m, 3H), 1.68 – 1.49 (m, 3H), 1.48 – 1.34 (m, 1H), 1.29 – 1.20 (m, 1H). ^{13}C NMR (100 MHz, $CDCl_3$, two diastereoisomers) major isomer δ 174.4, 137.6, 115.3, 73.6, 59.5, 40.4, 35.0, 34.8, 34.1, 29.8, 26.5, 21.9, 21.3; minor isomer δ 173.6, 137.3, 115.6, 72.3, 58.5, 36.3, 34.8, 32.5, 31.4, 30.0, 26.9, 21.0, 19.1. IR (neat): υ_{max} (cm^{-1}) 3938, 1727, 1642, 1433, 1212, 860. HRMS (ESI) calcd for $C_{13}H_{21}ClO_2Na$ $[M+Na]^+$ 267.1122, found 267.1120.

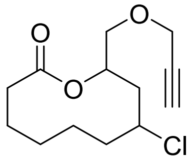

8-Chloro-10-((prop-2-yn-1-yloxy)methyl)oxecan-2-one (5g) Colorless oil (39.0 mg, 76%). R_f = 0.3 (petroleum ether/ethyl acetate = 50:1). d.r. = 3.:1, ^{1}H NMR (400 MHz, $CDCl_3$) δ 5.15 – 5.10 (m, 0.24H), 5.01 – 4.96 (m, 0.75), 4.47 – 4.41 (m, 0.24H), 4.20 – 4.17 (m, 2H), 4.05 – 3.99 (m, 0.77H), 3.78 – 3.65 (m, 2H), 2.52 – 2.35 (m, 3H), 2.28 – 2.17 (m, 2H), 2.11 – 1.94 (m, 2H), 1.88 – 1.76 (m, 1H), 1.68 – 1.53 (m, 3H), 1.47 – 1.41 (m, 1H), 1.24 – 1.13 (m, 1H). ^{13}C NMR (100 MHz, $CDCl_3$, two diastereoisomers) major isomer δ 174.2, 79.3, 75.1, 72.5, 71.0, 59.2, 58.6, 37.8, 34.9 (overlapped), 34.8, 26.7, 22.0, 21.1;

minor isomer δ 173.6, 79.2, 75.2, 70.9, 70.1, 58.8, 58.6, 36.8, 34.9 (overlapped), 32.7, 26.9, 21.2, 19.7. IR (neat): υ_{max} (cm^{-1}) 3294, 2091, 1721, 1445, 1205, 875. HRMS (ESI) calcd for $C_{13}H_{19}ClO_3Na$ $[M+Na]^+$ 281.0914, found 281.0916.

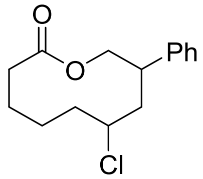

7-Chloro-9-phenyloxecan-2-one (5h) Colorless oil (32.6 mg, 61%). R_f = 0.3 (petroleum ether/ethyl acetate = 40:1). d.r. = 4.5:1, ^1H NMR (400 MHz, $CDCl_3$) δ 7.35 – 7.31 (m, 2H), 7.28 – 7.23 (m, 2H), 7.22 – 7.20 (m, 1H), 4.75 – 4.67 (m, 0.3H), 4.46 (t, *J* = 11.6 Hz, 0.8H), 4.34 (ddd, *J* = 10.8, 6.0, 4.4 Hz, 0.8H), 4.09 (dd, *J* = 11.2, 4.0 Hz, 0.8H), 3.91 (t, *J* = 11.6 Hz, 0.1H), 3.46 –3.24 (m, 1H), 2.64 – 2.44 (m, 1H), 2.36 – 2.06 (m, 3H), 2.19 – 2.05 (m, 0.4H), 1.97 –1.65 (m, 5H), 1.59 – 1.43 (m, 1H). ^{13}C NMR (100 MHz, $CDCl_3$, two diastereoisomers) major isomer δ 173.6, 141.2, 129.0, 127.5, 127.36, 68.8, 60.1, 44.7, 44.3 (overlapped), 37.3, 34.9, 23.7, 20.9; minor isomer δ 173.1, 140.2, 128.9, 127.7, 127.41, 69.0, 58.1, 44.3 (overlapped), 40.3, 34.6, 32.5, 25.0, 20.6. IR (neat): υ_{max} (cm^{-1}) 2341, 1728, 1263, 737, 700. HRMS (ESI) calcd for $C_{15}H_{20}ClO_2$ $[M+H]^+$ 267.1146, found 267.1134.

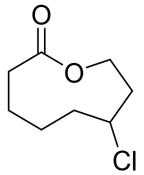

7-Chlorooxonan-2-one (5i) Colorless oil (25.0 mg, 71%). R_f = 0.3 (petroleum ether/ethyl acetate = 40:1). ^1H NMR (400 MHz, $CDCl_3$) δ 4.39 (ddd, *J* = 11.6, 6.8, 3.2 Hz, 1H), 4.31 (ddd, *J* = 11.6, 8.0, 2.4 Hz, 1H), 4.19 – 4.13 (m, 1H), 2.39 – 2.30 (m, 1H), 2.31 (t, *J* = 6.8 Hz, 2H), 2.27 – 2.18 (m, 1H), 1.93 – 1.87 (m, 2H), 1.79 – 1.56 (m, 4H). ^{13}C NMR (100 MHz, $CDCl_3$) δ 174.6, 61.6, 58.9, 36.8, 36.3, 33.9, 24.5, 21.0. IR (neat): υ_{max} (cm^{-1}) 2251, 1733, 1455, 1265, 755. HRMS (ESI) calcd for $C_8H_{13}ClO_2Na$ $[M+Na]^+$ 199.0496, found 199.0480.

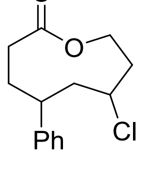

7-Chloro-5-phenyloxonan-2-one (5j) Colorless oil (34.1 mg, 68%). R_f = 0.3 (petroleum ether/ethyl

acetate = 40:1). d.r. = 1:1, ^{1}H NMR (400 MHz, $CDCl_3$) δ 7.34 – 7.28 (m, 2H), 7.25 – 7.19 (m, 2H), 7.18 – 7.14 (m, 1H), 5.03 – 4.97 (m, 0.5H), 4.62 (td, *J* = 11.6, 2.4 Hz, 0.5H), 4.27 – 4.22 (m, 0.5H), 4.18 (dt, *J* = 11.6, 3.6 Hz, 0.5H), 4.15 – 4.05 (m, 1H), 3.11 – 3.06 (m, 0.5H), 2.92 – 2.87 (m, 0.5H), 2.76 – 2.67 (m, 0.5H), 2.54 – 2.39 (m, 2.5H), 2.37 – 2.20 (m, 2H), 2.18 – 2.03 (m, 2H), 1.96 – 1.89 (m, 1H). ^{13}C NMR (100 MHz, $CDCl_3$, both diastereoisomers) δ 174.5, 174.1, 148.7, 147.1, 128.9, 128.7, 127.3, 127.0, 126.7, 126.3, 62.3, 60.8, 57.5, 57.1, 45.5, 45.5, 41.0, 39.7, 37.8, 35.9, 34.4, 33.9, 33.5, 32.8. IR (neat): υ_{max} (cm^{-1}) 2949, 1736, 1453, 1238, 1147, 756, 700. HRMS (ESI) calcd for $C_{14}H_{18}ClO_2$ $[M+H]^+$ 253.0989, found 253.0969.

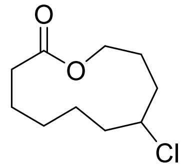

8-Chlorooxacycloundecan-2-one (5k) Colorless oil (23.3 mg, 57%). R_f = 0.3 (petroleum ether/ethyl acetate = 50:1). ^{1}H NMR (400 MHz, $CDCl_3$) δ 4.27 – 4.07 (m, 3H), 2.48 – 2.41 (m, 1H), 2.31 – 2.23 (m, 1H), 2.13 – 1.97 (m, 4H), 1.77 – 1.56 (m, 6H), 1.37 – 1.30 (m, 2H). ^{13}C NMR (100 MHz, $CDCl_3$) δ 173.8, 64.4, 60.7, 35.3, 34.3, 32.0, 26.1, 23.9, 22.7, 21.1. IR (neat): υ_{max} (cm^{-1}) 2924, 2389, 1727, 1267, 755. HRMS (ESI) calcd for $C_{10}H_{21}ClNO_2$ $[M+NH_4]^+$ 222.1255, found 222.1270.

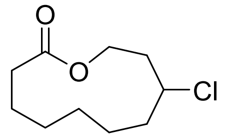

9-Chlorooxacycloundecan-2-one (5l) Colorless oil (23.6 mg, 58%). R_f = 0.3 (petroleum ether/ethyl acetate = 50:1). ^{1}H NMR (400 MHz, $CDCl_3$) δ 4.35 (ddd, *J* = 11.6, 8.0, 1.6 Hz, 1H), 4.24 – 4.14 (m, 2H), 2.41 – 2.34 (m, 1H), 2.34 (t, *J* = 6.0 Hz, 2H), 2.24 – 2.16 (m, 1H), 1.84 – 1.43 (m, 7H), 1.42 – 1.32 (m, 3H). ^{13}C NMR (100 MHz, $CDCl_3$) δ 173.4, 60.9, 60.2, 35.5, 34.2, 34.0, 26.0, 24.2, 21.3, 20.2. IR (neat): υ_{max} (cm^{-1}) 2920, 1756, 1267, 755. HRMS (ESI) calcd for $C_{10}H_{18}ClO_2$ $[M+H]^+$ 205.0990, found 205.1009.

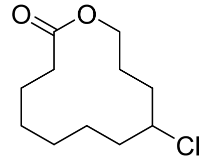

9-Chlorooxacyclododecan-2-one (5m) Colorless oil (15.4 mg, 35%). R_f = 0.3 (petroleum ether/ethyl

acetate = 60:1). ^{1}H NMR (400 MHz, $CDCl_3$) δ 4.57 – 4.52 (m, 1H), 4.29 – 4.23 (m, 1H), 3.96 – 3.90 (m, 1H), 2.46 – 2.32 (m, 2H), 2.09 – 1.99 (m, 2H), 1.97 – 1.74 (m, 4H), 1.67 – 1.56 (m, 3H), 1.50 – 1.28 (m, 5H). ^{13}C NMR (100 MHz, $CDCl_3$) δ 173.7, 64.3, 60.0, 35.2, 33.9, 33.2, 25.0, 24.6, 23.7, 23.6, 22.5. IR (neat): υ_{max} (cm^{-1}) 2913, 1745, 1259, 745. HRMS (ESI) calcd for $C_{11}H_{23}ClNO_2$ $[M+NH_4]^+$ 236.1411, found 236.1421.

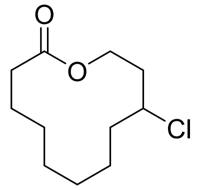

10-Chlorooxacyclododecan-2-one (5n) Colorless oil (26.2 mg, 60%). R_f = 0.3 (petroleum ether/ethyl acetate = 50:1). ^{1}H NMR (400 MHz, $CDCl_3$) δ 4.64 – 4.59 (m, 1H), 4.25 – 4.19 (m, 1H), 4.14 – 4.08 (m, 1H), 2.49 – 2.43 (m, 1H), 2.34 – 2.27 (m, 1H), 2.19 – 2.13 (m, 1H), 2.08 – 1.93 (m, 2H), 1.87 – 1.73 (m, 2H), 1.66 – 1.60 (m, 1H), 1.52 – 1.29 (m, 8H). ^{13}C NMR (100 MHz, $CDCl_3$) δ 173.7, 60.9, 58.9, 35.7, 35.6, 34.2, 24.7, 24.4, 24.2, 23.6, 21.9. IR (neat): υ_{max} (cm^{-1}) 2032, 1727, 1455, 1256, 983. 801. HRMS (ESI) calcd for $C_{11}H_{19}ClO_2K$ $[M+K]^+$ 257.0705, found 257.0703.

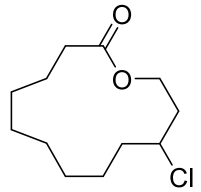

11-Chlorooxacyclotridecan-2-one (5o) Colorless oil (22.8 mg, 49%). R_f = 0.3 (petroleum ether/ethyl acetate = 60:1). ^{1}H NMR (400 MHz, $CDCl_3$) δ 4.35 – 4.28 (m, 2H), 4.08 – 4.01 (m, 1H), 2.45 – 2.38 (m, 1H), 2.33 – 2.26 (m, 1H), 2.23 – 2.15 (m, 1H), 2.07 – 1.99 (m, 1H), 1.93 – 1.74 (m, 2H), 1.70 – 1.64 (m, 2H), 1.60 – 1.54 (m, 1H), 1.42 – 1.29 (m, 9H). ^{13}C NMR (100 MHz, $CDCl_3$) δ 174.0, 61.5, 58.6, 37.5, 36.8, 34.7, 26.8, 26.4, 25.3, 25.1, 24.6, 23.3. IR (neat): υ_{max} (cm^{-1}) 2913, 1745, 1269, 758. HRMS (ESI) calcd for $C_{12}H_{21}ClO_2$ $[M]^+$ 232.1224, found 232.1216.

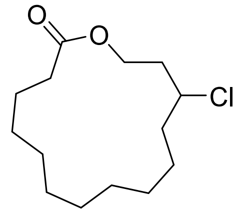

13-Chlorooxacyclopentadecan-2-one (5p) Colorless oil (37.5 mg, 72%). R_f= 0.3 (petroleum ether/ethyl

acetate = 40:1). ^{1}H NMR (400 MHz, $CDCl_3$) δ 4.36 – 4.22 (m, 2H), 4.05 (ddd, *J* = 12.8, 9.6, 4.8 Hz, 1H), 2.41 – 2.30 (m, 2H), 2.17 – 2.09 (m, 1H), 2.05 – 1.97 (m, 1H), 1.84 (ddd, *J* = 14.4, 8.8, 4.8 Hz, 1H), 1.74 (dt, *J* = 10.4, 4.8 Hz, 1H), 1.70 – 1.61 (m, 3H), 1.51 – 1.41 (m, 2H), 1.38 – 1.24 (m, 11H). ^{13}C NMR (100 MHz, $CDCl_3$) δ 174.0, 61.3, 58.8, 38.1, 35.9, 34.0, 28.0, 26.8, 26.6, 25.61, 25.62, 25.4, 24.9, 24.3. IR (neat): υ_{max} (cm^{-1}) 2927, 1733, 1263, 755. HRMS (ESI) calcd for $C_{14}H_{25}ClNO_2K$ $[M+K]^+$ 299.1175, found 299.1181.

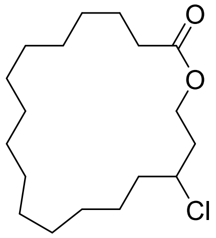

17-Chlorooxacyclononadecan-2-one (5q) Colorless oil (43.6 mg, 69%). R_f = 0.3 (petroleum ether/ethyl acetate = 100:1). ^{1}H NMR (400 MHz, $CDCl_3$) δ 4.33 – 4.21 (m, 2H), 4.07 – 3.99 (m, 1H), 2.33 – 2.29 (m, 2H), 2.15 – 2.08 (m, 1H), 1.99 – 1.90 (m, 1H), 1.79 – 1.74 (m, 2H), 1.66 – 1.63 (m, 2H), 1.50 – 1.44 (m, 2H), 1.35 – 1.27 (m, 20H). ^{13}C NMR (100 MHz, $CDCl_3$) δ 173.8, 61.2, 59.7, 38.1, 37.6, 34.6, 28.5, 28.1, 27.9, 27.7, 27.60, 27.55, 27.4, 27.0, 25.9, 24.9. IR (neat): υ_{max} (cm^{-1}) 2924, 1733, 1451, 1267, 1111, 755. HRMS (ESI) calcd for $C_{18}H_{34}ClO_2$ $[M+H]^+$ 317.2242, found 317.2226.

12. Characterization of Products 6-11

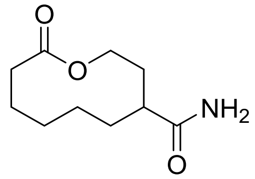

10-Oxooxecane-4-carboxamide (6) Colorless oil (23.9 mg, 60%). R_f = 0.3 (ethyl acetate). ^{1}H NMR (400 MHz, $CDCl_3$) δ 5.65 – 5.68 (br, 2H), 4.45 – 4.34 (m, 2H), 2.46 – 2,39 (m, 2H), 2.31 – 2.24 (m, 1H), 2.10 – 1.98 (m, 2H), 1.95 – 1.84 (m, 2H), 1.78 – 1.69 (m, 2H), 1.59 – 1.45 (m, 2H), 1.44 – 1.33 (m, 1H), 1.30 – 1.21 (m, 1H). ^{13}C NMR (100 MHz, $CDCl_3$) δ 178.3, 173.8, 63.5, 43.2, 34.9, 27.8, 27.1, 26.8, 22.0, 20.7. IR (neat): υ_{max} (cm^{-1}) 2932, 1729, 1453, 1266, 755. HRMS (ESI) calcd for $C_{10}H_{21}N_2O_3$ $[M+NH_4]^+$ 217.1547, found 217.1548.

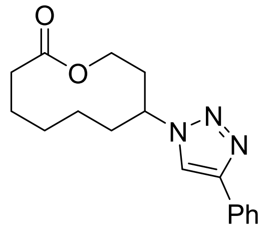

8-(4-Phenyl-1*H*-1,2,3-triazol-1-yl)oxecan-2-one (7) White solid (41.9 mg, 70%). Melting point (°C): 140-141. R_f = 0.3 (petroleum ether/ethyl acetate = 3:1). ^{1}H NMR (400 MHz, $CDCl_3$) δ 7.84 – 7.80 (m, 3H), 7.41 (t, *J* = 7.2 Hz, 2H), 7.32 (tt, *J* = 7.6, 1.2 Hz, 1H), 4.89 – 4.83 (m, 1H), 4.42 (t, *J* = 4.8 Hz, 2H), 2.50 – 2.44 (m, 1H), 2.40 – 2.28 (m, 4H), 2.08 – 1.91 (m, 2H), 1.86 – 1.78 (m, 1H), 1.63 – 1.52 (m, 3H), 1.36 – 1.25 (m, 1H). ^{13}C NMR (100 MHz, $CDCl_3$) δ 173.6, 147.5, 130.7, 128.9, 128.2, 125.7, 118.2, 61.6, 59.6, 34.7, 30.3, 29.8, 27.0, 20.60, 20.55. IR (neat): υ_{max} (cm^{-1}) 2920, 1726, 1432, 1267, 755. HRMS (ESI) calcd for $C_{17}H_{25}N_4O_2$ $[M+NH_4]^+$ 317.1972, found 317.1952.

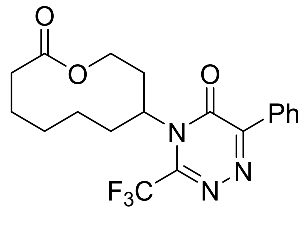

4-(10-Oxooxecan-4-yl)-6-phenyl-3-(trifluoromethyl)-1,2,4-triazin-5(4*H*)-one (8) Colorless oil (49.7 mg, 63%). R_f = 0.3 (petroleum ether/ethyl acetate = 10:1). ^{1}H NMR (400 MHz, $CDCl_3$) δ 8.27 – 8.25 (m, 2H), 7.58 – 7.54 (m, 1H), 7.51 – 7.47 (m, 2H), 4.36 – 4.33 (m, 2H), 4.24 (t, *J* = 8.4 Hz, 1H), 3.10 – 3.00

(m, 1H), 2.67 – 2.59 (m, 1H), 2.54 (dt, J = 15.2, 4.0 Hz, 1H), 2.32 – 2.24 (m, 1H), 1.94 – 1.89 (m, 2H), 1.73 – 1.67 (m, 2H), 1.62 – 1.54 (m, 2H), 1.50 – 1.42 (m, 1H). 1.30 – 1.24 (m, 1H). ^{13}C NMR (100 MHz, $CDCl_3$) δ 173.8, 159.8, 151.8, 142.4 (d, J = 33.7 Hz), 132.0, 131.5, 129.7, 128.5, 118.2 (q, J = 275.7 Hz), 62.4, 60.9, 34.8, 31.8, 31.2, 25.6, 25.0, 21.2. ^{19}F NMR (376 MHz, $CDCl_3$) δ -63.96. IR (neat): υ_{max} (cm^{-1}) 2934, 2345, 1767, 1267, 755. HRMS (ESI) calcd for $C_{19}H_{24}F_3N_4O_3$ $[M+NH_4]^+$ 413.1795, found 413.1785.

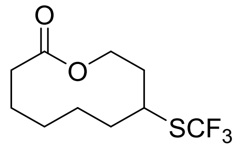

8-((Trifluoromethyl)thio)oxecan-2-one (9) Colorless oil (34.4 mg, 67%). R_f = 0.3 (petroleum ether/ethyl acetate = 50:1). ^1H NMR (400 MHz, $CDCl_3$) δ 4.44 – 4.32 (m, 2H), 3.58 – 3.52 (m, 1H), 2.43 – 2.24 (m, 3H), 2.08 – 1.91 (m, 2H), 1.88 – 1.72 (m, 3H), 1.58 – 1.47 (m, 2H), 1.36 – 1.28 (m, 2H). ^{13}C NMR (100 MHz, $CDCl_3$) δ 173.5, 132.6 (q, J = 304.6 Hz), 62.1, 43.8, 34.7, 30.5, 29.8, 27.0, 20.8, 20.5. ^{19}F NMR (376 MHz, $CDCl_3$) δ -39.56. IR (neat): υ_{max} (cm^{-1}) 2926, 1731, 1454, 1267, 1109, 755. HRMS (ESI) calcd for $C_{10}H_{15}F_3O_2SLi$ $[M+Li]^+$ 263.0899, found 263.0878.

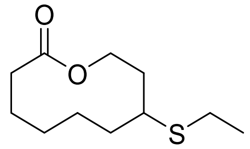

8-(Ethylthio)oxecan-2-one (10) Colorless oil (30.3 mg, 70%). R_f = 0.3 (petroleum ether/ethyl acetate = 50:1). ^1H NMR (400 MHz, $CDCl_3$) δ 4.45 – 4.39 (m, 1H), 4.35 – 4.30 (m, 1H), 2.98 – 2.92 (m, 1H), 2.51 (q, J = 7.2 Hz, 2H), 2.39 (ddd, J = 16.4, 8.0, 3.2 Hz, 1H), 2.27 (ddd, J = 16.0, 10.4, 2.8 Hz, 1H), 2.19 – 2.12 (m, 1H), 1.88 – 1.68 (m, 5H), 1.53 – 1.46 (m, 2H), 1.34 – 1.26 (m, 2H), 1.23 (t, J = 7.2 Hz, 3H). ^{13}C NMR (100 MHz, $CDCl_3$) δ 174.1, 81.7, 59.3, 40.8, 36.4, 31.2, 28.9, 27.0, 26.6, 21.3, 18.4. IR (neat): υ_{max} (cm^{-1}) 2932, 1728, 1453, 1265, 755. HRMS (ESI) calcd for $C_{11}H_{20}O_2SLi$ $[M+Li]^+$ 223.1339, found 223.1323.

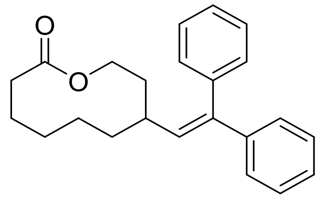

8-(2,2-Diphenylvinyl)oxecan-2-one (11) Colorless oil (6.7 mg, 10%). R_f = 0.3 (petroleum ether/ethyl acetate = 20:1). ^{1}H NMR (400 MHz, $CDCl_3$) δ 7.40 – 7.28 (m, 4H), 7.25 – 7.19 (m, 4H), 7.17 – 7.15 (m, 2H), 6.12 (d, *J* = 10.4 Hz, 1H), 4.44 – 4.39 (m, 1H), 4.21 – 4.15 (m, 1H), 2.55 – 2.48 (m, 1H), 2.40 – 2.31 (m, 2H), 1.90 – 1.73 (m, 4H), 1.67 – 1.42 (m, 4H), 1.34 – 1.27 (m, 2H). ^{13}C NMR (100 MHz, $CDCl_3$) δ 173.9, 142.6, 140.3, 140.2, 133.5, 129.7, 128.4, 128.2, 127.2, 127.1, 63.6, 36.9, 35.0, 30.5, 29.8, 27.5, 20.8, 20.7. IR (neat): υ_{max} (cm^{-1}) 2946, 1727, 1701, 1450, 1235, 1029, 764, 699. HRMS (ESI) calcd for $C_{23}H_{27}O_2$ $[M+H]^+$ 335.2006, found 335.1991.

13. References

1. (a) G. H. Posner, J. P. Maxwell and M. Kahraman, *J. Org. Chem*., 2003, **68**, 3049; (b) J. Du, X. Yang, X. Wang, Q. An, X. He, H. Pan and Z. Zuo, *Angew. Chem., Int. Ed.,* 2021*,* **60***,* 5370*.*
2. N. Hanaki, K. Ishihara, M. Kaino, Y. Naruse and H. Yamamoto, *Tetrahedron,* 1996, **52**, 7297.
3. S. Liu, M. Bai, P.-F. Xu, Q.-X. Sun, X.-H. Duan and L.-N. Guo, *Chem. Commun.,* 2021, **57**, 8652.
4. P. Ḿarce, J. Lynch, A. J. Blacker and J. M. J. Williams, *Chem. Commun*., 2016, **52**, 1436.
5. Y.-L. Su, L. Tram, D. Wherritt, H. Arman, W. P. Griffith and M. P. Doyle, *ACS Catal*., 2020, **10**, 13682.
6. W. Wu, J. Wang, Y. Wang, Y. Huang, Y. Tan and Z. Weng, *Angew. Chem., Int. Ed.,* 2017, **56**, 10476.
7. K. Zhang, T. Liang, Y. Wang, C. He, Y. Hu, X.- H. Duan and L. Liu, *Org. Chem. Front*., 2022, **9***,* 966.
8. M. J. Frisch, G. W. Trucks, H. B. Schlegel, G.E. Scuseria, M. A. Robb, J. R. Cheeseman, G. Scalmani, V. Barone, G. A. Petersson, H. Nakatsuji, X. Li, M. Caricato, A. V. Marenich, J. Bloino, B. G. Janesko, R. Gomperts, B. Mennucci, H. P. Hratchian, J. V. Ortiz, A. F. Izmaylov, J. L. Sonnenberg, D. Williams-Young, F. Ding, F. Lipparini, F. Egidi, J. Goings, B. Peng, A. Petrone, T. Henderson, D. Ranasinghe, V. G. Zakrzewski, J. Gao, N. Rega, G. Zheng, W. Liang, M. Hada, M. Ehara, K. Toyota, R. Fukuda, J. Hasegawa, M. Ishida, T. Nakajima, Y. Honda, O. Kitao, H. Nakai, T. Vreven, K. Throssell, J. A. Montgomery, J. E. Jr. Peralta, F. Ogliaro, M. J. Bearpark, J. J. Heyd, E. N. Brothers, K. N. Kudin, V. N. Staroverov, T. A. Keith, R. Kobayashi, J. Normand, K. Raghavachari, A. P. Rendell, J. C. Burant, S. S. Iyengar, J. Tomasi, M. Cossi, J. M. Millam, M. Klene, C. Adamo, R. Cammi, J. W. Ochterski, R. L. Martin, K. Morokuma, O. Farkas, J. B. Foresman, D. J. Fox, *Gaussian 16, Revision A.03*; Gaussian, Inc.: Wallingford, CT, 2016.
9. (a) A. D. Becke, *J. Chem. Phys.,* 1993, **98**, 5648; (b) K. Raghavachari, *Theor. Chem. Acc.,* 2000, **103**, 361; (c) A. D. Becke, *J. Chem. Phys.,* 1993, **98**, 1372; (d) C. Lee, W. Yang and R. G. Parr, *Phys. Rev. B.,* 1988, **37**, 785.
10. S. Grimme, S. Ehrlich and L. Goerigk, *J. Comput. Chem.,* 2011, **32,** 1456.
11. D. Andrae, U. Häußermann, M. Dolg, H. Stoll and H. Preuß, *Theor. Chim. Acta.,* 1990, **77**, 123.
12. (a) R. Ditchfield, W. J. Hehre and J. A. Pople, *J. Chem. Phys.,* 1971, **54**, 724; (b) W. J. Hehre, R. Ditchfield and J. A. Pople, *J. Chem. Phys.,* 1972, **56**, 2257.
13. F. Weigend and R. Ahlrichs, *Phys. Chem. Chem. Phys.,* 2005, **7,** 3297.
14. A. V. Marenich, C. J. Cramer and D. G. Truhlar, *J. Phys. Chem., B* 2009, **113**, 6378.
15. (a) K. Fukui, *J. Phys. Chem.* 1970, **74,** 4161; (b) K. Fukui, *Acc. Chem. Res.,* 1981, **14**, 363.
16. CYLview20; Legault, C. Y., Université de Sherbrooke, 2020 (http://www.cylview.org).

14. ^{1}H NMR and ^{13}C NMR Spectra of Hemiketal Hydroperoxides 1

^{1}H NMR (400 MHz, $CDCl_3$) and ^{13}C NMR (100 MHz, $CDCl_3$) spectra of product **1a**

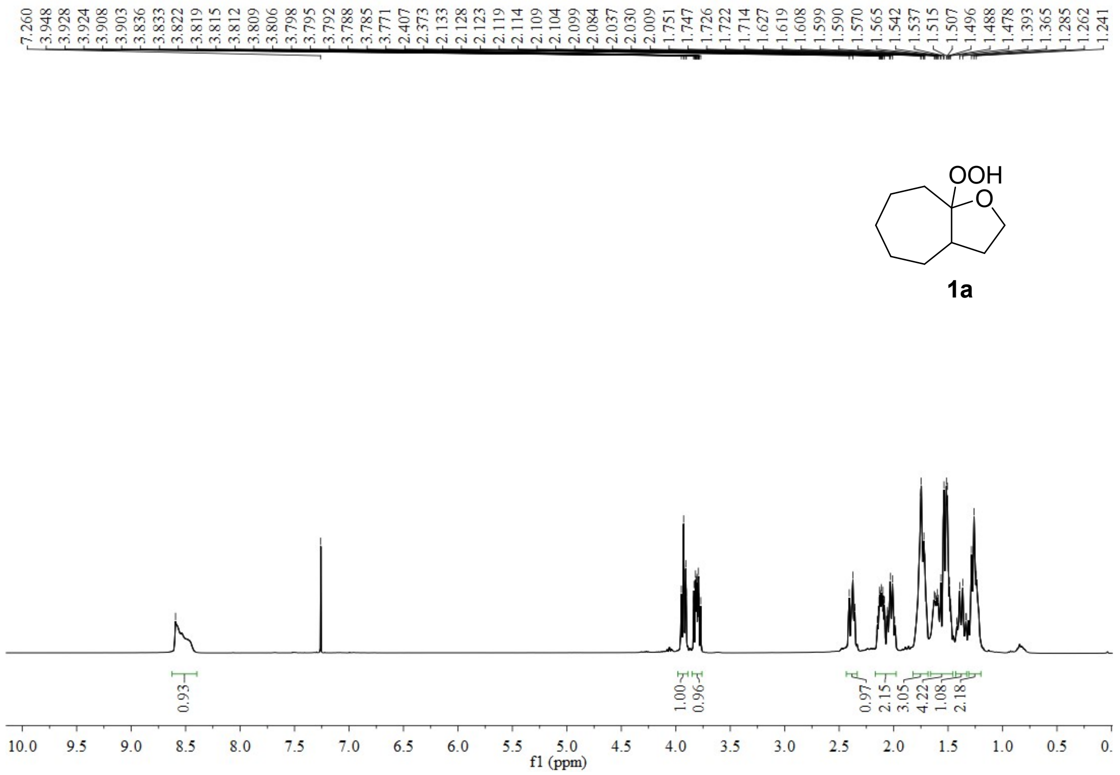

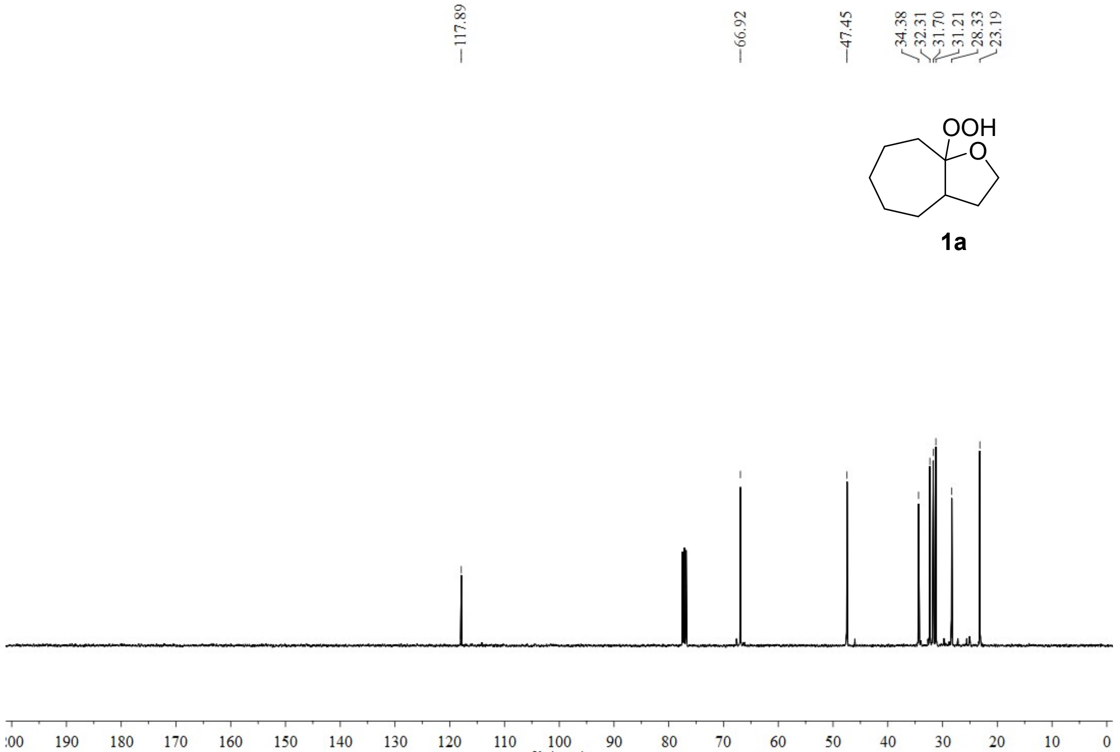

^{1}H NMR (400 MHz, $CDCl_3$) and ^{13}C NMR (100 MHz, $CDCl_3$) spectra of product **1b**

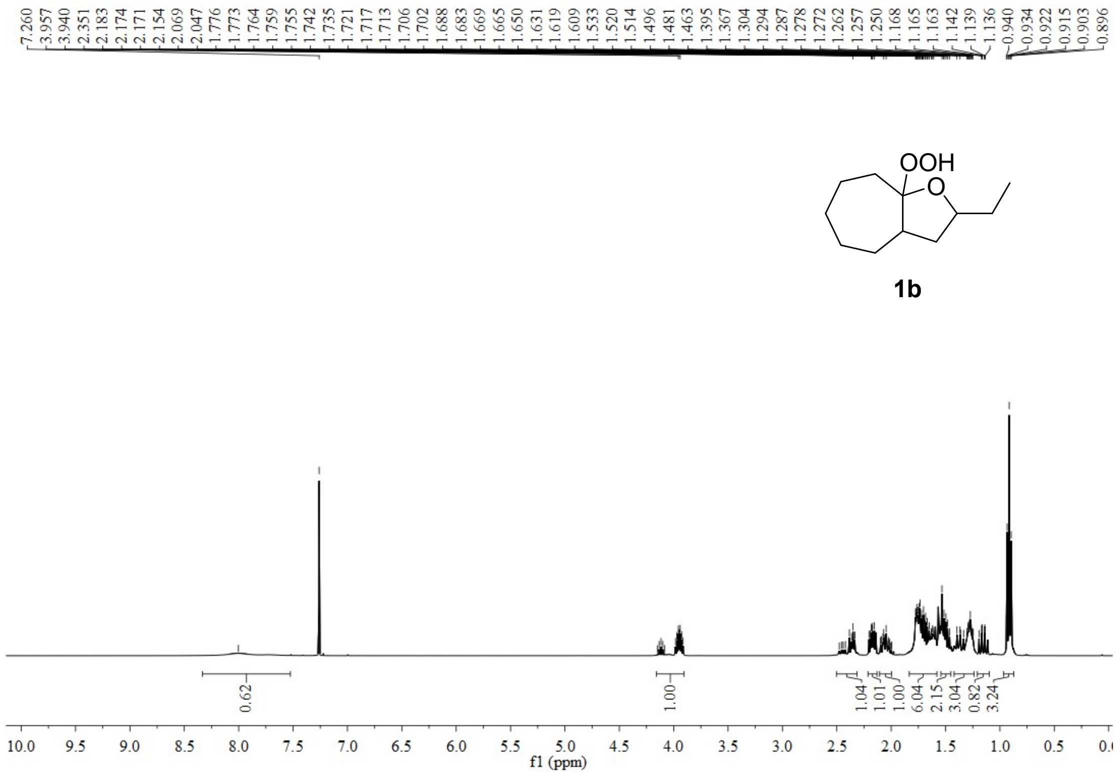

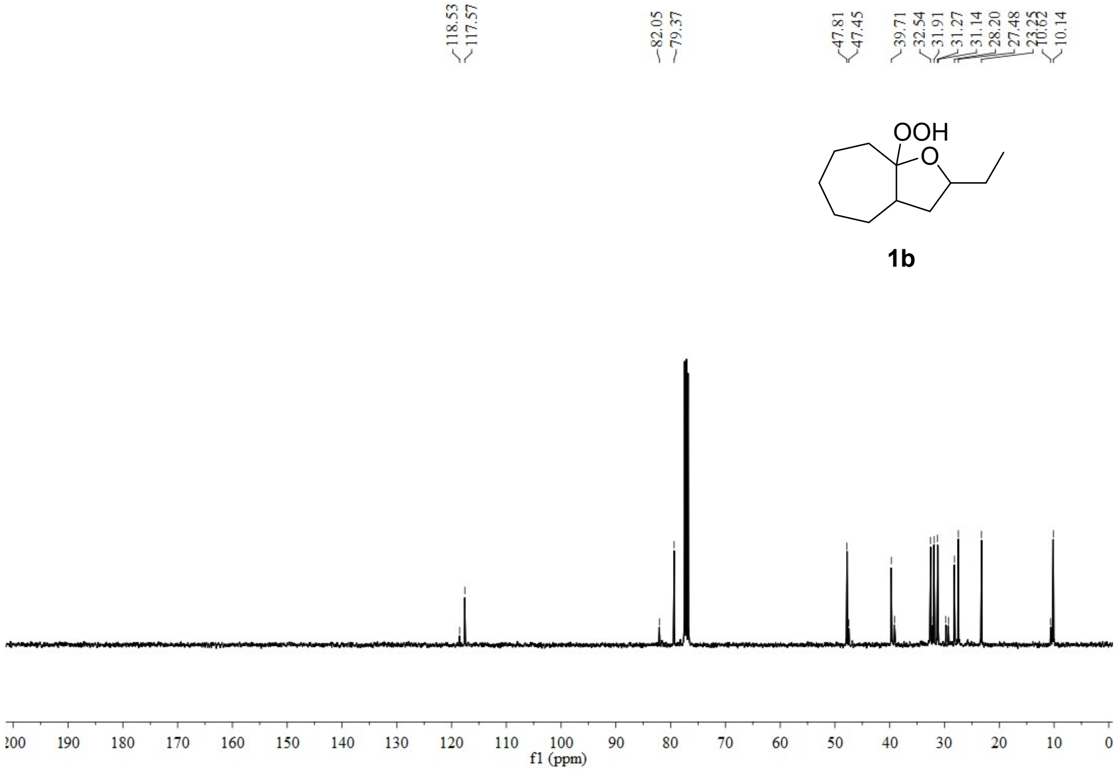

^1H NMR (400 MHz, $CDCl_3$) and ^{13}C NMR (100 MHz, $CDCl_3$) spectra of product **1c**

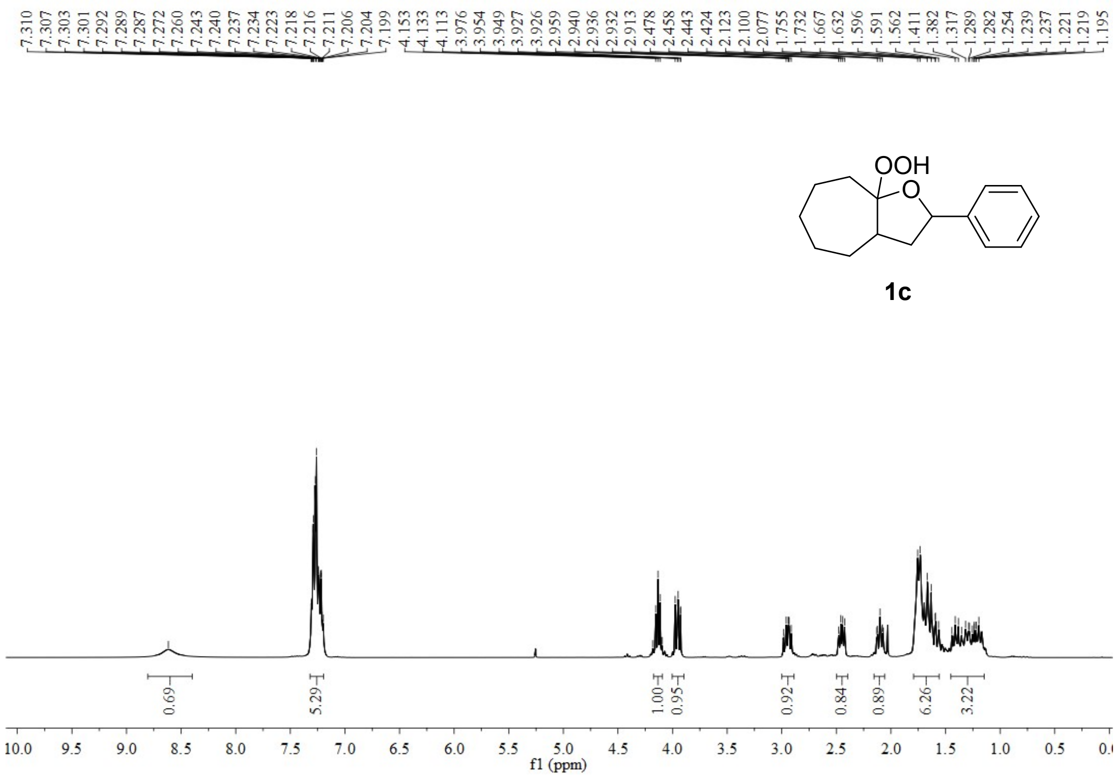

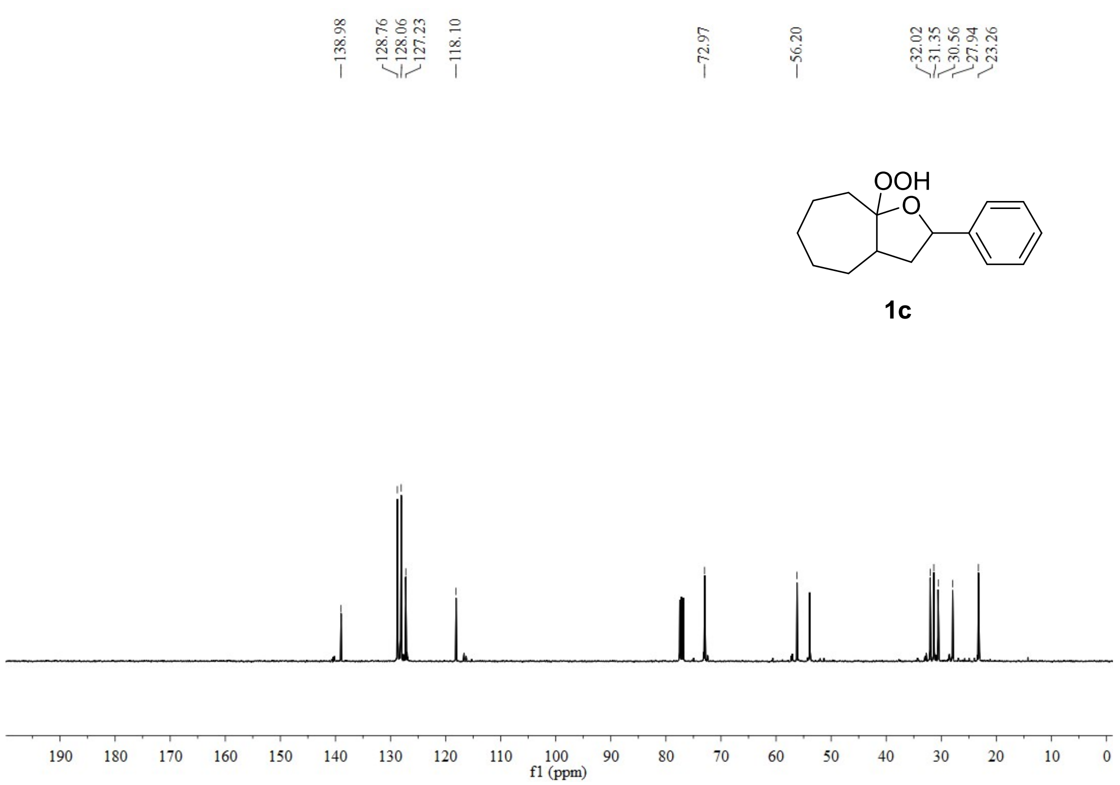

^{1}H NMR (400 MHz, $CDCl_3$) and ^{13}C NMR (100 MHz, $CDCl_3$) spectra of product **1d**

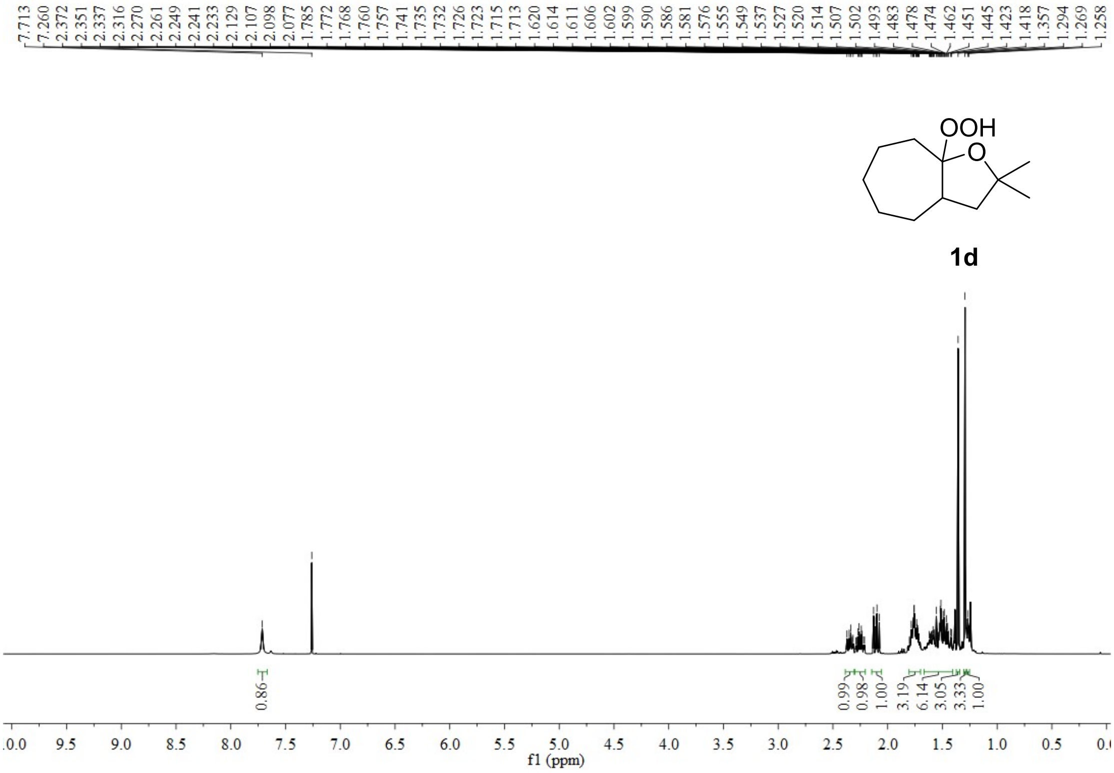

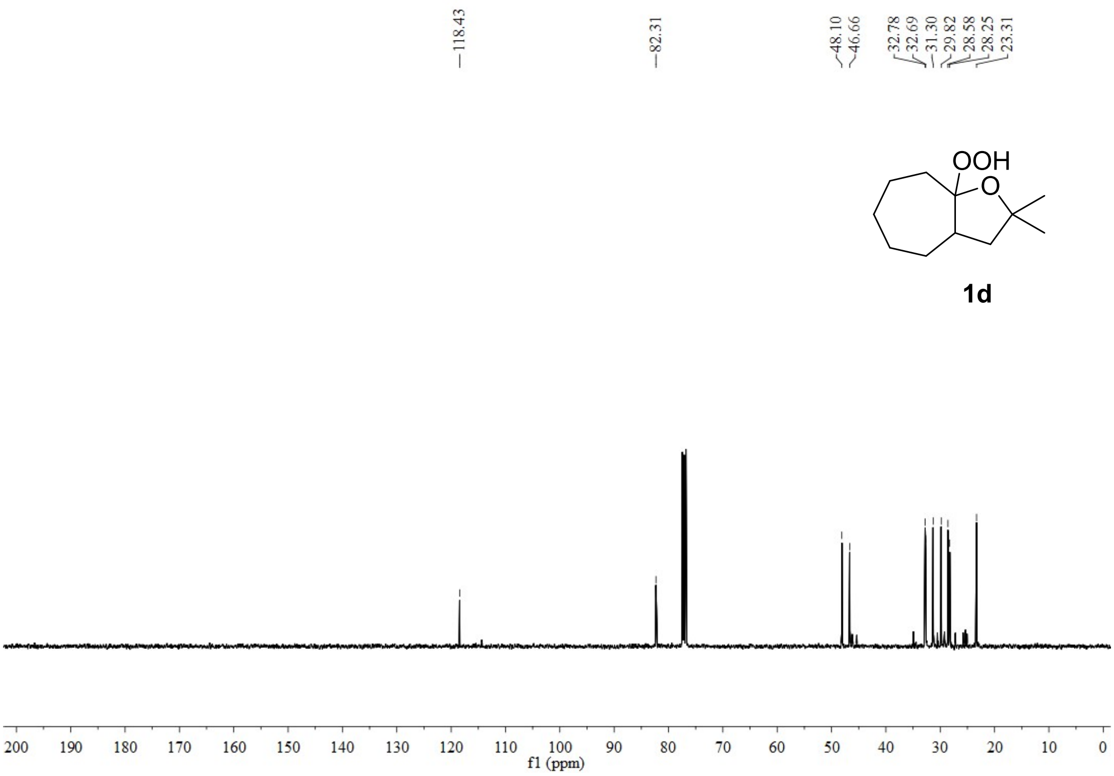

^{1}H NMR (400 MHz, $CDCl_3$) and ^{13}C NMR (100 MHz, $CDCl_3$) spectra of product **1e**

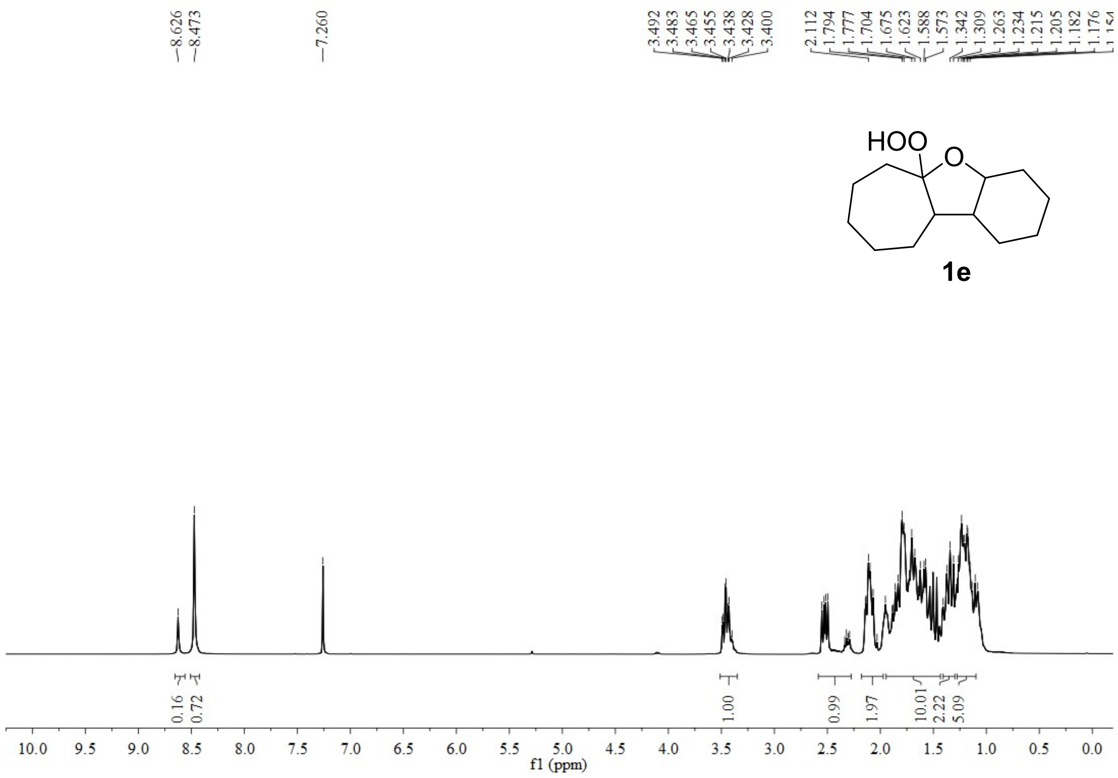

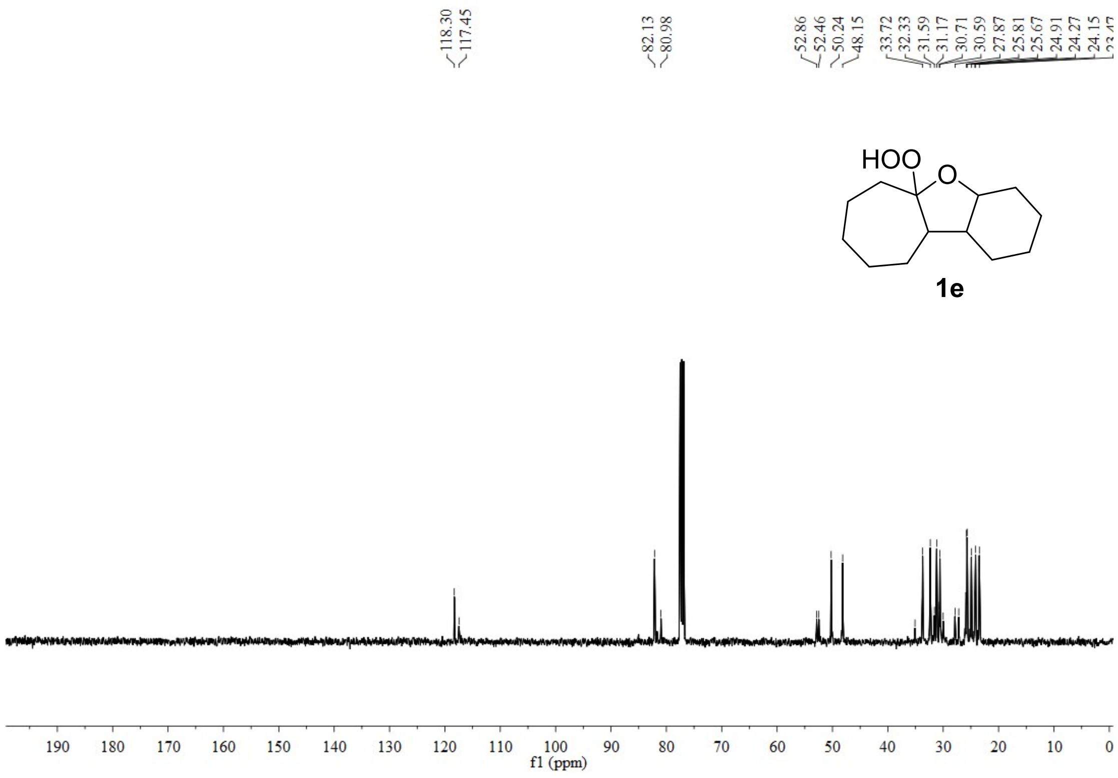

^{1}H NMR (400 MHz, $CDCl_3$) and ^{13}C NMR (100 MHz, $CDCl_3$) spectra of product **1f**

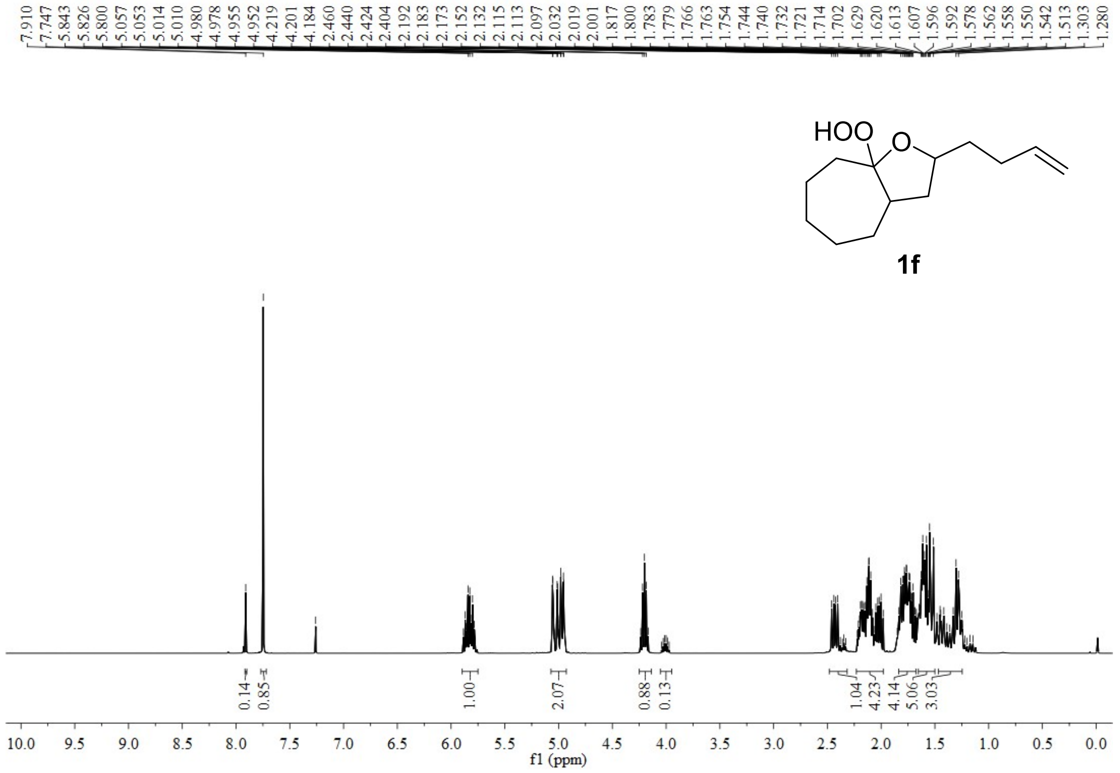

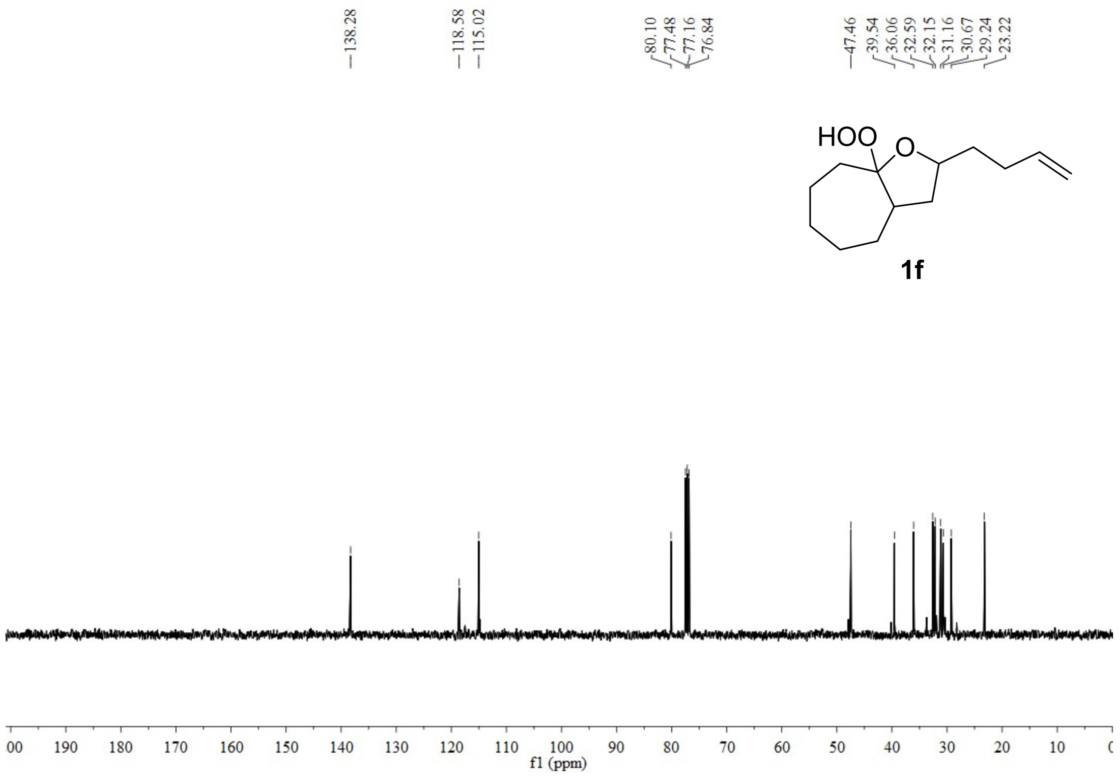

^{1}H NMR (400 MHz, $CDCl_3$) and ^{13}C NMR (100 MHz, $CDCl_3$) spectra of product **1g**

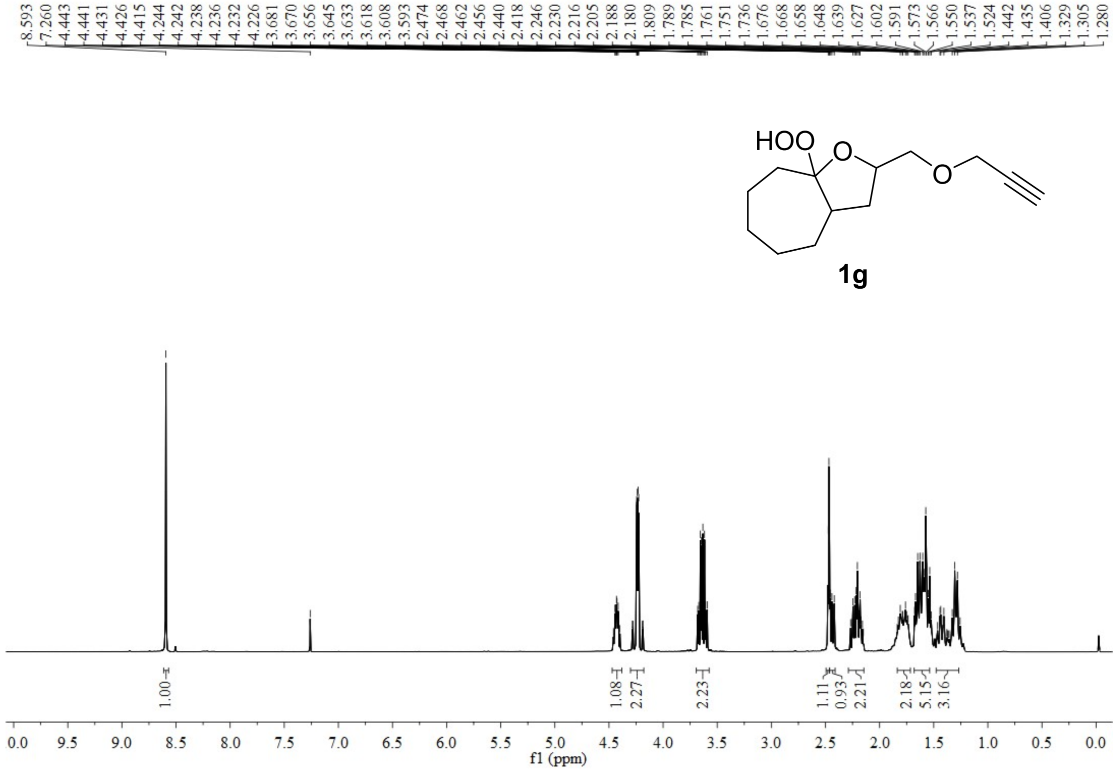

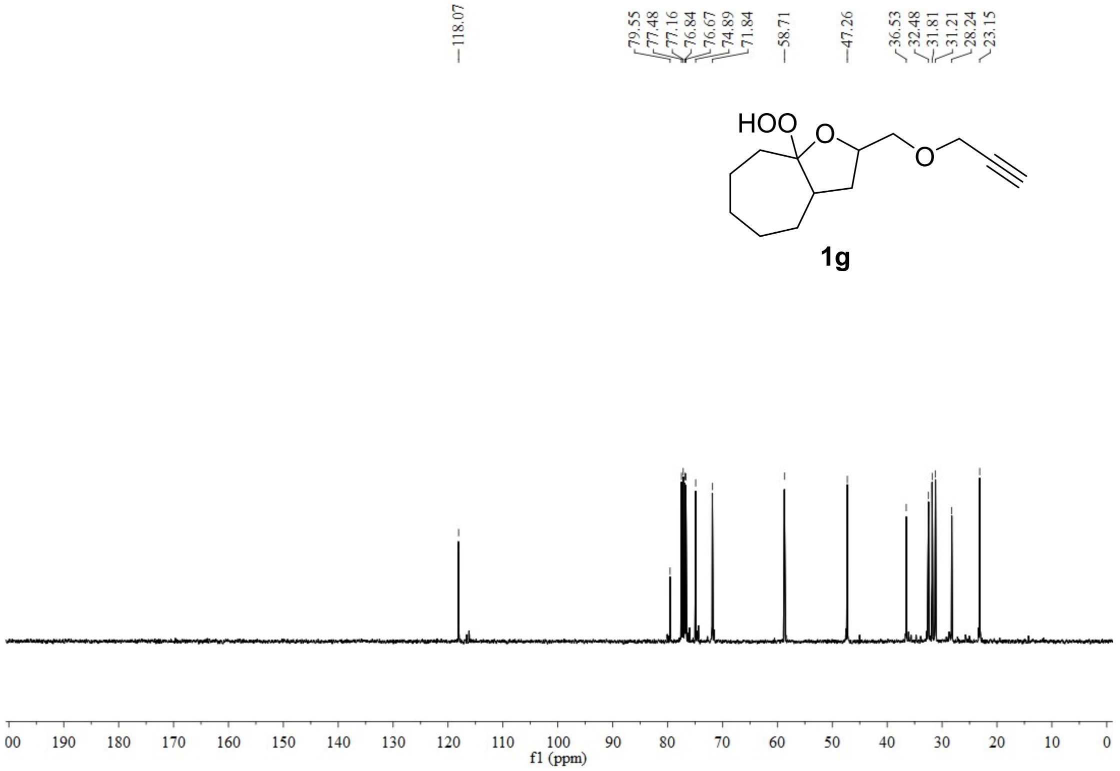

^{1}H NMR (400 MHz, $CDCl_3$) and ^{13}C NMR (100 MHz, $CDCl_3$) spectra of product **1h**

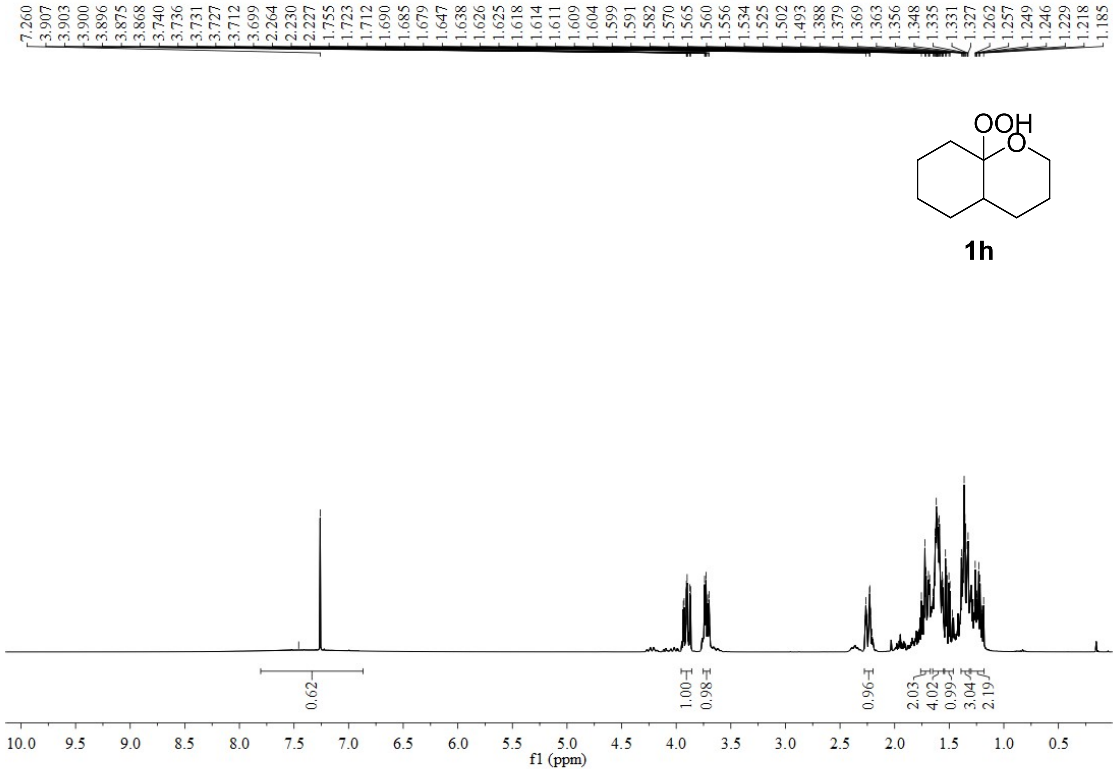

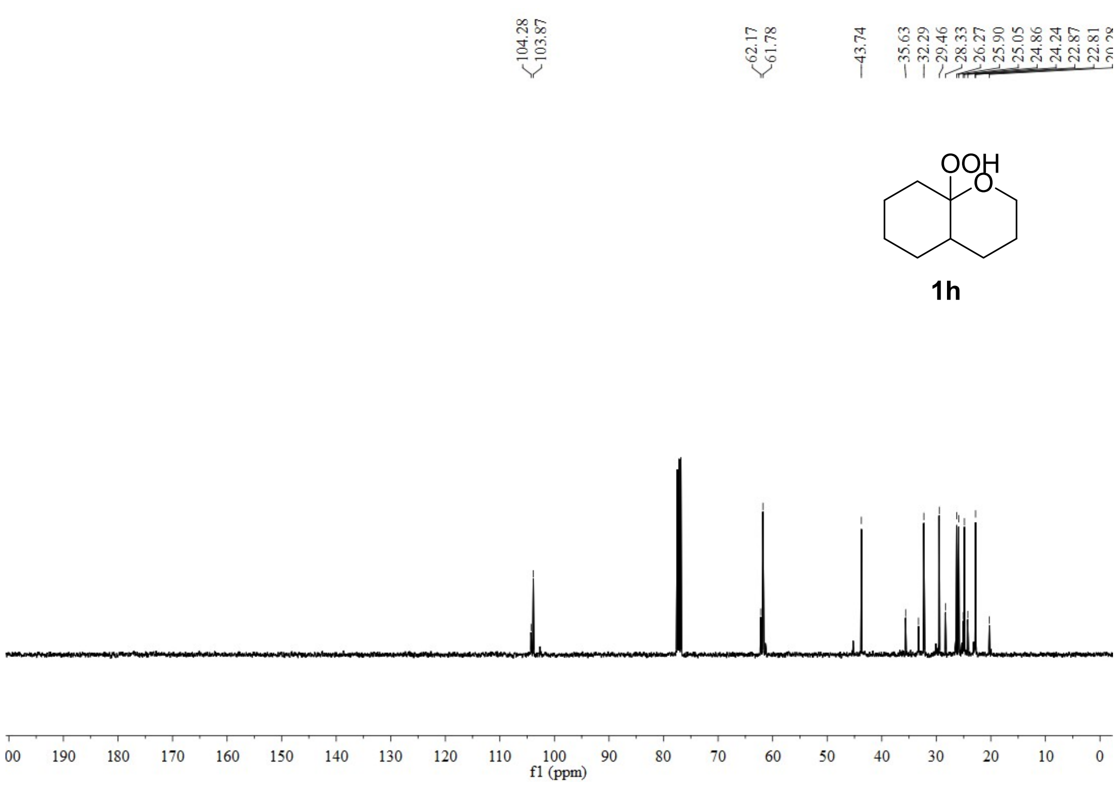

^{1}H NMR (400 MHz, $CDCl_3$) and ^{13}C NMR (100 MHz, $CDCl_3$) spectra of product **1i**

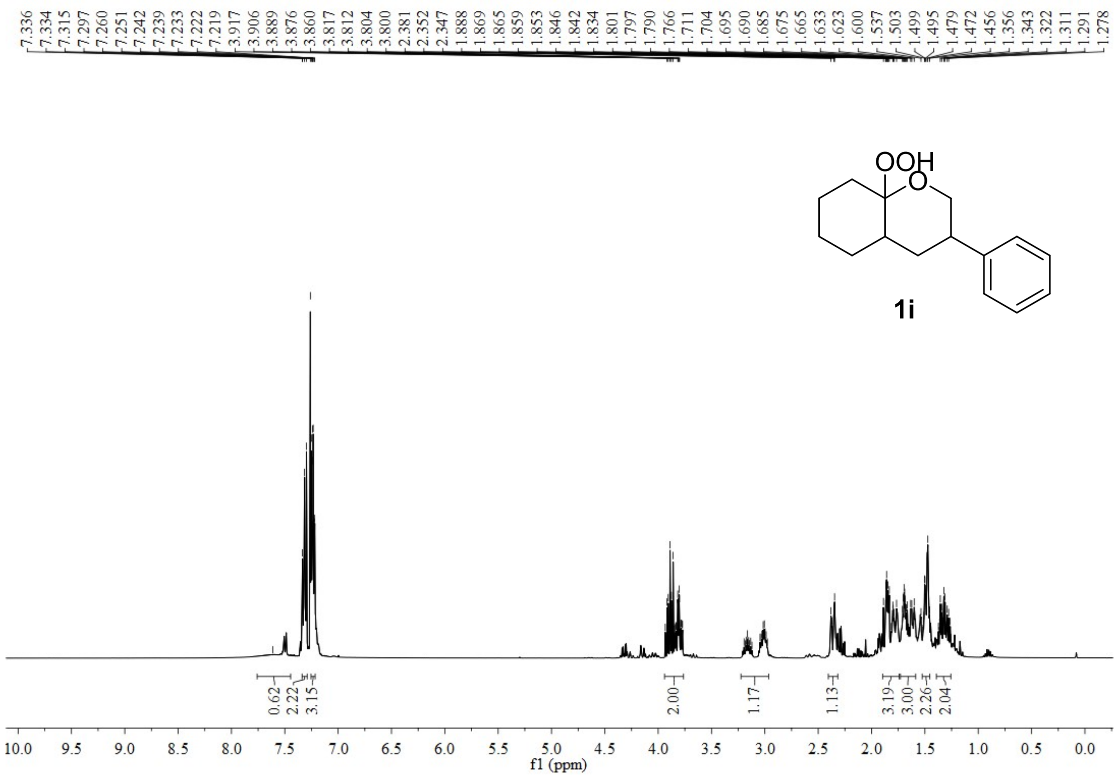

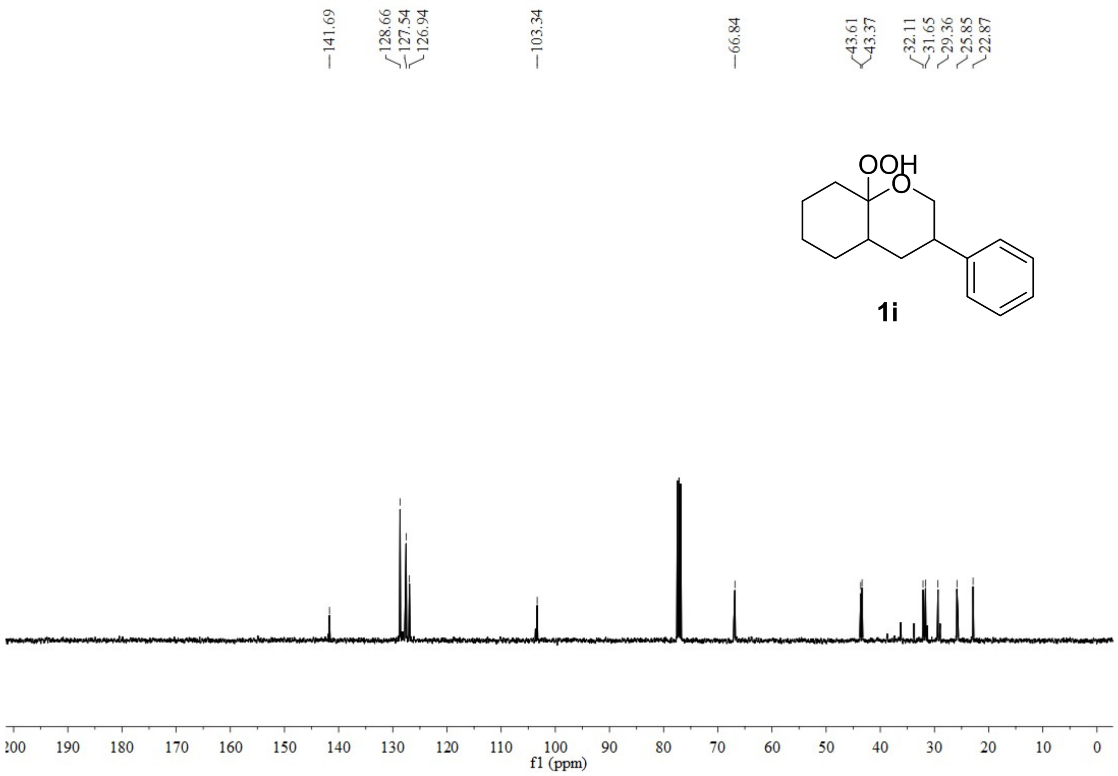

^{1}H NMR (400 MHz, $CDCl_3$) and ^{13}C NMR (100 MHz, $CDCl_3$) spectra of product **1j**

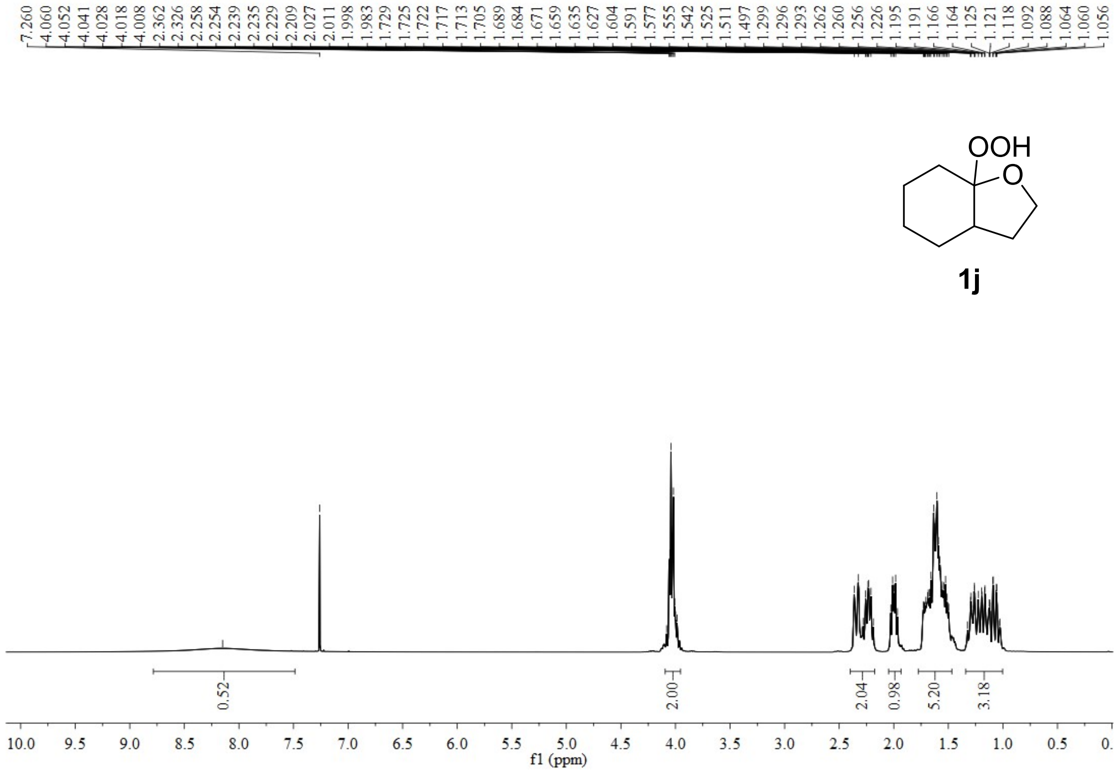

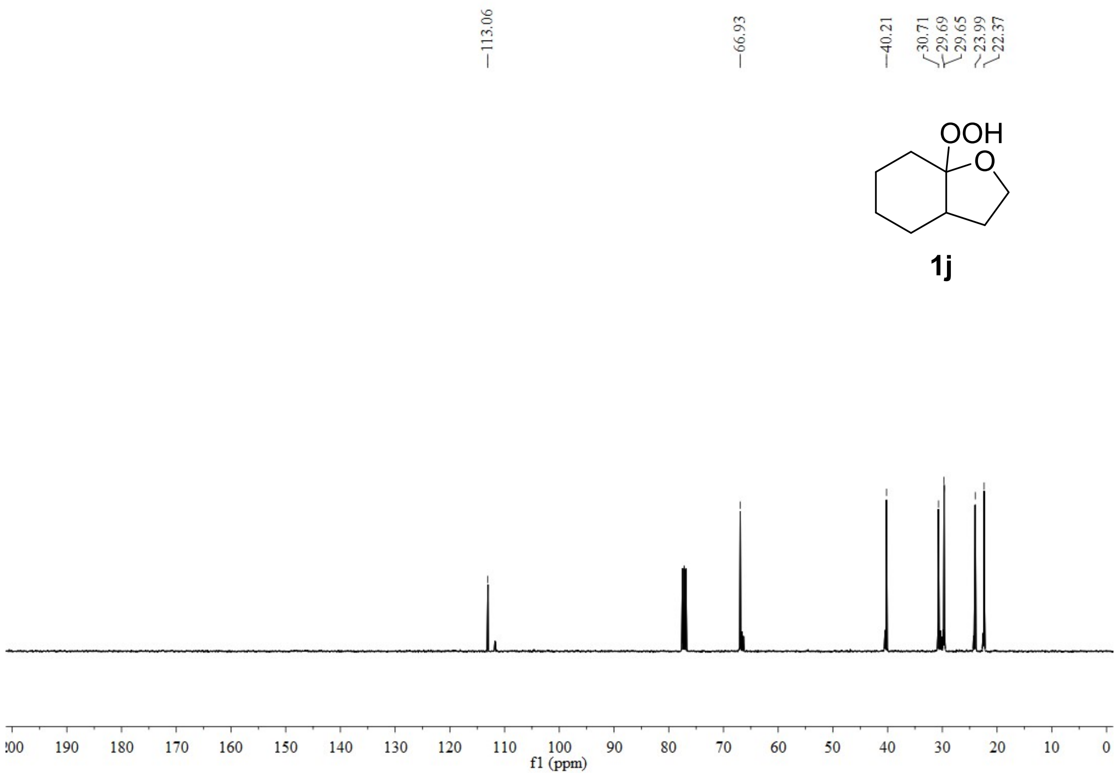

^{1}H NMR (400 MHz, $CDCl_3$) and ^{13}C NMR (100 MHz, $CDCl_3$) spectra of product **1k**

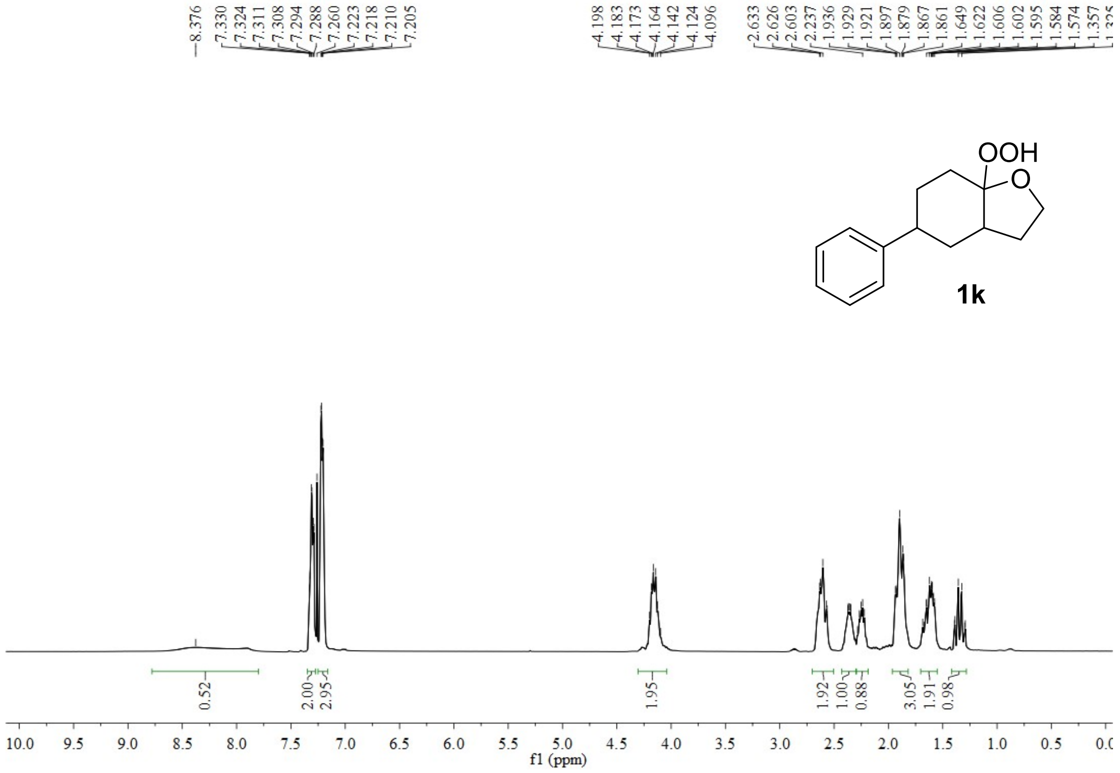

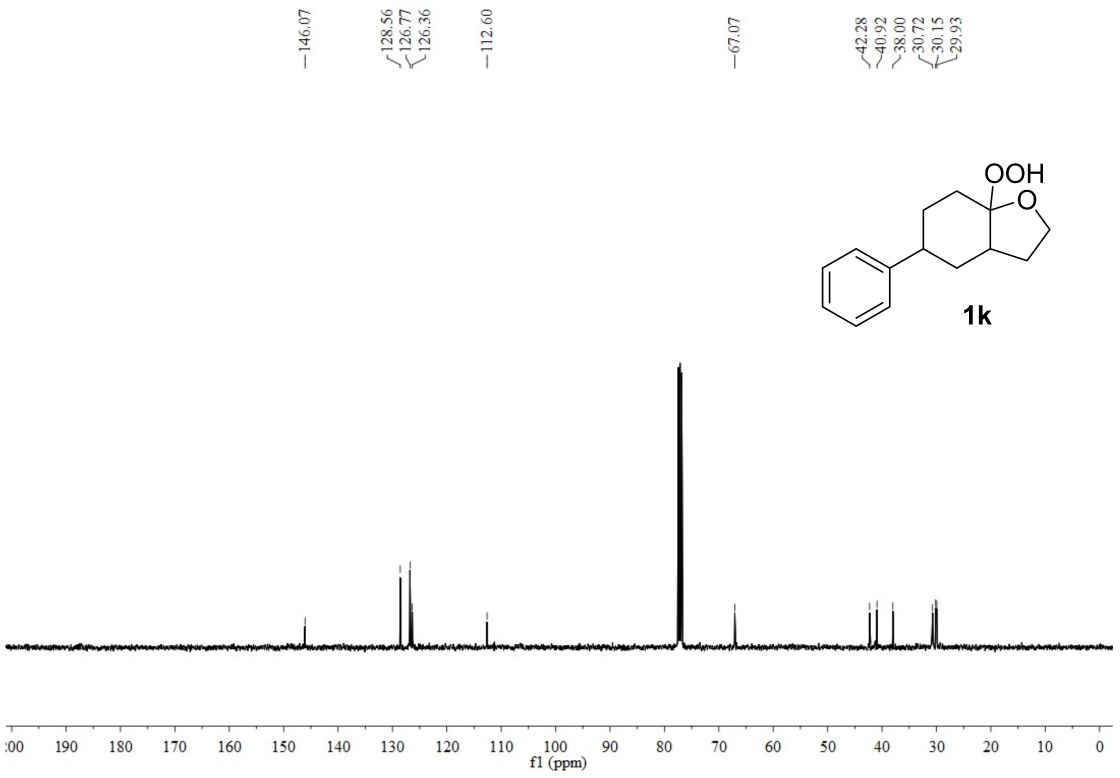

^{1}H NMR (400 MHz, $CDCl_3$) and ^{13}C NMR (100 MHz, $CDCl_3$) spectra of product **1l**

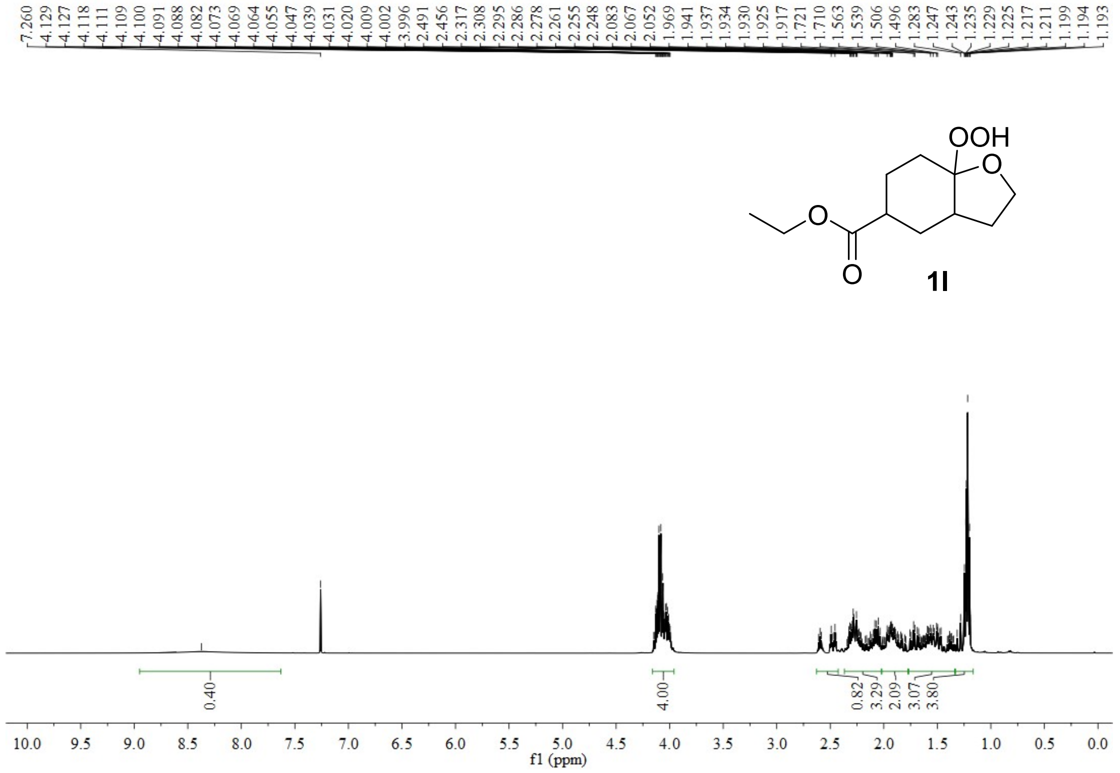

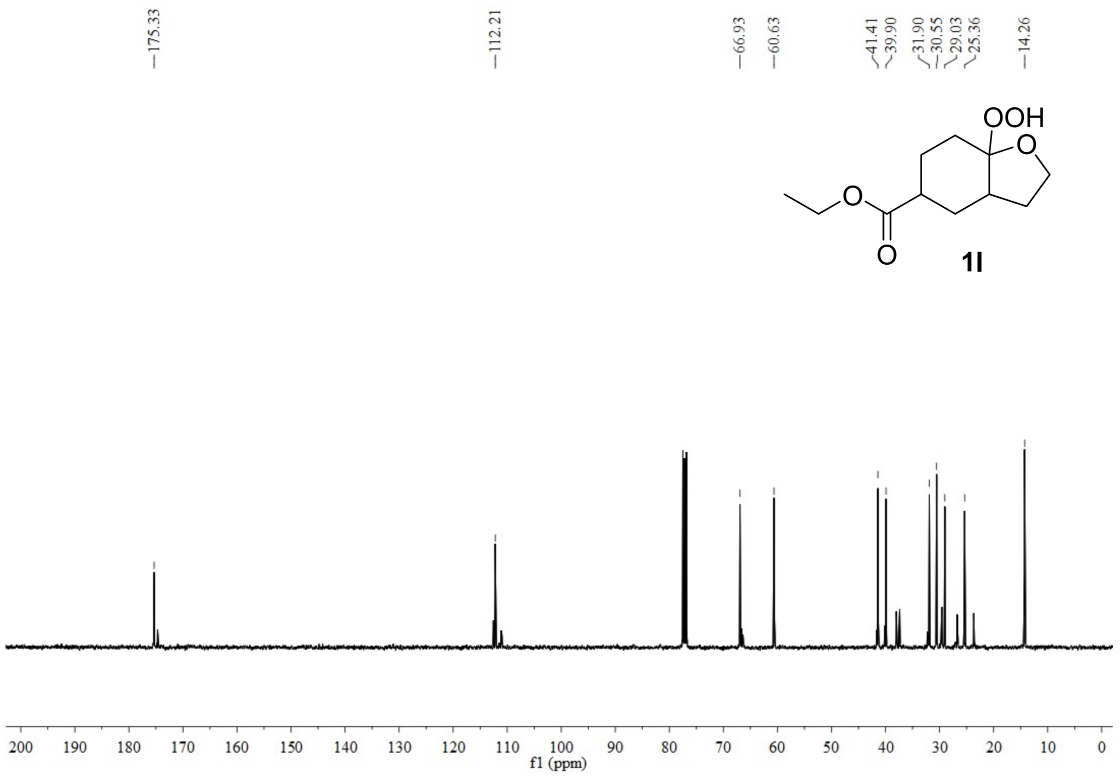

^{1}H NMR (400 MHz, $CDCl_3$) and ^{13}C NMR (100 MHz, $CDCl_3$) spectra of product **1m**

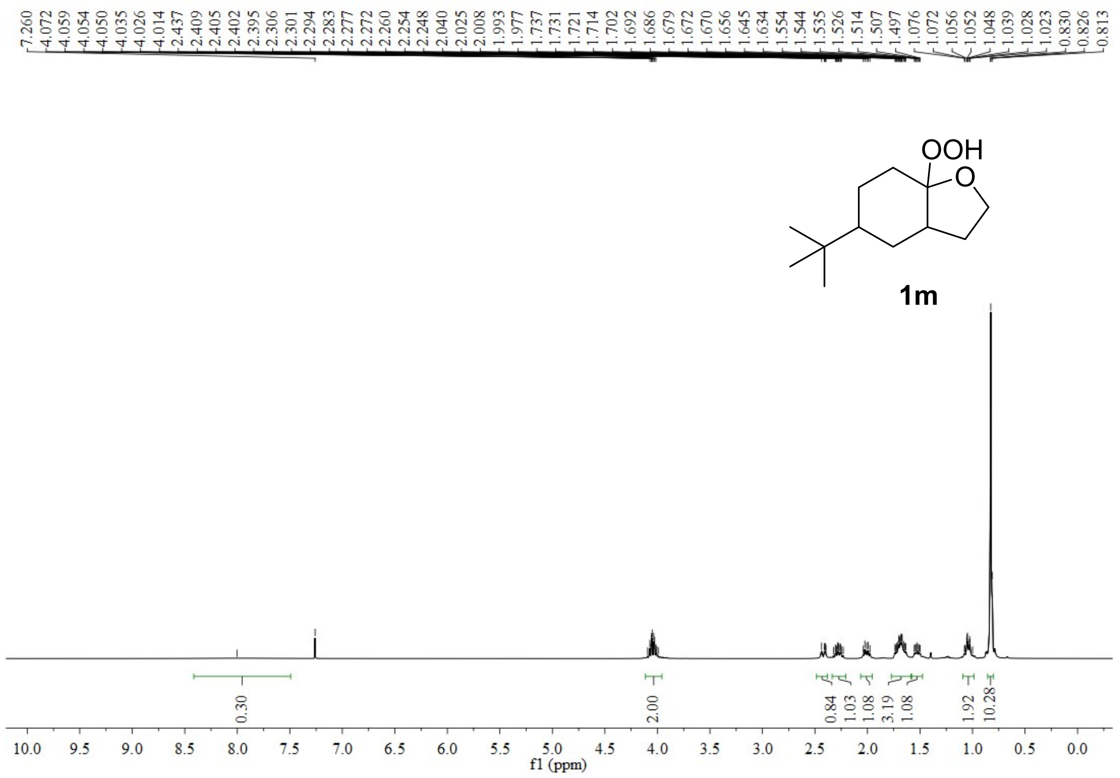

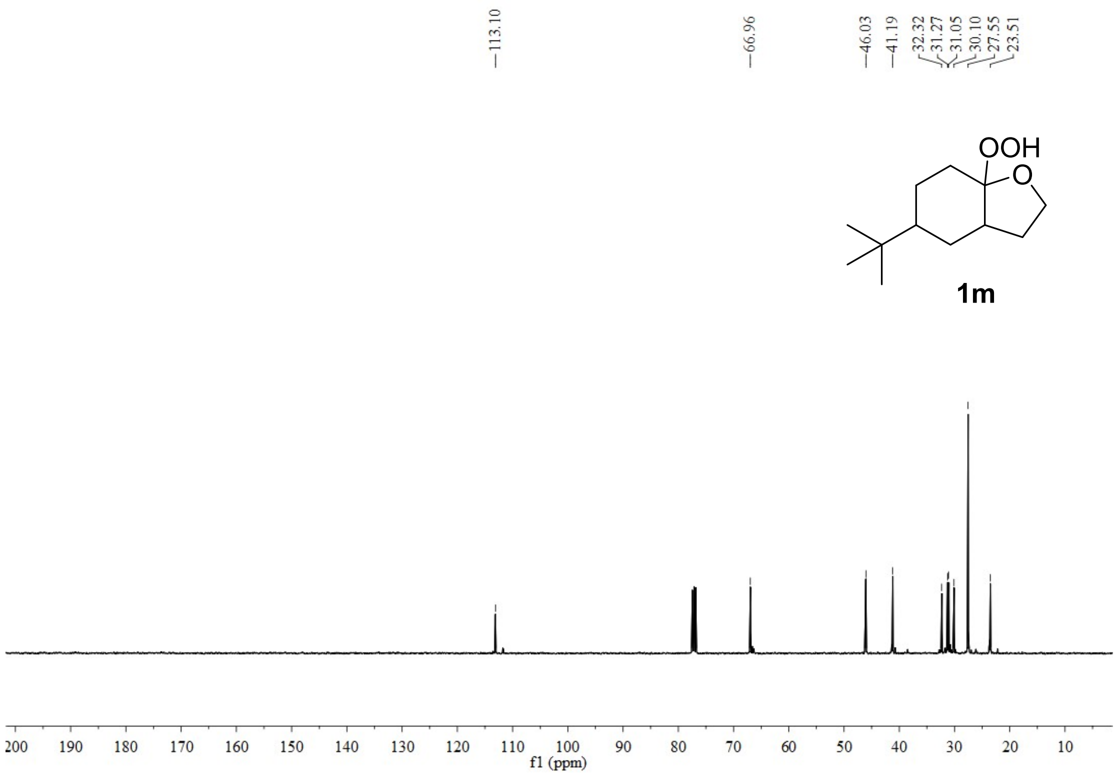

^{1}H NMR (400 MHz, $CDCl_3$) and ^{13}C NMR (100 MHz, $CDCl_3$) spectra of product **1n**

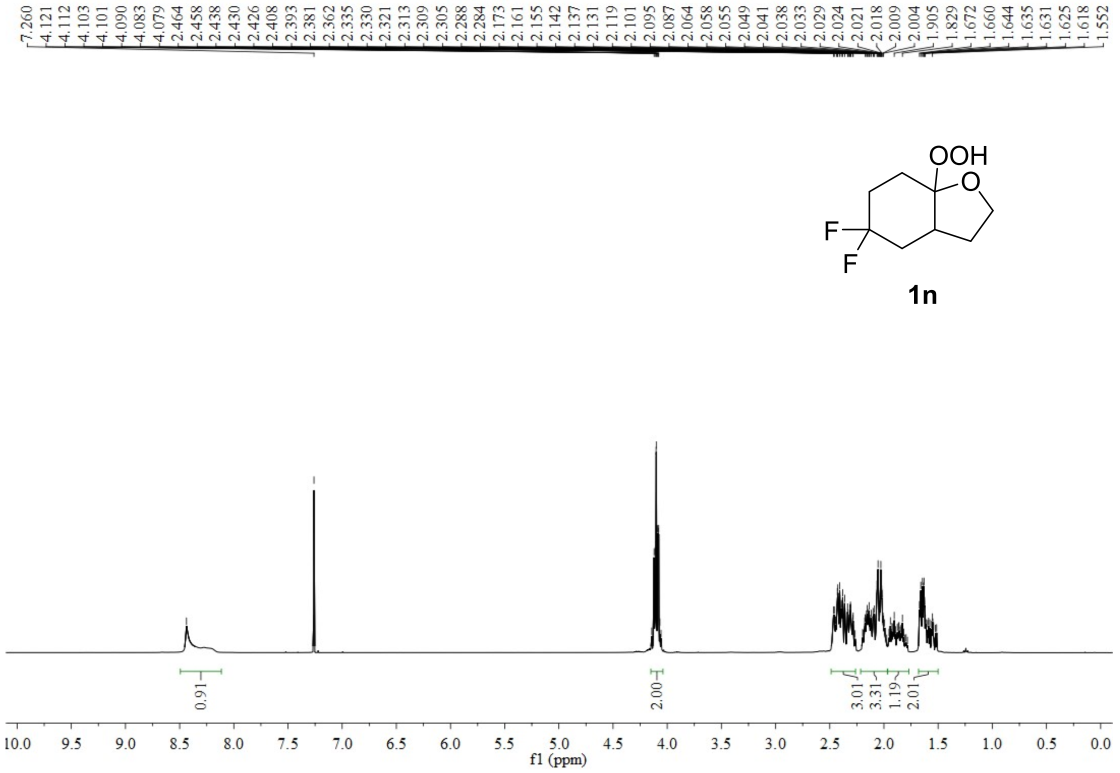

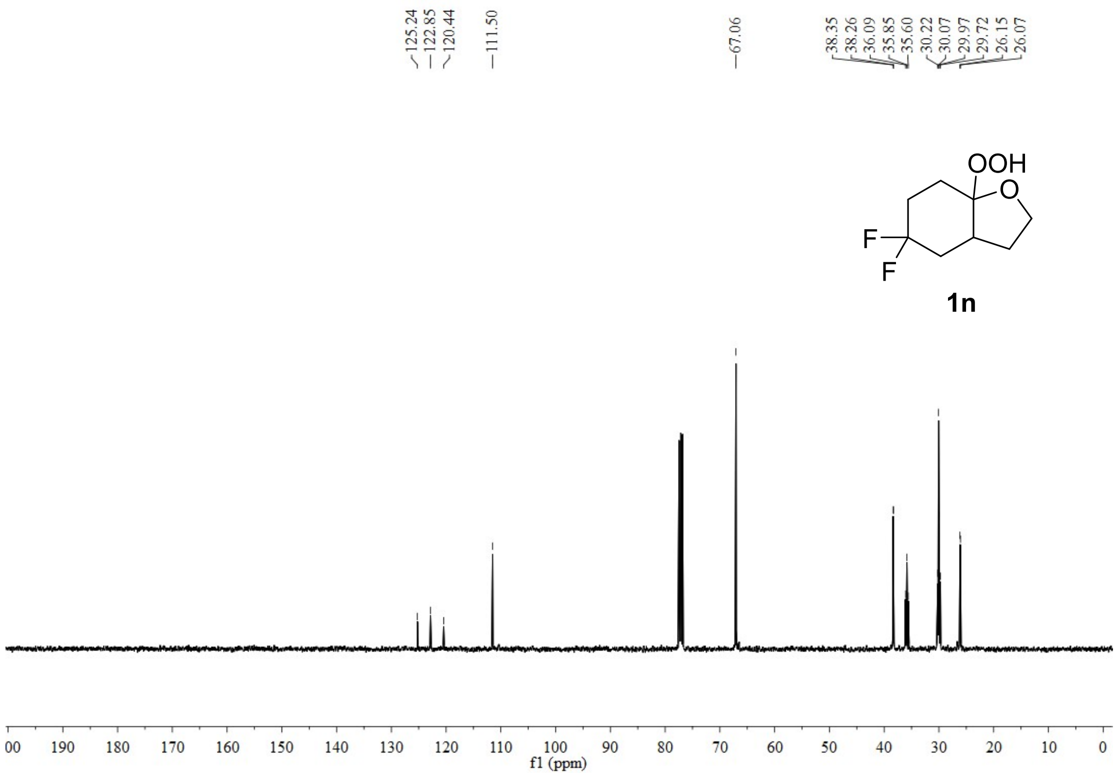

^{1}H NMR (400 MHz, $CDCl_3$) and ^{13}C NMR (100 MHz, $CDCl_3$) spectra of product **1o**

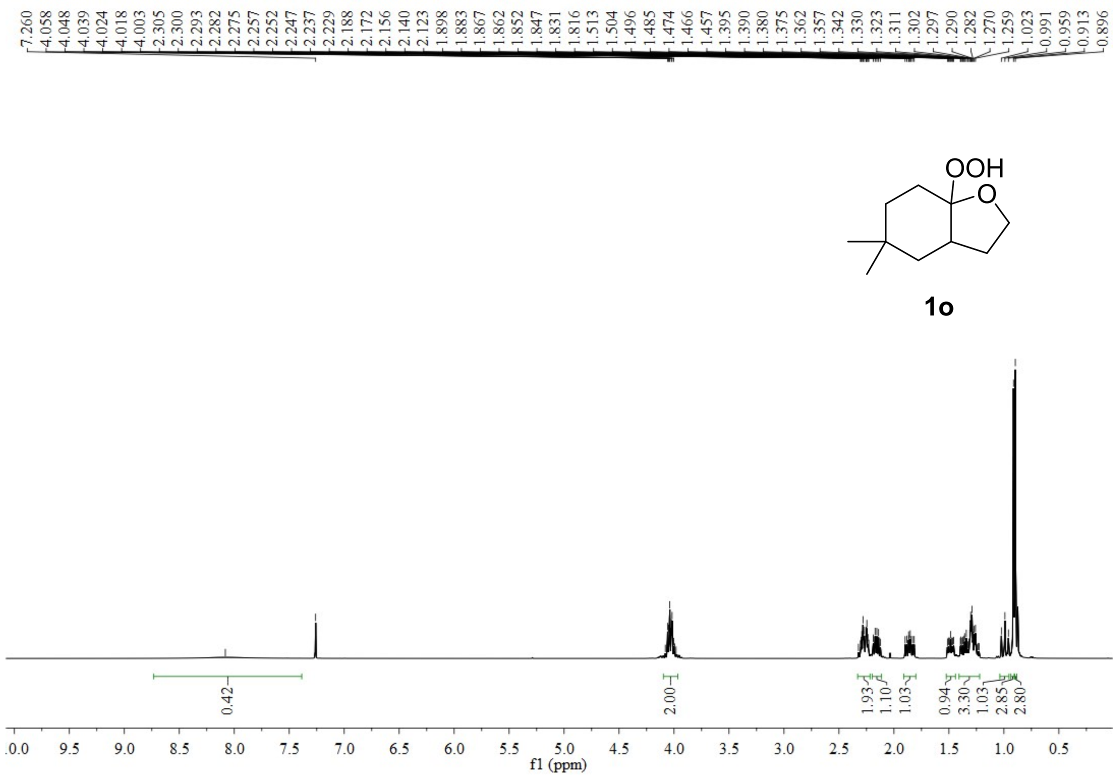

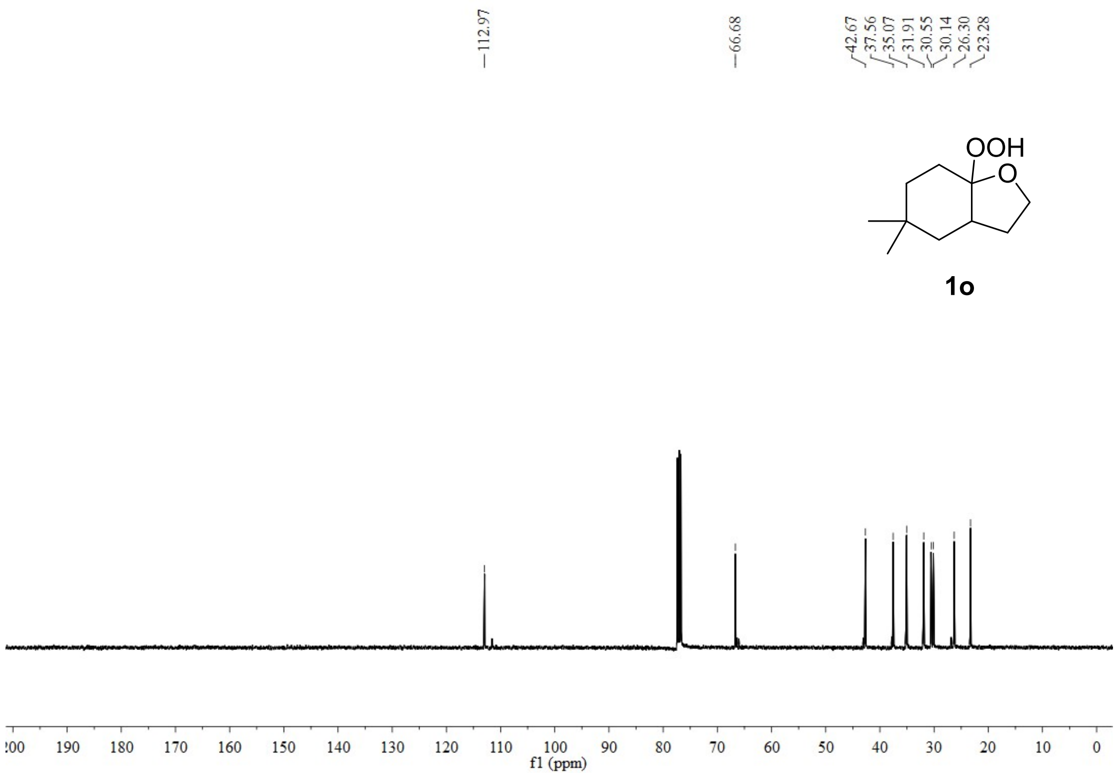

^{1}H NMR (400 MHz, $CDCl_3$) and ^{13}C NMR (100 MHz, $CDCl_3$) spectra of product **1p**

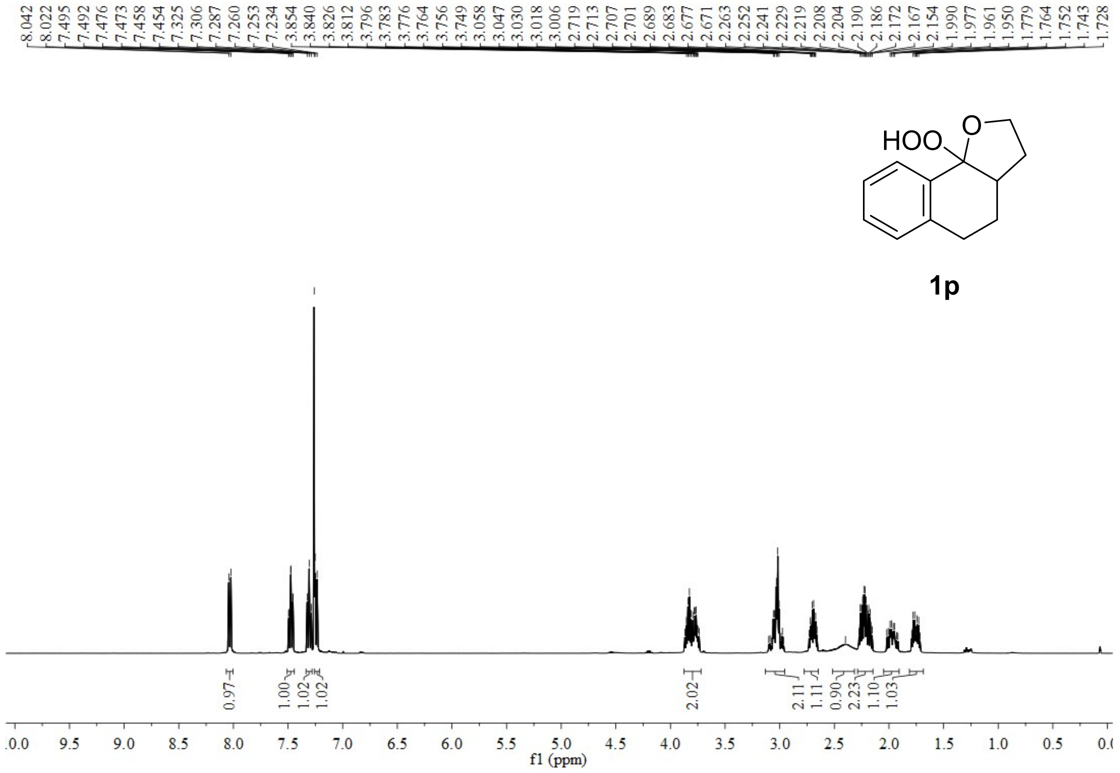

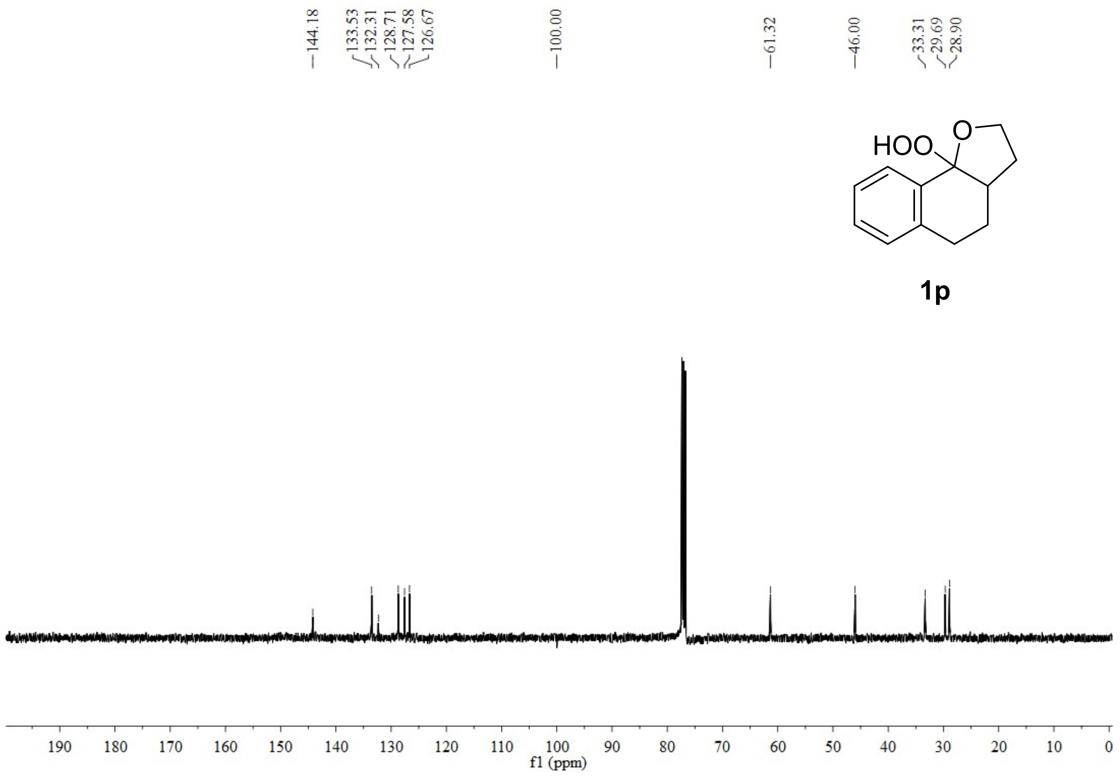

^{1}H NMR (400 MHz, $CDCl_3$) and ^{13}C NMR (100 MHz, $CDCl_3$) spectra of product **1q**

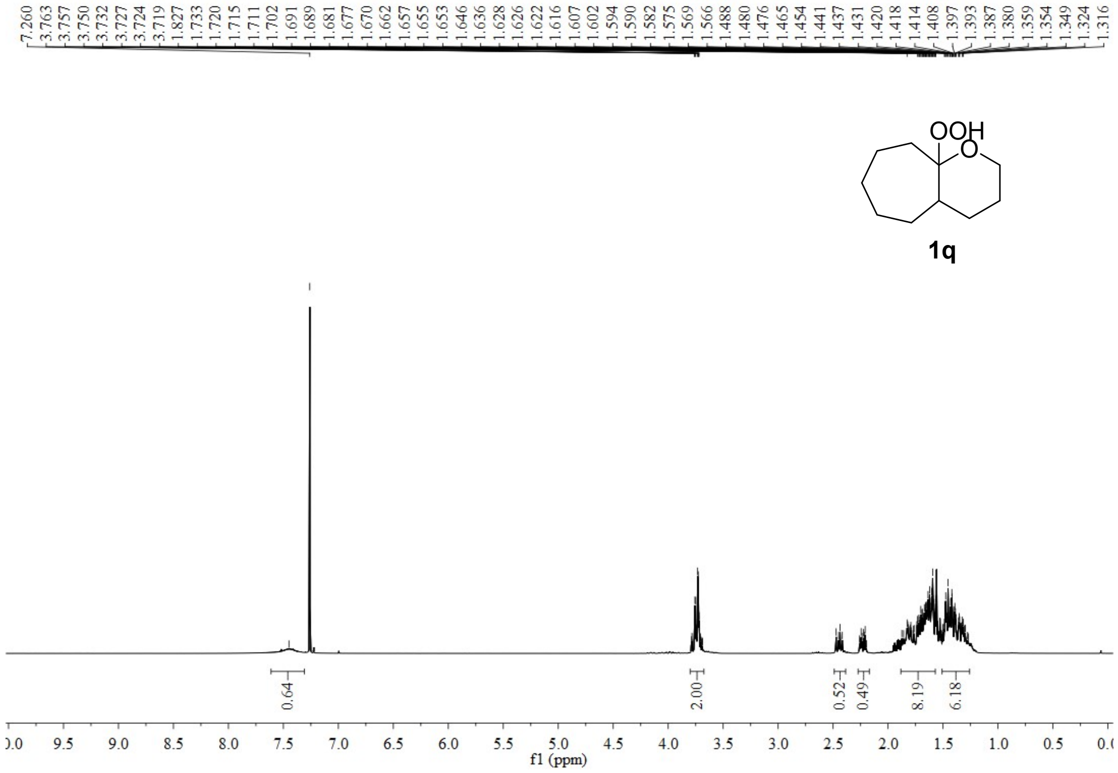

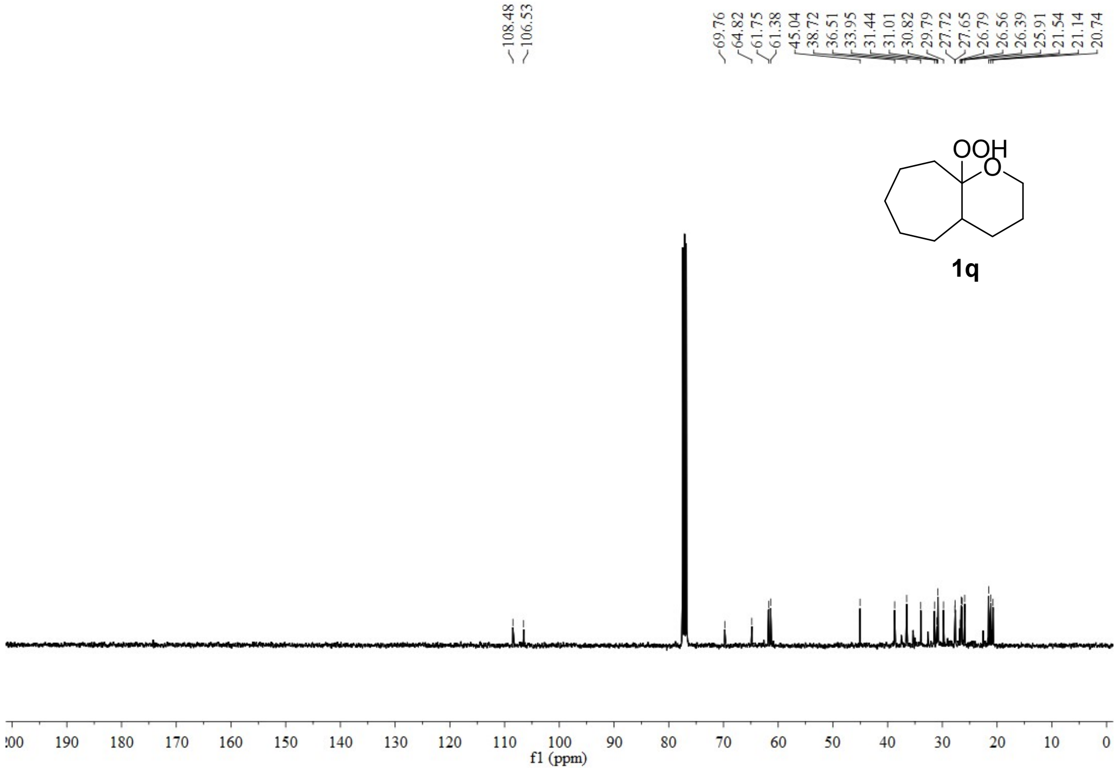

^{1}H NMR (400 MHz, $CDCl_3$) and ^{13}C NMR (100 MHz, $CDCl_3$) spectra of product **1r**

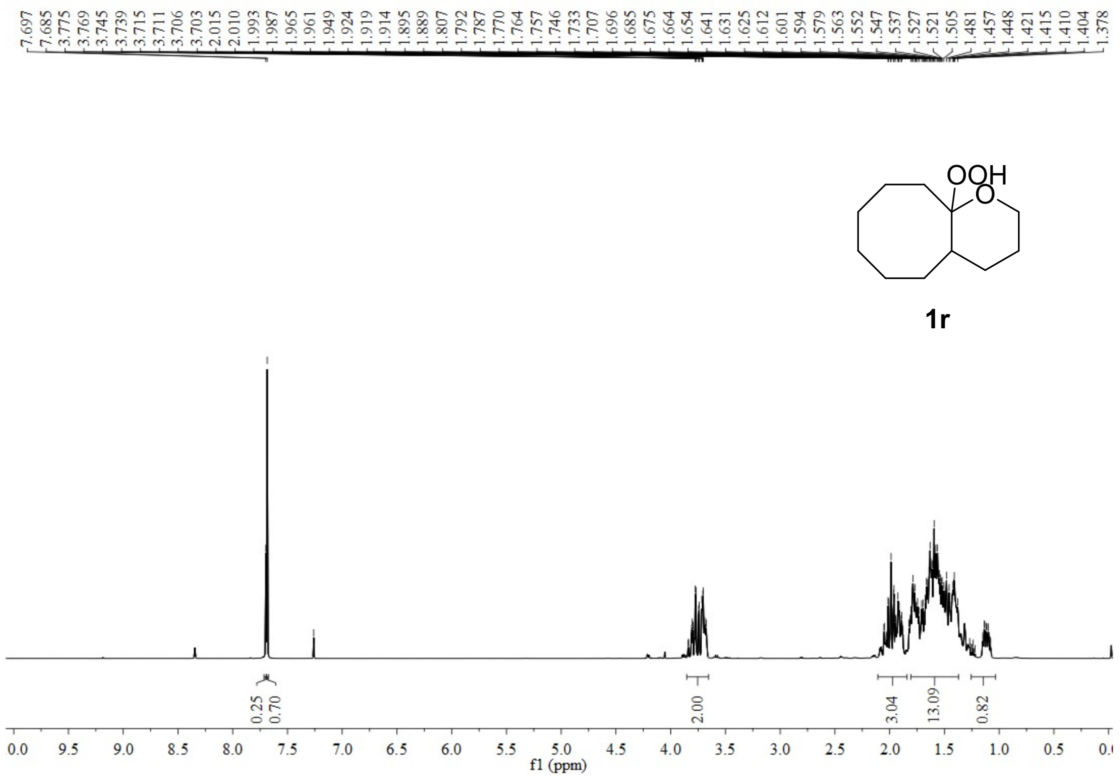

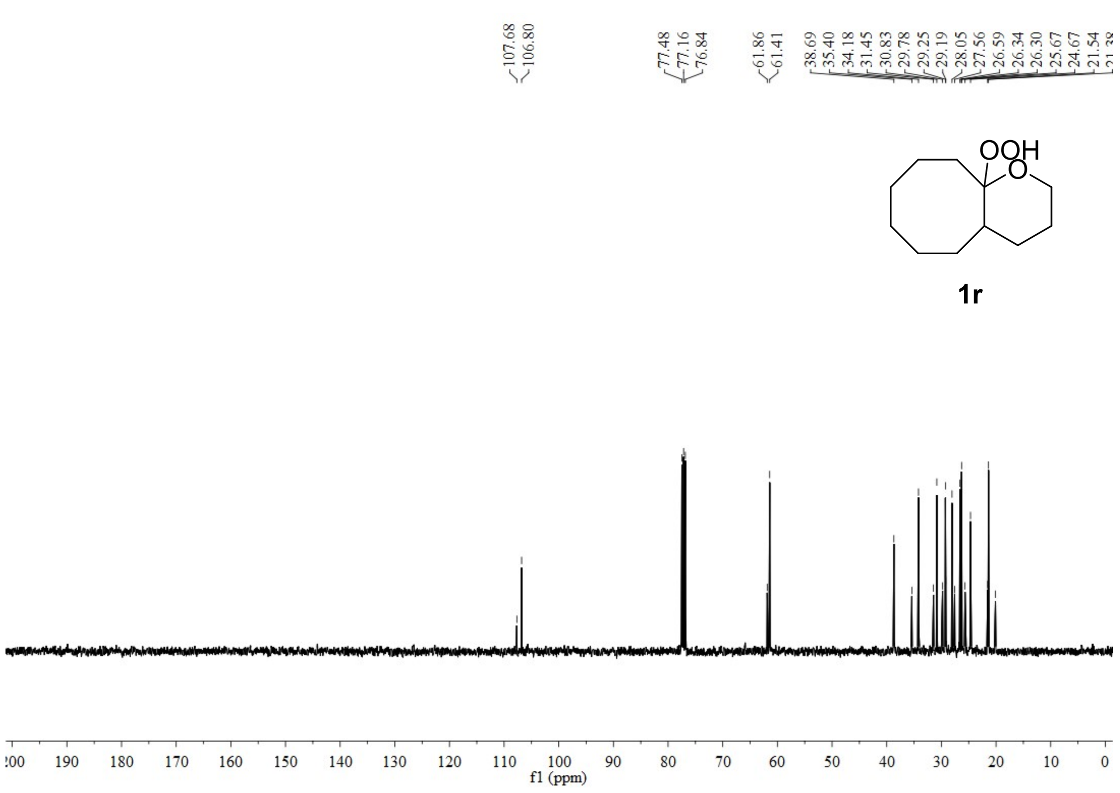

^{1}H NMR (400 MHz, $CDCl_3$) and ^{13}C NMR (100 MHz, $CDCl_3$) spectra of product **1s**

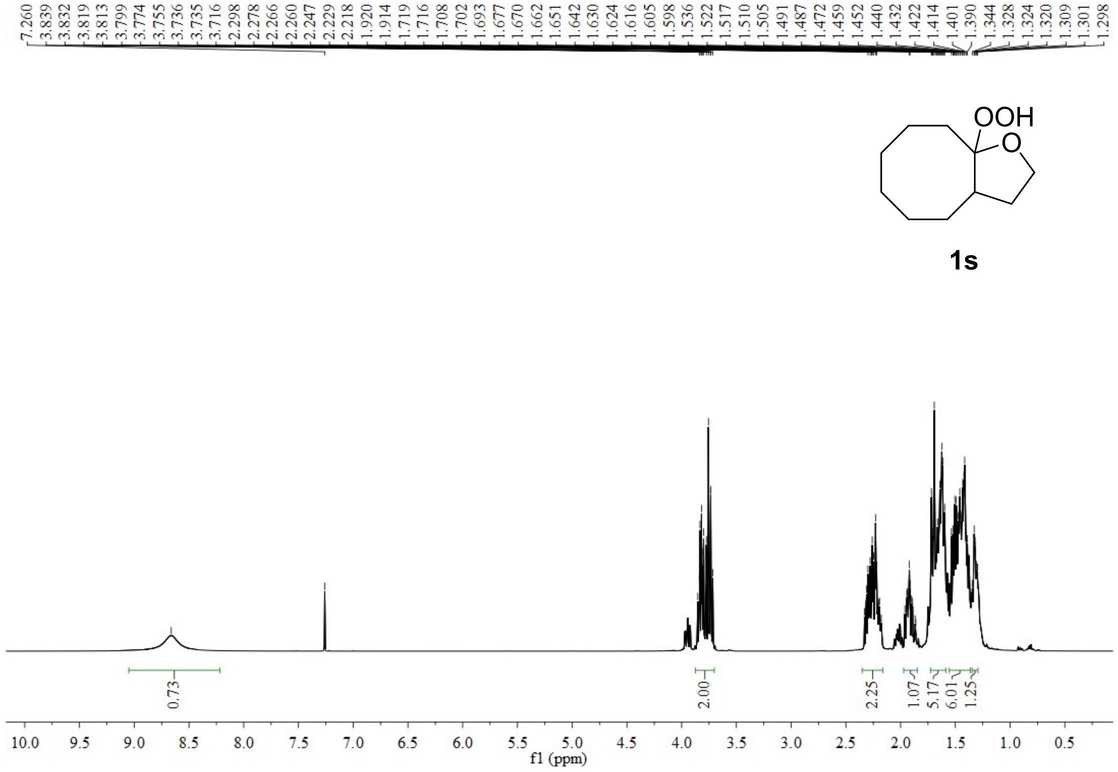

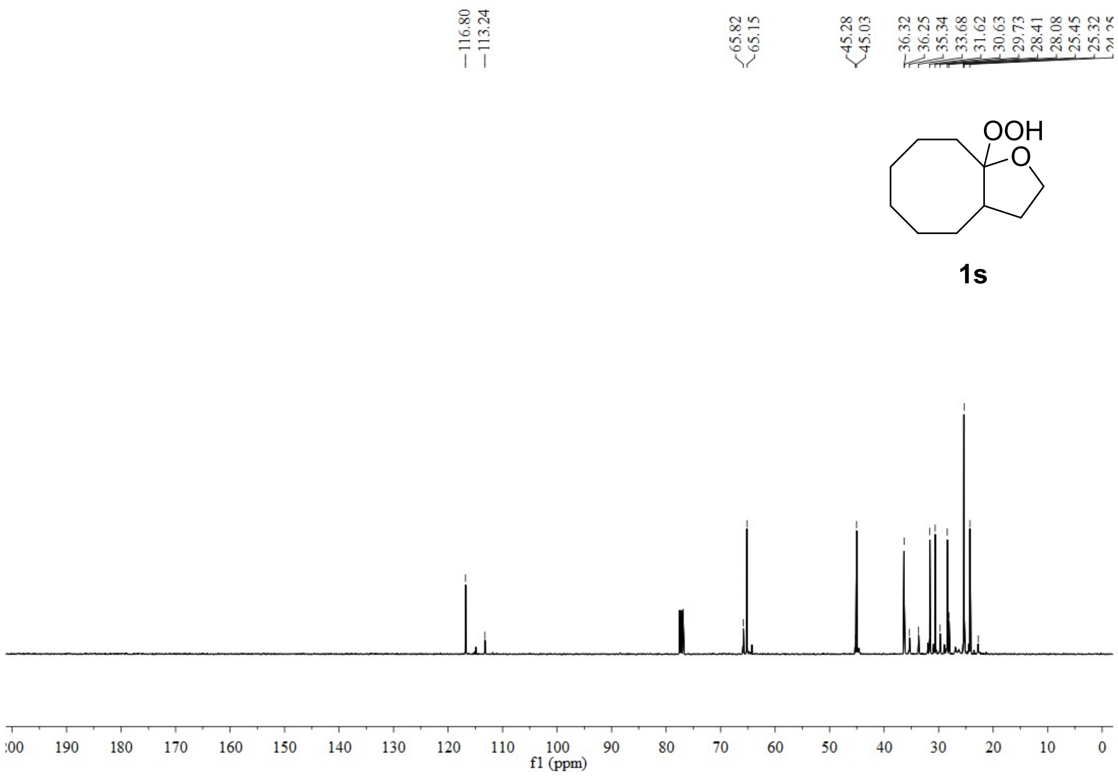

^{1}H NMR (400 MHz, $CDCl_3$) and ^{13}C NMR (100 MHz, $CDCl_3$) spectra of product **1t**

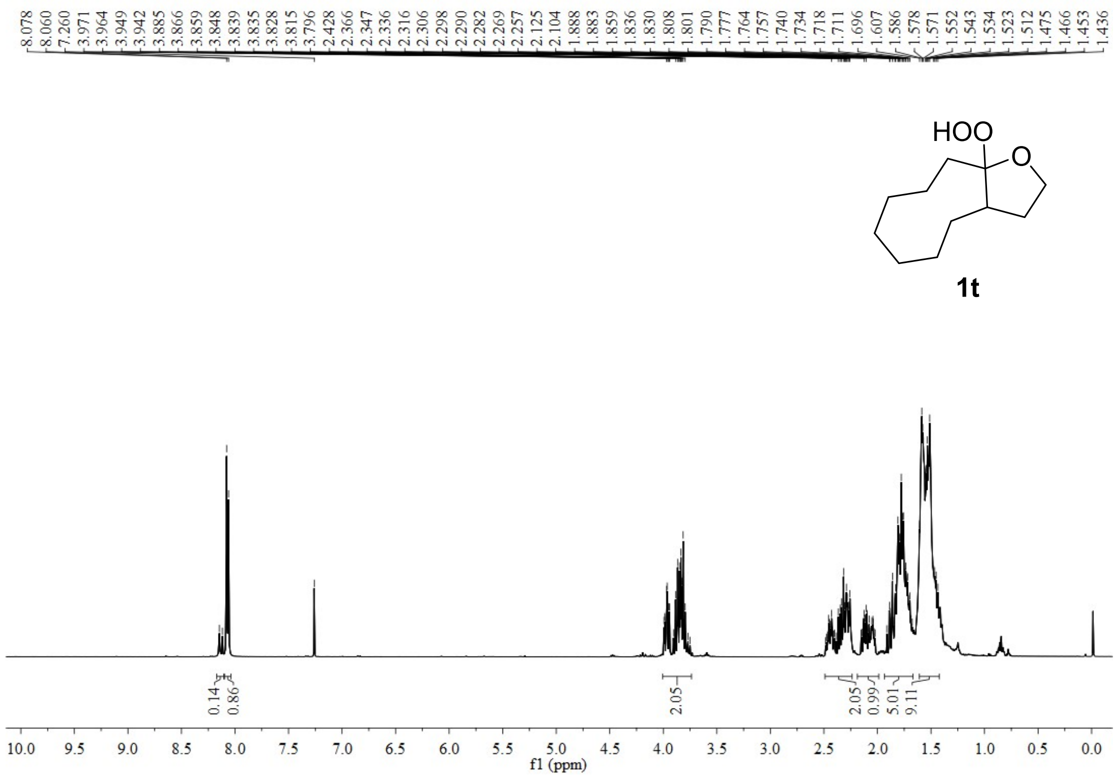

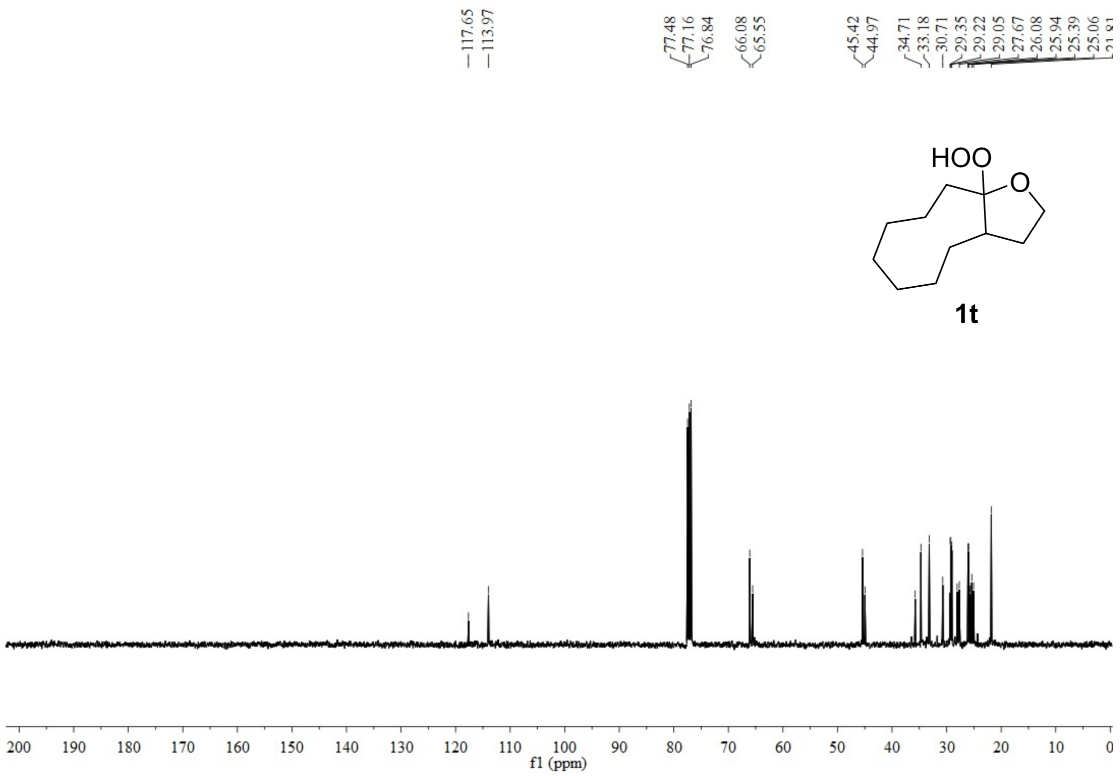

^{1}H NMR (400 MHz, DMSO-d_6) and ^{13}C NMR (100 MHz, DMSO-d_6) spectra of product **1u**

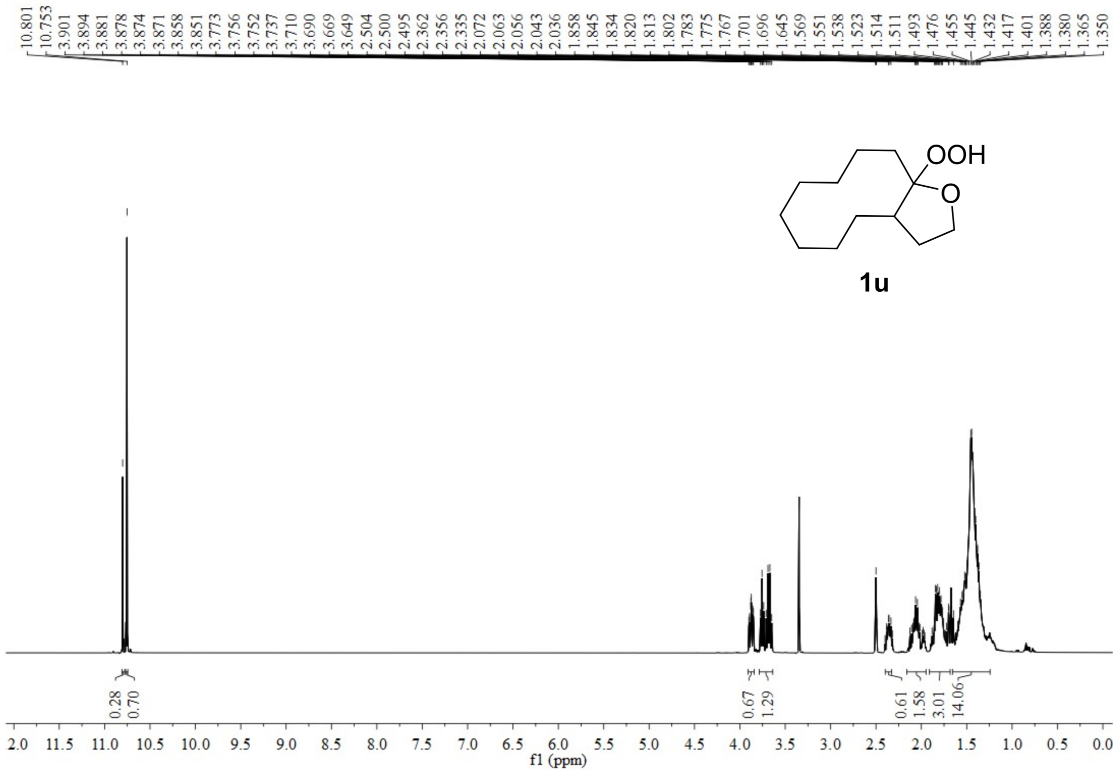

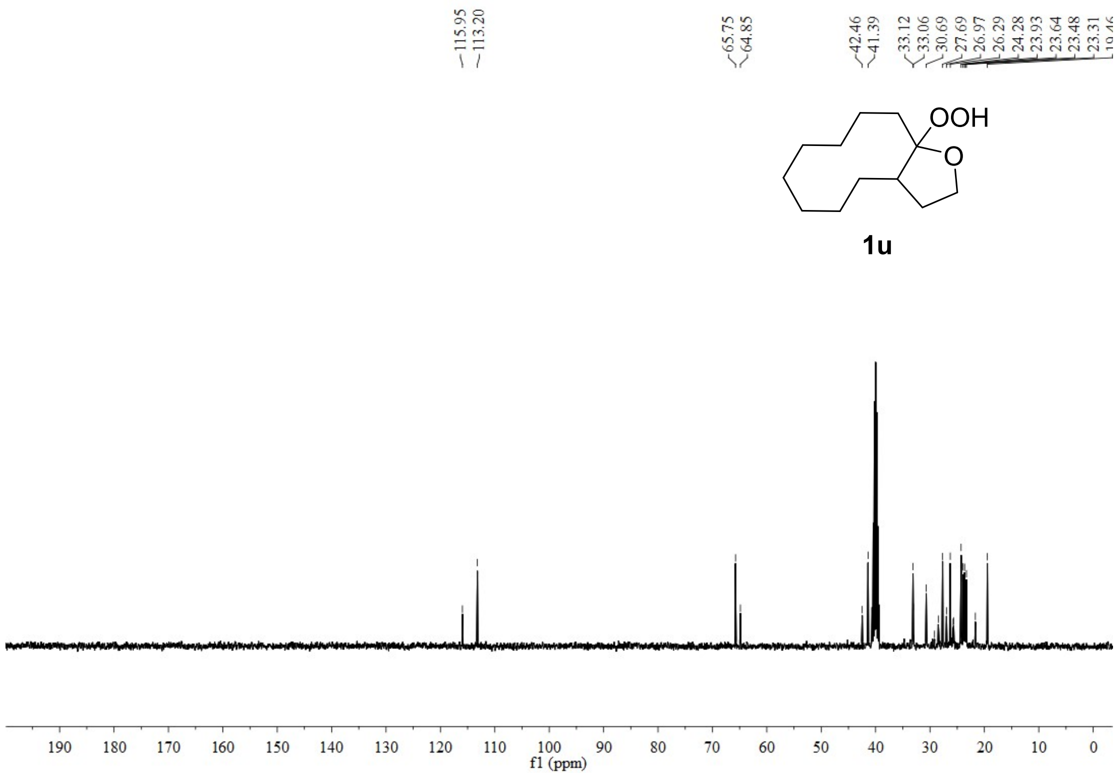

^{1}H NMR (400 MHz, $CDCl_3$) and ^{13}C NMR (100 MHz, $CDCl_3$) spectra of product **1v**

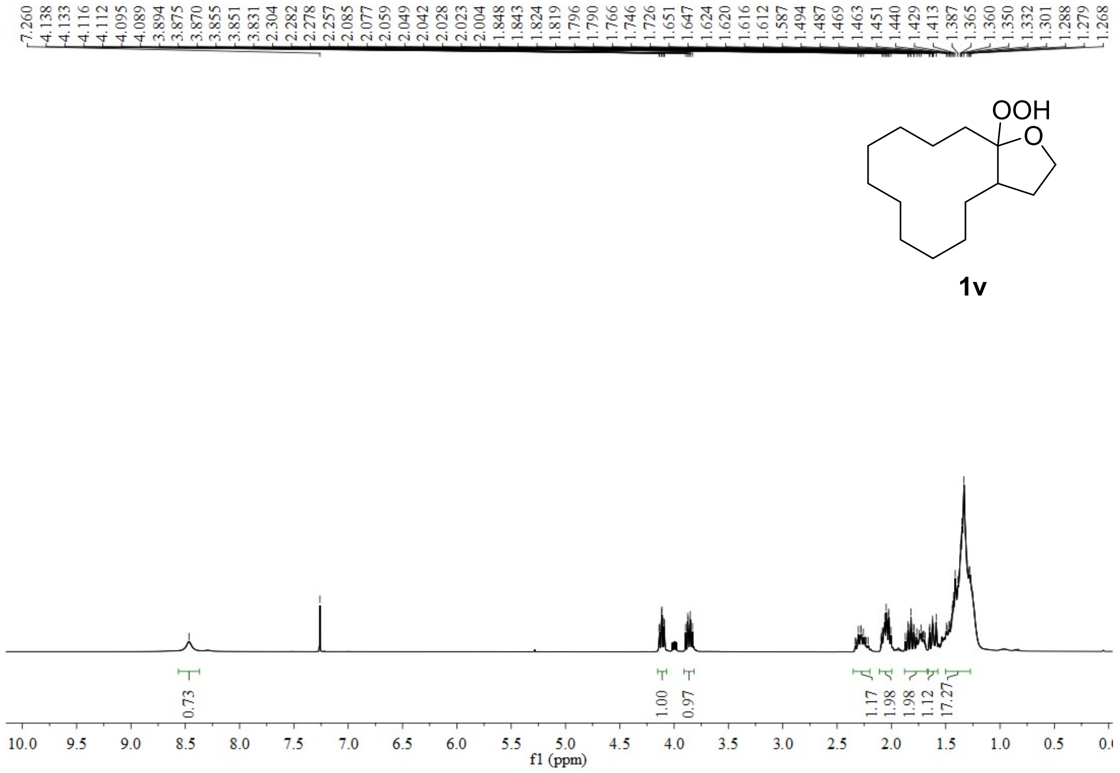

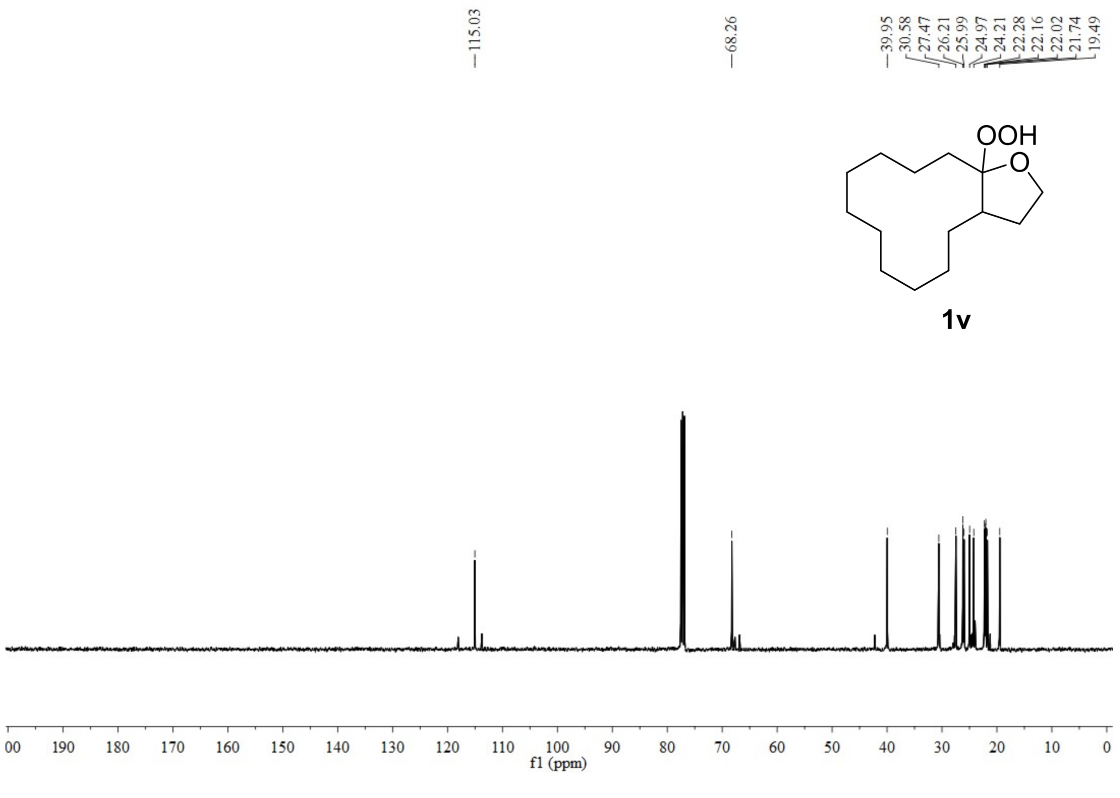

^{1}H NMR (400 MHz, $CDCl_3$) and ^{13}C NMR (100 MHz, $CDCl_3$) spectra of product **1w**

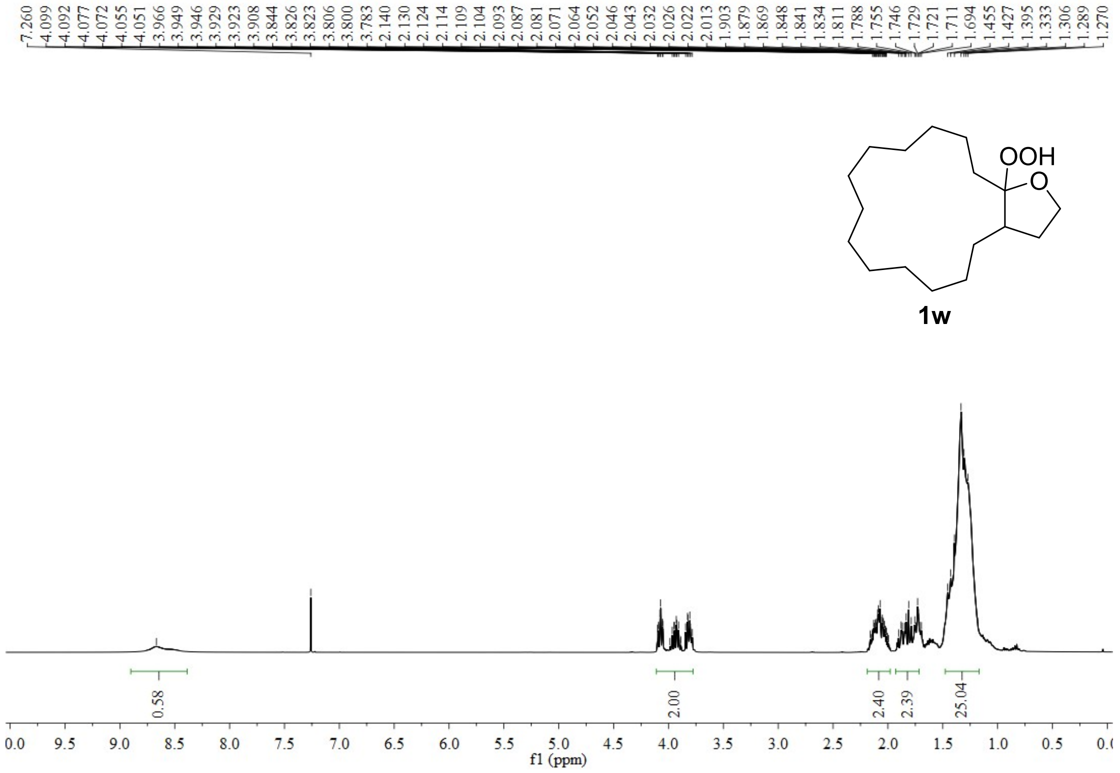

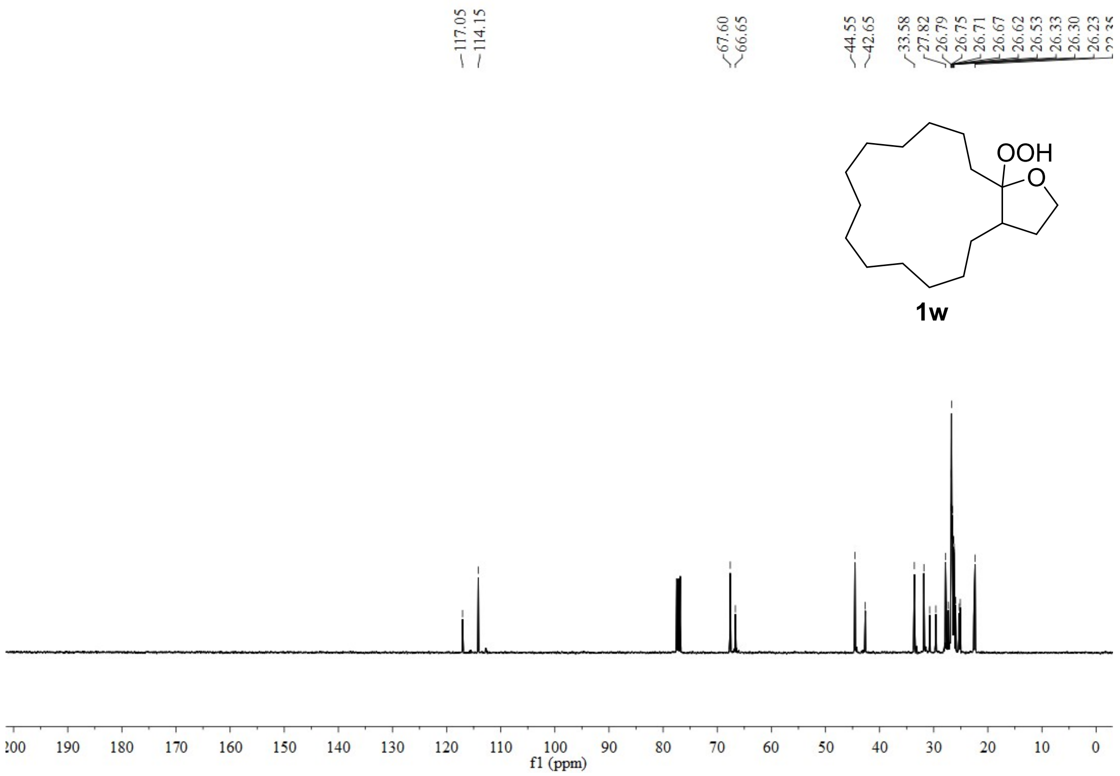

^{1}H NMR (400 MHz, $CDCl_3$) and ^{13}C NMR (100 MHz, $CDCl_3$) spectra of product **1x**

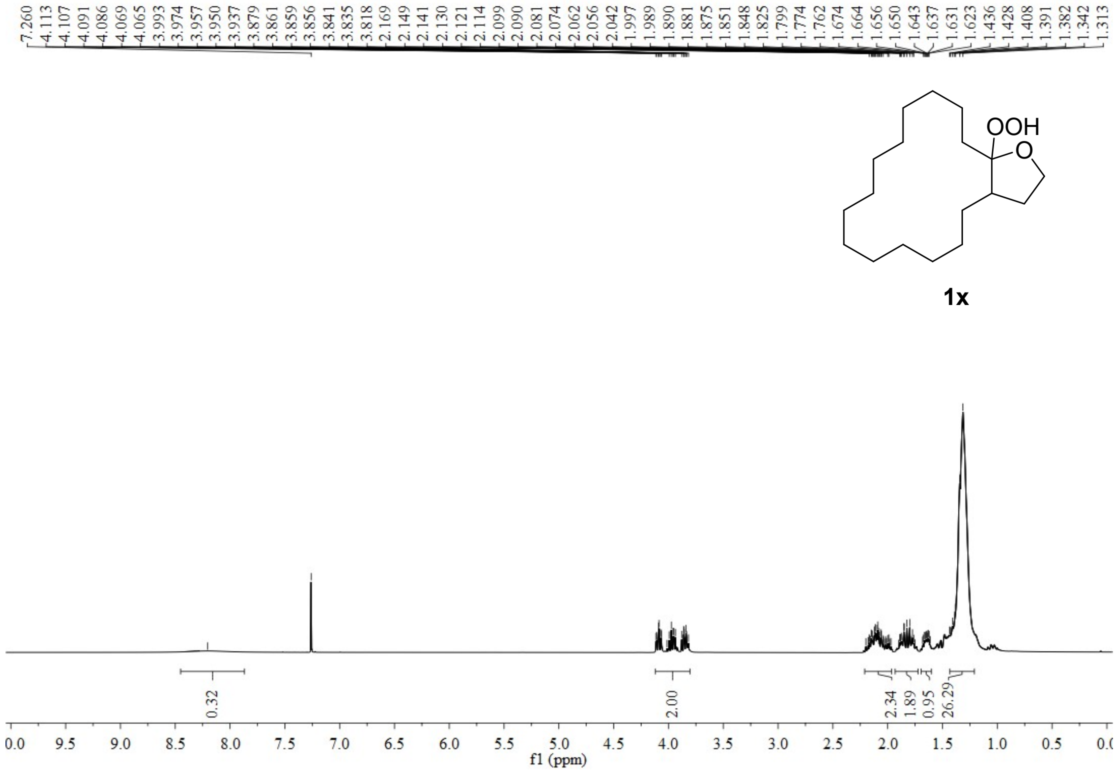

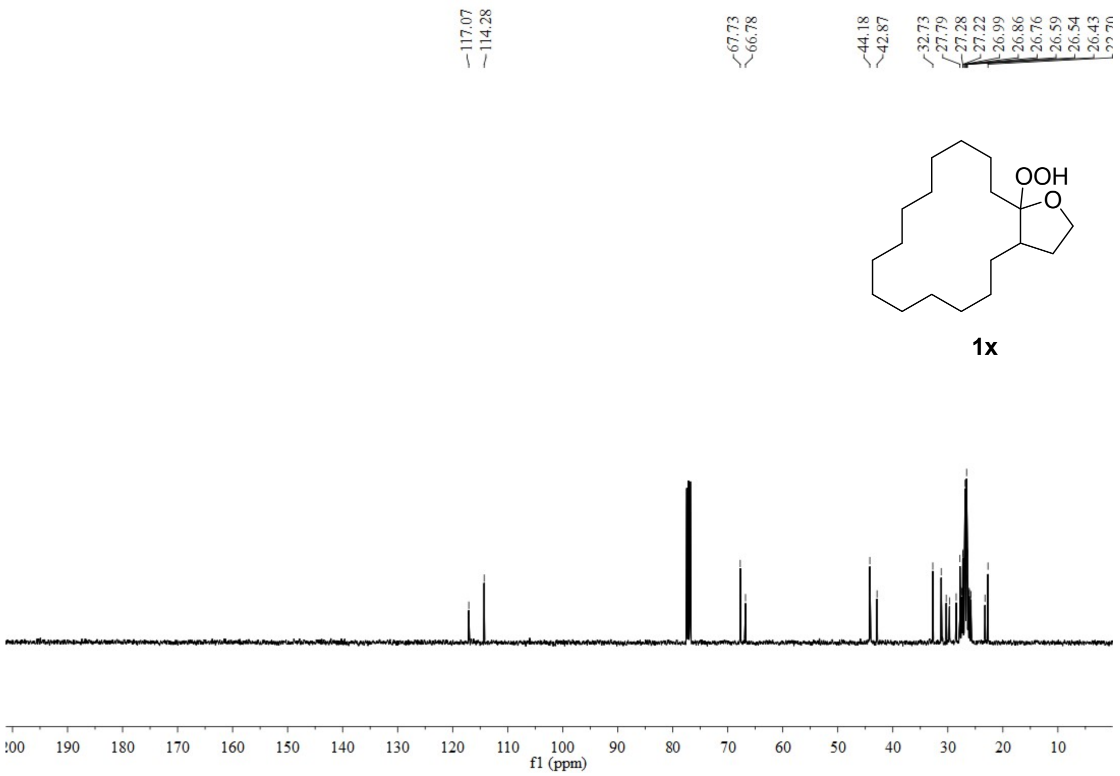

^{1}H NMR (400 MHz, $CDCl_3$) and ^{13}C NMR (100 MHz, $CDCl_3$) spectra of product **1y**

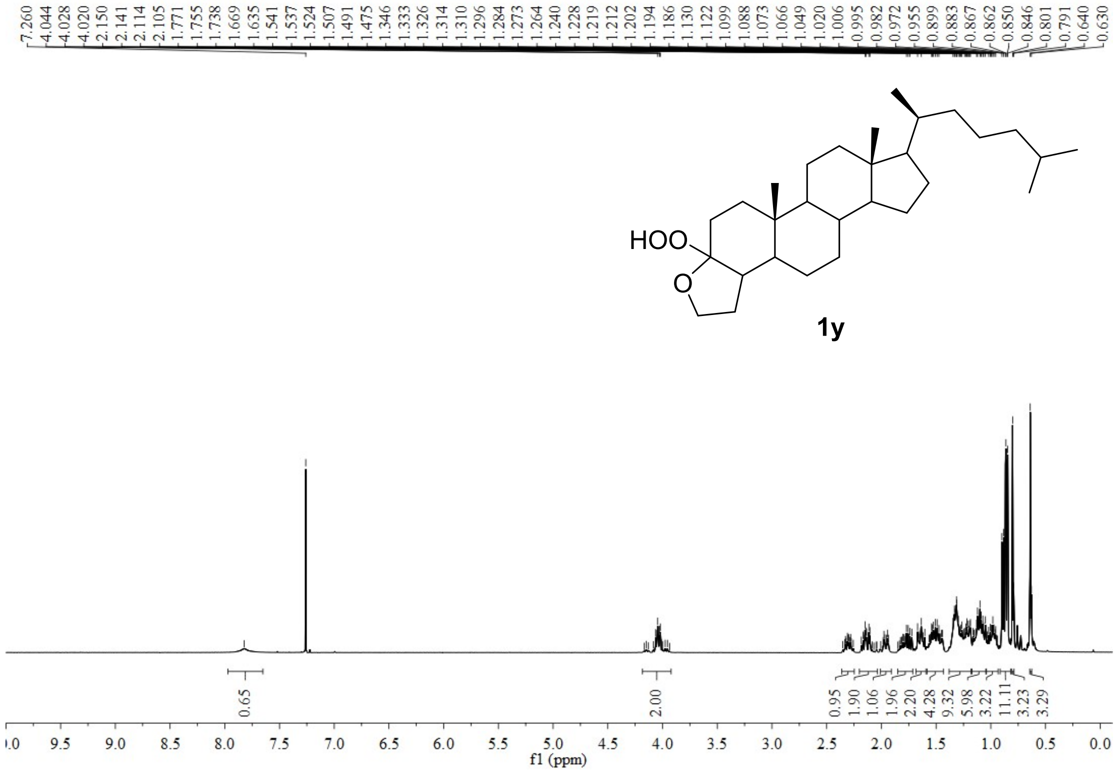

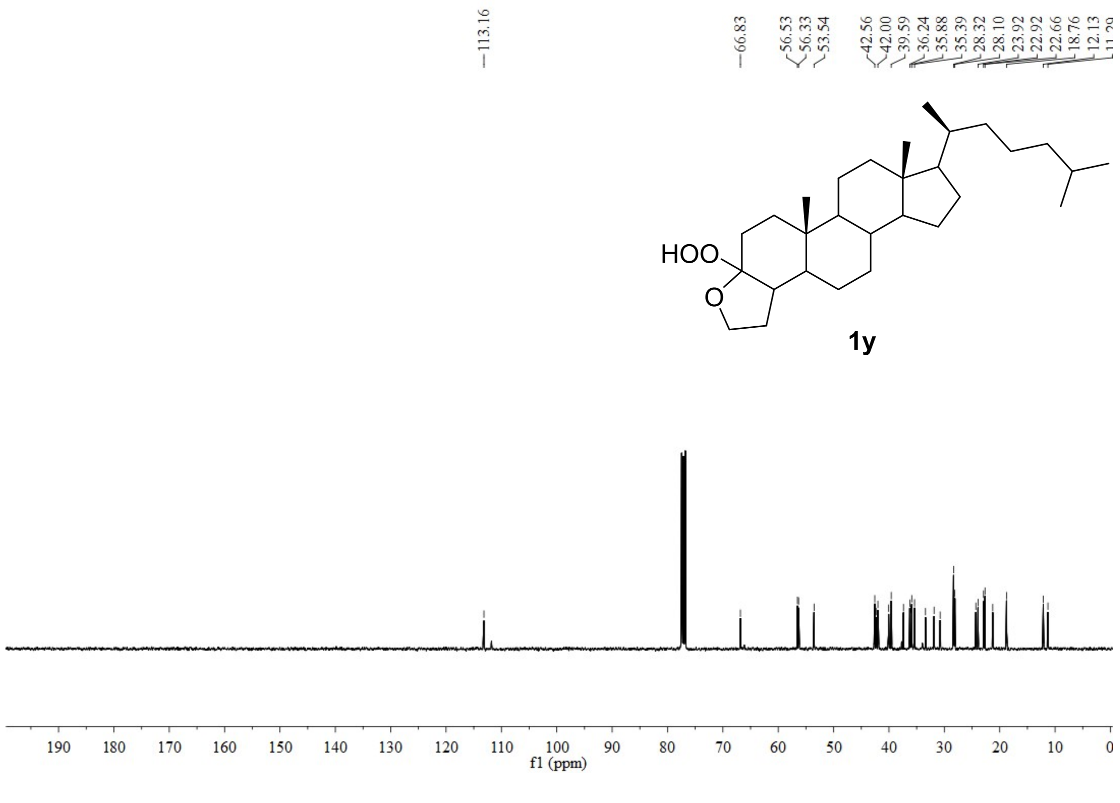

15. ^{1}H NMR and ^{13}C NMR Spectra of Products 2-5

^{1}H NMR (400 MHz, $CDCl_3$) and ^{13}C NMR (100 MHz, $CDCl_3$) spectra of product **2a**

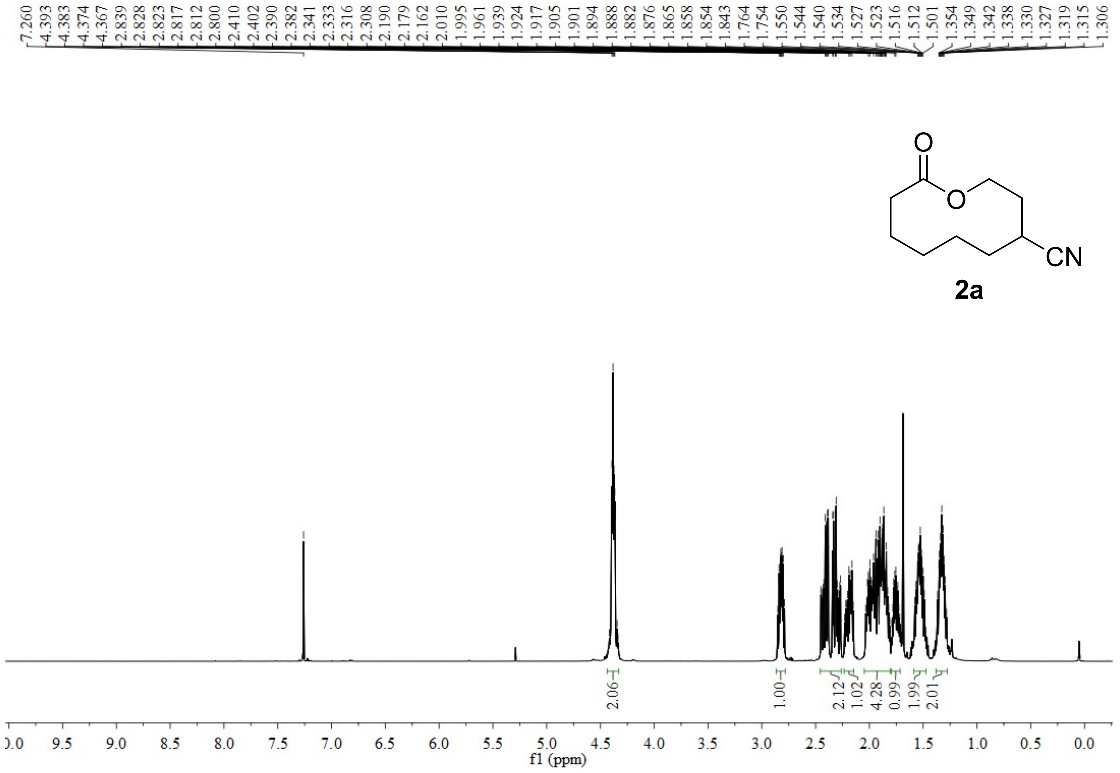

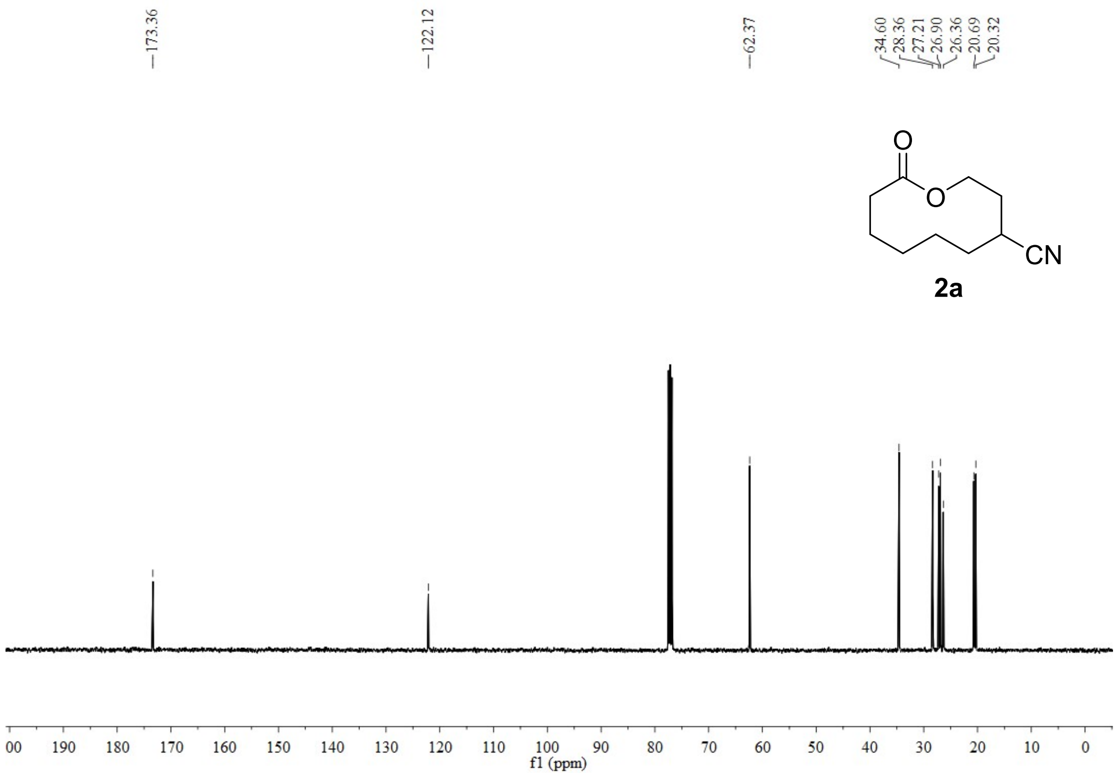

^{1}H NMR (400 MHz, $CDCl_3$) and ^{13}C NMR (100 MHz, $CDCl_3$) spectra of product **2b**

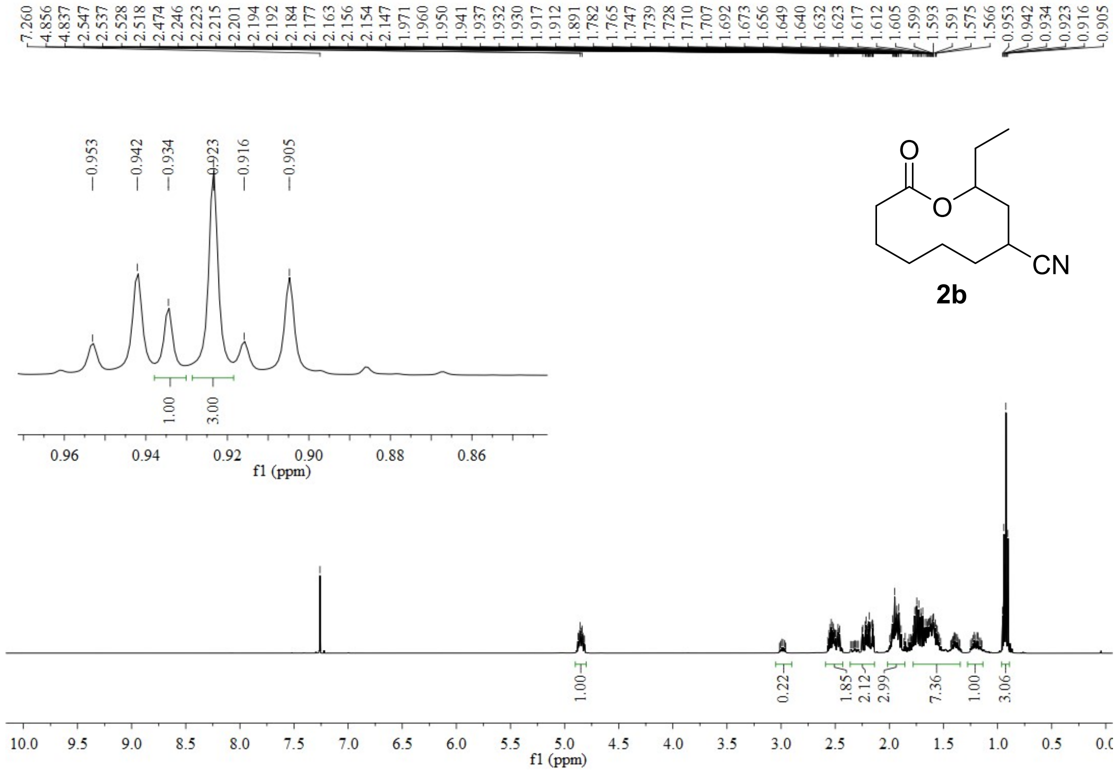

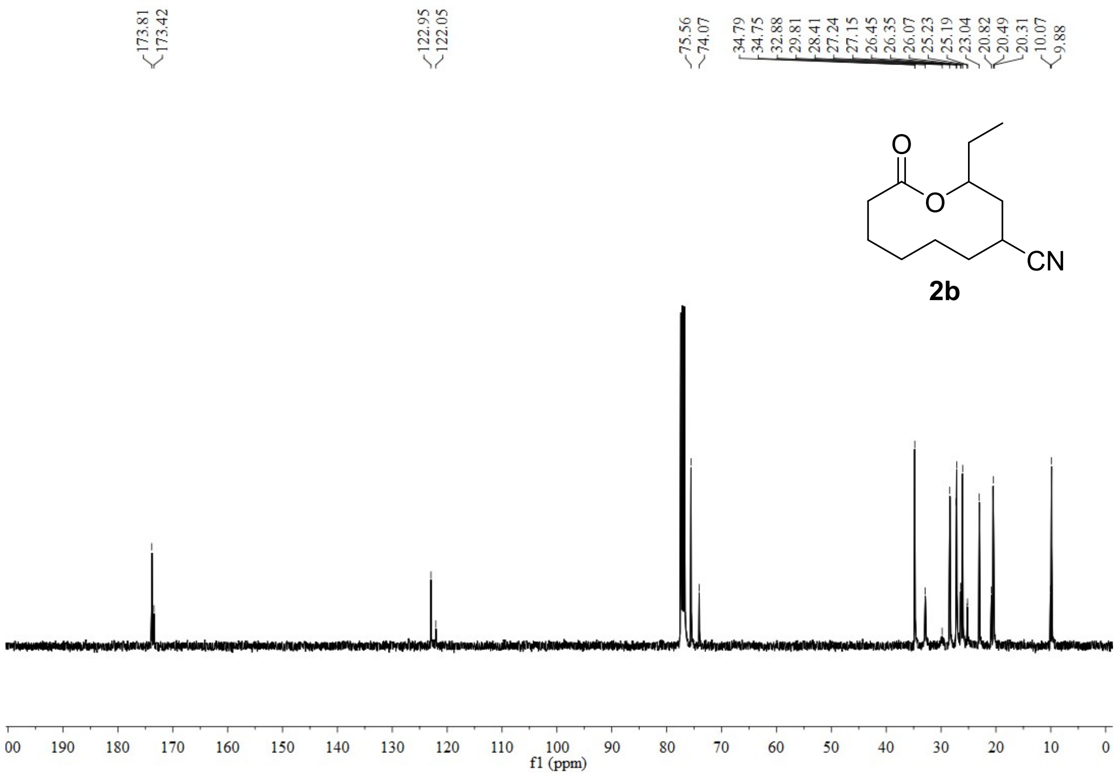

^{1}H NMR (400 MHz, $CDCl_3$) and ^{13}C NMR (100 MHz, $CDCl_3$) spectra of product **2c**

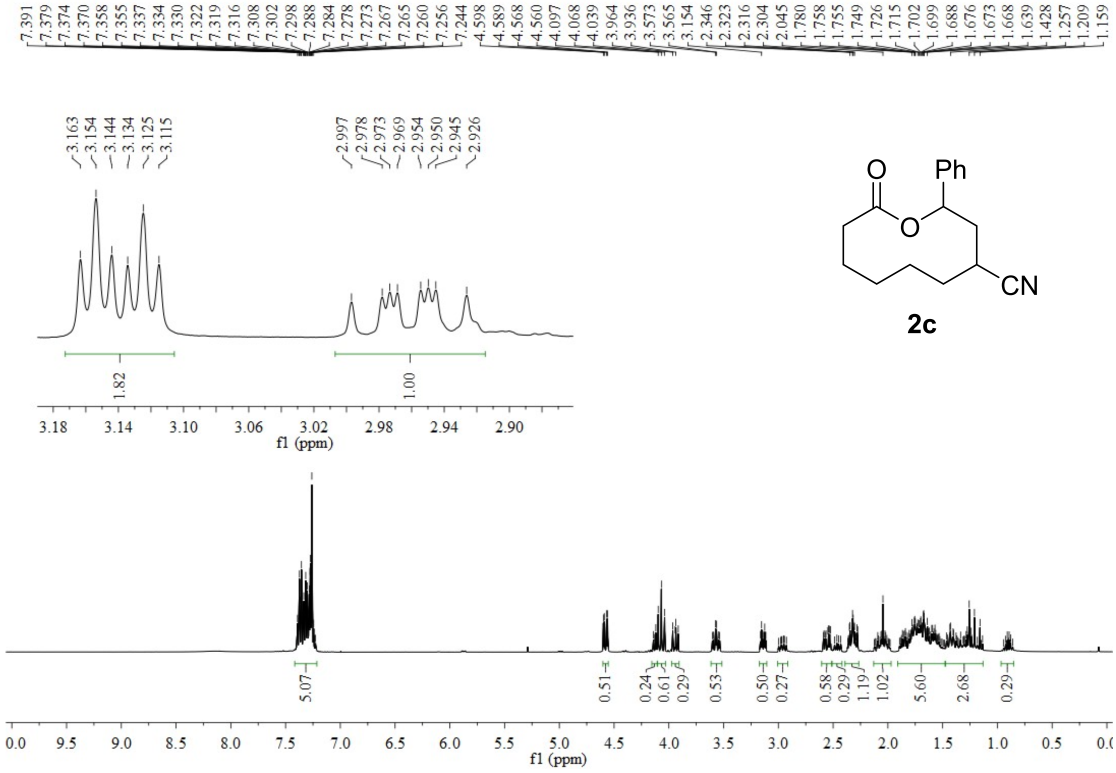

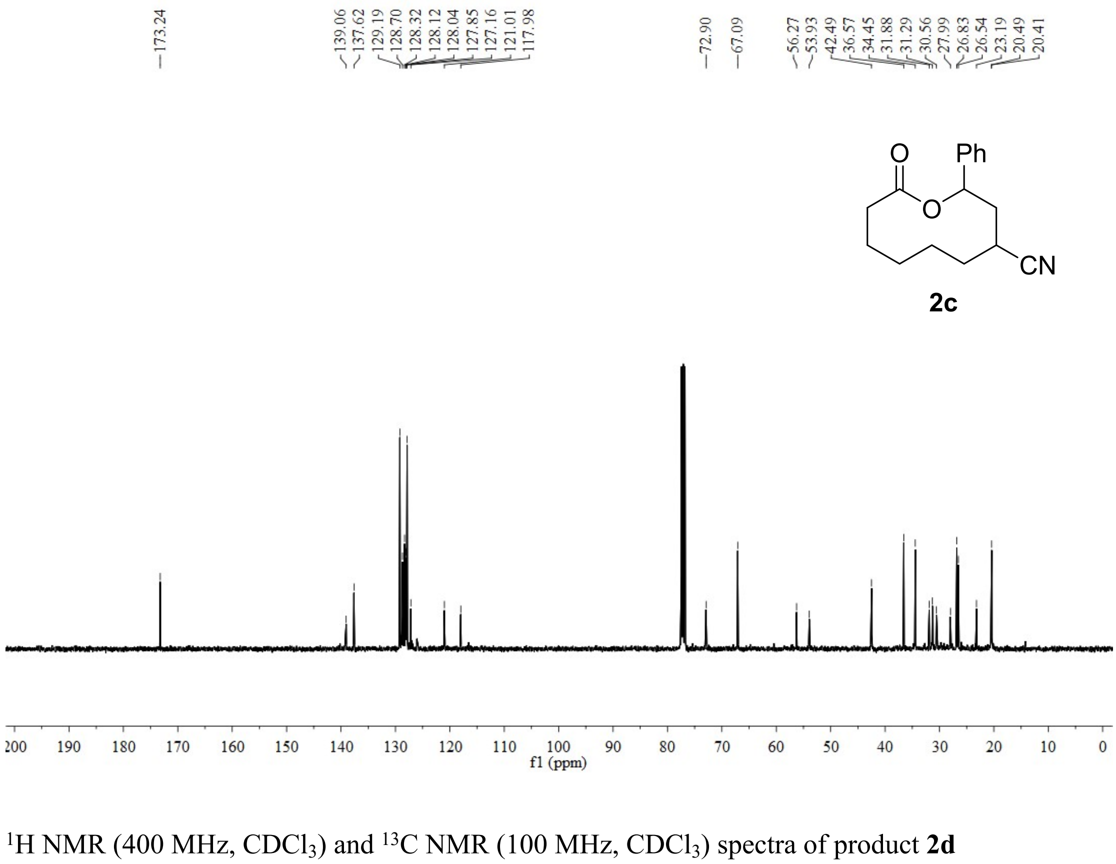

^{1}H NMR (400 MHz, CDCl$_3$) and ^{13}C NMR (100 MHz, CDCl$_3$) spectra of product **2d**

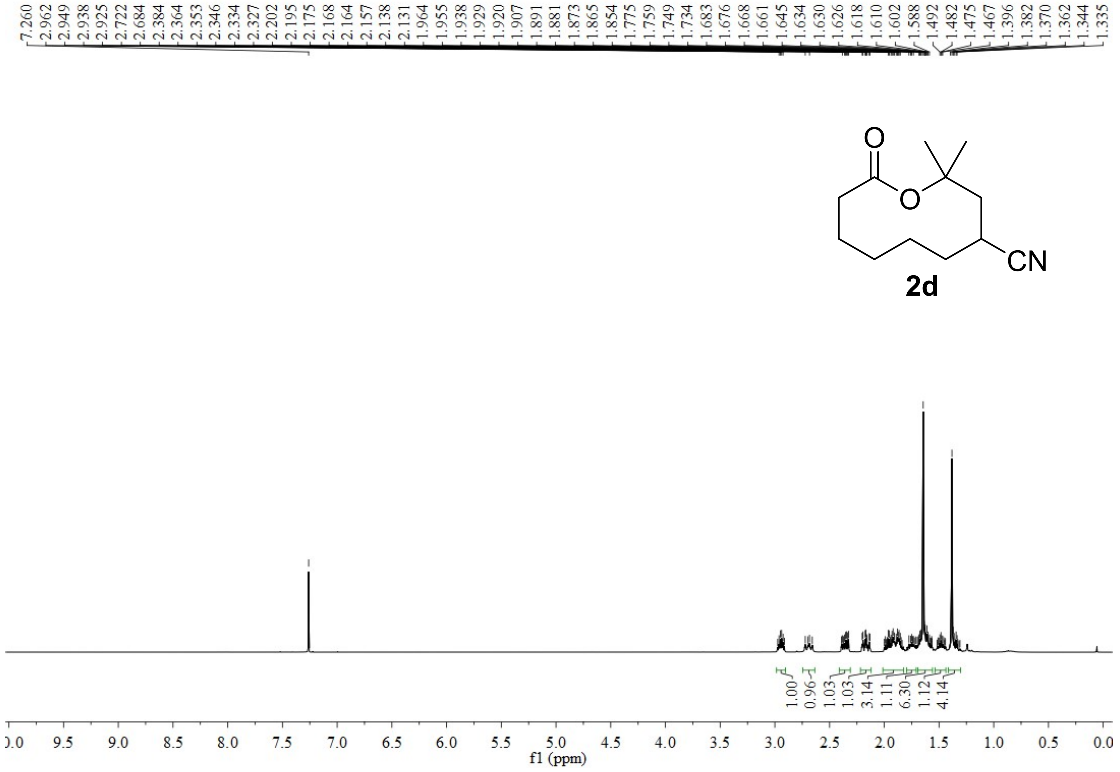

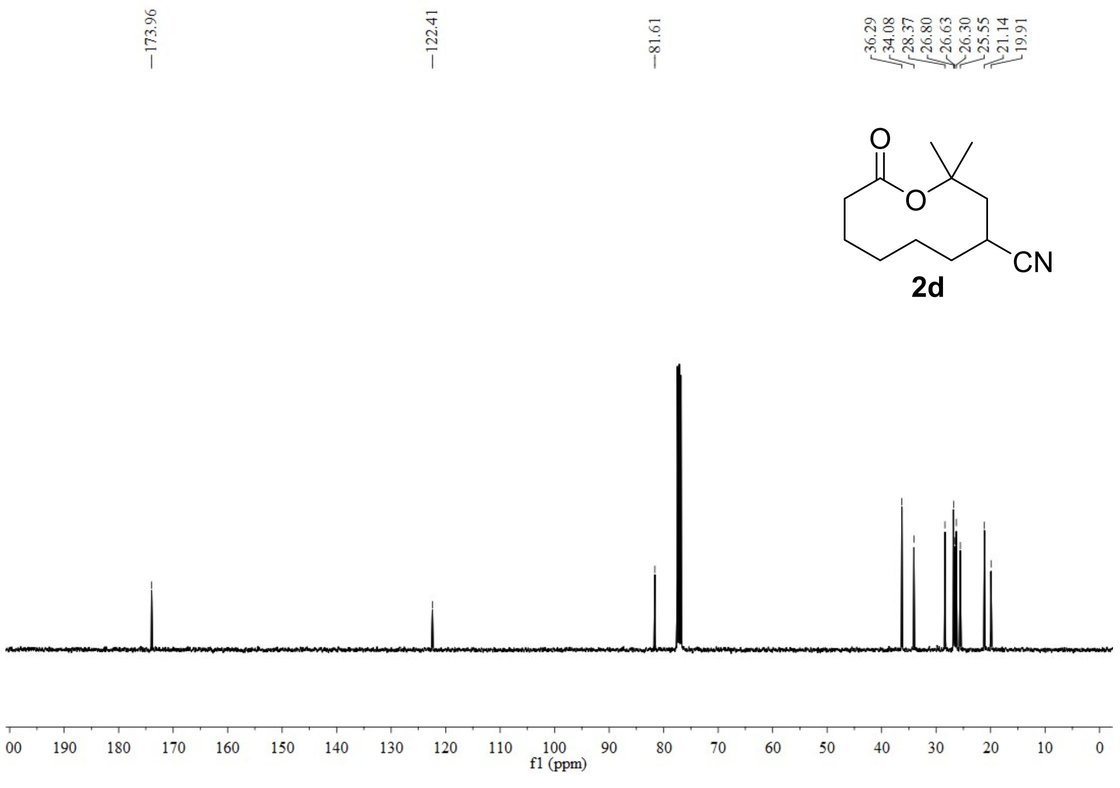

^{1}H NMR (400 MHz, $CDCl_3$) and ^{13}C NMR (100 MHz, $CDCl_3$) spectra of product **2e**

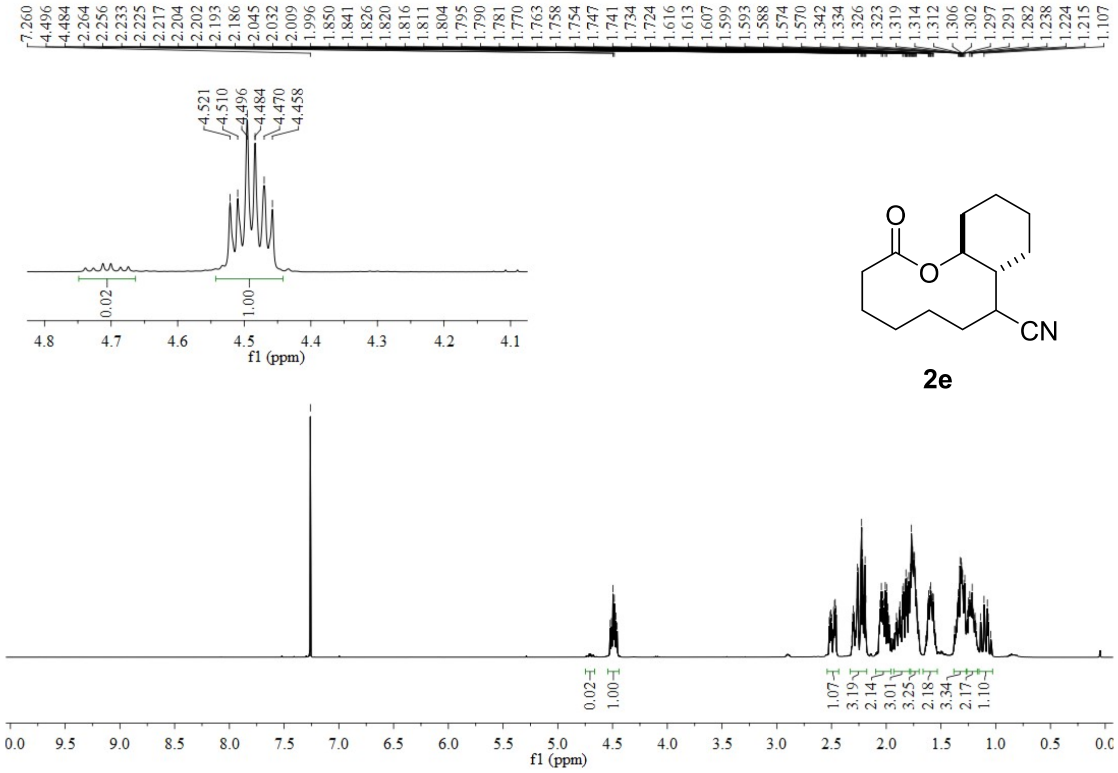

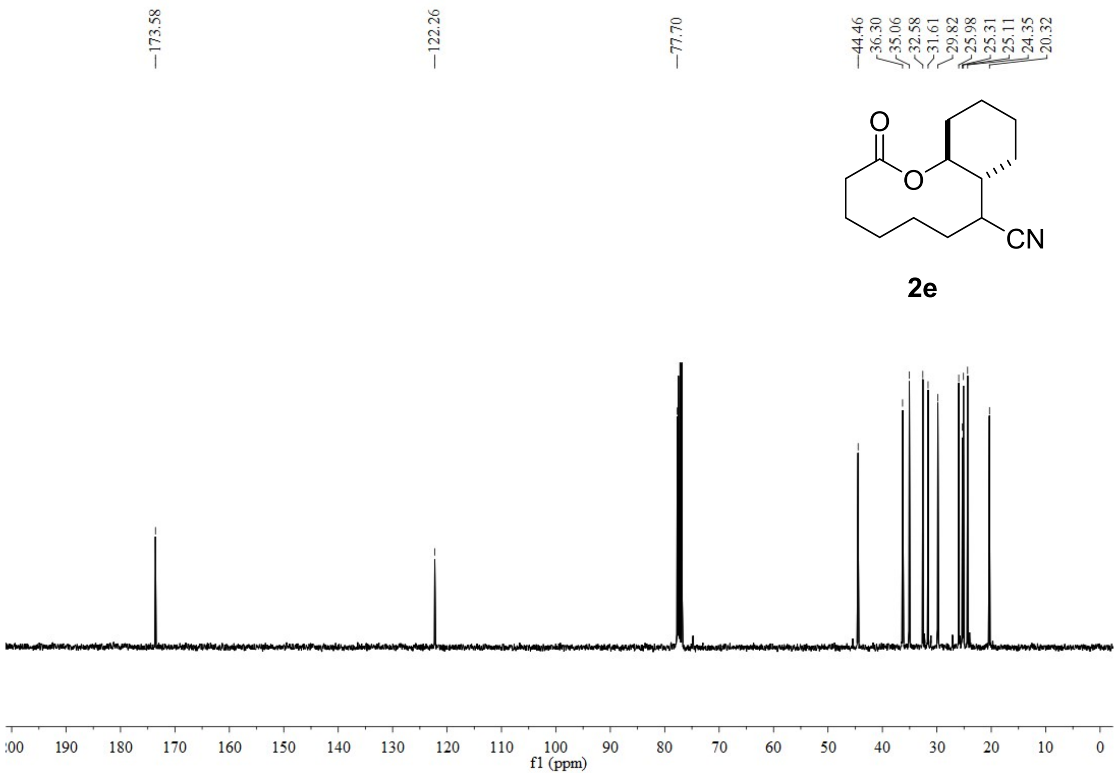

^{1}H NMR (400 MHz, $CDCl_3$) and ^{13}C NMR (100 MHz, $CDCl_3$) spectra of product **2f**

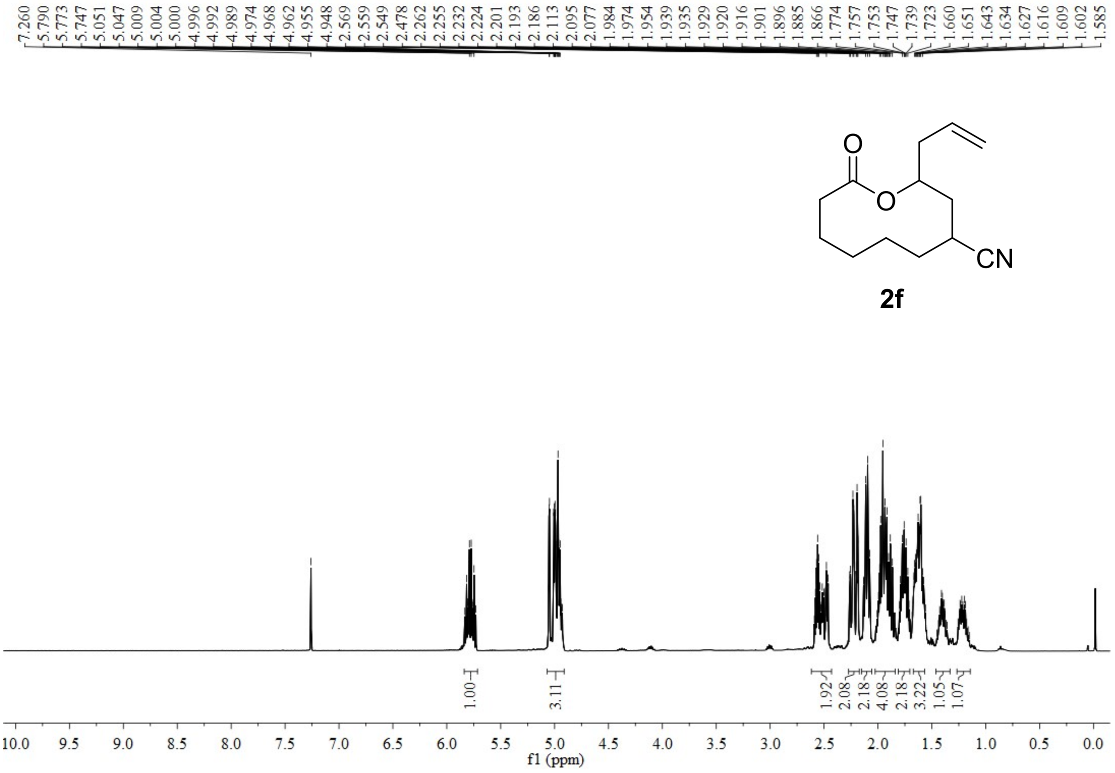

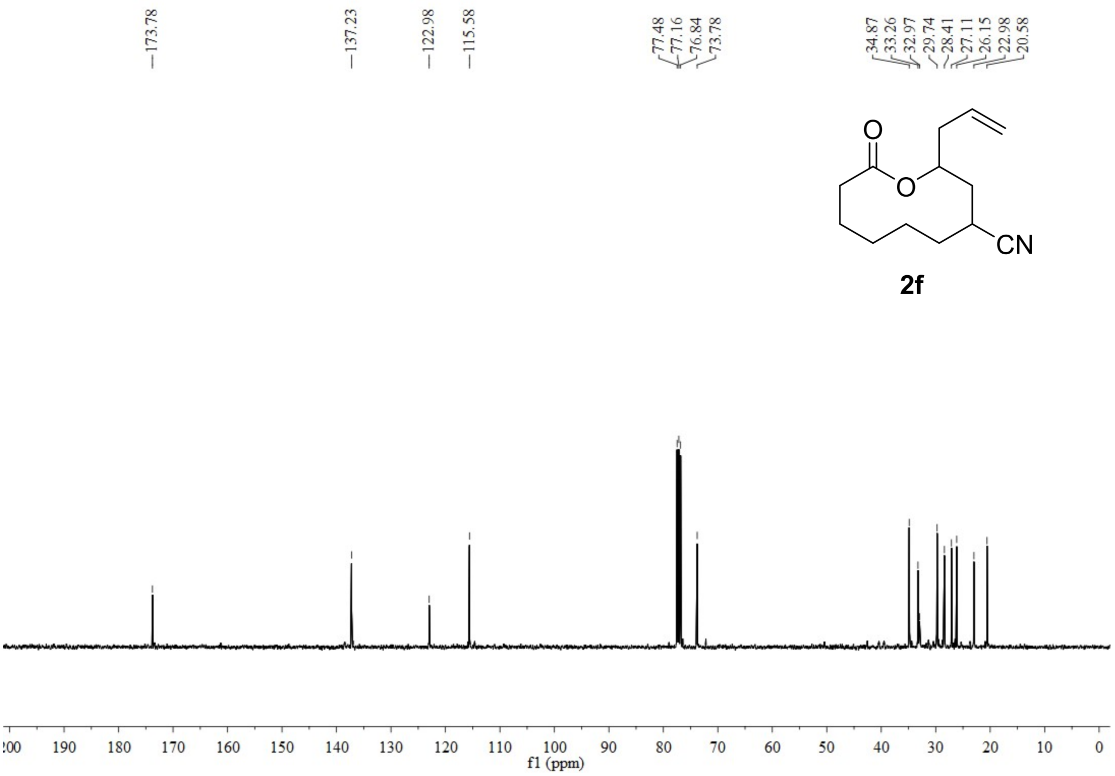

^{1}H NMR (400 MHz, $CDCl_3$) and ^{13}C NMR (100 MHz, $CDCl_3$) spectra of product **2g**

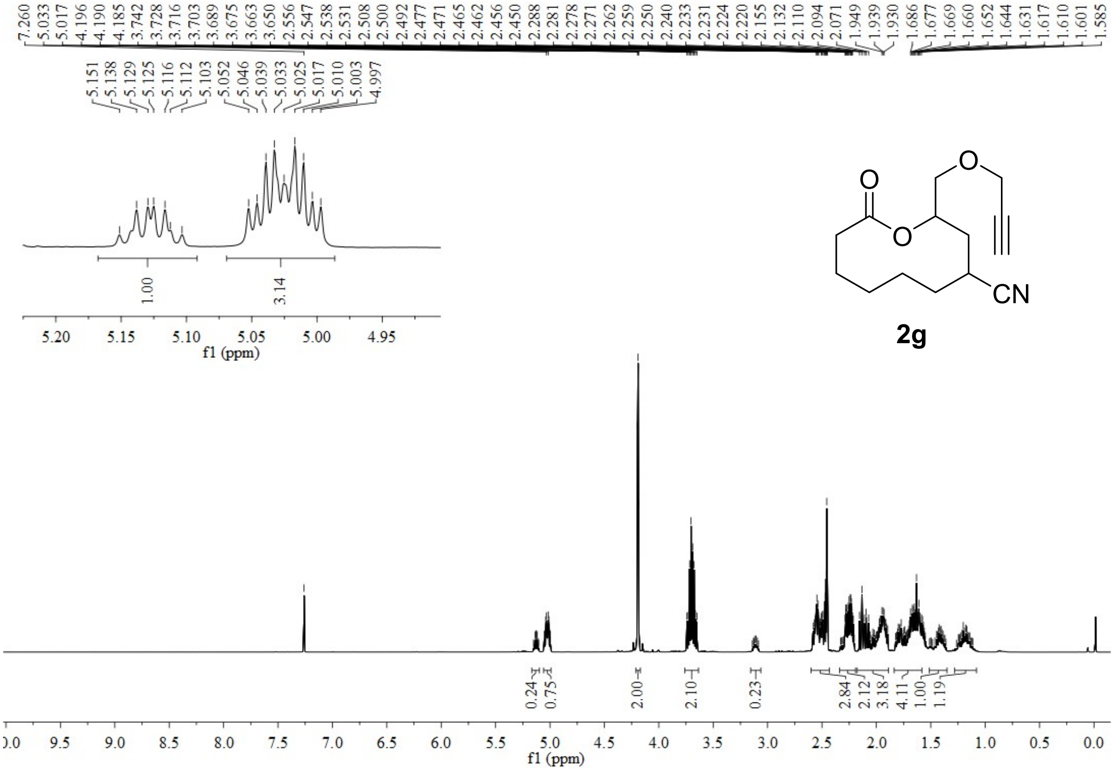

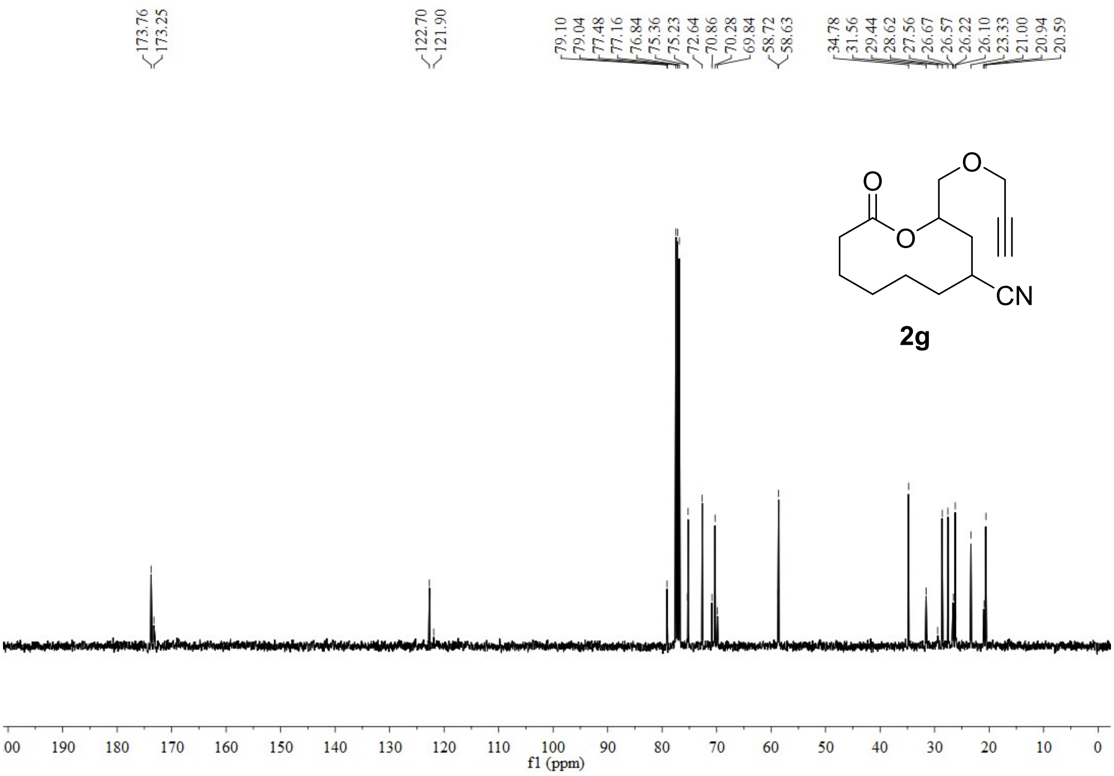

^{1}H NMR (400 MHz, $CDCl_3$) and ^{13}C NMR (100 MHz, $CDCl_3$) spectra of product **2h**

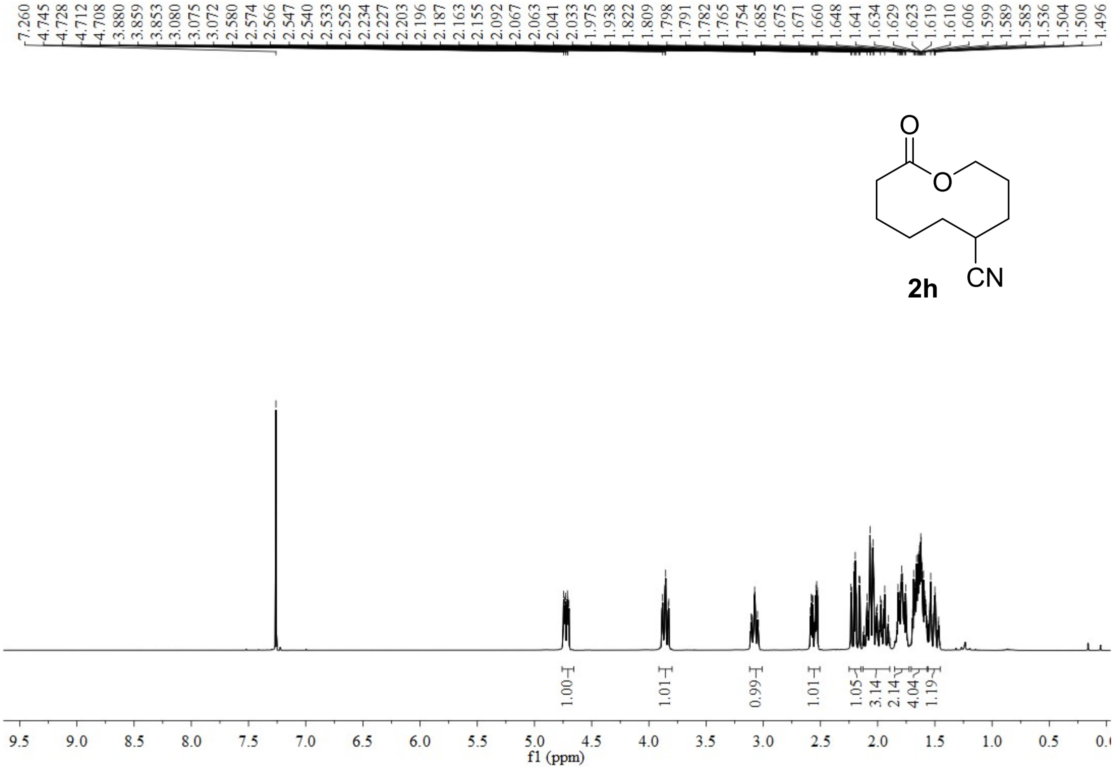

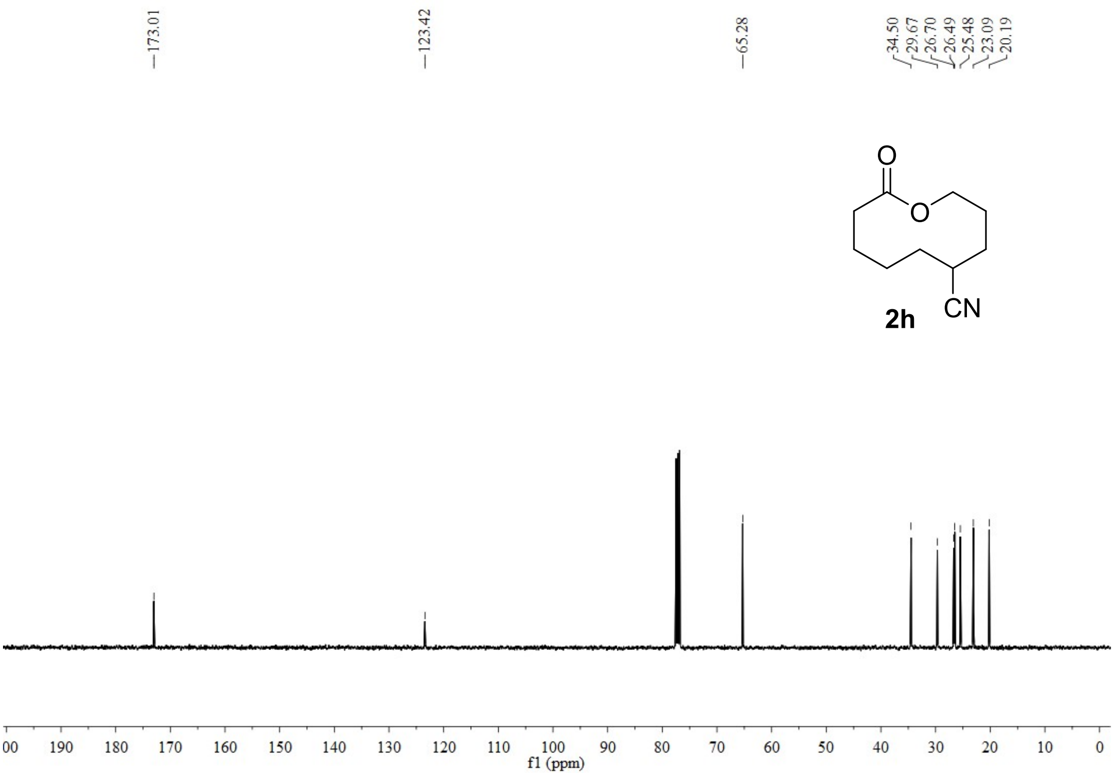

^{1}H NMR (400 MHz, $CDCl_3$) and ^{13}C NMR (100 MHz, $CDCl_3$) spectra of product **2i**

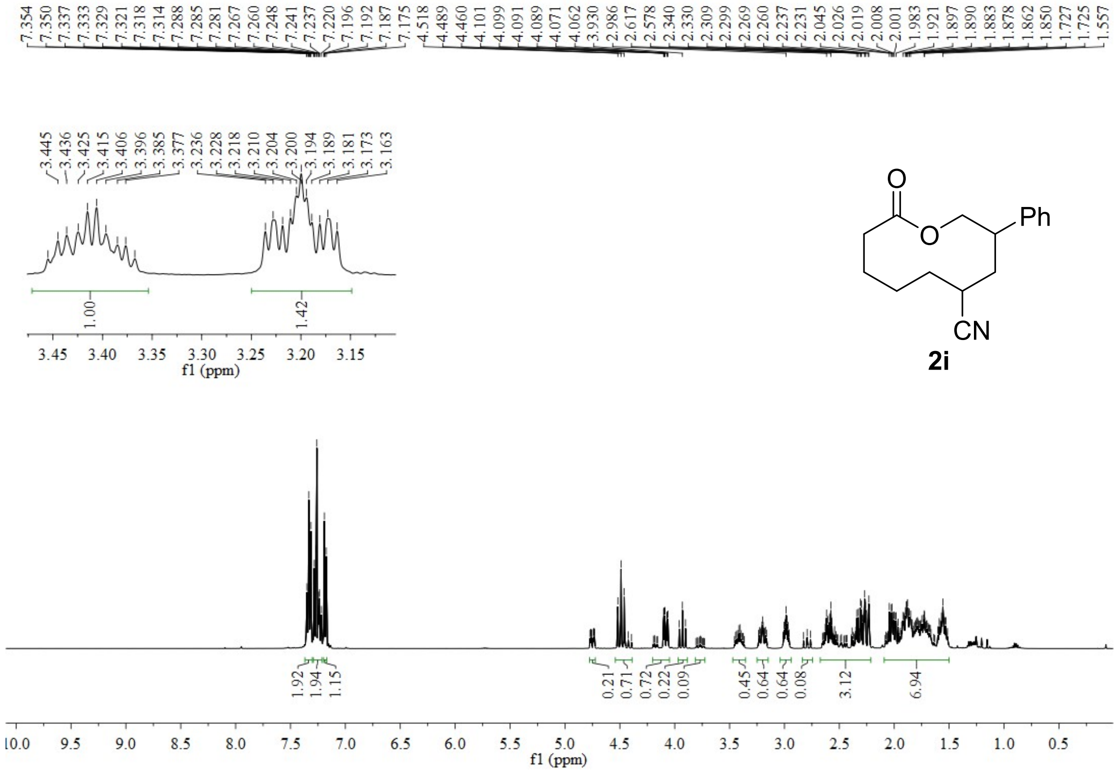

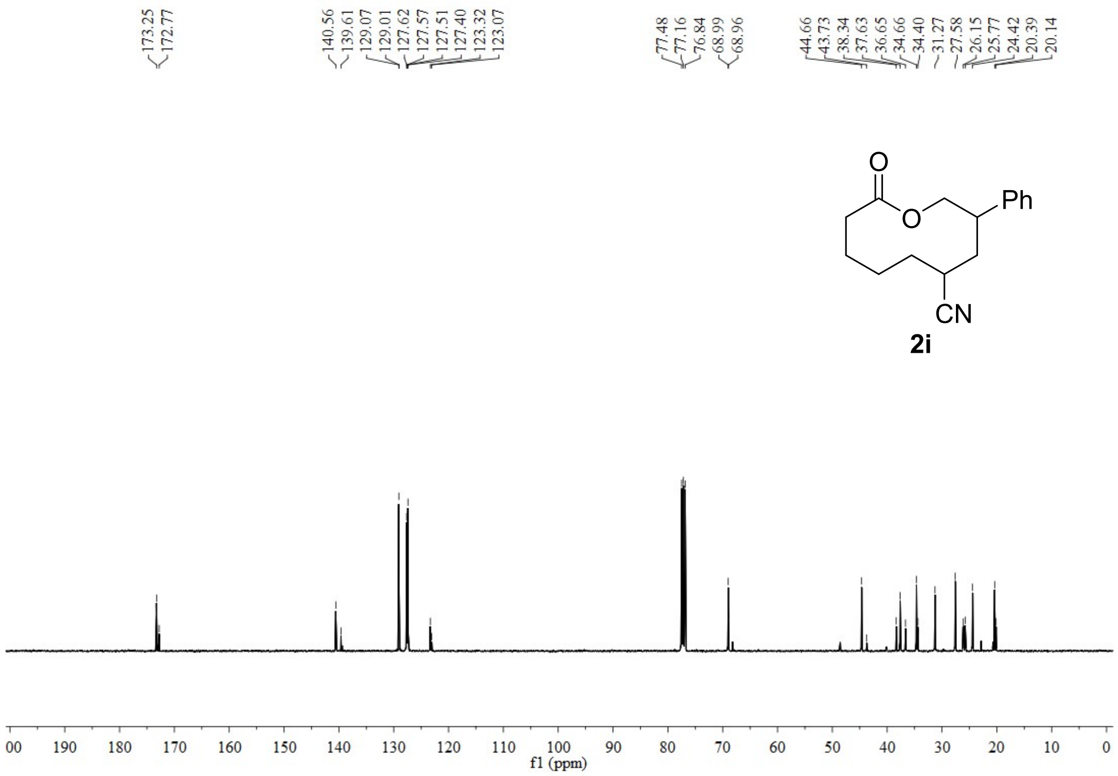

^{1}H NMR (400 MHz, $CDCl_3$) and ^{13}C NMR (100 MHz, $CDCl_3$) spectra of product **2j**

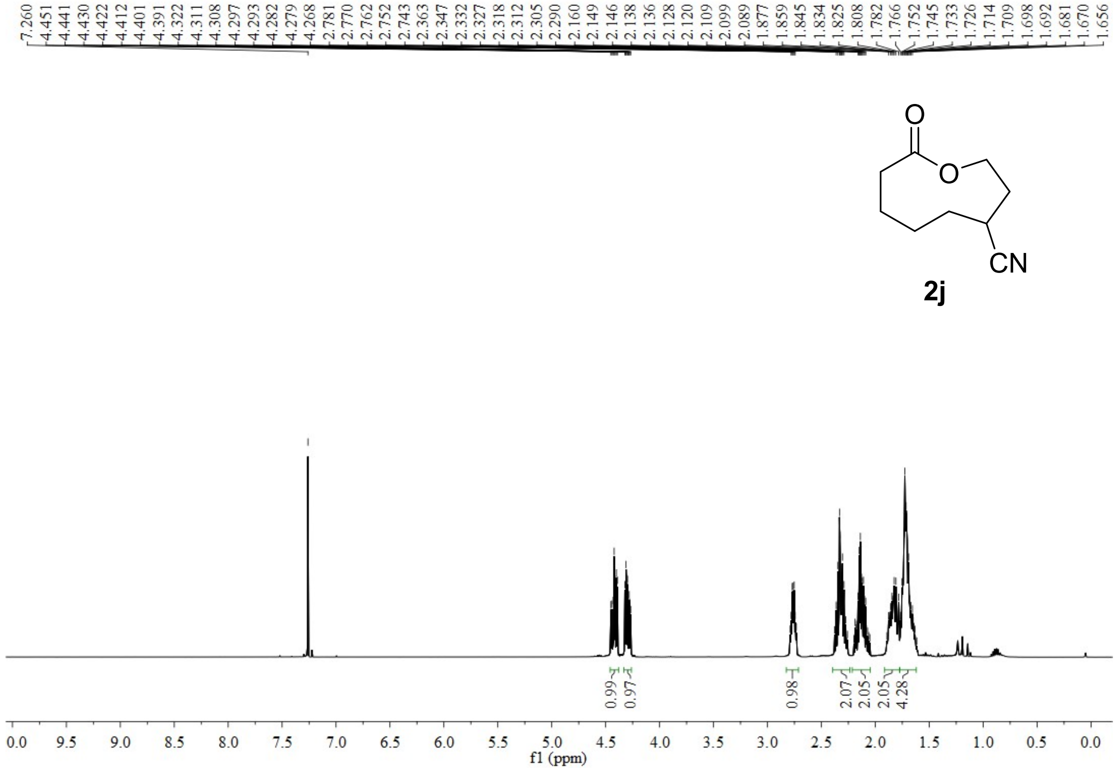

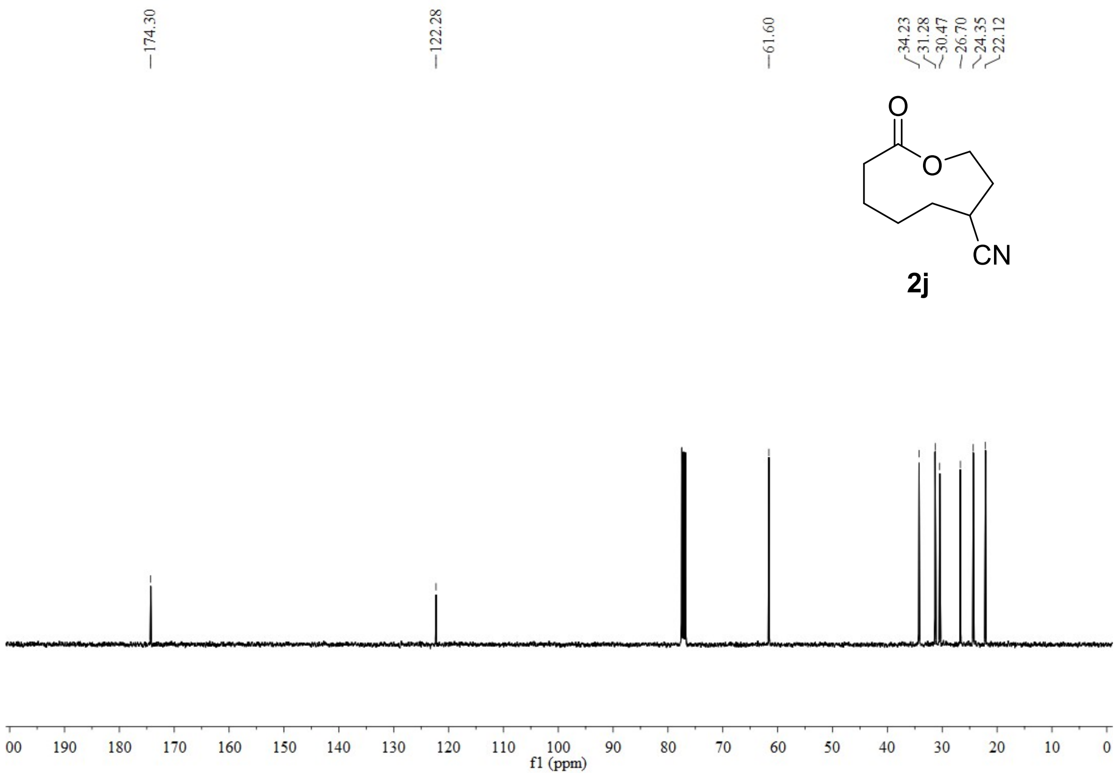

^{1}H NMR (400 MHz, $CDCl_3$) and ^{13}C NMR (100 MHz, $CDCl_3$) spectra of product **2k**

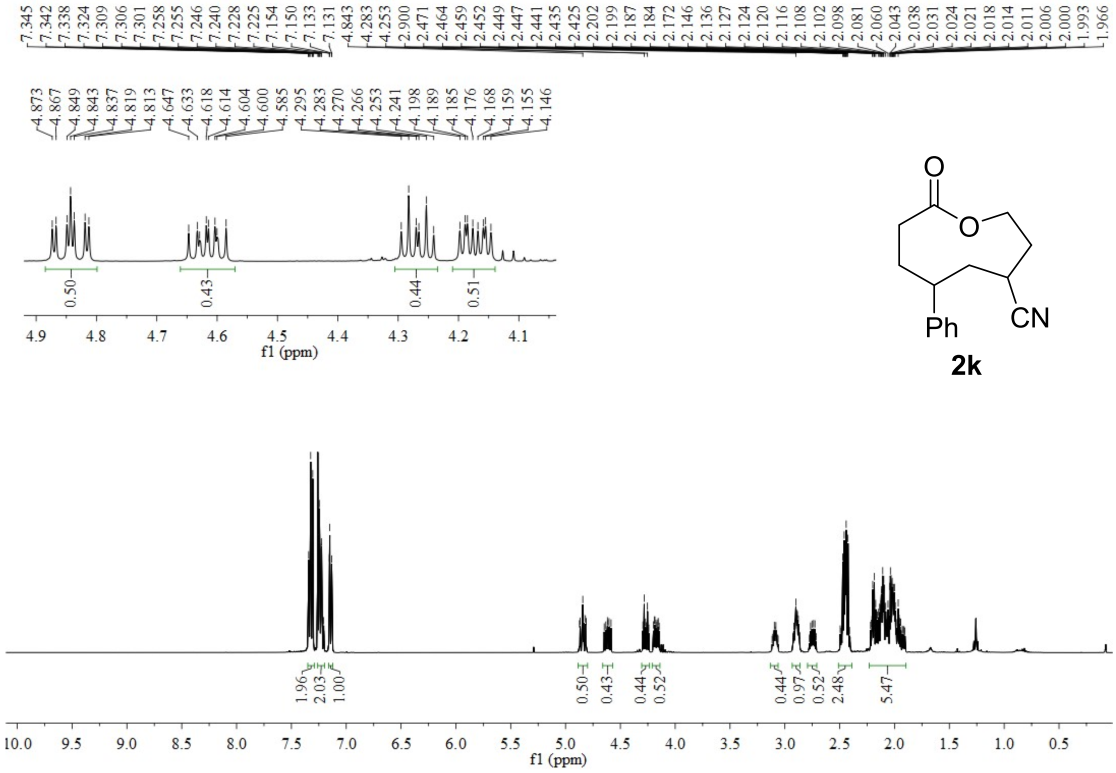

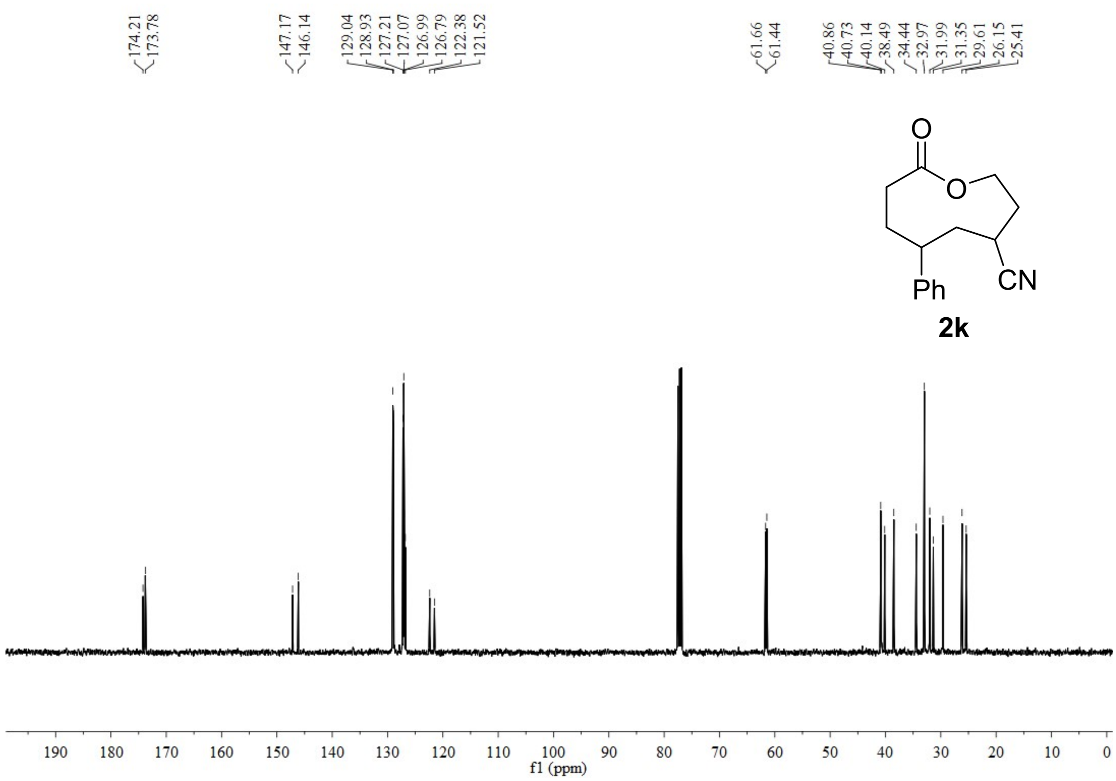

^{1}H NMR (400 MHz, $CDCl_3$) and ^{13}C NMR (100 MHz, $CDCl_3$) spectra of product **2l**

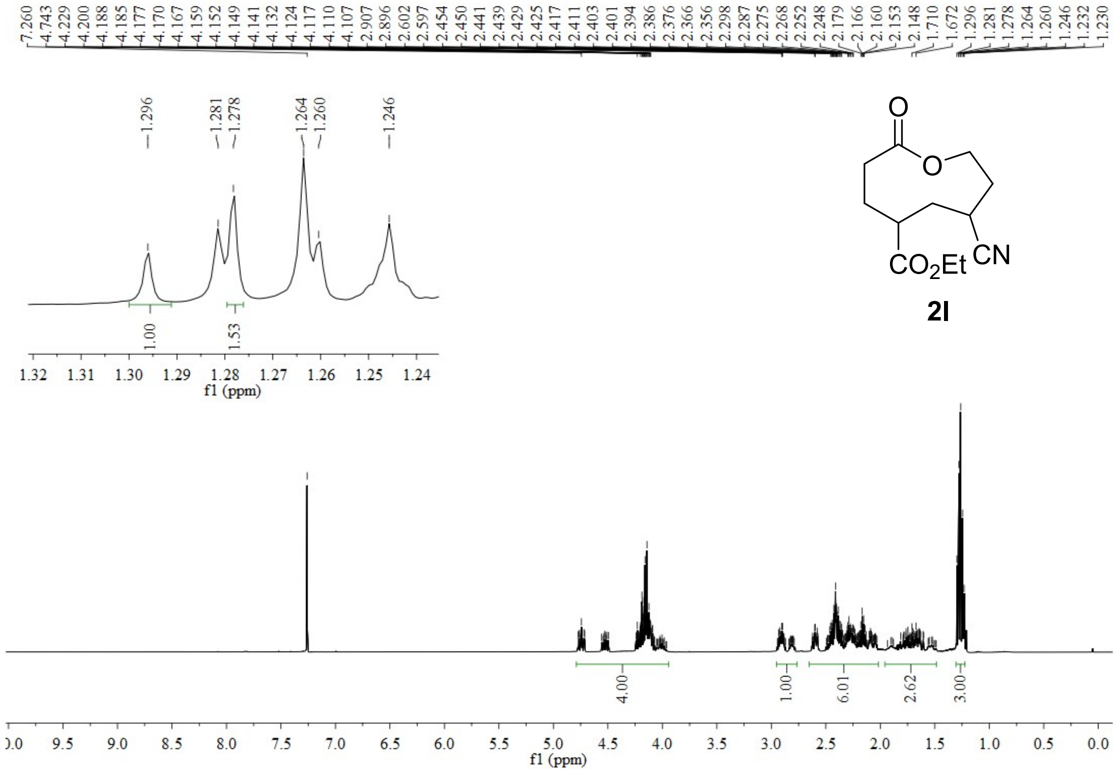

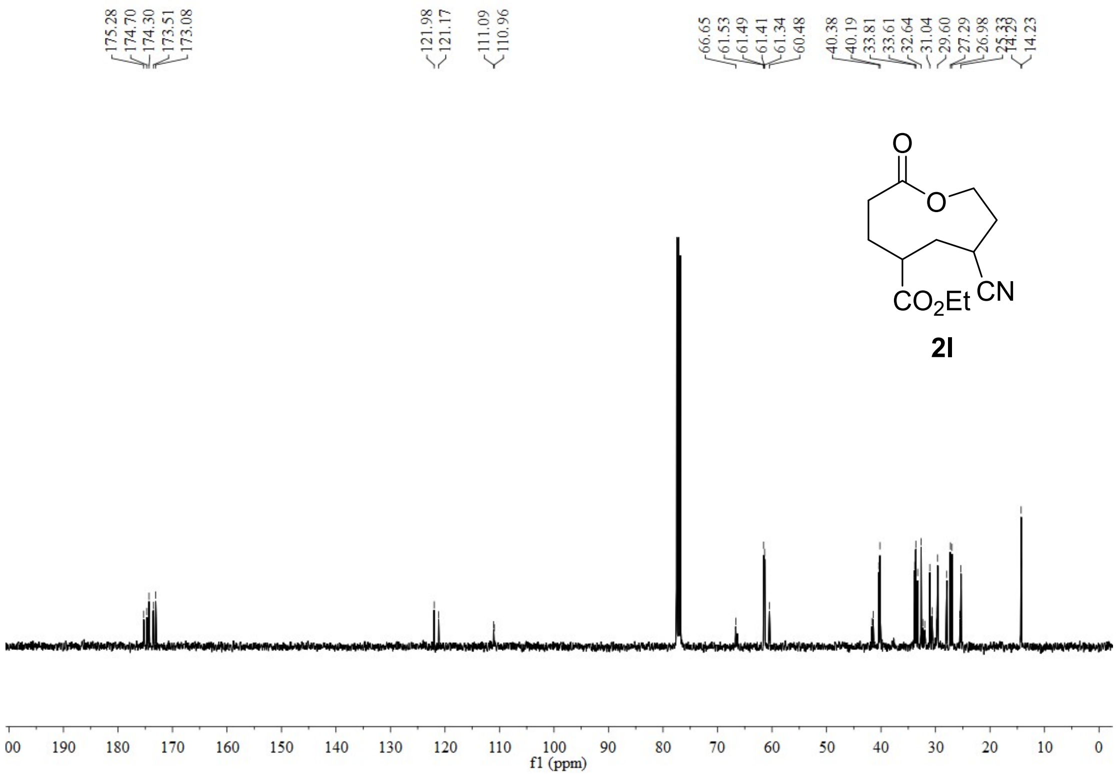

^{1}H NMR (400 MHz, $CDCl_3$) and ^{13}C NMR (100 MHz, $CDCl_3$) spectra of product **2m**

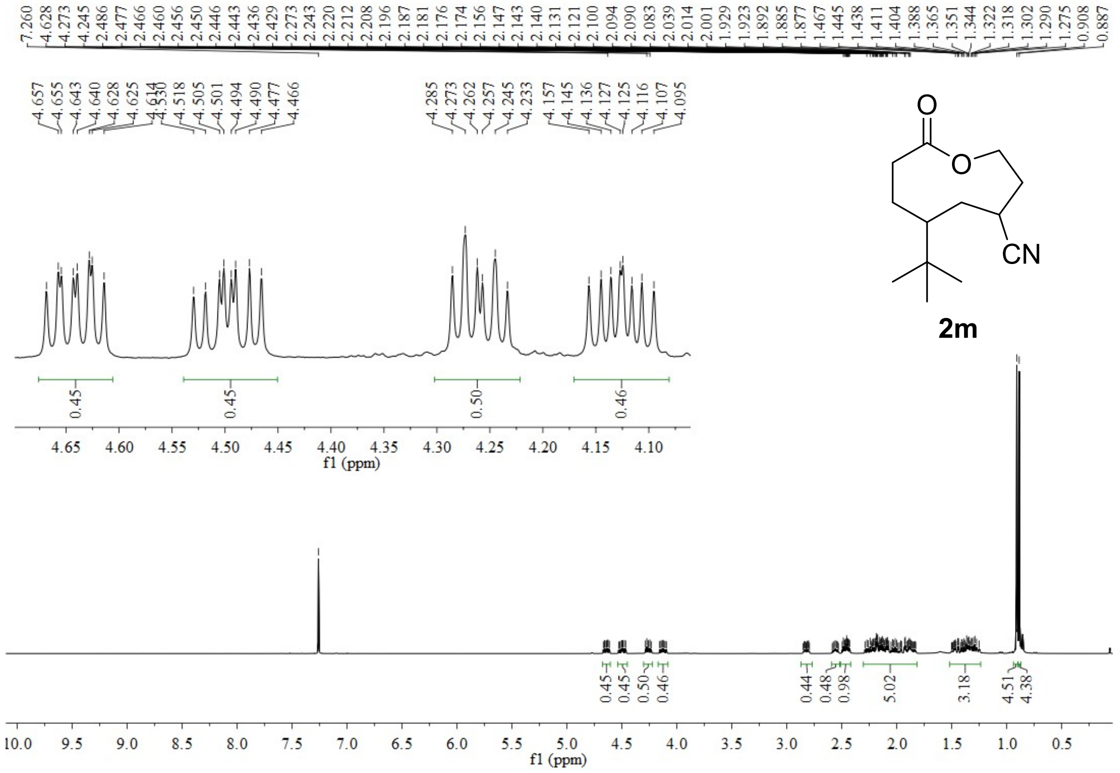

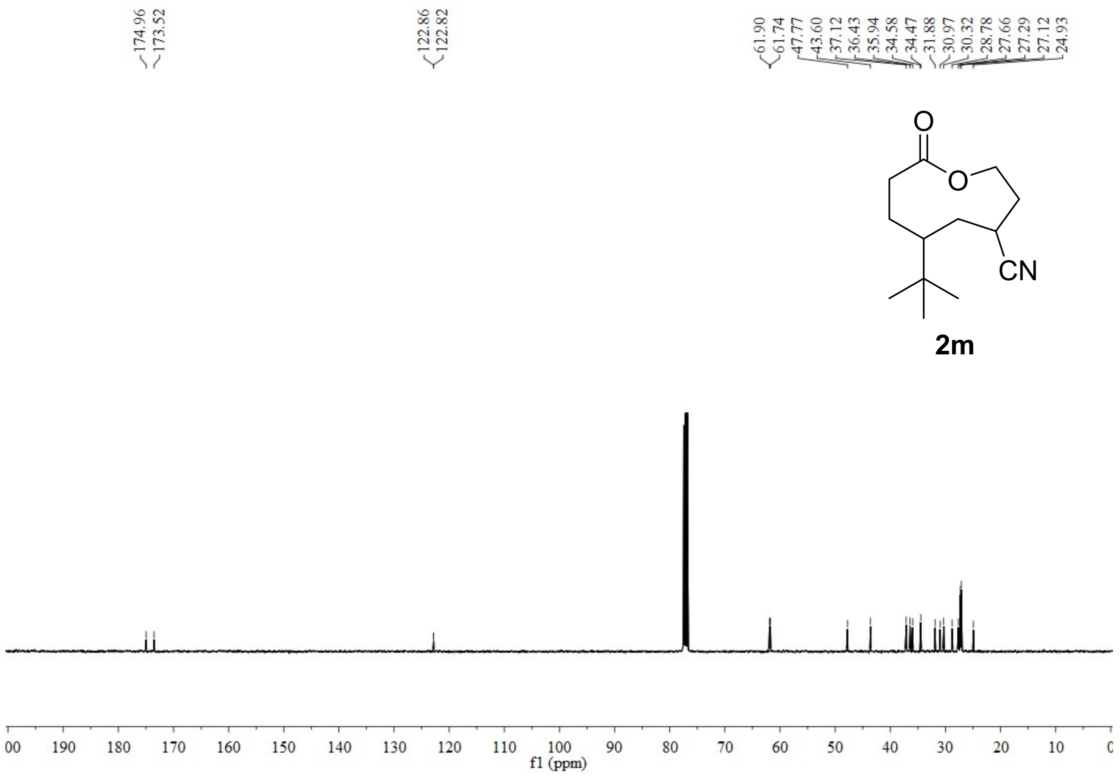

^{1}H NMR (400 MHz, $CDCl_3$) and ^{13}C NMR (100 MHz, $CDCl_3$) spectra of product **2n**

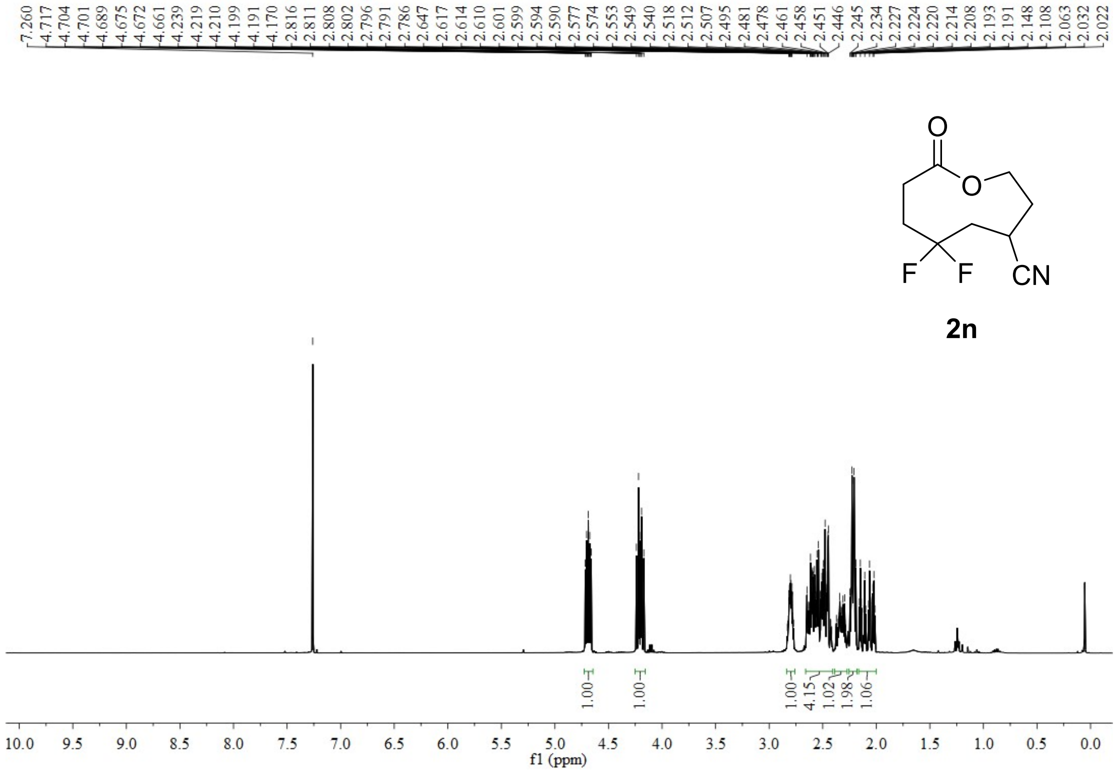

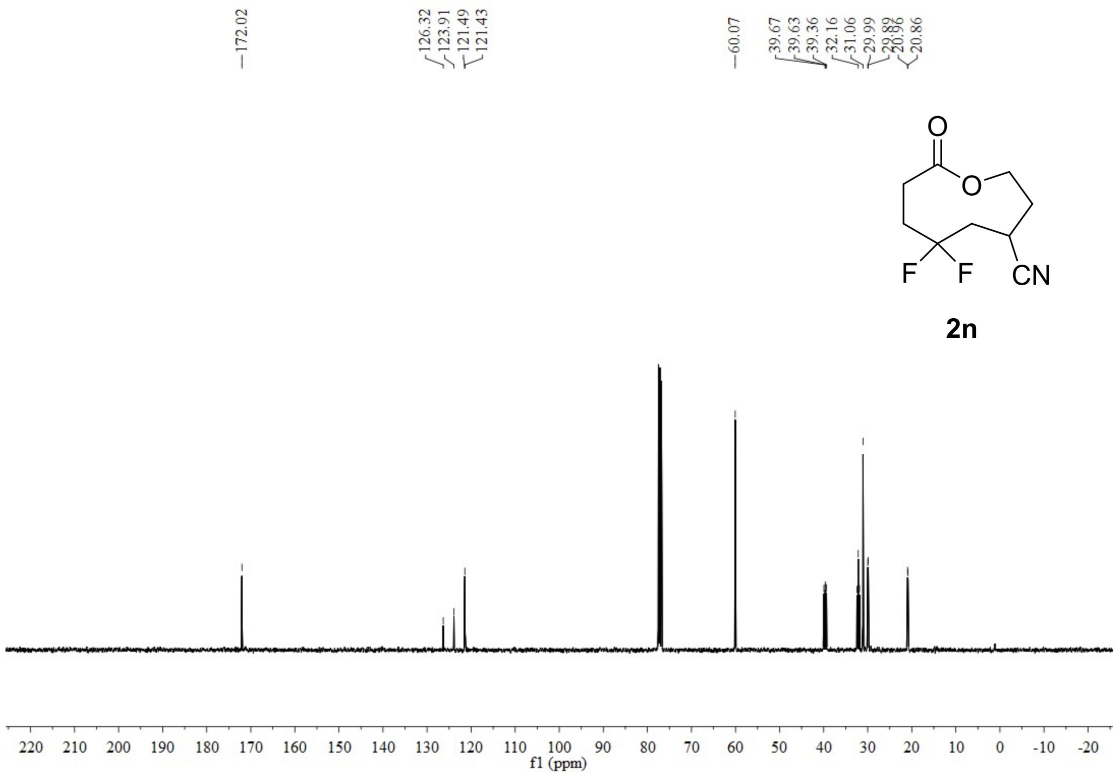

^{1}H NMR (400 MHz, $CDCl_3$) and ^{13}C NMR (100 MHz, $CDCl_3$) spectra of product **2o**

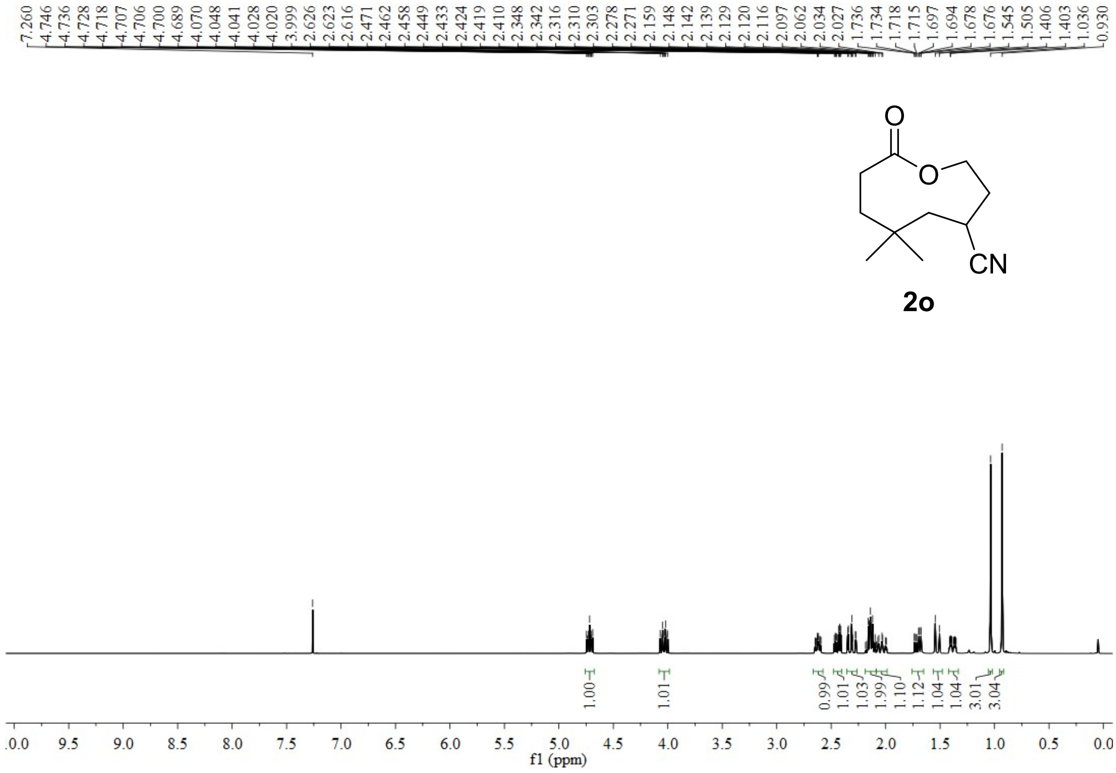

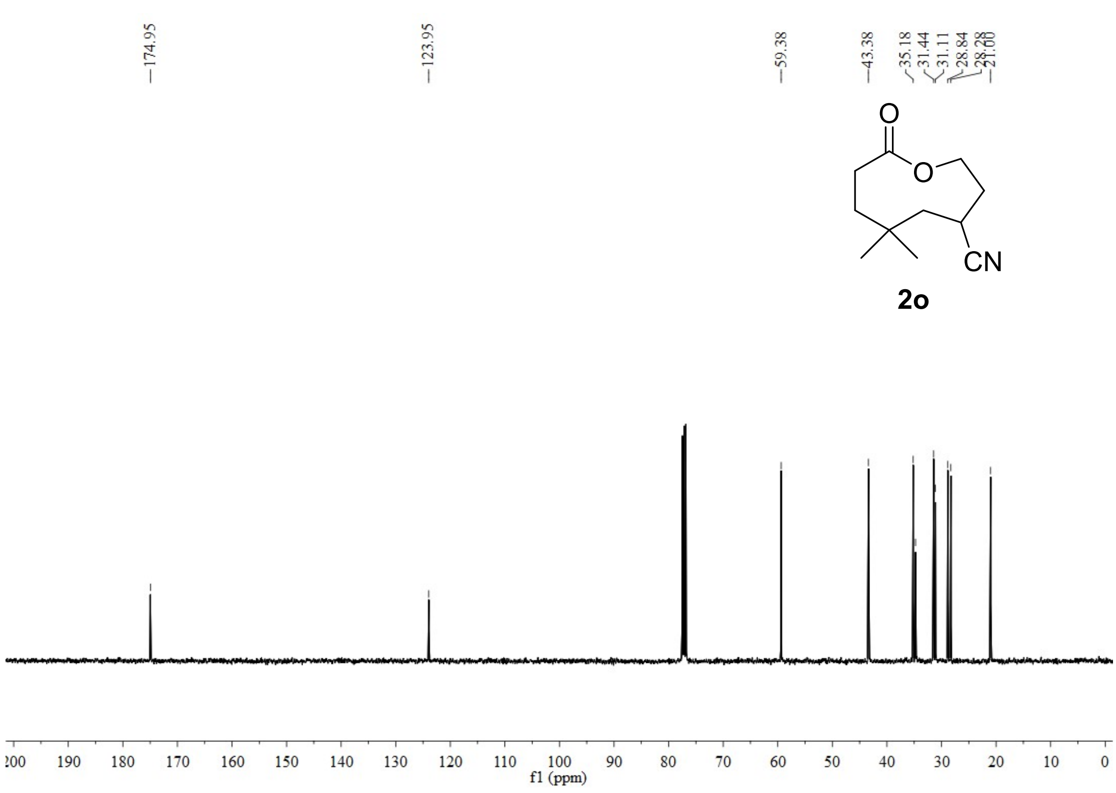

^1H NMR (400 MHz, $CDCl_3$) and ^{13}C NMR (100 MHz, $CDCl_3$) spectra of product **2p**

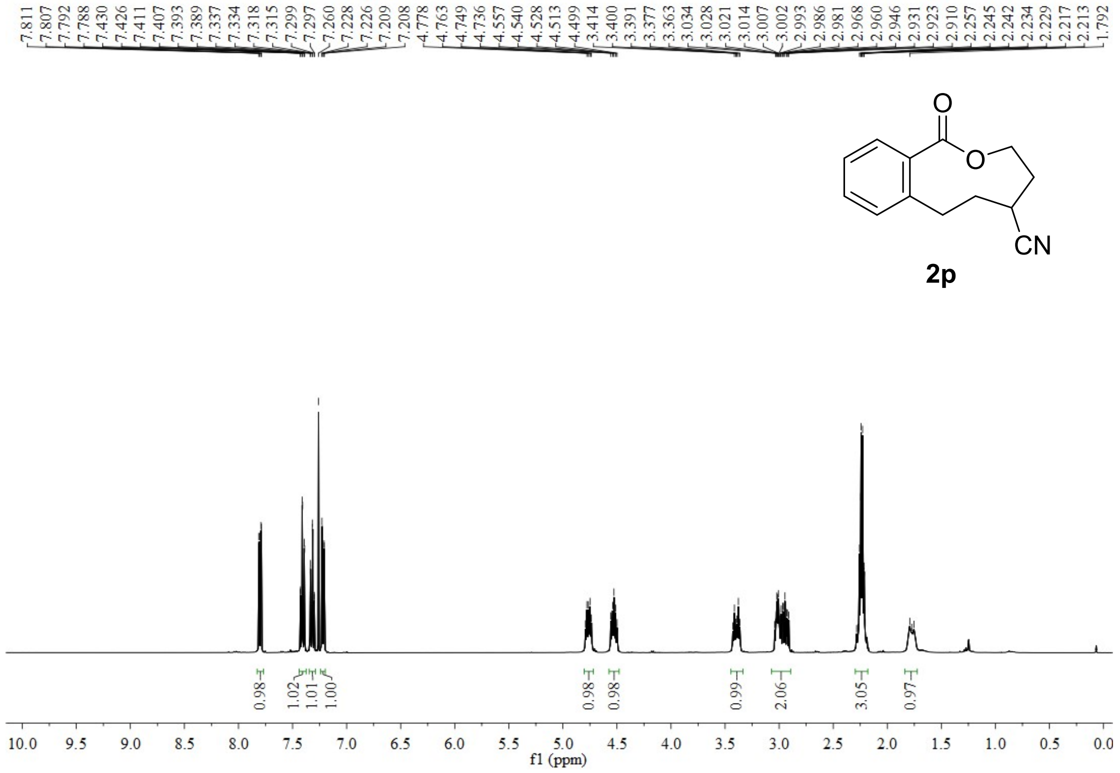

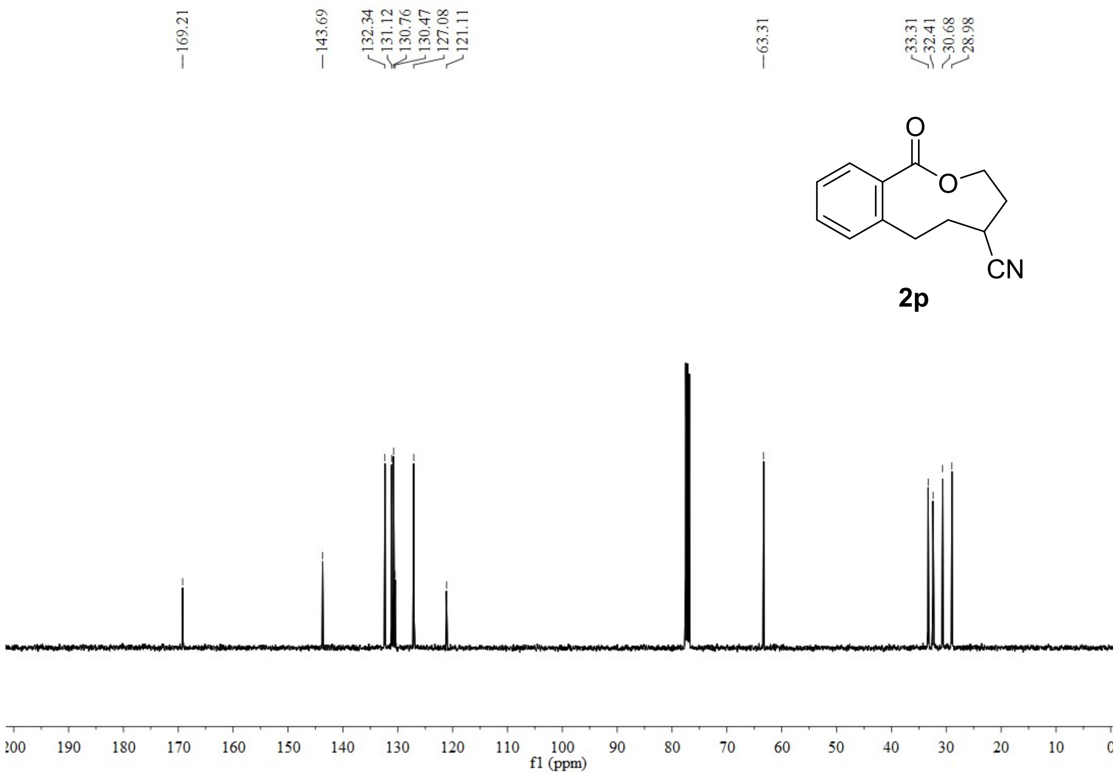

^{1}H NMR (400 MHz, $CDCl_3$) and ^{13}C NMR (100 MHz, $CDCl_3$) spectra of product **2q**

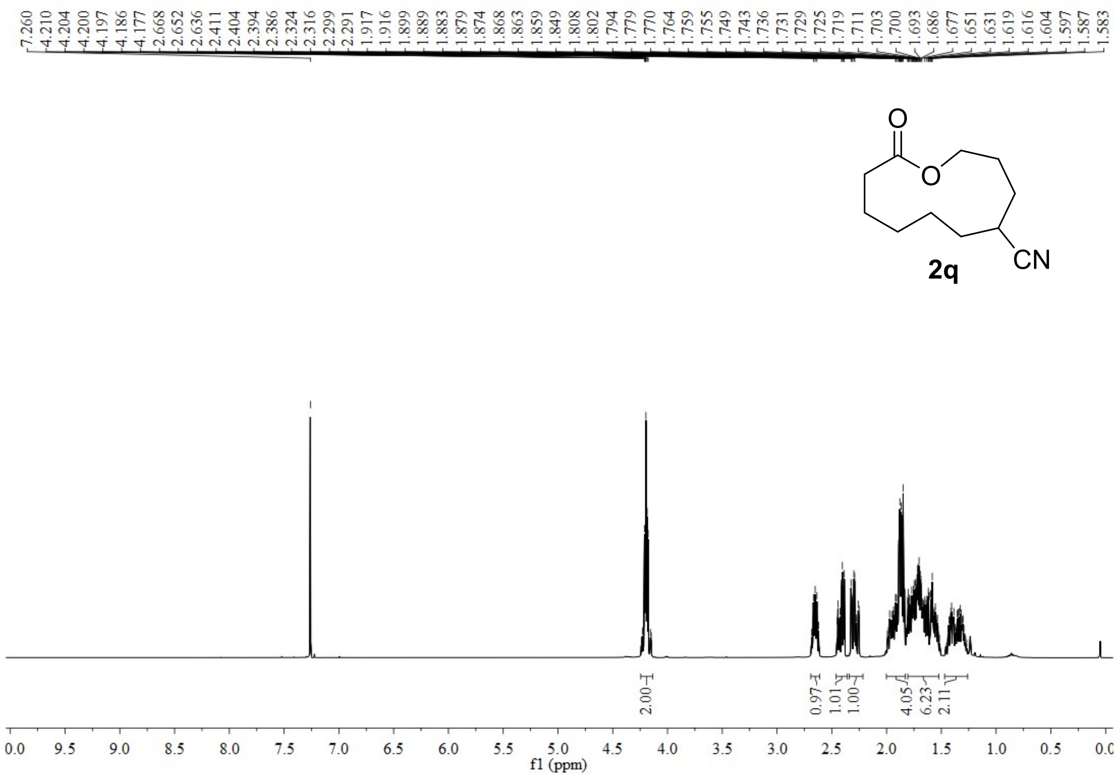

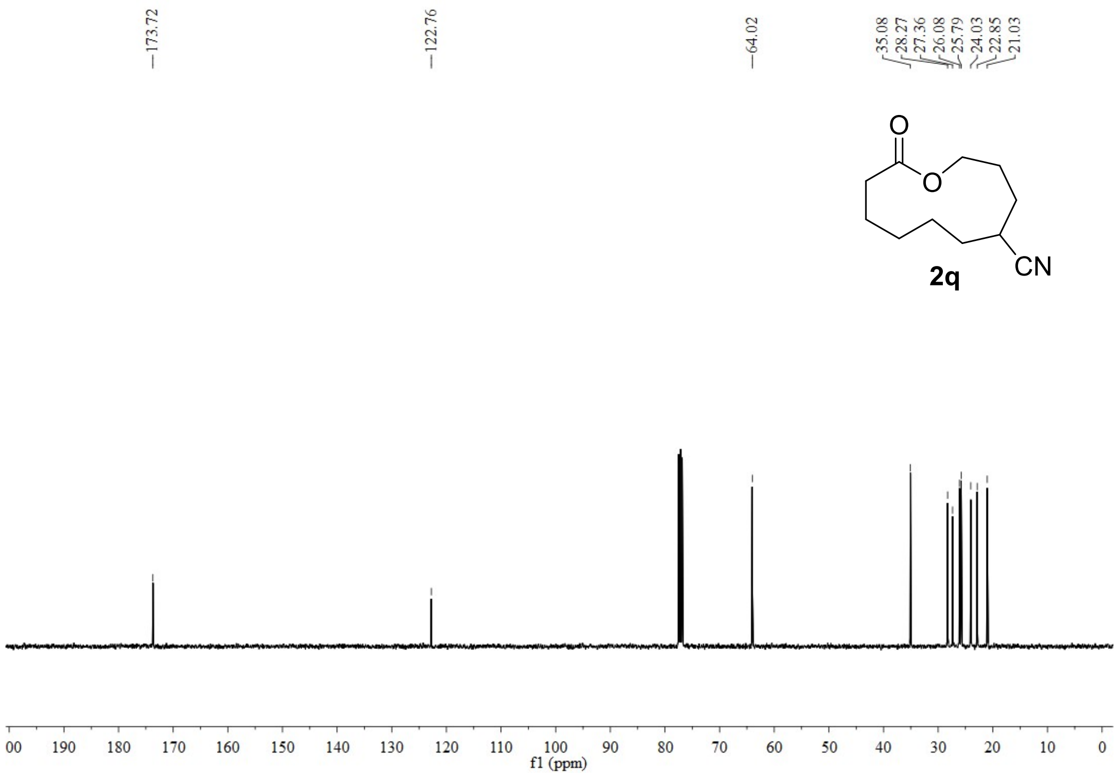

^{1}H NMR (400 MHz, $CDCl_3$) and ^{13}C NMR (100 MHz, $CDCl_3$) spectra of product **2r**

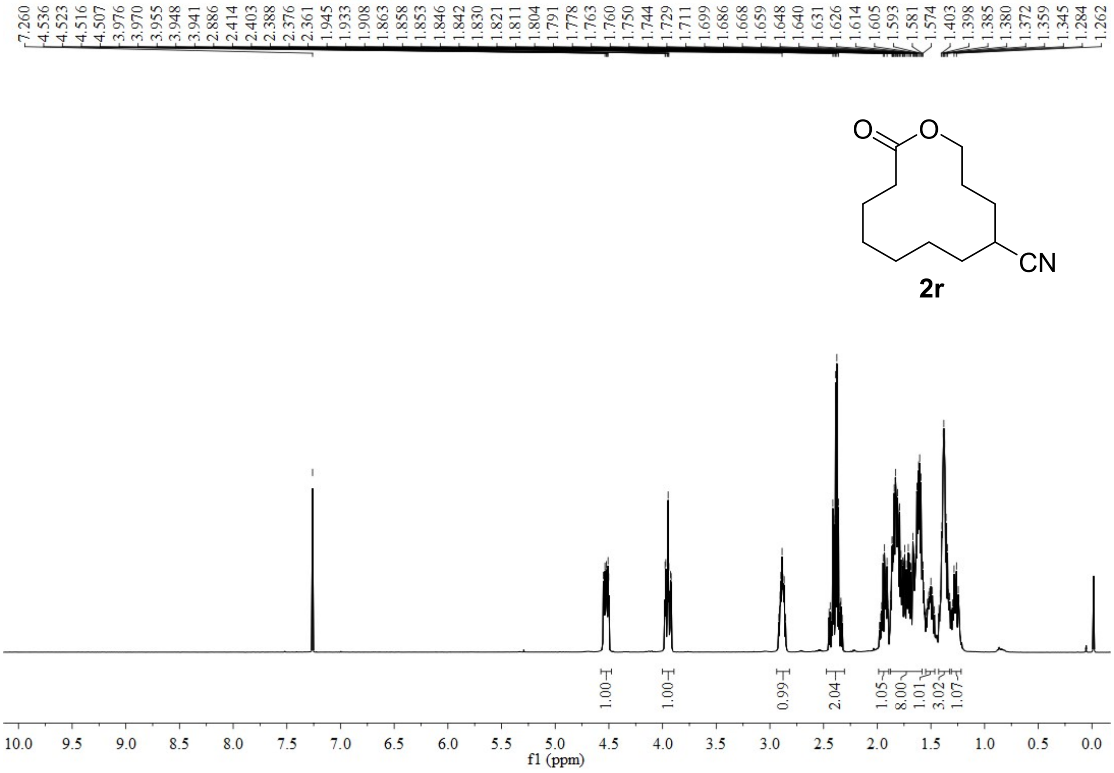

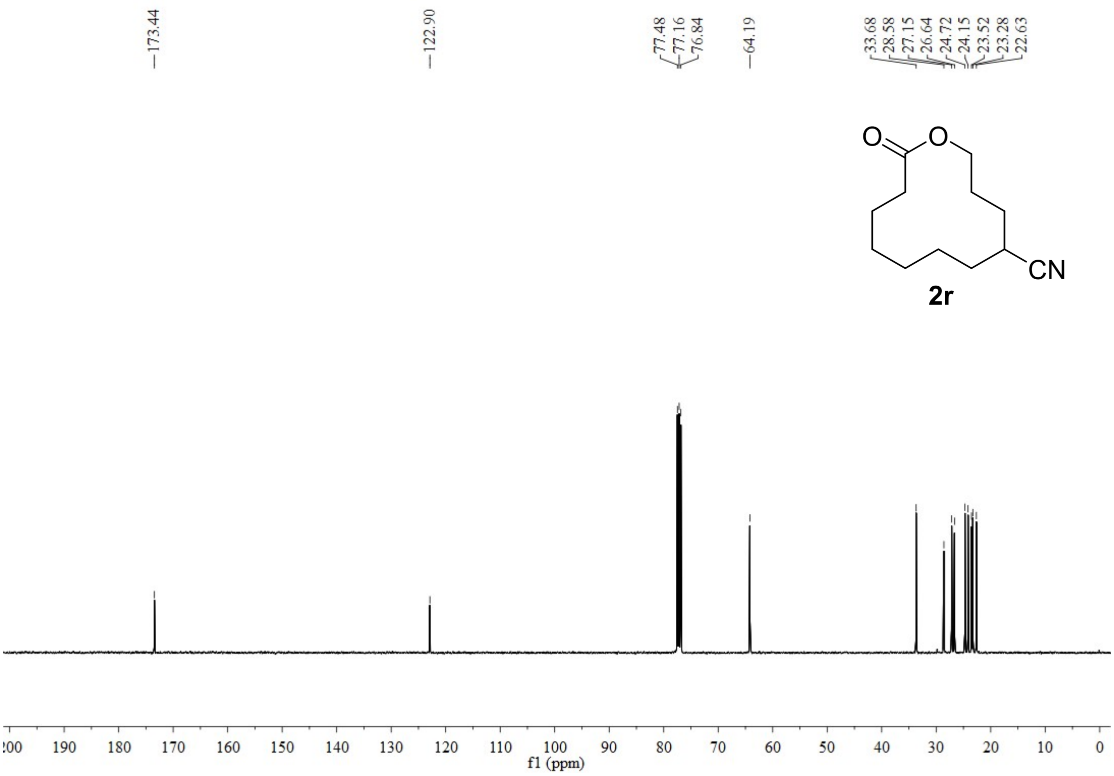

^{1}H NMR (400 MHz, $CDCl_3$) and ^{13}C NMR (100 MHz, $CDCl_3$) spectra of product **2s**

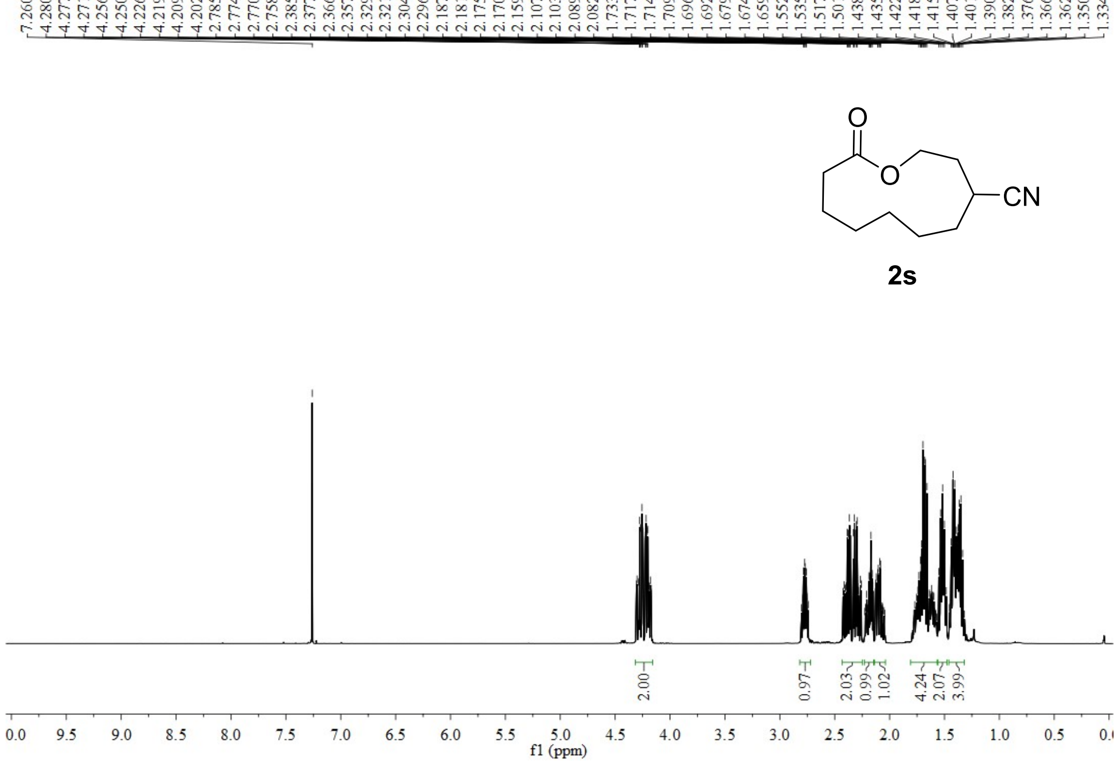

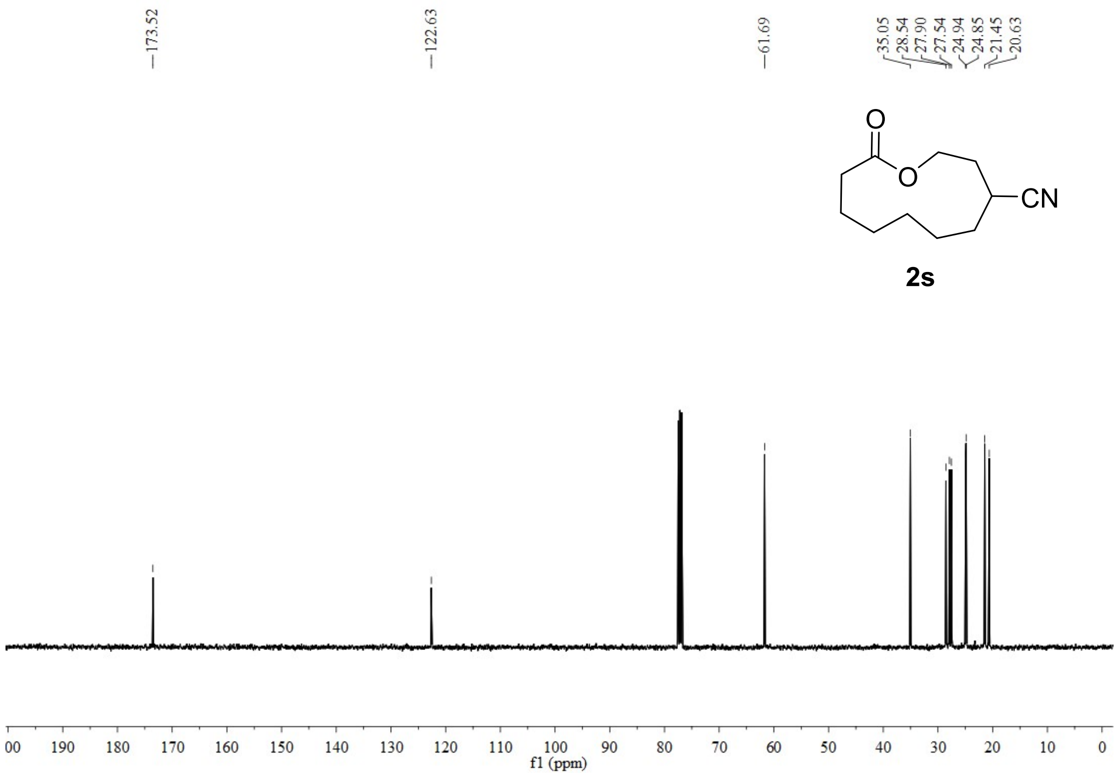

^{1}H NMR (400 MHz, $CDCl_3$) and ^{13}C NMR (100 MHz, $CDCl_3$) spectra of product **2t**

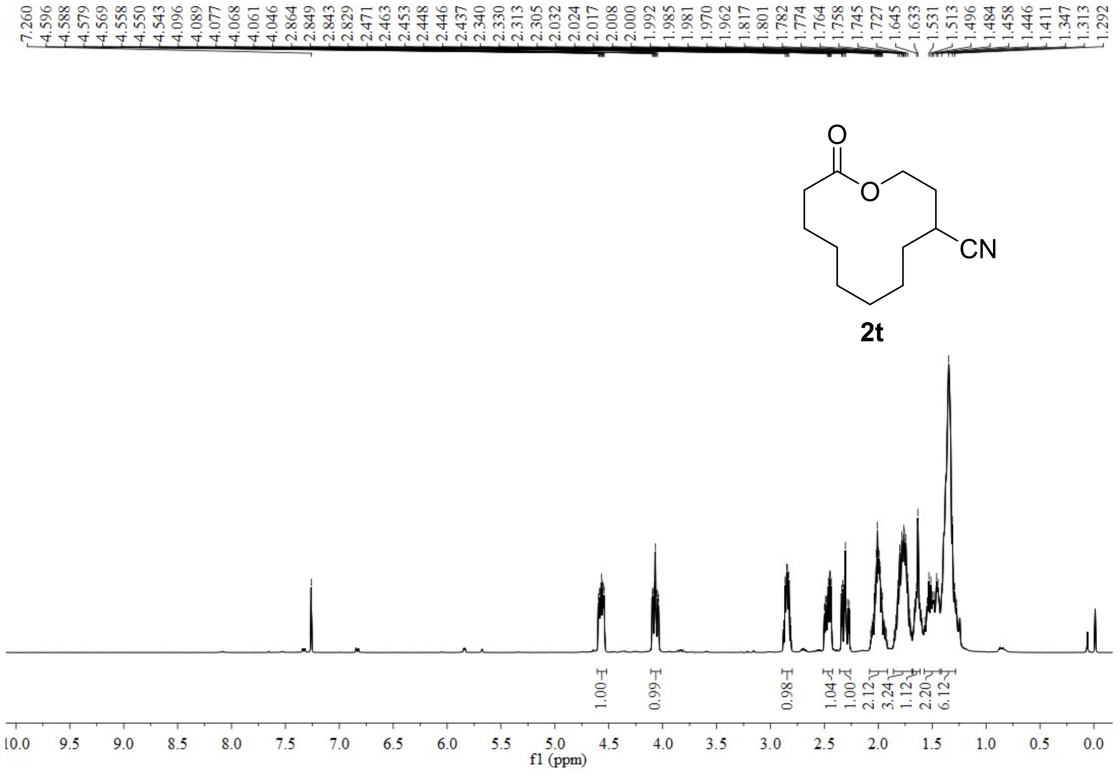

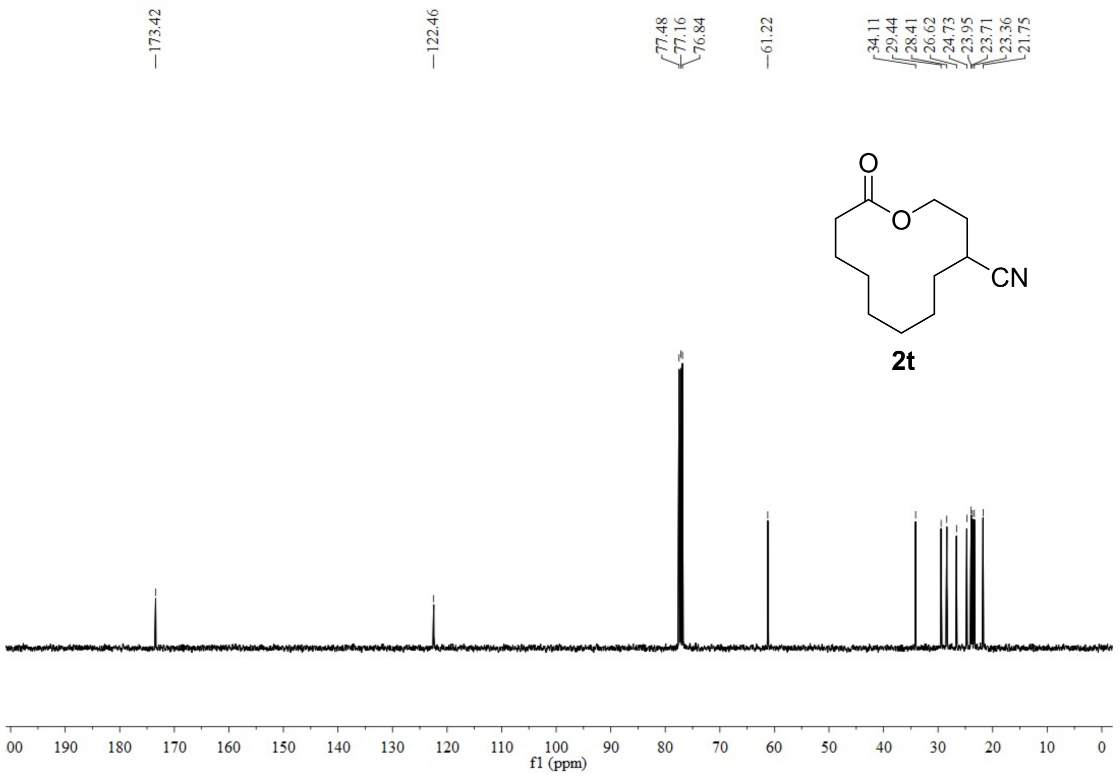

^{1}H NMR (400 MHz, $CDCl_3$) and ^{13}C NMR (100 MHz, $CDCl_3$) spectra of product **2u**

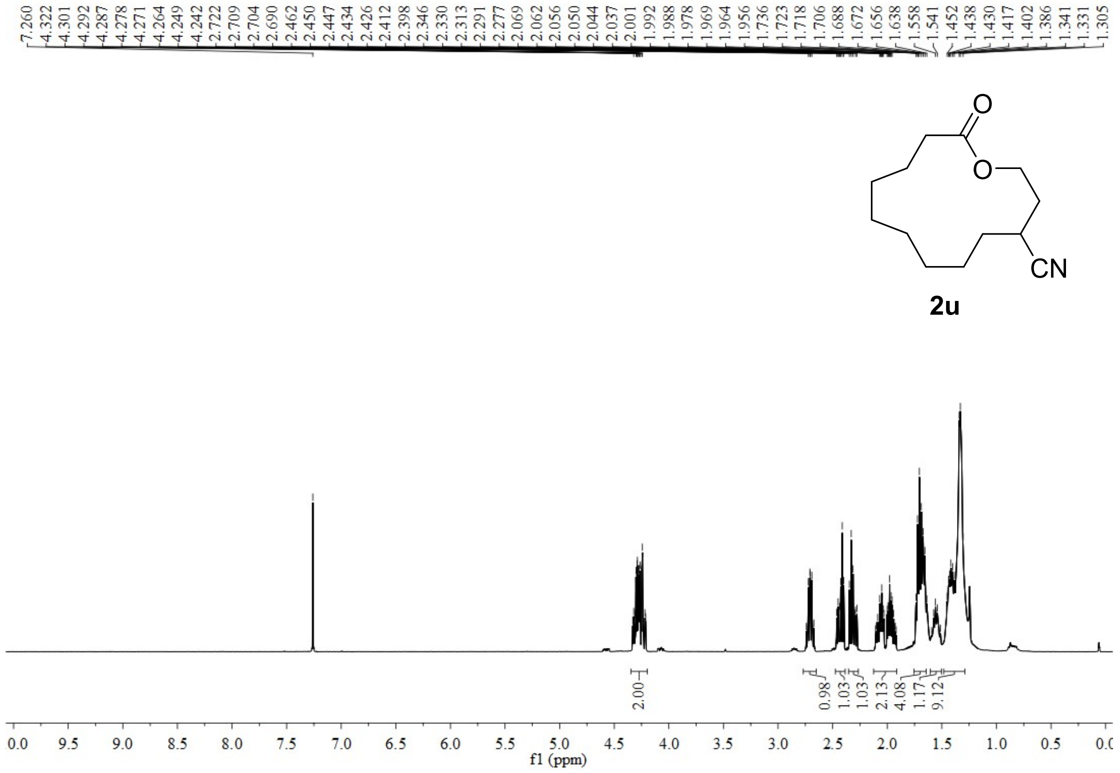

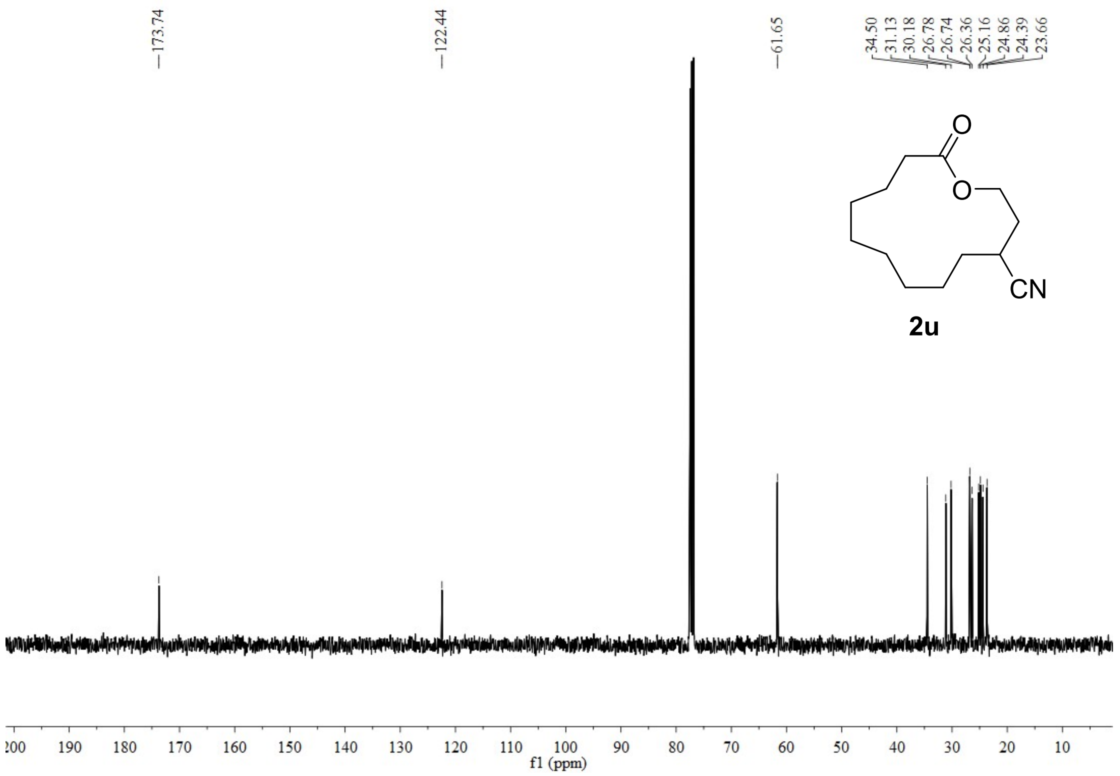

^{1}H NMR (400 MHz, $CDCl_3$) and ^{13}C NMR (100 MHz, $CDCl_3$) spectra of product **2v**

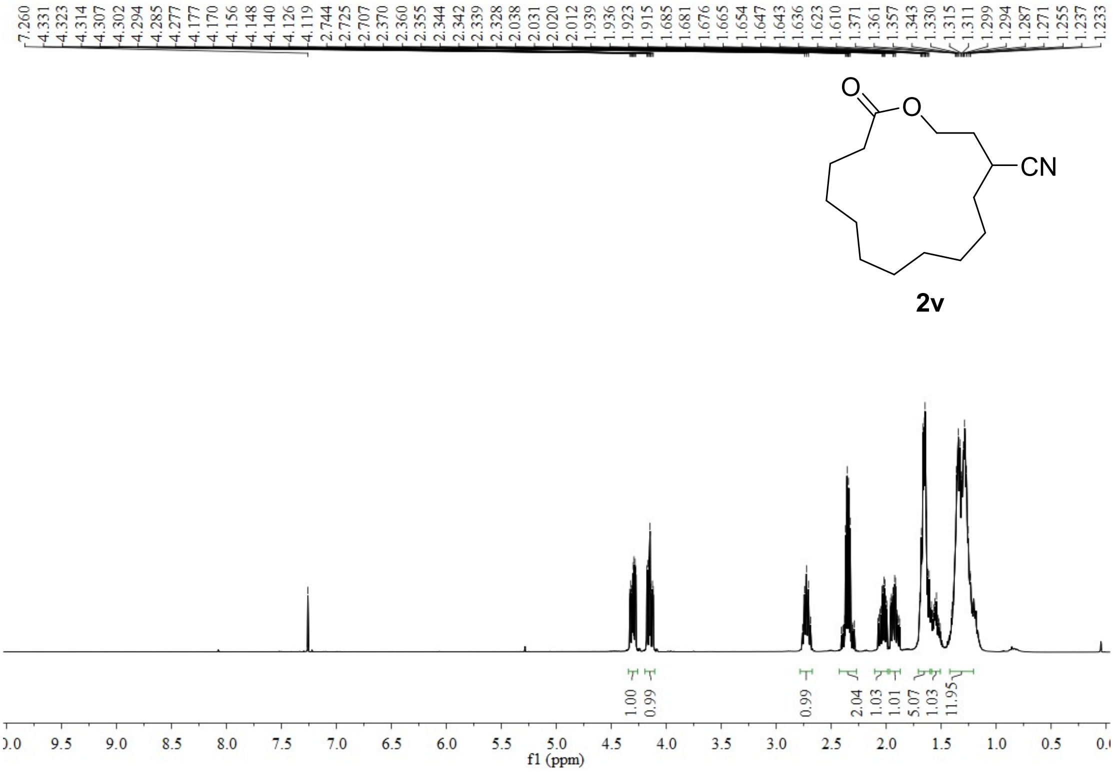

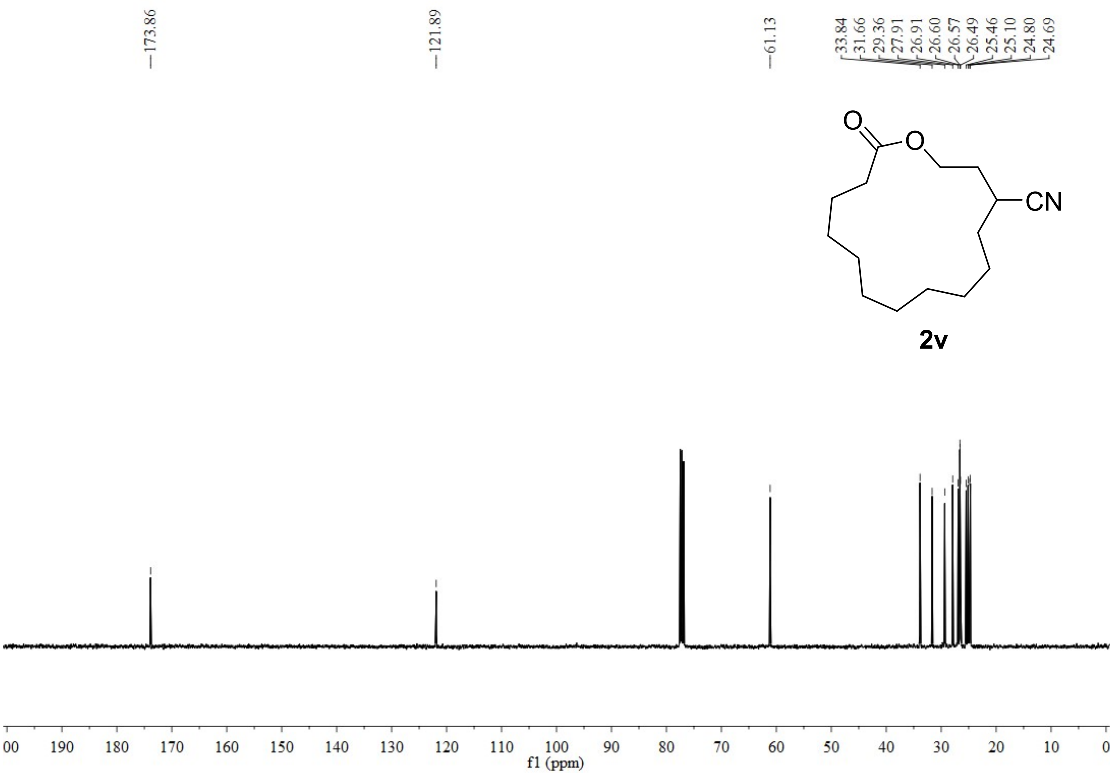

^{1}H NMR (400 MHz, $CDCl_3$) and ^{13}C NMR (100 MHz, $CDCl_3$) spectra of product **2w**

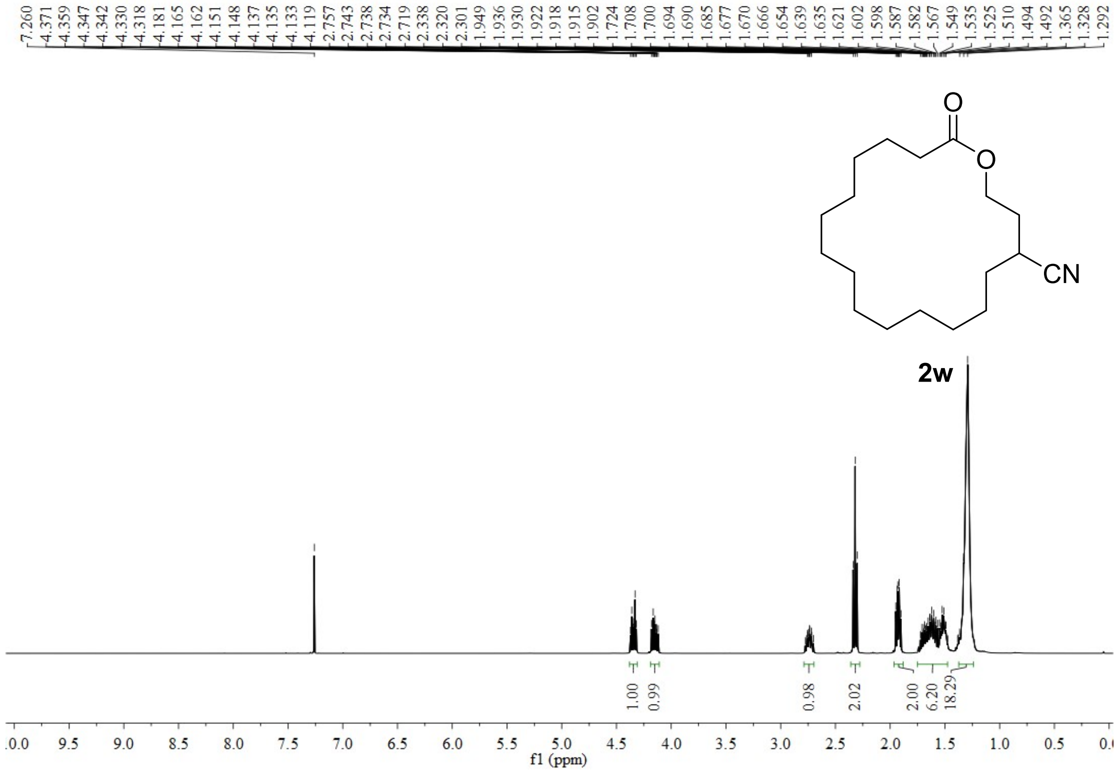

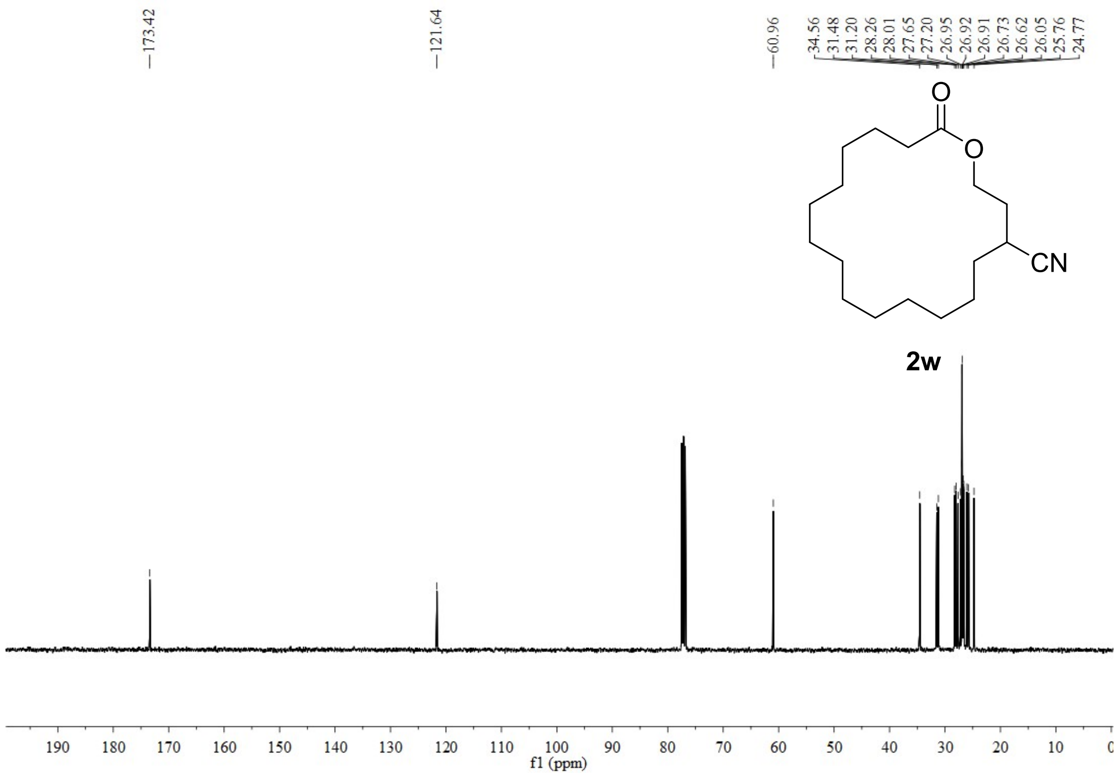

^{1}H NMR (400 MHz, $CDCl_3$) and ^{13}C NMR (100 MHz, $CDCl_3$) spectra of product **2x**

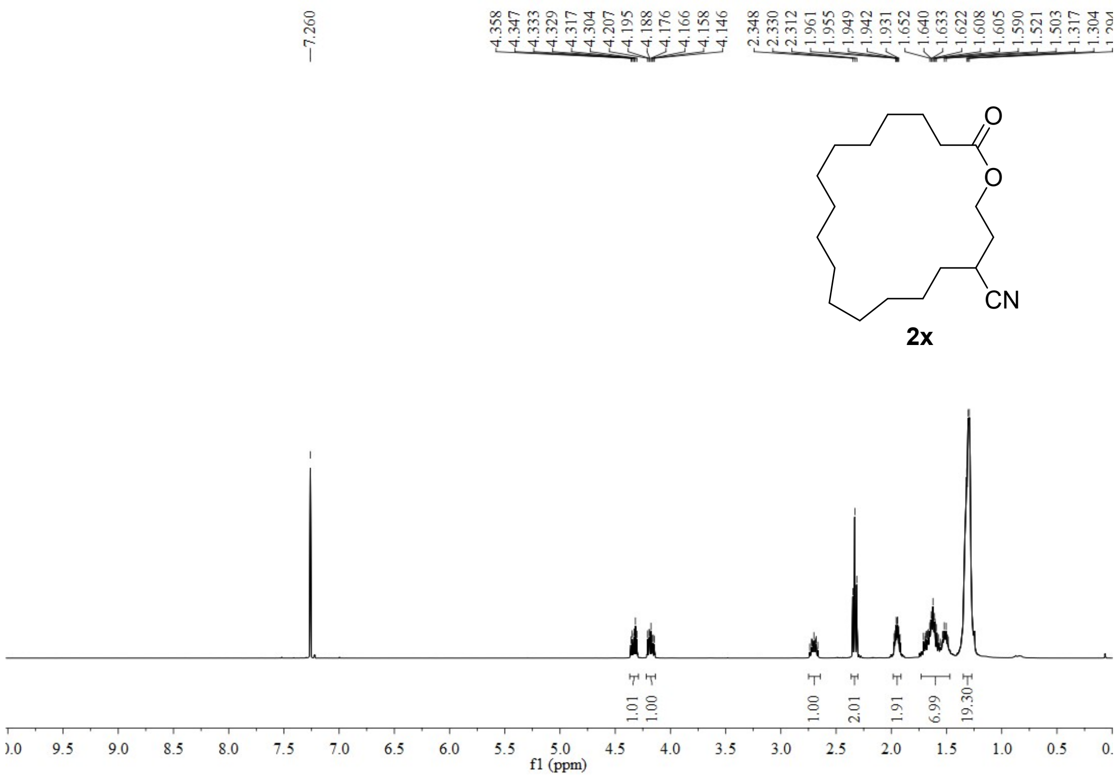

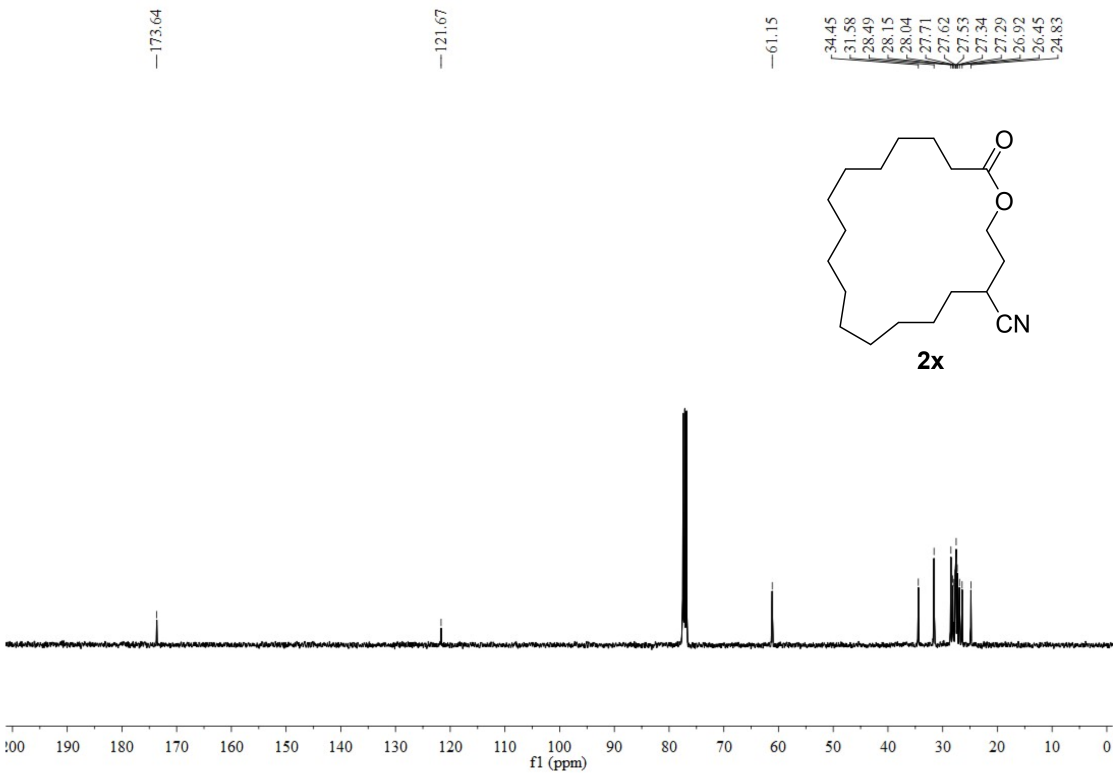

^{1}H NMR (400 MHz, $CDCl_3$) and ^{13}C NMR (100 MHz, $CDCl_3$) spectra of product **2y**

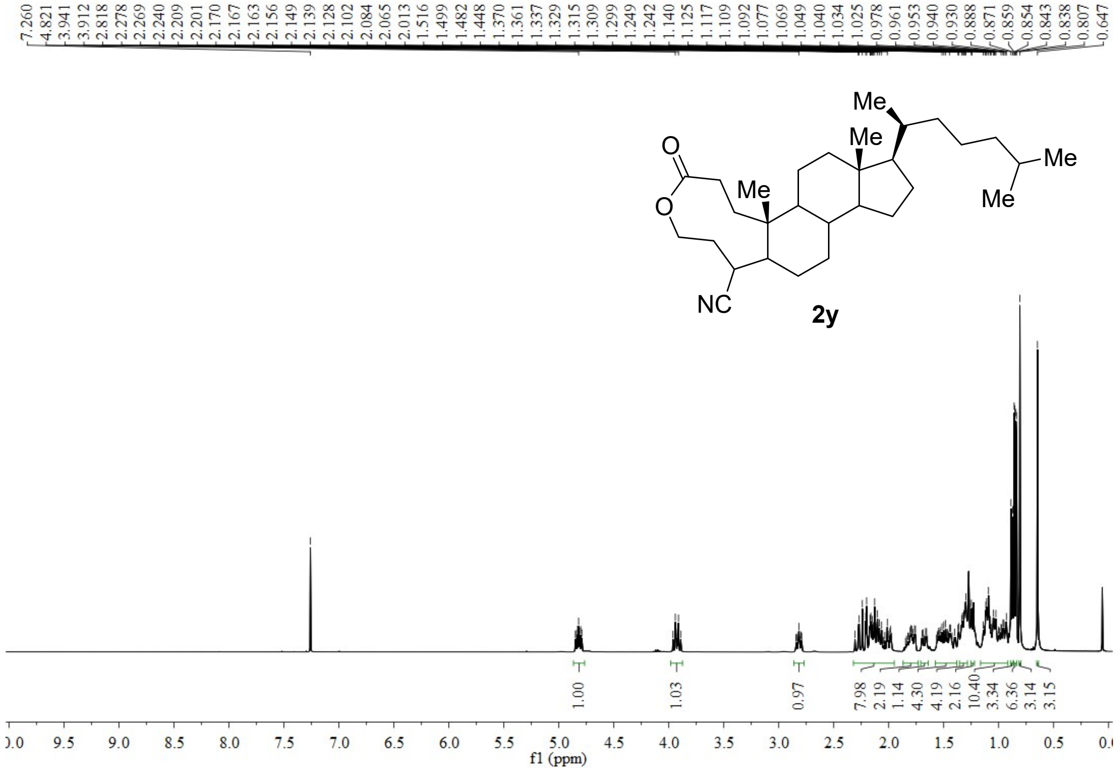

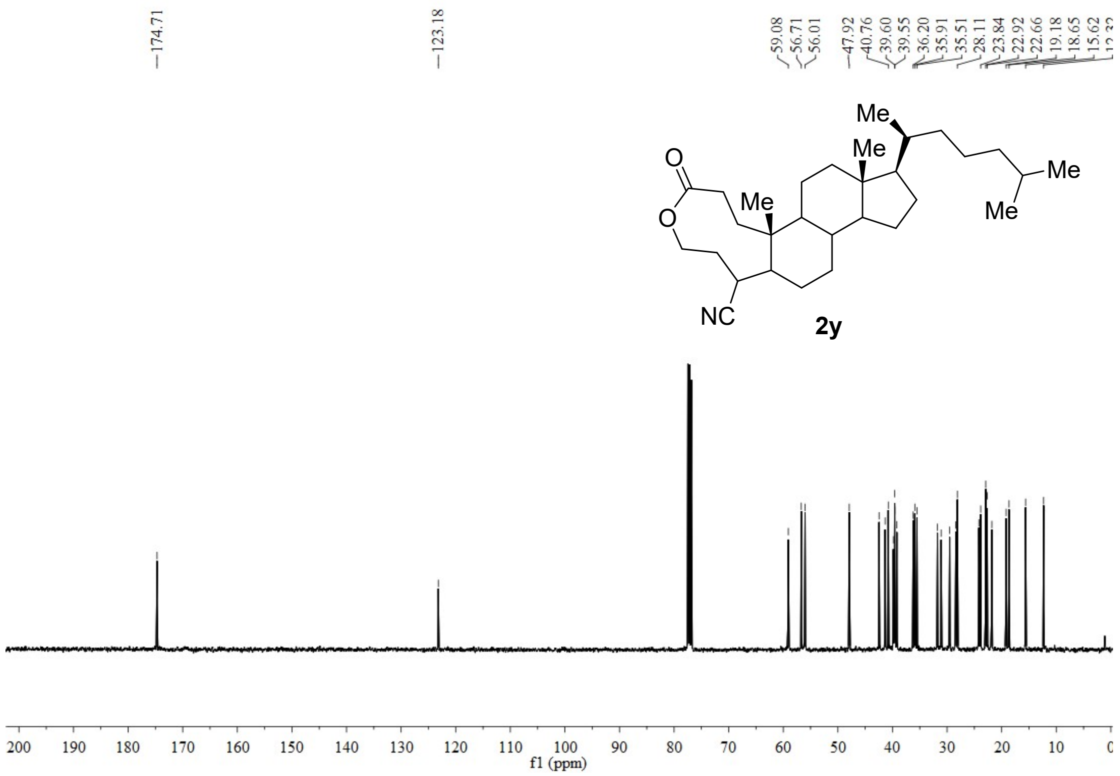

^1H NMR (400 MHz, $CDCl_3$) and ^{13}C NMR (100 MHz, $CDCl_3$) spectra of product **3a**

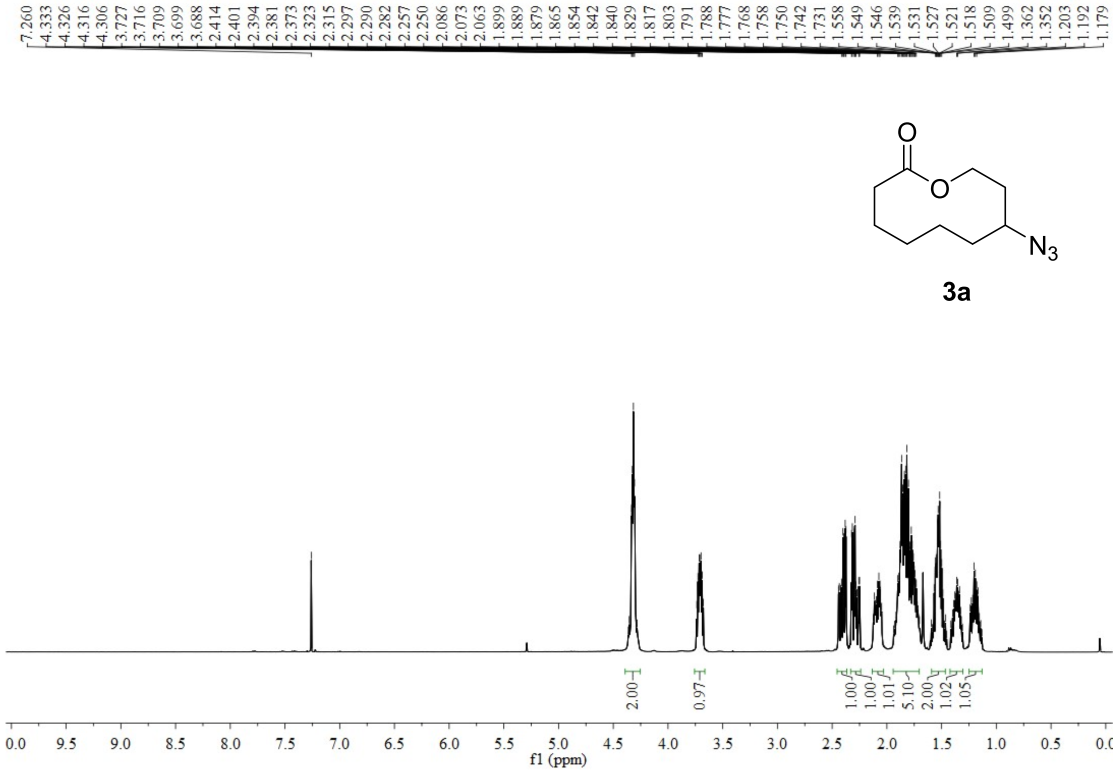

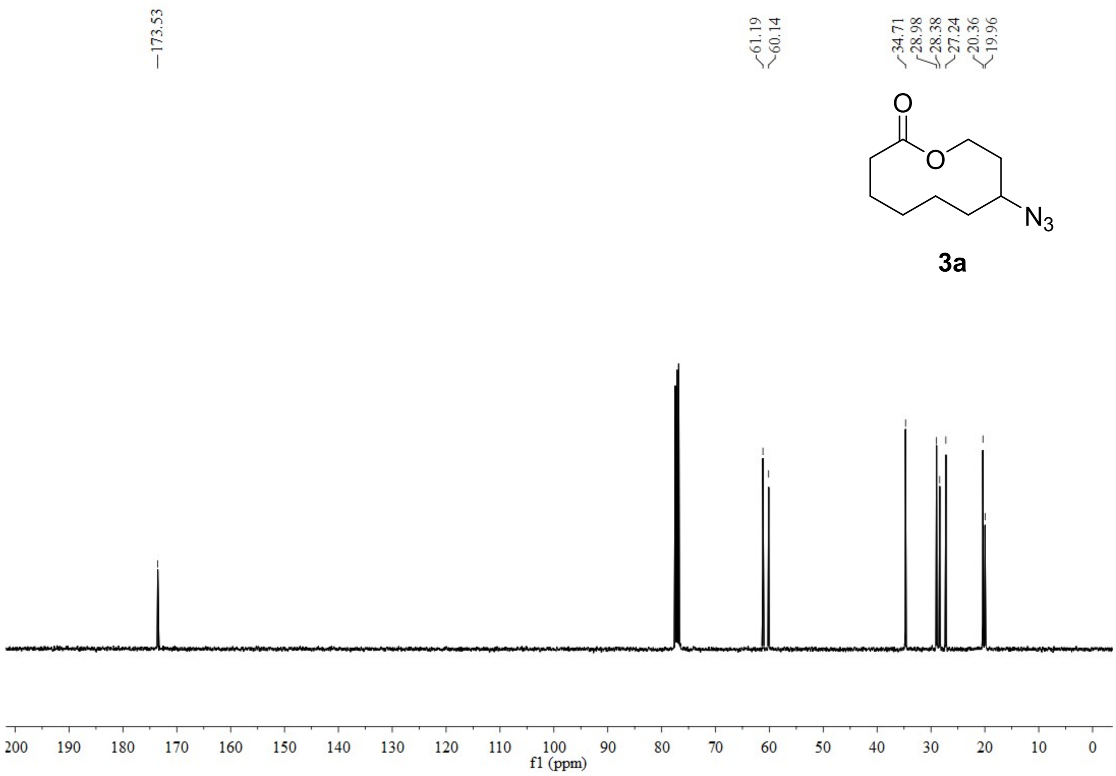

^{1}H NMR (400 MHz, $CDCl_3$) and ^{13}C NMR (100 MHz, $CDCl_3$) spectra of product **3b**

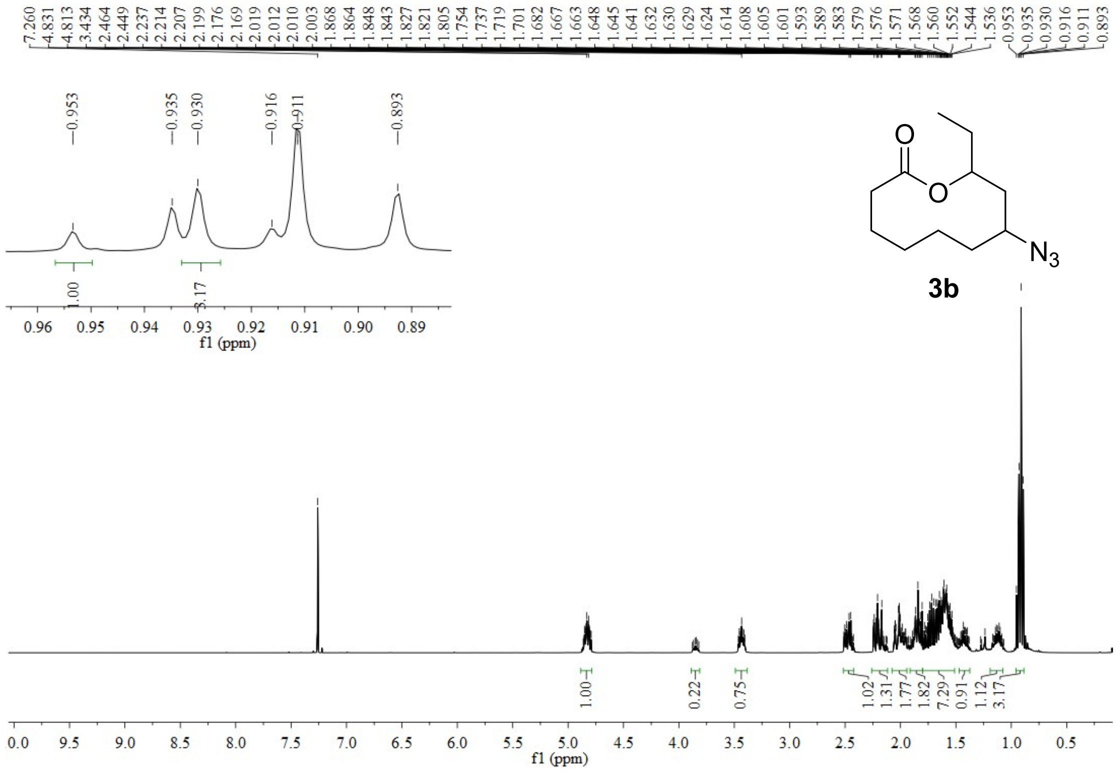

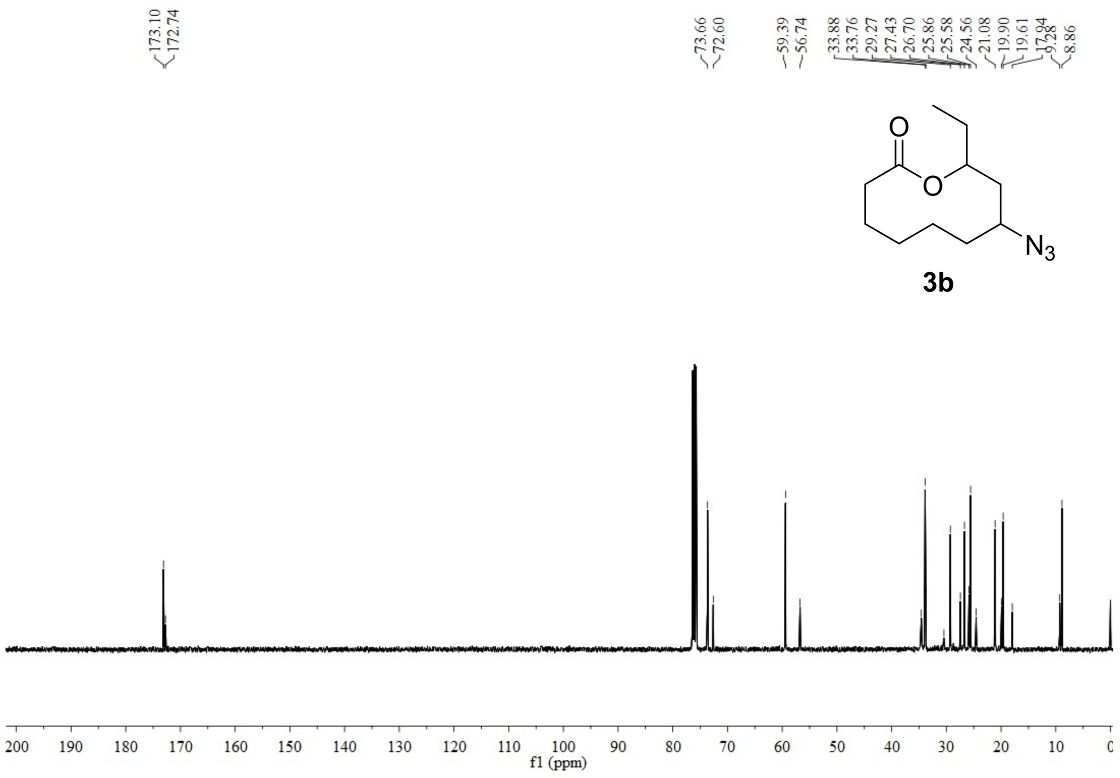

^{1}H NMR (400 MHz, $CDCl_3$) and ^{13}C NMR (100 MHz, $CDCl_3$) spectra of product **3c**

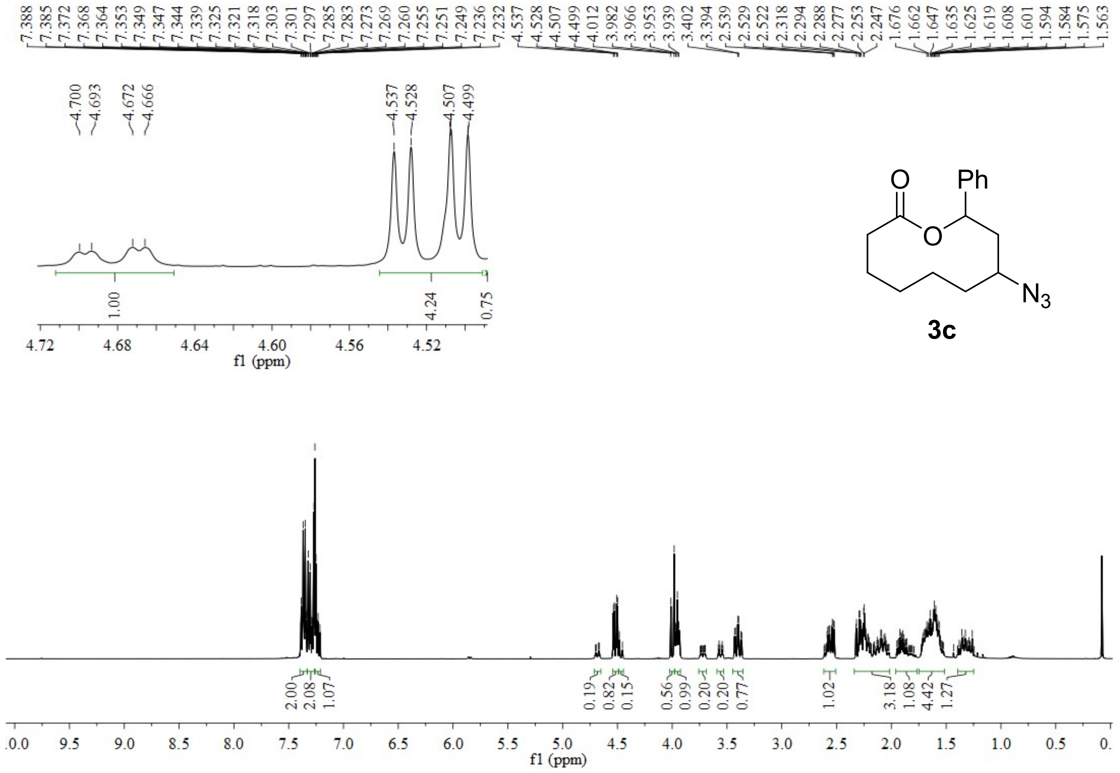

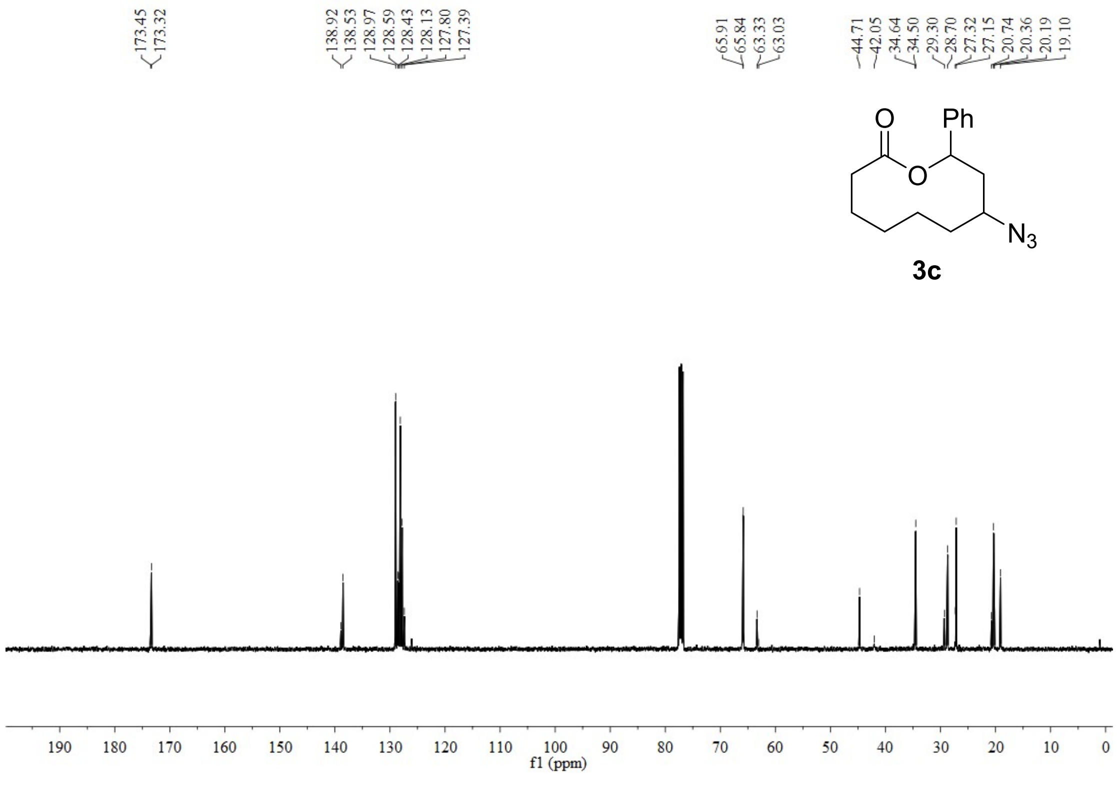

^{1}H NMR (400 MHz, $CDCl_3$) and ^{13}C NMR (100 MHz, $CDCl_3$) spectra of product **3d**

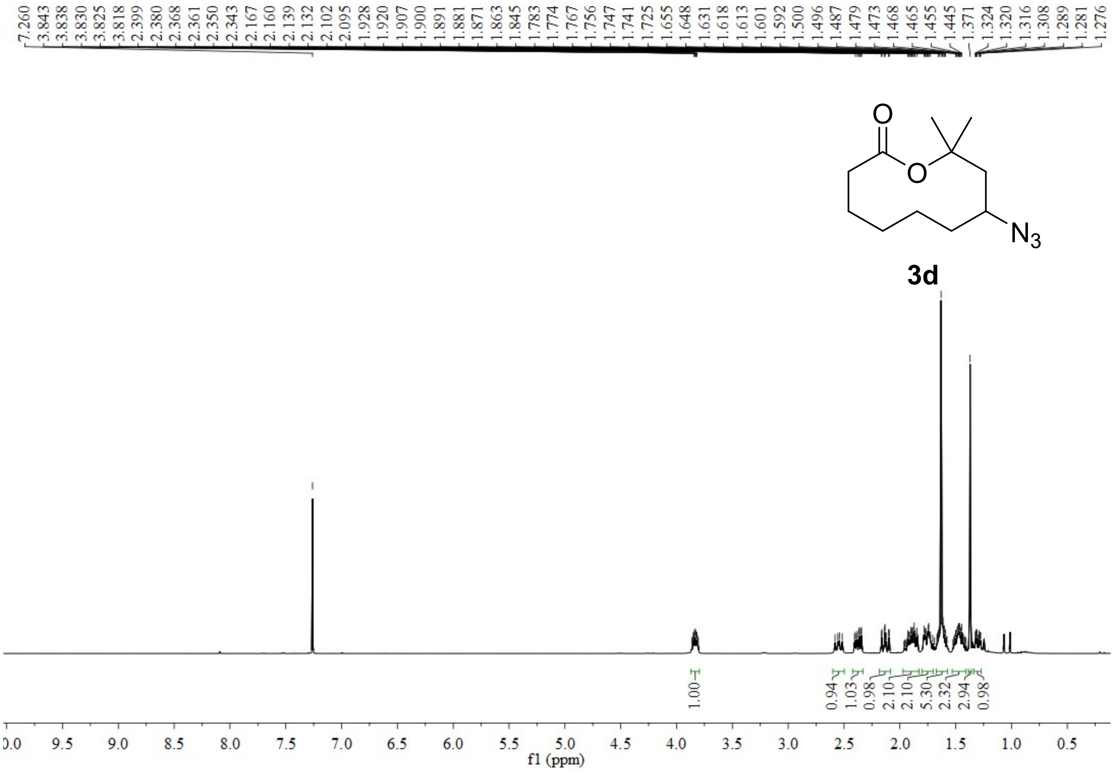

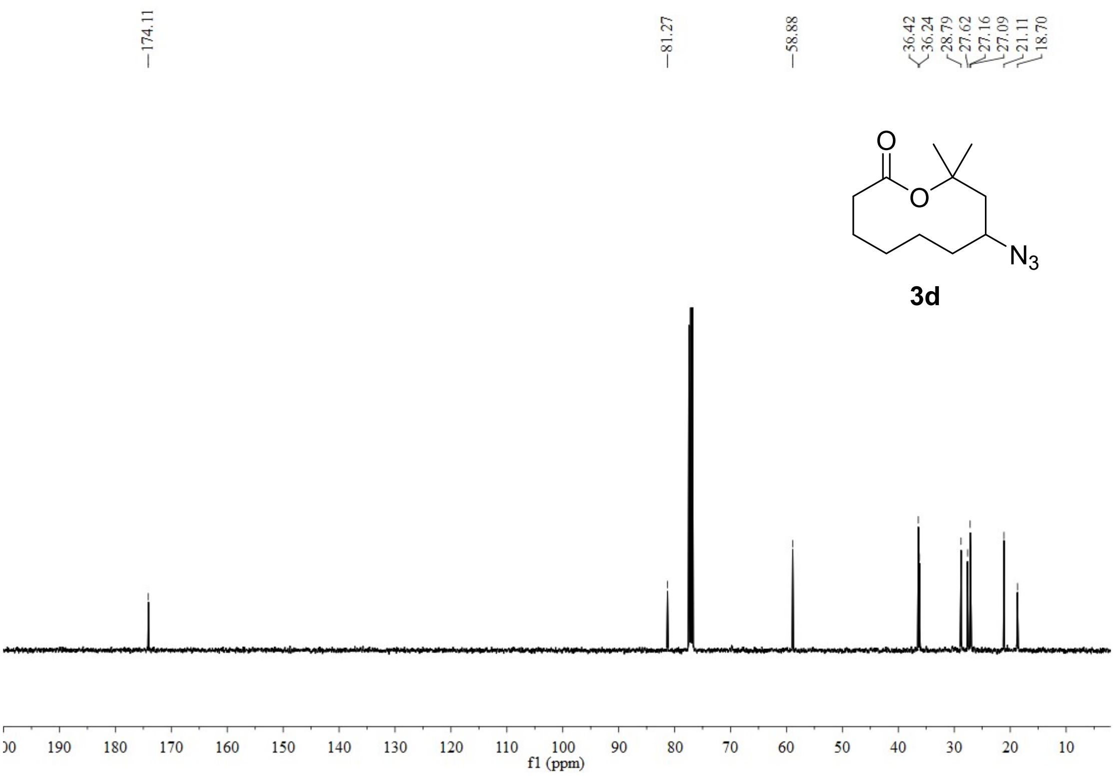

^{1}H NMR (400 MHz, $CDCl_3$) and ^{13}C NMR (100 MHz, $CDCl_3$) spectra of product **3e**

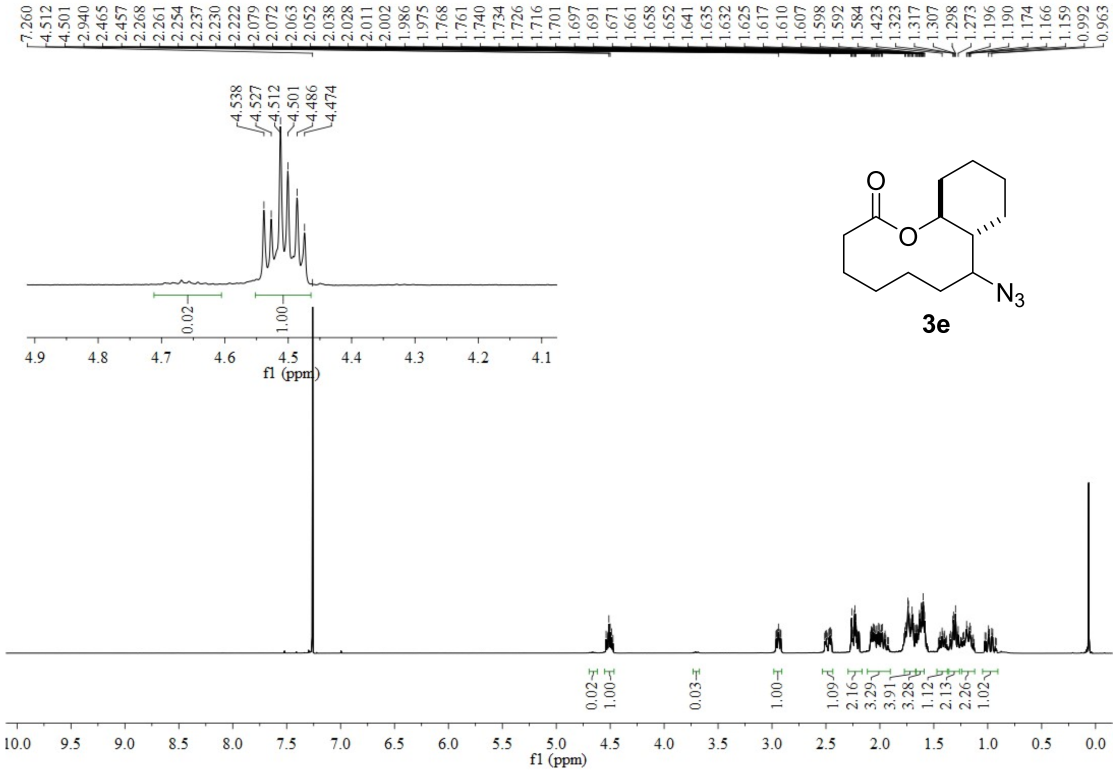

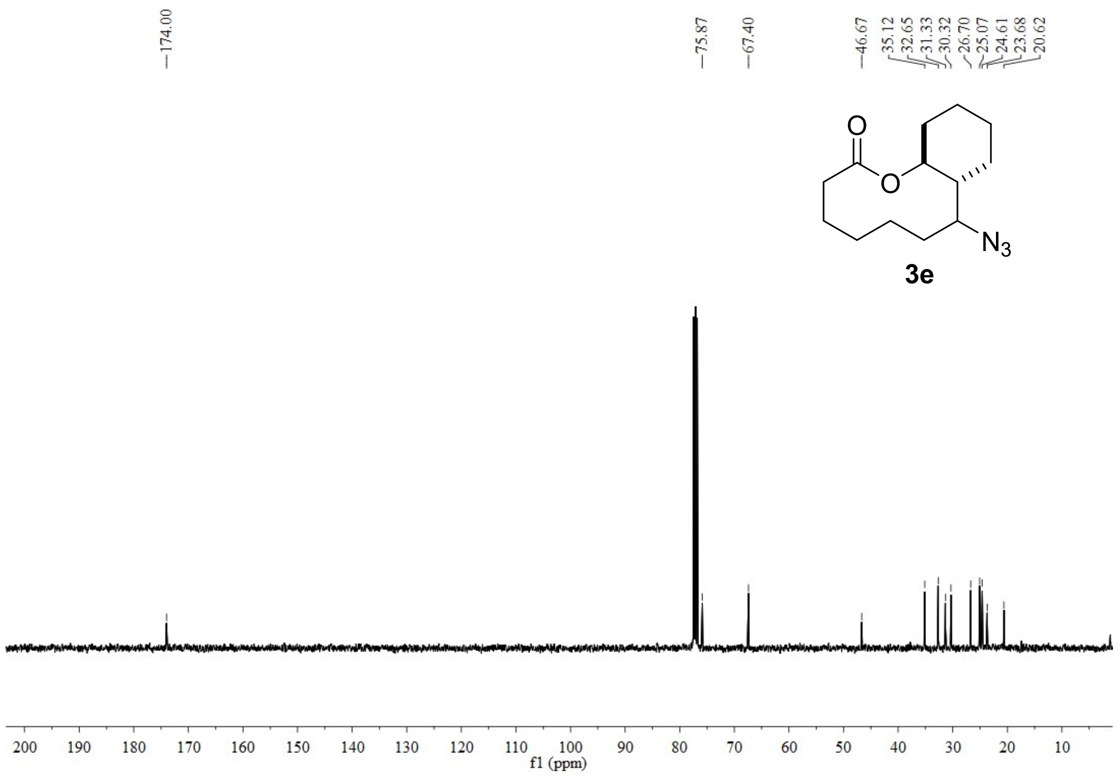

^{1}H NMR (400 MHz, $CDCl_3$) and ^{13}C NMR (100 MHz, $CDCl_3$) spectra of product **3f**

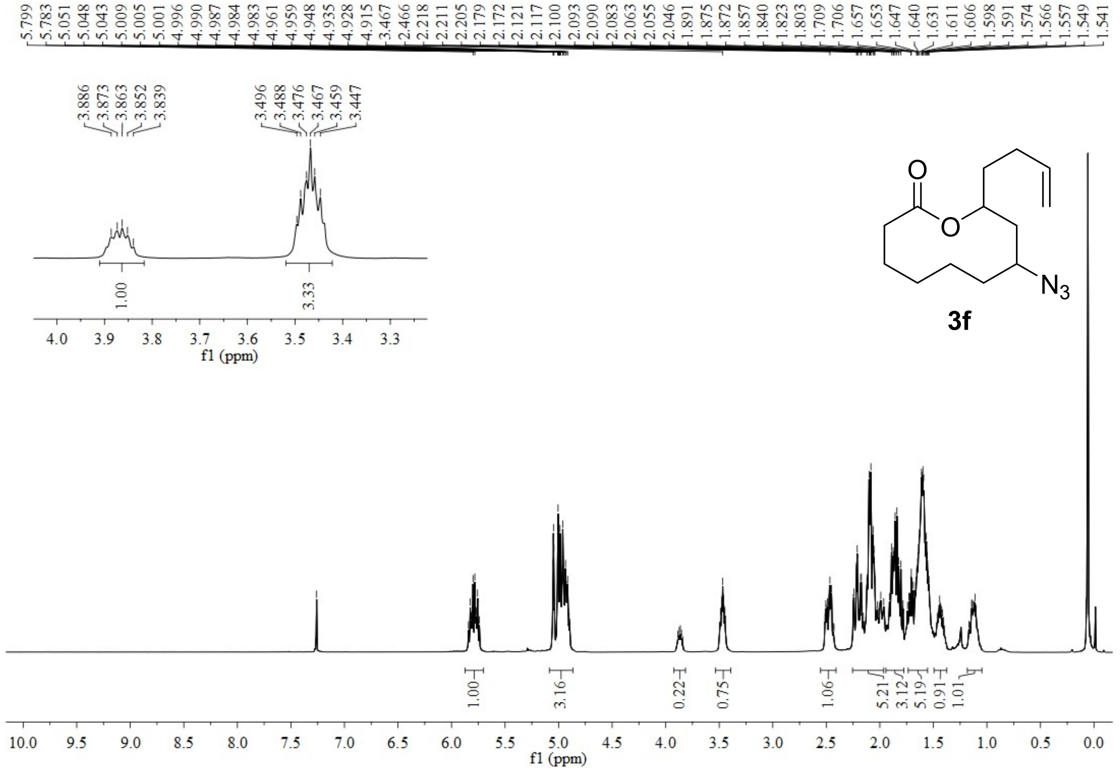

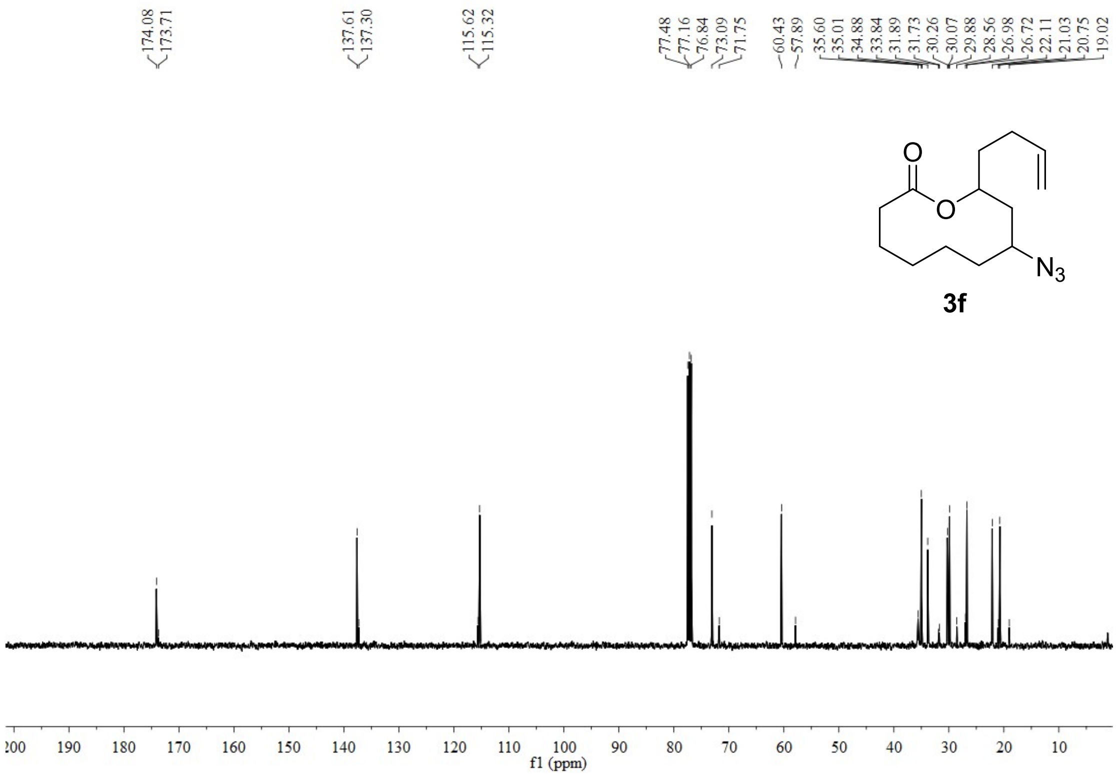

^{1}H NMR (400 MHz, $CDCl_3$) and ^{13}C NMR (100 MHz, $CDCl_3$) spectra of product **3g**

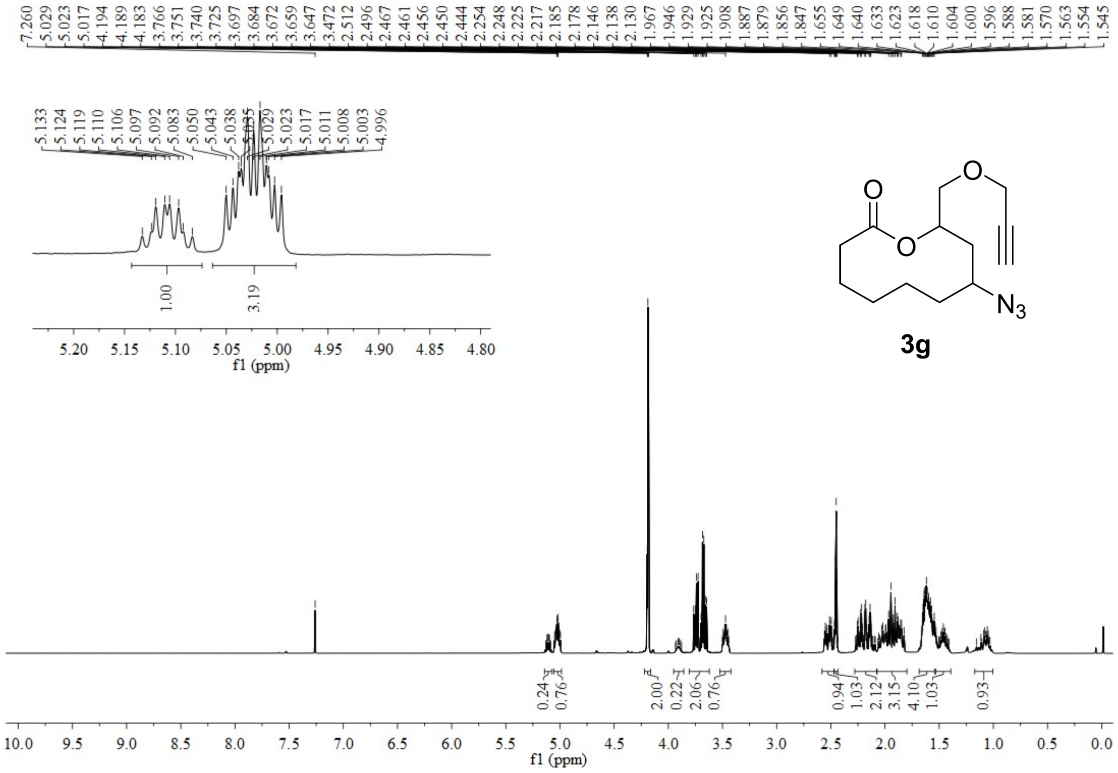

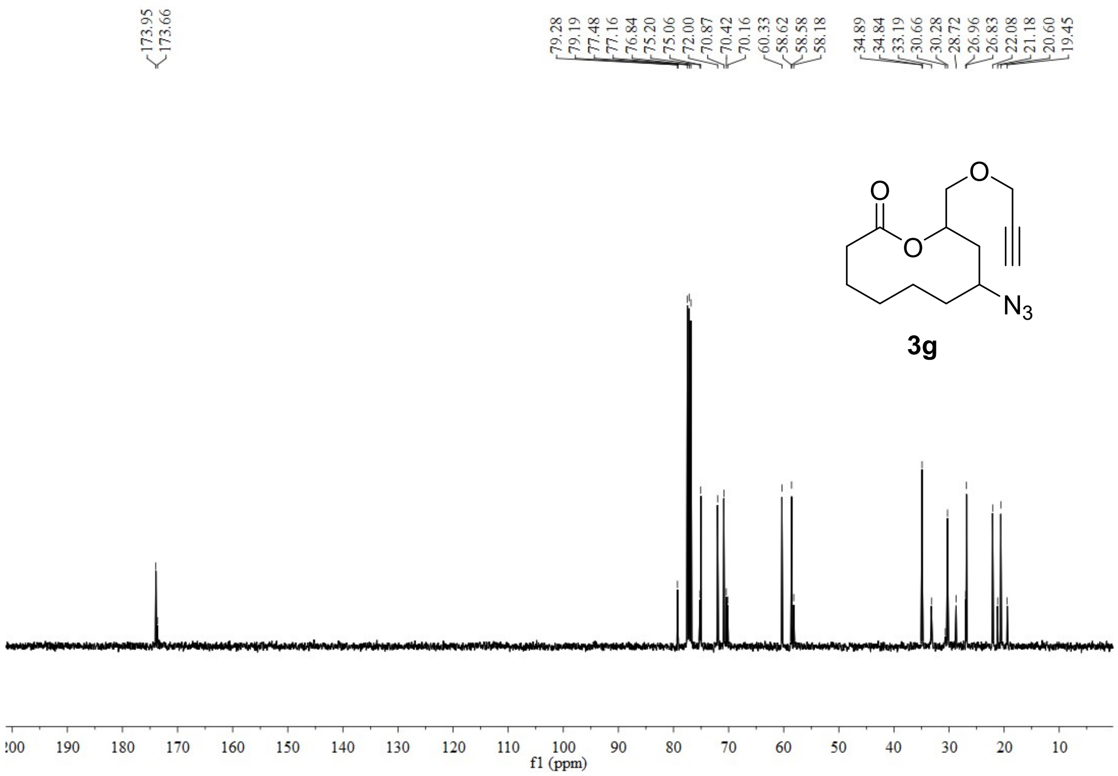

^{1}H NMR (400 MHz, $CDCl_3$) and ^{13}C NMR (100 MHz, $CDCl_3$) spectra of product **3h**

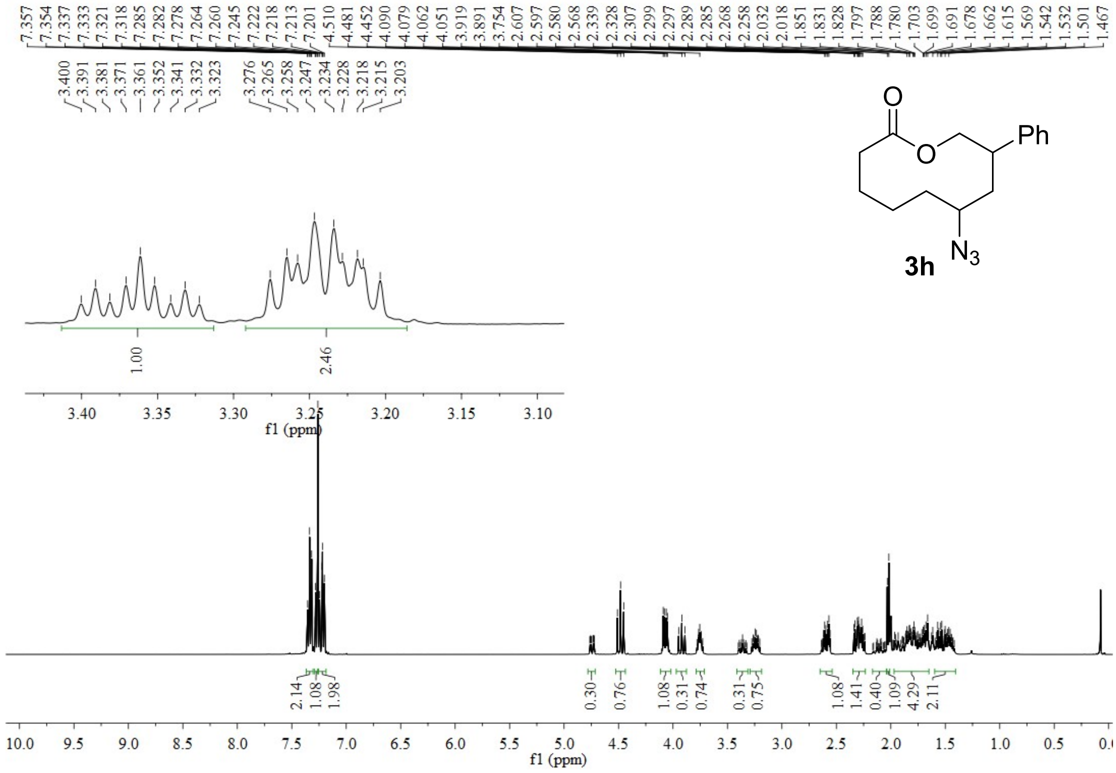

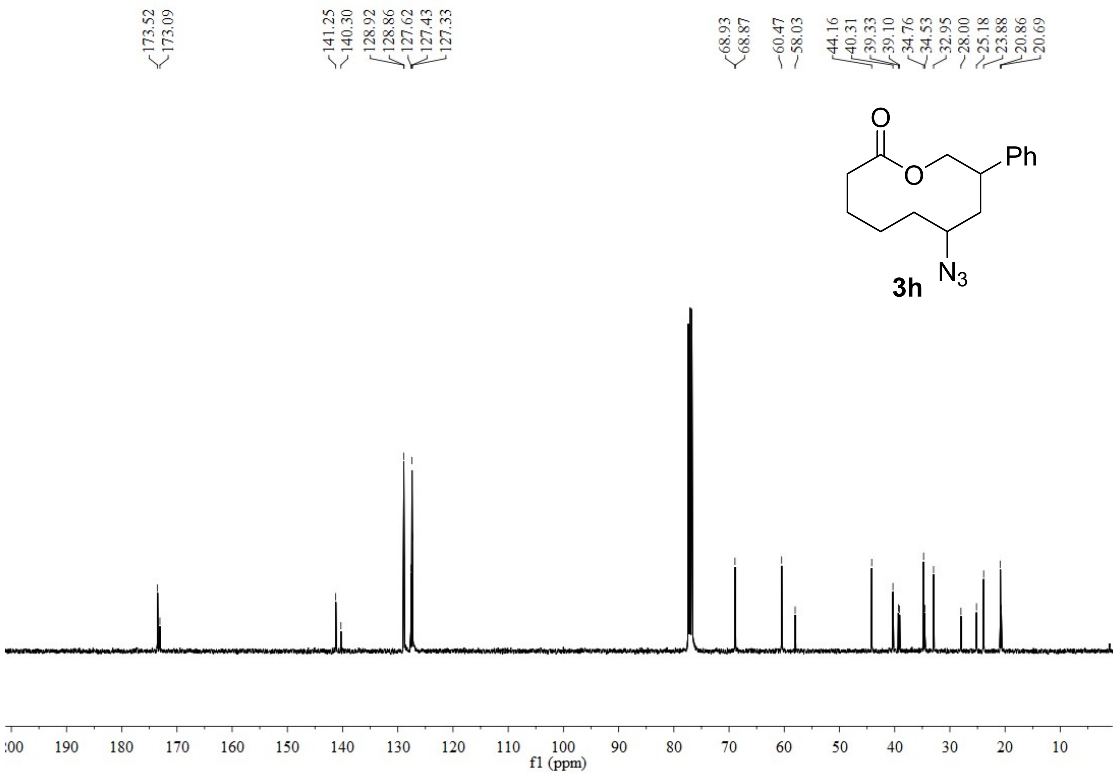

^{1}H NMR (400 MHz, $CDCl_3$) and ^{13}C NMR (100 MHz, $CDCl_3$) spectra of product **3i**

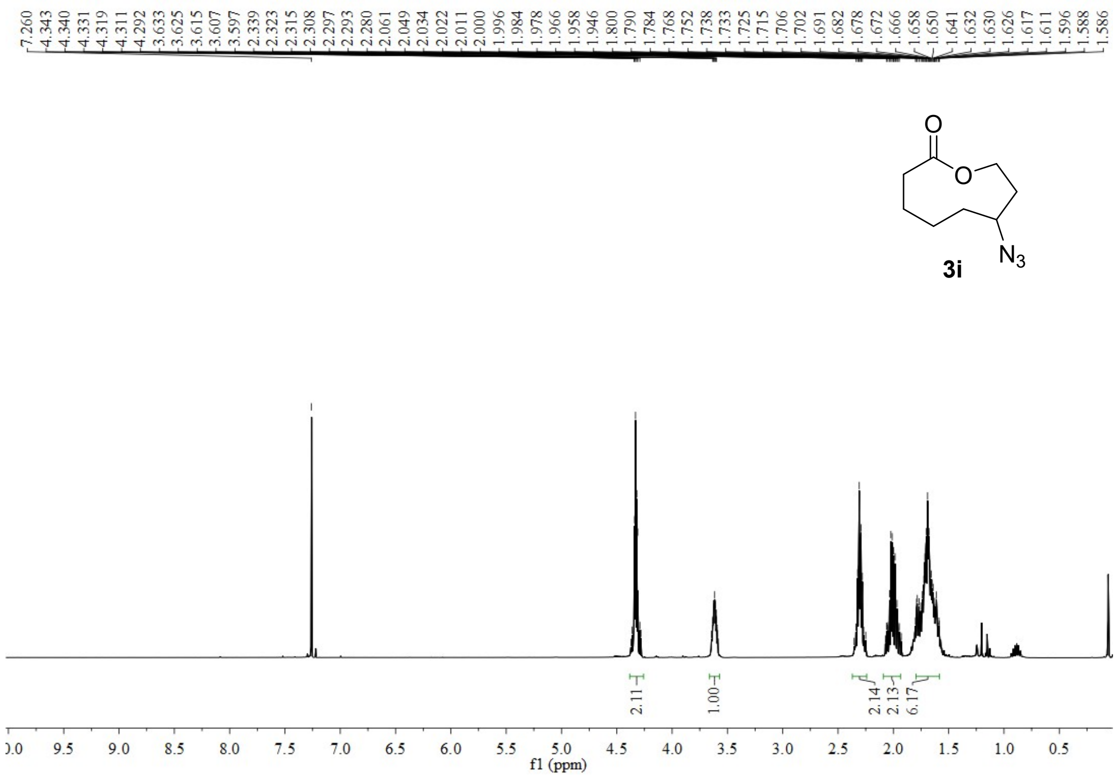

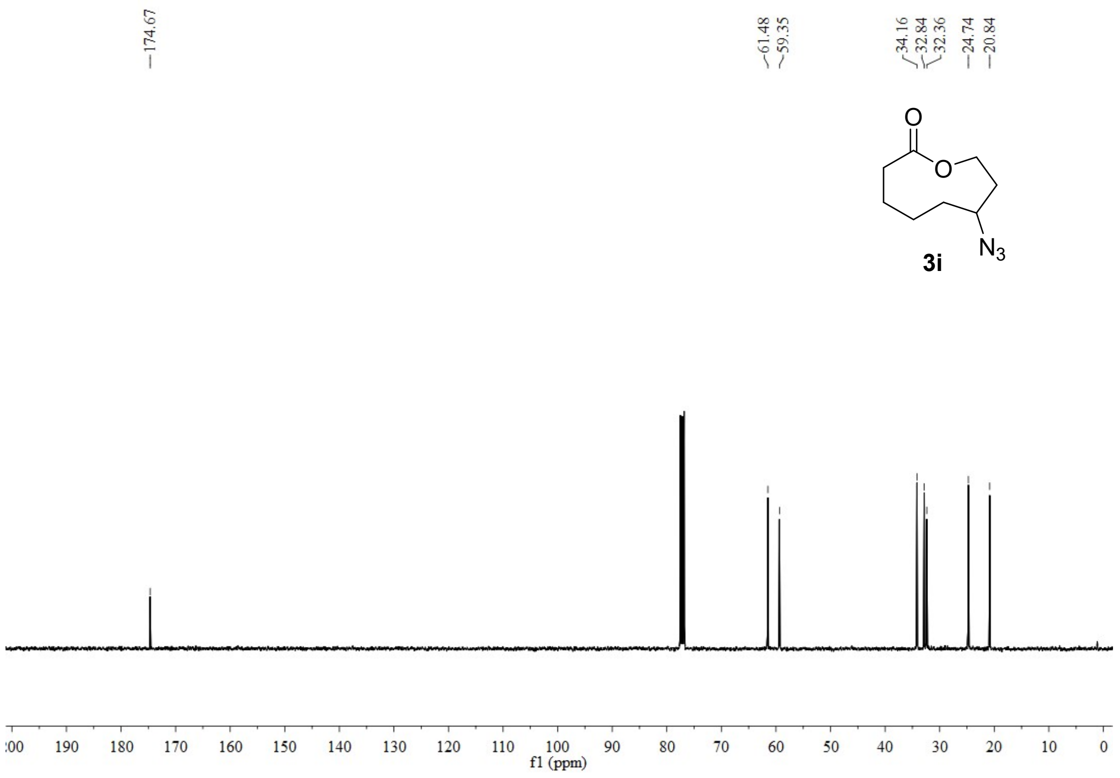

^{1}H NMR (400 MHz, $CDCl_3$) and ^{13}C NMR (100 MHz, $CDCl_3$) spectra of product **3j**

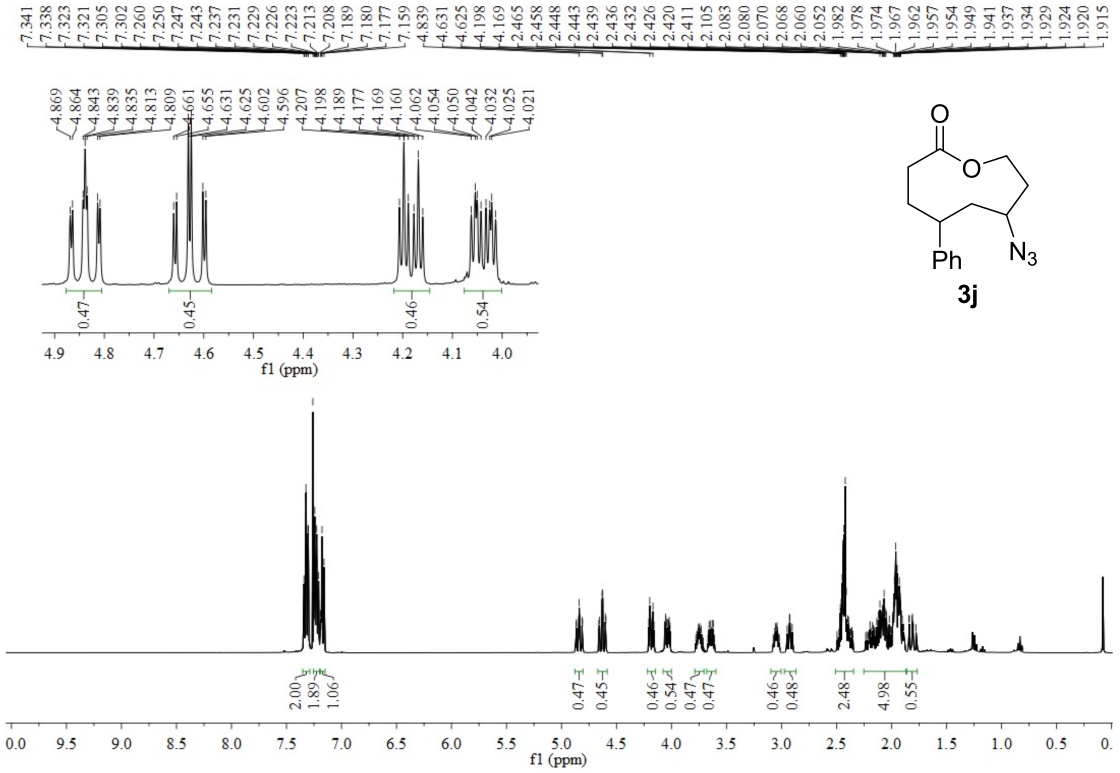

3j

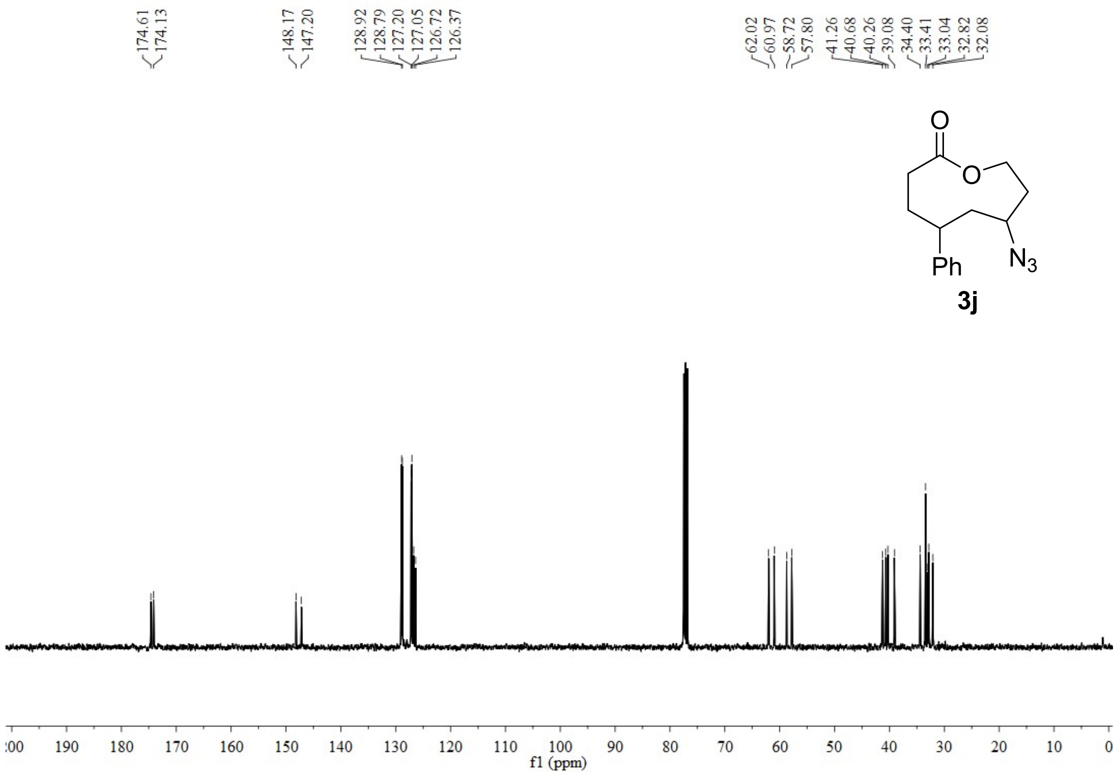

3j

^{1}H NMR (400 MHz, $CDCl_3$) and ^{13}C NMR (100 MHz, $CDCl_3$) spectra of product **3k**

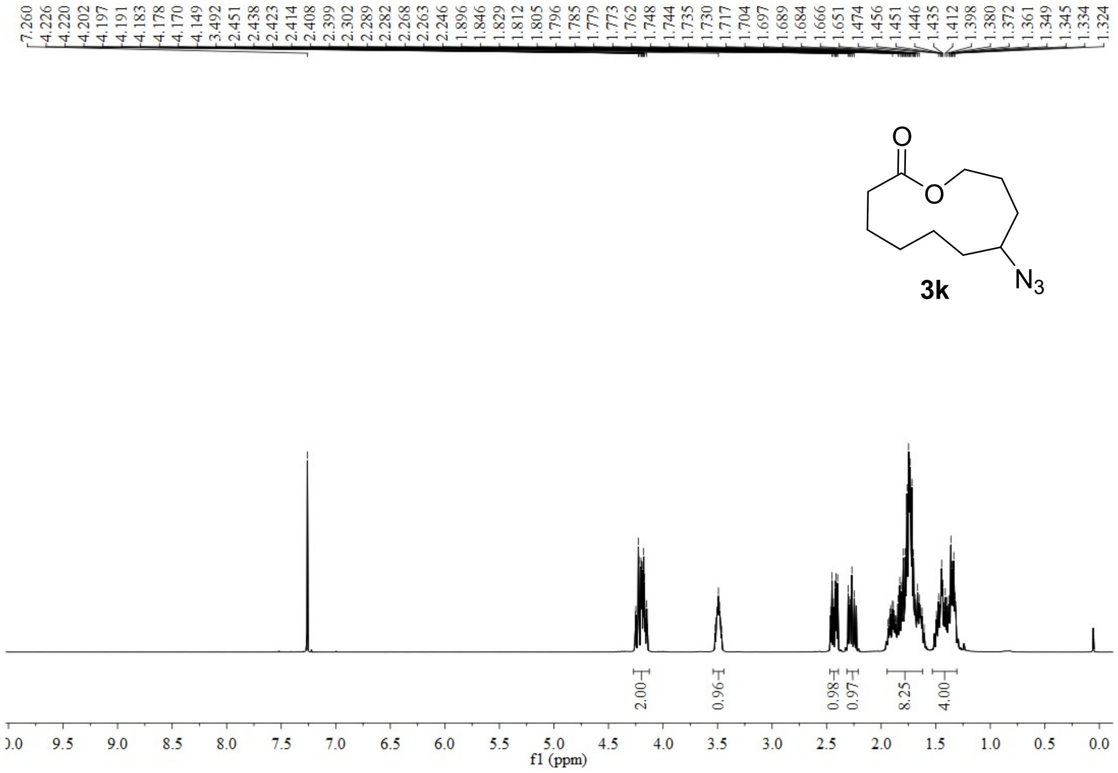

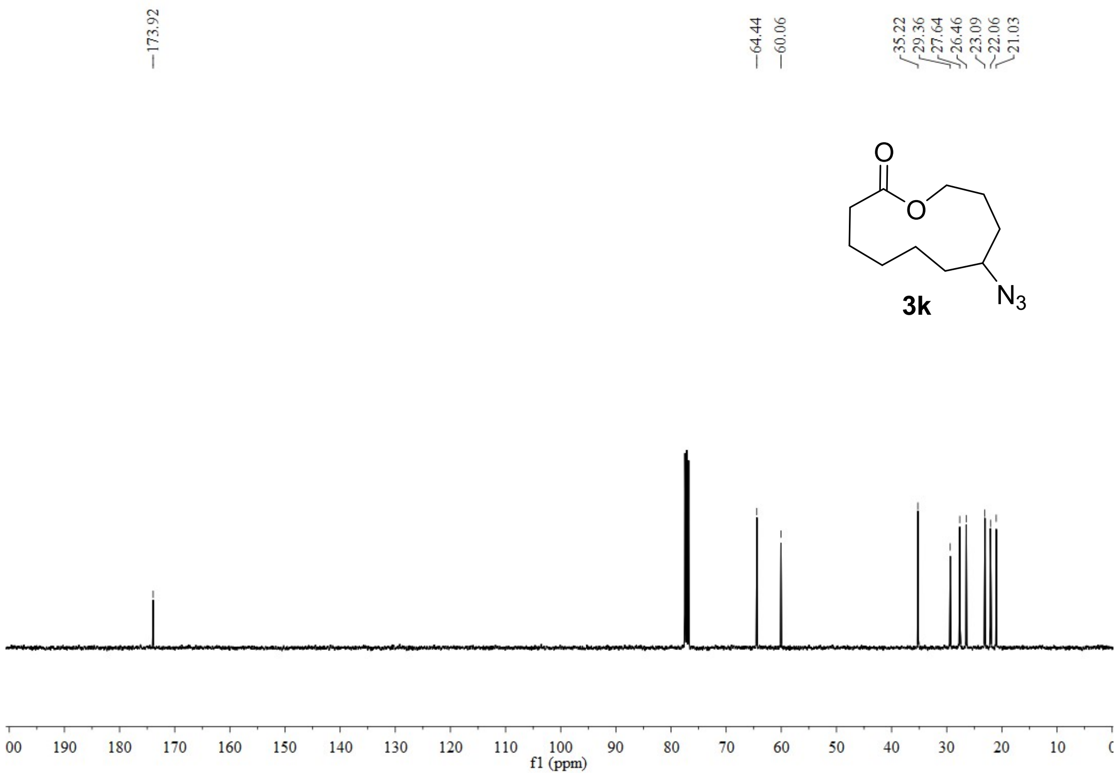

^{1}H NMR (400 MHz, $CDCl_3$) and ^{13}C NMR (100 MHz, $CDCl_3$) spectra of product **3l**

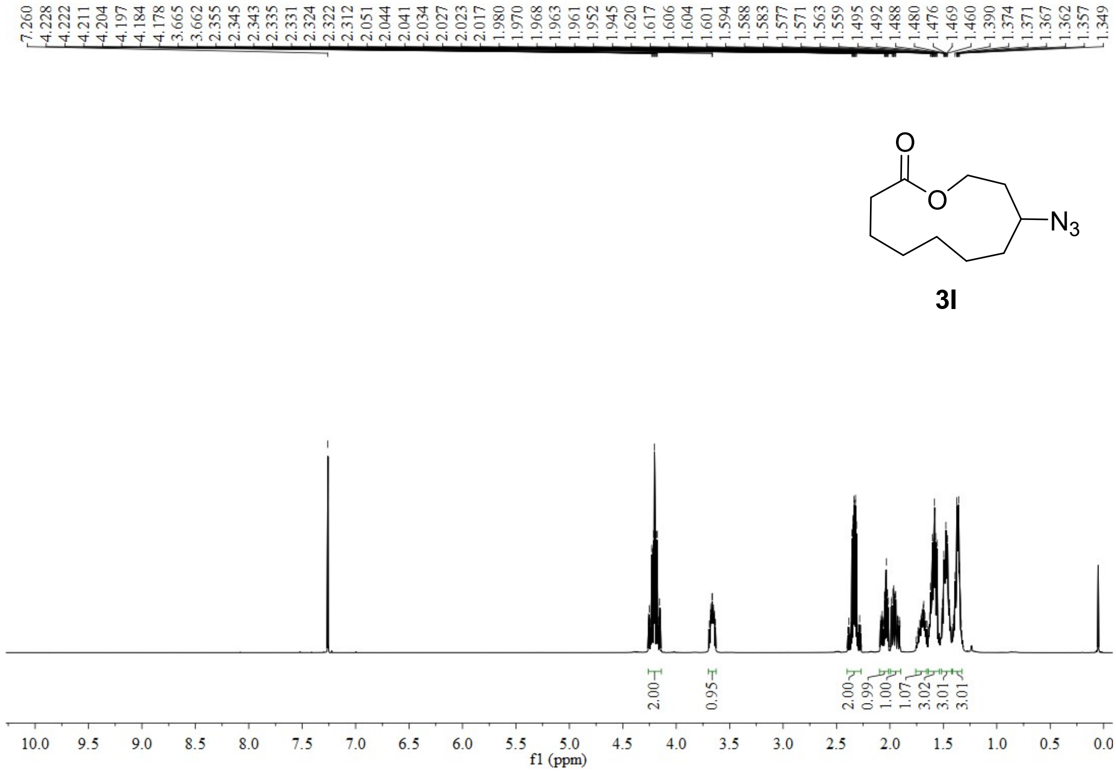

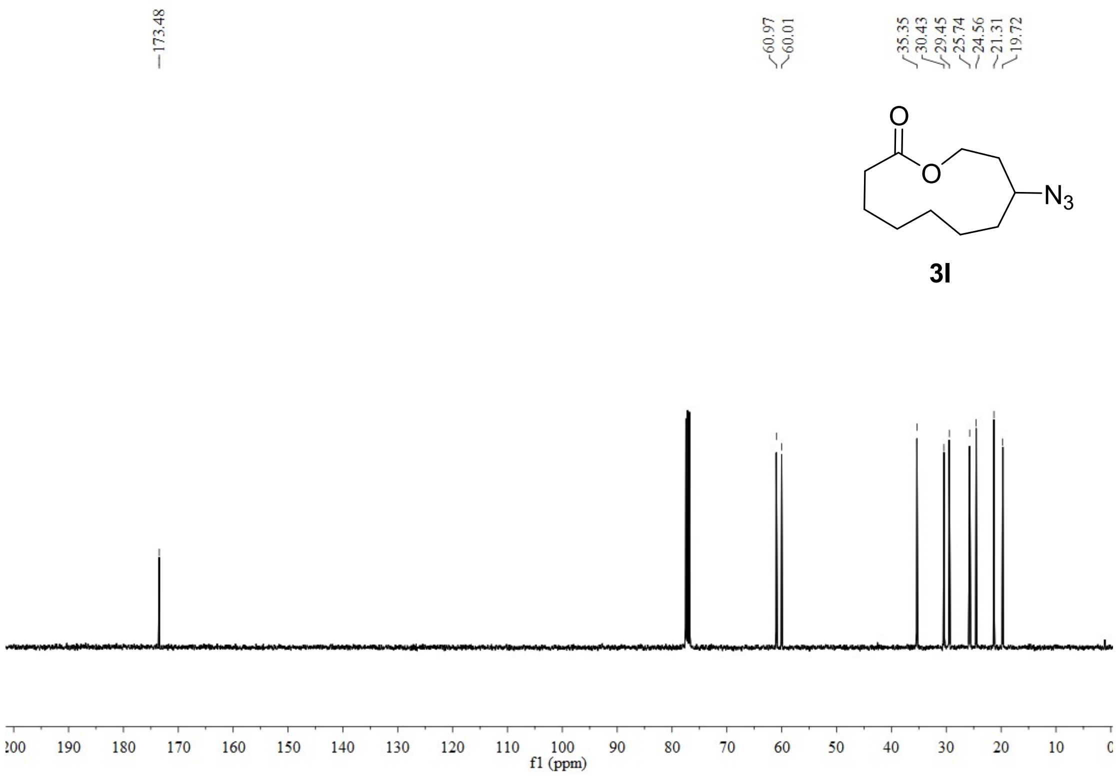

^{1}H NMR (400 MHz, $CDCl_3$) and ^{13}C NMR (100 MHz, $CDCl_3$) spectra of product **3m**

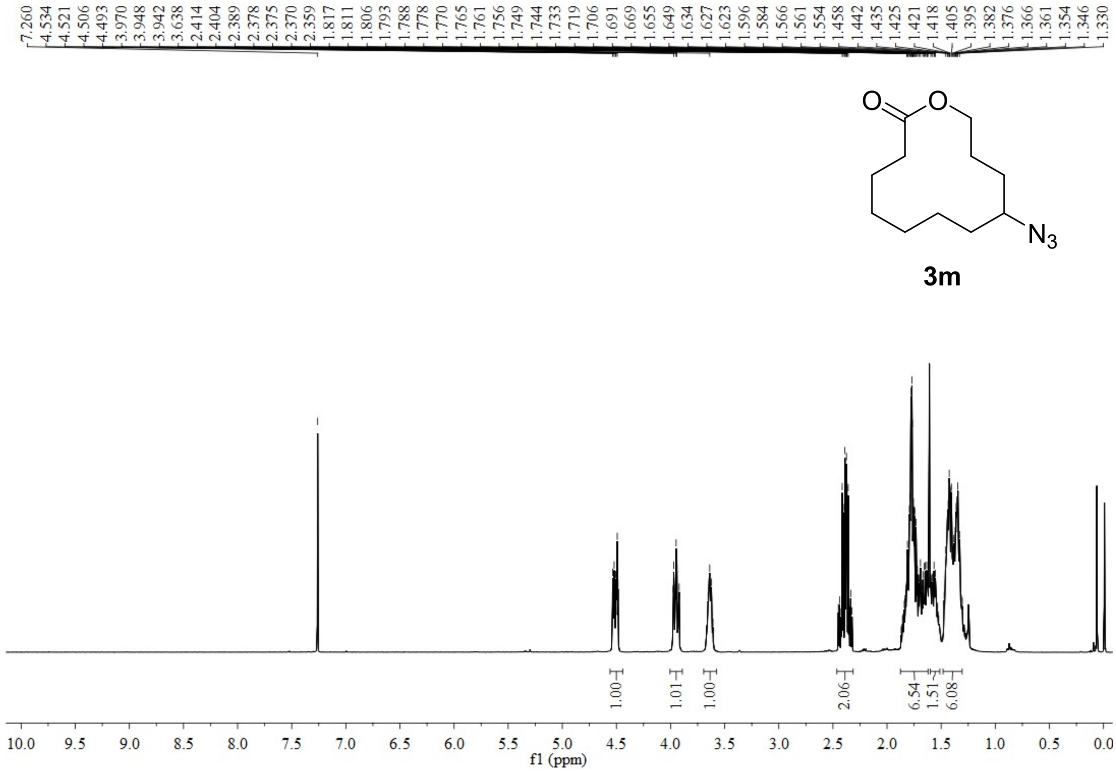

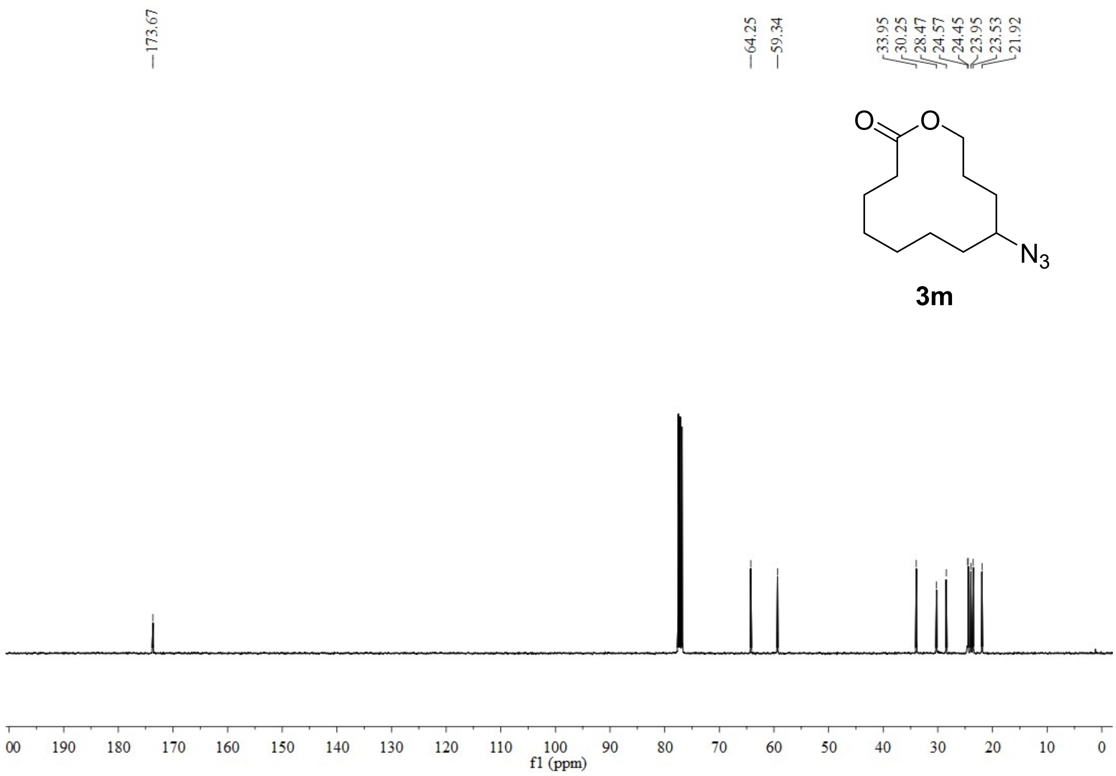

^{1}H NMR (400 MHz, $CDCl_3$) and ^{13}C NMR (100 MHz, $CDCl_3$) spectra of product **3n**

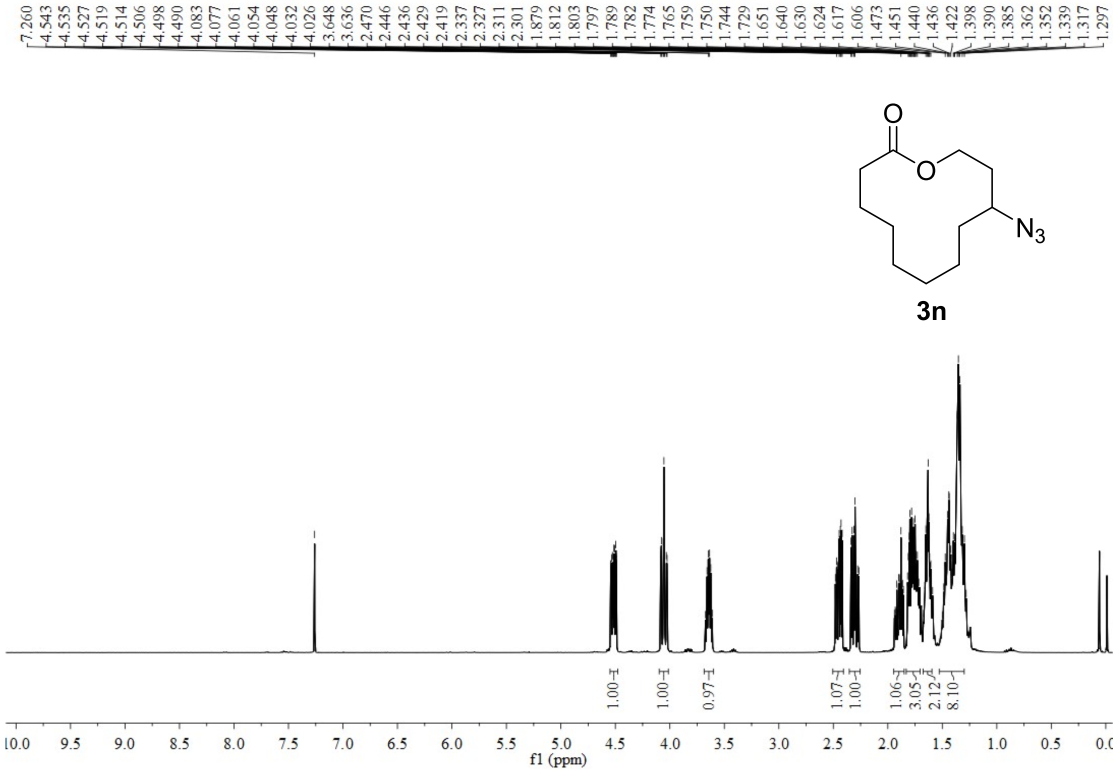

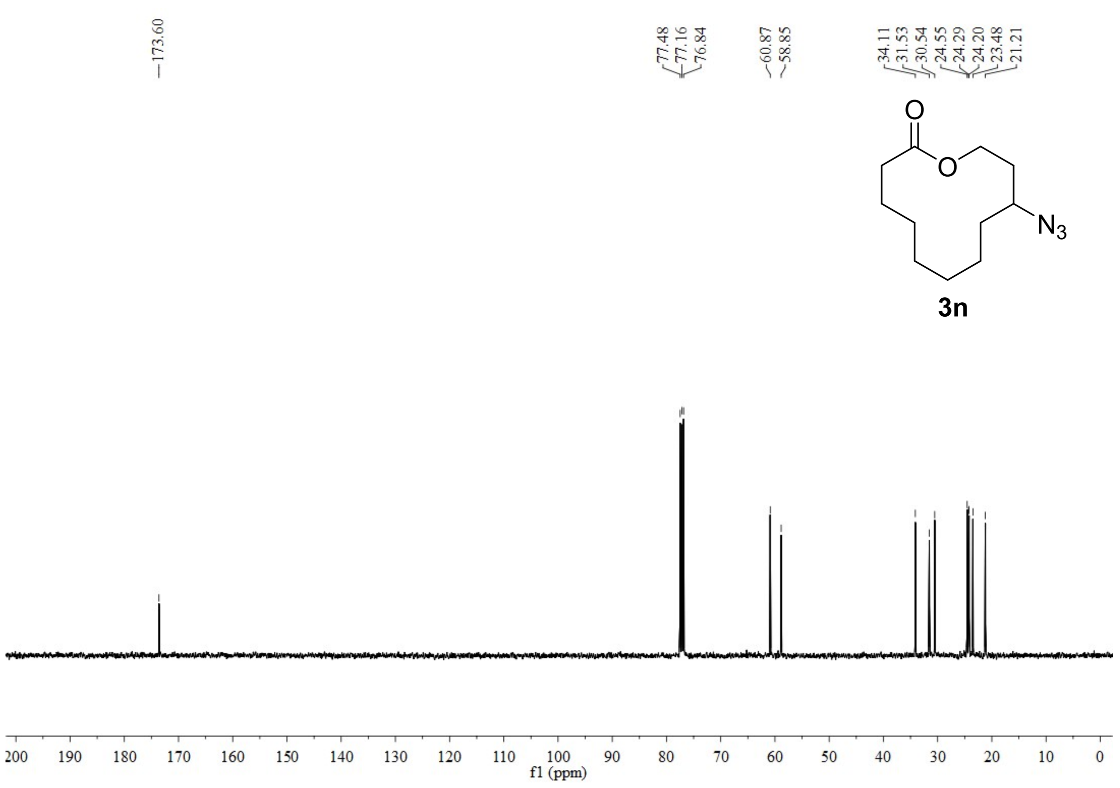

^{1}H NMR (400 MHz, $CDCl_3$) and ^{13}C NMR (100 MHz, $CDCl_3$) spectra of product **3o**

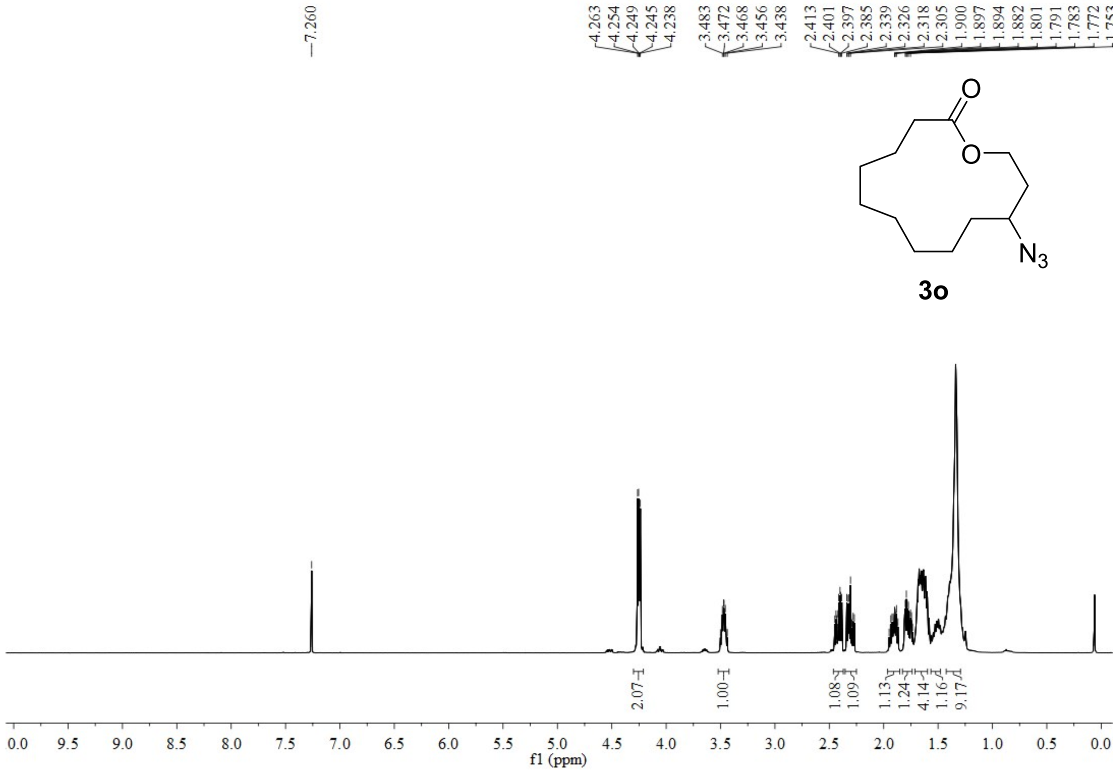

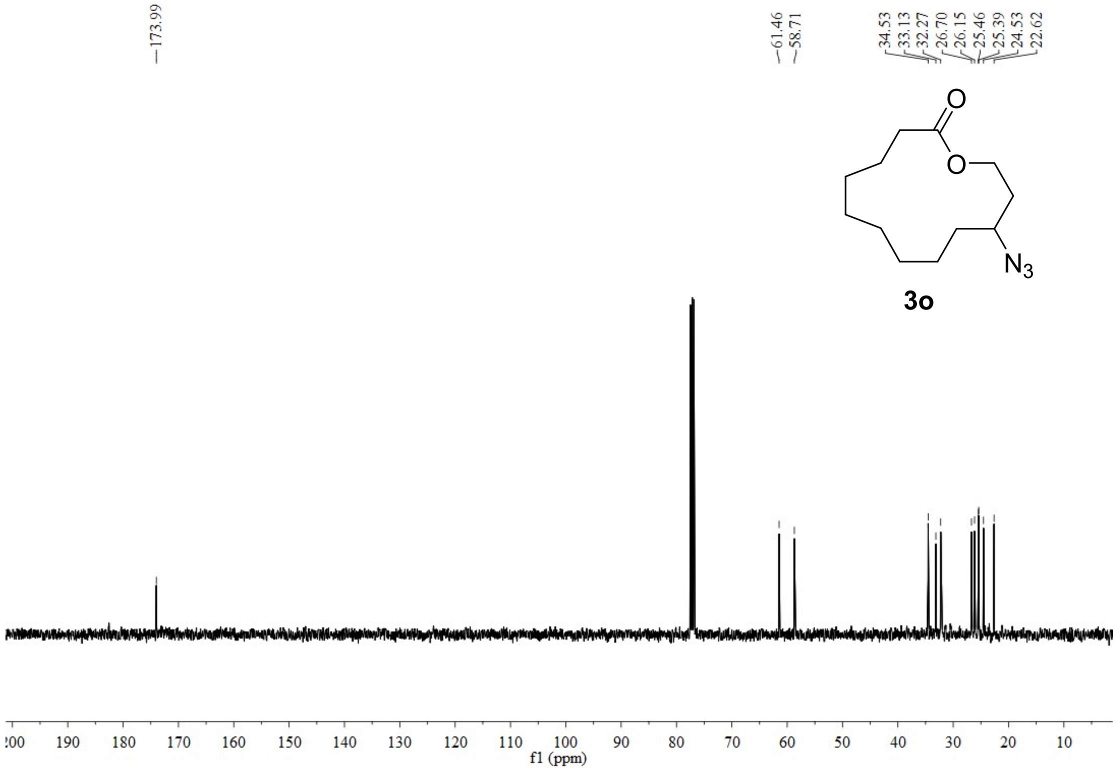

^{1}H NMR (400 MHz, $CDCl_3$) and ^{13}C NMR (100 MHz, $CDCl_3$) spectra of product **3p**

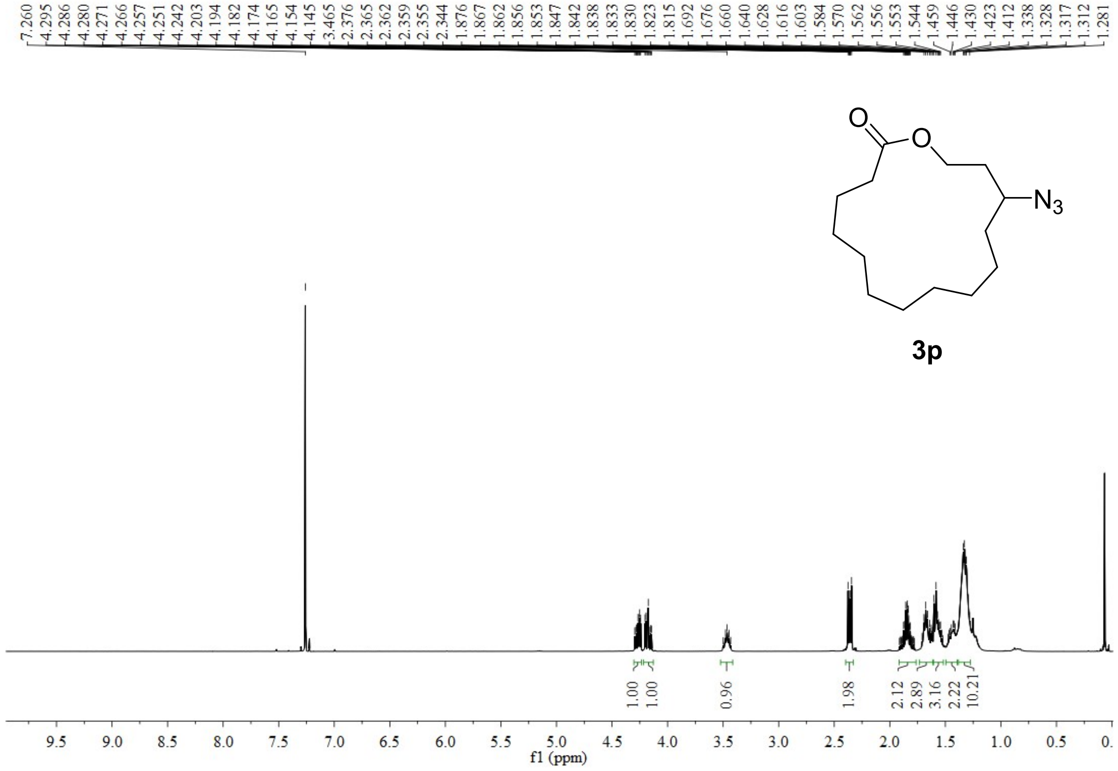

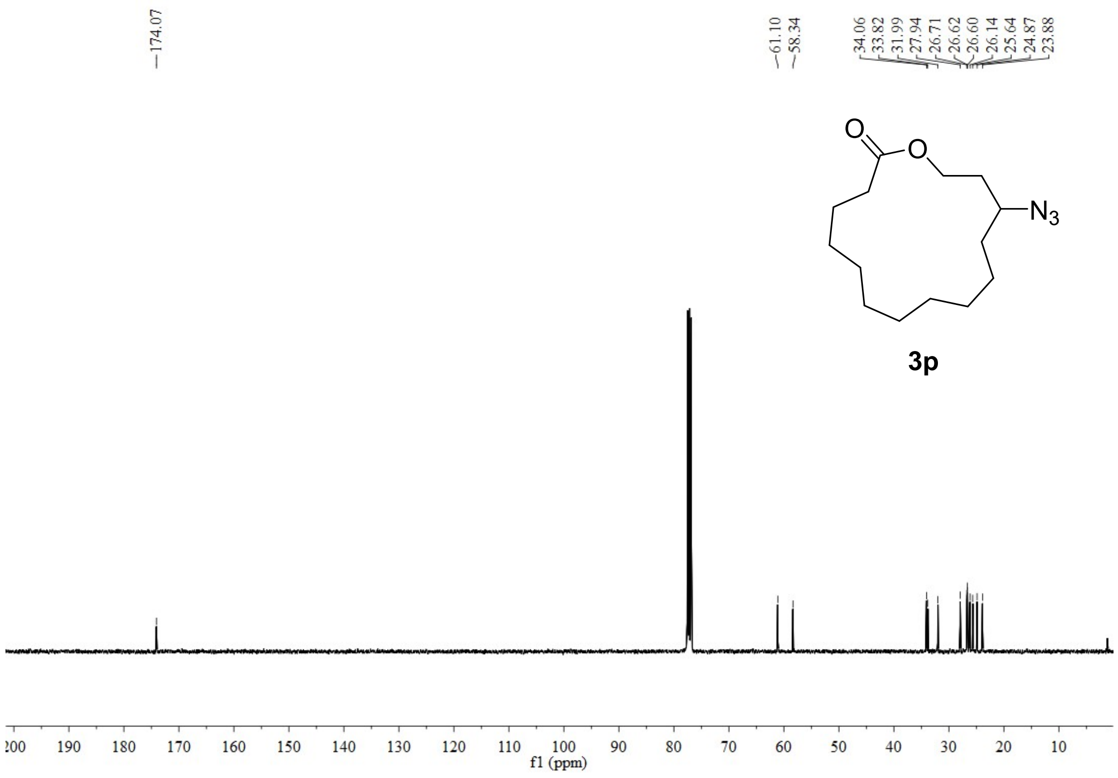

^{1}H NMR (400 MHz, $CDCl_3$) and ^{13}C NMR (100 MHz, $CDCl_3$) spectra of product **3q**

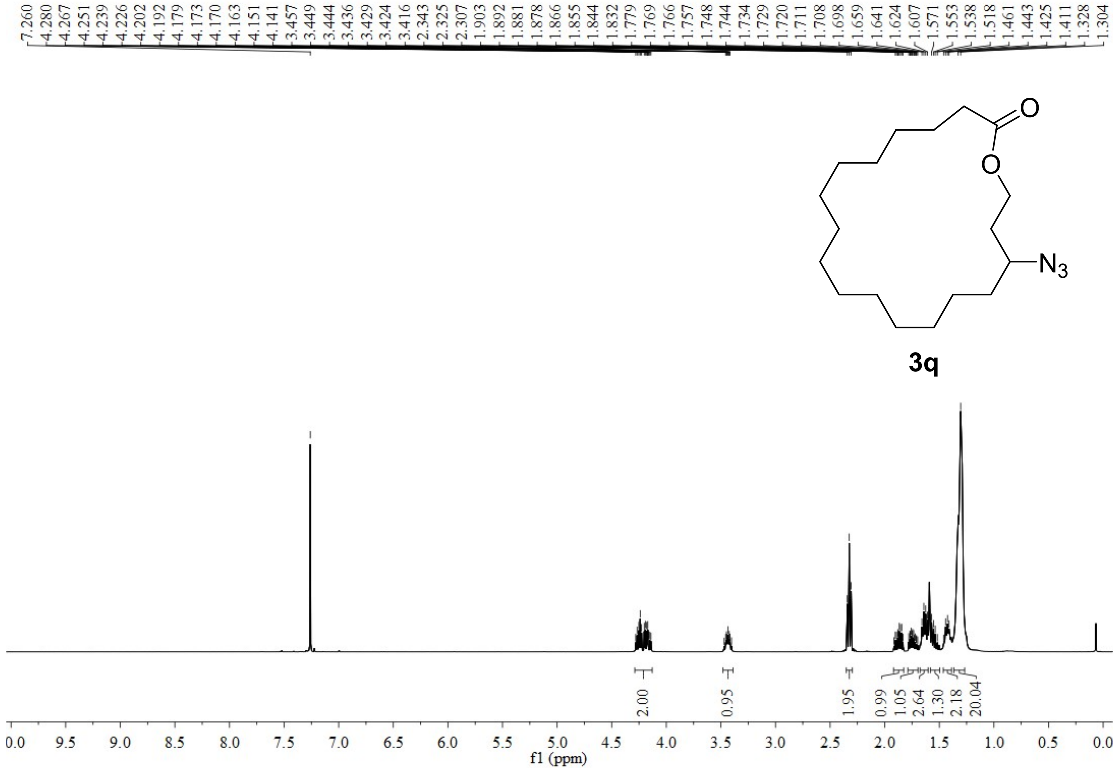

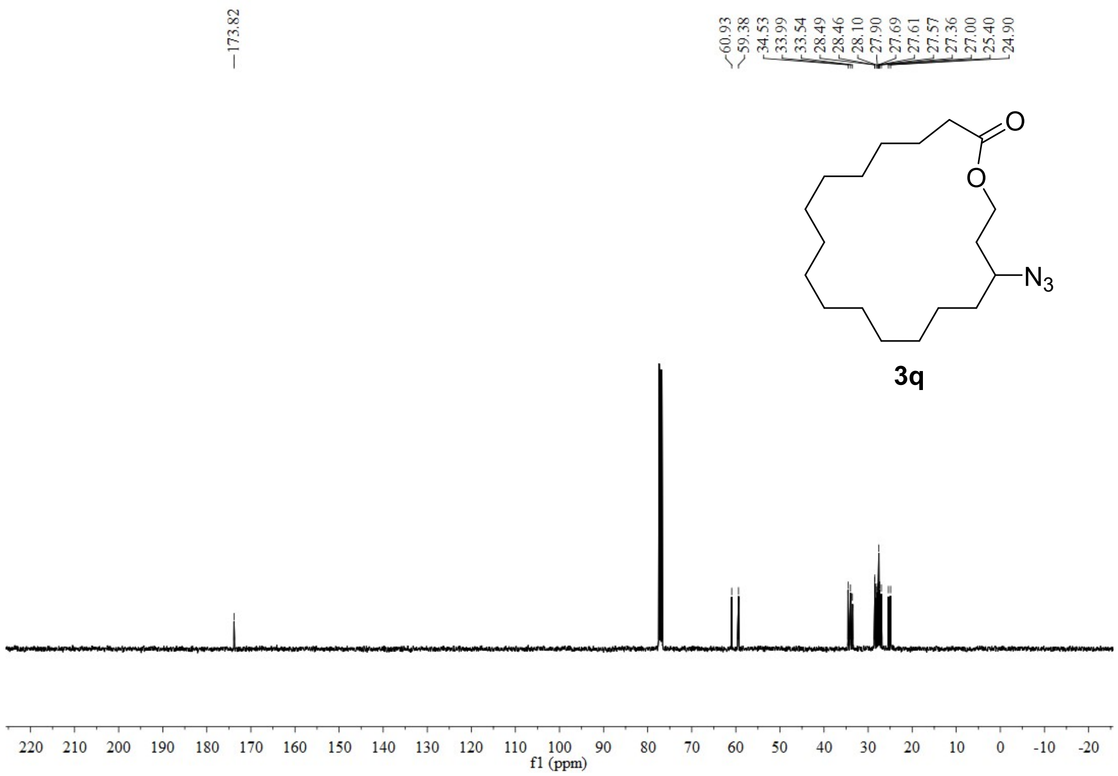

^1H NMR (400 MHz, $CDCl_3$) and ^{13}C NMR (100 MHz, $CDCl_3$) spectra of product **4a**

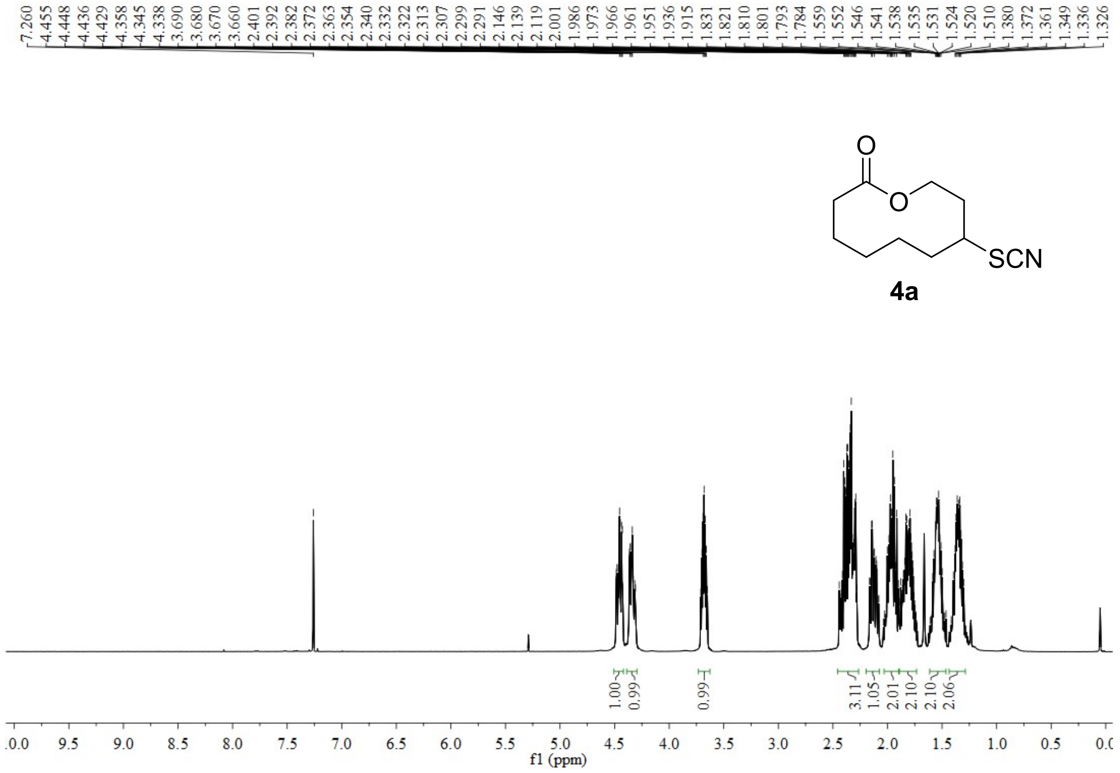

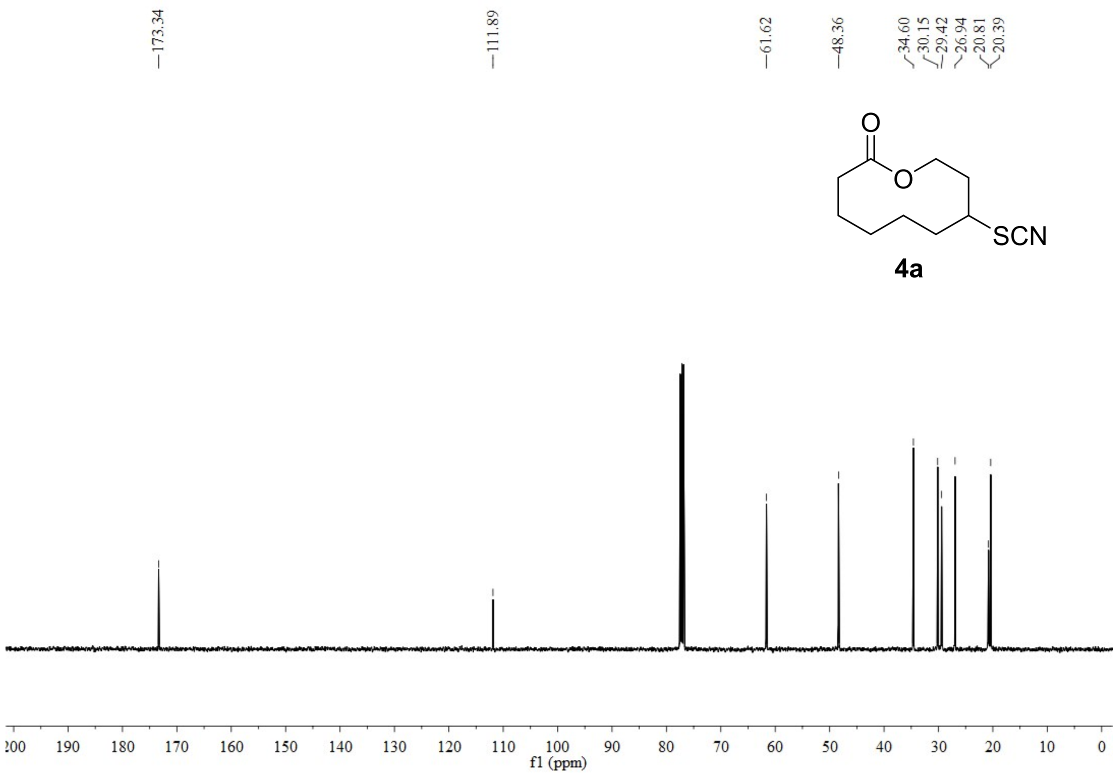

^{1}H NMR (400 MHz, $CDCl_3$) and ^{13}C NMR (100 MHz, $CDCl_3$) spectra of product **4b**

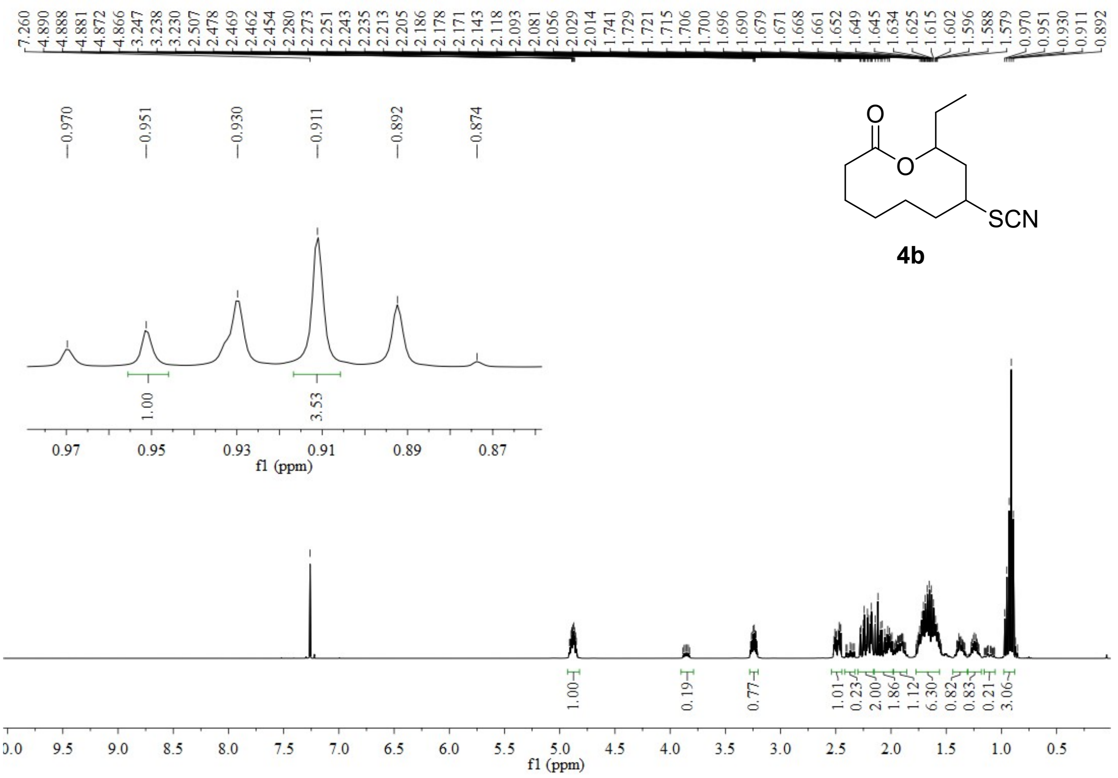

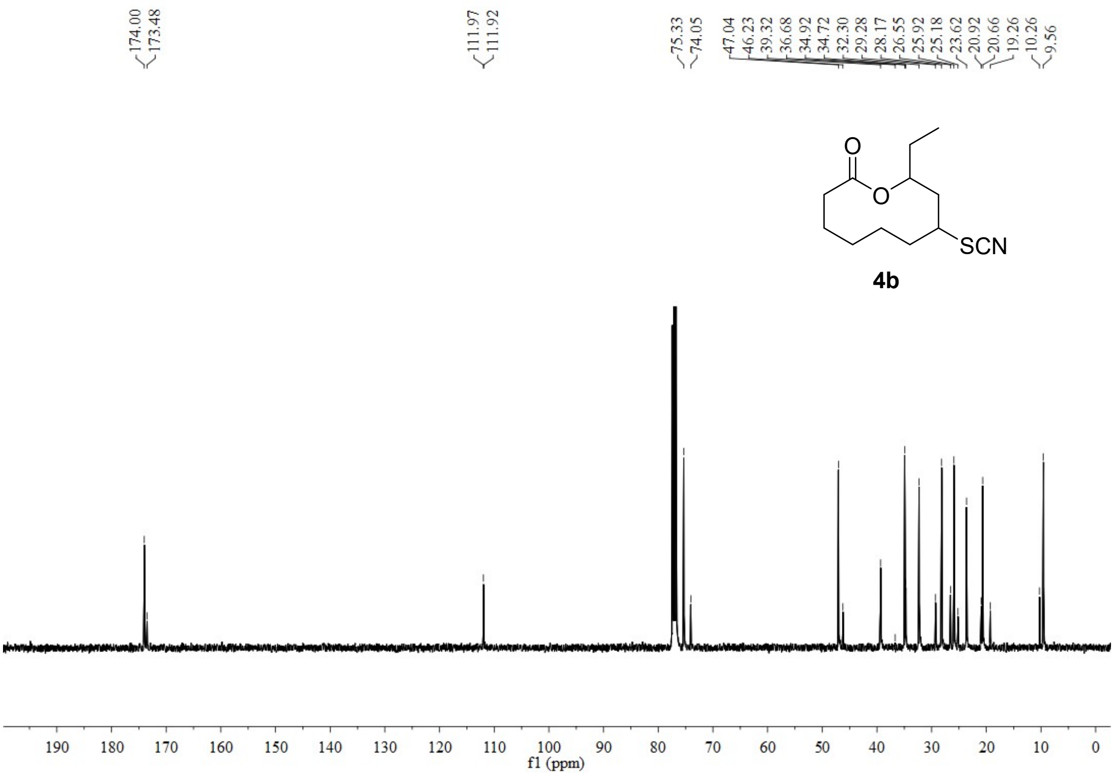

^{1}H NMR (400 MHz, $CDCl_3$) and ^{13}C NMR (100 MHz, $CDCl_3$) spectra of product **4c**

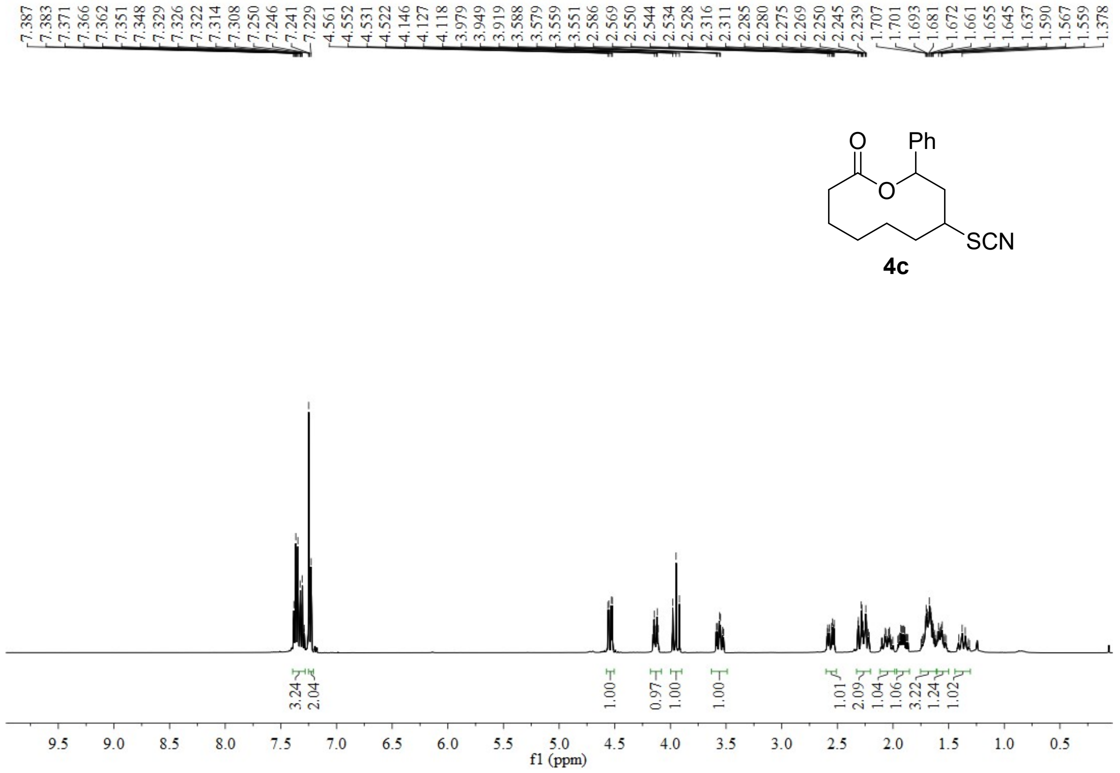

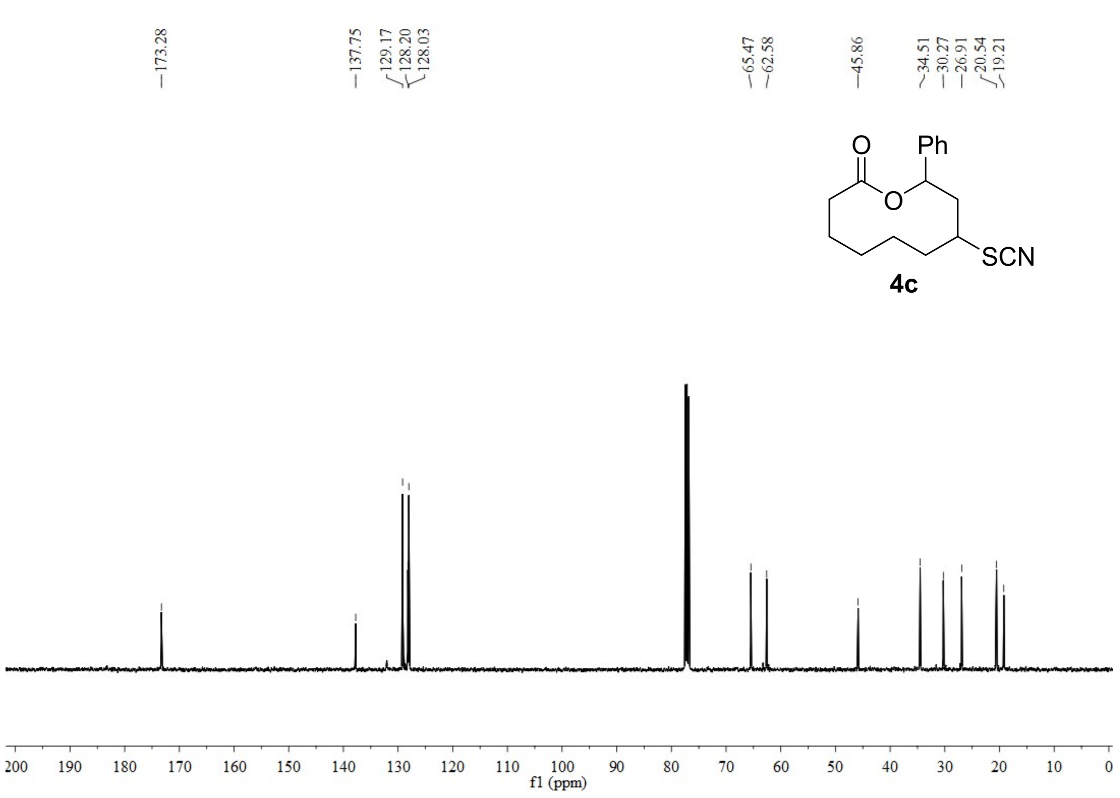

^{1}H NMR (400 MHz, $CDCl_3$) and ^{13}C NMR (100 MHz, $CDCl_3$) spectra of product **4d**

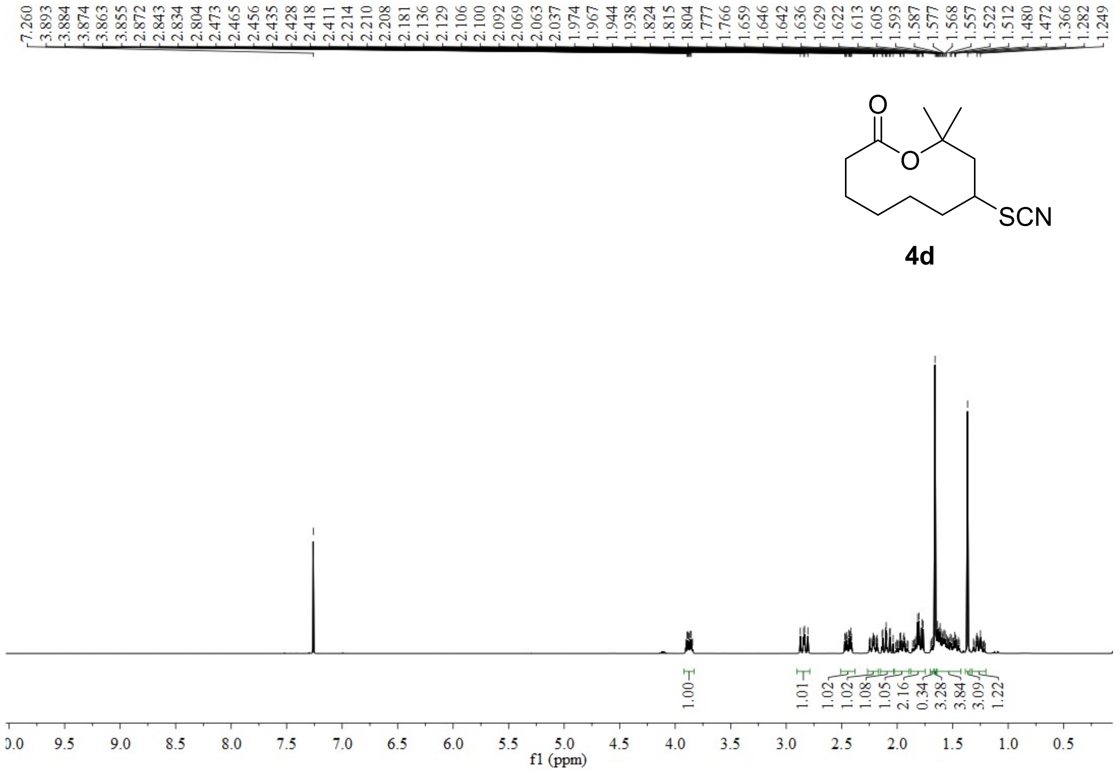

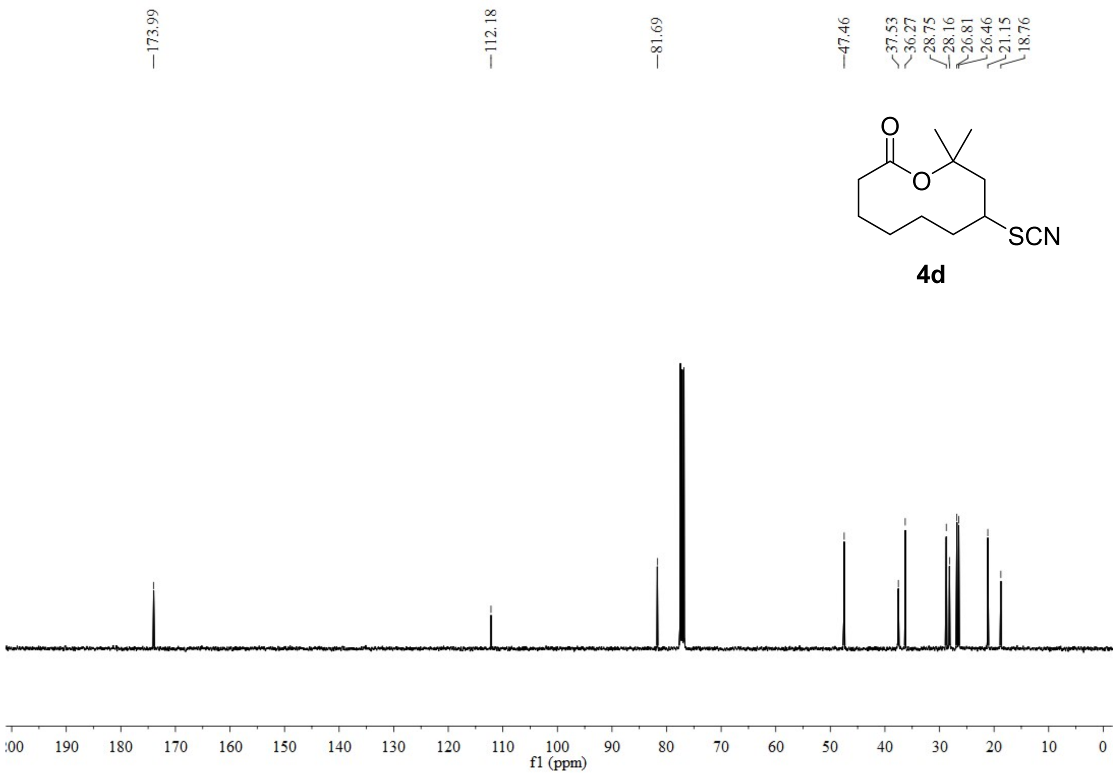

^{1}H NMR (400 MHz, $CDCl_3$) and ^{13}C NMR (100 MHz, $CDCl_3$) spectra of product **4e**

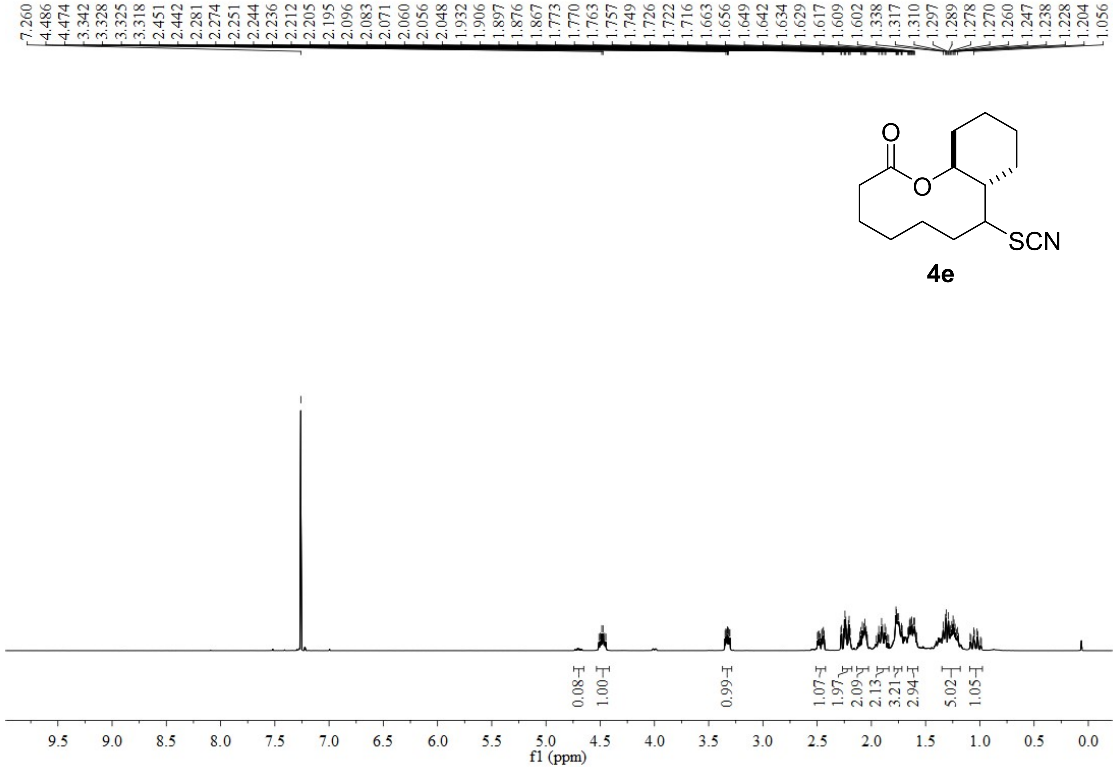

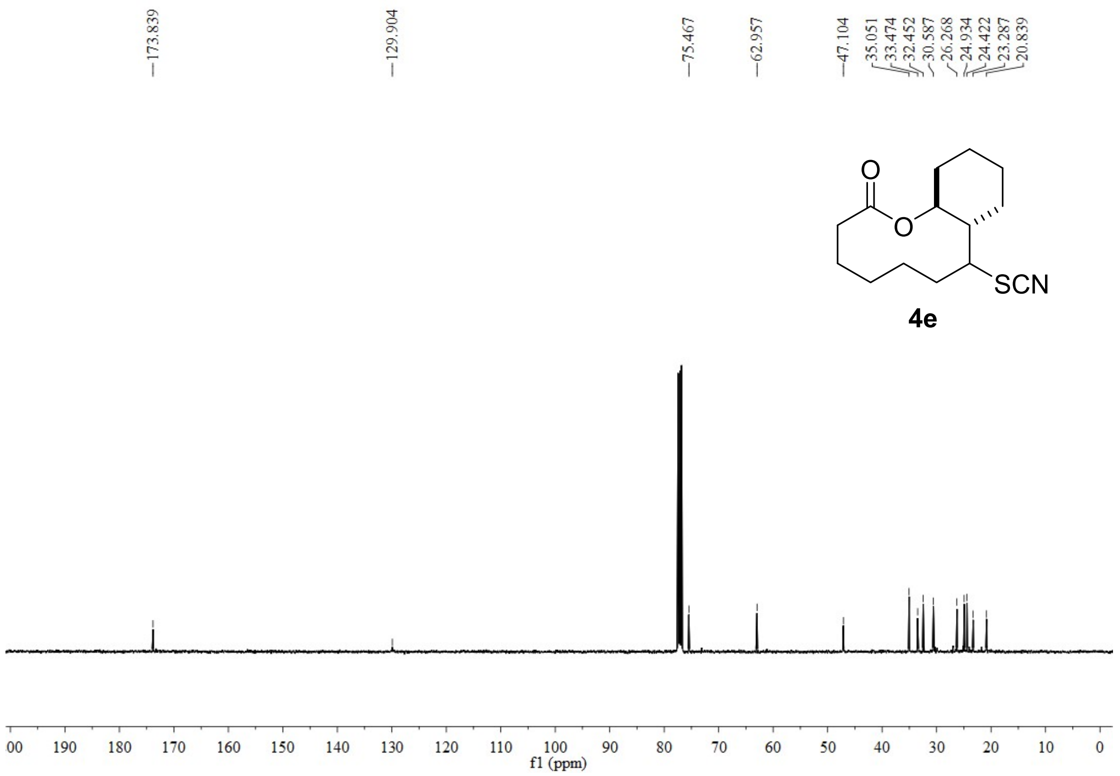

^{1}H NMR (400 MHz, $CDCl_3$) and ^{13}C NMR (100 MHz, $CDCl_3$) spectra of product **4f**

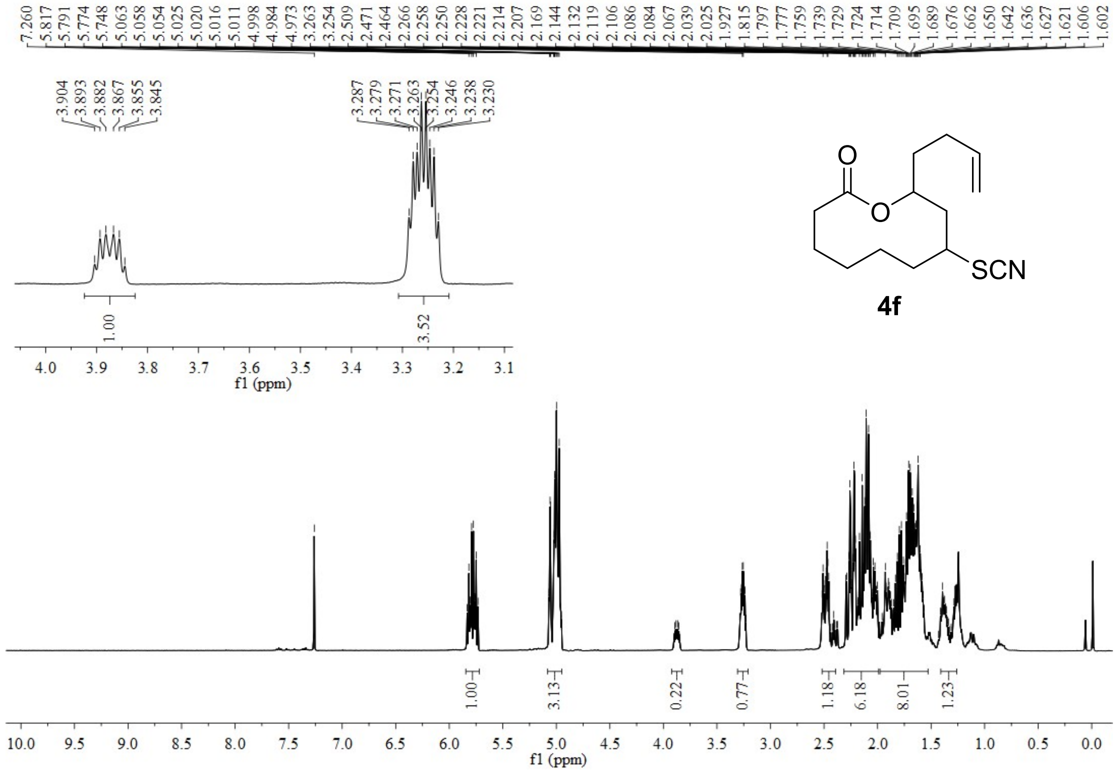

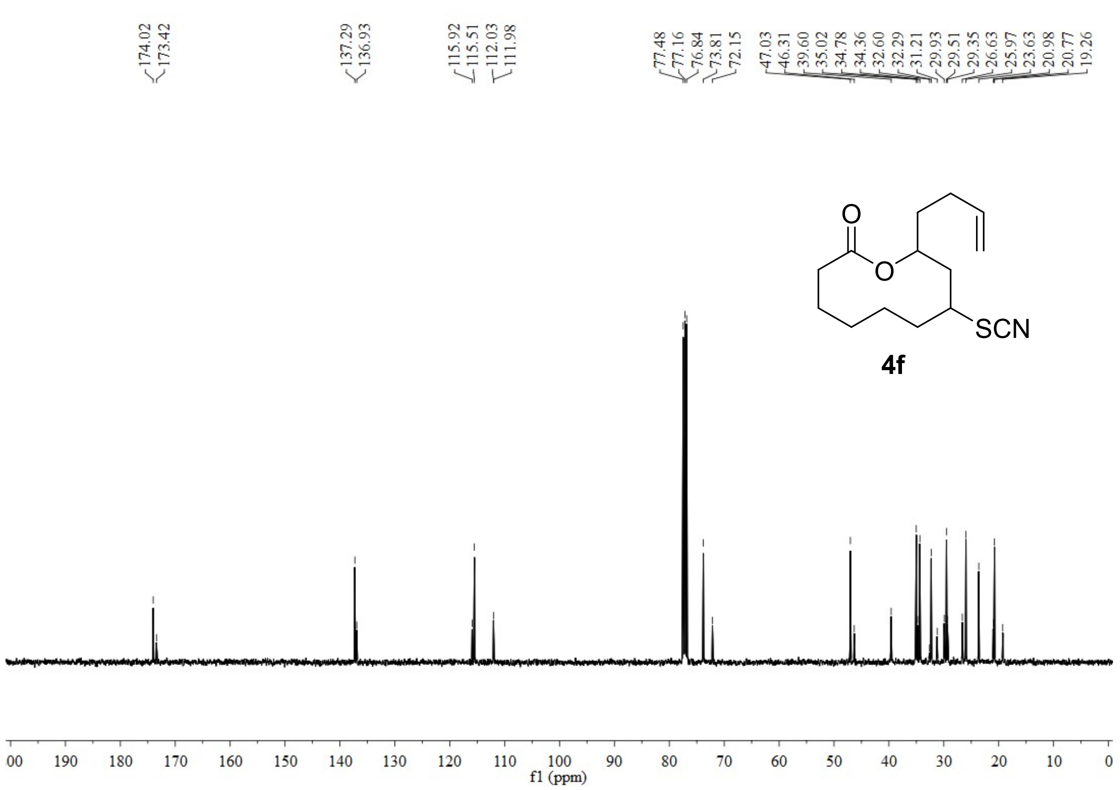

^{1}H NMR (400 MHz, $CDCl_3$) and ^{13}C NMR (100 MHz, $CDCl_3$) spectra of product **4g**

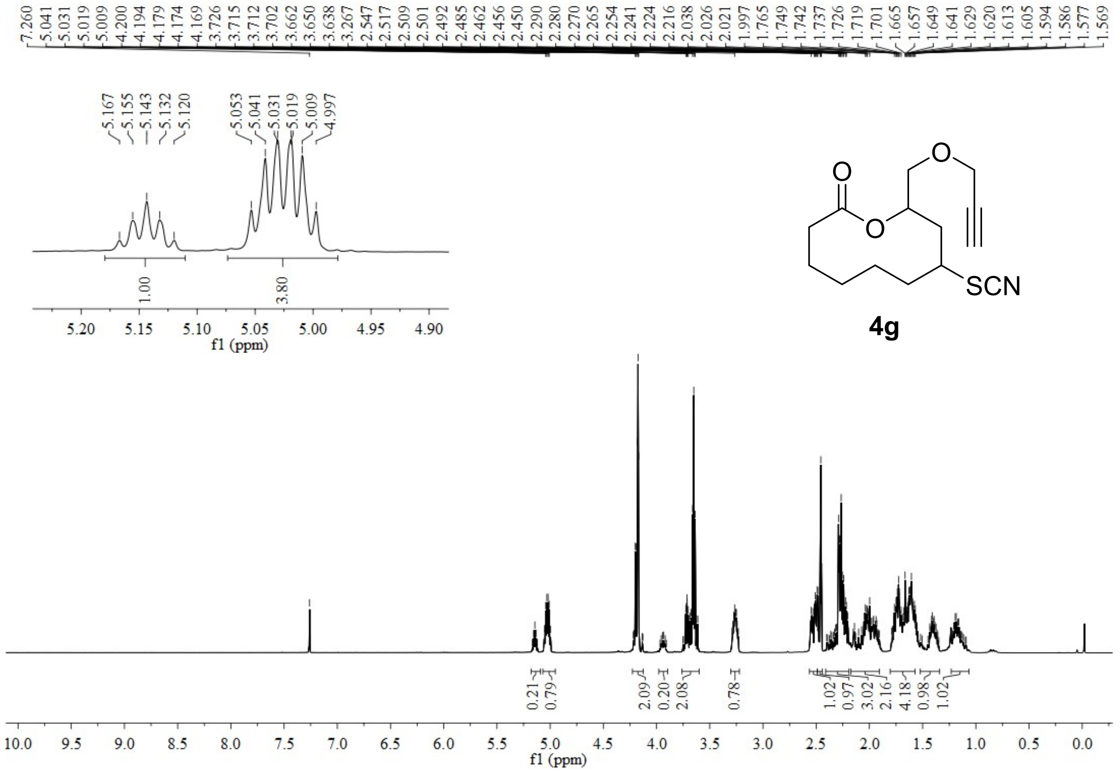

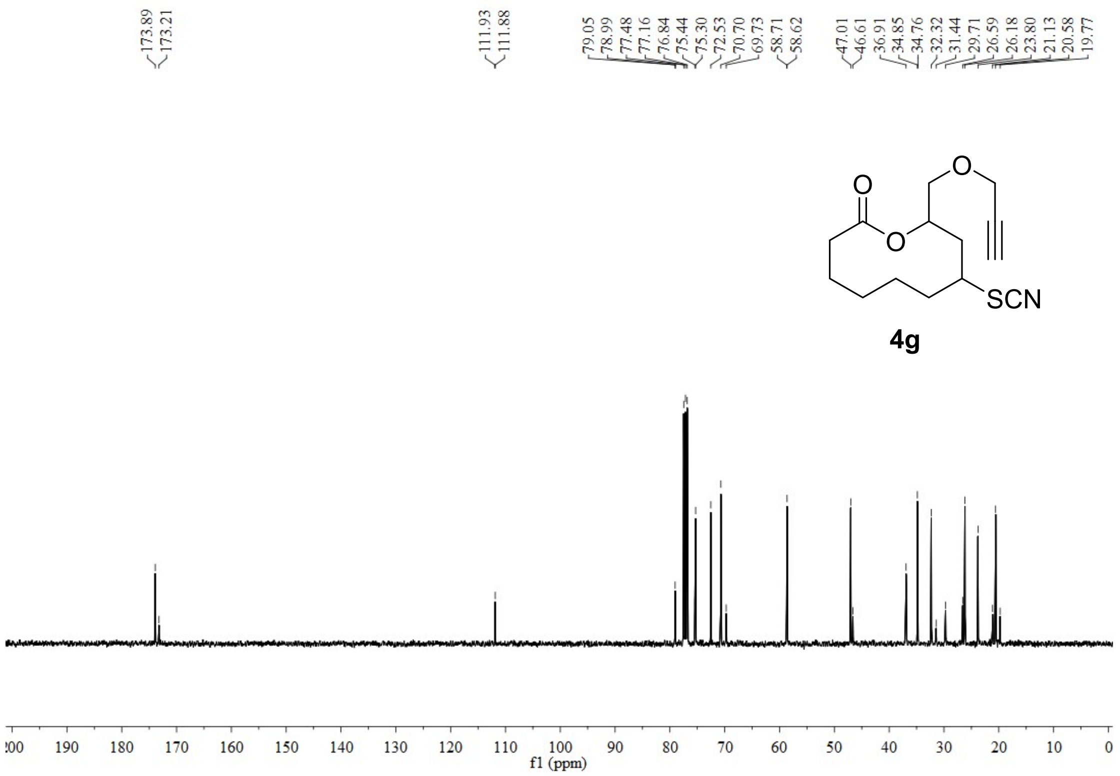

^{1}H NMR (400 MHz, $CDCl_3$) and ^{13}C NMR (100 MHz, $CDCl_3$) spectra of product **4h**

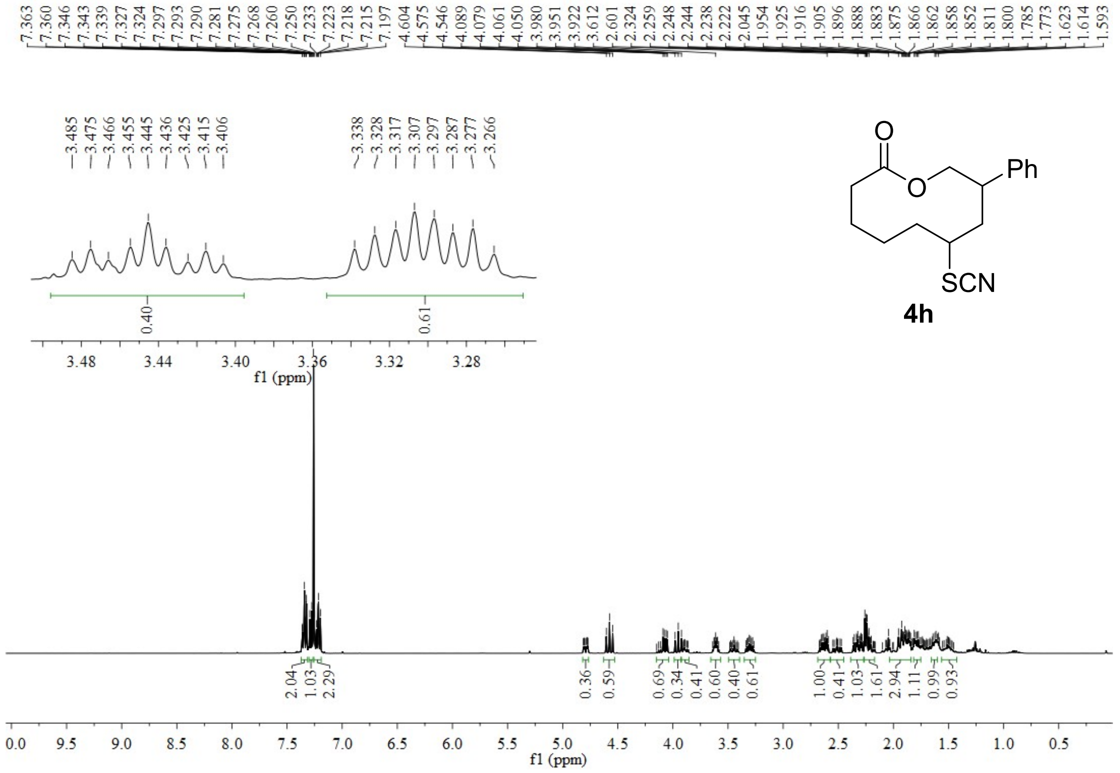

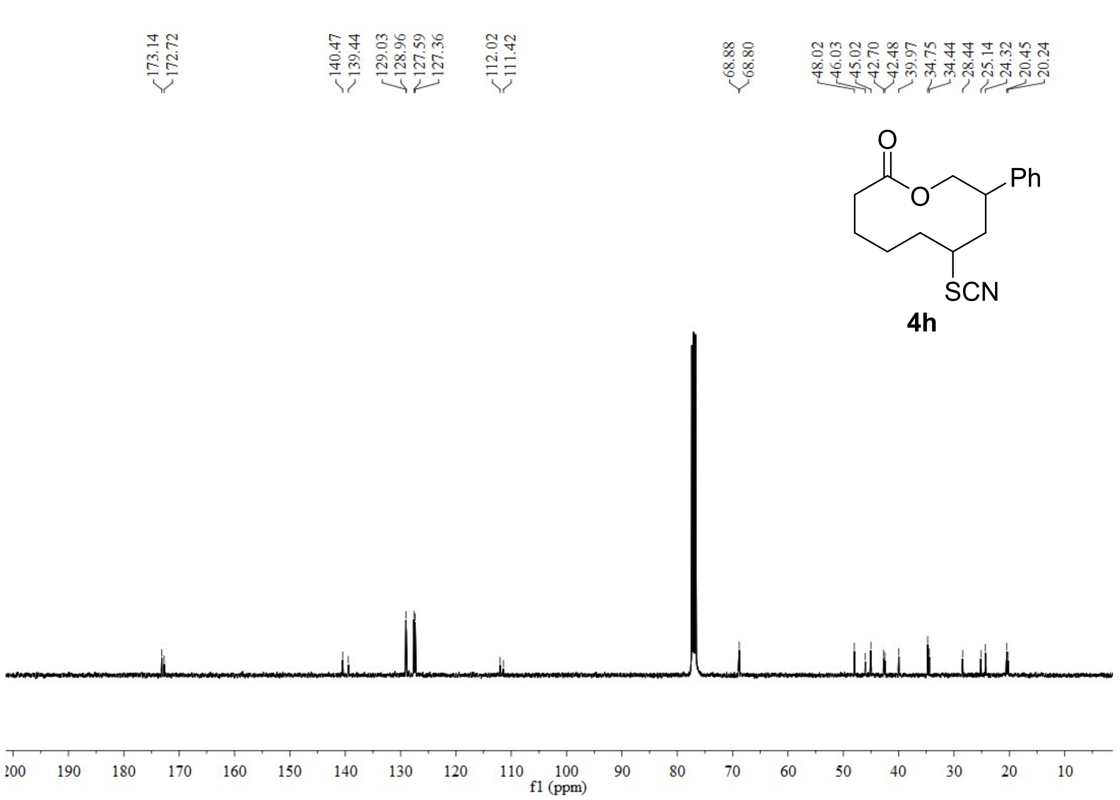

^1H NMR (400 MHz, $CDCl_3$) and ^{13}C NMR (100 MHz, $CDCl_3$) spectra of product **4i**

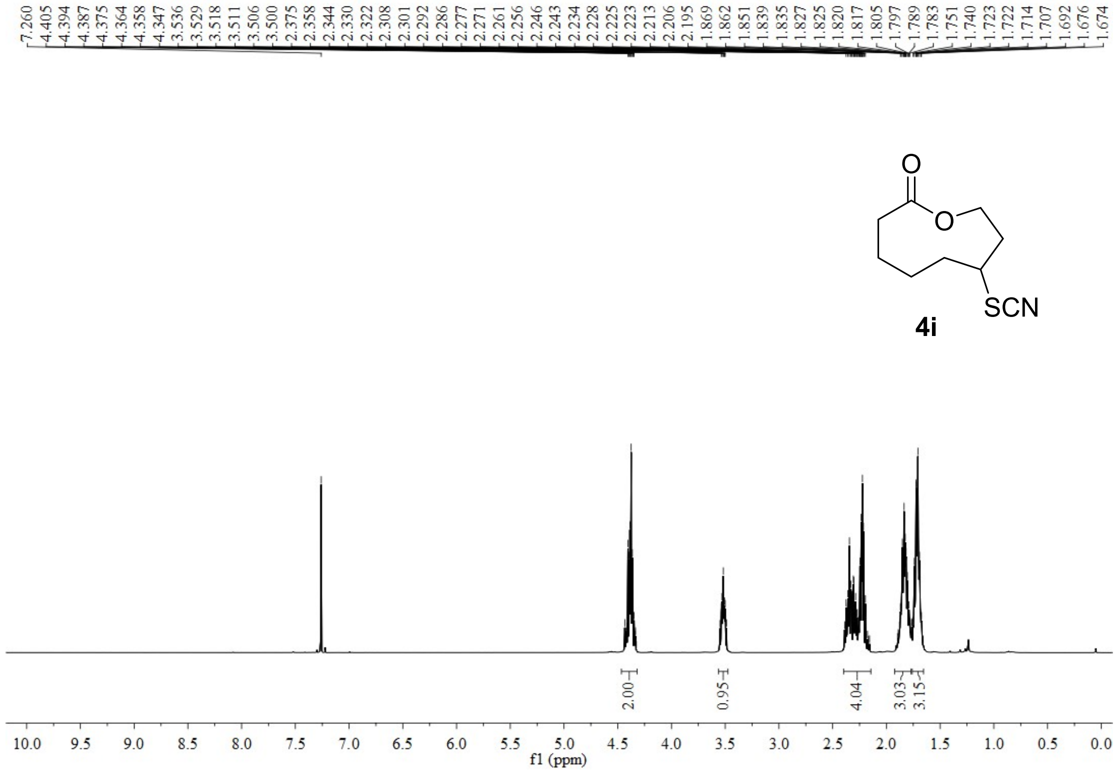

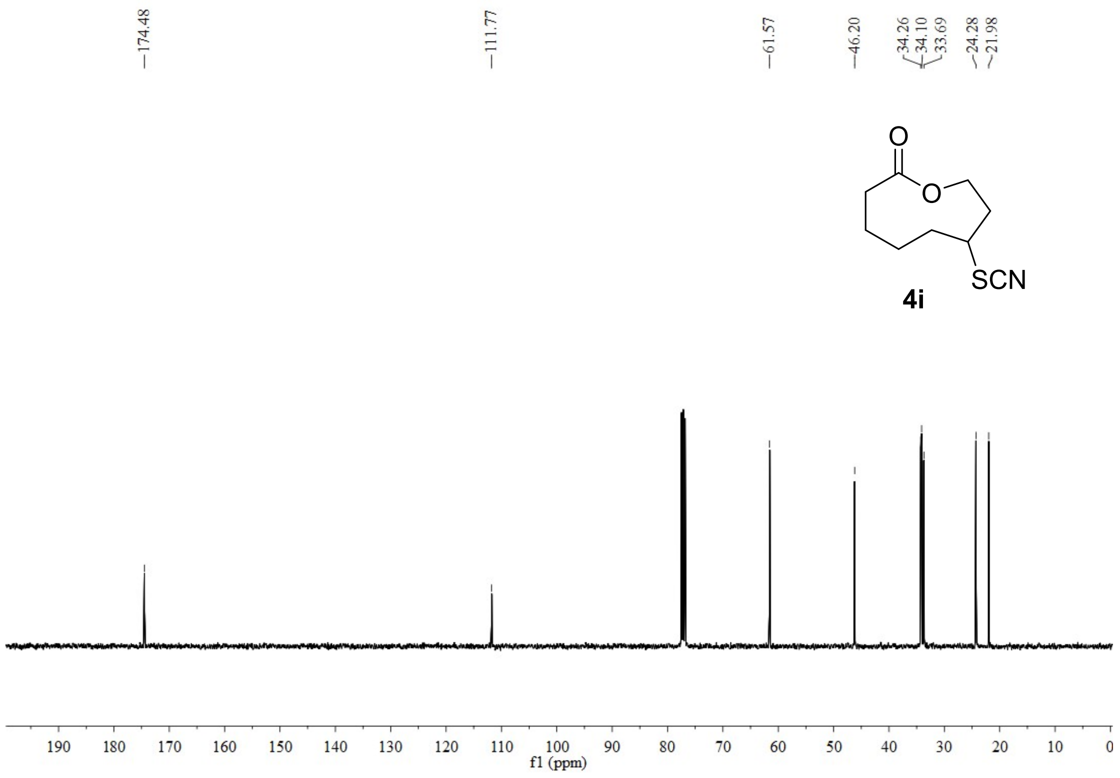

^{1}H NMR (400 MHz, $CDCl_3$) and ^{13}C NMR (100 MHz, $CDCl_3$) spectra of product **4j**

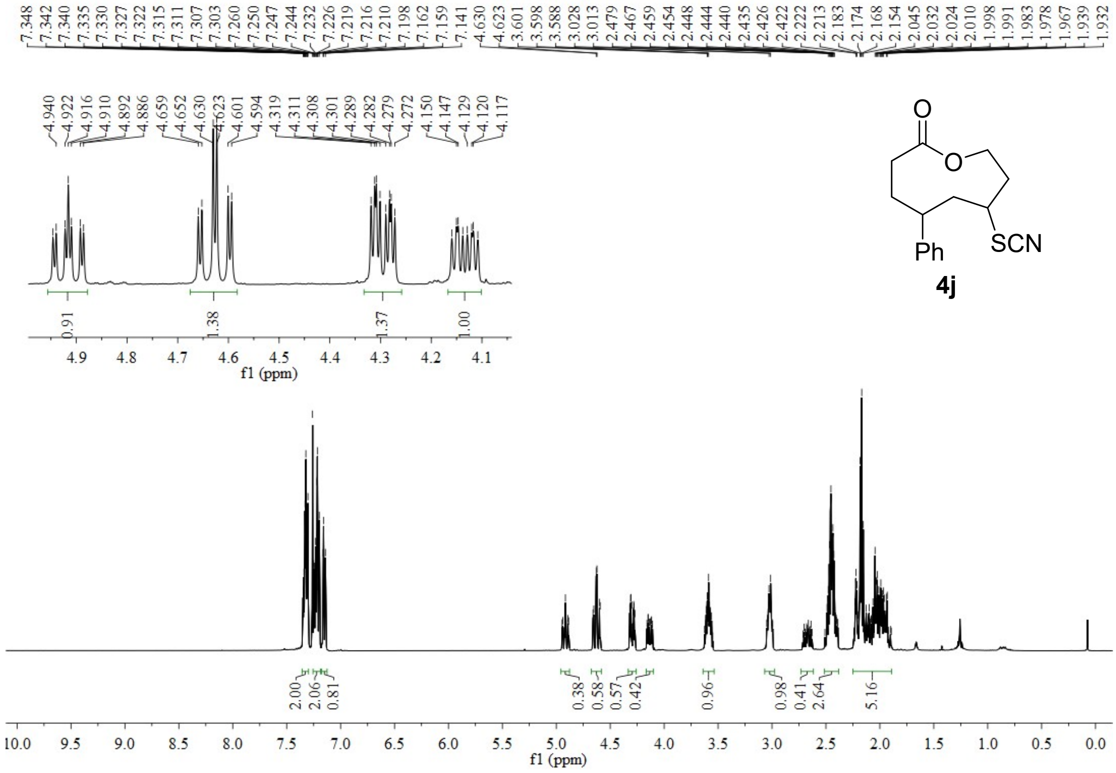

4j

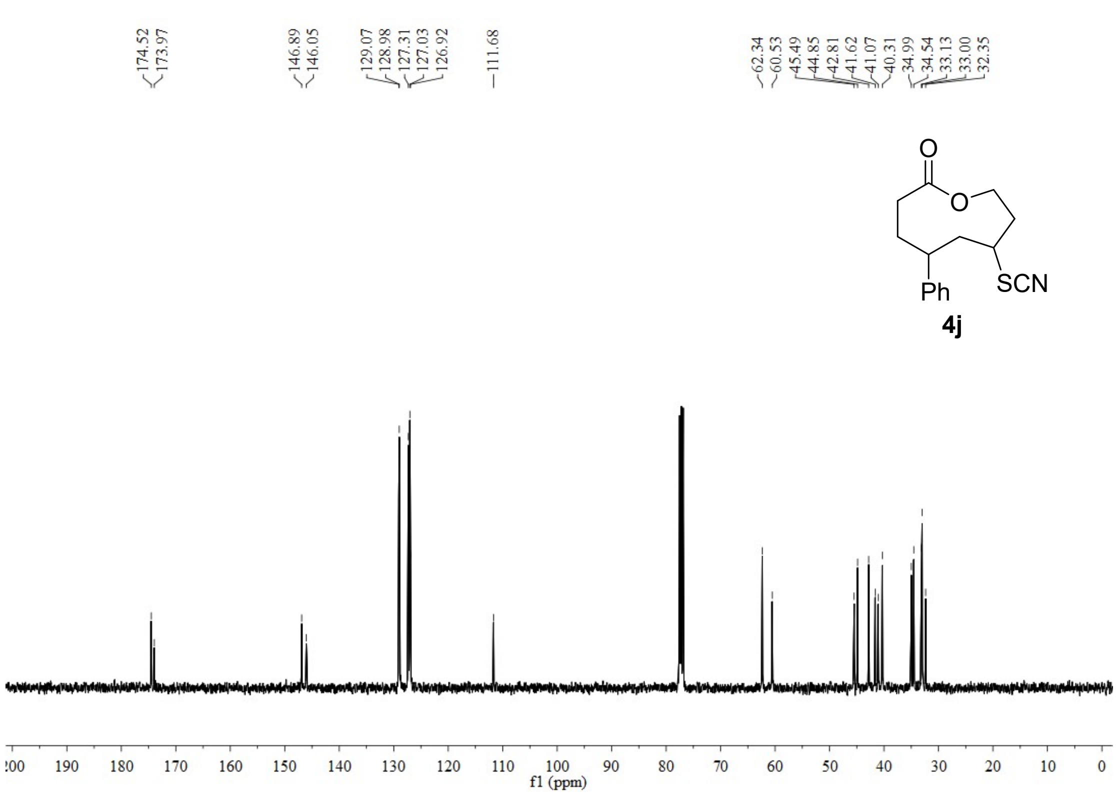

4j

^{1}H NMR (400 MHz, $CDCl_3$) and ^{13}C NMR (100 MHz, $CDCl_3$) spectra of product **4k**

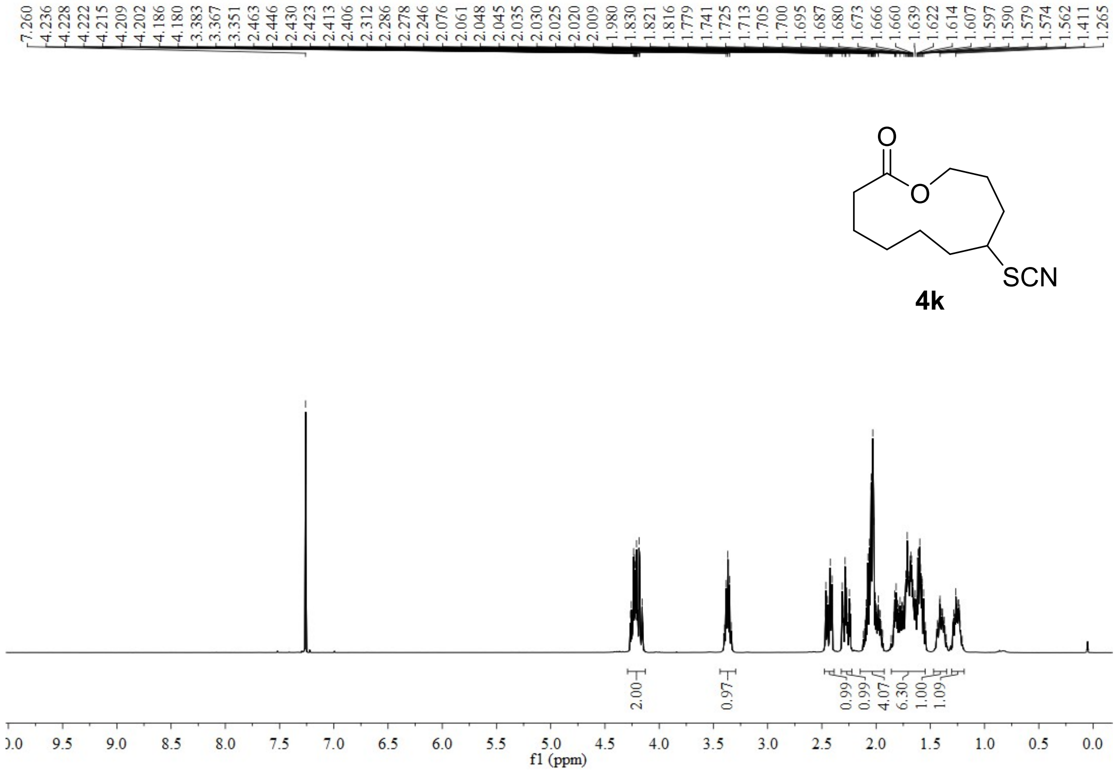

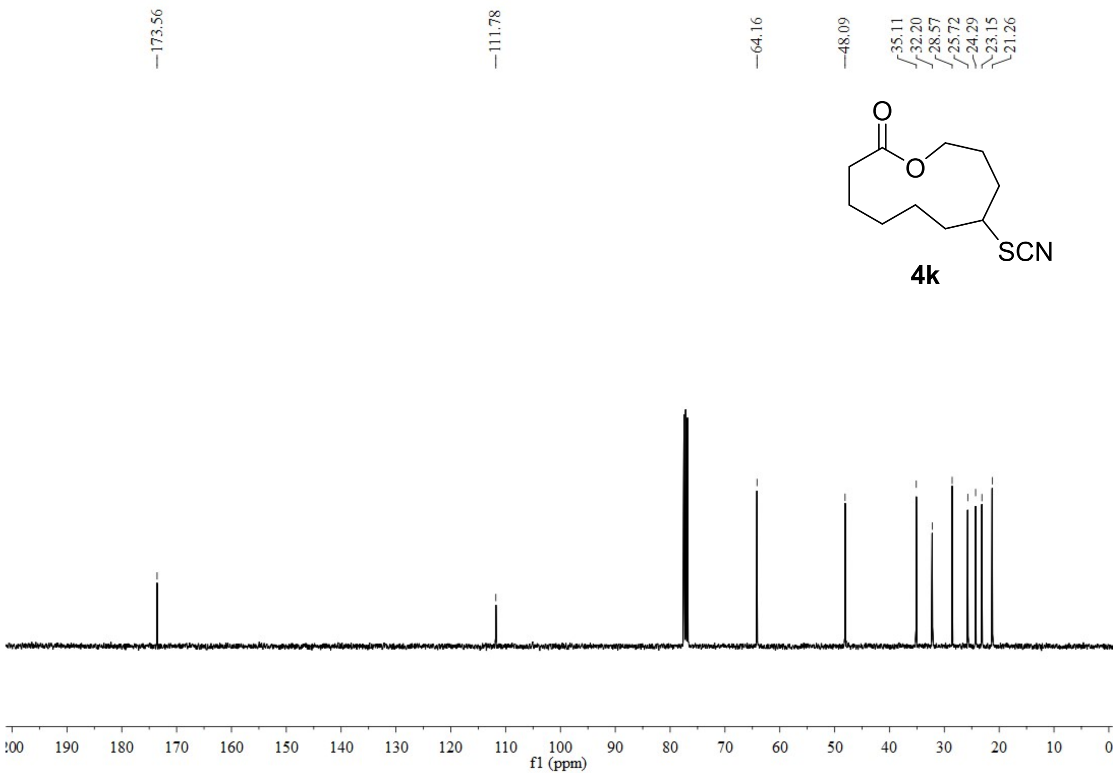

^{1}H NMR (400 MHz, $CDCl_3$) and ^{13}C NMR (100 MHz, $CDCl_3$) spectra of product **4l**

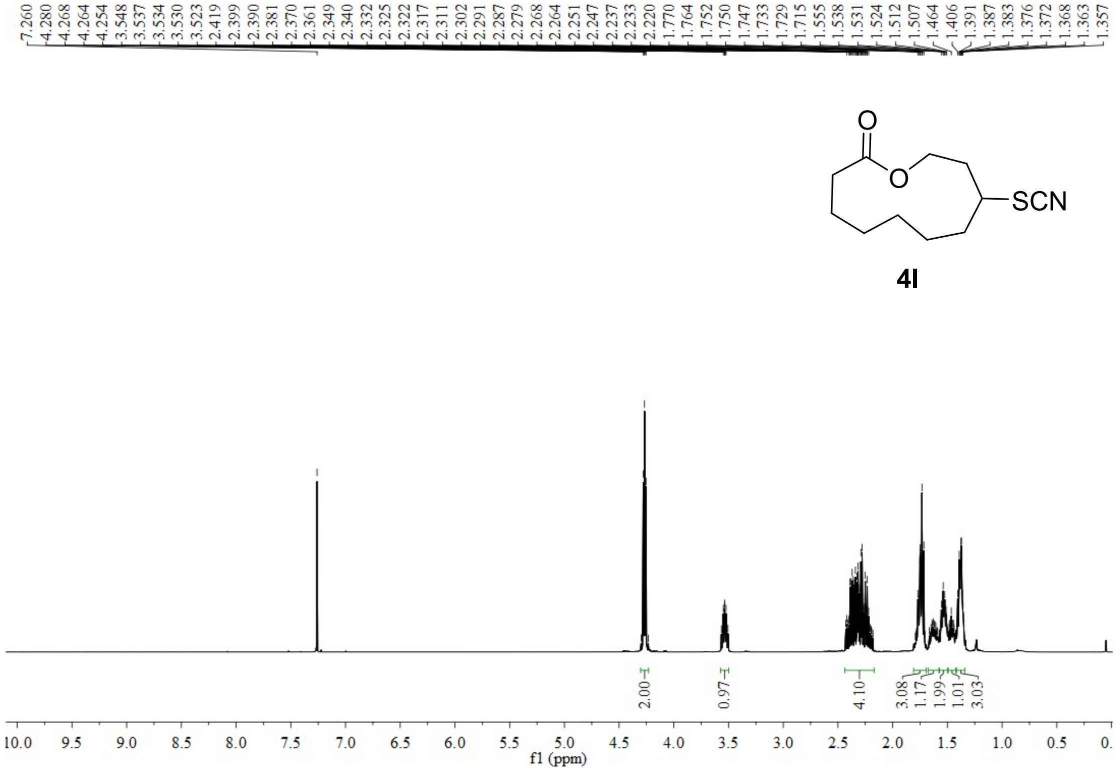

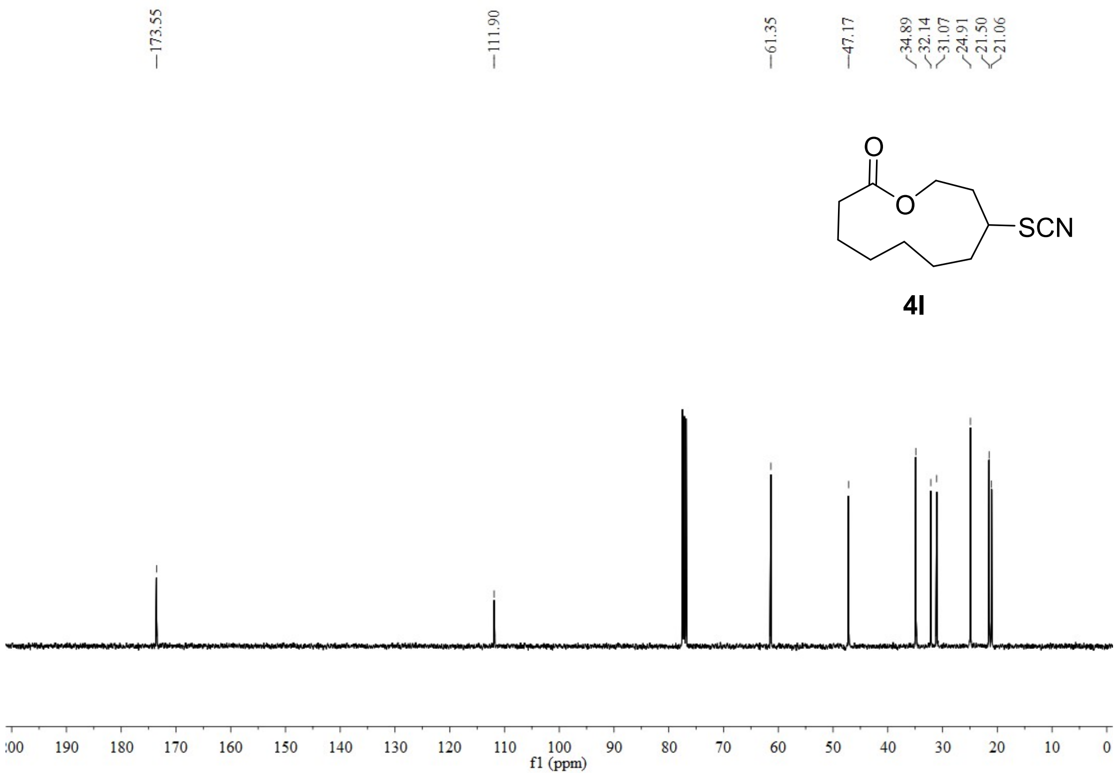

^{1}H NMR (400 MHz, $CDCl_3$) and ^{13}C NMR (100 MHz, $CDCl_3$) spectra of product **4m**

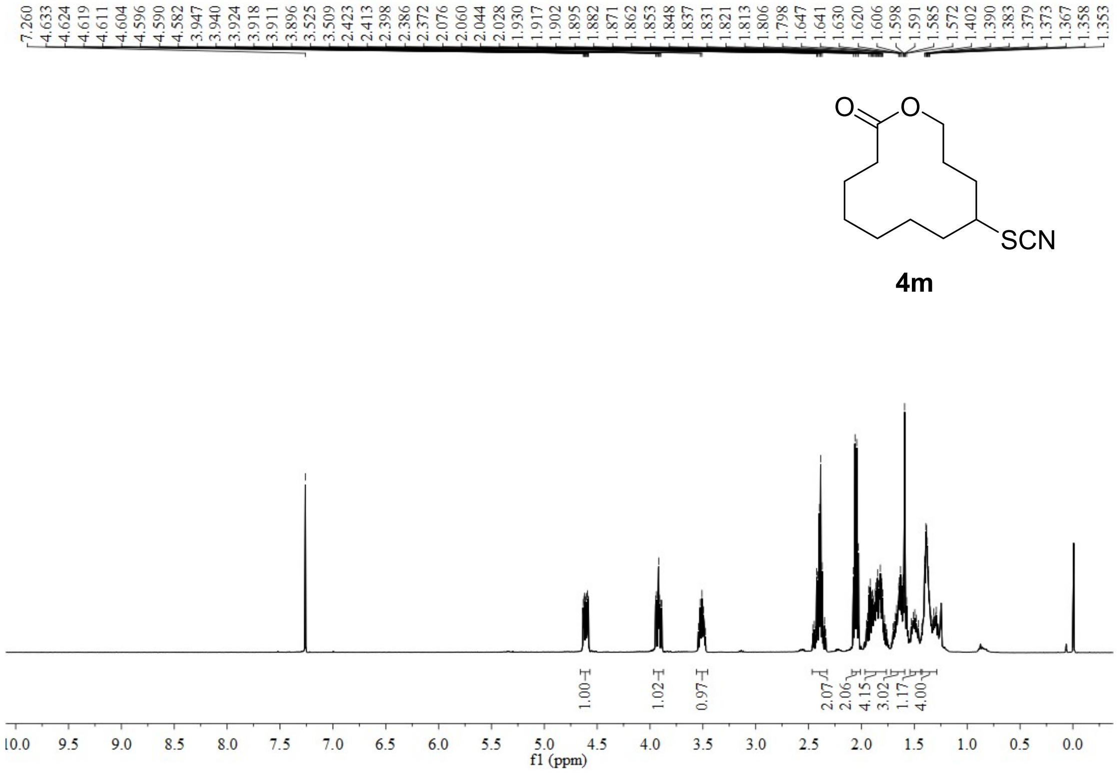

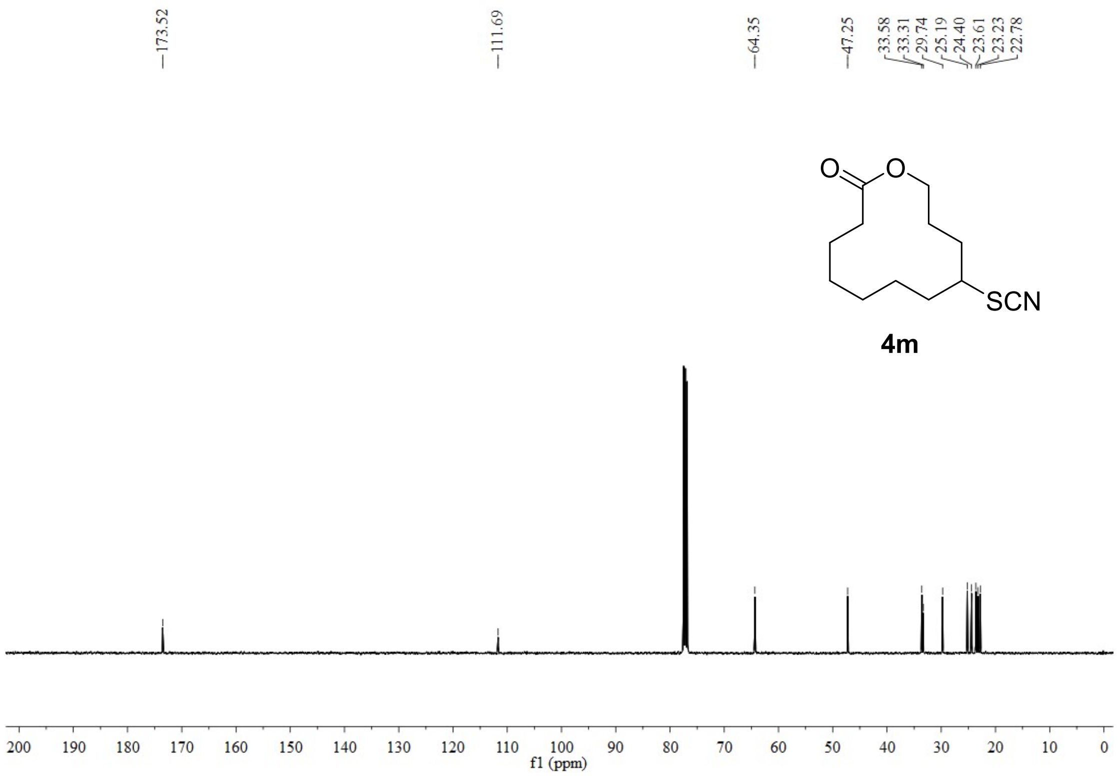

^{1}H NMR (400 MHz, $CDCl_3$) and ^{13}C NMR (100 MHz, $CDCl_3$) spectra of product **4n**

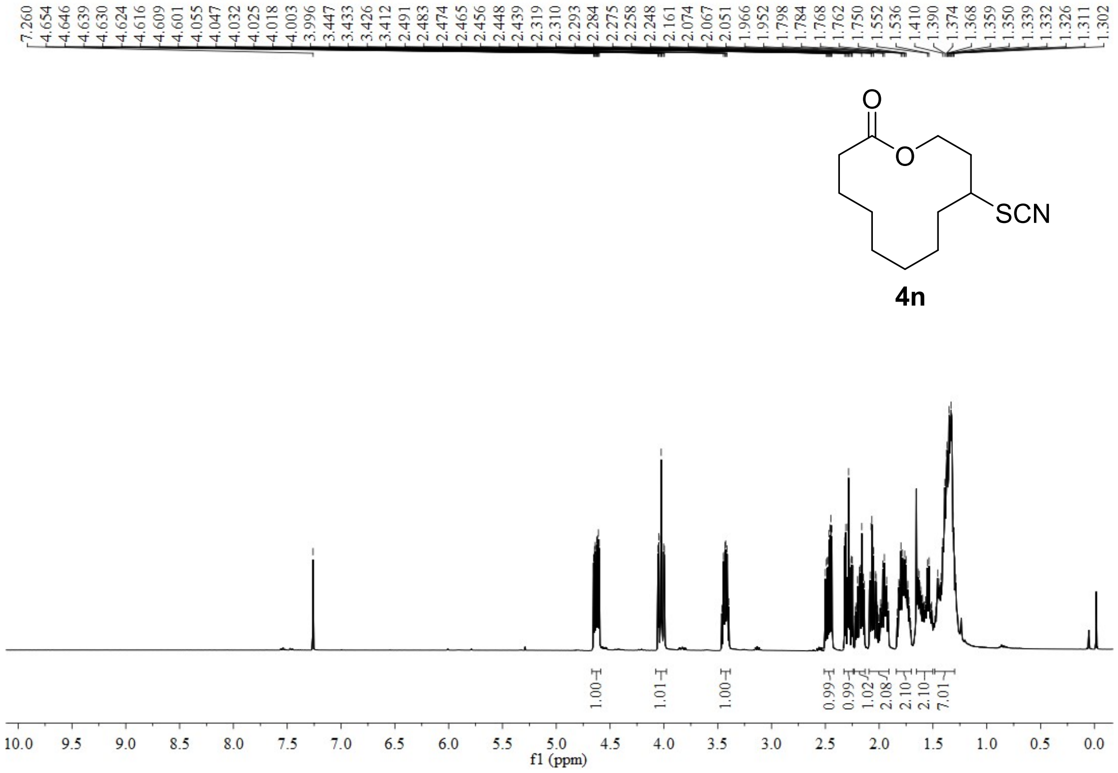

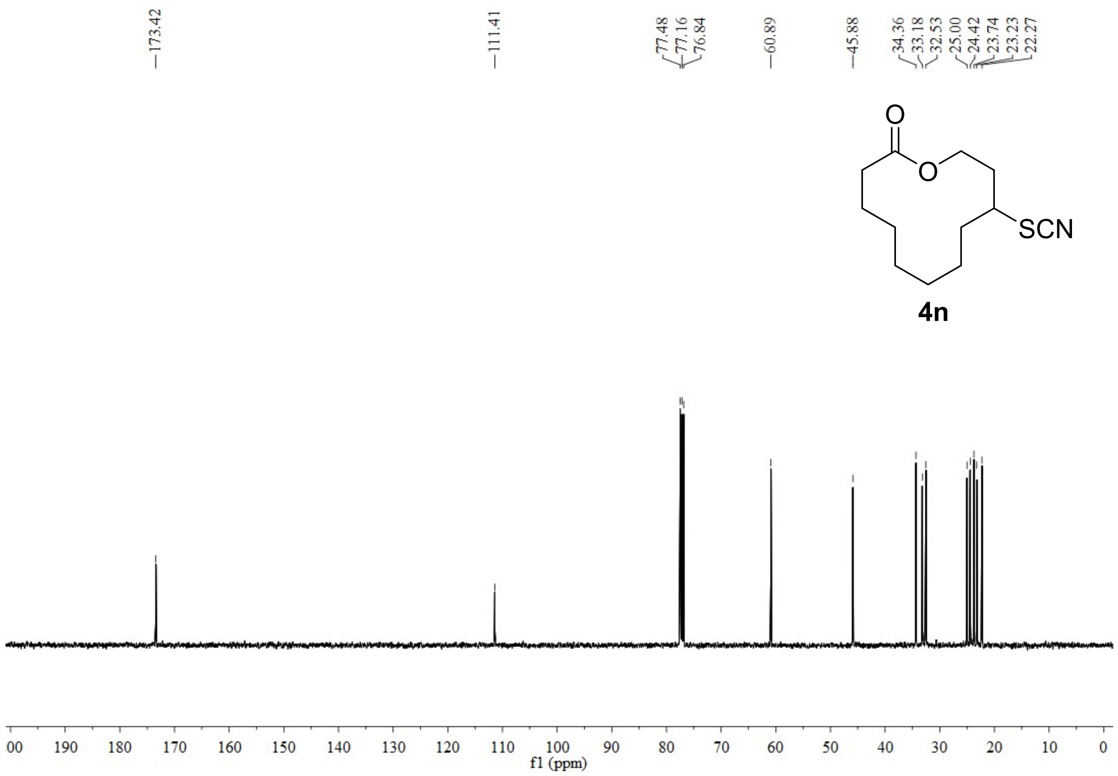

^{1}H NMR (400 MHz, $CDCl_3$) and ^{13}C NMR (100 MHz, $CDCl_3$) spectra of product **4o**

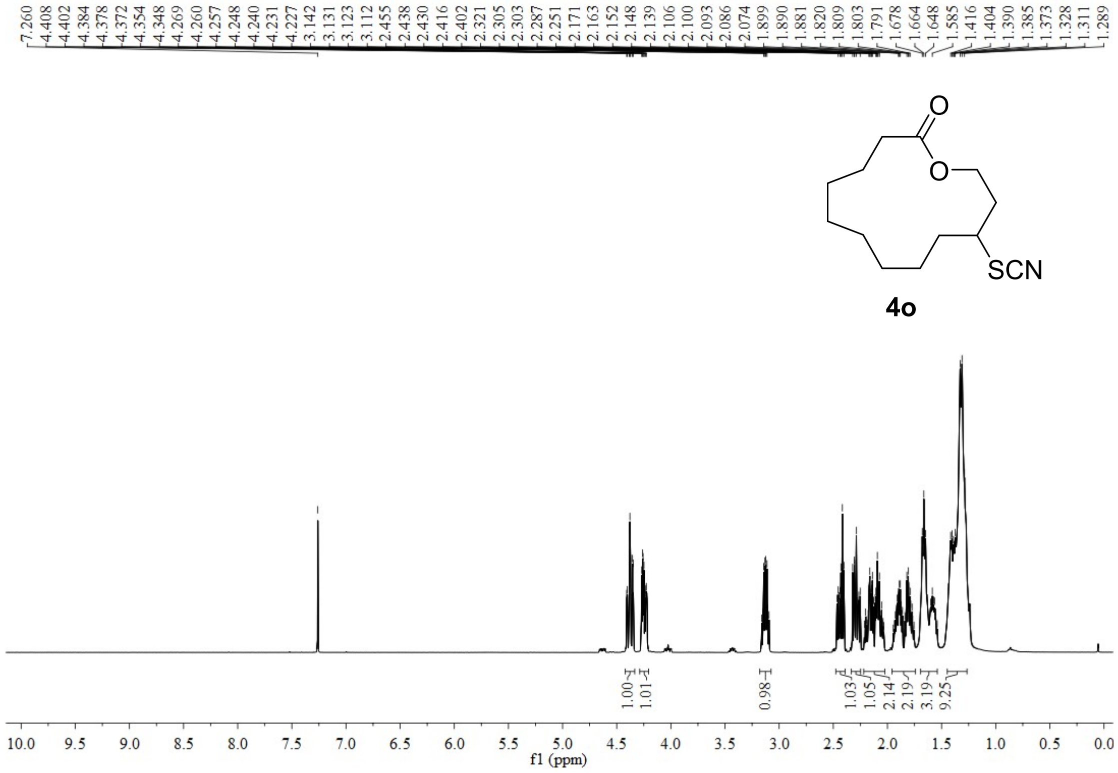

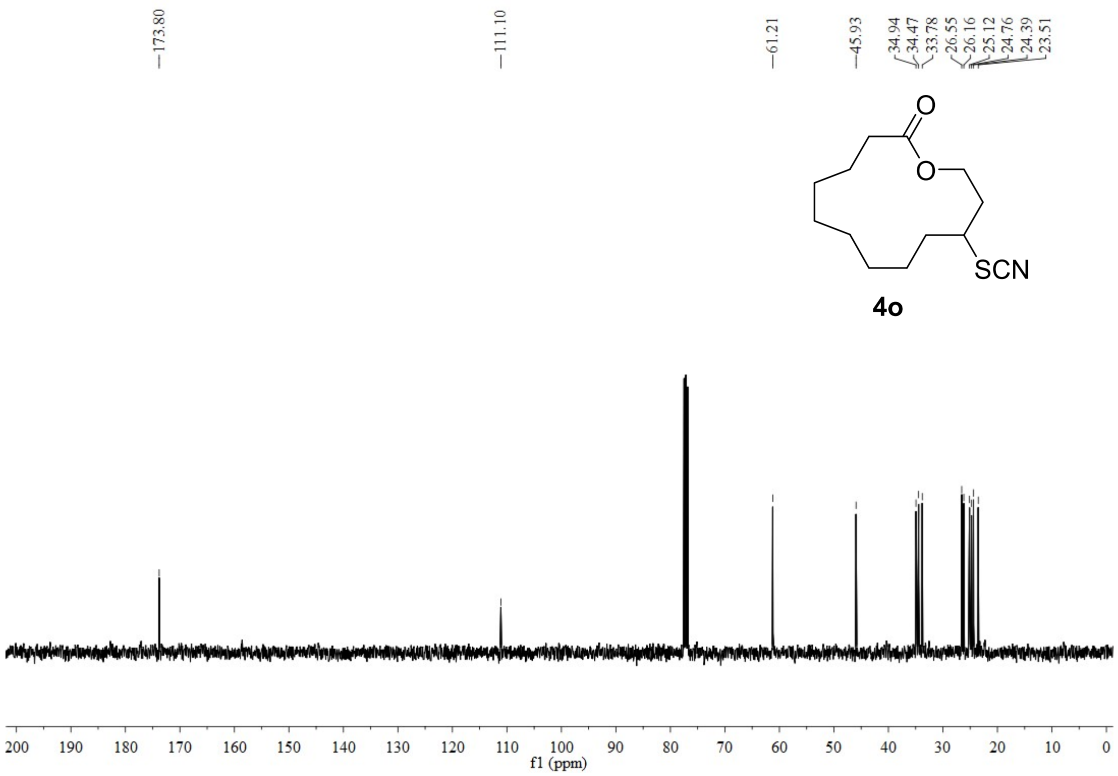

^{1}H NMR (400 MHz, $CDCl_3$) and ^{13}C NMR (100 MHz, $CDCl_3$) spectra of product **4p**

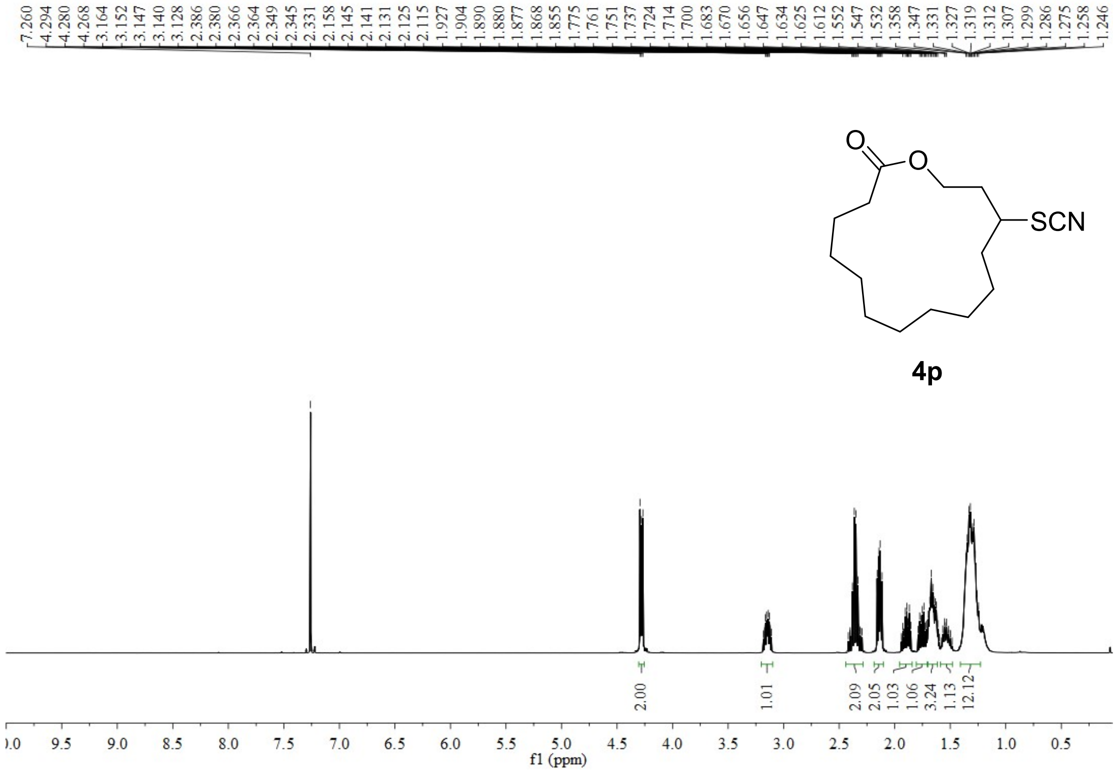

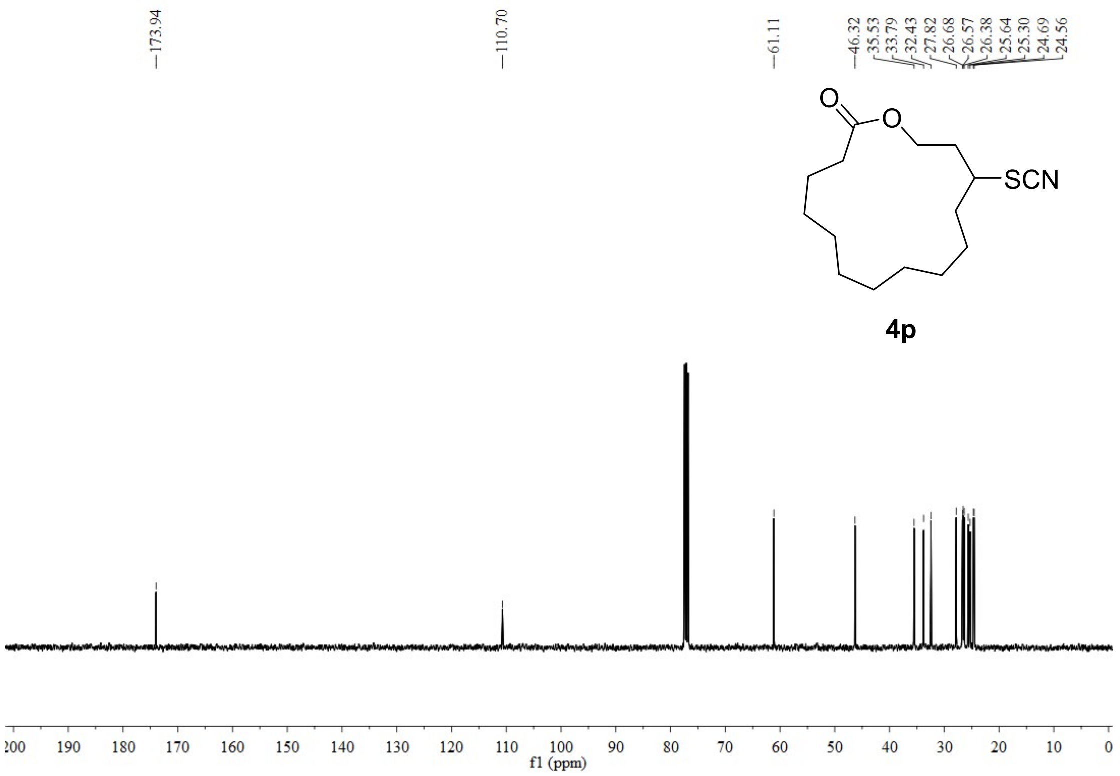

^{1}H NMR (400 MHz, $CDCl_3$) and ^{13}C NMR (100 MHz, $CDCl_3$) spectra of product **4q**

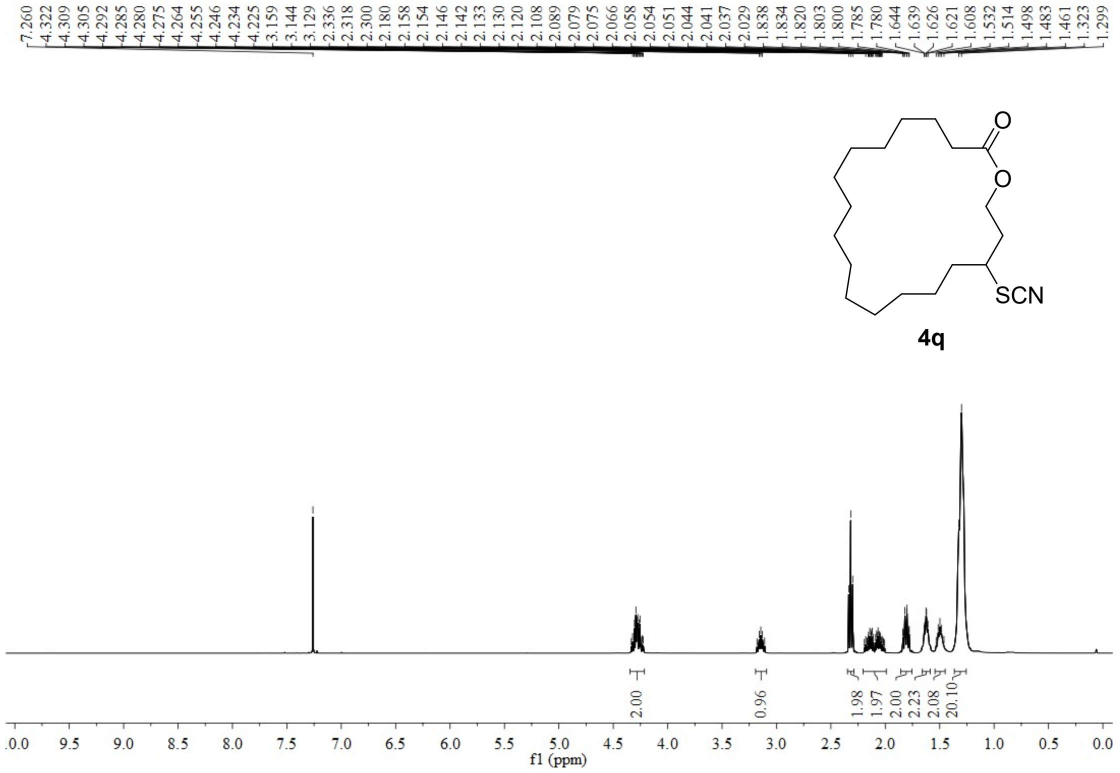

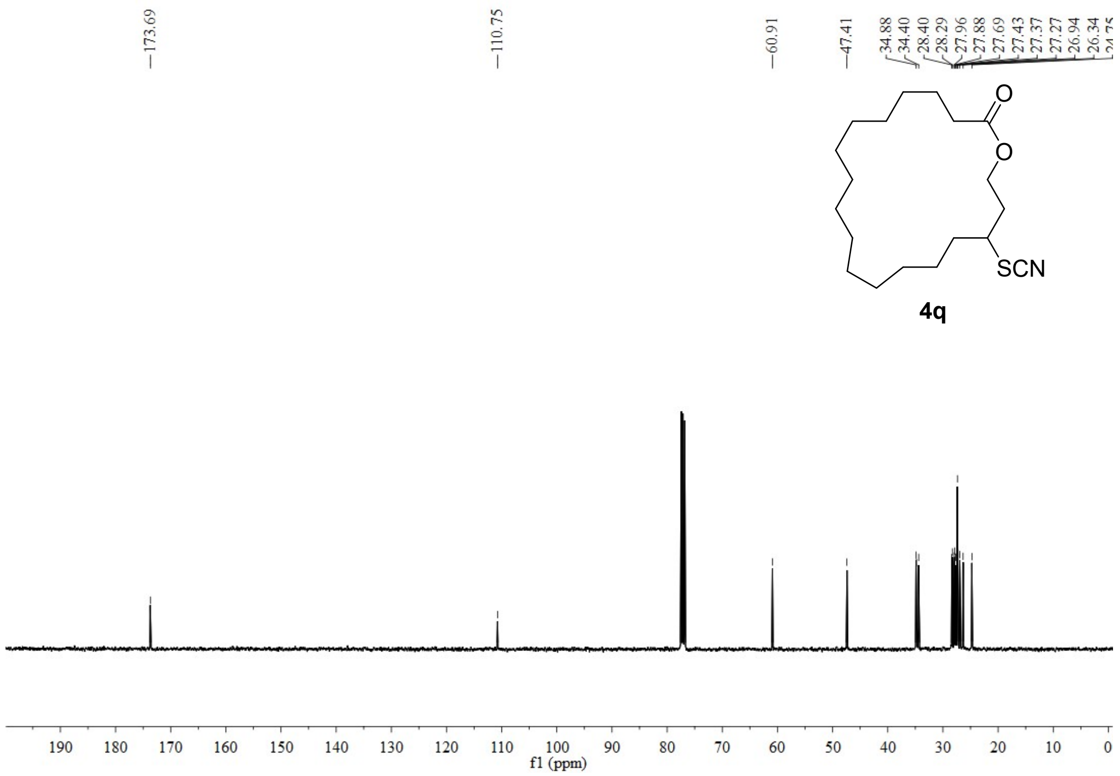

^{1}H NMR (400 MHz, $CDCl_3$) and ^{13}C NMR (100 MHz, $CDCl_3$) spectra of product **5a**

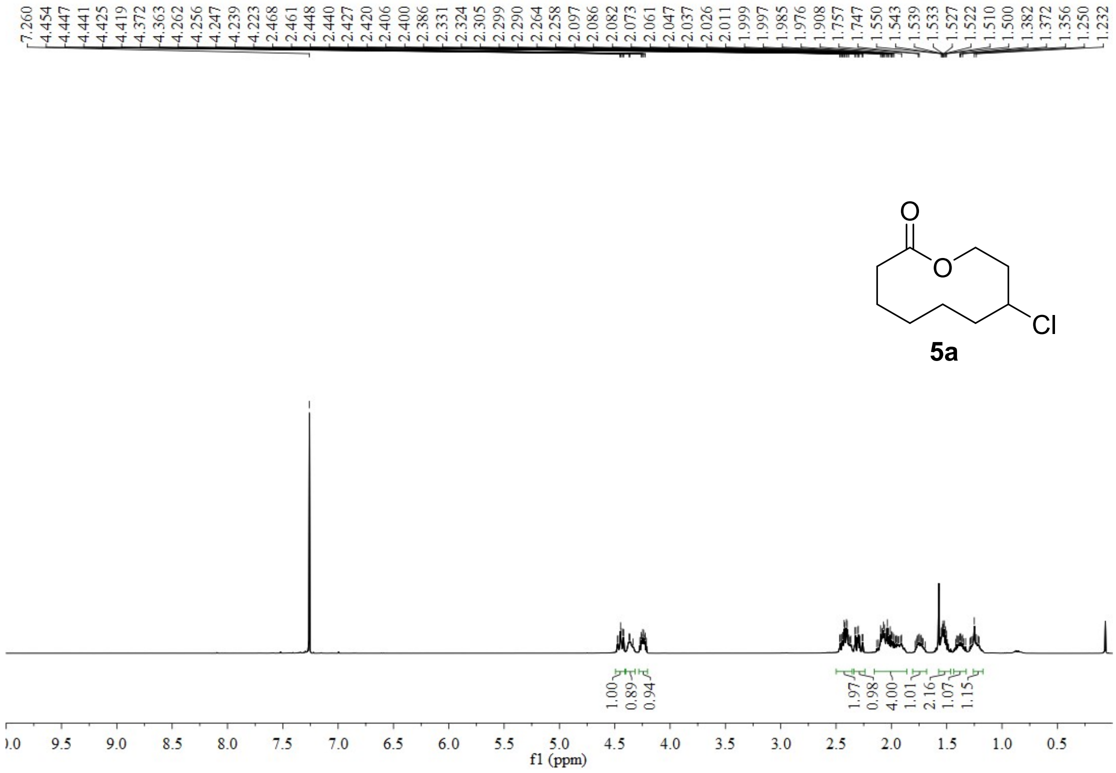

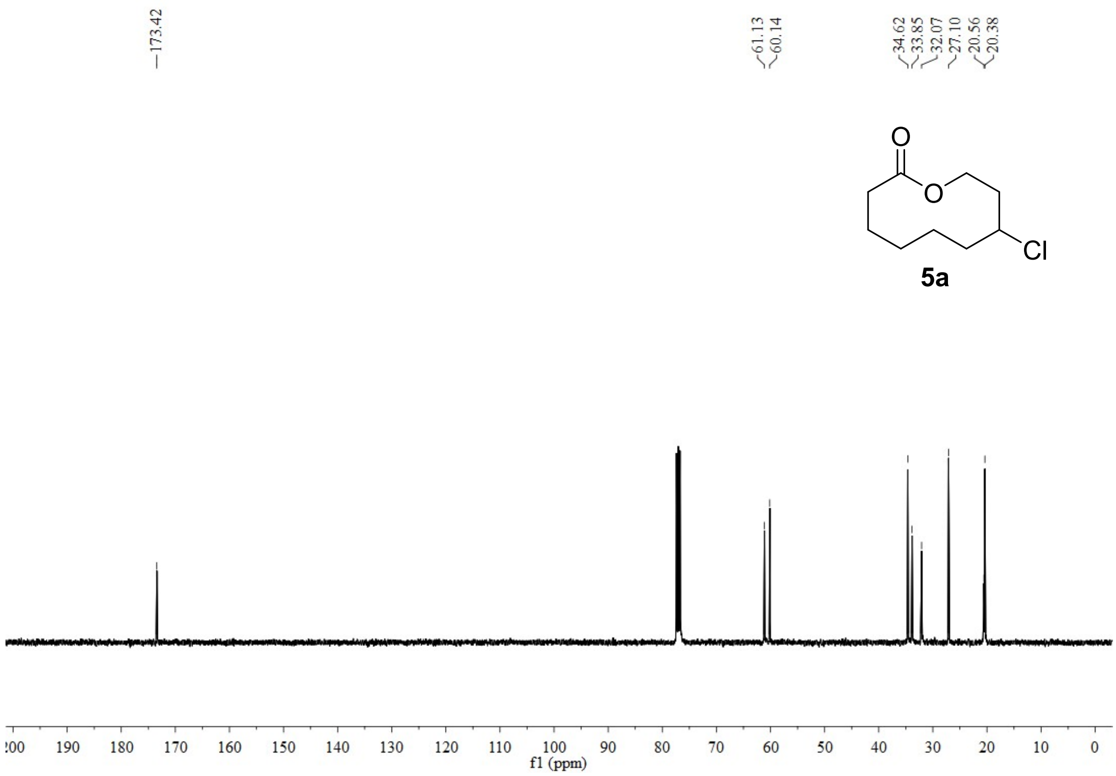

^{1}H NMR (400 MHz, $CDCl_3$) and ^{13}C NMR (100 MHz, $CDCl_3$) spectra of product **5a-2**

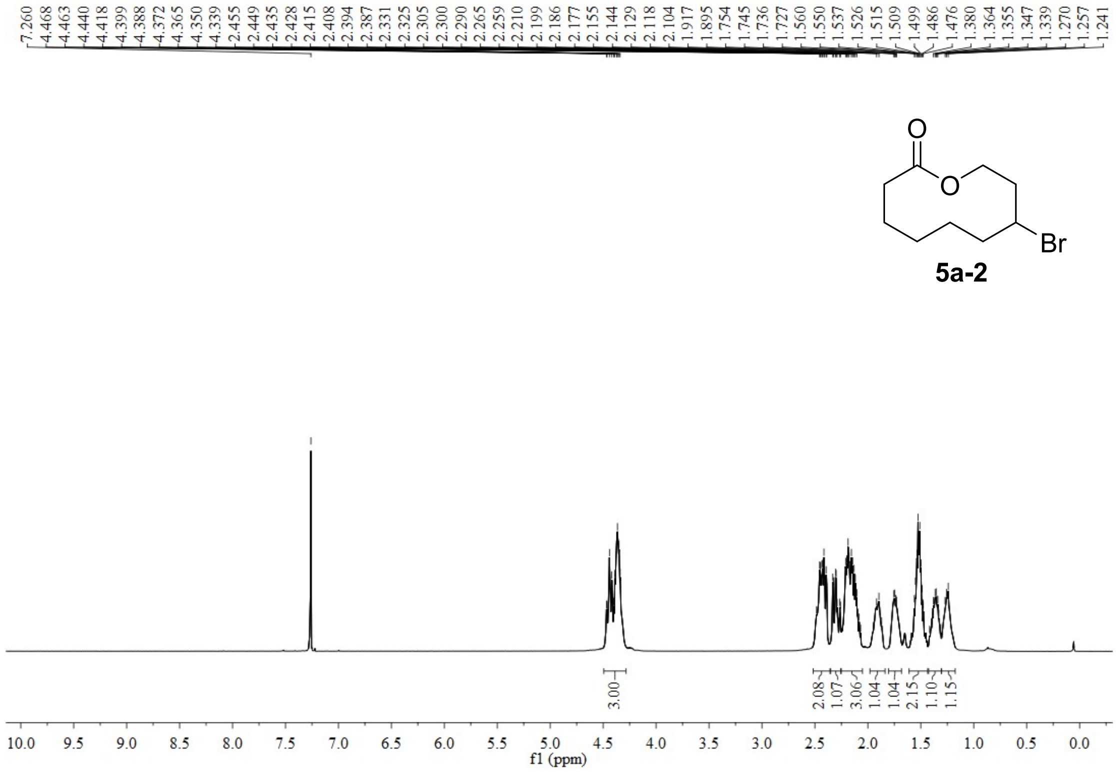

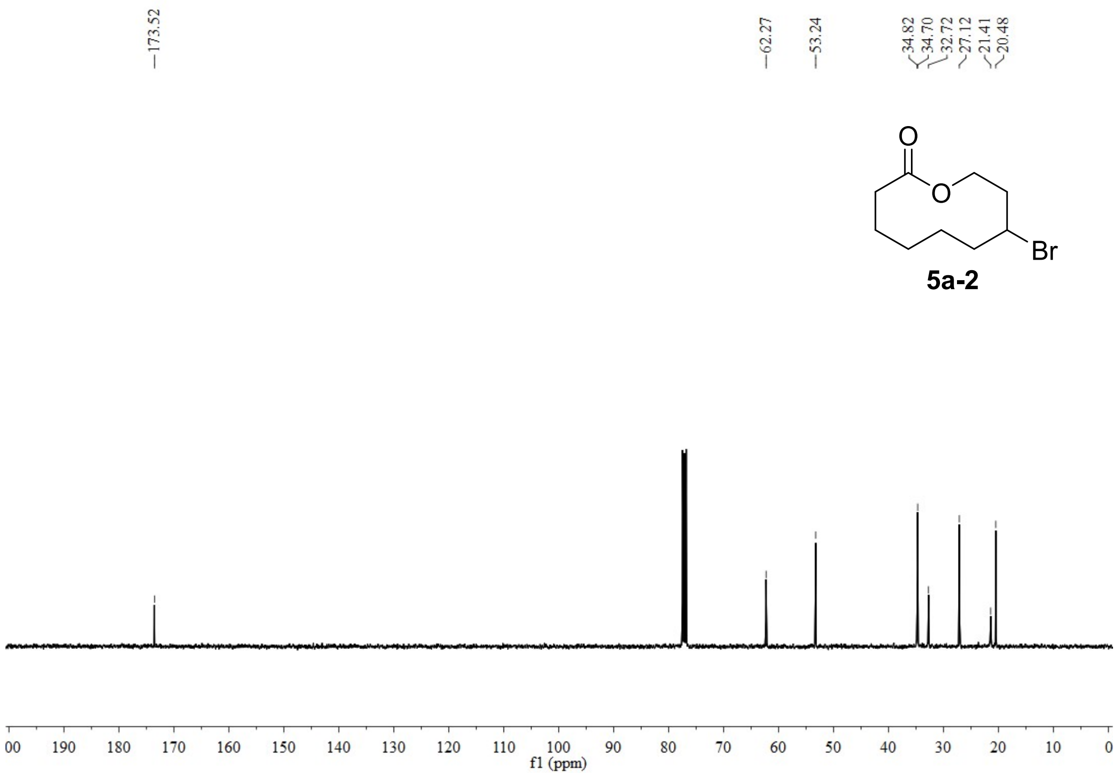

^{1}H NMR (400 MHz, $CDCl_3$) and ^{13}C NMR (100 MHz, $CDCl_3$) spectra of product **5a-3**

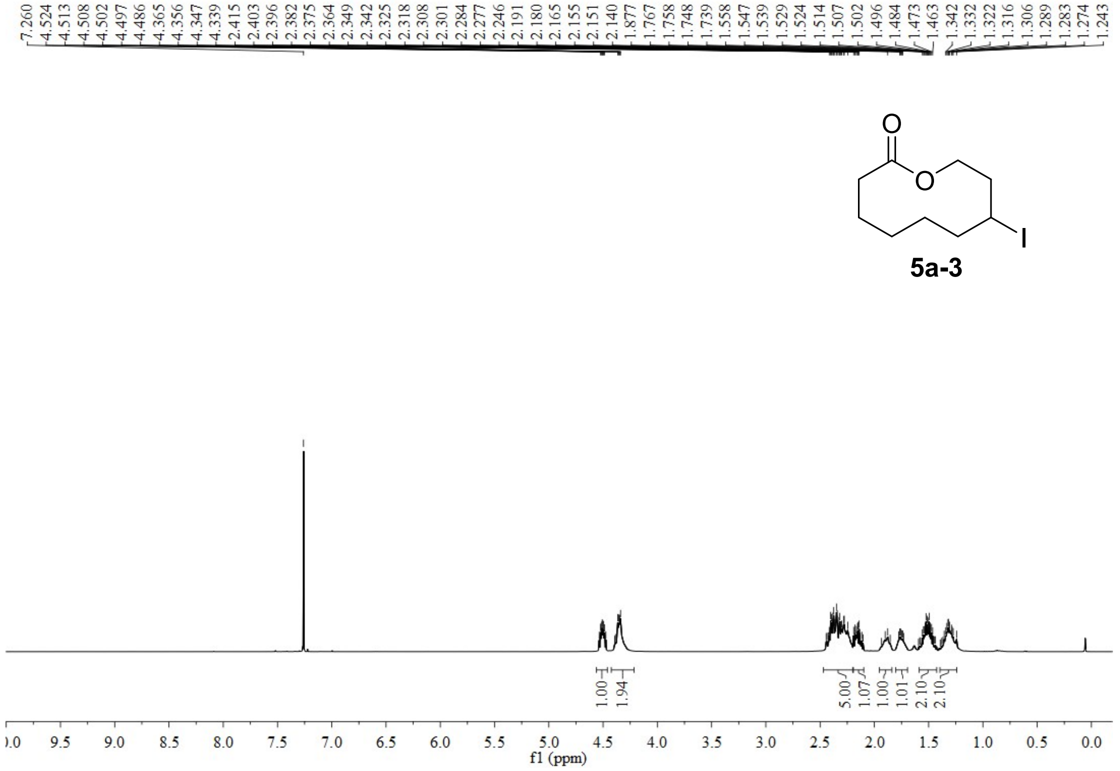

5a-3

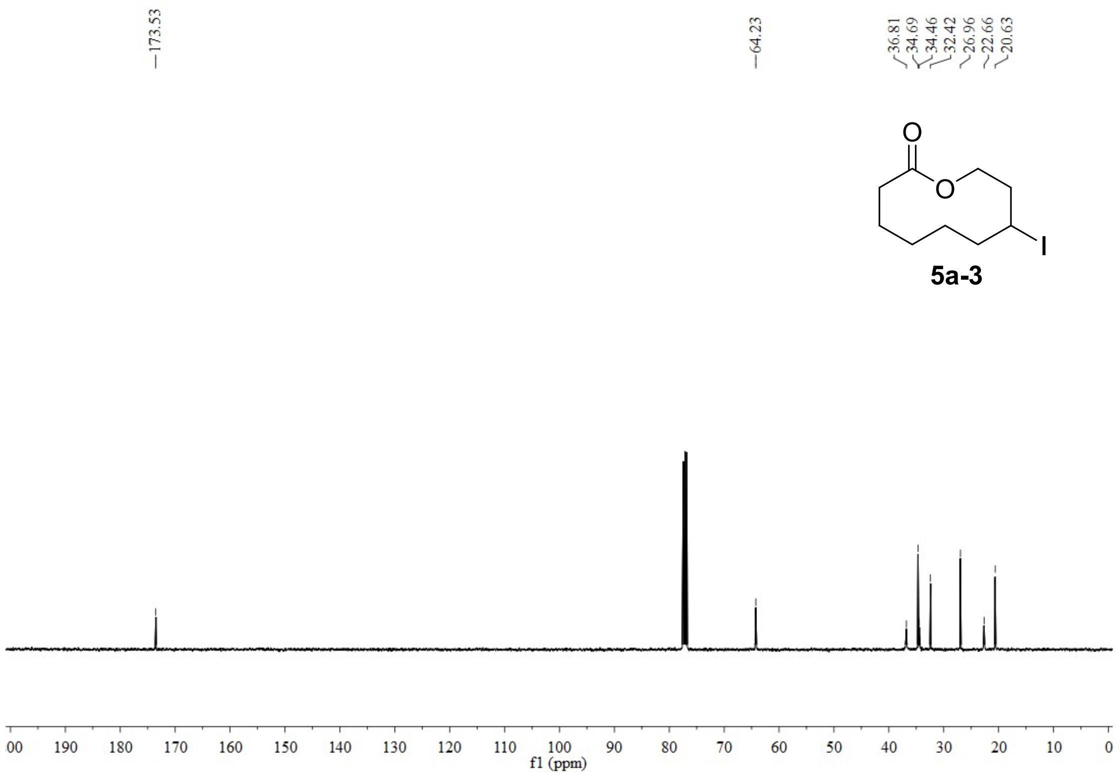

5a-3

^{1}H NMR (400 MHz, $CDCl_3$) and ^{13}C NMR (100 MHz, $CDCl_3$) spectra of product **5b**

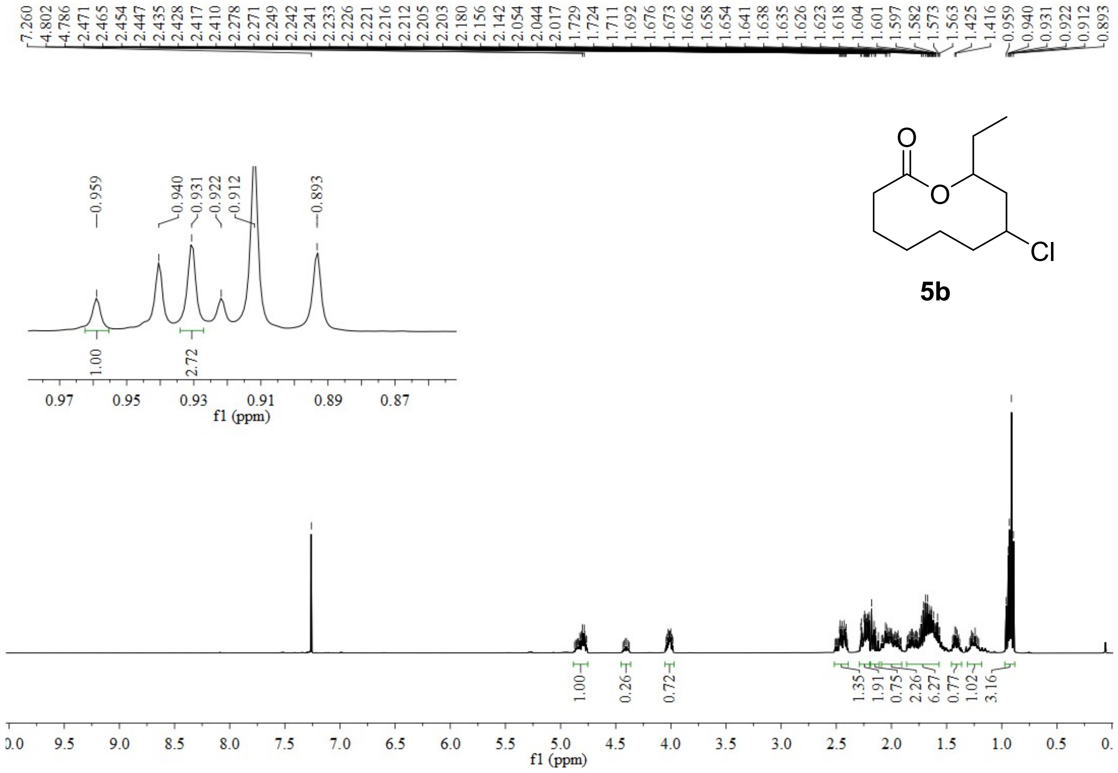

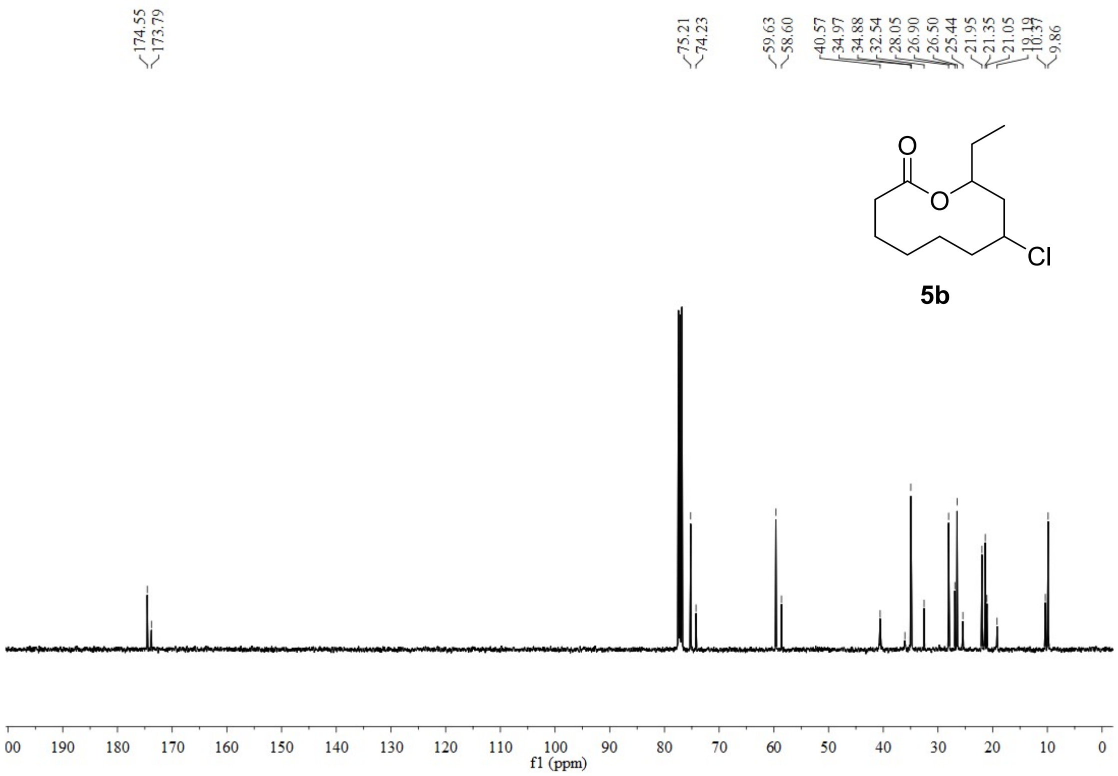

^{1}H NMR (400 MHz, $CDCl_3$) and ^{13}C NMR (100 MHz, $CDCl_3$) spectra of product **5c**

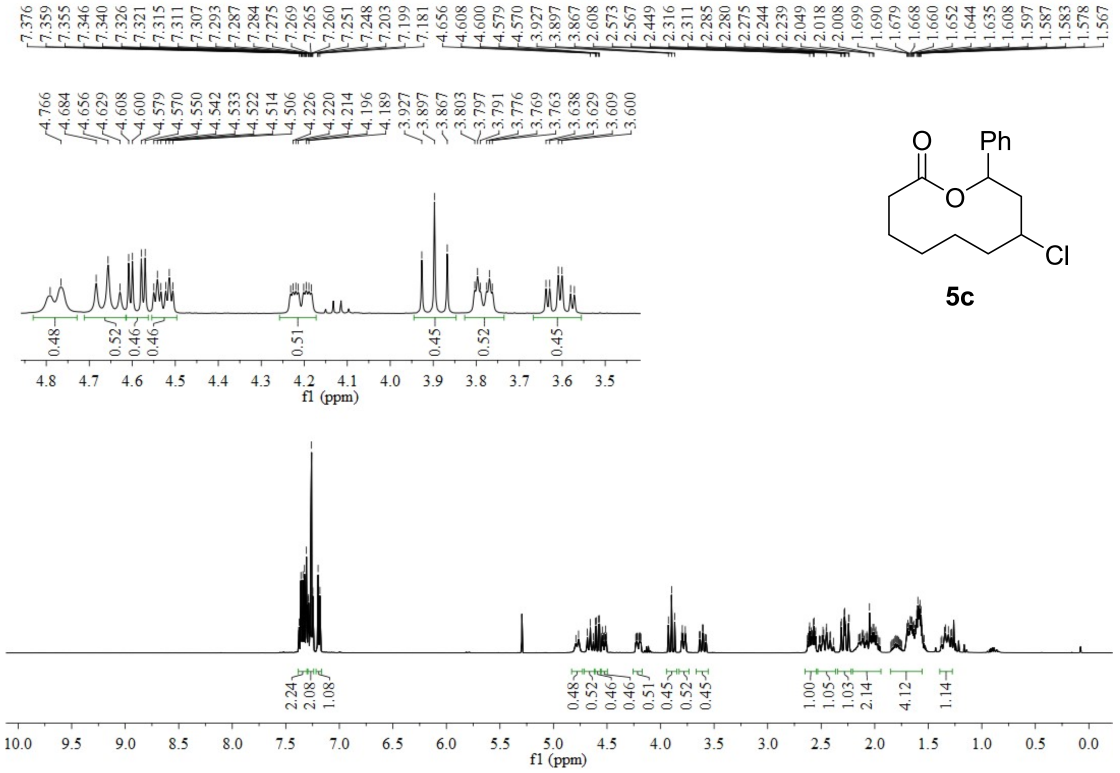

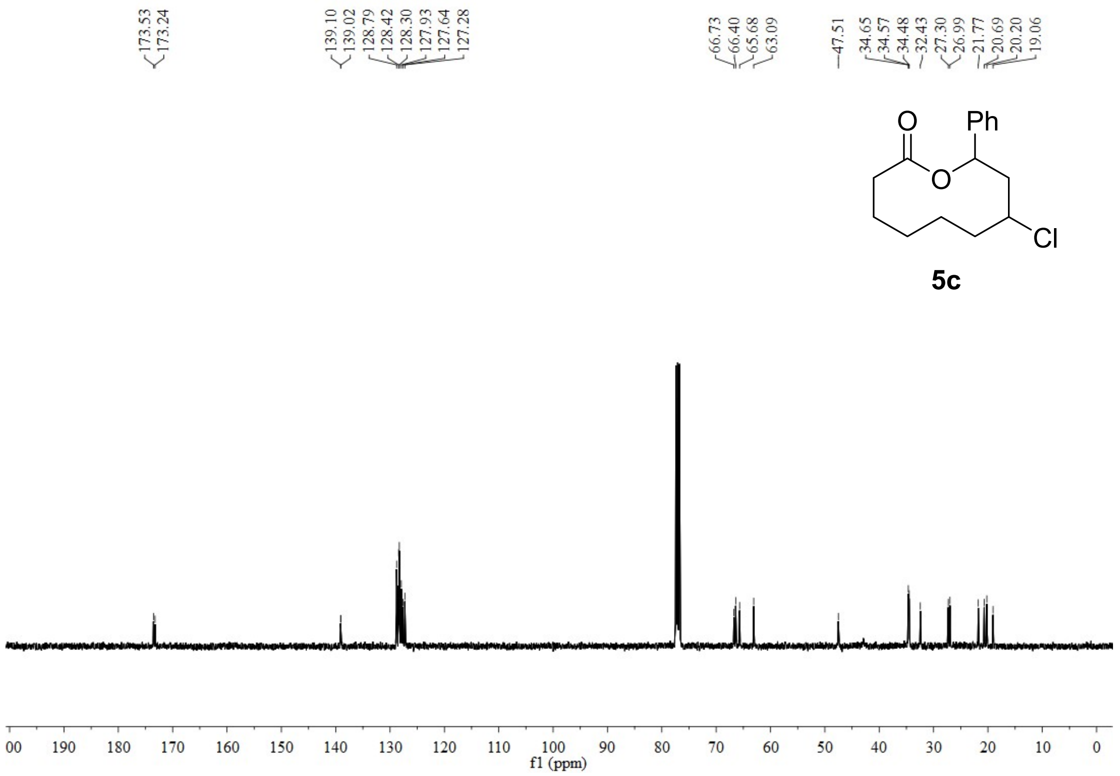

^{1}H NMR (400 MHz, $CDCl_3$) and ^{13}C NMR (100 MHz, $CDCl_3$) spectra of product **5d**

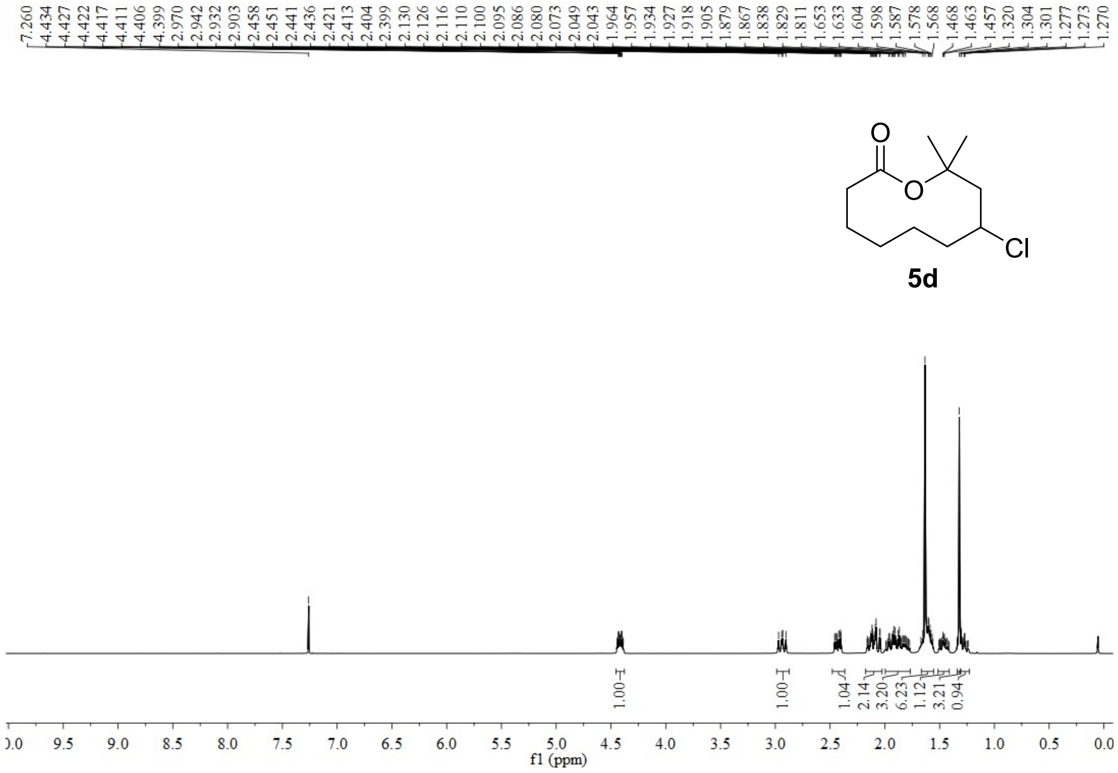

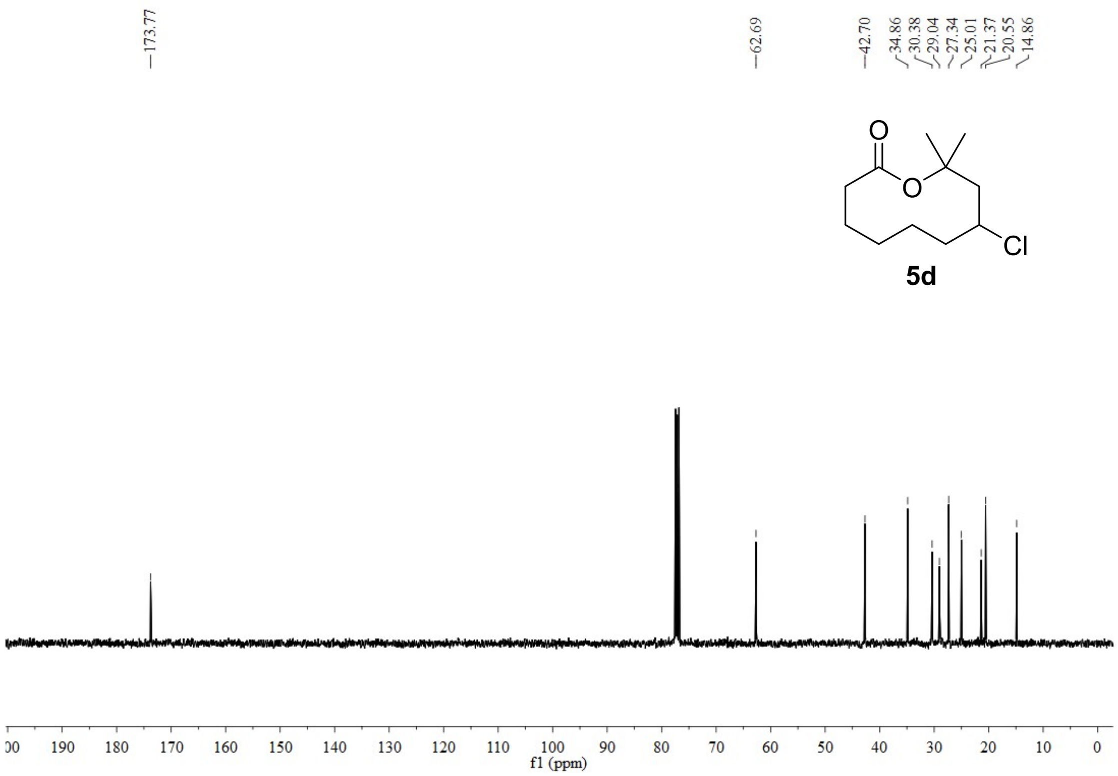

^{1}H NMR (400 MHz, $CDCl_3$) and ^{13}C NMR (100 MHz, $CDCl_3$) spectra of product **5e**

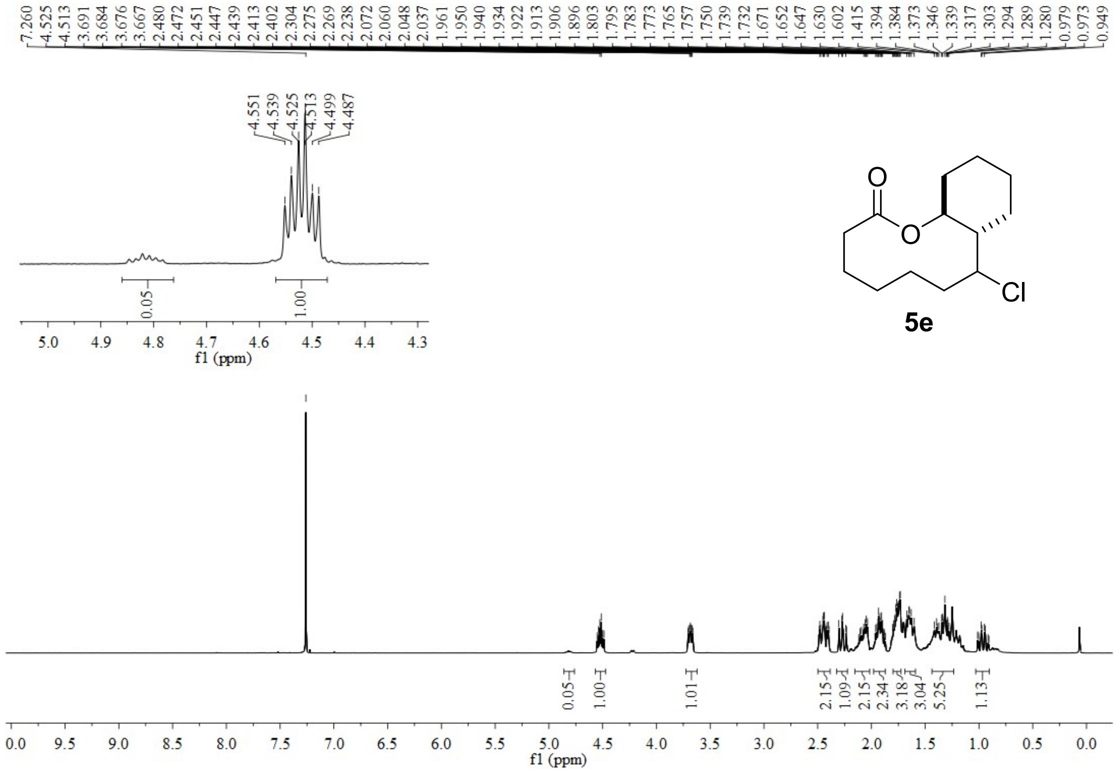

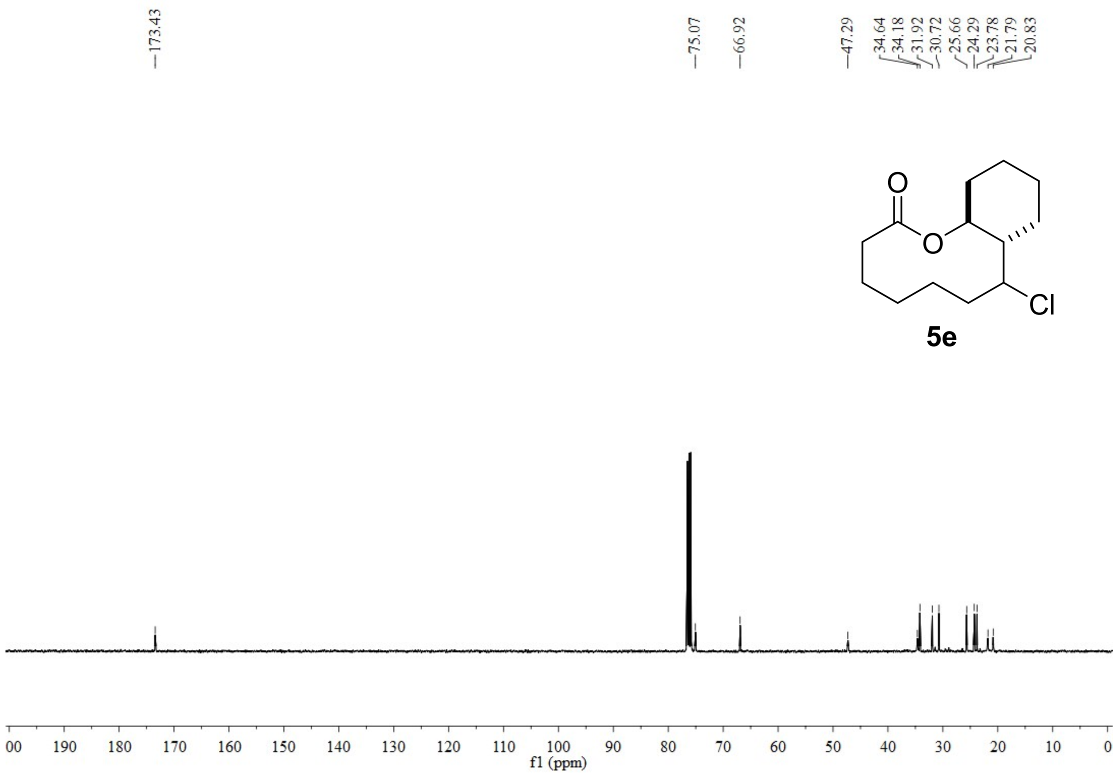

^{1}H NMR (400 MHz, $CDCl_3$) and ^{13}C NMR (100 MHz, $CDCl_3$) spectra of product **5f**

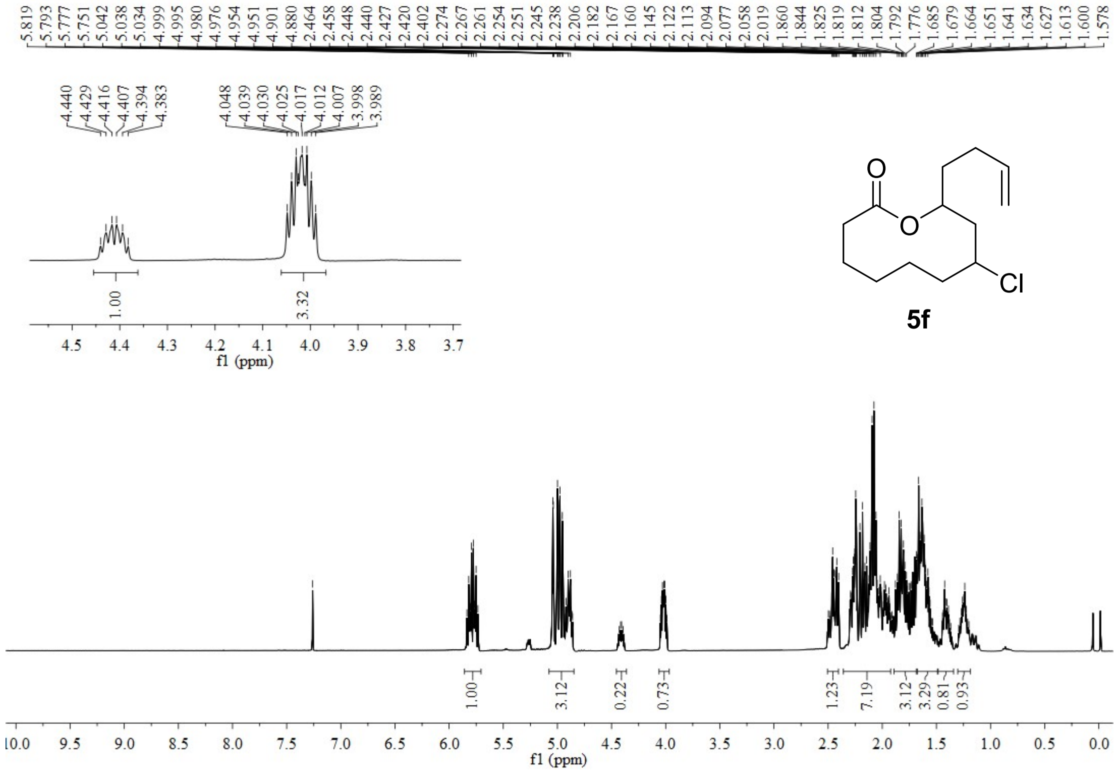

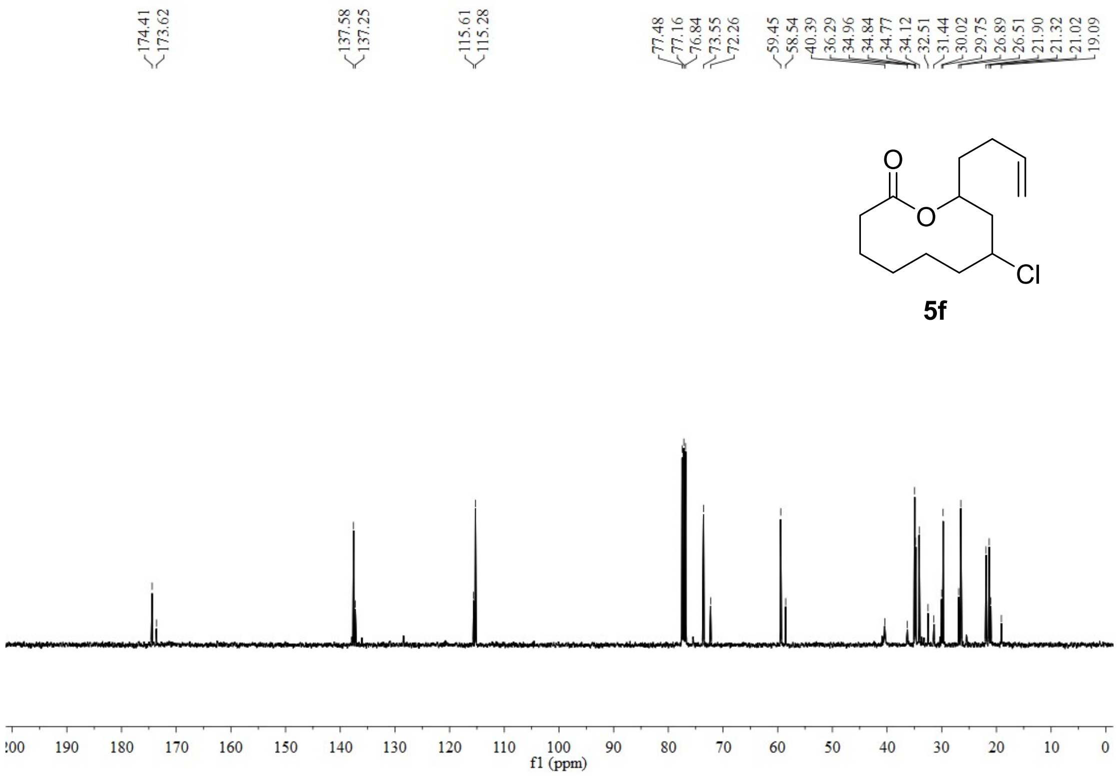

^{1}H NMR (400 MHz, $CDCl_3$) and ^{13}C NMR (100 MHz, $CDCl_3$) spectra of product **5g**

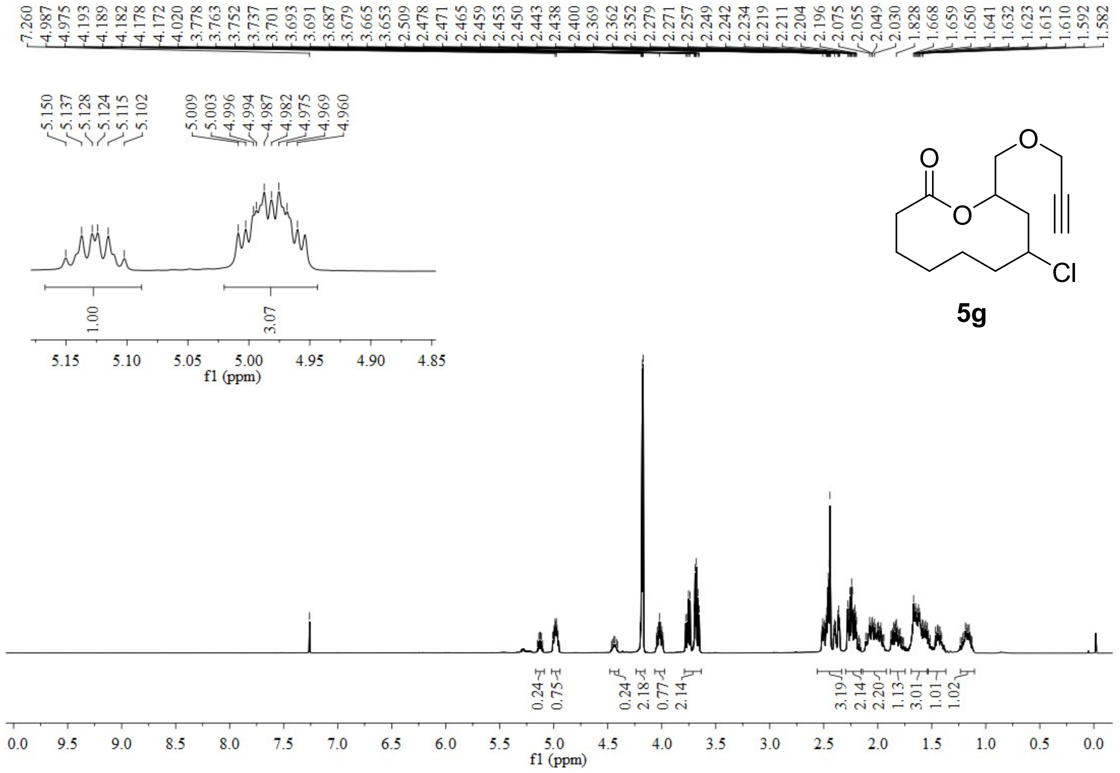

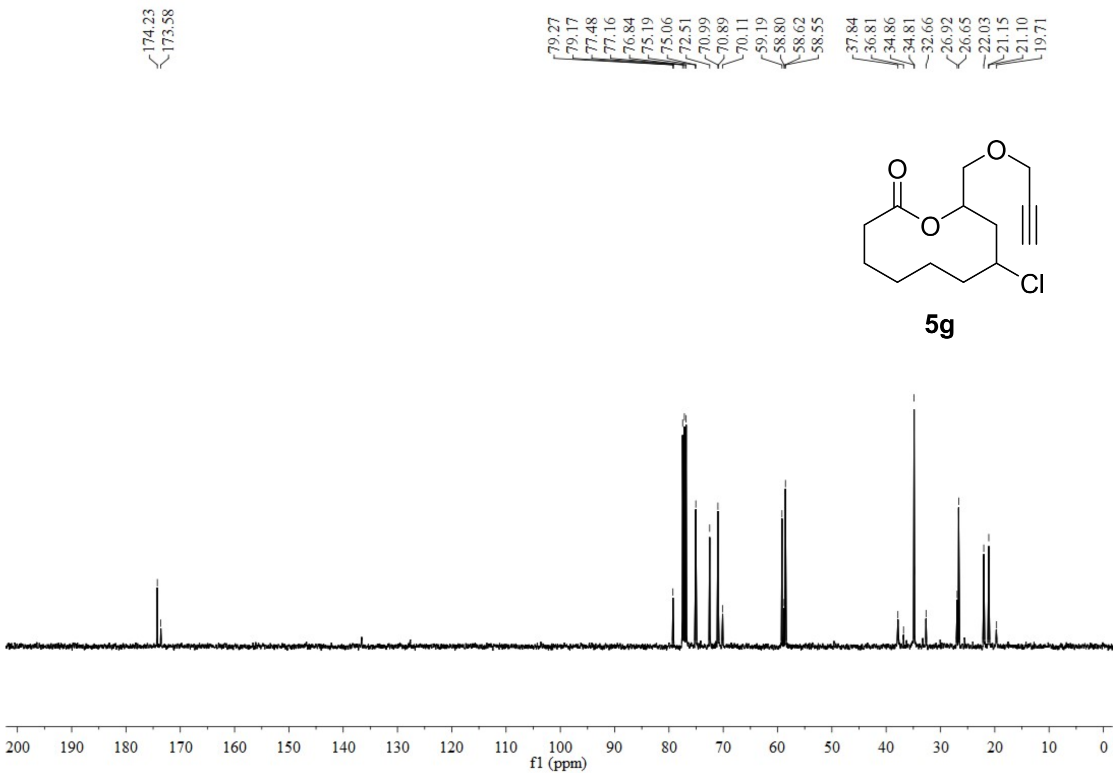

^{1}H NMR (400 MHz, $CDCl_3$) and ^{13}C NMR (100 MHz, $CDCl_3$) spectra of product **5h**

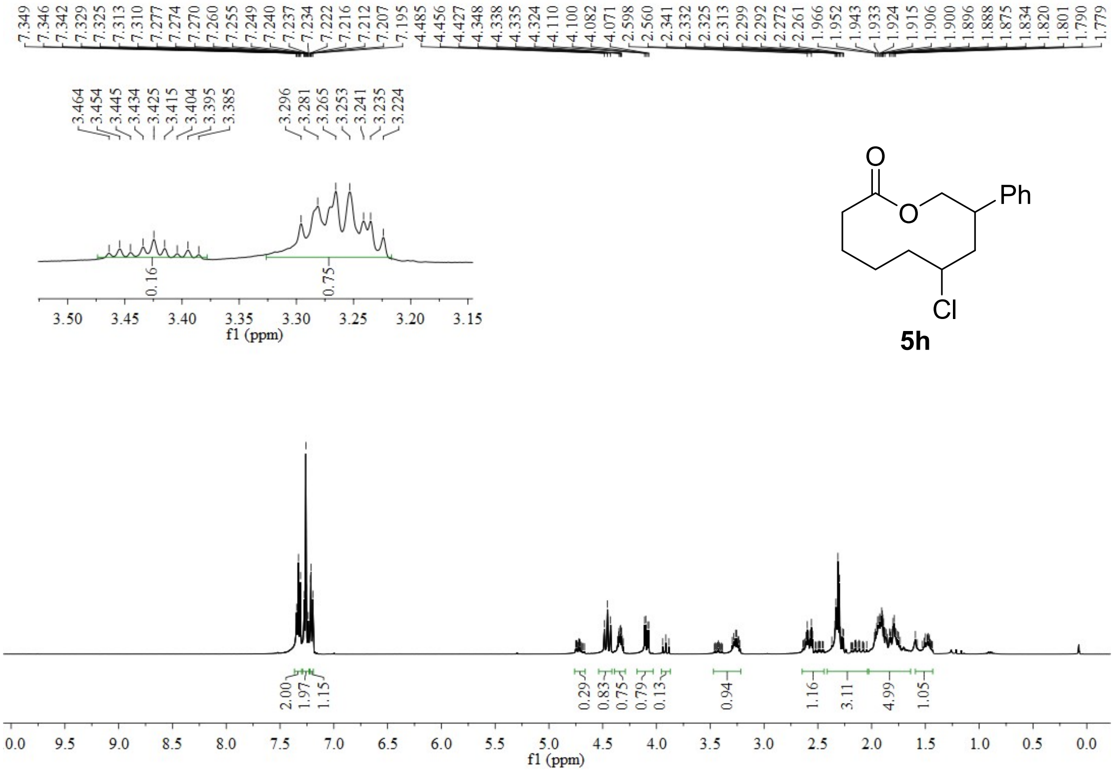

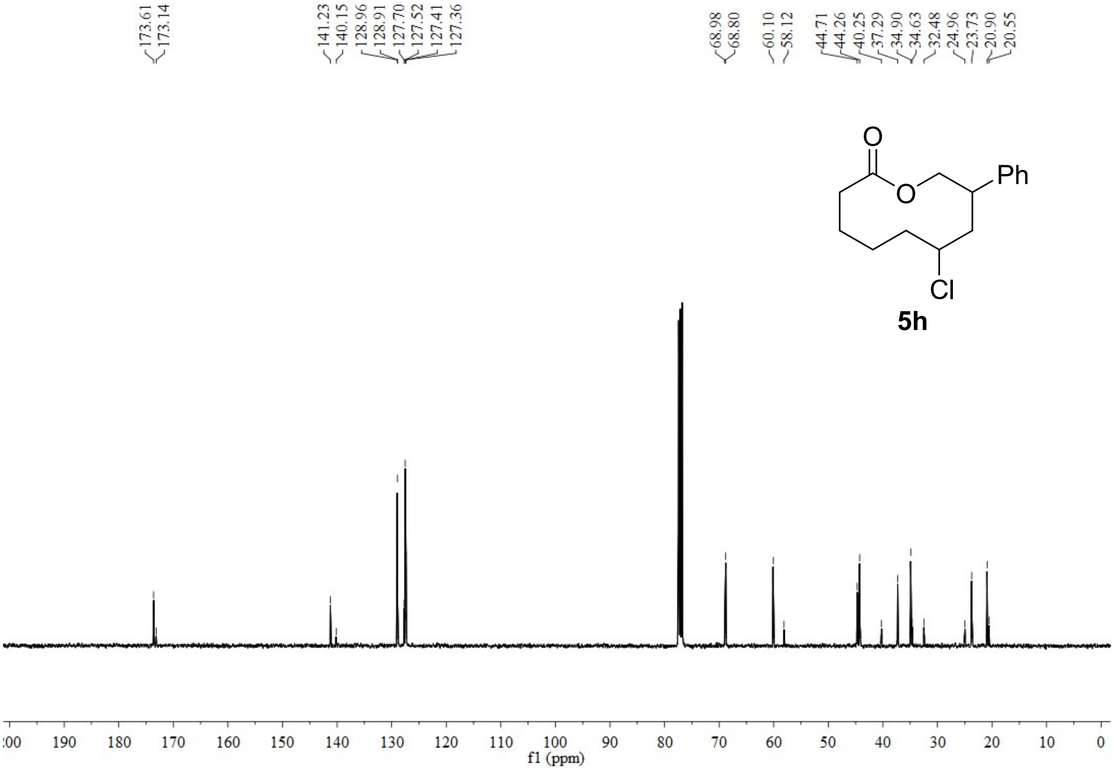

^{1}H NMR (400 MHz, $CDCl_3$) and ^{13}C NMR (100 MHz, $CDCl_3$) spectra of product **5i**

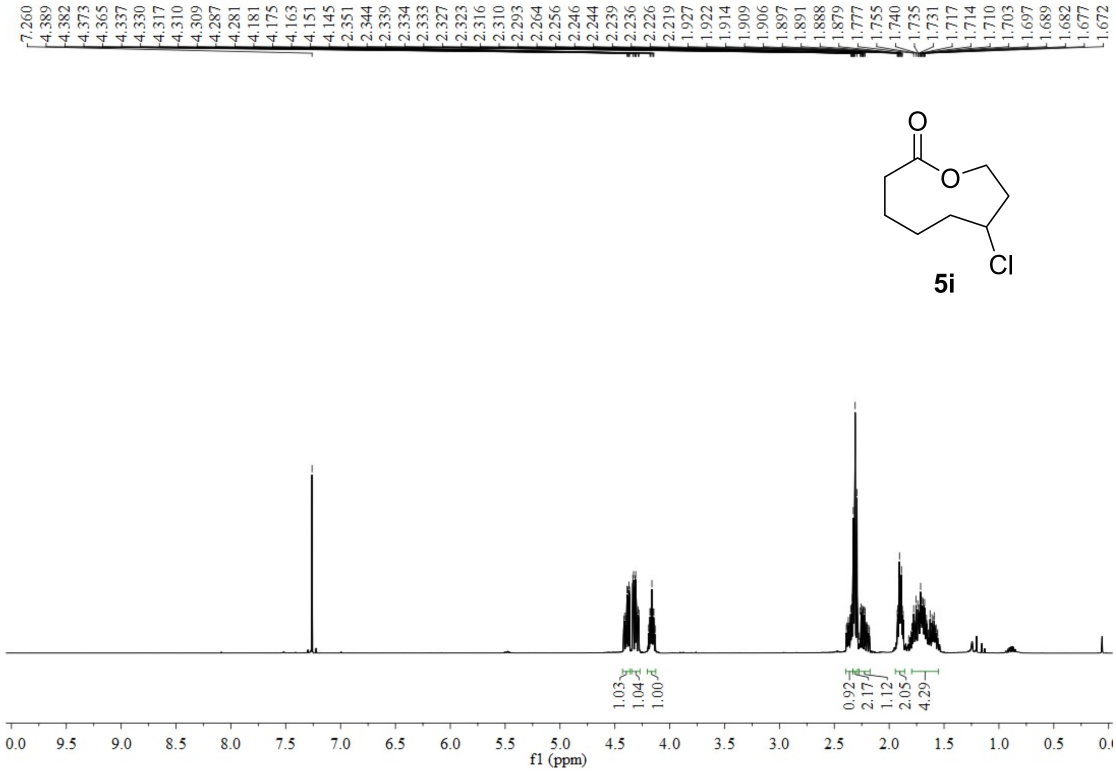

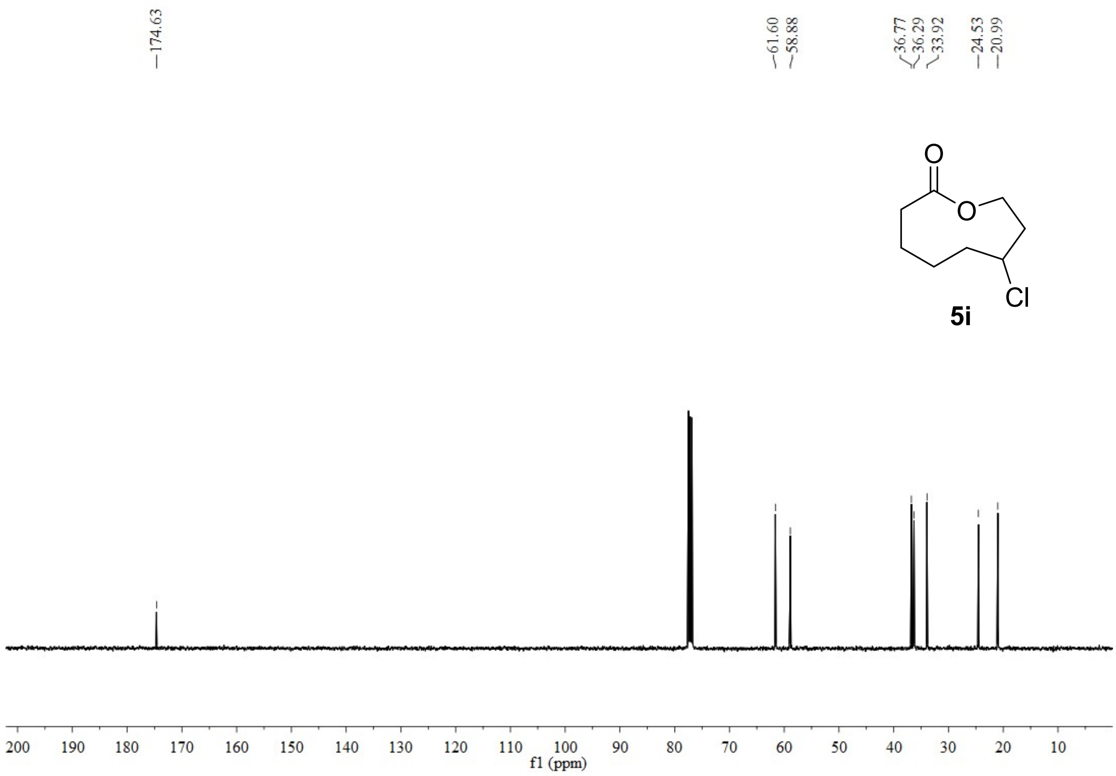

^1H NMR (400 MHz, $CDCl_3$) and ^{13}C NMR (100 MHz, $CDCl_3$) spectra of product **5j**

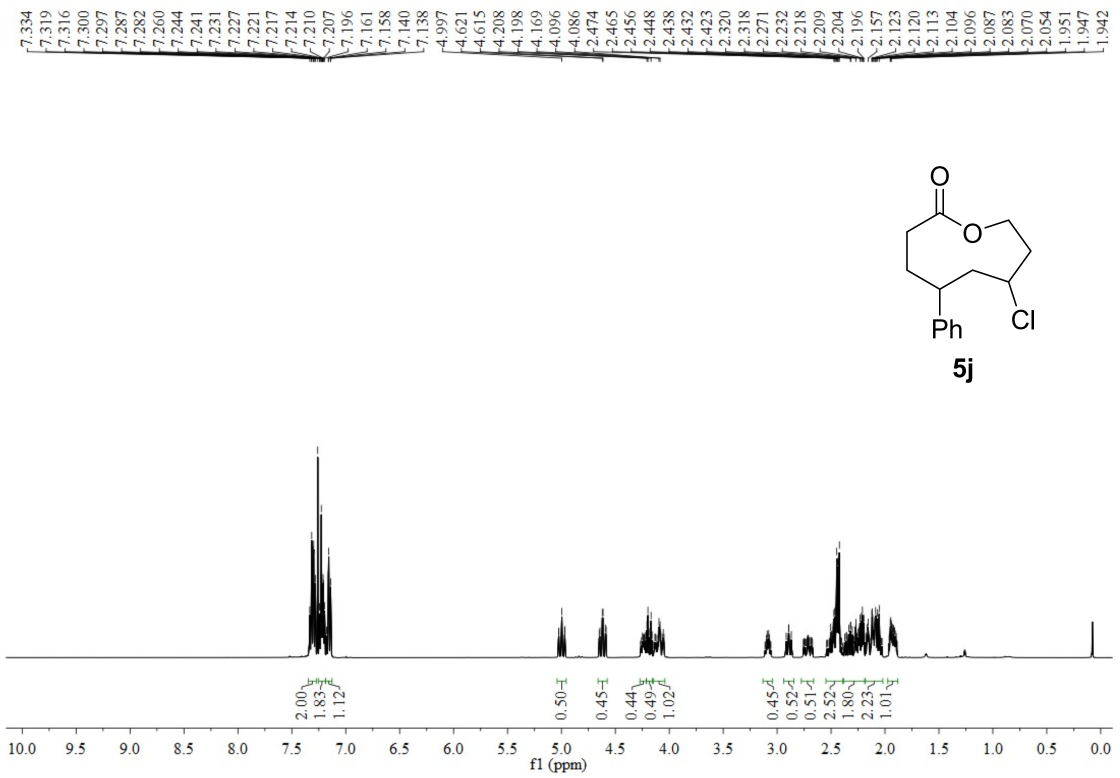

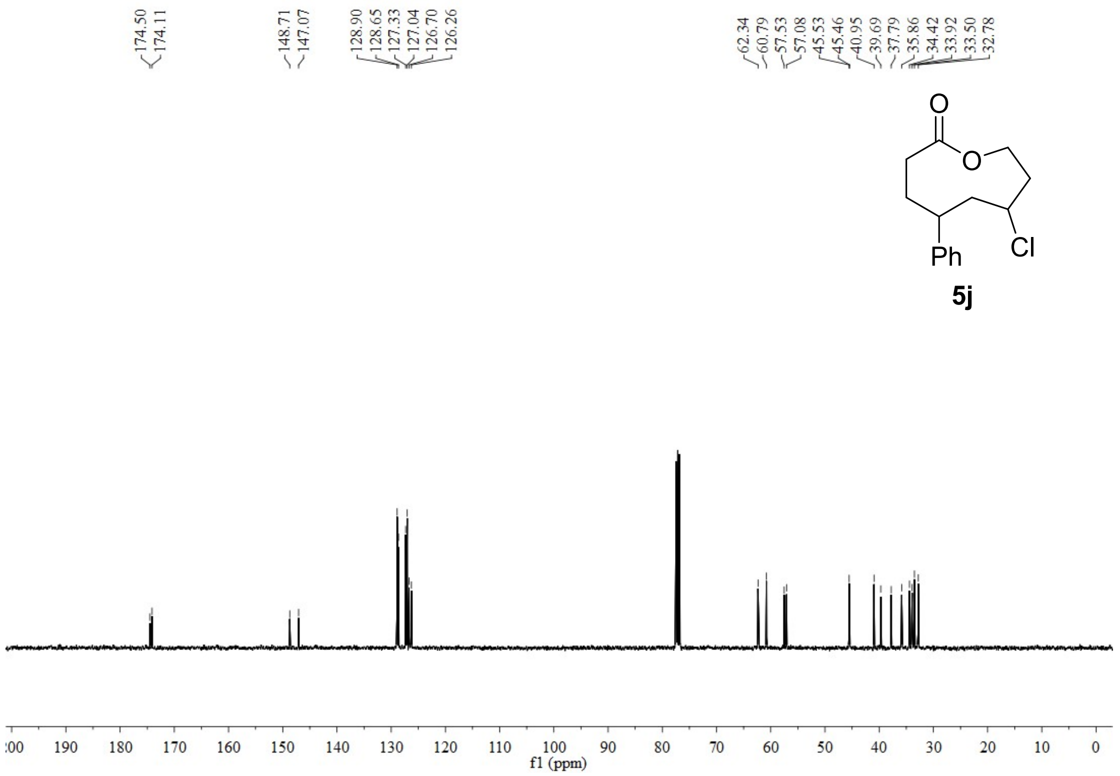

^{1}H NMR (400 MHz, $CDCl_3$) and ^{13}C NMR (100 MHz, $CDCl_3$) spectra of product **5k**

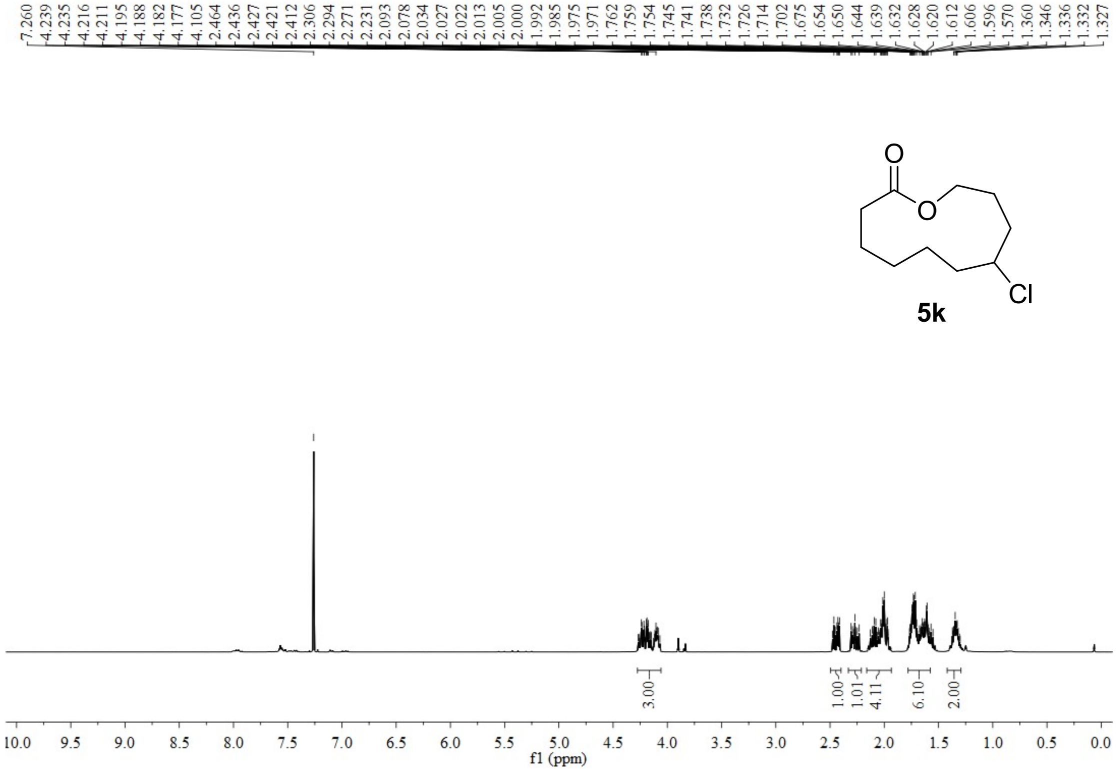

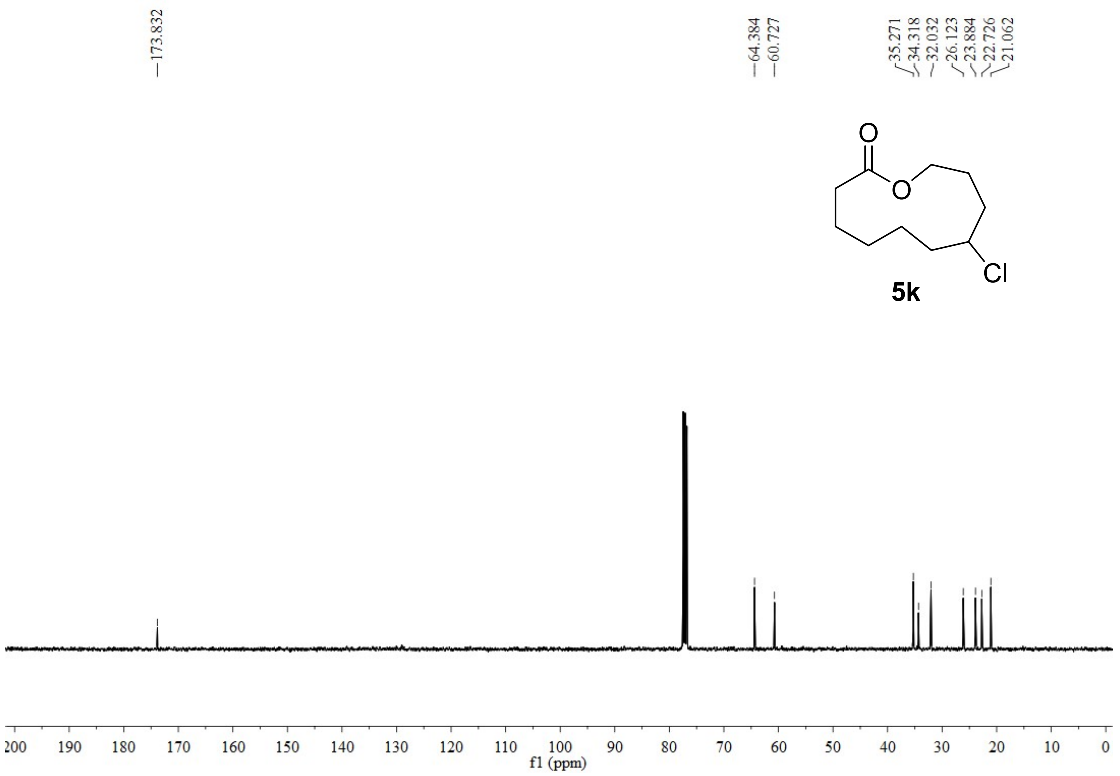

^{1}H NMR (400 MHz, $CDCl_3$) and ^{13}C NMR (100 MHz, $CDCl_3$) spectra of product **5l**

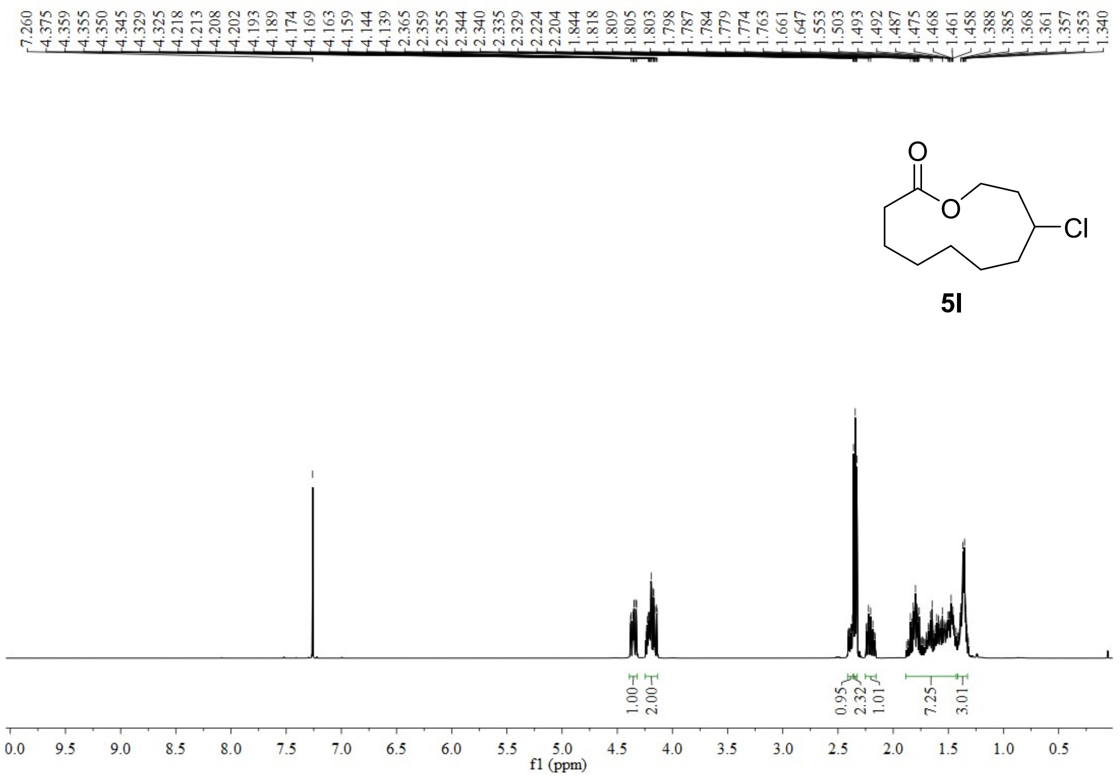

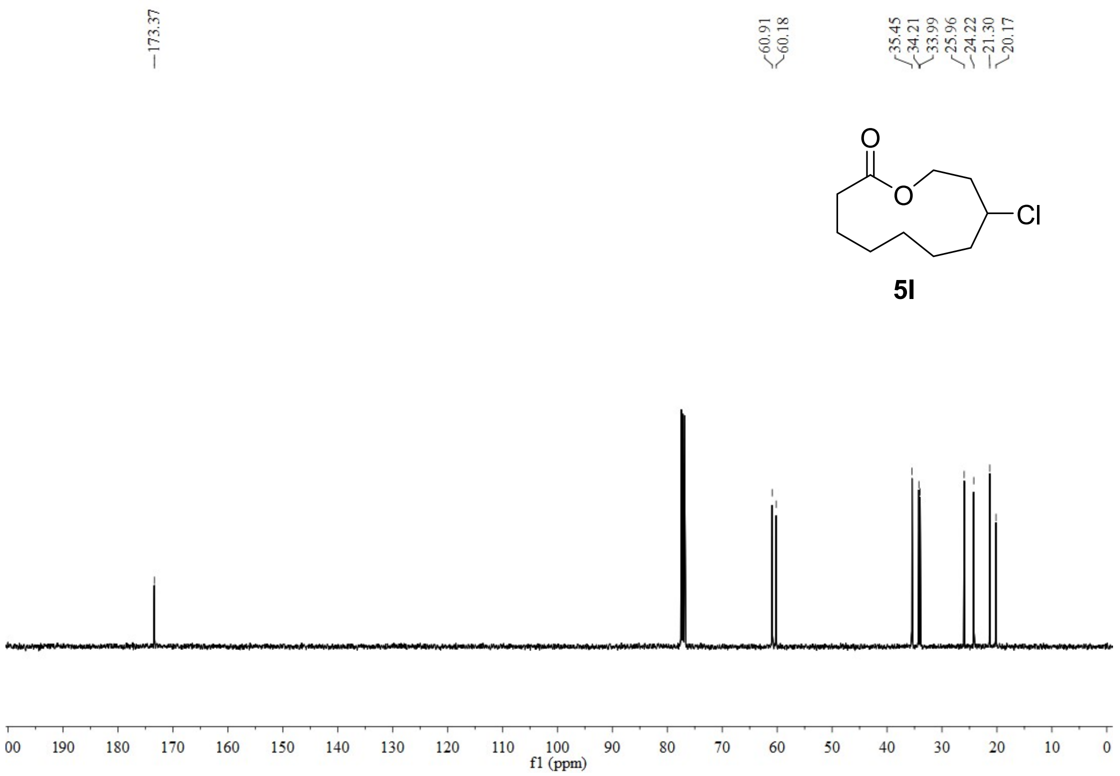

^{1}H NMR (400 MHz, $CDCl_3$) and ^{13}C NMR (100 MHz, $CDCl_3$) spectra of product **5m**

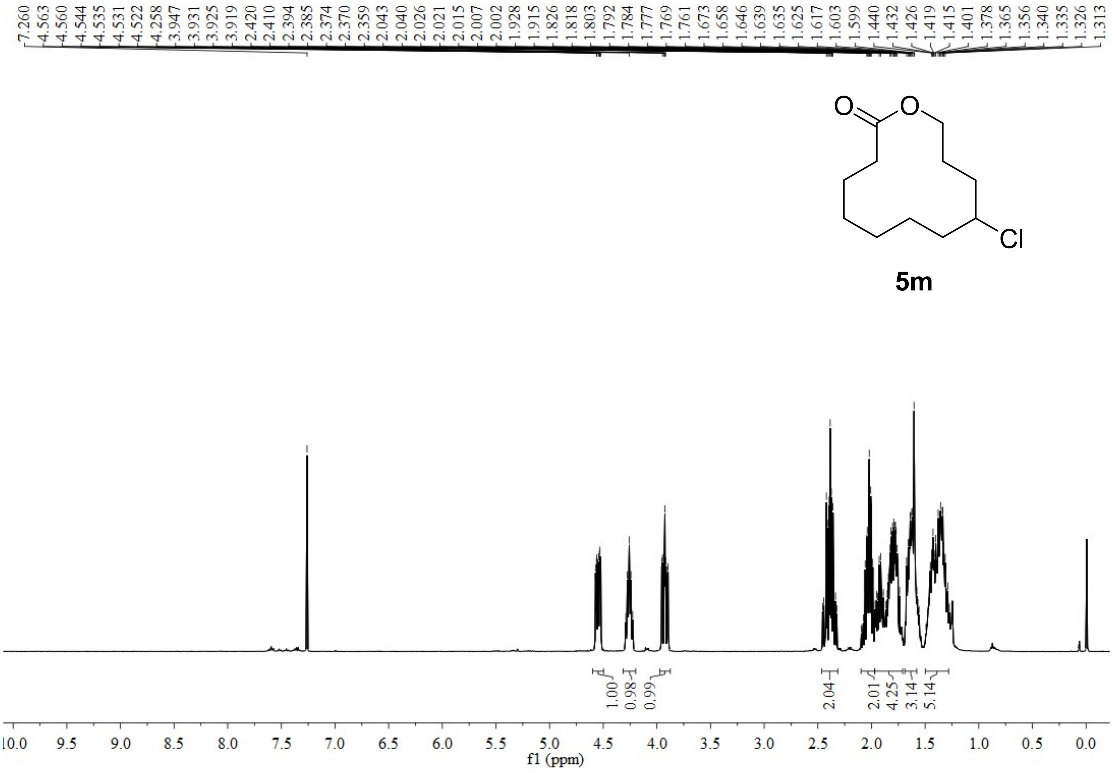

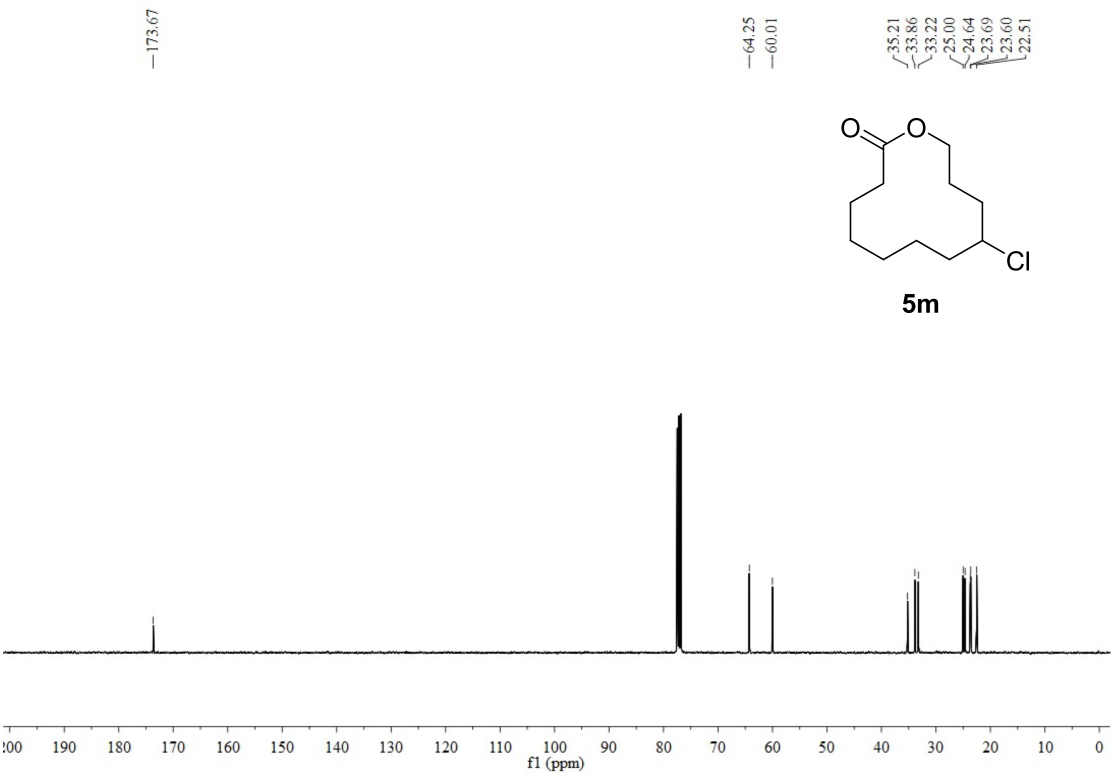

^{1}H NMR (400 MHz, $CDCl_3$) and ^{13}C NMR (100 MHz, $CDCl_3$) spectra of product **5n**

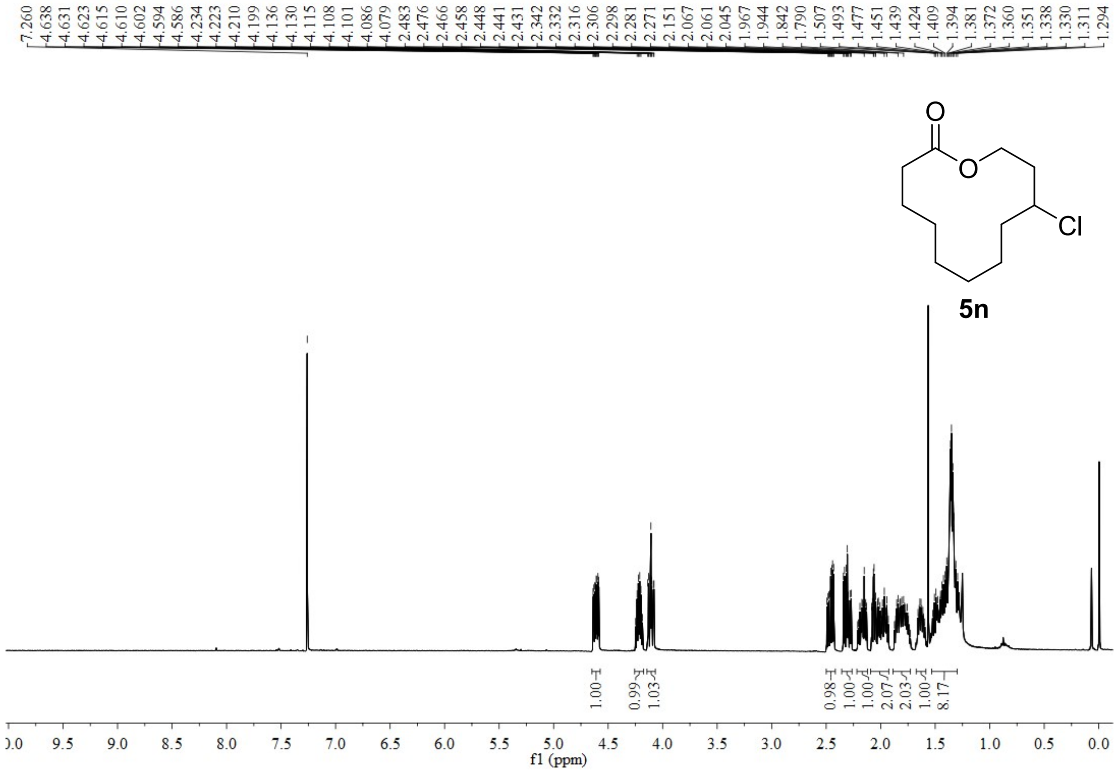

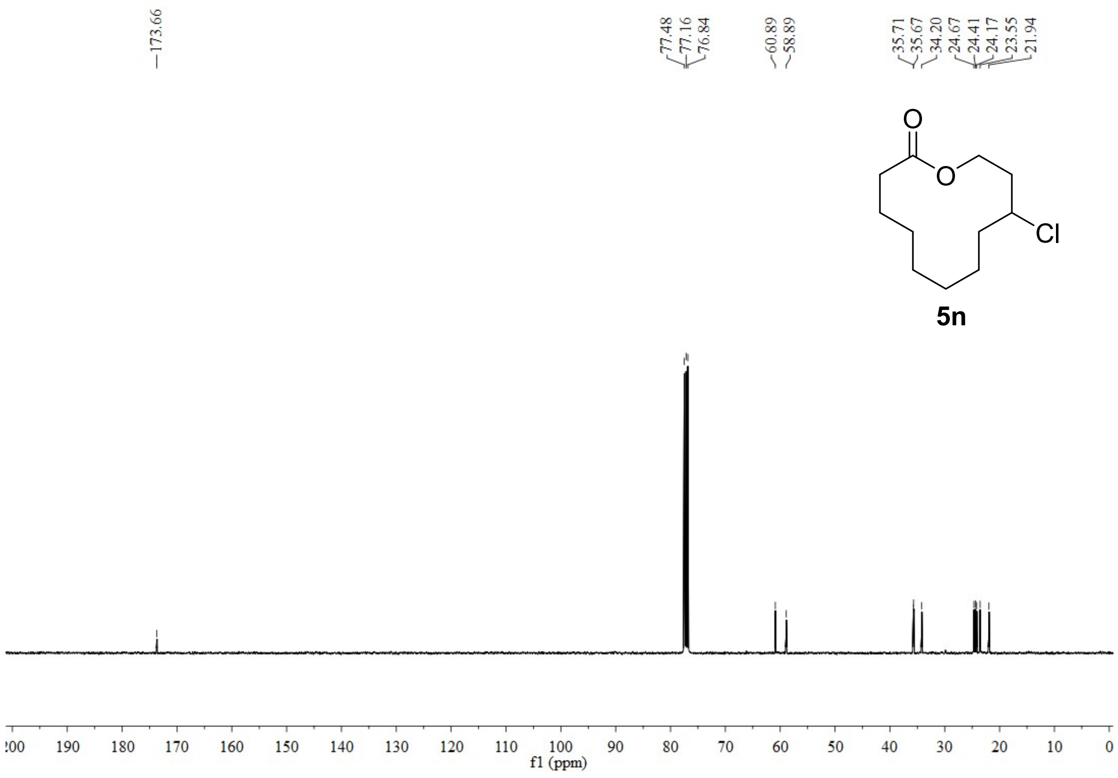

^{1}H NMR (400 MHz, $CDCl_3$) and ^{13}C NMR (100 MHz, $CDCl_3$) spectra of product **5o**

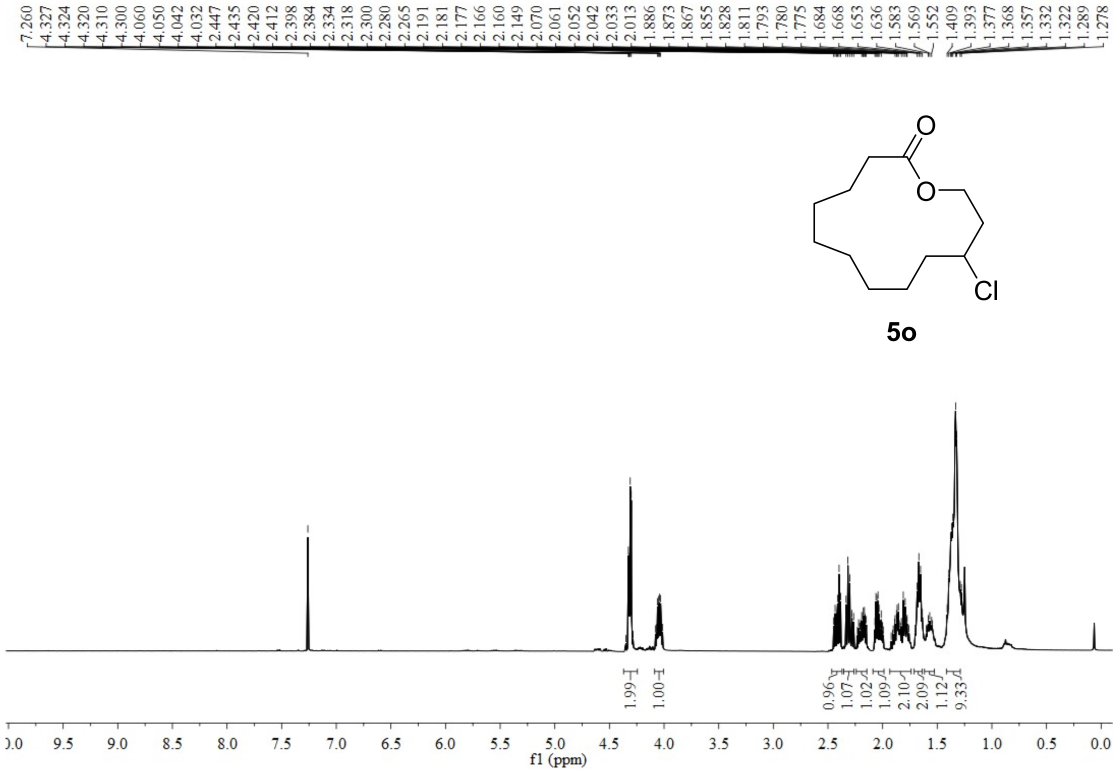

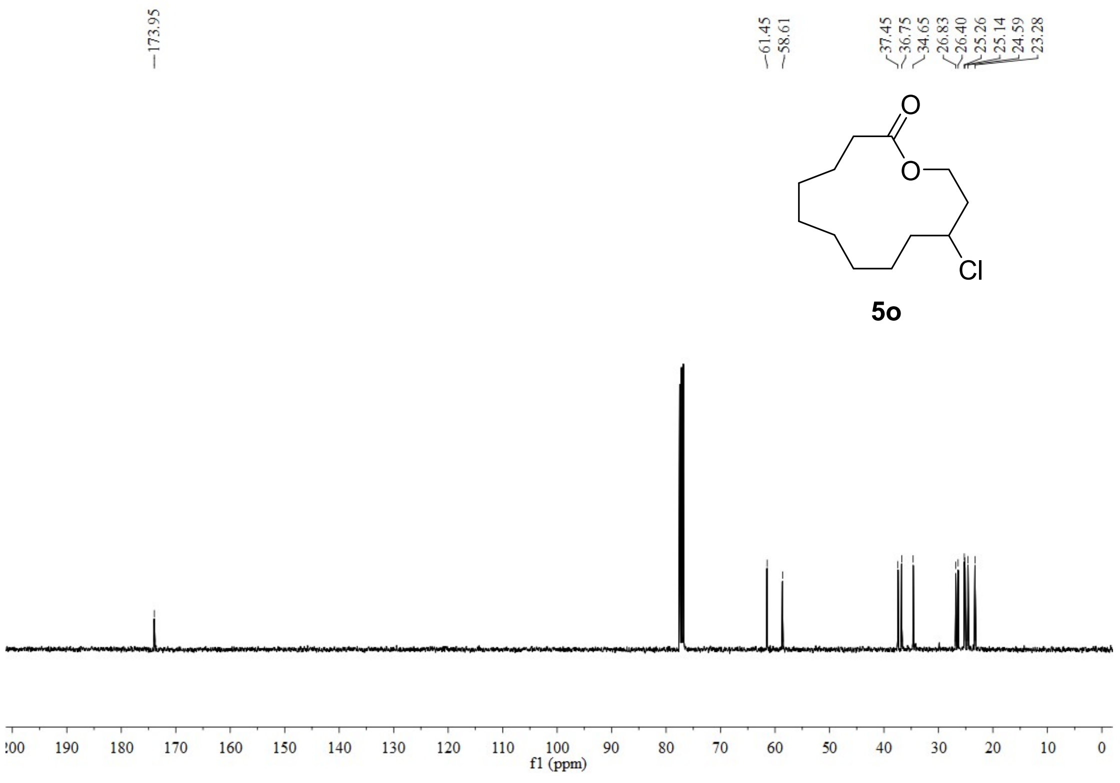

^{1}H NMR (400 MHz, $CDCl_3$) and ^{13}C NMR (100 MHz, $CDCl_3$) spectra of product **5p**

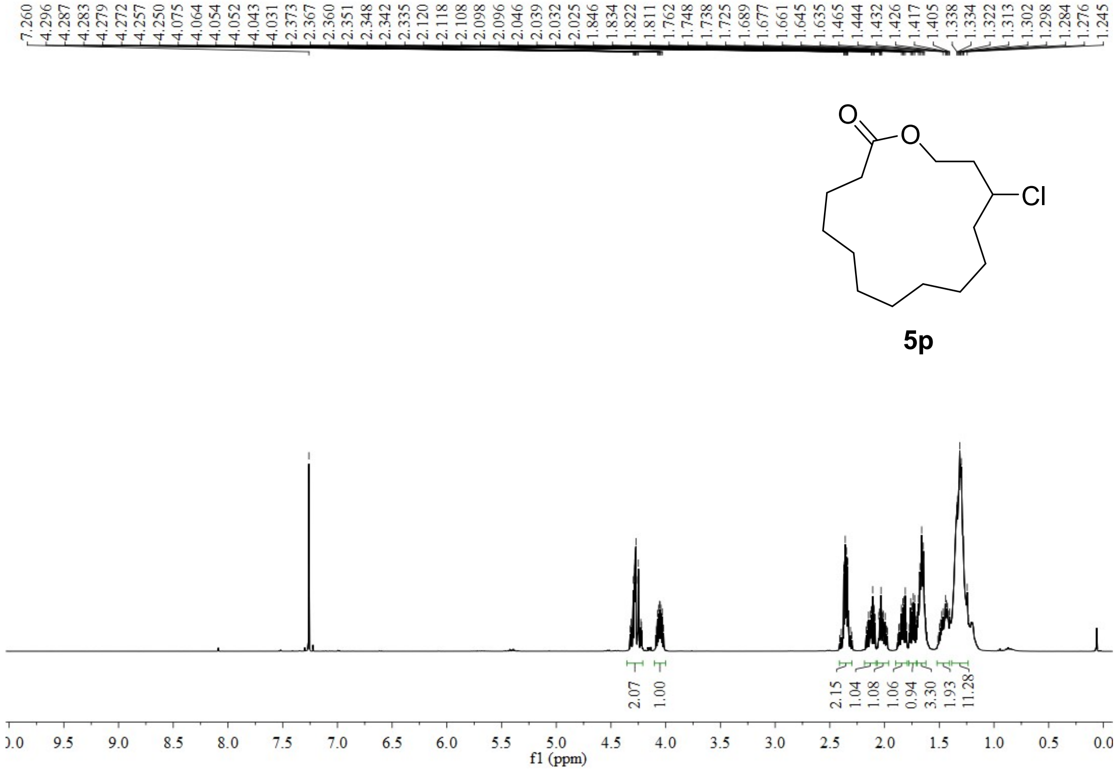

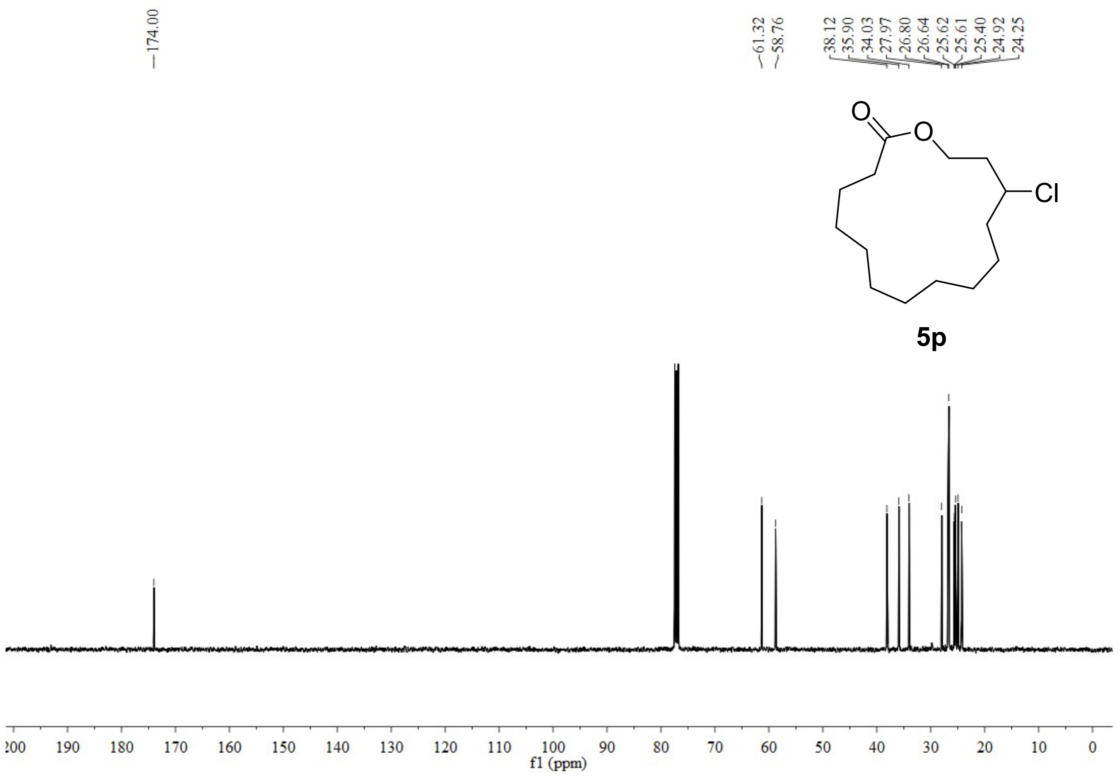

^{1}H NMR (400 MHz, $CDCl_3$) and ^{13}C NMR (100 MHz, $CDCl_3$) spectra of product **5q**

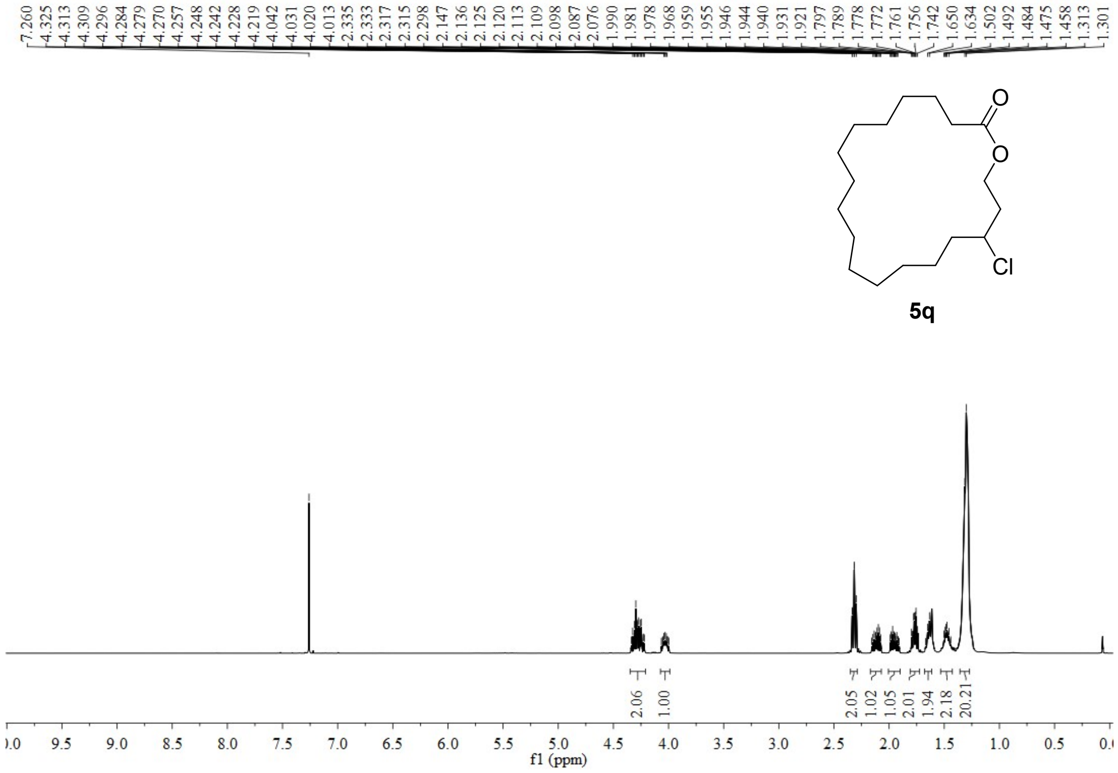
7.260
4.325
4.313
4.309
4.296
4.284
4.279
4.270
4.257
4.248
4.242
4.228
4.219
4.042
4.031
4.020
4.013
2.335
2.333
2.317
2.315
2.298
2.147
2.136
2.125
2.120
2.113
2.109
2.098
2.087
2.076
1.990
1.981
1.978
1.968
1.959
1.955
1.946
1.944
1.940
1.931
1.921
1.797
1.789
1.778
1.772
1.761
1.756
1.742
1.650
1.634
1.502
1.492
1.484
1.475
1.458
1.313
1.301
O
O
Cl
5q
2.06
1.00
2.05
1.02
1.05
2.01
1.94
2.18
20.21
f1 (ppm)

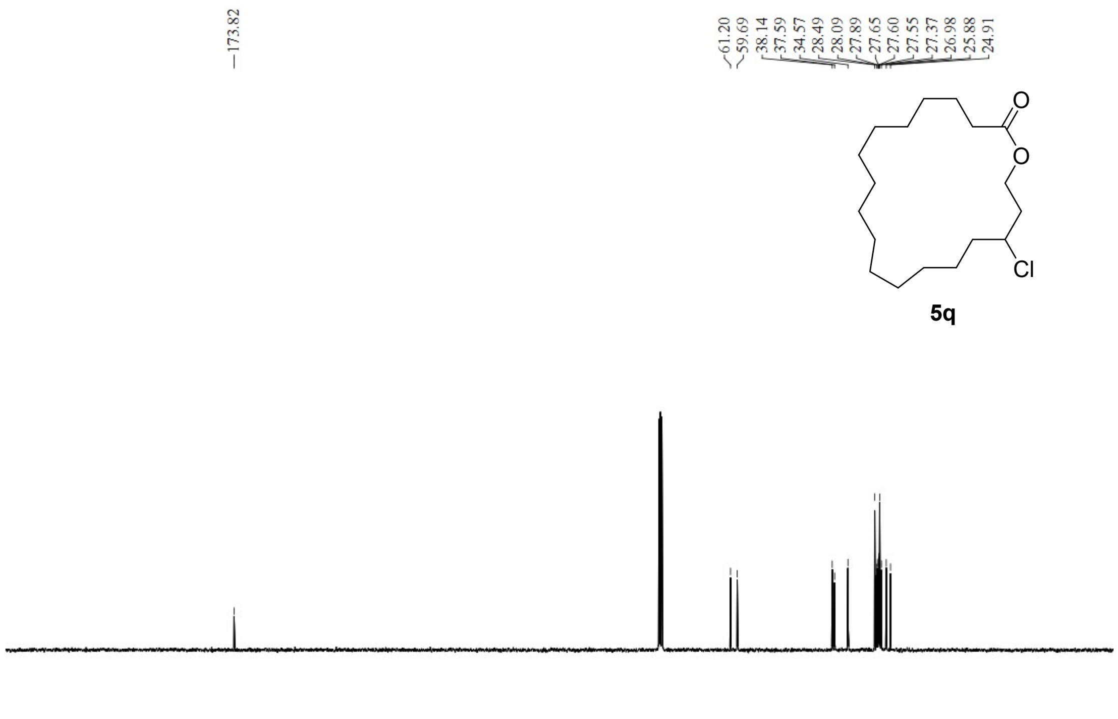
173.82
61.20
59.69
38.14
37.59
34.57
28.49
28.09
27.89
27.65
27.60
27.55
27.37
26.98
25.88
24.91
O
O
Cl
5q
f1 (ppm)

16. ^{1}H NMR and ^{13}C NMR Spectra of Products 6-11

^{1}H NMR (400 MHz, $CDCl_3$) and ^{13}C NMR (100 MHz, $CDCl_3$) spectra of product **6**

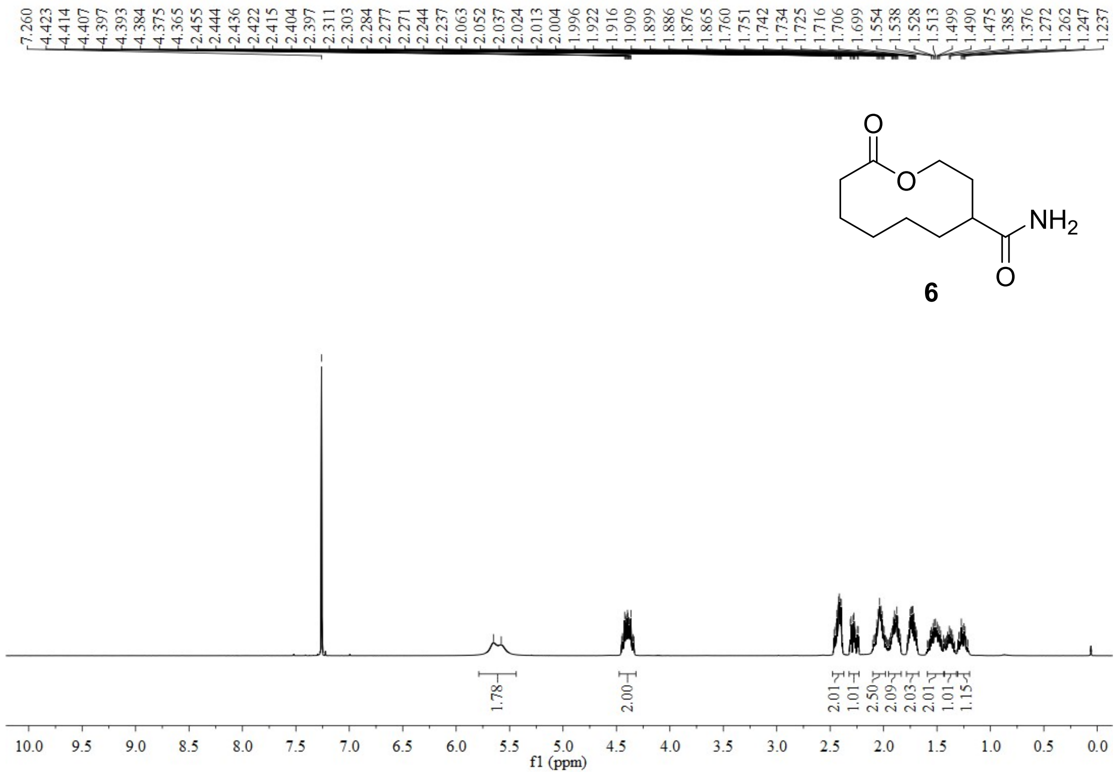

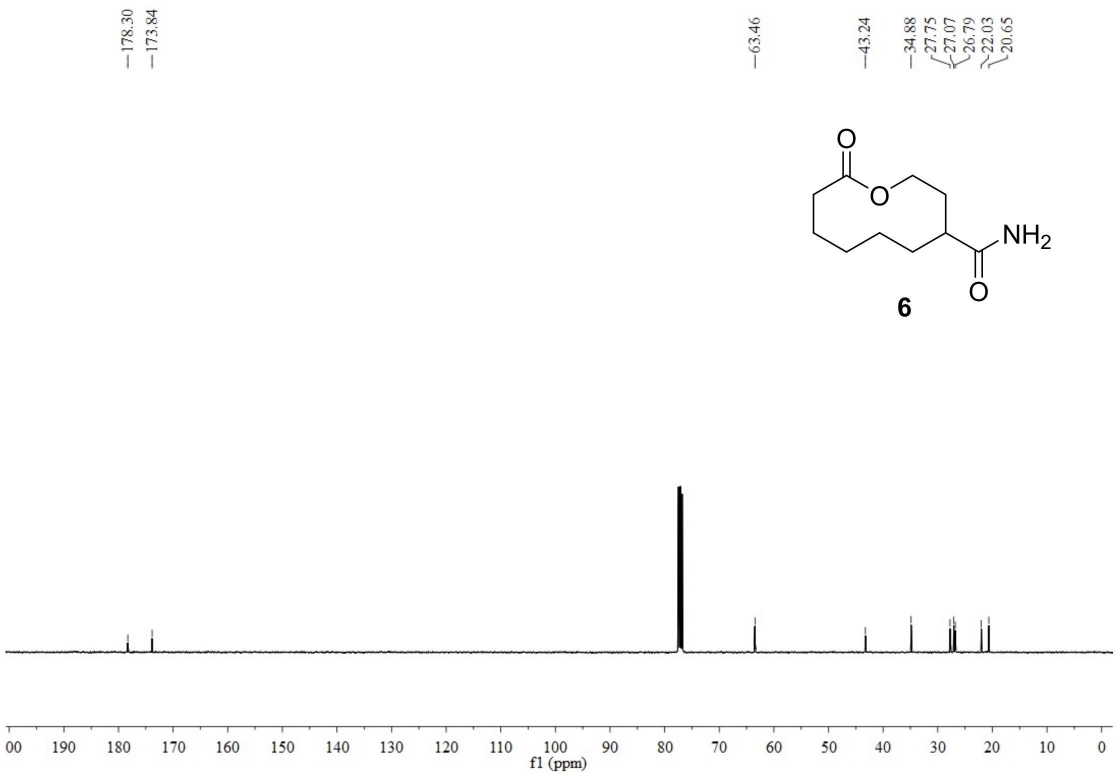

^{1}H NMR (400 MHz, $CDCl_3$) and ^{13}C NMR (100 MHz, $CDCl_3$) spectra of product **7**

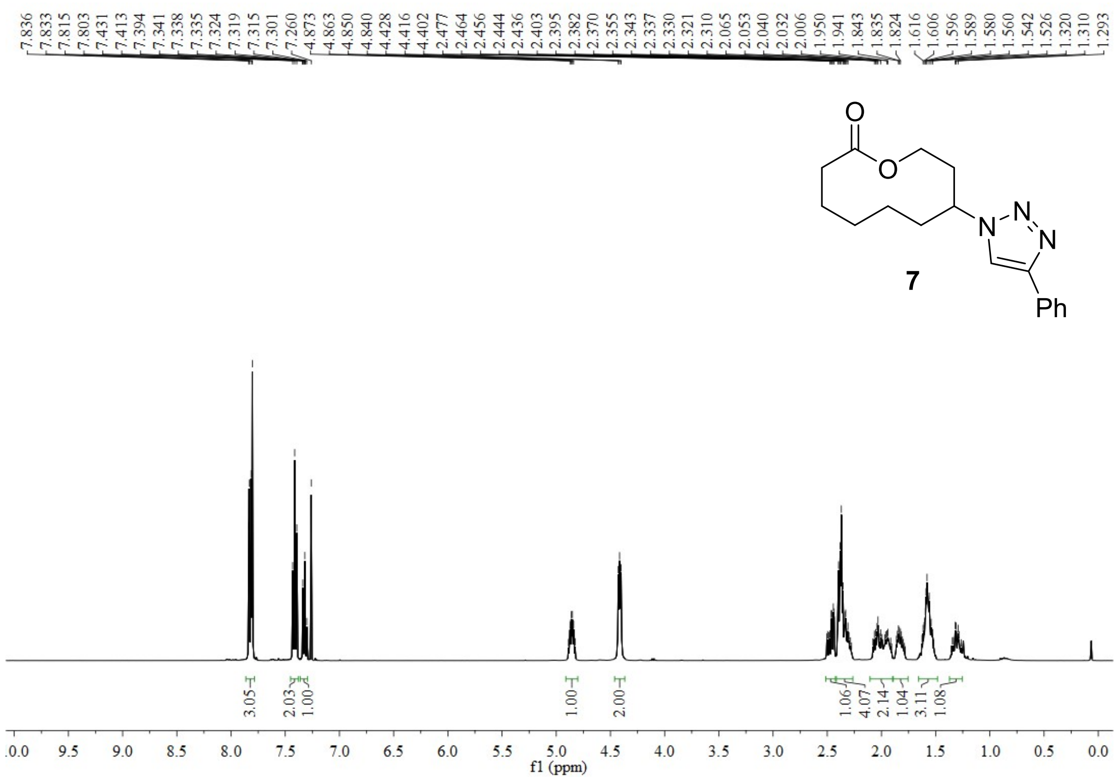

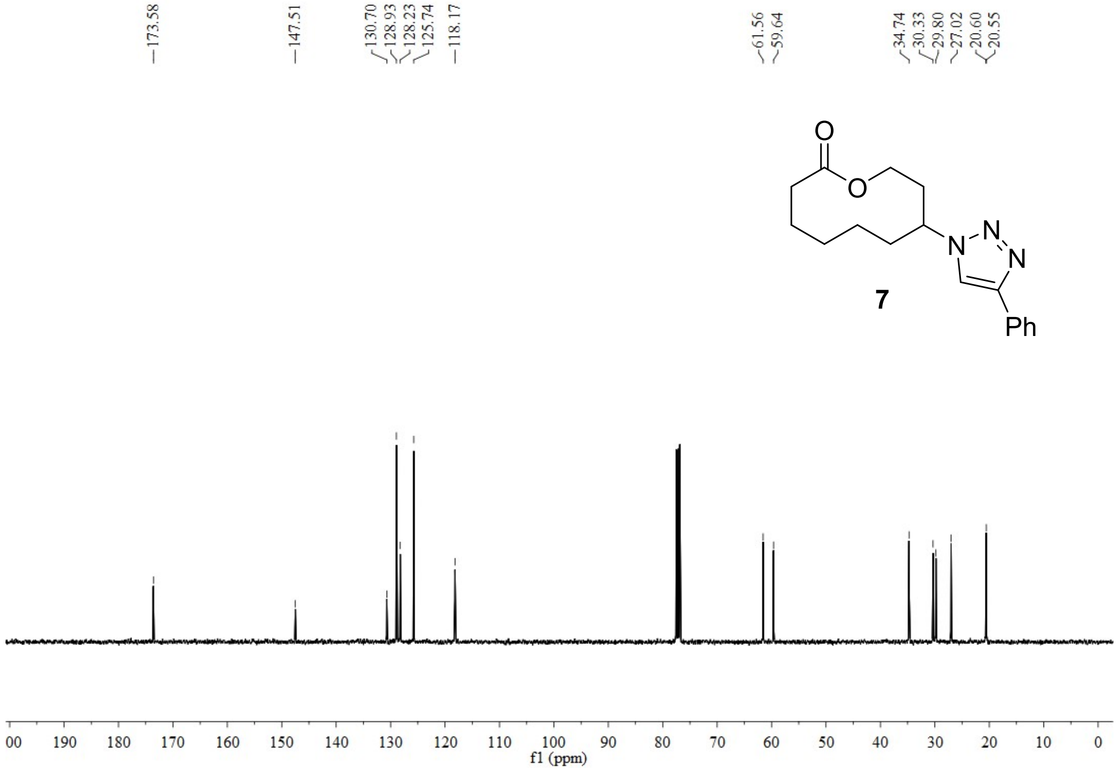

^{1}H NMR (400 MHz, $CDCl_3$) and ^{13}C NMR (100 MHz, $CDCl_3$) spectra of product **8**

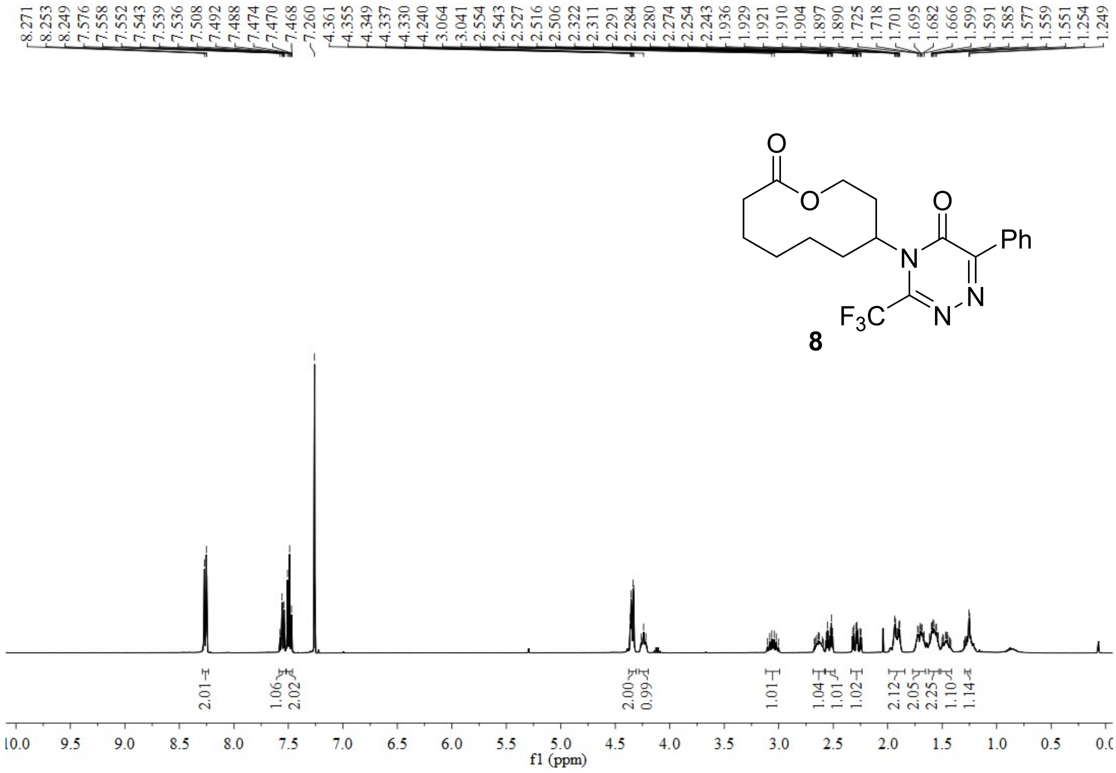

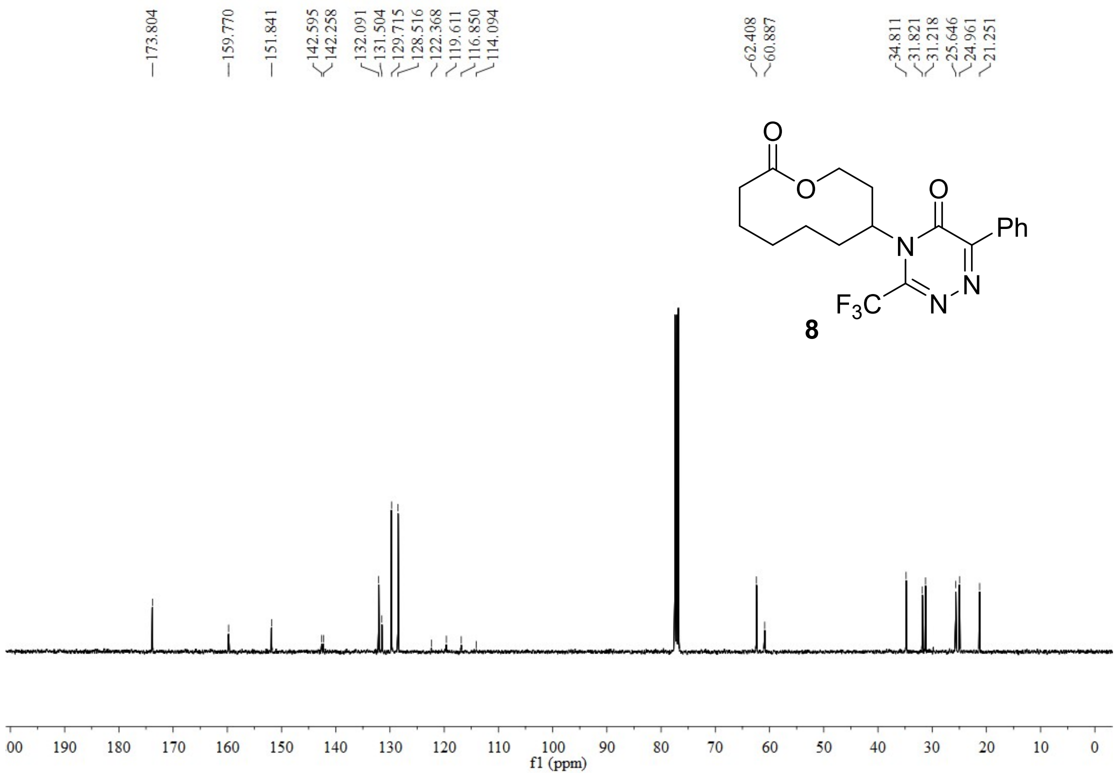

^{19}F NMR (376 MHz, $CDCl_3$) spectra of product **8**

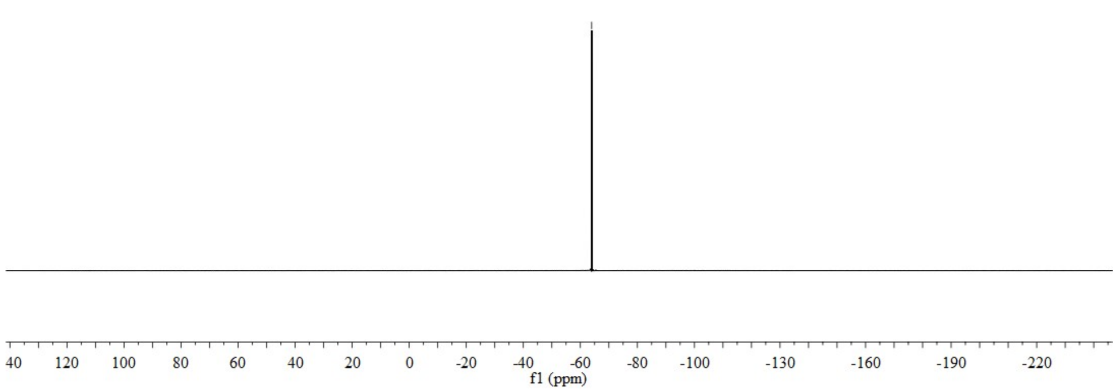

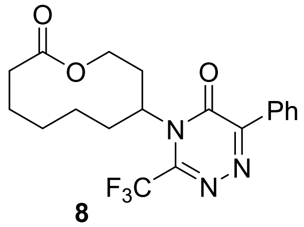

8

^{1}H NMR (400 MHz, $CDCl_3$) and ^{13}C NMR (100 MHz, $CDCl_3$) spectra of product **9**

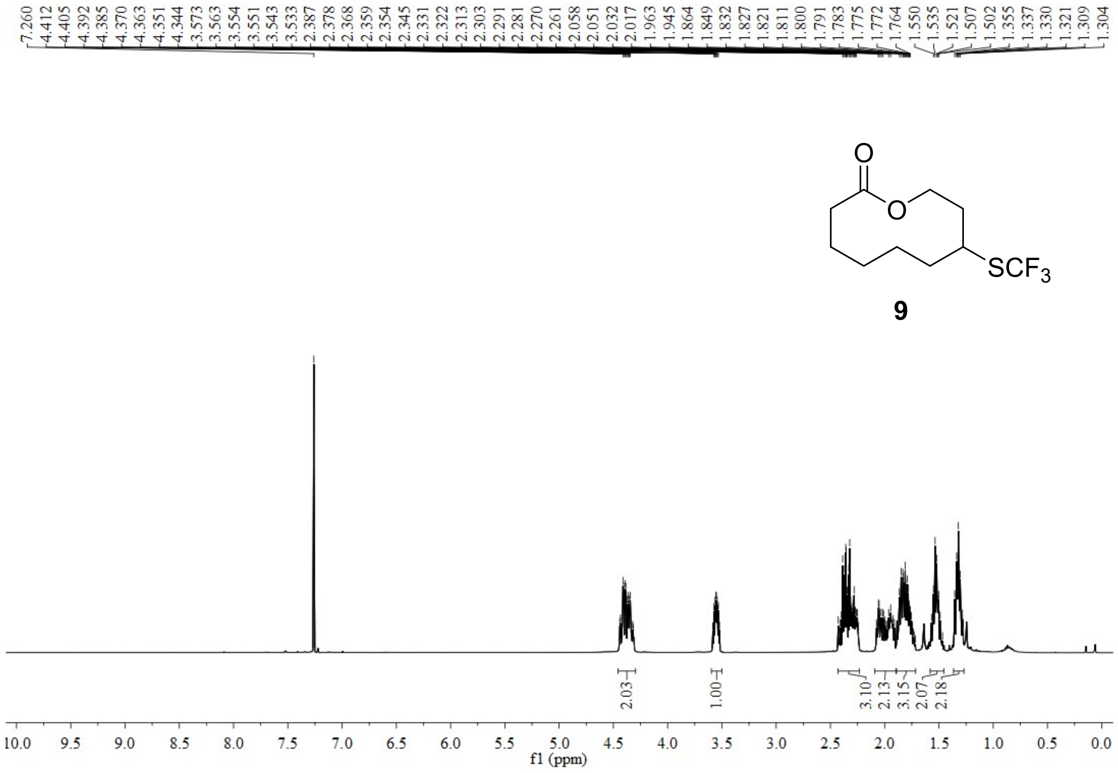

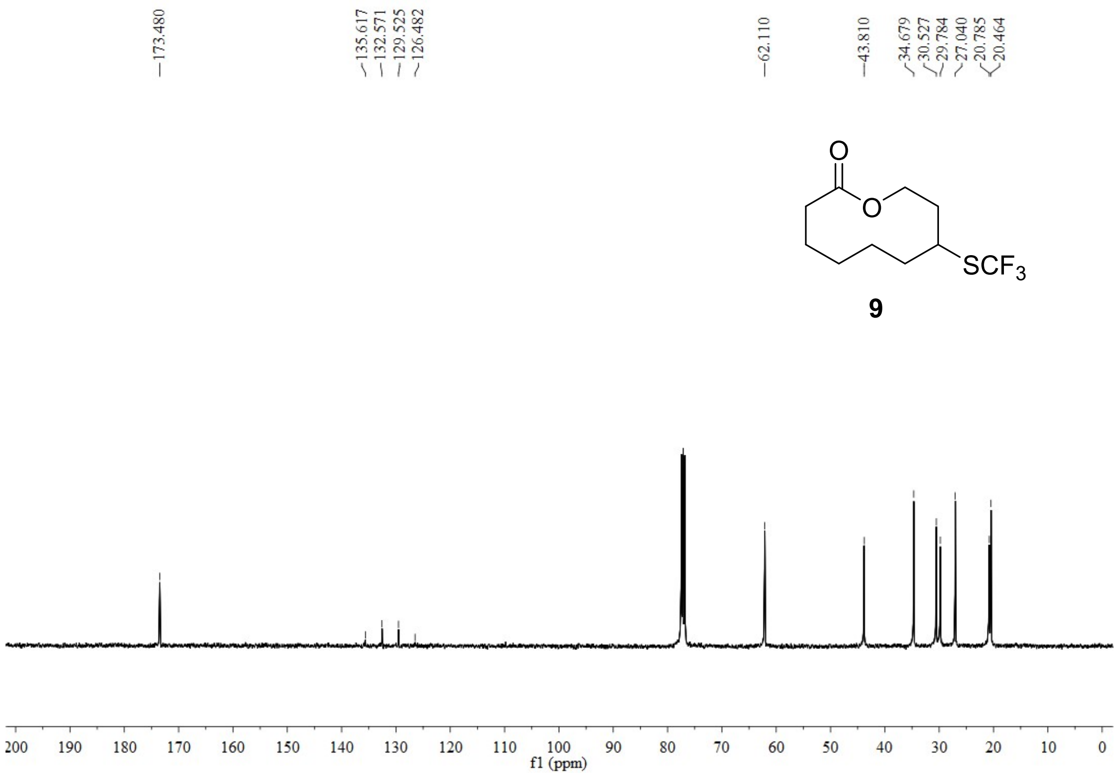

^{19}F NMR (376 MHz, $CDCl_3$) spectra of product **9**

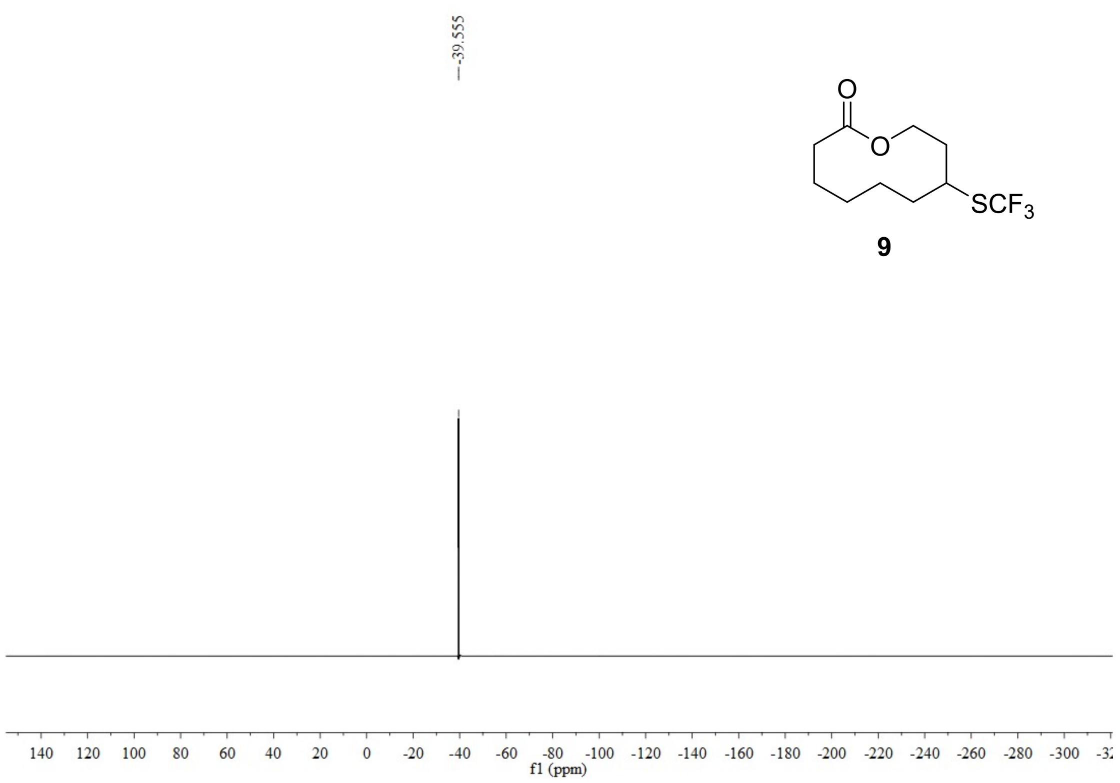

^{1}H NMR (400 MHz, $CDCl_3$) and ^{13}C NMR (100 MHz, $CDCl_3$) spectra of product **10**

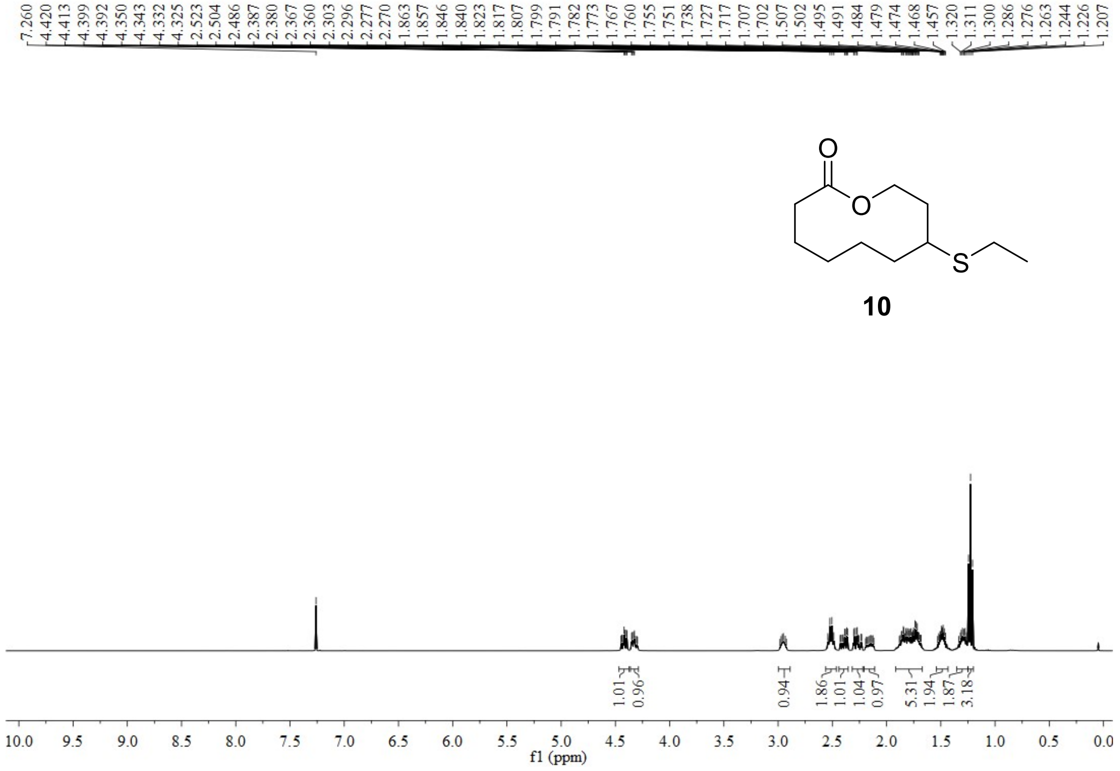

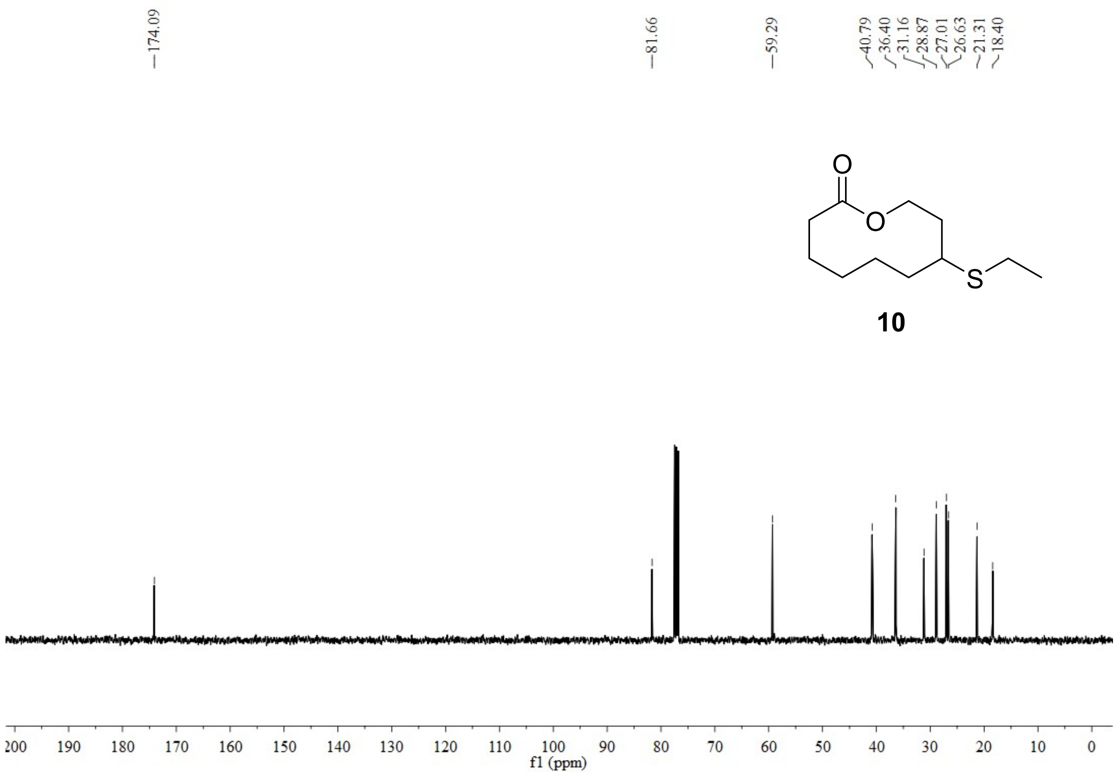

^{1}H NMR (400 MHz, $CDCl_3$) and ^{13}C NMR (100 MHz, $CDCl_3$) spectra of product **11**

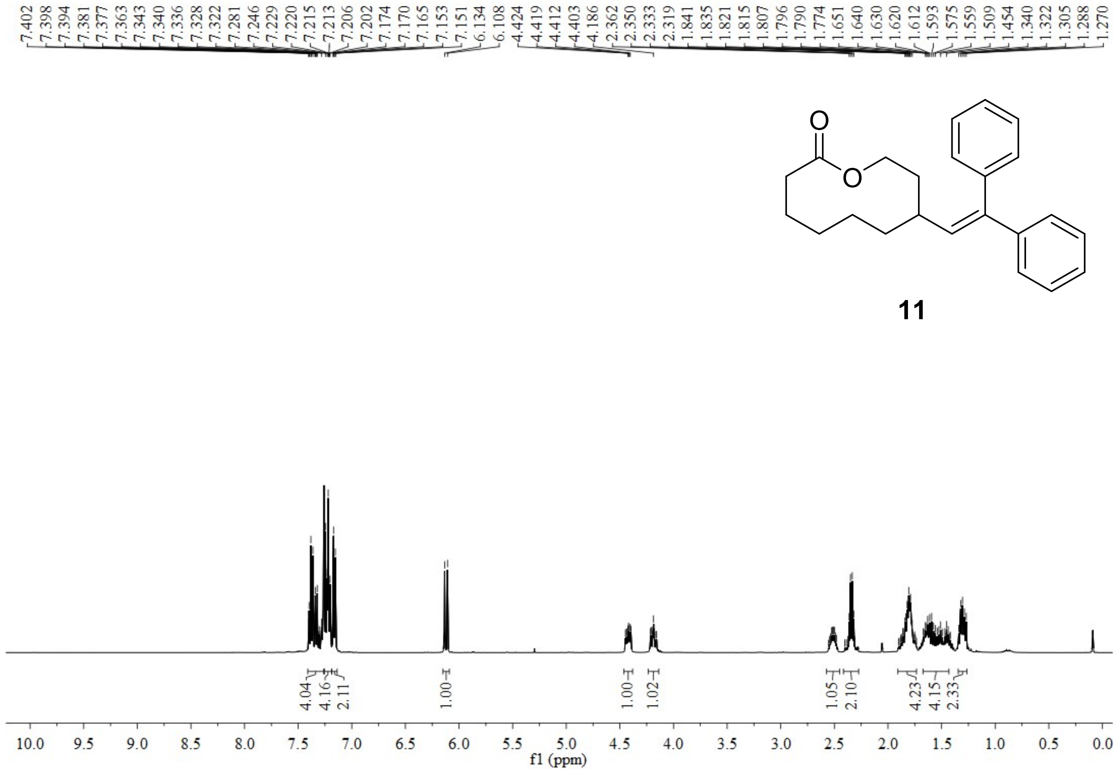
7.402 7.398 7.394 7.381 7.377 7.363 7.343 7.340 7.336 7.328 7.322 7.281 7.246 7.229 7.220 7.215 7.213 7.206 7.202 7.174 7.170 7.165 7.153 7.151 6.134 6.108 4.424 4.419 4.412 4.403 4.186 2.362 2.350 2.333 2.319 1.841 1.835 1.821 1.815 1.807 1.796 1.790 1.774 1.651 1.640 1.630 1.620 1.612 1.593 1.575 1.559 1.509 1.454 1.340 1.322 1.305 1.288 1.270
O
O
11
4.04 4.16 2.11 1.00 1.00 1.02 1.05 2.10 4.23 4.15 2.33
10.0 9.5 9.0 8.5 8.0 7.5 7.0 6.5 6.0 5.5 5.0 4.5 4.0 3.5 3.0 2.5 2.0 1.5 1.0 0.5 0.0
f1 (ppm)

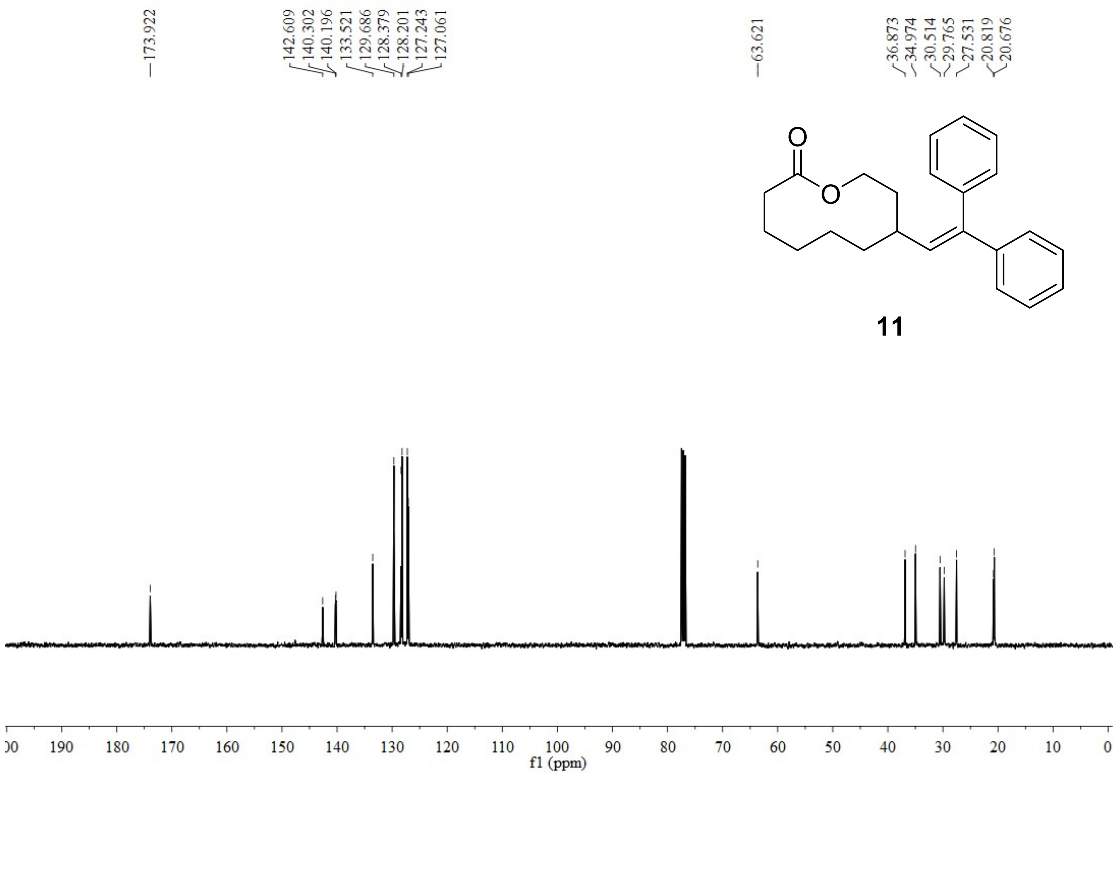
173.922
142.609 140.302 140.196 133.521 129.686 128.379 128.201 127.243 127.061
63.621
36.873 34.974 30.514 29.765 27.531 20.819 20.676
O
O
11
190 180 170 160 150 140 130 120 110 100 90 80 70 60 50 40 30 20 10 0
f1 (ppm)
